# Phylogenomic Resolution of Paleozoic Divergences in Harvestmen (Arachnida, Opiliones) via Analysis of Next-Generation Transcriptome Data

**DOI:** 10.1371/journal.pone.0042888

**Published:** 2012-08-24

**Authors:** Marshal Hedin, James Starrett, Sajia Akhter, Axel L. Schönhofer, Jeffrey W. Shultz

**Affiliations:** 1 Department of Biology, San Diego State University, San Diego, California, United States of America; 2 Department of Biology, University of California Riverside, Riverside, California, United States of America; 3 Department of Computer Science, San Diego State University, San Diego, California, United States of America; 4 Department of Entomology, University of Maryland, College Park, Maryland, United States of America; Field Museum of Natural History, United States of America

## Abstract

Next-generation sequencing technologies are rapidly transforming molecular systematic studies of non-model animal taxa. The arachnid order Opiliones (commonly known as “harvestmen”) includes more than 6,400 described species placed into four well-supported lineages (suborders). Fossil plus molecular clock evidence indicates that these lineages were diverging in the late Silurian to mid-Carboniferous, with some fossil harvestmen representing the earliest known land animals. Perhaps because of this ancient divergence, phylogenetic resolution of subordinal interrelationships within Opiliones has been difficult. We present the first phylogenomics analysis for harvestmen, derived from comparative RNA-Seq data for eight species representing all suborders. Over 30 gigabases of original Illumina short-read data were used in *de novo* assemblies, resulting in 50–80,000 transcripts per taxon. Transcripts were compared to published scorpion and tick genomics data, and a stringent filtering process was used to identify over 350 putatively single-copy, orthologous protein-coding genes shared among taxa. Phylogenetic analyses using various partitioning strategies, data coding schemes, and analytical methods overwhelmingly support the “classical” hypothesis of Opiliones relationships, including the higher-level clades Palpatores and Phalangida. Relaxed molecular clock analyses using multiple alternative fossil calibration strategies corroborate ancient divergences within Opiliones that are possibly deeper than the recorded fossil record indicates. The assembled data matrices, comprising genes that are conserved, highly expressed, and varying in length and phylogenetic informativeness, represent an important resource for future molecular systematic studies of Opiliones and other arachnid groups.

## Introduction

Animal molecular systematics is in the midst of transformation. Until very recently, studies of non-model taxa have relied upon a “PCR + Sanger Sequencing” strategy for a frustratingly small set of genes representing a minute fraction of the genome. These genes were for the most part “useable” rather than optimal (e.g., spread across the genome, evolving at different rates, easy to align, etc.). Various next-generation sequencing (NGS) technologies [1], [2] are rapidly changing this landscape, allowing for the comparative collection of partial or entire genomic data, even for non-model taxa. As proof of concept, Hittinger et al. [3] used Illumina short-read technology to generate comparative cDNA data (RNA-Seq [4]) for ten *Anopheles* mosquito species. These authors showed that large, highly-expressed, shared gene sets (>100 orthologous genes) could be consistently recovered using this strategy, and that these gene sets enabled robust phylogenomic analysis. This NGS-facilitated phylogenomics approach has also been extended to deeper phylogenetic levels in animals (e.g., in mollusks [5], in myzostomid worms [6]). As NGS data becomes less expensive, systematists working on non-model taxa will continue to scale up the taxonomic breadth of comparative phylogenomic datasets. Also, these comparative datasets provide glimpses of previously unknown genomes, facilitating downstream marker development, SNP discovery, and targeted NGS approaches [7]–[9].

The chelicerate arthropod class Arachnida (e.g., scorpions, spiders, mites, etc.) is rich in taxonomic diversity (eleven traditional extant orders, ∼97,000 described species [10]), but poorly characterized from a comparative genomics perspective. Complete genomes are currently only available for the Order Acari (mites and ticks; *Rhipicephalus (Boophilus) microplus* [11], *Ixodes scapularis* [12]), although various scorpions and spiders have been the subject of traditional EST or more recent RNA-Seq studies [13]–[16]. The order Opiliones is the third largest arachnid clade, with 46 families, approximately 1,500 genera, and more than 6,400 described species [17], [18]. Commonly known as harvestmen, Opiliones taxa are conspicuous members of nearly all terrestrial communities, and present compelling but generally under-appreciated opportunities for studies of ecology, sexual selection, historical biogeography, etcetera [19]. To date, large-scale studies of the nuclear genomes of harvestmen have not been conducted.

**Figure 1:**
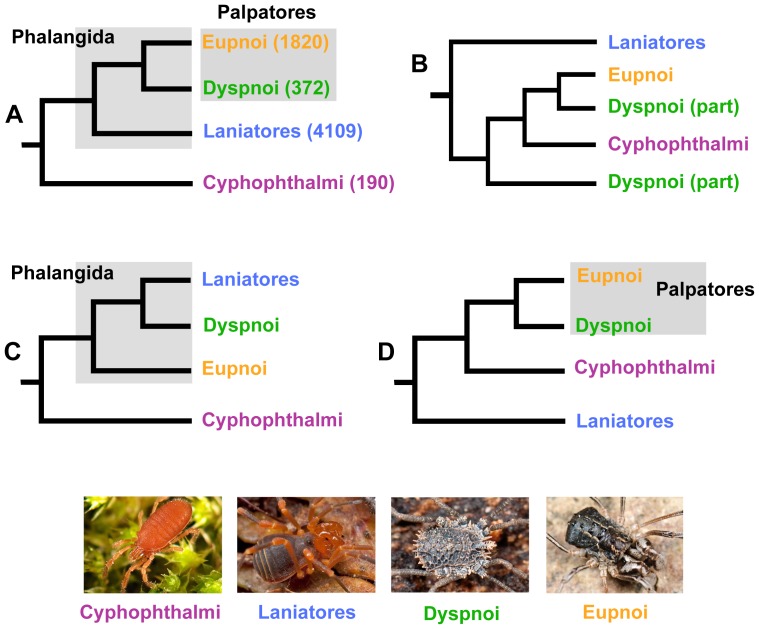
Alternative phylogenetic hypotheses for Opiliones. A) Classical higher-level phylogeny for Opiliones. Number of described species per clade from Kury [18]. B) Cyphopalpatores hypothesis. C) Dyspnolaniatores hypothesis. D) Laniatores basal hypothesis. For details see Table 1. E-H Representative live animals: E) *Siro* (Cyphophthalmi) F) *Sclerobunus* (Laniatores) G) *Ortholasma* (Dyspnoi) H) *Protolophus* (Eupnoi). Images by M. Hedin.

Two phylogenetic facts regarding Opiliones seem clear – the order appears monophyletic, and is comprised of four primary clades (Figure 1). Opiliones monophyly is supported by molecular phylogenetic studies [20], and several hypothesized morphological synapomorphies (e.g., prosomal defensive glands, direct sperm transfer organ, etc. [21], [22]). The four primary harvestmen clades (suborders) include the Cyphophthalmi, Eupnoi, Dyspnoi, and the Laniatores. Phylogenetic support for the monophyly of these individual clades is summarized in Giribet and Kury [20].

There are several deep-level phylogenetic problems in harvestmen that are still unresolved to varying degrees. The arachnid sister group to Opiliones remains elusive [23]. Morphological characters suggest that harvestmen are either sister to scorpions, or sister to a larger clade that includes scorpions, pseudoscorpions, and solifugids. Recent molecular phylogenetic and phylogenomic analyses generally fail to resolve the placement of harvestmen, and do not support a close Opiliones plus Scorpiones relationship [24]–[26]. Within Opiliones there is uncertainty regarding interrelationships of the four primary lineages, particularly in placement of the root, and whether Eupnoi and Dyspnoi together form a clade called Palpatores. The “classical” hypothesis of Hansen and Sørensen [27] is that Cyphophthalmi is sister to other suborders (together called Phalangida), and that Palpatores is monophyletic (Figure 1A). This topology is the most commonly supported hypothesis in recent modern analyses (Table 1). However, the early qualitative work of Martens and co-workers [28]–[30] differs considerably from this perspective (Figure 1B, Table 1), and there are multiple subsequent quantitative phylogenetic studies that suggest alternatives to the classical view (Figure 1C, 1D; Table 1). These alternatives do not appear to strictly hinge upon data type (i.e., molecules versus morphology) or analytical method (e.g., parsimony versus model-based approaches).

**Table 1:** Summary of subordinal phylogenetic hypotheses for Opiliones.

	Author	Data/Analysis	Comments
**Classical Hypothesis**	Hansen and Sorensen [27]	Morphology; non-cladistic	
	Shultz [75]	Morphology; parsimony	
	Giribet et al. [76]	rRNA; parsimony	
	Shultz and Regier [77]	EF1alpha, POL II; parsimony, maximum likelihood (ML)	No outgroups
	Giribet et al. [37]	5 genes; ML	
	Hedin et al. [78]	EF1alpha; parsimony	No outgroups
	Sharma and Giribet [35]	10 genes; ML, Bayesian	no outgroups; 4 mitochondrial genes
	Garwood et al. [33]	Combined rRNA and morphology; Bayesian	
**Cyphopalpatores Hypothesis**	Martens [29], [30]	Genital morphology; non-cladistic	
**Dyspnolaniatores Hypothesis**	Giribet et al. [76]	Combined rRNA and morphology; parsimony	
	Giribet et al. [79]	rRNA, morphology, combined; parsimony	
	Garwood et al. [33]	Combined rRNA and morphology; direct optimization parsimony	weakly supported (Fig. 3)
**Laniatores Basal Hypothesis**	Giribet et al. [37]	5 genes; direct optimization parsimony	weakly supported (Fig. 5)
	Garwood et al. [33]	rRNA; Bayesian	weakly supported

Opiliones suborders are ancient groups, and as such, phylogenetic resolution of subordinal interrelationships might be expected to be difficult. The oldest known fossil harvestmen (*Eophalangium*) from the Devonian Rhynie Chert (∼410 Myr) represents one of the earliest known land animals [31], [32]. This long-legged fossil has been classified as a Eupnoi based mostly on genital morphology, but a phylogenetic analysis has never been conducted. Recently, formal combined evidence phylogenetic analyses have placed well-preserved Carboniferous (∼305 Myr) harvestmen as members of Dyspnoi (*Ameticos*) and Eupnoi (*Macrogyion*) with reasonably high confidence [33]. The fossil record for Cyphophthalmi and Laniatores does not include Paleozoic forms [34], but molecular clock analyses suggest ancient ancestry. Relaxed clock BEAST analyses of nucleotides using an uncorrelated lognormal model estimate a most recent common ancestor for Laniatores at ∼350 Myr (95% highest posterior density (HPD) 307–382 [35]), and a most recent common ancestor for Cyphophthalmi at ∼330 Myr (95% HPD 297–362 [36]). Taken together, fossil plus molecular clock evidence indicates that land-dwelling Opiliones suborders were diverging in the late Silurian to mid-Carboniferous time interval [37].

Although previous phylogenetic studies in Opiliones have used mitochondrial, rRNA, and a handful of nuclear protein-coding genes, a phylogenomic analysis has never been conducted. Phylogenomics-scale data are expected to help resolve ancient, relatively rapid diversification events in the tree of life, although many analytical caveats exist [38]. Here we present the first phylogenomics analysis for Opiliones, derived from comparative Illumina RNA-Seq data for a taxon set representing all four primary harvestmen lineages.

## Materials and Methods

### Taxon Sample, RNA-Seq

None of the exemplar species used are of conservation concern, and none were collected on lands requiring special collecting permits. Our taxon sample includes representatives of all four primary harvestmen suborders: Cyphophthalmi (represented by *Siro*), Eupnoi (*Leiobunum* and *Protolophus*), Dyspnoi (*Ortholasma*, *Trogulus*, and *Hesperonemastoma*), and Laniatores (*Sitalcina* and *Sclerobunus*). The Dyspnoi sample includes representatives of the two primary Dyspnoi lineages (Ischryopsalidoidea and Troguloidea [28]), as does the Laniatores sample (“Insidiatores” and Grassatores [35]). The Eupnoi sample includes representatives of only the Phalangioidea, but not the primary sister lineage Caddoidea. Additional taxonomic information is provided in Table 2.

**Table 2:** Taxonomic and RNA Extraction Information.

Suborder	Species	Geographic Origin	Sex, No. Individuals, Tissues
Cyphophthalmi	*Siro acaroides* (Ewing, 1923)	west of Wemme, Clackamas County, Oregon, USA	8 adults (sex unknown) - entire
Eupnoi	*Protolophus singularis* Banks, 1893	near Guatay, San Diego County, California, USA	1 male cephalothorax
“	*Leiobunum verrucosum* (Wood, 1870)	near Damascus, Montgomery County, Maryland, USA	2 immatures –midgut removed
Dyspnoi	*Ortholasma coronadense* Cockerell, 1916	San Diego, San Diego County, California, USA	1M, 2F –midgut removed
“	*Trogulus martensi* Chemini, 1983	Unteruhldingen, Baden-Wurttemberg, Germany	2 adults (sex unknown) – one entire, one midgut removed
“	*Hesperonemastoma modestum* (Banks, 1894)	Palomar Mountain, San Diego County, California, USA	10 adults (sex unknown) - entire
Laniatores	*Sitalcina lobata* Goodnight and Goodnight, 1942	near Julian, San Diego County, California, USA	3M, 4F - entire
“	*Sclerobunus nondimorphicus* Briggs, 1971	near Rhododendron, Clackamas County, Oregon, USA	Two adults (sex unknown) –midgut removed

Specimens destined for RNA extractions were either sacrificed immediately prior to extraction, or preserved as fresh specimens in cold RNAlater, then extracted later. We sought “general purpose” transcriptomes, and typically extracted from whole animals, sometimes combining several small specimens in a single extraction (Table 2). Total RNA was extracted using TRIzol (Invitrogen, Carlsbad, CA) and the RNeasy Minikit (Qiagen, Valencia, CA). *Protolophus* total RNA was shipped to Cofactor Genomics (www.cofactorgenomics.com), where a duplex-specific nuclease (DSN) normalized library was constructed, and 60 basepair (bp) paired-end reads were generated using Illumina GAII technology. All other RNA samples were sent to the Genomic Services Lab at the HudsonAlpha Institute for Biotechnology (www.hudsonalpha.org), where non-normalized libraries were prepared using the Illumina TruSeq RNASeq kit, and sequenced as 50-bp paired-end reads using Illumina HiSeq technology.

### *de Novo* Assembly

Data for *Protolophus*, *Sitalcina*, and *Ortholasma* were passed through an Illumina chastity filter, but were otherwise not filtered prior to *de novo* assembly. The program FastQC [39] was used to confirm data quality prior to assembly for these taxa. Fastq files for all other taxa were filtered for low-quality reads prior to *de novo* assembly using a custom Python script. *De novo* assemblies of paired-end data were constructed with Oases v 0.1.18 [40], which uses as input preliminary assemblies produced by Velvet v1.1.05 [41]. A k-mer length of 31 was used for all assemblies. Gene transcripts (contigs) resulting from assemblies were given taxon-specific names in text files, then all transcripts were combined into a single “eight harvestmen transcript” file. Oases sometimes provides alternative assemblies for the same putative transcript; these redundant transcripts were retained at this stage of analysis.

### SCORP - Matrices with Scorpion & Ixodes as Outgroups

Nucleotide data from three different scorpion venom EST projects (*Heterometrus*, [14]; *Hadrurus* [13]; *Lychas* [42]) and a targeted Sanger sequencing phylogenomics project (*Hadrurus* [24]) were retrieved from GenBank. FASTA files from these projects were combined into a single file, which was searched locally against the eight harvestmen transcript file using tblastx (max hits = 20, max e value 1e-10) in Geneious Pro v5.4 [43]. Searches resulting in short regions of overlap (<200 bp) or apparent paralogy (multiple divergent gene transcripts from the same harvestmen taxon, for one or more taxa) were discarded. Genes were only retained if *Siro*, and at least one taxon representing Laniatores, Dyspnoi and Eupnoi, were present for the target gene region. For promising candidate genes, the scorpion and *Siro* sequences were searched locally against the tick (*Ixodes*) peptide set (blastx, max hits = 2, max e value 1e-10), downloaded from VectorBase (http://iscapularis.vectorbase.org/). *Ixodes* sequences were retained if both *Siro* and scorpion matched the same sequence. Nucleotide data were initially aligned using the Translation alignment tool in Geneious Pro (12∶3 gap open to gap extension penalty ratio). Non-coding data 5′ of start codons, and 3′ of stop codons (not always present in partial scorpion gene fragments) were trimmed from matrices, and matrices were re-aligned using the Geneious Pro MAFFT v6.814b plug-in [44] with default parameter settings. Assembled transcriptomes for some taxa (*Protolophus* in particular, see Results) were fragmented for certain genes – i.e., represented by multiple transcripts arranged in an adjacent or minimally overlapping manner for a single putative ortholog. If orthology seemed apparent (e.g., for *Protolophus* we expected high similarity to *Leiobunum* sequences and identical *Protolophus* sequence in regions of transcript overlap), these multiple different transcripts were merged into a single contiguous “super-transcript” for that taxon [3]. Matrices were exported to EXCEL to check that harvestmen transcripts were not represented more than once in the SCORP gene set.

### Tick - Matrices with *Ixodes* only as Outgroup

Annotations available at VectorBase were used to classify *Ixodes* proteins into the following categories: single copy, single exon proteins (SCSE, n = 507); proteins with introns but no annotated paralogs (IntNoPar, n = 3286); proteins with a single annotated paralog (SPar, n = 1160). This reduced *Ixodes* protein set was searched locally against the eight harvestmen transcript file (tblastn, max hits = 20, max e value 1e-10). A strict filter was applied to retain candidate proteins: 1) only proteins represented in all 8 harvestmen transcriptomes were retained; 2) genes or gene regions less than 300 bp were discarded; 3) proteins with evidence for paralogy (multiple divergent transcripts for the same region for a single taxon) in one or more taxa were discarded; 4) harvestmen proteins with high similarity, but conspicuously longer reading frames than in *Ixodes*, were discarded; 5) difficult-to-align proteins (>5% gaps in tblastn alignments) were discarded; 6) complete proteins with both start and stop codons in harvestmen were preferred.

As for the SCORP matrices, nucleotide data were initially aligned using the Translation alignment tool in Geneious Pro, terminal non-coding data were trimmed, and matrices were re-aligned using MAFFT. In some cases “super-transcripts” were merged (see Results). Matrices were exported to EXCEL to check that harvestmen transcripts were not represented more than once in the TICK gene set, and were not also found in the SCORP gene set. We discontinued the above process after finding 300 suitable and unique TICK proteins.

### Phylogenomics

The 300 TICK proteins were subdivided into 3 separate 100-gene bins based on relative rates of evolution [45], using average pairwise nucleotide divergence as a measure of such rate variation (calculated in Geneious Pro). Five total concatenated nucleotide data matrices (SCORP, TICKFAST, TICKMED, TICKSLOW, TICKALL) were analyzed unpartitioned and with codon partitioning (i.e., 3 total partitions), using RAxML version 7.2.8 [46]. A GTRGAMMA model was used for each partition, as the GTRCAT model is not recommended for matrices with so few taxa [47]. Multiple ML searches (100) were conducted per matrix, each using a randomized stepwise addition parsimony tree to initiate an ML tree inference. Non-parametric bootstrap replicates (500) were conducted on each matrix using a partitioned GTRGAMMA model. Bootstrap values were visualized on the best-scoring ML tree derived from multiple ML searches.

The five DNA matrices were also translated to amino acids for phylogenetic analysis, with stop codons recoded as missing data. For each concatenated amino acid matrix a single best-fit model of protein evolution was chosen using ProtTest v2.4 [48], for a subset of models also available in RAxML. As above, multiple ML searches (100) and non-parametric bootstrap replicates (500) were conducted per amino acid matrix. Bootstrap values were visualized on the best-scoring ML tree from multiple inferences.

We also conducted a Bayesian phylogenetic analysis of the TICKALL protein matrix using PhyloBayes version 3.3 [49]. PhyloBayes can implement a site-heterogeneous Bayesian mixture model (CAT) that allows site-specific rates for different amino acid positions in a protein alignment [50]. The CAT model has been shown to better capture the complexities of protein evolution, and is more robust to long-branch attraction than standard site-homogeneous protein models [50], [51]. Using a default CAT-Poisson model, two Monte Carlo Markov chains were run in parallel until the largest discrepancy observed across all bipartitions (maxdiff) was lower or equal to 0.3, and effective sizes for all summary variables exceeded 50. A majority-rule posterior consensus tree was reconstructed from a set of post burn-in trees (discarding 20% as burn-in).

### Divergence Time Estimation

The MCMCtree Bayesian program in the PAML v. 4.4c package [52], [53] was used to estimate harvestmen divergence times using a relaxed molecular clock. Analyses were conducted on nucleotide data from the TICKALL matrix, partitioned by codon position, without *Ixodes* outgroup sequences. The tree topology was constrained to ingroup relationships recovered in RAxML and PhyloBayes analyses of TICKALL nucleotides and amino acids (see Results). Priors for lineage-specific substitution rates were specified using autocorrelated and independent rates models [54]–[56]. Both of these analyses were run with and without sequence data. We estimated the parameters (shape and scale) of the gamma distribution of the overall substitution rate prior (μ) using Baseml estimates of the substitution rate for each partition. The substitution rate prior (μ) was estimated under three calibration schemes (see below); each calibration scheme used three fixed calibration points. The shape and scale for μ were 0.42 and 1.31, 0.34 and 0.13, and 0.40 and 1.17, under calibrations schemes 1, 2, and 3, respectively. The gamma distributed prior for σ^2^, which specifies how variable the substitution rate is among branches, was set to shape = 1 and scale = 1. The analysis was run with birth rate, death rate, and species sampling priors of 2, 2, and 0.1, respectively. The HKY sequence model was used and gamma priors for κ (the transition/transversion ratio) and α (shape parameter for among site rate variation) were left as default [54]. Calibrations (see below) were treated as soft boundaries (i.e., 2.5% chance date falls beyond boundary [55], [57]). The first 20,000 iterations were discarded as burnin, followed by 50,000 iterations sampled every five iterations. Analyses including sequence data were run twice to assess MCMC convergence.

Three different fossil calibration strategies were used as follows: **1)** We treated the Garwood et al. [33] fossils as ***stem group*** members of Eupnoi (*Macrogyion*) and Dyspnoi (*Ameticos*) respectively, and constrained the minimum age of the common ancestor of the group including these fossils ( = Palpatores) at 305 Myr. We treated the Huang et al. [58] *Mesobunus* fossil (165 Myr) as a crown group phalangioid, and the Giribet and Dunlop [59] *Halitherses* fossil (100 Myr) as a crown group troguloid, constraining the minimum age for these clades. **2)** Garwood et al. [33] fossils were treated as ***crown group*** members of Eupnoi and Dyspnoi, constraining minimum ages for these clades at 305 Myr; *Halitherses* (100 Myr) was treated as a crown group troguloid, constraining the minimum age for this clade. **3)** *Eophalangium* was treated as a ***stem group*** Eupnoi, constraining the minimum age of the common ancestor of the group including these fossils ( = Palpatores) at 410 Myr [31], [32]. *Mesobunus* (165 Myr [58]) was treated as a crown group phalangioid, and *Halitherses* (100 Myr [59]) as a crown group troguloid, constraining minimum ages for these clades. All calibration strategies required “soft” maximum ages for Opiliones, which we set at 500 Myr. This age is older than the oldest known paleontological evidence for terrestrial arachnids (∼430 Myr [60], [61]), and is arguably too old. However, recent molecular clock studies have estimated Cambrian or Pre-Cambrian divergence dates for the common ancestor of arachnids [26], [62], [63]. Schaefer et al. [62] suggest that arachnids were colonizing terrestrial habitats approximately 150 Myr before the earliest known terrestrial animal fossils.

### Phylogenetic Informativeness of Genes

Gene-specific measures of phylogenetic informativeness (PI [64]) were calculated using the online PhyDesign application [65]. PI values attempt to measure the informativeness of a character set (“genes”, either nucleotides or amino acids) in comparison to other character sets, over time intervals relevant to the clade of interest. Informativeness values can be visualized as PI profile curves, where total area and peak curve values provide evidence for differential informativeness over time [64]–[67]. Net and per-site PI values were calculated for 300 individual genes in the TICKALL matrix for both nucleotides and amino acids, excluding *Ixodes* sequences. The chronogram from MCMCtree calibration analysis 1 (replicate 1) was used as an input tree. Nucleotide site rate models were estimated using HyPhy [68], with empirical base frequencies and a time reversible mode of substitution (transitions = 2, transversions = 1). Amino acid site rate models were estimated using rate4site [69] using a JTT model and ML inference method. Similar model settings were recently used in a phylogenetic informativeness analysis of vertebrate genes [70]; crown group vertebrates are likely younger than Opiliones, sharing a common ancestor ∼420 MYA [71].

## Results

### Assemblies

Between 2.5–6 Gb of post-filter Illumina data were used per taxon assembly (Table 3). For non-normalized libraries (all taxa except for *Protolophus*), *de novo* assemblies resulted in approximately 50–80,000 Oases transcripts over 100 bp in length, with mean transcript lengths ranging from 471–694 bp (Table 3). Assembled sequences have been deposited in the NCBI Transcriptome Shotgun Assembly (TSA) database. Assembly of the normalized *Protolophus* library data resulted in many more transcripts of shorter average length (i.e., a more fragmented transcriptome; Table 3). The number of Oases transcripts per taxon is an overestimate of harvestmen transcriptome sizes (because of exogenous sequences, “redundant” Oases transcripts, multiple non-overlapping or partially overlapping transcripts for any single gene, etc.), but full characterization of single harvestmen transcriptomes was not a focus of this paper.

**Table 3:** Raw data and Assembly Information.

*Taxon*	# Illumina PE Reads (post-QCfilter)	# Oases Transcripts	Mean, Max Transcript Length	No. “Super Transcripts” in Phylogenetic Matrices
*Siro*	50341482×50 bp = 2.52 Gb	53,006	477, 6979	12 in 367 (0.0327)
*Sitalcina*	85228254×50 bp = 4.26 Gb	79,075	471, 12417	16 in 367 (0.0436)
*Sclerobunus*	88356268×50 bp = 4.42 Gb	77,342	675, 17875	8 in 367 (0.0218)
*Ortholasma*	80744734×50 bp = 4.04 Gb	50,665	570, 11074	18 in 366 (0.0492)
*Trogulus*	54675768×50 bp = 2.73 Gb	77,444	567, 9614	5 in 366 (0.0137)
*Hesperonemastoma*	120052764×50 bp = 6.00 Gb	68,378	613, 8952	23 in 365 (0.0630)
*Leiobunum*	62470530×50 bp = 3.12 Gb	56,048	694, 11393	12 in 365 (0.0329)
*Protolophus*	74989348×60 bp = 4.50 Gb	282,910	231, 8979	85 in 363 (0.2341)

### Data Matrices & Phylogenomics

Summary information for all individual gene matrices (e.g., gene identities, nucleotide alignment lengths, PI values, average pairwise divergence values, taxon composition, etc.) is available in Table S1; aligned nucleotide matrices are available in Text S1. The final SCORP concatenated matrix included data for 67 unique protein-coding genes, with a total nucleotide alignment length of approximately 32 kb. Nucleotide alignment lengths of TICKFAST, TICKMED and TICKSLOW 100-gene concatenated matrices were approximately 72, 67 and 74 kb, respectively (Text S1). The proportion of gaps and missing data for individual nucleotide matrices is very low (2.8%, 1.8%, 1.3%, and 5.24% for TICKFAST, TICKMED, TICKSLOW and SCORP respectively). When present, we found that alternative Oases transcripts for the same taxon differed only in UTR regions (which were trimmed from matrices); alternative transcripts were thus randomly chosen for inclusion in phylogenetic matrices. The number of super-transcripts used per taxon is low (<5%) for all taxa except for *Protolophus*; approximately 23% of individual gene matrices include a *Protolophus* super-transcript (Table 3, Table S1).

**Figure 2:**
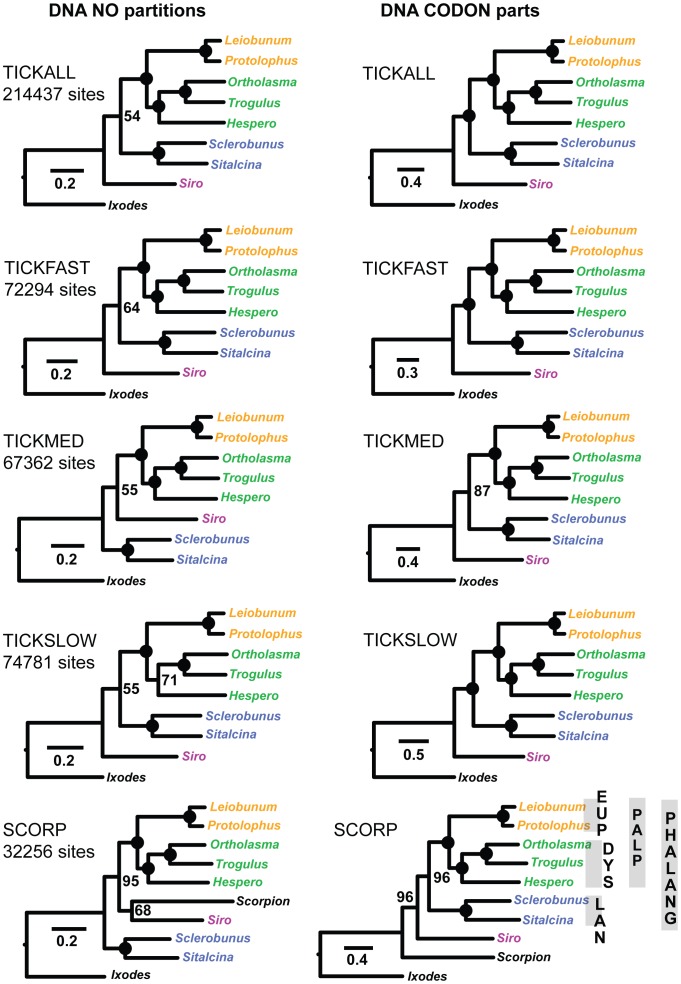
RAxML trees inferred from analyses of nucleotide data. Analyses without codon partitioning on left, analyses with codon partitioning on right. Circles at nodes indicate bootstrap values of 100. Abbreviation for *Hesperonemastoma* = “Hespero”.

RAxML analyses with codon partitioning result in identical topologies, with proportionally similar branch lengths, for all concatenated nucleotide matrices (Figure 2). Across all trees, all but three nodes are supported by bootstrap proportion values of 100. The recovered tree topology includes a monophyletic Eupnoi, Dyspnoi, Palpatores, Laniatores, and Phalangida, consistent with the classical view of Opiliones relationships (Figure 1A). Rooting of the SCORP matrix with *Ixodes* supports Opiliones monophyly (bootstrap = 96). The phylogenetic placement of *Protolophus* is as expected in all analyses (sister to *Leiobunum* in Eupnoi), suggesting that the inclusion of super-transcripts did not negatively impact phylogenetic analyses.

Phylogenetic analyses of unpartitioned concatenated nucleotide matrices strongly support Eupnoi, Dyspnoi, Palpatores and Laniatores (bootstrap = 100), but are less resolved at deeper phylogenetic levels. First, although analyses of TICK matrices recover the classical Opiliones tree, these analyses do not strongly support Phalangida monophyly (bootstrap values less than 70; Figure 2). Second, analysis of the SCORP matrix does not recover Opiliones monophyly, instead suggesting a scorpion + *Siro* relationship (Figure 2). Analyses of the unpartitioned nucleotide matrices with third position sites removed result in trees consistent with the classical view, with bootstrap proportion values at or near 100 for all nodes (Figure S1).

**Figure 3:**
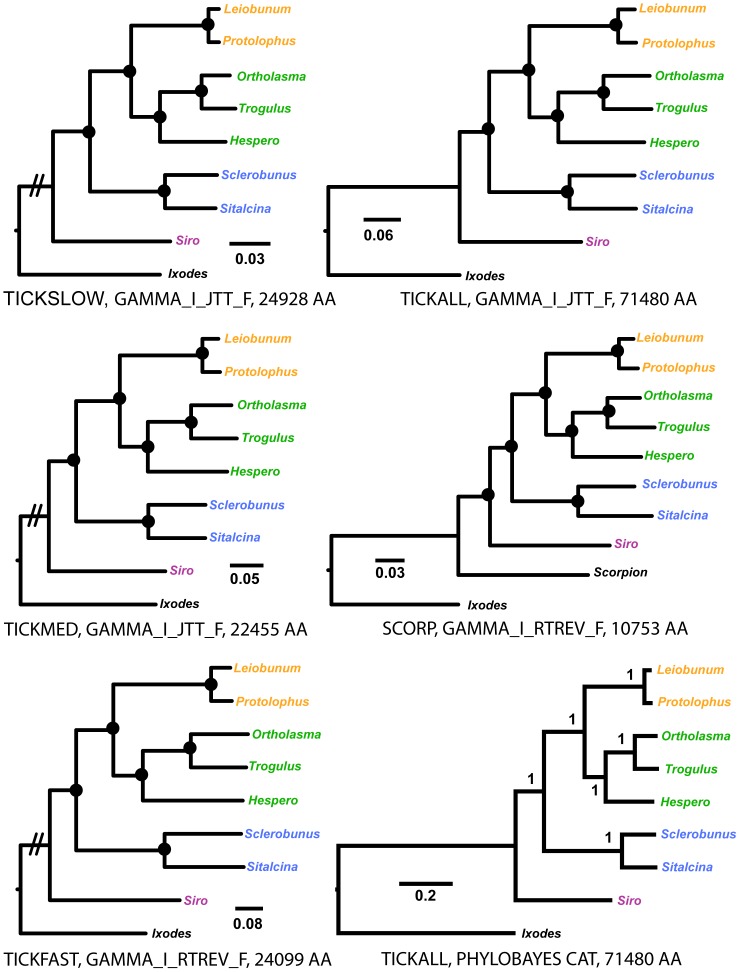
RAxML and PhyloBayes trees inferred from analyses of amino acid data. Root branch for TICKFAST, TICKMED and TICKSLOW trees not drawn to scale. Trees resulting from analysis of SCORP matrix rooted presuming Scorpion plus Opiliones relationship. Circles at nodes indicate bootstrap values of 100. Abbreviation for *Hesperonemastoma* = “Hespero”.

Figure 3 shows best-fit models of protein evolution for individual concatenated amino acid matrices. For any given matrix, a single best-fit model was consistently preferred using the multiple criteria available in ProtTest (e.g., AIC, BIC, etc. [48]). RAxML analyses of the amino acid matrices result in identical topologies, with similar branch lengths, for all data partitions (Figure 3). All nodes for all trees are supported by bootstrap values of 100. The recovered tree topology is consistent with codon-partitioned nucleotide analyses, and the classical view of Opiliones relationships (Figure 1A). Rooting of the SCORP matrix with *Ixodes* supports Opiliones monophyly (bootstrap = 100). PhyloBayes analysis of the TICKALL amino acid matrix implementing the CAT model recovers the classical tree, with posterior probability values of 1.0 at all nodes (Figure 3).

### Comparative Gene Ontologies

Representative nucleotide sequences (usually *Ixodes*, scorpion for 9 SCORP matrices where *Ixodes* was unavailable) for four separate matrices (SCORP, TICKFAST, TICKMED, TICKSLOW) were imported into Blast2GO V.2.5.0 (http://www.blast2go.com [72]) for bioinformatic functional annotation (see Text S2). Matrix-specific GO terms for biological process and molecular function are provided in Table S2. Consistent with the observations of Hittinger et al. [3], our RNA-Seq derived phylogenomic matrices appear dominated by core metabolic genes.

### Divergence Time Estimation

**Figure 4:**
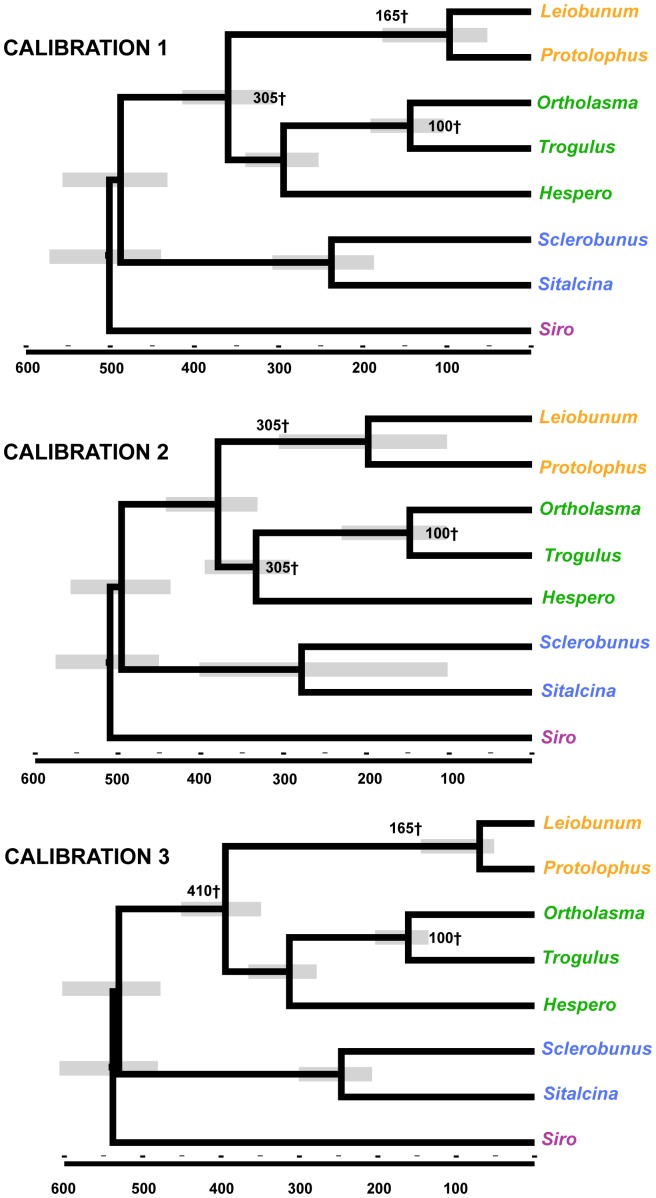
MCMCtree chronograms. MCMCtree chronograms resulting from three alternative calibration schemes (replicate one) using autocorrelated rates model. Scale in millions of years. Fossil calibrations (treated as soft minimums) indicated by cross symbols. Soft maximum of 500 Myr for the root used in each analysis (see Material and Methods). Abbreviation for *Hesperonemastoma* = “Hespero”.

Here we focus on results of MCMCtree analyses under the autocorrelated rates model; results from independent rates model analyses are provided in Text S3. In general, these alternative analyses imply generally similar nodal times estimates (Text S3). Autocorrelated rates chronograms resulting from alternative calibration strategies are shown in Figure 4. Although replicate MCMCtree analyses result in similar most recent common ancestor (MRCA) time estimates for calibration strategies 1 and 3, this was not the case for calibration strategy 2, where MRCA divergence time estimates were more variable over replicates, particularly for the MRCA of Eupnoi (Table 4). Confidence intervals for the MRCA of Eupnoi and Laniatores are also conspicuously large for calibration strategy 2 (Figure 4, Table 4). Time estimates for nodes that are unconstrained in two or more analyses are reasonably consistent. Calibration strategies 1 and 3 suggest a MRCA for Dyspnoi at ∼300 Myr. All calibration strategies suggest a mean MRCA estimate for Laniatores at 220–280 Myr, which is younger than implied by relaxed clock BEAST analyses of Sharma and Giribet (mean estimate of ∼350 Myr, HPD 307–382 [35]). Old fossil calibration minimums within or at the base of Palpatores (for all calibration strategies), in combination with relatively long branches to Laniatores (e.g., Figure 2), imply ancient origins for Phalangida (MRCA mean estimates of 480–530 Myr). These time estimates for Phalangida are older than in previous relaxed molecular clock analyses (mean estimate of ∼415 Myr [37]; mean estimate of ∼427 Myr, HPD 417–438 [35]). Analyses of Giribet et al. [37] were conducted with a fixed root age for Opiliones of 420 Myr.

**Table 4:** MCMC tree results for the autocorrelated rates model.

Calibration	Root	Phalan	Lan	Palp	Eupnoi	Dyspnoi	Trog
Setting 1, run 1	501, 440–567	488, 424–556	237, 185–309	360, 310–413	97, 51–174	294, 253–345	144, 106–186
Setting 1, run 2	499, 438–566	485, 420–554	224, 147–317	360, 310–412	103, 51–178	295, 254–344	146, 106–191
Setting 2, run 1	510, 457–578	496, 440–565	278, 101–398	380, 334–440	198, 97–303	333, 289–398	148, 110–235
Setting 2, run 2	515, 464–584	503, 448–572	267, 191–375	378, 336–432	149, 55–279	319, 280–377	157, 105–223
Setting 3, run 1	538, 483–611	530, 474–603	246, 206–300	394, 351–447	70, 53–145	313, 277–358	161, 136–199
Setting 3, run 2	538, 482–611	530, 472–601	250, 206–317	394, 350–447	75, 53–156	314, 277–362	162, 134–204

**Notes** – time in Myr. Phalan = MRCA of Phalangida, Lan = MRCA of Laniatores, Palp = MRCA of Palpatores, Trog = MRCA of Troguloidea.

### Gene Informativeness

**Figure 5:**
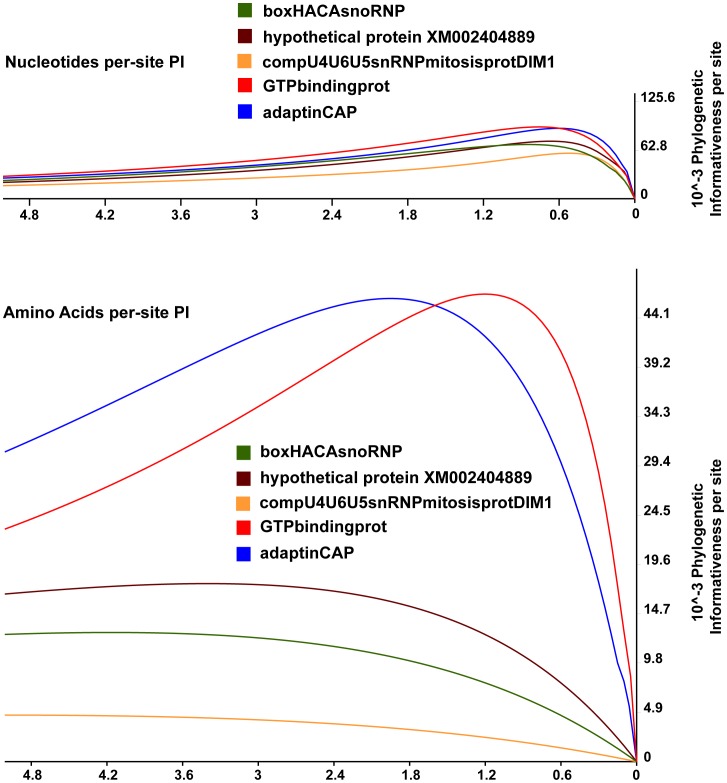
Phylogenetic informativeness profiles. Per-site nucleotide and amino acid phylogenetic informativeness profiles for five exemplar TICKALL genes.

Nucleotide and amino acid PI values for 300 TICKALL genes are provided in Table S1. Summary information for each gene includes the sum of PI values over the entire chronogram time interval (for both net and per-site PI), and the time interval at which PI value is maximized (same for net and per-site PI). Net PI values are confounded by gene length, i.e., longer genes have generally higher net PI values (Table S1), so we focus on per-site values. Nucleotide max PI values are universally smaller (values ranging from 0.50–1.10, i.e., 50–110 Myr) than corresponding amino acid PI values (values ranging from 1.20–5.01), suggesting that amino acids are more informative for older divergences, which is an expected result. The narrow range of nucleotide max PI values in comparison to amino acid values also suggests a greater breadth of informativeness in amino acid characters for the ancient divergences considered here. This greater breadth of informativeness is also suggested by summed PI values for nucleotides versus amino acids (nucleotide ranges 3.3–9.91, amino acid ranges 0.16–12.62). Figure 5 illustrates this difference in informativeness breadth for five representative genes.

## Discussion

One of the primary goals of this phylogenetic study was to resolve the interrelationships of Opiliones suborders. We successfully used comparative RNA-Seq data to assemble large matrices of putatively orthologous genes, and unlike many other phylogenomic studies [73], these matrices include a very low percentage of missing data. Phylogenetic analyses using various partitioning strategies, data coding schemes, and analytical methods overwhelmingly support the “classical” hypothesis of Opiliones relationships (Figures 2 and 3). This hypothesis includes the higher-level clades Palpatores and Phalangida, and is inconsistent with Cyphopalpatores and Dyspnolaniatores hypotheses (Figure 1B, 1C).

As in many phylogenomic studies, our analysis includes many genes and few taxa, and increasing the taxonomic sampling breadth within Opiliones should be a goal for future phylogenomic studies. A potentially more serious shortcoming relates to use of only a single outgroup (tick) in many analyses, and the fact that this outgroup is perhaps too distant from Opiliones. A potential phylogenetic artifact when using a distant outgroup is long branch attraction, as divergent ingroup taxa (e.g., *Siro*) may be pulled to a basal position [38]. We attempted to accommodate this possibility by including scorpion sequences in some analyses, working from the Opiliones plus Scorpiones sister relationship inferred from morphology [23]. Tree topologies within Opiliones are identical for SCORP and TICK amino acid matrices. Even so, it is not clear that Scorpiones represents the best taxon choice for rooting harvestmen trees, as published molecular phylogenetic and phylogenomic studies with sufficient taxon sampling fail to support a close relationship of Opiliones and Scorpiones [24]–[26], [74]. Given the general lack of genomics resources currently available for Arachnida, and the fact that the sister group of Opiliones is effectively unknown at present, we argue that our rooting approach was justified. It could also be argued that all Opiliones outgroups are effectively equivalent given the deep and apparently rapid divergences near the base of Arachnida. A large-scale phylogenomics analysis of all arachnid orders, an obvious target for comparative RNA-Seq phylogenomics, would help to resolve the issues discussed here.

The assembled SCORP and TICK data matrices represent an important resource for future molecular systematic studies of Opiliones and other arachnid groups. The 300 TICK genes were present in all harvestmen transcriptomes, suggesting conservation and high expression levels, a result supported by GO analyses (Table S2). The 300 TICK gene set includes apparently single copy-genes (or if members of gene families, including easily recognizable divergent paralogs) that vary in length and phylogenetic informativeness (Table S1), and are easily aligned. We envision two strategies for future use of this gene set in Opiliones and other arachnid groups. First, retrieving complete or partial gene sets in newly available transcriptome data will greatly streamline downstream phylogenomic analysis. Because of conserved function and high expression levels, we hypothesize that a majority of these genes will be present in most arachnid transcriptomes. Second, some members of these gene sets can be used as resources for a traditional Sanger sequencing strategy, or NGS targeted amplicon sequencing. Proteins that are single copy, single exon genes in *Ixodes* are highlighted in Table S1, and several of these genes are long (over 1 kb). Although this intronless condition is not expected to apply in all arachnids, these proteins are clear candidates for PCR primer design. Overall, we view the gene set assembled here as a resource which allows us to resolve a higher-level phylogenetic framework, and subsequently move towards the tips of the Opiliones tree. This general strategy should be reproducible within and across all arachnid orders.

The early diversification history of Opiliones is enigmatic. The fossil taxon *Eophalangium* is both remarkably old (∼410 Myr) but also surprisingly modern in several aspects of morphology. Authors of the original description suggest that this taxon represents a crown group Eupnoid [31], [32]. At the same time, the earliest known terrestrial arachnid fossils are only about 20 million years older (∼430 Myr [60], [61]), and are among the first known land animals. This paradox implies that Opiliones diversification may have been remarkably rapid, with the radiation of multiple divergent morphological clades (i.e., Cyphophthalmi, Eupnoi, Dyspnoi, Laniatores) occurring over a very short time interval. However, estimated branch lengths from codon-partitioned nucleotide analyses (Figure 2), and all amino acid analyses (Figure 3), appear inconsistent with a rapid radiation scenario at the base of Phalangida or Palpatores.

An alternative explanation is that Opiliones history is deeper than the recorded fossil record indicates, and/or that *Eophalangium* is not as phylogenetically nested as previously hypothesized. Relaxed clock analyses conservatively treating *Eophalangium* as a stem group Eupnoid (Figure 4, calibration 3; Text S3) imply a deep cryptic history for Phalangida and Opiliones, and branch length compression at the base of all chronograms (e.g., Figure 4) suggests that a soft max of 500 Myr for the root of Opiliones may be too recent. Additional analyses treating *Eophalangium* as a stem group Palpatores or Phalangida would be worthwhile, but imply perhaps untenable patterns of morphological character evolution within Opiliones. As hypothesized for mites [62], small omnivorous harvestmen may have been transitioning from marine to terrestrial habitats during the late Cambrian and Ordovician, but are currently unrecorded in the fossil record. An increased phylogenomic sample for Opiliones and other arachnids, in the context of a more densely sampled fossil calibration analysis, is needed to further explore this possibility.

## Supporting Information

Figure S1 **Results** **of analyses of unpartitioned nucleotide matrices with third position sites removed.**
(TIF)

Table S1 **Summary information for individual gene matrices (e.g., gene identities, nucleotide alignment lengths, PI values, average pairwise divergence values, taxon composition, etc.).**
(XLSX)

Table S2 **Gene ontologies.**
(DOCX)

Text S1 **Individual gene matrices.**
(DOCX)

Text S2 **Results** **from gene ontology analysis.**
(DOCX)

Text S3 **Results** **of alternative clock analyses.**
(DOCX)
