# Supplementary figures and images for: Phylogenomic Resolution of Paleozoic Divergences in Harvestmen (Arachnida, Opiliones) via Analysis of Next-Generation Transcriptome Data
Source: PLoS One. 2012 Aug 24;7(8):e42888. doi: 10.1371/journal.pone.0042888 (PMC3427324; doi:10.1371/journal.pone.0042888)

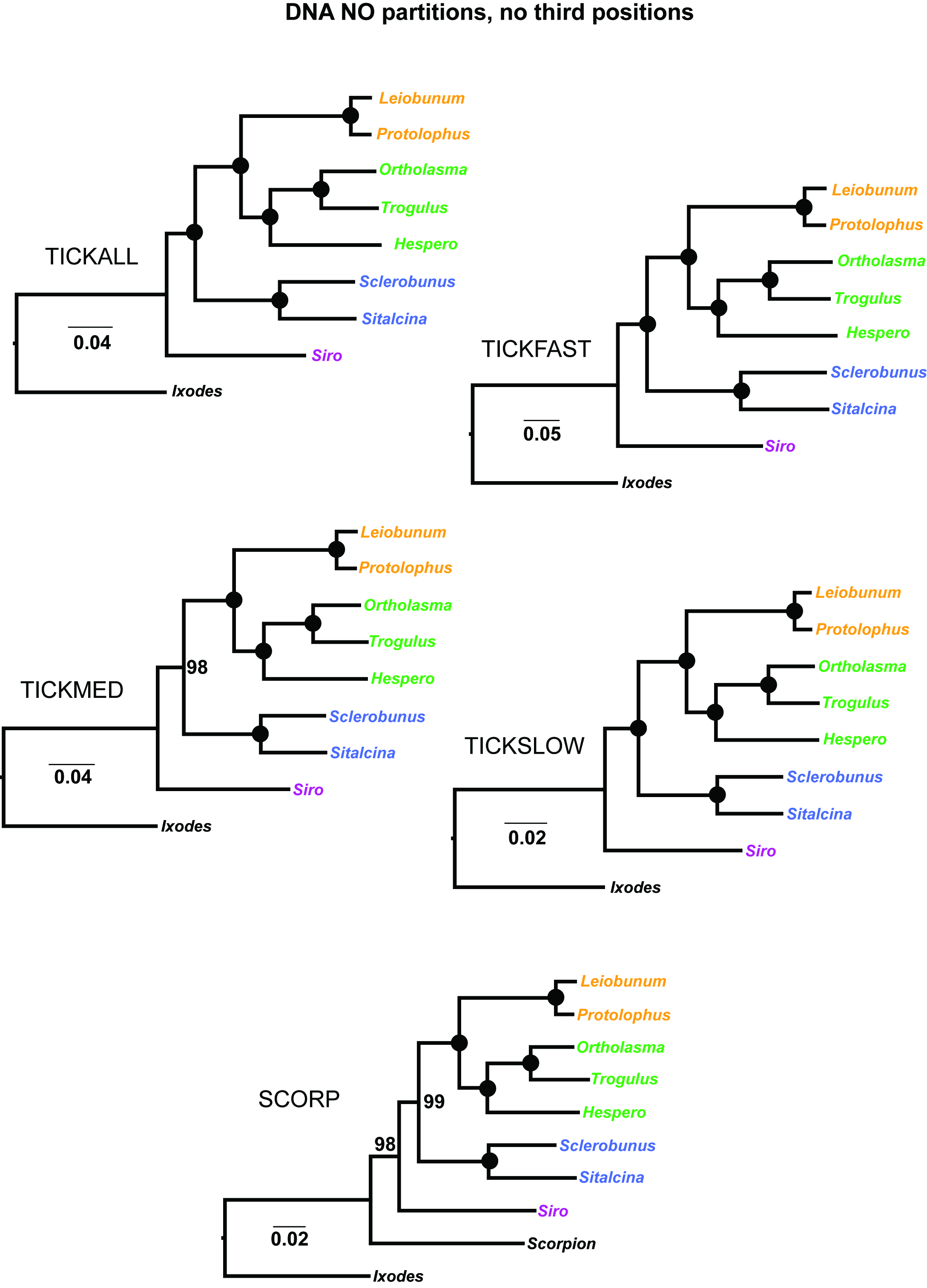

Supplement: Figure S1 — Results of analyses of unpartitioned nucleotide matrices with third position sites removed. (TIF) [file pone.0042888.s001.tif]
