# Supplementary material for: Phylogenomic Resolution of Paleozoic Divergences in Harvestmen (Arachnida, Opiliones) via Analysis of Next-Generation Transcriptome Data
Source: PLoS One. 2012 Aug 24;7(8):e42888. doi: 10.1371/journal.pone.0042888 (PMC3427324; doi:10.1371/journal.pone.0042888)
Supplement: Table S2 — Gene ontologies. (DOCX) [file pone.0042888.s003.docx]

**Table S2**

| **Biological Process** | **ME** | **FA** | **SL** | **SC** |  | **ME** | **FA** | **SL** | **SC** | **Molecular Function** |
| --- | --- | --- | --- | --- | --- | --- | --- | --- | --- | --- |
| GO:0008152 metabolic process | 33 | 39 | 24 | 42 |  | 31 | 28 | 38 | 31 | GO:0005488 binding |
| GO:0009058 biosynthetic process | 10 | 21 | 13 | 22 |  | 8 | 13 | 20 | 15 | GO:0003676 nucleic acid binding |
| GO:0044249 cellular biosynthetic process | 3 | 15 | 10 | 16 |  | 6 | 7 | 12 | 9 | GO:0003723 RNA binding |
| GO:0009059 macromolecule biosynthetic process | 3 | 15 | 10 | 16 |  | 0 | 1 | 4 | 5 | GO:0003677 DNA binding |
| GO:0034645 cellular macromolecule biosynthetic process | 3 | 15 | 10 | 16 |  | 3 | 2 | 7 | 3 | GO:0008135 translation factor activity, nucleic acid binding |
| GO:0006412 translation | 3 | 15 | 10 | 16 |  | 7 | 1 | 0 | 2 | GO:0005515 protein binding |
| GO:0006807 nitrogen compound metabolic process | 8 | 4 | 2 | 8 |  | 8 | 1 | 7 | 15 | GO:0000166 nucleotide binding |
| GO:0009056 catabolic process | 5 | 2 | 3 | 6 |  | 1 | 0 | 0 | 0 | GO:0006630 lipid binding |
| GO:0044237 cellular metabolic process | 14 | 21 | 14 | 29 |  | 0 | 1 | 0 | 0 | GO:0043167 ion binding |
| GO:0044260 cellular macromolecule metabolic process | 5 | 17 | 13 | 20 |  | 0 | 1 | 0 | 0 | GO:0005509 calcium ion binding |
| GO:0006091 generation of precursor metabolites and energy | 2 | 1 | 1 | 6 |  | 0 | 1 | 0 | 0 | GO:0046872 metal ion binding |
| GO:0034641 cellular nitrogen compound metabolic process | 8 | 4 | 2 | 8 |  | 0 | 1 | 0 | 0 | GO:0043169 cation binding |
| GO:000613 nucleic acid metabolic process | 8 | 4 | 2 | 8 |  | 0 | 0 | 1 | 0 | GO:0030246 carbohydrate binding |
| GO:0044267 cellular protein metabolic process | 5 | 17 | 12 | 19 |  | 0 | 0 | 2 | 0 | GO:0019825 oxygen binding |
| GO:0006464 protein modification process | 2 | 2 | 3 | 3 |  | 28 | 27 | 22 | 29 | GO:0003824 catalytic activity |
| GO:0044238 primary metabolic process | 23 | 28 | 16 | 31 |  | 12 | 10 | 11 | 7 | GO:0016787 hydrolase activity |
| GO:0005975 carbohydrate metabolic process | 3 | 3 | 1 | 3 |  | 2 | 1 | 1 | 0 | GO:0016788 hydrolase activity, acting on ester bonds |
| GO:0043170 macromolecule metabolic process | 12 | 21 | 15 | 24 |  | 0 | 1 | 1 | 0 | GO:0004518 nuclease activity |
| GO:0010467 gene expression | 3 | 0 | 10 | 16 |  | 2 | 0 | 0 | 0 | GO:0042578 phosphoric ester hydrolase activity |
| GO:0019538 protein metabolic process | 12 | 21 | 14 | 23 |  | 2 | 0 | 0 | 0 | GO:0016791 phosphatase activity |
| GO:0009987 cellular process | 16 | 23 | 19 | 31 |  | 2 | 0 | 0 | 0 | GO:0004721 phosphoprotein phosphatase activity |
| GO:0016043 cellular component organization | 5 | 3 | 9 | 3 |  | 2 | 2 | 2 | 3 | GO:0008233 peptidase activity |
| GO:0006996 organelle organization | 3 | 2 | 5 | 3 |  | 7 | 9 | 7 | 9 | GO:0016740 transferase activity |
| GO:0065007 biological regulation | 4 | 2 | 6 | 4 |  | 0 | 0 | 2 | 4 | GO:0016772 transferase activity |
| GO:0050789 regulation of biological process | 4 | 2 | 6 | 4 |  | 0 | 0 | 0 | 1 | GO:0016773 phosphotransferase activity, alcohol group as acceptor |
| GO:0051179 localization | 6 | 5 | 9 | 7 |  | 0 | 0 | 2 | 4 | GO:0016301 kinase activity |
| GO:0051234 establishment of localization | 6 | 5 | 9 | 7 |  | 0 | 14 | 4 | 9 | GO:0005198 structural molecule activity |
| GO:0006810 transport | 6 | 5 | 9 | 7 |  | 2 | 1 | 3 | 2 | GO:0005215 transporter activity |
| GO:0045184 establishment of protein localization | 2 | 1 | 3 | 4 |  | 1 | 0 | 2 | 1 | GO:0030234 enzyme regulator activity |
| GO:0015031 protein transport | 2 | 1 | 3 | 4 |  | 0 | 2 | 1 | 0 | GO:0004872 receptor activity |
| GO:0006811 ion transport | 1 | 2 | 1 | 1 |  | 0 | 2 | 1 | 0 | GO:0060089 molecular transducer activity |
| GO:0033036 macromolecule localization | 2 | 1 | 3 | 4 |  | 0 | 2 | 1 | 0 | GO:0004871 signal transducer activity |
| GO:0043412 macromolecule modification | 2 | 2 | 3 | 3 |  | 0 | 1 | 1 | 1 | GO:0009055 electron carrier activity |
| GO:0008104 protein localization | 2 | 1 | 3 | 4 |  |  |  |  |  |  |
| other | 12 GO | 6 GO | 3 GO | 12 GO |  |  |  |  |  |  |

ME = tick medium, FA = tick fast, SL = tick slow, SC = scorpion. “other” = Biological Process GO terms shared by 3 or fewer matrices
