# Supplementary material for: Phylogenomic Resolution of Paleozoic Divergences in Harvestmen (Arachnida, Opiliones) via Analysis of Next-Generation Transcriptome Data
Source: PLoS One. 2012 Aug 24;7(8):e42888. doi: 10.1371/journal.pone.0042888 (PMC3427324; doi:10.1371/journal.pone.0042888)
Supplement: Text S1 — Individual gene matrices. (DOCX) [file pone.0042888.s004.docx]

**Text S1**

[TICKFAST_NCHAR=1068;1-acyl-glycerol-3-phosphate acyltransferase]

Ixodes TTCATGACCAGCGGCCTCATCCTCAACGTCTTCCAACTCCTGGTGTACCTGCTCGTGCGTCCATTCTCGCGGCATTTGTACCGGCGGATCAACTACTATCTCATCTACACTTCTTGGTCCCAGGTGGTGGCCTTGGCCCAGTGGTGGTCCGGTAGCCGCCTGAGGGTTTGGGGCACGGACGAGGACCTCGAGGCGCTCCTAGCGGACCACCACCTCTGTGTCATGAACCACAGCTACGAAGTGGACTGGCTGGCCTGCTGGATGGTGACCGACCAGTTTGGAGTCCTTGCGAATGCAAAGACATTTGCAAAGAAGTCCCTCCAGTGGGTGCCCATCATTGGCTGGAACTGGGCGCTCTCCGAGCACATCTTCCTCGAGAGG------AGCTGGGAAAAGGACTCCCAGACGATTGGGGGCAAGCTGGACCTTCTTCTTGACTACAAGGAC---AAGATTCTTCTGCTTCTGTTCTCTGAAGGAACACGCTTCAATGAGAAGAAGCACAAGCTGAGCCTTGAATTTGCCAACAAGCGGAACCTCCCAAAGCTCAAGCATCACCTCCTCCCCAGACCCAAGGGGTTTGTGTACTGCGCCCAGCACTTCAAGCAACGAGGCGTTCCGGCAATCTTCGACGTGCAACTCGGGTTCCGAGACTCCCCGAACCCGCCAAAGATAATGACCATTCTCAATGGGCATCCCTTCGTGGGAGACATCTACTTCCGGAGAGTGCCCCTGAATCAAGTTCCAACTGATACGGAAGAGGAGTGCACCAAGTTCCTCTACGACTTGTATGTGCACAAGGACAAAGTGATGGAGGACTACCTCGAGACCGGCCGATTTCCGGGCACCGTGAGGGAGCTCCCCGTTCGGGTGTGGCCGGCAGTGAACATGACGGCCTGGGCCATCCTCACTGGAGCGCCGTGTCTGTACGCCTTC---TACGCCATCCTGACCAGCGGCAGCACCTTTACG---------CCTTTCCTCGTTTTCCTTGCAGTGTTCTCTGTTCTTAATTGGCTCGTAGGGCTCTCTGAAATTGAGAAGAGTTCGTCGTACGGG [1047]

Siro TTCTTGCTAAGCAGTTTGATTCTGAATGGAATTCAGTTGCTCTGTTATCTTATTATCAAGCCATTAAGCAAAGATCTTTTTAGGAGGATCAATTATTACCTCATTTATTCATCATGGTCGCAGGTTGTTGCCATTGCAGAATGGTGGTCAAAATCTGAATTGATCATGTATGGCACAGATGAAACATTTAAAGACTTTGGCAAAGATCATGCCATGATTGTAATGAACCACAAATACGAAATCGATTGGCTGTACGCTTGGATGGTTTGCGACAAGCTTGGAAACTTGGGTGGTGCTAAGTCATTTGCAAAAAGCTCACTGAAGAAACTTCCAATTATCGGTTGGGCGTGGATATTTGCCGAAATGATCTTTTTAGAACGA------AACTGGGAAAAAGATTCACGCACGTTGCACTCTCACCTGGATGAAATAATGAGCTACAAAGACACCAGCTGCTTAATCCTCCTTTTTGCTGAAGGAACGAGATTTACTAACGAAAAACATCAAGCCAGTTTGGAATTTGCAAAATTGAAAAATCTTCCTCAGTTAAAATATCACCTTCTTCCTCGAACGAAAGGCTTTGTTTATTGCATGAAACATTTG---CAAGGCAAAATGGATGCGATCTATGACGTGGAGTTGGTGTTTAAAAAAGAACCAATC---CCGACAGTTAATAATTTGTTGATGGGCCAGAAAATGGTTGGCCATATGTACATCAATAGGATTCCACTTGACACTATTCCAACTGATTCTGATGAAGAATGTGCAAAGTGGTTGTATCAGCTTTATGAAAAGAAGGACAGATTAACAGAAGAGTTTATCCAGACAGGCAGGTTTTCAGAGAAAGTGCAAACCTTACCTTATCGTTACTGGGGTCCACTTAATTGGTTGTGTTGGGCTGTAGCAGTTGGGCTTCCATTTGTTACATTCTTA---TGGAAAATGATGGCTTCAGGAAATACTCTAATTATGGCAGCTGCAACAGTTGTTGTTGCTTCTGTTGTGATTGGTATCGTGTGTTTTCTAAATGTCACAAAAGCCAGCAAAGGATCGTCGTATGGC [1053]

Sitalc TTCCTCCTAAGCAGTCTGCTTATGAACCTCTTACAGCTATGCCTATTCTTAACTATTAAAAAAGTCAATCTGCACTGGTATAGGAAACTCAACTACTACCTCATCTATGCATCTTGGTCTCAGGTGGTGGCAATCGCAGAATGGTGGGGAGGTTCTTCGTGCTGTATCTACGCAGATGACGACACGTGGAACAATTTCTGTAAGGAACATTCCATAGTGATCACCAATCACAAGTATGAAATAGACTGGGCTGTCTGTTGGATGGTCTGCGACAAGTTTGGTGCCTTAGGGGGTTCTAAGTCATTTGCCAAATCGTCGTTGAAGAAACTTCCAGTGATTGGCTGGGGCTGGACGTTCTGTGAGATGATCTTTTTAGAGCGG------AACTGGGAGAAAGATTCTAAACTGCTGCCAAAAGCGTTAGACGTGATCATGGACTACAAAGATACCAACTGTCTTATTCTTTTTTTTGCGGAAGGCACTCGCTACACCAAAGAAAAGCATGAGACAAGCTTACAGTTCGCCAAGGAGAGGAATCTGCCTCAACTTAAATATCATTTATTGCCTAGAACGAAAGGCTTCAACCTTTGTGTCAAGCACTTA---AAAGGAAAAATGGATGCTGTTTACAACATCCAGTTAGCGTTTAGTAAAGACAGTGCCAAGCCAACAGCCAGCAGCCTTCTCTGGGGTCGTCAGGTGAAGGGTCACATGTACCTGAAGAGGATCCCACTAGCCGATATTCCTGTGGACGACGATCACCTTTGTTCTGAATGGCTCTATGATCTCTACAAAGAAAAGGATGAGCTTATGGAAAAATTTGAGAAGGAAGGTTGTTTCCCGGGTCCCAGACATGACCCCAAGGCCCGTTTGTTTCCCCTACTTAACCTAGCTGGCTGGGCTCTGGTCTTCGGGGTCCCCATCGTCTATTATTTA---CTTAAGGCTCTTCTTTTCGGATCCAGCTTCGTCATTGCTGTAGCCAGCACCATCATCGCCTGCCTTGCGGCTGGGATCTACCTAATGGTGCGAGCCACAAAAGCAACGAAGGGGTCATCATACGGA [1056]

Sclero TTTCTGCTGAGCAGTTTCATCCTAAACATACTACAATTATTGTTATTCTTCACTGTGAAGAGACTCAATATATCGCTATACAGGAGACTCAACTATTATCTCATTTACTCTTCTTGGTCACAGGTGGTTGCCTTGGCGGAATGGTGGTCGGGGTCCCATTGTAGTATCTATGCAGACGAAGAAACCTGGAAGCACTTTGGCCAAGAGCACTCCATTGCACTTATGAATCACAAGTTCGAGATTGATTGGGCTGTTTGCTGGATGGTAGTTGACAAACTAGGCTATTTAGGGGCTGCCAAAACCTTTGCCAAAAGTTCCCTGAAAAAGTTGCCAATAATTGGCTGGGGTTGGGTATTCTGCGAAATGATTTTCTTGGAACGTAATTGTAATTGGGATAAAGATTCTTCCACGATGAAGGAAGGTCTGGATGCAATTATGGAATATCAGGAT---AAGTGCCTTATTCTTCTTTTTGCGGAAGGCACTCGCTTCACAAAAGAGAAACATGCGACCAGCATCCAGTTTGCGAAAGAAAAGAACTTACCTGAACTGAAGTACCACTTGCTGCCAAGGACAAAAGGATTCAACTACTGCGTCAAGCACCTT---AATGGAAAGATGGATGCCATCTACGACATTCAAGTAGCCTTCAGTAGAGGCAGTGCCGAACCCACGGTAAGCAGCTTTATGTGGGGAAAGCCCGTTCAGGCCCACATGTACGTTCACAGAATACCCTTGGCTGATATACCCACACAAGACGATGCAAAGTGTTCTCAATGGCTTTATGACCTCTACATTCACAAGGATCAACTAATGGAAGAATTTGAAGCGGAAGGCCGGTTCCCCGGTATCCAAAAGGATCCTCCTCGAAGGCTACCTGGCGTTTTGAACCTTGGTGGTTGGGCTATTGCGGTTGGCAGCCCCATCATTTATTTTCTA---CTCAAAACCATGCTTTTTGGCTCTGGTCTTAAAATGGCCATTGCTACATCCATCCTTGCTCTTTTGGTCGCAGTAGTATATTTGATGTTTCGCGCAACCAAAGCAGGCAAAGGATCTTCCTACGGC [1059]

Trogulus TTCGTATTGGGAAGTCTCATCCTGAATTTCTTTCAATTATGTATTTATTGGTTTATTCGTCCCTACAACAAGCATCTCTACAGGACTATTAATTATTATCTTACATATTCTTCCTGGTCACTTACCCTCGGGATAGCAGAATGGTGGGGTGGATCGAGATGTCGATTGTATACTGATGAAGAAAGCAAGAAGCACTTTGGAAAGGAGCATACTCTTGTATTGATGAACCACAAATACGAAGTTGATTGGCTGATTACTTGGATGGTTGCTGAAAAATTTAATGTTCTTGGGGGTTCAAAATGCTTTGCCAAAAAGTCTTTGAGGAGTTTACCAATTATTGGATGGGCCTGGATATTCTGCGAAATGATATTCCTGGAAAGA------AATTGGGAGAAGGATTCCATGACAATAGGGAGTGGTCTGGACAACATTTTGGAGTACAAAGACACAAGCTGTGTGTTACTACTGTTTCCTGAGGGCACAAGATTTACCGAAGACAAATATAAATCAAGTTTGCTATTTGCTAAAGAACATGGTCTACCAGAACTCAAATATCACTTGCTGCCCAGAGCAAAAGGATTCAATTTTTGTGCAGCATATCTT---AAAGACAAGATCACAGCCATTTATGACGTCCAACTAGGCTTTCCCAAAGGCGTCAAC---CCTTCAGTCATGCAACTCATTCAACGCCAACCCATTTTTCCCGATCTCTACATTCGGCGAATTCCTATGGAGAAGGTGCCAACAGATTCTGATGAAGCTACTACTAAATTTCTTTACGAATTGTACCAGCACAAAGATAAGCTAATGGAAGATTACTGGGAAACTGGAAAATTTCCTGGCGATTTGACTGTACTGGAACCCCGCATTTCAAGCCTCGTCAATCTGTGCTTCTGGACACTGGTGATTGGGCTTCCAATCATTTATTTGATT---TTCAAGGTTATCTTTTTGGGCAGCACAACAGTCATCATAATAGCCACCTCCCTATTTGCCTTAGTCCTAACTGGCATTTACTACATGCTGGGTGTAACTCAGGTCAAGAAAGGATCTGCTTACGGC [1053]

Ortho TTCCTAATCGGTAGCCTCATCCTGAATTTCTTTCAACTATGTATATATTGGTTAATCCGGCCATTCAATAAGCATATCTACAGGACTATAAATTATTATTTGATCTATTCATCATGGTCATTAACATTGGGTATAACAGAATGGTGGGGAGGCTCAAGACTGAGGCTGTATGCCGACGAAGACTTCTCAGAAAACTTTGGCAAGGAGCATACACTAGTTTTAATGAACCACAAATATGAAGTTGATTGGATAGTAACCTGGATGATTGCTGATAAATTTAACGTTCTTGGAGGATCAAAATGTTTTGCAAAAAAATCTCTAAGAAGTTTACCAATCATTGGATGGGCCTGGATATTTTGTGAAATGATATTTTTGGAAAGG------AATTGGGAAAAAGATTCCAAATTGATAGGCACTGGTTTGGATAATATCTTGGAGTACAAAGATACAGCTTGTGTGTTACTGTTATTCCCAGAGGGTACCAGGTACACAGAAGATAAACATAAAGCCAGTATACAATTTGCAAAGGATCATAATCTTCCAGAGTTAAAATATCATCTCCTACCAAGAACTAAAGGATTCAATTTTTGCGTTGCTCACCTT---AAGGACAAAATTACGGCCATTTATGATGTCCAGTTAGGTTTCCCAAAGGATGAAAAT---CCATCTGTTCTGCAAATCCTCCAGCGTCGAAGAATTCTCCCTGACCTTTACATGCATCGTATTCCCATGGAAAAGGTGCCTACAGAATCAGAGGCAGCTTCGTCAGAGTTCCTCTATGATCTCTACAGAAAAAAAGATAAACTAATGGAAGAATATTGGAAGACTGGCAAATTCCCGGGGAAGTGCACTGAGTTAAAGCCCAGAATCTGGAGCCTTCTAAACCTCATCTTTTGGACTTTGGTCGTTGGTCTACCCATAGTTTACTATCTC---CTCAAGATAATTTTGCTCGGAAACACTACTGTAATACTAATTACCTCTTCTGTAGTGGTGCTAGTTCTAACCGGAATCTACTACATGCTGGGTGTAACACAAGTCAAGAAAGGGTCAGACTATGGC [1053]

Hespero TTTTTGCTAAGCAGTTTAGTGCTAAACTTTATTCAATTGGTCAATTATCTATTGATTTGGCCGCACAATAAACGATTGTACAGACGCATCAACTATTTTCTTGCTTATTCGTCGTGGTCTCAGGTTGTAGCCATTGCAGAATGGTGTGCCGGATCCAAAGTACAGGTTTTTGCTAATGAGAAAACGTGGGAAACATTCGGCAAACAACACAGTTTAGTTCTGATGAATCATAAATACGAAGTAGATTGGATCGTTACTTGGATGACCTCGGATAAATTCAAAATATTGGGTGGTTGTAAATGCTTTGCCAAAAGCAGTTTACGAAGTCTTCCCATTATTGGTTGGACGTGGATCTTCTGCGAAATGATTTTCTTGGACCGA------AATTGGACCAAAGACAAAGAAACATTGGGCACTAAATTGGACGCTATTCTGGAGAATAAAGACACGGAGAGTTTAATGCTCCTGTTCCCAGAAGGAACCCGATTTACATTAGAGAAATACGAAGCCAGTATGGAATTTGCCAAATCGAAAAATTTACCCCAAATGAAATTTCATTTGCTTCCGCGAACGAAAGGATTTAATTTTTGCGCTGCTCATTTA---AAAGACAAAACTACGTGCATGTTTAACATCCAATTGGGATTCCCCAAGGGCACAAAT---CCTAGTGTACTCTCTATATTGAAAGGAGAGCCAATGTTTCCTGATATTTACATTGACTACATTCCGATTAAGGACATTCCGACCGATTCAGACGAGGCTATGAATAAATTCATGTATGATTTATATCACAAGAAGGACGATTTGATGGAAAATTACTGGAAAACTGGTAAATTTCCTGGTAAAGCGATATCCCCTCGTAGACGAATAACTCCTCTATTGAATTTGGTCATTTGGTCGGCCATAACTGGACTGCCCGTCATTTATTTCGTG---CTTAAAGTCATTTTGAGTGGAAGCGTAACTACTATTATGATAACAGCTACAATTTTTGCTTTGGCCATTGTGGCTGTTTATTTCTTATTGGGAGCAACTCAGGCCACAAAAGGATCGTCTTATGGT [1053]

Leiobunum TTCTTAATTAGTAGTCTTATTTTAAACTTTCTTCAACTATGCATTTATTTTATAATTCGTCCCATCAATATTCATTTATTCAGAAGAATTAACTATTATTTAATATACTCATCTTGGTCTCAGGTTGTTGGCATAGCGGAATGGTTTGCAAATTGTTACGTAAAGGTCTATGCTAATGATGAAGTTTGGAGAGATTTTGGAAAAGAGCATTCTATAGTGCTTATGAACCATAAATATGAAGTTGATTGGCTTTATGCTTGGATAGTAGCTGACAAATTCAAAATTTTAGCAGGTTCAAAATCCTTTGCCAAAAGCTCACTCAAAAAGTTGCCAATAATTGGATGGGCCTGGATATTTGCTGAGATGATATTTTTGGAAAGA------AATTGGGAAAAAGATTCAGTAACTTTAACAGAAAAATTAGACGCTATTATGGACTACTTTGACACAAAATGTTTGATTCTTTTATTTGCTGAAGGCACTAGATATACCAAAGAAAAATATGAAGCTAGTTTACTTTTTGCGAAAGAAAAAGGGCTACCTCAGCTGAAGCACCATTTACTTCCCAGAACCAAGGGATTTAATTTCTGCGTTAAACACCTT---AAAGGAAAAATGCCAGCTATCTATGACATTCAATTGGCCTTCCCCAAGGATCAAAAA---GTTTCCGTTCAAAAGATTTTGTGGGGAAATCAACTAGTTGGGGACATGTATATTAGGAAATTTAGATTCGAGGACATTCCAGTTGATAATGATCAAGAATGCACCGAGTTCTTATACAATGTGTATCGACAGAAGGATGAACTCATGGAACAATATTGGCAAACTGGAAAATTTCCAGGGGAACACAGAGTTCTCAAGCGTCGTATTTGGGGAGCTTTAAATTGGCTTCTATGGGCTTTCCTAATTGGCACACCCATTTTATATGTCTTAATATTTAAAGTAATTTTGTCTGGAAGCGCTACTACAATTGTAATAGCTACAACTTGTATAGTTTTAGTATCAACCGGCATATACATAATGTTGGGAGTTACACAAGCTAAAAAAGGTTCCTCTTATGGC [1056]

Proto TTCTTAATTAGTAGCCTTATATTAAACTTTTTTCAACTATGCATTTATTTAATAATTCGACCCATCAATGTACATTTATTTAGAAGAATTAACTATTATTTAATATATTCCTCATGGTCTCAGGTTGTTGGCATTGCGGAATGGTTTGCAAATTGTTACACAAGGGTCTATGGTAATGATGAGGTCTGGAAGGACTTTGGGAAAGAACACTCAATAGTTATCATGAACCATAAGTACGAGGTTGACTGGCTCTATGCGTGGATAGTAGCTGACAAATTTAAAATTTTAGCTGGATCAAAATCGTTTGCTAAAAGTTCGCTAAAAAAGTTGCCTATAATTGGGTGGGCCTGGACTTTTGCTGAAATGATATTCTTGGAAAGA------AATTGGGAAAAAGATTCGGCAACTTTAACAGAAAAATTAGATGCTATAATGGACTATGTTGACACAAAATGCCTAATCCTTCTGTTTGCTGAAGGCACTAGGTACACCAAAGAAAAATATGATGCTAGTTTACTTTTTGCCAAAGAAAAGGGTCTACCTCAGCTAAAGCATCATTTGCTTCCTAGAACTAAAGGGTTCAATTTCTGCGTTAAGCATCTA---AAAGGCAGAATGCCAGCTGTTTACGACATTCAATTGGCCTTCCCTAAGGATCAGAAA---GTATCTGTTCAAAATATTTTGTGGGGGAAACAACTCATTGGGGATATGAACATCCGGAAAATTAAGTTTGAGGACATTCCAGTTGACAATGATCAAGAATGCACAGAGTTCCTATACAACGTATATCGTCAGAAAGATGAACTGATGGAAGAATATTGGAAAACTGGGAAATTTCCTGGAGAATACAAAATTCTTCAGCGCCGCATTTGGGGACCTTTAAATTGGCTTCTGTGGGCTTTCATAATTGGCACTCCTACTTTCTACTTTTTAATATTTAAAGTTCTTTTGTCTGGAAGCCCGACTATGATTGTAATAGCTACAACTTGTATCGTTTTAATATCTGCTGGCATCTACATAATGCTGGGAGTAACCAAAGCTCAGAAAGGTTCTTCCTACGGC [1056]

[TICKFAST_NCHAR=990;26S proteasome regulatory complex, subunit PSMD5]

Ixodes ATGCTCTTGCAGGTGTTCGTGGAGATAGCCAAGGCATCTCCGGAGATGCTGGAGAAGCTGGCCGAGAGTGGTCACCTTGACAAGCTGCTCCAAGAGACGAACCGGGGGGACGTCCTGGTGCGGCTCAATGCCCTAGAGCTCTTGACGGAGCTGGCCTCCAAGAGCCATGGCCTGGACTACCTTGAGAAGAAGGGAATCTTGGCGAAATTGAGCAGCGAGACAATCAACGTGGCCAAGGACCCACTGGGAAGCCTCCTTGGACCTGGCTTGCTGAAGTTCTTCGGCAGCATGGGACAGACCAGTCCCCAGCGGCTGTTGGTAGACTACCCGCAAGTGCTCGGCTTCATCTTTGAGTGCGTC------CAGGACGGCGACTCAGCCAGCCAGGCCGTGGCGGTCGAGACCATCGGTGTCATTGGTTCCAGTGTCCAGGGGAAGCGGGCACTGCGGTCGCACGGTGATAAG------ATGAAGCAGTATCTGAAAACCCTGAGTCAAAACATTCAAGGAGGAAGGAGT---GAGCTTCGTGTGCGGTCCCTGGATGCCCTGGCCAACATACTCCACATAGAGGGC---------GATTCCTGCAAGGACATCATTCGCCTGACGGAGCAGTGGTACGACGCCTTCGGCGAA---GCCCCCACCAGGCTCCTGCTGGGCGTCTGCAAGCAGCCCTTCCCGGACCTCCGTTGCTCTGCCCTGGGAGTGGTCTGCGAGGTGGCCCACAGCCCGTGGGGCCAGGCCGCTTTGGCCAGCCACCCGGGCTTCCTGGAGTACCTGCTCGATCGCTCCACAGAGTCCGACAAGGAAGGCAAGGAGGCCAAGTTTGCCATCGTGGCCGCGCTCGCGCAGTGTCCGTCCCAACCTCGGGGTTTCTCGTCGGAAGACTGGCAGAGGATCAGGACCGCCCACAAGGAAGGGCCGTTTTACGTGGCGGCTGAGGCA---GCCGTCGCTTTCGAGGGCTCCTCGTGA [960]

Siro TTTCGCTTGTACCAGTTGATGGTGTCCATTGCAAGTTCATCTCCCACATCTCAGCAAACTGTAGCTGATTACCTCGAAAGACTAATTTTAGAATCCAAAAAAAGGGAGGACATTCTGGTTCAGTTGAATAGCATCGAACTCCTAGGAGAACTGGCAACTGTTCCGCATGGTCTTGAGTTACTCTCTCAAAGAGGTGTCATGCAGCATTTTGAACAAATTCTGCGAGATTCTACTTCAAATCCATTCTCATCATTTCTGTTGCCTGGTTTGTTGCGGTTTTTCGGCAACATTGGTATTGAGAATCCAAAGCCAGTTGCTTCAGTTCTAACAGTTGTCTTCAAAATTGTTCTCACATCAGAC------------------AGCATACTTCAGGGTATTGCCATTGAAACTATTGGAGCTGTTGGAACGTCATACATTGGCAAAACTCTTCTTAATCAGCAGTCAGAA---------ATGGGGGACTGTTTGCAAGTCATTGGACGAATTATTGCTTCAGGAGAATCG---GAGCAGAGAGTACGAGCCATTGATGCCCTTGTTCGCCTTCTTACAATTAAGGAC------AATGTGAATTCCAATGAAGTTGAAAGCATAATAGAAAAGTGGTACCACGTGTTGGGGAGA---GATGCAACCAGTGTGTTTGTGGCCTTATGCAAGCAGCCTTTTCAAGAGATGAAATTAGGGGCCCTGGATGTATTGGGCCAATTGTCCTCCAAAGAATGGGGTCAGAAGGAGCTGGTCTCACAGCCAGGATTCCTTGAATATCTTTTGGACCGATCAACTGAGCACGATAAAGAAGGGAAAGAGAAGAAATACATAATTGTCCGAGAAATTACCCAATCAGTCACAGCAGCCTCAATCTTTTCTGCAGACATTCTTGTCAAGCTGAAAAACTACACAAAAGAAGGACCTTTCTTCGTTCATGCAGAAGCA---GCAGTTGGATTTGAGAGTGCTGAATGA [948]

Sitalc TTCAGACTCTACGATGTGATGGTCAGTATCGCCAGACAACGTGAAGCAGTTTTACCCTTAATCGCGGAGGAGTTCTCGGAACCTCTCCTCGCGGAACTGGACTCTGGCGATGTCCTGTTACAACTCAACTGTCTCCAACTACTCTCCGATCTTGCCTGCGTTCCGCACGGTCTGGCATTTCTGCAAGAAAGAGGCCTGTTGCAACGACTGGACTCTCTCCTATTGTCCTGCAATGAAGATCCACTGGCATCTTTTCTTATACCAGGAATTGTCAAGTTTTTCGGCAGCGTTGGTCGCGTGAGGCCCGAAGAAATCTTCGAGAACCATAAATCGGTGGTCCAGTTTCTGTTGTCGTCTATC------GAAGTAGCGGACCCCTCTGTACTGACTGTGACAGTCGAGACTTTAGGCATCATAGCTACCTCAACTGAAGGAAAAAGGCAACTACAAAGGACAGGTGATCAG------CTGAAAGGTTGTTTGCAGAGCATGGGACGTCTTCTTCACGGCGGAAACCCCGAAGAACTAAAAGTTCGAATTCTGGAGATGTTGAAGAACATGCTGCAAATATCGTCACTGGATGAACAGGAAAAAGAAGAGATGAGTAGCCTGACAGAGAATTGGTTTTGTGAACTCCATCCT---GAACCGTGCCAAGTAATCATGGATCTGGCCAAACAGCCATTTTCCCAGTTGCGCTGCGCTGCCCTGGAGCTGATTACCACCATTGCACCTTGCTACTGGGCACAACTCATGATGCACAGACAGCCTGGTTTCATCGAGTATCTACTGGATAGAACAACAGAAAGAGAAAAGGAAGGAAAATATGCAAAATACGGGATAGTTCAAGCTTTGGCCGACTCCTCTACTTGTGGTCAGGTGTTCGGGGAGGAAGCGATTATTCAGCTGCGTCAGTTTGTGCGCGAAGGACCATTCTACGTTAGAGCAGAAGCTGCCACCGTTGCTTTAGAAGGGCCCTCTTGA [975]

Sclero TTTAGGCTATATGATGTAATGGTTGGAATCGCAACGCAACGAGCTGCTGCACTTCCTTTGATAGCCGAGGAATACTCTGAGCCCCTGCTATCGGAACTCGACTCGGGGGACATCCTATTGCAATTGAACTGCCTCCAACATTTAGGGGAACTCGCGACCTCCCAACACGGACTCTTGTTTCTTCAGAAGAAAGGACTCCTAGATCGTCTAGACTTCCTGCTGGCTACCAGCGCCGAGGACACTCTGGCTTCTTTCCTCGTACCAGGTATCTTAAAATTCTTCGGCAGCGTGGGCCATCTGCGACCAGAAGAAGTATTTGAGAATCACACGTCAGTGGTGCACTTTATTATGTCTGCAATT------GAAGGAGGAGACCCTTCCTTATTGACTGTGGCAGTGGAGACTTTGGGAATCTTGGGCCATTCTCACCAAGGGAAGCGGTTGCTGCACAGGACGGGAGGCAGT------TTGGAGAGCTGCTTAAGAAGTGTTGGCAGTTTGTTACAAGGGGGAAGCACCGAGGAACTCAAAATAAGGGTCCTAGGAATGCTGACACAAATCTTCCAACTGTTACCT---------TCTGAGTCTGAAGGACTCGCGTCTGTGACGGAAGCCTGGTTCCATTTTCTGGGGCCT---GATCCTCTCCAACTGATGCTCAGGTTGGCCAAACAGCCTTTCCCCGAGTTACACTGTGCCGCCTTGGAATTCCTCAGGAGCCTGGTCTCCTGGCATTGGGGGCAGCTCCTTTGCCAACAGCAAGCGGGCTTTCTCGAGTATCTACTAGACAGGTCCACAGAGCACGAAAAGGAAGGGAAGGAAATCAAATACAAGATTGTTCAGTCATTGGTGGAGTCTGGCACGAGTAGCCAGGTATTCGGAGAGGAGGCCCTGACTCAGCTCAGGCTATTTCTGAGGGAGGGACCCTTCTATATCAGAGCGGAGACT---GTTGTGGCTCTAGAATCTGCATCCTGA [963]

Trogulus TTGCGAGTCTACGATCTTATGATCTCCATTGTTTGTCACTTTCCCTCATCTCTCCCATTGGTTGCTGCTGAATATTCTGATCAAATACTACAAGAGTTGGAATCAAATGATGTCTTACTTCAACTCAACTGTGTAGAATTACTTACAAAATTGGCACTGACCAGCCATGGCATGAATTTCTTAGAACAGCAAGGTGCAATAGAAAAGTTTAGAAGCATTTTGTGTGAACCCAGTGATTATGGATCCCATTCTTTTTTGTTTCCTGGTTTAATACGGTTTTTTGGTAACCTTGGAGTTCGGCATCCTGAAAAATTATTTGGCGATTATCCTGATGTCCTAACTAACATACTGACAACCATT------------TACGATCCTGAGCTATTTATGGTTGTAACTGATACATTGGGTGTTATTGGCAGTACTGGTATTGGAAAACGTAAATTAGATTCACTTGCAGAATGTGATCTCCTCGATAGAGCTTTACAACGCATTGGCAGTTGCATAGAAATTGGTCCATCT---GAATCAAGAATGAGAGCATTAGAGGCCTTGTCCAATTTGCTTCGTATTGAGAAA------ACTGACAGCTGTACAGAATTGGATAGAATTGTTGAAAACTTCTTTGGGAAGTTGACCACTGGAGATCCCATGCTGTTATTGCTAACTGTTTCGAAATTGCCTTTTGTGGAATTAAGATGTGGAGCTTTGGAGGTGATAAAACAAATTGCTTCTCGTGAATGGGGCCAAAAGTTGCTCCAAGCACAACCAGGCTGTGTTGAGTACTTGCTGGATCGCACAACAGATTCTGATAAGGAGAGCAAGGAAGCAAAGTACAGTGTGGTTCAGGAAATAATCGAATCTTCATCAGCAAGGTCCACATTCGACTCTGATGTCTATGCTAGGCTGATCGAATTCTATAAGGAAGGCCCATTTTATGTTGCCACGCAACTG---GAAGTTTCATTTGAACCCGCCTCTTAA [966]

Ortho TTTCGAGTTTATGATCTGATGATTTCTATAGTGTGCAACTTTCCATCTGCCCTTCCCCTGGTGGCAGCCGAATATTCTGATCAGCTCTTAGAGGAACTGGAATCAAATGATGTCCTCTTACAGTTGAACTGCGTGGAACGCCTCACCACTCTGGCCCTCACTAACCACGGTCTCAGTTTTCTTGAACAGCAAGGTGCAATTGAAAAGTTCAGCGATATACTTCTGGAGTATAGTCCTAACAACTATGCAAAGTTTCTTTTTCACGGTTTAATAAAATTTTTCGGTAACCTGGGTATCCAACACCCGGATAAATTTTTCGCGGACCACACGGAAGTACTTCACTTCATCCTGAGGGTCATAGACATAGAGAGTCAAGATCCCTCGCTCATCACATTGTCACTGGAGACATTGGCAGCCGTCGGCAGTTCAAGTCTTGGAAAACGCAAGTTGGATACCCACGCAGAACCTGAAATTCTGGCTAATGCATTTTATATTATGGGCTGTTATATGGAAAGAGGTGCCCCT---GAGCTTAGATTGAGCTCGCTTCAGGCTATCTCGACTTTACTGCAAGTCAAGAAA------TCCGAGGTGTGCCTGGAACTGGACAACATCGTTGAAAAATTCTTCAGACTGTTGAACTCT---GACCCGGTCGGAATATTACTTAGCGTCTCTAAACTCCCGTTCACGGATTTACGTTGCGCGGCTCTGGACGTGATAAAACAGATAGCATCTCGTCCGTGGGGTCAAAGGTCGCTCCAGACACATCCGGGCTGTATTGAGTACCTCCTGGACCGGACCACGGACGCCGATAAGGAGAGCAAGGAAGCAAAGTTCGCGGTGGTACAAGAAATCGTTGAGTCGTCCTCAGCCGGTTCCGTTTTTGATGCTGATGTTCTGGACCGGCTTACCCAGTTTCATAAGGAGGGACCATTTTATGTTGCTACCCAAATG---GAAGTGTCCTATGAACAAGGGTCTTAG [975]

Hespero TTAAGAATATACGATGTGATGATCTCTATAGTTTGCGACCACCCTTCTAGCTTGCCTTTAGTGGCTTCCGAATATTCGGATCAACTATTAAACGAACTCGAATCCAACGACGTTTTGCTCCAATTAAATTGTGTGGAACTTTTGACCAAGTTGGCCCTTACTAACCACGGGCTGACTTTCTTGGAACAACAAGGAGCCATCGAGAAATTCAGGAGCATTTTATTAGATTCGGCCGATGAATCGGCAACTACTTTCTTATTTCCTGGCCTAATTAAATTCTTCGGCAGTCTCGCCCTCAGACATCCTCAGAAATTCTTCGAAGAATATTCGGATGTACTTCATTATATTCTCACCATTATA------GAATGTCACGACGCTACGCTATTAGGCGTTTCCATAGAAACATTCGGAGTTATTGGAAGCACCGCAATTGGGAAACGGAACATTGATATTCATGCACAGTCTGGTTTACTAGATATTGCATTTCAAACAATCGGTAAATTTATCGAAGCAGGACCCTCT---GAAATAAGATTACGAGCTTTCGAAGCATTTATTAATCTATTAAAATATCAGAAA---AATGAAACCAAATCGAATGATTTGGACTGTATAGTGGAAAAATGGTTGAATCGAGTGAGCAGC---GATCCTATCCGTCTGTTGACCACAATCTGCAAACAACCTTTTGTCGAATTGCGTTGCAAAGCTATGGAAGCTTTAAAGCATATTGCTGGCAATGAATGGGGTCAAAGGTTACTAAATGCCCAACCAGGTTTCCTAGAGTATTTATTGGACCGGAACACTGAGGCTGACAAAAATGGCAAAGAATCCAAATTCGCTATTGTGCAATCTATAATCGAAGCTTCGACTGCTAGTTCGGTGTTCGAAGCTGACGGTTTAGCCAAATTGGCTGAATTTTACAAAGAAGGACCGTTTTACGTCACTGCCCAAACT---GAAGTCTCATTCGAACAAGCTTCTTAA [972]

Leiobunum ATGAGAGTTTACGATGTTTTAATTACAATTTCATGTGACCAACCAACGGCACTGCCCTTAATAGCTTCAGAATATTCCATGCAAATACTTTCCGAGTTAGAATCTGATGACATATTGTTGAAACTTAATTGCATTGAGATATTAACTAAACTTGCTCTGACAAATCATGGCCTGGTATTTCTCGAACATCAAGGAGTAATTGAAAAATTCCGTAATATTTTGTTGGATTCATCAGATGATTCTATGGCTACATTTCTTTTCCCTGGTTTATTAAAATTTTTTGGTAATTTGGGCCAACAAAACCCTCATTTATTAGCGAAAGAATACTCCGATGTTCTGCATTACACATTATCAGCTGTT------GAATCGGGAGATCCAACTCTTAGAGCGGTTGCTATTGAAACTGTCGGTATAATAGGGACTAAAAATGAGGGTAAAATGATGTTGGATAAAATTCCGGGCTTA---AATGTTAGTGACACAATGCACGCAATTGGCCGCCAGGCTGAAACTGGAAGTGAC---GATATTAAACCTAGAGCTTTAGAAGCAATAGCAAACTTGCTTTTTATTGACAAA------GAGTCAGAAAAGACTGAAGTTGATGATATTGTTGAGAAATGGTTCCGAAAATTCTCGTACGGAGGATCATTGCGAATGTTATTGTCCCTTGCGAGAATACCATTTATAGAGGTTCGCTGCAATGCACTATTAGCTCTTAAACACGTAGCAACCAGAGAATGGGGACAAAGGCTTATAGCGTCTAGTCCTGGGTGTGTTGAATATCTCTTAGACCGTTCAACAGAAAGTGAAAAGCAGGGGAAAGAGTCCAAATATGCAATTGCGGTAGCTATAGTTCAAAGCAGCACCGCGAGTAGCGTTTTTGATGGTGACACCATGCAGAAAATATCAGATTTCTGTAAAGATGGTCCATTTTACGTCGGTTCCTCAGCA---GAGATAGCTTTTGAACAAGCGTCTTAA [969]

Proto ATGCGGGTTTACGATGTTTTAATTACAATCTCATGCGACCAACCCACTGCGCTACCGTTAATAGCTTCGGAATATTCCATGCAAATATTATCAGAGCTAGAATCCGACGACATTTTGTTGCAACTTAATTGTATCGAGATTCTAACTAAACTCGCACTCACAAATCACGGACTTGTGTTTTTGGAACATCAAGGAGTGATCGAAAAATTTCGTAACATTCTGCTAGATTCATCGGACGATTCTATGGCGACCTTTCTTTTTCCAGGTTTATTAAGATTTTTTGGCAATTTGGGCCAAGGCAACCCAAATTTATTAGGGAAAGAATACTCCGATGTTCTGCATTACACTTTATCAGCTGTC------GAAACAGGCGATCCAACTCTTAGAGGAGTCGCGATCGAAACTGTTGGTATTATTGGCACCAAACACGCGGGCAAAATGATGTTGGATAAAATTCCGGGATTA---TGCGTAAGTGACGCTTTGCACGCAATTGGTCGCCAAGCTGAAAATGGAAGCGAA---GATATTAAACCTAGAGCTTTAGGCGCAATTGTGAACTTGCTTTTTATAGAAAAT------GAATCATCCACGCCCGAAATCGACGATATTGTGGAGAAATGGTTCCGAAAATTCTCATTCGGTGGAACCTTGCGAATGTTATTATCGCTAGCGAGAATACCATTTATAGAAGTCCGTTGTTTAGCGTTATTGGCGATTAAGCACGTTGCCAGCAGAGAATGGGGACAAAGGGTTATGTCTACGAGTCCCGGGTTTGTTGAATATCTTCTGGACCGTTCAACGGAGAGTGAAAAGCAGGGTAAAGATGCTAAATACGCAATTGCTATTGCCCTAGTCCAAAGCAGTACCGCAAGCCAGATTTTCGACGCAGAGACCATGCAAAAAATAACAGAATTCTGCAAAGAAGGGCCATTTTACGTTGGATCCTCGGCA---GAGATATCTTTCGAGCAAGCTTCTTAA [969]

[TICKFAST_NCHAR=516;28S ribosomal protein S24]

Ixodes ATGCCTCCCTCCACCCAGGTCCTG------------------------------------------CGGTGGGAGGTGCGGGGCATCCACACGTCGGGCGTGAGCGCGGCCAACCGCGCGGGCCGGTACCGGGTCACCAGGAACCACAGCAAGCCCCTGACGTACGAGATGGCCAACCCGCCCCACCAGATCGCCCACAGGAAGGCCTGGAACTCCTGGAACACAAGCAACCTGCAGGGCGGCCTGAGGCAGTCGGAGACCCTCATCGAGGACATGTTCATCAGGAAGTTCATGACCGGCACGTGGCACCGGATGTTTGTGTCGGAGATCCTGATCAAGCGGCGGCACAACATGGTGATGGTGGGCGGCATCGTGCAGCGCTCGGTCATCCCCCGCAAGATGTACTTCCTCCTGGGCTACACCGAGGAGATCCTCAGCTACGTGCTCAAGTGCCCCGTCAAGCTCGAGCTCCAGTCCACGGCGGACAAGAAGGACATGGTCTTCAAGTACATCTAG [474]

Siro ATGCTAACGGGATTAACGAAGAAT---------ACTGCATTCATT---------------------ACAAGTTTCTATAGAAACTTCCATGCGACAAGTGCTTGCACGAGAGTTCAAGCGGGAAGATACAAGGTGACTTTAAAGCATGACAAACCATTAACGTACGAAATGGCAAATCCACCCCACCAAATAGGGCACAGGAAATCTTGGAATTCTTGGAACACCTGCAATATGTTTGAAGGACTTCGAGCTTCAGAAACGGCAGTGGAGGATGTATTCATTCGAAAGTTCATGGCAGGGACTTGGCACAGACTATTTCTGTCAGAAGTTATCATTAAGAGACGGCATAACATCATTTACATAGGGGGCATTATACATCAGACCATTCTCCCGAGAAAAATGTACTTTTTGATTGGCTATACAGAGGAGATTTTGAGCTTCATTTTAAAATGCCCTATCAAATTGGAGTTGCAGTCCACGCCAGAAAAAACCGATGTCATATTCAAGTATATCTAA [486]

Sitalc ATGGTGGCTGCGGTAACGTCAGCG------GGTACTAAGCTATGCTCCTCGTTAAAATTG---TCACTGGTGTTAAAGCAAACACTCCACACAACAAGAGTATGTGAGAAAACCCAGGCTGGCCGTTATTTTCCAACCCCAAAGCAGAACTTTCCTCTCACGTACGAGATGGCTAACAAACCTGATAGGATCGGAGTATGCAAAGCCTGGACATCATGGAATACTAGTAACCTACTGGATGGCCTGCGCCGTTCGGAAACAGCAGTAGAAGATATTTTGATTCGCAAGATAGTGTCCGGAACTTGGCATGGACTTTTCGCATCGGAAATAATCATTAAACGACGTGCTAACATCATCTATATCGGTGGTTTTATCGTCAGGAAAACCAATGCTCGCAAAATGTATTTTCTGATCGGATACACTGAAGAACTTCTTAGTTGCCTTTTTAAGTGTCCCGTCAAATTAGAACTGAGCACCATCGACAATCACAGAGAGATGATAGTTAAATATATATAA [507]

Sclero ATGGCATCTTTTCAGGCTGTCGGGTTGTCTTCAGTTCTTCTAAAGTCTCCCTGCTACCTGCTGAACGAAGCCTTCCGAAGATCGTTTCATATGACAGCGTCTTGCGATAAGATTCAAGCCGGGCGTTACAAACCAACCAAGAAGCACACCAAACTGATGACCTACGAAATGTCCTGTCCTCCCAATTATATTGGACAACGCAAATCTTGGAAATCTTGGAACACGAGCAATTTATTGGATGGGCTGCGAAGACCCGAGTGCGCAATGGAAGACATTTTTATACGGAAATTCGTTACGGGAACATTTCACAATATATTTGTTACAGACATCCTGATTAAACGTCGGCACAATATTGTTTACATTGGTGGCATCGCAGGGTCGAGTATTCTCCCTCGCCGCTTATACTTTTTGATAGGCTACACAGAAGAACTCTTGGCCTACGTGCTGAAATGCCCCGTGAAACTGGAAATCTGTACGGTCGACGAGAAAAATGATGTGGCTTTCACGTTTATTTAA [516]

Trogulus ATGCTAAATAAACTCGCAACAGCA------ATTAACCTTTTAAATGGCATCCGATCTTCAGTTAATGTCTCCCAAGTTCGGTTGATTCAAAGTACAGCACCATGTCAAAAAGTGCAAGCAGGAAGATTCAAAGTTACTCGATTTCATAATCGCCCTTTAAATTATGAACAAGCTAATCCACCACATTACATTGGTCACAGAAAGACTTGGAATTCCTGGAACACAAGTAACTTAAAGGATCATGAACGGCGATCAGAAACGGCTGTTGAAGATTTATTCATCCGTAAGTTCATGTTTGGTACATGGCACAGACTTTTTGTATCTGAAGTTATCATCAAGAGACGCCACAATGTTATTTATATAGGTGGTATTGTTAATCAAAATATCCAAGCAAGAAAAATGTATTTCTTAATTGGTTACACAGAAGAACTTTTGTCTCTGTTTTTAAAATGTCCAATCAAAATGGAACTACAGTCAGTTAATAGTAGGGAT???????????????????????? [510]

Ortho ATGCAAATTAGCCAAGGAACAGCG------------ATATTAAGATGTATTCGATCCCAG---ACACTACAGCAGATACGATTAATCCAGACTACTGCAATGTGTCATAAAGTACAAGCGGCCCGTTTTAAAGTCACCCGGTTCCACAACAAACCCTTAAACTACGAAGAGGCCAATCCACCGCATTATATAGGGCACCGAAAATCCTGGAACTCATGGAACACCAGCAGCCTAAAAGACGAAGAGAGGAGTTCTGAAACAGCCGTGGAAGACTTTTTCATCCGAAATTTCATCTTTGGAACCTGGCACCGCCTCTTTGTCTCCGAGATTATAATCAAACGACGTCACAACATAATCTACATCGGAGGAATTGTAAACCATAAAGTCCAGGCTAGGAAAATGTATTTCTTAAAGGGATACACGGAAGAACTTTTATCATTATTTTTAAAATGTTTGGTTAAAATGGAACTGCAGTCTGTGGCGAGCAAAGACGACGTTATTTACAAATATATTTAA [501]

Hespero ATGTCTGTTGCTACTAGATTGCTG------TCCAGAGTTTATTGTCCCTGGAGAGGA---------TCTTCCAACATTAGATCGTTTCATGTCACGCCATCTTGCAGCAAAGTAACTTCAGGTCGATATAAAGTGACACCAAAGCAAGACAAACCTTTGACCTACGAGCAAGCAAACCCTCCATACACCATTGGTGTTAGAAAATCGTGGAACTCTTGGAACACCAGCAGTTTGCACGAGGCGGAGCGTCAGTCTGAAACATCCGTTGAAGATTTGTGGATTCGTAAGTTTATGTTTGGAACTTGGCATCGCTTATTTCTCTCCGAAGTGATTATTAAAAGACGTCACAATATAATTTACATTGGGGGAATTGTCAATCAGGCTGTGCATGCTCGTAAAATGTATTTCCTCATCGGATATACAGAGGAACTTTTGAGCCTATTTTTAAAATGTCCCGTTAAACTAGAGTTGCAATCGACACCCGATAAAAAAGACGTCATTTTCAAATATATTTAA [501]

Leiobunum ATGGCTTTAAACCAAATTAAAGGT------TACCAAATCTTTAAAACCTCTAATTTAGTA---TTCAGTTTTATATCCCGAGGGTTTCACCTATCTGAAACATGCTGTAAAGTCACTTCTGCCAGATATAAAGTTACCTTTAAACAAAACAAACCCCTAACATATGAGCAAGCAAATCCGCCACATTTTATAGGAGTTAGGAAATCCTGGAATTCATGGAATACAAGTAACTTATTTGAATCAACAAGGCAGAGTGAAACTGCGGTAGAAGACTTTTTCATTAGAAAGTTCATTTTTGGCACATGGCATCGCTTGTTTTTATCAGAGGTTATCATTAAACGTAGACATAATGTAATTTACATTGGTGGAATTGTATGTCAAAGTATCCTGCCCAGAAAAATGTATTTCTTAATTGGTTATTCAGAAGAACTGTTAAGTTACTTCTTAAAATGCCCTGTAAAATTTGAGCTTCAGTCTACTTCTGATAAAAAGGACGTAATATTCAAATACATCTAA [507]

Proto ATGGCTTTAAGCCTATTTAAAAGA------TGTATTAACGTTAATTCCTCAAATTTGGTG---TGGAGTTATGCTTCCCGAGGATTTCACGTGTCTGAAACATGCTTGAAAGTCACTTCTGGAAGGTACAAAGTTACCTTCAAACAAAACAAACCCCTTACATATGAGCAAGCAAATCCTCCTCATTTTATAGGTGTTAGGAAATCGTGGAATTCGTGGAACACAAGTAACTTATTTGAATCAACCAGGCAAAGCGAAACCACGGTGGAAGACTTTTTCATAAGGAAATTTATTTTTGGAACCTGGCATCGCTTATTTCTATCAGAAGTTATAATTAAACGTAGGCATAATGTAATTTACATTGGTGGAATTGTAACTCAAAGTATATTGCCCAGAAAAATGTATTTCCTRATTGGCTATTCRGAAGAACTGTTAAGCTACTTCTTAAAATGCCCAGTAAAATTTGAACTTCAATCTACTGGTGATAAAAAAGATGTAATATTTAAATATATATAA [507]

[TICKFAST_NCHAR=1059;28S ribosomal protein S9]

Ixodes TCTGAAACCAAGGTTCAAAAAATAAGCAAGGCCATGAAGTCGTACTTGGACAGAGCAAAGGCATACGATGAATTCATTCAGAAGGAAAAGAACGAATTTGAAATTGGAAAGCGCCACCTTGCCAACATGATGGGCGTGGATGCTGATGAAATGACTCAAGCTGATGTTGATCGTGCCATTGAGTACCTGATGCCATCAGGGCTGTACGAAAAGAAGGCACGACCACTTATGAAGCCCCCAGAGGAAGTGTTTCCCGCAGAGAAAGCTGCCCAGTTTGATTACACAGGAAGGCCTTTTCATTACCTGTTCTACACAACACGCCCAAACTACTATGGCCTGCTGCATGATGCTGTGAGCAAACTGACAGAGCTCAACAGGCTGGAAAATCGCATGATCAGCAAAGGTGTGCTTCAGGCGGCGGCTGATCAGAAGGAAAACTTGGACGACAGTATGTGGCTGTCGAAGAAAGAGTTGGAGGTCCGTCTGCTGGAG---ACTTTAAAGGACACGCAGTATGATTACTTCGTGAAGACCTTTGAGCGTCTGGCAGAGCACCCCTACTCTTCCCACGCCAGAGAGTTTATCCTCGAGTACAGGAAGACAAGAACCTCCTCT---TTGAAGGAAGACGTCGTGCCGCCCTTGCTGTACGACGAGACGGGGCGGCCGTACATGAAAGCACAAGGTTTCAGAAAACGGACTAAAGCTGAAGTGACTGTGTGGGGGAACGGCACCGGGAAGTTGAGCATCAACGGTCATGGGATCGACTACTTCCGCGAGATCCGCGACAGGGAGCAGGTGATGTTTCCACTGCAGTTCACCGGCCTGCTCATGAAGGTGGACTTCGAGGCCACGGTGGAGAAGTCC---AACGGTTTCATGTGCGAGGCCGGCGCCATTCGCCACGCCCTCGCCCTGGCCCTGCGGAGCTTCGTTCCGATCGAGGAGGTCGAGAGGATGAGGATCGCCGGCCTACTGTCGAGGGACAGGCGTTGCAGGGAAAGGAAAAAGCCAGGCCAA------------------GAGGAGCCCGCCGCAAGTTGA [1032]

Siro TCGGAAGATAAAATCCAGAAAATTAGCAAAGCTATGAAAGCTTACGTCGAGCGAGCTCATGCGAACAATGTGTTTATGAAACAACAAACTGAAGAATTTGAAATGGGAAAACGCCATCTAGCCAACATGATGGGAGTCGAACCACATCAGATGACGCAAAATGATATTAATGAAGCCATTGCTTATTTAATGCCATCAGGATTGTTTGAACCAAAGGCCCGACCATTTATGAAACATCCAGATGAGGTATTTCCTAAACAAAAAGCTGCTGAATTTGACGTTTACGGCCGACCATATCATTTTTTGTTCTACACTGGAAAGCCACAGTTTTACCAACTATTACATGATGTAGGTTGCAAATTTGAAACATTGGATGCATTTCAAGACAAAATGATTCAACAAGGAGTTTTACAACCGCCTCCTGAAATGAAGCTCATCTTAACTGGCAGTGACTGGATTAAAGTAGAGGCCCTTAAGGAAAAACTTTTGGAA---GAATTAAATGAATATGATTACGATTTTTTTCTTGTGTCAATGAAAAGGTTTGTAGAGCATCCATATTCGTATCGAGAAAAAGATTTCATCATGAAATATCGAAAAGTTTTGGCTGTTGGC---GTTTCAGAATTAATTATTCCACCTCTATTGACAGATGAAAATGGACGACTTTATTCAAAAGCCACAGGATATCGTAAGTTCTCGGTGGCTAATGTTACTCTGCGAGCTAATGGAACAGGAAAAGTTAAAATCAATGGCAAAGACATTTGTTATTTTGAAAACCTTCAAGACAGGCATCAACTTCTCTTTCCACTGCAGTTCACTGATTTTCTTCAGAAGGTCGACATTGAATCTGATGTCGAAGGAACG------GGAGACACTGCCAAATCGGGAGCCCTTCGTCATGCCATATCTCTGGCGCTGTTGCCTTTTGTGGAAAAAGAAATGATAGAGAAGATGCGCCTTGCGGGTTTGCTAACGAGGGACCCGAGGTGTAGAGAAAGAAAGAAGCCGGGCCAAAAGGGAGCTCGTGCAAAGTATACTTGGTTAAAAAGATGA [1047]

Sitalc GAGAGTATCCGTCTACAAAAAATTAAAAGAACACTGCGGTTCCATATGGAGAAGATGGAGCGAGAAGGACTAACCATGAAAGAAAAGGAGGCAGAGTTTGAGATTGGACGAAGACATTTGGCAAATATGATGGGGGCAGATCCTAATACTTTCAGCCAGAAGGATATTGACAAATCTATTGAATATTTAATGCCTTCTGGTCTATTCGATCCACGAGCAAGGCCACAAATGAAGCCACCAAAGGAATTGTTTCCACCACTGAAAGGAATTCAATTTGATTCAACTGGAAGACCAATGAATATGTTTTTCTACACTGGAAGGGAAAATTTATACATGATATTTCATAACATTTCAAGTAAACTGGAGGAATTGAACAAGTTTGAAGACCAGATGATTGCCAAAGGCATTAGCAGCCCTCCTCCTGAAACTGCAATATTTATTGATGATTCTCAATGGATCAGCCTGGTAGAGCTGAGAGAGATACTACATGACAAGAAACTTCCAGAGGCGGAGTACAACTATTTGATCGAGAGTCTTGAGCGTCTGGTAGGCCACCCTTACTCCTTCCGGGTCAAGGATTTCATTACCAAGTTGAGGACACCAAAGCTTGCCTATGACCTGTCAAGTTCAAATTTCTCACCACTCACAGTTGGTGATGATGGAGTACCTTATGCAACAGCTTTGGGCTCAAGAAAATTGACCCGTGCCAAGGTTACTGTGAAAGGCAAAGGTACGGGAGAAATAGTTATCAATGGGATGGACATTAGCTTCTTCCCTTTGAAGCAAGACAGAGAACAGCTTCTATTCCCACTGAAGTTTTCTGGTCTACTAAACCAAGTAGATATCGAAGCAACGATAACAGGCCCA---GGGGGTCATTCTGCCGAAGCAGGAGCCCTTCGACACGGCATTGCTCTGGCACTGGGAAGCTTCGTTCCACCAGAAATGGCATCAAAAATGAGATTGGCTGGTCTGATTACTAGAGACATCAGGAAGAGAGAAAGAAAAAAGCCAGGTCAAAGGGGTGCCCGAGCAAAGTTTAAATGGGTGAAACGCTAG [1056]

Sclero GAGAGTATAAGACTGAAAAAGATTAGAAGGGTTTTAAGACAACATTTTCACGCCGCCAAAATGGATGAGGAATTCATGGAAAAGAAAGAAGAGGAATTTCAAATTGGAAAACGGCACTTGGCCAACATGATGGGCATGGACGCTGAAACTATGACCCAGAGTGATATTAATACTGCAATTGAATATCTAATGCCTTCCAGCCTTTTTGAGACACAGGCAAGACCAATGATGAAGCCTGCCAGAGAAATGTTTCCCCCGCCTAAAGGTATGGAATTTGATGCCACGGGCAGACCCTTGAACATGTTTTTCTACACGGGACTAGCCATGTACTATAATGTCCTACATGATATGGCGATCAAATTGGAAGAACTGGAAAAATTTGAAGATAAAATGCTATCAAAGGGATTTGCAGCCCCACCACCTGAAACATTGATTAATTTGGAAGATAGCGATTGGATTTCTTTGGAGGCTTTCAAAGCAAGAACTTTGGAGCCAAATCTTAAAGTGGCCAATTACTACTACTTTATATCTTCCATGGAGCGTTTAGTGGCCCACCCGTACTCGTATCGTGTCAAAGATTTCATCAATGAGTTTAGAGTTCCGAAAATTGGAGCA---AAAAGACTAGCTTCCTATCCACCATTGACTGTTGATGACGATGGAGTGCCTTATGCCACAGCGACTGGAAGACGTAAAGGTACAAGGGCAGTAGTGACAGTTAGGGGCAGAGGATCTGGTTTAGTGGATATCAATGGACACGATATCAGTTTTTTCAAGTTCAAGCAAGACAGAGAGCAACTGCTTTTTCCTTTGAAGTTTTCTGGTATGCTAAATGAGGTGGACTTCAAAGCGGTTGTGACGGGAAAC---GGCGGTCACTCGGCAGAGGCTAATTCAATTCGGCACGGGCTCTCGCTGGCTCTGGGAAGTTTCGTGTCTTCCGAAACTGCCGAAAGCATGAGACTAGCTGGACTTATGAATGCCGACAGAAGAACGAAAGAGAGAAGCCATTACGGCCATAAGGGGGCTCGAGAAGGATACAATTGGAGCAAACGATGA [1053]

Trogulus TTTGAAGATCGTGGAGTGAAAATTAGTAAAGCTATGAAAGCCTATCTAGAAAGAGCTAAGAAACAAGATTCTTTCATGATTGAACAAACTGCTGAATTTGAAATTGGTAAACGTCATTTGGCAAATATGATGGGAACTGACCCTGAAACATTTTCTCAACATGATATTAATAGAGCTATTGAATATTTAATGCCTTCTGGGTTATTCGACTGGAGAGCAAGACCGATGATGAGGCATCCAAAGGAAATATTTCCTGTAAGAAAAGAGGCGCAGTTTGATGAGACTGGACGACCATTCAACACATTTTTCTACACCAATCGAAGTGGTTATTATGGAGTATTGCATAATATTTCAGATTCACTGCAGCAATTAGATATGTTTGAAGATGACATGATTAAAAAGGGTGTAGTTGAACCTCCGCAAGAGAAATTATTAACGCTGAGTGGAAGTGAATGGATTTCCAAAAAAGGATTAGAGGATTTGATTTTAGAG---GAAGTTTCTGAGCAACTTTATAAGTACTTTCTGTTATCAATATCCAAATTGGCTTACCATCCATATGCATACTTGAAAAAGGATCTTATACTAAATTATCGAAAGAACCTTGTTTCTTCT---TCAGATACCATAATAGCTCAACCGCTCACAATTGATGAAAATGGAAGGTCATATGCAAAAGCTATAGGCGGCAGAAAGAAGCTAAGGAACGCTGTTACAGTGTATGGAAATGGAACTGGAAAAGTTGATATCAATGGCAAAGATATATCCTTCTTTCAGTCAATTCAAGACCGGGAACAATTATTGTTTCCCCTGCAATTTACTGGCTTGTTGAACACTGTAGACATTGTAGCGGAAGTGGATACCTCTGTAGGTGGTCACTCGGGCTGGGCAGGTACCCTGAGACATGGTATCTCCATGGCACTCATTAGTTTTGTATCTACTGAAATGGTGGAGAAAATGCGAATTGCTGGATTGCTTCAAAGAGACGTTCGACTAAAGGAGCGAAAGAAACCAGGGCAGAAAGGAGCTAGAGCTAAATATACATGGAAAAAACGCTGA [1053]

Ortho TTTGAAGATCGTGGTGTAAAAGTCAGTAAAGCGATGAAGGCATATCTGGAGCGTGCTCAAAAACATGAGCATTTGATGAGTGGAGAAATTGCAGAATATGAACTAGGGAAACGTCATTTAGCAAATATGATGGGTGCTGACCCGGAAACATTTACGCAACTGGACATTGATAGAGCAATAGAATACTTAATGCCCTCAGGTATTTTTGATTGGAGAGCAAGACCATGGATGAAACATCCAAAAGAAGTGTTTCCAAAGAGCAAAGAAGCACAATTTGATGAAACAGGACGACCATTCAATACATTCTTTTATACCAATCAAAGTGCCTATTATGAACTAATGCATAAAATTTCAGATCAGTTTGAACAAATGAATGAGTTTGAAGATAAGATGATAAAAAAGGGAATTAGTAGCCCCCCTGAAGAAGTTAAAATAAATTTGGCAGAAAGTGAATGGATAACACTAACAGCATTTCAAGAGTTGATTTTAGAG---ACTGTATCGGAACAAAATTTTAAATATTTCATTTTATCAATGTCACATTTCGTGGAGCACCCATATTCTCATTTGAAAAAAGATTTTATAATGCAATTCCGGAAGAATCTTGTGTCTTCT---TTGTCTTATGTGGAAATTCCACCGCTCACTATTGGTGAAGATGGACGACCATTTGCCAAAGCCATAGGATCTAGGAAGAAGCTGCGAAATGAGGTTATAGTTTATGGCAATGGAACGGGCCAAGTCGATATTAATGGCCATGACATATCATTCTTTGAGTCTTTTCAAGATAGAGAACAAATACTTTTTCCTCTTCAATTCACTGGTCTGTTAAATGAAGTAGACGTTGTGGCTAAAGTAGACACTTCAGTAGGTGGGCACACAGGCTGGGCAGGCTCGGTGCGACATGGGATCTCCCTTGCTCTGATGAGCTTTGTAGCAACAGAAATGGTTGACAAAATGAGATTAGCTGGACTTATCCAAAGAGATTTGAGATATAGAGAACGAAAGAAGCCTGGACAGGCTGGAGCCAGAGCGAAGTTTACATGGAAAAAACGTTGA [1053]

Hespero CCAGAGAATCGTGTTAAAAATATAAGCAAAGCTATGAAAGCATATTTAGAAAGAGCTCAGAAACATGATGAGTTTATGGTTGAGCAAACCAGAGAATTCAACATGGGAAGAAGACATTTAGCCAACATGATGGGTGTTGATCCTGAAGCTTTTAGTCAAGCTGATATTAATCAAGCTATTGAATATTTGATGCCTTCGGGTGTATTTGATAAAAAAGCAAGACCTTTAATGAAACACCCCAAAGAAGTTTTCCCAAAGAAAAAAGCAGCGGAATTTGATGTCACTGGTAGACCTTACAATACATTTTTCTACACAGGTCGAAGCAGCCTTTATCAGCTTTTACACACTGTTGTGGAACATATTGAGAAACTGAACAGTTTCGAAGACTCCATGATTAGAAAAGGAATAGCAAACTCTCCACCCGAAATGATATTAAATTTGGGTGGAAGTGAATGGATAAAGATCAATGATCTAAATAAAATTCTTTTAGAA---GAAATACCCGAGCAATATTATAATTATTTCATCATATCCATGACTCGGTTGGCCGAGCATCCTTATTCTTATAGAATAAAAGATTTTATAATGAGTTTTCGATCATCTTTAGTATCTTCA---TTAGCAATGATGGAGATCCCACCGGTAACTGTGGACGAAAATGGTAAAACTTTTGCAAAAGCTGTCGGTAAAAGAAAAAAAATCAGCTCCGAAGTCACAGTTTGGGGTAACGGCACTGGAAAACTTGATATTAATGGGACTGATATTCAGTTTTTCCCATCAATTCAAGACAGGGAGCAGTTGTTATTTCCCTTGCAATTTACGGGTTTTGTAAATAAAATTGATATCGAAGCCACAGTAGATCTTACCAAAGGAGGTCATTCGTCTTGGGCAGGATCCATTAGGCATGGAATTTCATTGTGTCTTAGAAGTTTTGTGGAACCACACGTTGTAGAGGAGATGAGAATAGCTGGATTGTTATCGCAGGACCCCCGAAAACAAGAGCGTAAAAAGCCTGGTCAGAAAGGAGCTAGAGCAAAATTTACTTGGAAAAAGCGTTAA [1053]

Leiobunum ACTGATGATAGAACGGTGAAAGTCAATAGAGCAATGAAAGCTTACCTAGAACGAGCTCAAAAGCATGATGACTTTCTAAAAGAACAAACTGCAGAATTTGAAATAGGAAAAAGGCACTTAGCTAACATGATGGGTGCTGATCCAAATAATTTCACTCAAAATGATATTGATAATGCGATTTGTTACTTGATGCCTTCTGGACTTTTTGATAAGAAAGCAAGGCCCATGATGAAACACCCTAAAGAAATTTTTCCAAAAAGGAAAGATGCTGAATTTGATAAAACAGGAAGACCATTCAATCCTTTTTTTTACACTGGAAAACCTAATTATTATCAACTTCTTTTTAACATTGTTGACAATTTACAAAAACTATATGATTTTGAAGATGAAATGATCCGAAAAGGAATAAGTGAACCATTGCCAGAGTCTAAATTAGATTTAGGTGGGAGTGAATGGCTTAGTTATGAAATGTTTCTTAAAACAATAATGGAA---GAAGTTCCAGATTATAACTATGATTATTTCATCAAATCCATTATAAGTTTATCTGAACATCCTTATTCATTTCGTTGTAAAGATTTTATAATGAAATATAGAAAACCATTGTTTTCATCC---GATAAAAGTGTTAAGATTCCTCCTTTGACGATTGATGAAAATGGTAGGACATATGCAGAGGCGAAAGGTAAAAGGAAAAAAACGCAAGTGAAAGTTGTAGTTTTTGCCAATGGCACTGGAAAAATTAAAATTGATGGAAAAGATTTGACGTATTTCCCTCTCATTCAAGATAGGGAACAGCTGCTTTTCCCGCTTCAATTTACTGGTCTTCTGAATAAAGTAGATATTGATGCTAAAATAATAGATACTAAAGGGGGCTCAAGTTGTCAAGCAGGTGCTATAAGACATGGAATTTCTCTTGCTTTAAGAAGTTTTGTAGATTCGGAGCTTGTGGAAAAAATGAGATTGGCTGGATTATTAACGTCAGATCCAAGACGAAAAGAAAGACAGAAACCTGGTCAAGAAGGTGCCCGAAGAAAATATACTTGGAAAAAGCGATAG [1053]

Proto ACTGATGATAGAACAGTGAAAATCAACAGAGCAATGAAAGCTTATCTCGACCGGGCACAGAAGCAAGATGTTTTTATGAAAGAACAAACTGCAGAATATGAAATTGGAAAGAGACATTTGGCTAACATGATGGGAGCAGATCCGAATAATTTCACTCAAGATGATATTGATAATGCCATTGCATACTTGATGCCCTCTGGACTTTTCGATAGGAAAGCAAGGCCAATGATGAAACACCCCAAAGAAATTTTTCCCAAAAGAAAAGATGCAGAATTTGGGAAAACAGGAAGACCATTCAATCCTTTTTTTTATACTGGTAGACCGAATTATTATCAGGTTTTATTTAATATTGTTGATAATCTACAAAAATTAGATGATTTTGAAGATACAATGATTAAAAAGGGTATAAGGGAACCATTACCAGAGTCAAAATTAGAATTAGGTGGAAGTGAATGGATTAGCTGTGATGTATTTTTAAAACATATAATGGAA---GAAGTTCCCGATTATAAGTATGATTATTTTATCAAGTCTATCACAAGCTTAACTGAGCATCCTTATTCTTTCCGATGTAAAGATTTTATCATGCAATTCAGAAAGCCATTAATTTCTTCT---GATAAAATTGTTGAGATACCACCTTTAACTGTTGATGAGAATGGTAGAACATACGCCGAGGCAGTAGGTAGAAGAAAGAAAACTCAATCTAAAGTAGTAGTTTTTGCAAATGGCACGGGAAAAGTGCAAATCAATGGCAAAGATATGACATATTTCCCTCTCATTCAAGACAGGGAGCAATTACTGTTTCCACTTCAATTTACTGGTCTATTGAATAAAGTAGATATTGTTGCGGAAATAATATCTACTAGAGGTGGTTCTAGTTGTCAAGCAGGTGCTGTACGACATGGAATTTCTCTTGCTCTAAGAAGTTTTGTTGATTCAGAGTTCTTGGAAAAAATGAGATTGGCTGGATTATTAACTGCAGATCCAAGAAGGAAAGAGAGACAGAAACCTGGTCAAGAAGGTGCCCGAAGAAAATATACTTGGAAAAAACGGTAG [1053]

[TICKFAST_NCHAR=681;39S ribosomal protein L28]

Ixodes AAGAAG---TTTGTCCCGGTTTTTGAGCGGTTTCCCGAGCATTACAAGCGTTTTCTGGAAGAACTAAAAAAAACTCCGACTCCCGTTCACTACAAGCCTCCT------AAAGAAAAATATGTGCAGAACCCTGACACGAAAGAAATTACACGAGTGGAAGACATACCCATTCCTGTGATCTATCCCAAAGAATGTCAACAAGGAATCTGGGGTGGAGAAGGGATCATTTCCGGATACCGACGCCGGAAG------TATGGCCGCTACAGCAAGTACTTCACTCCCATCCTCAAGCGTGGCGTCGTATACAGCGAAATCCTCGACAAGTACATGCGTGTGACACTCACCGAGAGGACCGTGTTGCTGATTAACCAAAATTTTGGGTTCGACCAGTACATCCTCAAGACACCGGTGCAGGACTTGAAGTCCCAGCTTGCGCTGGATCTGAGGCGTAAGATGTTGCTGGCTTTGGCAAGGAAAGACTTGTACCACGACGATGAGAAGAAGCAGAGGGAGGTCCTTGAGAAGTACAAGGAGTTCATCATCCCTGAAGAAGAGGCAGAGTGGTTCGGGCTGACACCAGAGCGGGCTGAAAAAAAGCTTATGATGGAGGAGGACAAGGTAAAAGGT---GTACCCCTGAAGTACCAGTTCCTCAAGGACTACCTCGCCGAACTCGAA [663]

Siro CGTTTTACTCAGGCGTATTTGTTAACCCGTCTACCCCAGCAGTATTTAAACTACAGAAAAGAATTACAAAAACCTGCTACACCAGTTCATTATGTTCCTGAA------GAGGGTATATGG---AAGCGTCTGCGGAATGGTACCATAAGACGAGTTGAGAATGTTATGCTGCCTCTTATACGCGTTCCAAAAGAAGATGAAGGCTTATGGGGTGGAGAAGCTATAGTCAAAGGATTTAAGAAGAAAGACGCTAAGAAACGAAGAGTCCCTCACTATTGGGTTCCACATTTGATGAATTCTCTGGTGTACAGCGAAATCTTGAATACACACATGAAAGTAGTTGTGACCAACAGAACGTTACATCTCATCGACAGCCACTATGGCTTCGACAGTTATATTTTAAGGACTCCAGTTCAAGACTTGAGGTCACAGTTGGGGATGGACATTCGAAGAAAATTGCTATTGGCTCTCTTGAGGAGAGACATGTGGCATGATGACCCTGAAAAACAGGAGAGAATCTATAACAAATACAAAGACTGCATTATTCCTGAGGAAGAAGCCGAGTGGTTTGGCTTGTCTTTAGTAGAAGCTCTCAGGAAGCAAAGAAAGATCGAAGTAGAACTTAGCAAACCTGTTCCTTACAAGATTCAATATCGAAAGGAGCTCGTTGAACAACTTAAA [672]

Sitalc CGCTGG---GACCCAGAGATTCTGGCCAAGCTCCCAGAACACTACCACCGCTTCATCCAGGAGGCCACCAAAACACCCACGCCTGTTCACCATATTCCCCACACGGGGAGCAATCTACTGGAGAAGGATCCCGAGACCGGAGAAATCAAACGAGTGCAAGACTGTCCCATTCCCGTGAGTTACCCGAGAGAACACGACTATGGCCTTTGGGGTGGAGAGGGCGTGATTGCGGGCTACGGGAGGCGCAAACCCTTTCATCCACTATTCCCAAAGTTCTATGTTCCCCGATTGTATCGTACCATCGTTTACAGCGAGATCTTCGACACTTACCTGTCTGTCATTGTGACACGAAGAACGTTGAGACTGATCGACGCAGCTTTTGGCTTCGACAATTACATACTTAAAACTCCGTTGGCAGACCTAGGTTCTCAGTTAGCCTTTGACCTGCGTCGCAAGATGTTGCTGGCGTTGGCCAAGAAATCCATTTACCCCAACGACCCCGTTCAGAGGGACAAGGTCATCTCCAAATACAAAGAATGCATTGTTCCGGAGGAAGAGGCGGACTGGTTTGGCCTTCCGATCTTCAAAGCCGTGTCCCGAGCGTTGGAAGAAGACGCCATCTTGAATGCTCCACAGCCTCTCAAGATTCAGTATCGGGCACAACTCATAAACTGGCTCAAA [678]

Sclero CGCTGG---GATCCGGAGATGATCTCTAAATTGCCGGAACACTATCAAAGGTTCATGGACGAAGCAAGGAAGCCTCCGTCCCCGGTTAACTATATCCCAGAGACC---AATAAATATTGGGAAAAGGATCCCAAAACTGGAGAAGTGAAGAGATTTCAAGATTGTCCAATTCCATTAATATATCCCAAAGAATACGATACGGGCCTTTGGGGCGGCGAAGCGATCGTCAAGGGCTTCATGAAGAGAAAGAAAAATAAGCTTAGGTGGCCAAAATACTACGTCCCGTCCTTACTACGACACGTGGTGTATACGGAAATCTTGGACAAATACTTATCAGTGATCTTAACAAACAGGACACTTGAGCTGATCGATTCGCATTATGGCTTCGACAGTTATATCCTGAGGACTCCTCTGGCCGATCTTGGATCTCAGTTGGCATTCGACCTTCGTCGAATGATGCTGTTAGCGTTGGCGAAGAAAAAATTGTACCCCGAAGATCCGGTCAAAAGAAACAAGATACTCAACAAATATAAAGACTGCATTATTCCTGTAGCAGAAGGAGAGTGGTTCGGCTTGCCTTTGGACGAAGCAATGGAAAAAGCATTGACTGAAGATGAAATTGTGAATGCACCAAAACCACTCAAAATCCAGTTTCGAAAAGAACTGATCCAAACACTCAAA [675]

Trogulus CGTTGG---AGAACGGGTATTCTGGCACGTTTGCCTGAGCACTACAAAAGACACAGACTGGAACTGAGTAAACCATCAACTCCCGTTCATTACCAAGCAGAA------GAAGGTCCCTGGAAAATTATGCCAGAAACAGGACATGCAGTTCGAGTAGAAAATGTTGCAATACCAACTCTGACATTTGAACAAATGAACGAAGGAATTTGGGGTGGAGAATCCATCATCAAAGGATTTACAAAAAAGAAGCCAAGAGTACGTAGGAATCCCCATTTCTGGATTCCCAAACTTAAGAAAACTGTAGTGTACAGCGAAATTTTAGATAAATATTTGTCAGTTGTGGTGACTAAGAGAACGCTTTCCTTGATTGATGAGAACTATGGCTTCGACAATTACATTCTTAAGACTCCAGTTCAAGATTTGAAGTCTCAACTGGCTCTACAATTACGGAGAAAAATGATTCTGGCTTTGATT------------CGACCGGGCGATGATCCCAACAAATTAACGCTAGCGGAGAAACACAAAGAACACTTACTGCCCGAAGAAGAAGCGGAATGGTTTGGATTGTCAATAAAGGAAGCTTGCAATAAGCAAATGAATTTAGAAGAGCAAGAAAGGAAACCGCAACCTTTAAAAATTCAATTCAGGAAAGAACTTTTTGAGAAATTAAAA [660]

Ortho CGTTGG---AAAGTTGGAGTGCTATCTCGTCTGCCTGCACACTACCGAAAGCATAGGATCGAACTTTCAAAACCCCAAGAACCCGTTCATTATATCCCAGAA------GAAAGCCATTGGACAACCAAGCCTGAAACAGGAGAAATTGTTAGAAAGGAAACTGTACCAATCCCAGGCATTCTTGTTCCAAAAATGCATGAAGGTCTATGGGGAGGAGAAACTGTTATTAAAGGATACTTCAAAAAGAAAGATAGGGTTAGAAGGGTGCCCCATTTCTGGATCCCATCACTTCAAAAGACCATTGTTTATAGTGAAGTCCTTGACAAATATCTAAGGGTTGTTGTTACAGATAGAACACTGAGAAAAATCGACGAATGCTATGGATTCGATAATTATATATTTAAGACTCCAGTCCAAGATTTAAAATCTCAACTTGCACTGGACCTGAGAAGAAAAATGATTCTTACTTTGATC------------CGACCAGACGGAGATCCAAAAAAGGTTGAACTTGCAGAGAAGTACAAACCTTATCTTTTGCCTGAAGACGAAGCAGAGTGGTTTGGACTGTCGGTTGCTAAAGCATGCAAGAAGCAATTGGAATTGGAGAACACTATTAATAAACCCAAACCTCTAAAGCTACAATTTCGGCAAGAACTTTTAGAGAAACTGAGA [660]

Hespero AAATGG---AAAATCGGCGTCTTGGCACGATTACCAGAGCATTATAAAATGCATCGAATGGATTTGGCCAAGCCCAGAACGCCTGTAAATTATATACCTGAG------GAAGGGCATTGGAAAAGGAACCCAAAAACAGGAGAAATAACAAGAGTTTATAATCTTGCTATACCCTTCGAACATTCTACTAAAATGGACGAAGGTATTTGGGGCGGTGAAGGAATCATCAAAGGTTTCAGAAAACCTAACCCGCTGAAAAAAAGAAATCCGTATTACTGGGTCCCGTCATTAAAACAGCAAGTTTTGTATAGCGAAATTTTGAACGAGTACATGACCGTCGTAGTCACTTTTAGAACCTTGGATTTAATCGATCAACATTACGGATTCGATAGCTATATTTTAAGAACTCCAGTTCAAGATTTAAAATCACAACTCGCCTTAGATATTCGAAGAAAGCTTATTTTGACTCTGATTAAAAAAGATTTCTGGCCTGATGATCCTGTCAAACATAATAAAATCTACGAGAAATATAAAGACTGCATTATACCAGAGGATGAAGCAGAATGGTTTGGACTTAGTCTTAAAGATGCGTGCGGCAAACTTTTAAATCAAGAAAAAGCAGCGTATAAACCGGTTCCTTTAAAAGTTCAATATAGAAAAGATCTCATTGAAAAACTAGAA [672]

Leiobunum CGATGG---AAATTAGGTACTCTTTCGCGTCTACCCGAACATTACAAAAAATTTCGATTGGACCTATCCAAAGAACCCGCACCTGTCCACTATAACCCCGAA------ACAGTTAATTTCAAGAAAAATCCTGAAAATGGACAAATTTTTAGGGTACAAAATGTTCCTATTCCAACTTGGAAAGTACCAGCCGCAAATCAGGGTTTATGGGGCGGAGAAGAAATTATTAAAGGTTTTAGAAAACCAGGCTCA---TATAGAAGGGTACCGTCTTTTTGGATTCCAACTTTACATAAGAAAATAATTTATAGTGAAATATTCGATAAATATCTATCGGTAATTGTTACCCAGCGAACATTGAAGCTAATAGATGAATGTTATGGCTTTGACAACTATATACTCAGGACGCCGGTTCAAGACTTAAAGTCACAACTTGCATTGGATTTAAGACGGAAAATGTTACTAGCTTTAGTTAGGAGAGATATTTACAAAGATAATCCAGAGAAACAAGAAAAAATCTTGAAAAAATACGGGGATTGTATTATTCCTGAAGAAGAGGCTGAATGGTTTGGTTTGAGCTTAAAAGAGGCGTGTAAAAAACAACTTAACATTGAGAAAGTATTAAATAAGCCTGTTCCGTTAAAAATTCAGTTCCGAAAAGAACTAATAGCTCATTTAAAA [669]

Proto CGGTGG---AATTTGGGTACTTTTAAGCGTCTACCCGAGCACTACAAAAAATTTAGAAATAATCTAAACAAGGAATCCACACCTGTCCATTATAAGCCAGAA------ATAGTTAATTTTAAGAAAAATCCAGAAAATGGACAAATTTTTCGGGTACAAAATGTACCAATTCCTACAATGAAAGTCCCAGCAGCAAATCAAGGGATATGGGGAGGAGAAGAAGTTATTAGAGGTTTTAGAAAACCCGGTTCA---TATCGAAGGGTTCCATCATATTGGATTCCTACTTTGCATAAAAAAGTAGTTTATAGTGAAATATTTGATAAATATTTATCGGTAGTCGTTACCCAAAGAACTTTGAATTTAATTGATGAATGTCATGGTTTTGATAATTATATTCTCCGGACACCTGTTCAAGATTTTAAATCACAACTAGCGCTTGATTTAAGGCGAAAAATGTTACTAGCGTTGGTTAGGAAGGATTTCTACAAGGATAATCCTGAAAAACAAGAAAAAATCCTACAAAAATACGCAGATTGTATTATTCCAGAAGAGGAGGCAGAATGGTTTGGTTTGAGCCTAAAAGAAGCCTGTAAGAAACAACTTAAAATTGAGGACGAATTAAATAAGCCTGTTCCTTTAAAGATTCAGTTCCGACAAGATCTAATAGCCTATTTAAAA [669]

[TICKFAST_NCHAR=801;39S ribosomal protein L44]

Ixodes ATGCTGATGGAAATGAAAAGACGCCGCAAAAAGGCCGGACCGGAGCCGCTACGCCACCGCTCGGTCTGGGTCGAGTGGAACTACGACTGCGAGATCTACGCGTTTGCCAAGCGTCTCGGCGAAACCTGGTCCGACGAAACGCTGCGCACCGCCTTCGTCCAGCAGTCTTTCGTCGAACGCGAAGCCGAACACCGTCGC---GAGCTGGGAATGCCACTGGGCGACGACGGAACGCTAAGCCTAACTCCCAACACCGACCTCGCCCGCGCGGGCCGCCAGCTCTGCGACTCTTACGCCGACGAATACTTGGTTAGAGAGTTTCCGCAACTCCCACGCGAAGGAGTGAACGCCGTGAAGGAGCACCTAATGTCCGACCAAGTGTTGTCGCACGTTTCGAAAAACATCGGGACCTCGGATTTGATCCTCTGCGCCGACTTCCCGCCGGAGCAGACGACTCTTGCCAACGTCCTGATGGCTTTGGTCGGCGGACTGGCCCGCGACTGTGGGAAGGACCGGGCCTGTTTGTTTGTGCGGGACTTCGTACTGGCGCAGTTGGTCGGCAAAGACGTGAACGAACTGTGGGTCGTGGACAAGCCGATTGAGAGGCTCGCGTCTCTACTTCAAGGGCAGGGGATGAGTCCTGCTGAACCCAGACTACTGTGGGACTCGGGCCGAACCACGCTGGAAGCGAGCTATTGCGTCGGGTTCTATTCCGACAAGAAGCTCCTTGGTTCGAGCGTCGGCGAGACTGTGGAGATTGCTCAAGAGATGGCAGCACGCGACGCCCTTAGGAGGTGGTAC [798]

Siro ATCATGAGAGAATTTAAACACCGACAAGAAAAAGCAGGACCAGAACCATTACGCCATCGTTCAGTCTGGAATAACTGGAACTACAATGCAGAACTGTATGCATTTAGCAAGAGACTGCAGGAAGAATTCAACGATGCAACTCTAAGAAGAGCGTTTACACATCGGTCTTATATCGAACAGGAAGATGCTAAACGAAAA---GAACTTGGGGTC---CAGACAGATGACATTCATTTGAATTTGCAAGATAATTCTGAGCTAGTGCAATTAGGAACAGAGATCATGAAAAACTATATAACCAGTTATTTAAGGCACGCATATCCATTGCTCCCAAGTGAAGGCATTAGTGCTGCCACAGAATATTTAATGGACGAAAAATTAATTGCAATGGTGGCATCAAACATTGGAATTCCGGATTTGATGTTGTGTGGAGACTTTCCACCAGAAAAGTCCACTTTAGCCAACAACTTCAAGGCCATTGTTGGAGCGATAGAAAGTGACCAGGACCGAAACCGAGCAGAAATATTTGTGCAAGACTTTATCCTGACACAGCTCATTGGCAAAGATATCAATGAAATTTGGGAAATTCCAAATCCAATGGCACTATTAAAGAAAATCCTAGGACAACAG---------GAACTTGAACCAAGATTGATCAGAGAAACTGGTAGAAACACACTGTTCGCTGTATATGTTGTTGGCCTGTACATCAATAAGGAGCTGTTCAGTGAAGGAGCTGGAGAGTCTGTTACAATAGCAATCGAGATGGCTGCCCGAGATGGACTAAGAAGGATTTAC [786]

Sitalc ACAATGAACAAAAGAAAAGCTGAAATCCAGAAAACTTCACCACCTCCACTAAGACCAAGATCAGTCTGGATTAACTGGAACTATAGAGCAGAGATATTCGCATTTGGAAAGCGTTTGAGTGAGGATCTTCCCAAGAACTCTTTACAGAGGGCCTTTATCCATCCATCTTACGTGGAAAAGGAAGCAGCCAAGAGGAAA---GAAATGGGAATT---GAATCCATA---GCTGATGAAACCATGGAAGATAATTCTGCACTTGTCAAAGAAGGTGAAAAGCTGATGGCTGATTTTCTACAGTCTTACCTTCAGATGGCTTACCCTCTTTTACCACTGGAAGGAAGAAAGAAATTGCAAGAACATCTTTTGGAAACCAGTCTGATAGCTCACATAGCCTCGCATCTTGGATGTGCCGATCTCATTGTAGCTGCTGATTTTCCAGCAACCAAAGAAACACTTGCTTCCACGTTCAAGGCGTTAGTAGGAGTTATAAAAGCTGAAGGGAGTGTAAAGAGGGCGGAAGTATTTGTGCAAGATTTCGTCATTCCTCAGCTTCTGGGAAAGGATGTTCTGGAAATCTGGGACGTCCCAAATCCAACTGGTTATCTCAAAGACTTGCTTCAGCAGTCAGGAAAG---GATCCTGAACCAAGGTTGCTTAGGGAAACGGGACGGAACACCATCTTTGCGGTCTATGTTGTAGGCATTTACGTGGACAGAGAACTCATAGGCCAAGGGCCAGGAGAAACGCTGGCCATAGCCGAAGAAATGGCCTGTTTAGATGCACTGAAGAGGTTATTT [789]

Sclero ???????????????????????????????????????????????????AAGCCGCGGTCCATCTGGATAAACTGGAACTACAAGGCAGAACTGTTTGCGTTTGGGAAGCGTCTGAATGAAGAGCTGCCTGAGGAGGCTTTGCTGCAGGCATTCACCCACAGTGATTACATTCAAGCAGAGGAAGCCAAACGGCGA---GAACTTGTGGCT---TCTTCAACCAATGTAGCACTGGGTTTACAAGACAACACCGAAGCAATTTCAGATGGAGAAAAAGTAATGAGCAGTTTCATTGAGAAATACCTTCAGGCATTATATCCCCGATTACCGCAAGAAGGCATAAACTCTCTTCATGATCACTTGATGAGTGATGACCTTATTTCCAGCGTTGCCTCGCTTCTTGGATTCACAGATTTAATTTTGTCAACTGATTTCCCACCAAAAAAGACGACCATTTCGGCTACTTTTAAAGCTGTTGTAGGAGTCATCAAGCAAAACAAGGGTCTCGGAAGGGCAGAACTTTTTGTGCAAGATTTTATTTTGCCAGAACTCATAGGTAAAGATGTCCTAGAAATTTGGGAGATTCCCAACCCAGTTGGCTATCTGAAGAATATGATTTCTACTTTGAACAAA---GAACCTGAAACCAGGTTGTTGAGAGAGTCAGGAAGGAACACTTTGTTTGCAGTGTACATGGTTGGCATTTACGTGGATAAAGAGTTAATTGGAGAAGGATCTGGTGAAACGTTGGGCATTGCAGAAGAAATGGCTGCTATCGATGCCATCAAAAGATTGTTC [792]

Trogulus TTAATGAAGGCTTTAAAAAAAAGAAAGGAAAGAGAA---GTTGAAGTATTACGACGACGTTCTGCATTCATAAATTGGAACTATTCTGCTGAACTTTTTGCATTTGCTAAAAGGTTAAATGAACCATTATCTGAAAAGACTCTACAAACCGCATTAACACATACATCATATATTGAACAAGAAGAG---AAACATTTAGGTGGACTTGGCATA---GAAAATGCT------TCACTTAATTTGGAGGATAATAAAGAATTGATCAAATTAGGGGAGCTATCTATGGAAAATTATATATTGAAATACTTGAGGCATTCATATCCACATTTACCAGAAGAAGGCATACGATGTGTCTATGAATTCCTGATCACAGACTCCTTGTTATCACACATTGCTAAGAACCTTGGGCTCACAGAGTTAATATTGTCAGCTGACTTCCCAGTAGAAGATAAAACACTGGCTTCCACACTGAAAGCACTTGTAGGGGCTATATCTACAGACAGGAACACCGAAGCAGCACAAAATTTTATTTTGGACTTTATCTTGACTCACTTAAGTGGAAAGGACATTCATGACCTTTGGGAAATCCCAAACCCAATTGCAATGCTTAAAGATATCTTACAGAAATCAGGAAAATGTGAACTTGAGCCAAGACTTCTAAGGGAGACTGGACGCAACACTATTTTTGCAGTTTATCTGGTTGGGATTTATTTCGATAAGGAACTTATAGGACAAGGACCTGGTGAAACTTTAGAAATAGCACAAGAAATGGCTTCGTTGGATGCATTGAGAAGAATATTT [786]

Ortho TTGATGATGACACTTAAGAAGCGGAAAGATAAGGAA---GAGCCAGTCCTAAGGCCACGATCTGCCTTTGTAAACTGGAACTATTCAGCAGAACTCTTTGCTTTCACAAAACGCTTGAATGAACCATTATCCGAAGAATCCATACGAACTGCATTCACTCACAAGTCCTATGTTGAACAAGAAGAA---AAAAGTCTAAAGGAGCTTAGCACC---CAAGAAACC------TCACTGAATTTATCTGATAATGAGAAATTAATTCATTTAGGACAGGATGTTATGGACTCATACATAAAGAAATACTTGAGACATTCCTATCCGTCTTTACCAGATGAAGGCTTACTAACCATTTATGAGTTTCTACTCAAAGAAGATTTGTTAGCATACATTGCGAAGAACCTTGGAATGGGCGAACTAGTCCTTTCAAGTGATTTTCCGGTGGAACAGAAGACTCTGGCTACTACGTTTATGGCTATTGTAGGAACTATTGCTAATGACCGGGGAATTGAAGCCGCACAGAACTTTGTGTTAGATTTTGTATTGACTGAATTGAGTGGAAAAGACATTCACAGTATTTGGGACATTCAGAATCCTTTTGGAATGCTTAAAGATATTTTGCACAAAGATGGAAAATCAGAACCAGAACCAAGGTTGTTAAGAGAGGCCGGGCGTAACACCATATTTGCTATTTATGTAGTTGGTCTATATGTTGATAAGAAACTTATTGGACAAGGGCCTGGTGAAAGCTTGGGTGTAGCCCAAGAAATGGCAGCCTTAGATGCTTTACGGAAAATATTT [786]

Hespero GTTCTTAAAGAATTAAAAGAAAGGGAGAAAAGAGCT---GTCAAAATTGTCATACCTAGAACACAGTTTAAAAATTGGAATTATACAGCTGAACTGTTTGCCTTCACAAGCCGACTCAATGAGAGTATCAGCGATGAGAATTTACGCACAGCATTTATATATTCGTCTTACGTTGAAGCAGAGGAA---AAACAAAGACAGGAATTAGGGGCT---GAATCCGCT---------CTGAATTTGAGCCATAATGAACAACTGGCTCTGTTAGGAGATAAAATTATGGAGGATTATATTGTGCGGTATCTGAGAAAGTCTTATCCGCTTTTACCTGAAGATGGAATTTTGTGTTTGAAGGATCACTTGATGAGTCCTGAATTAATAGCTTATGTAGCAAAAAAATTAGGATTTTCAGACTTAATCCTAACTAAAGAATATCCTCCCGAAACTCAAACATTGGCTACAGCTTTTAAAGCAGTTGTAGGAGCCATTGTTAAAGATAATAATATAGCAAGGGGAGAGAAATTTGTTCTCGATTTCATCATAACTCAGCTAATCGGCAAAGATATTCACGAAATTTGGGAGATCCCAAACCCTATGGGAATGCTGAGGGTTGTTTTAGAGAAACAAGGCAAATCCGAACCCGAGGCTAGACTGCTTCGAGAGTCTGCGAGAAACACGATTTTCTCTGTATATTTGGTGGGCATTTACGTAGATAAGGAGCTGATAGGAGAAGGACCGGGCGAAACAGTAGTGATAGCGCAAGAAATTGCAGCAATCGACGCGTTACGTAAAATATTT [783]

Leiobunum GTTTGGAGGGAAATGAAACATATAAAAGCATTGGAA---AAAGAGGTACTCAGACCACGATCTGCATTTGTTAACTGGAACCATTCGGCTGAAATGTTTGCCTTTAGTAAGCGTATCAATGAGGAACTCTCTGAAGAAACTTTACGTCAGATCCTTATTCACCCATCATACATTGAGAAAGAAGTG---AAGTCCAGGAAAGATCTTGGAGTA---GCTTCAGAT---TTAGTTTTAAATCTTGTTGATAATACTGATTTAGTTAAAATTGGTGCTCCTTTTATGTCTAATTTTGTGTCATCATACCTAAAAGAACAATTACCAAATTTGCCTGAAGATGGATTAATTACTCTACATGATTATTTGATATCTGAGGATAACCTGTCAAATATAGCACGAAATATTGGTATTACAGACCTTATTTTGTGTGCAGATTTTCCAGTTGAAAATAATACTTTATCTATAACTTTGCAAGCTTTAGTTGGTGCAATTTTAAAAGATGAGGGAAATTTAAAAGCACAAAGCTTTGTCCTAGAAATGGTGCTAACGCAGCTTATAGGCAAGGATATAATGGATATTTGGGATATTCCTAATCCAATGGGAACTCTTCGGAATATTATATTTAAACAAGGAAGATCGGAACCAGAGGCCAGGCTGCTGAGAGAAACTGGGAGAAACACAATATTTTCAGTTTATGTAGTTGGCTTGTACATAGATAAGGAATTAATAGGACAAGGGCCTGGTGAAACAATTCAAACTGCAACTGAAATGGCAGCCTTGGATGCTCTAAAAATAATTTTT [789]

Proto GTGTGGAAAGAAATGAAAGTAAGGAAAAAATTGGAA---AAGGAAGTACTCAGACCACGATCTGCATTTTCTAACTGGAACCATGCTGCAGAAATCTTTGCGTTTAACAAGAGGATCAATGAGGACCTCTCTGAAGAAACCCTTCGTCAAACTCTCGTTCATCCATCCTATATTGAGAAAGAGCTT---AAAGCCAGGGAAGAAATGGGAATG---GCTACTGAT---ATAGCCTTTAATCTTACAGATAATACTGATTTAGTTAATATTGGCACCCCATTTATGTCAAACTTTTTGTCTTCGTACTTAAAAGAGCAATTACCACACTTACCCGAAGATGGATTAATAGCTATACGTGAATATTTGATGTCCGAAGACAACCTGTCAAATATTGCTCGGAATATTGGAATGACAGACCTTGTTTTGTGTGCTGATTTCCCTGTTGAAAATAAGACATTATCCACGACTTTGAAAGCTCTAATTGGTGCAATTTTAAAAGATGAGGGAAACAATAAAGCCAGAAAATTTGTCTTAGAATTAGTAATAACTCAACTCATAGGCAAGGATATAATGGATTTTTGGCATATTCCTAATCCAATGGGAACACTGCGTAATATTTTAATTAAACAGGGAAGGTCGGAACCAGAGGCCAGGCTGCTAAGGGAAACTGGGAGAAACACGATTTTCTCAGTTTACATAGTTGGCTTATACATTGATAAAGAATTAATAGGACAAGGGCCTGGTGAAACAATTCAGGTTGCGACTGAAATGGCTGCCTTAGACGCTTTGAAAATCATTTTT [789]

[TICKFAST_NCHAR=375;39S ribosomal protein L51]

Ixodes CGGAGGTACGGCTACGAGAACCCCCTCATGGAGCCAGGGTTGCTGCCCAGACTGCCAAAGTTAAAAAGGAAGCTCAAGAGCCAACCTGTGTACGCACCCAAAGACGCCTGGTGTGAACGCCGTGCCCTTTTCGGGCAGAACGATTACATTGACATCCTGGGAAGCGGGGCCCTGAAGCCGATCCACATCATGCAGAATGTGCCCCAGTGGCTGCGGGGCTTCCAGGGCAATGAATACCAGATGCTGCTCCGGCGGAGGAGGGTCAACCAGGAATGGAAGGAGACAAGGCCCACCCGCTACCGCAACATGGCCAAGCGCATTCGCTACCTCTACAAGGTGCTCAACCACAATACAAAG------------TCCTGA [363]

Siro AGAAGATTCGGTTACGAAAATCCGATTTTCGACGGAGGACTCCTGCCTCGGCTTAAAGACGCTCCAAGAATTCGT---CAACAACCTGTTTACCGACCCAAAGATCATTGGCATGAGAAGAAAGCTCTTTTTGGCCAAAATGACTACATTGATATTCTTGGTGACACCAACACCCACCCAGTGCGATTATTGACTAATATGCCGAGGTGGTTGAGAGGATTTCAGGGTAACGAATATCAAATGCTTCTTCGCAAAAGAGTTGCCAAATATGGCTGGAAATGGTCTCGACCAGCCAAATTTCACGACTTGGACAAACGTATTAAATTTCTTTACAAGCGACTAAATTACAAAACAAAG------------ACTTAG [360]

Sitalc CGGCGAATGGGATACGAAGACCATCTGATGCCTGAAGGTCTATTACCTCGAAGTAAAGATGCAAAGCCTTACAAA---GGAATGAAAAAGTTTGAGGTTACGGATCGCTACACAGACGAAAAAGCCCATTTTGGACAGAATGATTACATTGATATCTTGGGTGAGGGGGACATCAAACCGATCATGCTGGCCAAAAACATCCCAGAGTGGCTCCGGGGATTCCAAGGAAACGAATTGCAGATGCTCATCTTAAAGAAAAGAACTCATGCCCAGTGGAAATGGCTCCGGCCCCAGATGTGGCACAATTTGAACAAGAGGATAGACCGTCTCTACAAATACTACATCTACAAAACCAGG------------AAGTAA [360]

Sclero CGCAAGATGGGCTTCAAGGATAACCTTATGCCTGAAGGATTACTTCCACGATCGAAAGACTTTAAACCCTACACC---AACCTGAAGCGATACACCCCAGTCGACAGGTTCTCCGATTATAGAGCGCATTTTGGACAGAACGACTATATAGATATCTTGGGGGACAAGCCGATCCAACCCATCAAACTGGCCACCAACGTCCCTAACTGGCTGCGAGGGTTCCAAGGGGACGAGTATCATATGCTGCTTAAAAAACGAACAGCGTTCCACCACTGGCAGTGGCTGCGGCCCAAGATGTGGAGCGACCTCACCAAACGCATCAACTATCTCTTCAAGTTTCACATTCATAGAAGGAAA------------CCTTGA [360]

Trogulus CGCAGACGTGGTTATGAGGAAAAATTATTTCAACCTGGTTTATTGCCAAGACTGAAAAATGCTAAACCATTACCT------ATACCAAAATACAAACCAGTTGATAGATGGGCTGAAAAGAGAGCACTGTTTGGCCAAAATGATTACATAGATATATTGAGTGACAAGAGAATACATCCAACCCGTGTGTTAGCTGATGTCCCCAATTGGTTAAAGGGTTTTCAGGGAAATGAATTTCAGATGTTGATCAGGAAAAGAAAATTCTTTCCCTATTTAAGATGGACTAGGCCAAAAGCCTATCATGAATTAAACAAACGTATAAAATATCTTTTTAAGAGACTTAACCACAAAACAAAA------------ACCTAA [357]

Ortho CGGAGGCGCGGATATGAAGACAAATTGTTTCAACCAGGTTTGCTACCAAGGCTGAAAAACAGCCAACCATTAGCT---GACCTACCAAAATATCAACCTGTTGATCACTGGGCTGAAAAACGAGCTTTGTTTGGTCAAAATGATTACATTGATATACTTGGGGACCATAACATTCATCCTACACGTGTGTTGAAAGATGTTCCAAACTGGTTAAAAGGATTTCAGGGGACTGAGTTTCATATGCTTATTAGAAAAAGAAAGCATTTTCCTGACTGGAAATGGACTAGACCAAAGTCTTATCATGAAATGAACAAACGTATTAAATATCTCTTTAATCGTGTGAACAGAAAAACTAAA------------ACATAA [360]

Hespero AGGCGATATGGTTATGAACTCAAGACATTTGAAGGAGGTCCTTTACCTCGGATTAAAGACGAACCTCTTAAGAAA------TTGCCCCTTTATAAAGCCTATGATGGTTGGGCTGAAAAGAGAGCCCTGTTTGGACAGAATGATTATATCGATATTCTTGGTGATAAAAATATTCACCCTGTTGAACTATTGGTGGACATTCCTAAATGGCTAAGAGGATTTCATGGACGAGAATACCACATGTTATTGAAAAGGAAACAGGCTTTTAAACATTGGAAATGGACACGGCCAAAACAATGGACCGAAATGCTTAAGAGAGTTCGATACTTATACAAAAGATTAAATCGAAAAACAAGA------ACTTACCATTAA [363]

Leiobunum AGAAGATATGGATATGAAAATCCCATAGTGGAAGAAGGTTTGCTTCCAAGAGTAAAAGATAGACCATTACAATCA------CTACCCAGATTCAAAGTTCGGAATGTATGGGCTGAAAAAAGAGCTCTGTTTGGGCAAAATGACTATATTGATATTTTAGGAAATGGTAAAATTCATCCAACTAGAACAATGACAGATGTACCACTCTGGTTAAGAGGATTTCGTGGAAGTGAAATGCAGATGTTAATCAGAAAAAGAAAAGCCCACTATTATTGGAAATGGACAAGACCTAAAGCCTTCAAGGATTTGAACAAGCGTGTAAACTATTTATATTATAGGTTAAATCATTACACAAAAGACAATGTTTTTGGATAA [369]

Proto AGGAGATATGGCTATGAAAATCCCTTAATGGAAGAAGGTCTGCTACCAAGATTAAAAGATAAACCAGTAGTATCG------CTACCCAGATTCAAAGTCAAGAATGTATGGGCTGAGAAAAGAGCTCTGTTCGGTCAAAATGATTATATTGATATTTTAGGTGATGGCAAAATTCACCCAACTCGAACAATGACGGATGTACCTATGTGGCTAAGAGGATTTCAAGGAAGCGAAATGCAAATGCTTCTCAGGAAACGGCAAGCCTTTCACTATTGGAGATGGCTGAGACCTAAAGCATTTAAAGATTTGAACAAGCGCGTAGGATATTTATACAGGAGATTAAATCGGAATACCAAAGACAATATTTTTGGCTGA [369]

[TICKFAST_NCHAR=516;3-hydroxyanthranilate 3,4-dioxygenase]

Ixodes ACAGTGTTCTACGTAGGAGGTCCAAACGTGAGGCGAGACTACCACATCGAGGAAGGCGAGGAATTCTTCTACATGCTCCGCGGGGACATGGTCCTCAAGGTGCTCGAGCGCGGACGTGCCAAGGACGTGGTCATCCGGGAAGGGGAGGTGTTCCTGCTGCCCGGGCGCATCGCCCACTCGCCCCAGAGGCTCGCGGACACCGTGGGACTCGTCGTGGAACGGGAACGTGCAAGCCACGAACAGGACTGTCTCAGGTTCTACACAGACGACACGTGCAGCCAGGTGCTGCACGAGCGGTGGGTGTACTGTAAGGACCTCTACCACGACCTGGTGCCCCTCATCAACGAGTTCCTGGGCTCCGAGCAGTGTCGCACAAACCGGCCCGGACCCGGGTCCTTCCTGGGCAAGCCGGCCTACGACGAGAACACAGAAACCACGCTGTCGCCGCCCTTCAACTTGAACCAGTGGCTCCAGAGGCACGACAACCTCCTG------AGCCAACCCAACGCTAAG [510]

Siro AAAGTATTTTTCGTCGGTGGACCTAATCAACGCCAAGATTTCCATCTAGAAGAAGGCGAAGAGTTTTTCTATATGCTTCAAGGAGACATGGTCTTAAAGGTCATGGAAAGGGGTCAAATACGTGACGTCAACATCAAACAAGGTCAGGTTTATCTTCTGCCCGGTCGTATCCCGCATTCACCTCAACGAATGCCAGACACTATGGGTTTGGTGATTGAACGTGAGCGTTCGGCCCACGAGATGGATTGCCTCAGGTACTTCGTT---GAG---AGCGGACAGACCCTTTTCGAGCGTTGGTTTCACTGCGACGATCTCGGCAGTCAACTGGGTCCAATCATTCAGGAATTCCTTGCATCGGAGCAACATCGCACTGGCAAACCCATCGCTGGCGCAATCACTGAAAATCCTCCTTATGACGTGGATAGTCGACGTCACTTGGAAGAAGCATTCTCGCTTTCTGATTGGTTGAAAGAGAATCGTCAGGACATC------GTCCGTCATGGCAAGAAG [504]

Sitalc AAAGTTTTCTTTGTGGGAGGACCAAATATCAGAAAAGATTTCCATCTTGACGAAGGAGAAGAGTTTTTCTATATGCTGAAAGGGGACATGTGCCTTCGAGTTTTAGAGAAAGGACAGTTCAAGGACATCCACATCAAAGAAGGGGAGTGTTTCCTGCTACCCGGCCGAGTACCCCATTCGCCGCAACGGCAGGCGAACACCGTAGGCCTGGTCATCGAAAGAGAGAGAACCTCCACTGAAATGGACTGTCTTAGATACTACGTGGGGGAC---AGCACGGATATTTTGTACGAAAGGTGGTTTCACTGCGAAAACCTGGGCACCCAGCTGCCACCCCTCATCAAGGAGTTCTTTGCATCTCAAGAATATCAAACTGGGAAACCTCTTCCAGGAAGCGTGAGAACAGTGCCACCTTTCTGGGACGATGACCAACGAATTCTGGACCAACCCTTTTCTTTAAACCAGTGGCTGACTGACAACGCCCCGACTATC------CACGAAAAGGGACAACTG [507]

Sclero AAAGTATTCTTCGTGGGAGGACCGAACGTTAGAAAAGATTTTCATATCGAAGAAGGAGAAGAGTTCTTCCTAATGTTGAAAGGCAACATGTGCCTGAAAGTGTTGGAGAAGGGAGCATTCCGGGACATACATATCAGAGAAGGAGAAGCCTTCCTACTGCCGGTGCGTATTCCCCACTCCCCCCAACGGCAGGCCGACACCATGGGACTTGTCATAGAACGCGAGCGGCAACCCGAAGAGATGGATTGTCTAAGGTATTTCGTAAATGAC---TCCACGGACGTTTTATTCGAACGGTGGTTCCATTGCGTCAACTTGGGAACACAGCTTGGGCCCATAATAAACGAGTTTTTCCAATCCGAGGAATTCAAAACGGGAAAGCCCAATCCAGACAGTCTTGCCGCCAAACGTTCTTTCGAAGTGGACTGTGAAAGGACCCTTGGGTCACCTTTCTCGGTCTCGGAATGGCTTCGAAACAACAGAGAGAACATT------TCGCAACGGGGAAGGATG [507]

Trogulus AAAGTCTTCTTCGTGGGCGGACCCAACGTCAGAAAGGACTTCCACATCGAGGAAGGAGAGGAGTTCTTCTACATGGTCAAGGGCGACATGTGCCTGAAAGTCCTGGAGAGAGGACACTTCCGGGACATTTCCATCAAGCAAGGAGAGGTCTTTCTGTTGCCGAGTCGCATTCCTCACTCTCCTCAGAGGTCAGCGGAGACCATCGGCCTGGTGATCGAAAGGGAAAGGAGCTCGCAGGAAATGGACGGACTCAGGTACTTCGTCGGCGAC---ACCACGGAAGTCCTCTTCGAGCGCTGGTTTCACTGCCAAGACCTCGGACCTCAGCTAACCTCCGTGGTCCAGGAATTCTTCAATTCTCAAGAATACAAGCTGGGGAAACCA---CTGACAACCGGAAAGTCGGTGGCGCCTTACGAAGTGGACGCATCGAGGACACTGGATGACCCTTTCCTCTTGGGAGACTGGATTCGGGAGCATCGATCCCGCCTC------TCCCAACCGGGCGGCCTG [504]

Ortho AAAGTGTTTTTTGTTGGCGGACCAAATGTAAGGAAAGATTTTCATATTGAGGAGGGAGAAGAGTTCTTCTATATGCTGAAAGGGGATATGTGCTTGCGTGTTGTTGAGCAAGACGCATTCAAGGACATACATATCAAAGAAGGAGAGGTCTTTCTGCTGCCGGGTCGCATACCGCACTCCCCTCAACGAAAAGCCGATACCTTTGGTCTGGTCATTGAAAGAGAACGAAAACTGGATGAAATTGATGGCCTGAGGTATTTTGTGAACGAT---ACTACAGATATACTTTTCGAGCGCTGGTTTTATTGTGACGATCTAGGAGGCAAACTTTCAGCTATTGTTAAAGACTTTTTCAACTCCGAAGCCTACAGAACGGGCAAACCG---CAGAATGAGCAACGTCTGGTGGCTCCTTACGACATAGACCACAGGAGGTCTCTGGGCAAACCGTTCTCTCTGAAAGAGTGGATTTCAGGTCATGGTTCTGAGATC------TGTAGGCCAGGTGGTTTG [504]

Hespero AAAGTTTTTTTCGTTGGAGGTCCGAACAGCAGAAAAGATTTTCATATCGAAGAAGGAGAAGAGTTATTCTACATGATAAAAGGTGACATGTGCCTTCGAGTTTTGGAACAAGGGCAATTTCGAGACATTCATATACGGGAAGGAGATGTATTTCTATTGCCTTCAAAAATTCCTCATTCTCCTCAACGCATTGCCGATACTGTGGGCTTGGTCATTGAACGAGAAAGAAAATTAGATGAATTAGATGGACTAAGATATTTCATA---GATAATTCCAATGAAGTACTGTTTGATAGTTGGTTTTATTGCGACGATTTGGGACCCCGGTTGGGCAAAGTTATTGAAGATTTTTTCCAATCTGAAGAACATCGTACAGGAAAACCT------TCCAAAGCTATAGCACCTGCTCCGTACGACGTGGATAACGTTCGCAAATTAGAAAACCCATTTTCCCTGAGTAATTGGATAATTGACCACGAAAAAGATCTGAATTGTGAAAAAACTTCTCGTCAA [507]

Leiobunum AAAGTATTTTTCGTTGGAGGTCCAAATAAAAGGGCAGATTTTCATATCGAAGAAGGCGAAGAGTTTTTCTACATGATAAAAGGAGATATGTGTTTGACCGTCTTGGAGAAAAATACTTTCAGAGATATTATTATAAAAGAAGGAGAGGCGTTTCTACTTCCAGGTCGAATTCCACATTCTCCTCAGCGTAAACCTAATACGATTGGGCTTGTCATTGAAAGAAGAAGGGCCCCCGAAGAGATGGACTGCTTGAGGTATTACGTG---GATGGAACTAAGGAAGTGTTATACGAAACTTGGTTTAACTGGGAACGTAGCGCCTCGAAACTGGGCGAATACATCGAGGATTTTTTCCGATCCGAGCAATTCCGAACAGGAAAACCGTTGCCGGGTTCCATAGATATTGAGAAATCTAAAAACGTCGACTCTGAAAGAGAGTTGGAATCGCCCGTTTGTTTGACAAATCAAATTGCAACGGTGCTTAGCGACGACAACGTAATGAAACGGTCTTCGATA [513]

Proto AAAGTATTCTTTGTCGGTGGTCCAAATAAAAGAGCAGATTTTCATATTGAAGATGGCGAAGAGTTCTTCTACATGTTAAAGGGAGAGATGTGCTTGACAGTCTTGGAAAAGAACACGTTCAAGGATATTATCATAAAAGAAGGAGAGGCGTTTCTGCTTCCGGGTCGAATTCCTCATTCGCCTCAGCGCAAGCCGGACACRATTGGACTGGTCATTGAGAGAAGAAGAGCTCCTGACGAAATGGACTGCTTAAGATATTACGTGGACGAG---ACGAAAGAAGTTCTGTACGAAACTTGGTTCAACTGGGAACGCAGCGCTTCGAAACTCGGCGAATACATCGAGGAATTTTTCCGATCGGAGCAGTTCCGAAGCGGGAAACCGCTCCTGGGTTCTATTAACAGCGGGAAATCGGAGAACGTCGACAGCAAAAGAGAGCTCGGATCGCCGATTCGTTTGCGTGATCGAATCGACGACAACGCGGAGACAGAG---------------TCACGGATA [498]

[TICKFAST_NCHAR=2775;4SNc-Tudor domain protein]

Ixodes ATGGC------------------------------------------------AGGCATCGTCAAGCAGGTCCTGTCTGGGGACACGGTAGTGATCCG---TGGGCAGCCCCGGGGTGGTCCGCCGCCCGTGCGGACCCTCTACCTGTCCAACATCACGGCCCCCAAGCTGGCCAAGCGTCCCACCGAGACCATCAGTGAG------------------ACCAAGGACGAGCCCTTTGCCTGGGAGGCCCGGGAGTTCCTGCGCAAGAAGCTGGTGGGCAAGGAGGTGATCTTCTTTGTGGAGTACAGCGTAAGC---------AACCGGGACTACGGCACTGTTTACCTGGGCAAGGACAGGAGCGGCGAGAATGTTGCAGAGTCCCTCGTGTCCGAGGGTCTGGTGGATGTTCGGCAAGGA------GGCAAGGGGGAGAGCCACCAGCGCCTCTGCGACCTCCAGGAGGTGGCGAAGTCGGCCGGGCGGGGCAAGCACGGCCCGGACGCG---AGCTCGCACGTGCGGGACGTCAAGTGGACCCTGCGGGACAACGAGGACCCCCGGACCTTCGTGGACCGACACGGCCGGAAGCCCATTCCAGCCGTGGTGGAGCACGTGCGGGACGGCTCCACCGTGCGGGTCATGCTGCTGCCGGATTTCCATTACATCACGCTCATGCTGTCCGGCATACGGTGCCCGTCGACGCGTCCGGACGAGTCCTCCTCGGGGGCGGAGTCTCCCCTGGTGGAGGAGGCCAAGTACTTCACGGAGAGCCGGCTGCTGCAGAGGGACGTGGAGGTGGTCCTGGAGGGGGCCACCAACCAGAACTTCACCGGCTCCGTGCTGCACCCCAATGGCAACATTGCAGAGGGGCTGCTGAAAAACGGCTTTGCCAAGTGCATTGACTGGAGTCTGACAACGGTCACTGGAGGTTCTGAGAAGCTCAAGGCTGCCGAAAAGGAGGCCAAGGAGAAGCGGCTGCGGCTGTGGAAGGACTACTCCGCCCCCACCGCGGGGCTAGGGGCG---GACGCCAAGTTCGAGGGCAAGGTGGTCGAGGTGATCAATGCGGACGCCCTCGTGGTGCGGCTC---GAAGACGGAGAGCTGCGCAAGATCTTCCTCTCGAGCATACGGCCGCCCAGGCGGTCGGAGGAGACGAAGGCGAGCGGT------GGGGAGTCCGGGGGCAAGGAGCGCAACTTCCGGCCCCTGTACGACATCCCCTTCATGTACGAGGCCCGGGAGTTCCTGCGCAAGAAGCTCATTGGCAAGAACGTCCAGGTGGGCGTGGACTACAAGCAGCCAGCCTCC------------------AACTCTTTCCCGGAGAAGACCTGCTGCACCGTCACCATCGGCGGCATCAACGTGGCGGAGGCCCTGGTGAGCAAGGGCCTGGCGACGGTGGTGCGCTACCGG---CAGGACGACGACCAGCGCTCGGCCCACTACAACGAGCTGCTGGCCGCGGAGATGAAGGCCCAGAAGTCCAGCAGGGGCCTGCACTCCAAGAAGGACGCCTCAGGACACAGGGTCGTCGACCTGTCCGGGGACCCGGCCAAGTCGAAGCAGTTCCTGCCCTTCCTCCAGCGGGCCGGCAAGATGGAGGCCGTGGTGGAGTTTGTGGCCAGCGGGTCCCGGCTGCGCCTCTACATCCCCCGCGAGAACTGCCTGGCCACTTTTCTGCTTGCCGGCATCTCGTGCCCGAAGGCCTCGCGA------CTCCAGGGTGGCCAGCAGGTGGAAGGGGAGCCCTTTGGCAACGAGGCCCTCGCCTACACCAAGGGACTCTGCCTGCAGAGGGAGGTGGAGGTTGAAGTGGACGCCATGGACAAGGCAGGCAACTTCATCGGATGGCTGACGGTGGAGGGGGTGAACCTGTCGGTGGCCCTGGTGCGGGAGGGCCTGGCTAGCGTGCACTTCACGGCGGAGCGCAGCGCCCACTTCCGGGCCCTGCAGCTGGCCGAGGAGCAGGCCAAGCAGCGTCGCGACCGCATCTGGGCCGGCTGGGAGGAGCCCTCC---GAGGAGGCCAAGCAGGCCGAGGTGGTCTCGGAGCGCAAGGTCACCTACAAGAACGTCTTGGTCACCGAGGTCAAGCCGGACCTCTCCTTCTACGTACAGTTCTTCGACGACGGACCGAAAGTGGAGGAGATGCTGACACTGCTGAGGCAGGAGCTGACGGAGCACCCCCCGATGCCGGGCGCCTACGCCCCCAAGAAGGGAGACCTGTGTGCAGCCAAGTTCAGCGAGGACGACCTCTGGTACAGGGCCAAGGTCGAGAAGGTCTCCAGTTCCGGCGAAGTGGACATCTTCTTCATTGACTACGGCAATCGGGACAAGACGGACGTGAGCCGGCTGGCGCCCCTGCCCTCGCTGGGCATCCGGGACTTGCCCCCGATGGCCCGGGAGTACTCGCTGGCCCTGGTGGCCCTGCCCAAGGACCCCGAGCAGGCCCAGGAGGCCCGCCAGGCCATGGTACAGCTGACGGCCGAGGCAGCCCTGCAGCTGAACGTGGAGTACCGGGTGGGGGGCCAGGACTTTGTGACCCTGCTGGTCAAGGAGGGCGGCCTGGACGTGGGCAAGAGCCTGCTCCAGGAGGGCTGGGTGCTGCTGGAGGAGCGCAGGGACCGCCACCTCCAGGACCTCGTCCGGGAGTATGCCCAAGCTCAGGACTCTGCCAAGGCTAAGCGGCTGAACCTCTGGTGCTACGGGGACGTGACAGAGGACGACTCGAAGGAGTTCGGA [2646]

Siro ATGTCGCAGCAAACGCAACCACAATCTCCTACT------------GTCCAAAGGGGCATCGGAAAATTAGTTTTGTCTGGTGATTCCGTGATCATTCG---CGGACAACCCAGGGGTGGCCCTCCCCCCGAAAGGCAACTTAATTTGTCTTGCATAATTGCTCCCAAATTAGCTCGTCGGCCGCCACAAGGCTCCGATACATCTGAT------------TCCAAAGATGAGCCTTTTGCTTGGGAAGCTCGCGAATTCTTACGAAAGAAAATAGTCGGTAAAGAGTTGTGCTTTGTGGTCGACTACAAAATTCCATCATCG---GGACGAGAATGTGGTTTTATCTATGTTGGAAAAGACACGACAGGAGAAAATGTAGCCGAATCTTTAGTAGCCGAAGGGTTGGTCGAAGTGAGGCAGACT---GGCAAATCCAACGAAATGCAACAGAAATTGTCGGAGCTTGCCGATTCGGCAAAGTCTGCCGGAAAGGGAATATACGCTCAGGATGCG---CAGAGTCACGTTCGAGATGTCAAATGG---------ACGATCGAAAATCCTCGTAACTTCGTGGATTCTTTGCATGCAAAGCCAACTAAAGCCGTCATAGAACACGTTCGTGACGGGAGCACGGTTCGAGCTATTCTGTTGCCAGAATATCACAACATCACGCTCATGATCTCGGGAATAAGGTGTCCTGCGGTAAAGATGGGTGACGAAATT---GGTGTAAGCGATCCTTTTGCTCTAGAGGCAAAGTATTTTACAGAAAGTCGGTTGCTTCAAAGAGAGGTTGAAGTCGTCTTGGAAAGCGTTTCCAATCAGAACTTCTTGGGTTCGATCATTCACCCGAATGGCAATATTGCGGAACACTTATTGAAAGAAGGCTTTGCAAAATGCATTGGTTGGAGTTTGAGTTTCGTCTCGGTGGGACCAGAAAAATTACGAGCTGCCGAAAAGCAAGCGAAAGAGAAAAAGCTGAGGATTTGGAAAGATTACGCTCCAAGCGGACCCGTTATTGACGCCAAAGATCGTGAATTTCTTGGAAAAGTTACGGAGATTATCAACGCAGACGCTTTGGTAGTCAAGCTT---AATGACGGAGCTCCCAAAAAAATCTTCTTGGCTAGCATTAGACCACCCAGACTGAGCGAAGATAAACAAGCCGAG---------ACGCAAAAAGACAACAAAGGACGCAACTTCCGACCCTTATACGAAATTCCGCACATGTATGAGGCTCGCGAATTTCTTCGAAAGAAACTGGTGGGAAAACGAGTCAACGTTTCCATTGATTACATTCAACCCGCTTCC------------------AATAACTTCCAAGAAAAAACGTGTTGTACGGTGACCATCGGTGGCATCAACGTGGCCGAAGCTCTCGTTTCTAAGGGACTCGCGAGCGTCGTCAGGTACCGC---CAAGACGACGACCAGCGTTCGACTCATTACGATGACTTGTTGGCTGCGGAAATGAAAGCGCAGAAATCGGGCAAAGGCATTCACAGCAAGAAAGATCCGCCAGTGCTTCGCGTCGTCGATTTATCGGGGGACCTTTCCAAATCCAAGCAGTTCTTTCCATTTTTACAACGTGCTGGGCGATCGGAAGCAGTCGTGGAGTTCGTCGCCAGCGGTTCCAGGATGAGGTTATTTGTGCCAAGGGAAAACTGTCAAATCACCTTCTTATTAGGGGGTATTAGTTGTCCGAGGGCTTCCAGG------CAAATCGGGGGTGGTGGAACGGAAGCAGAACCATTCGGTGACGAGGCGTTGCTATTCACCAAAGATCTGTGTTTGCAAAGAGAAGTTGAAATCGAAGTCGATTCGATGGATAAAGCTGGCAATTTCATTGGCTGGTTGTGGATTGAAAATAATAACCTCTCAGTGGCATTGGTTCAAGAAGGTTTGGCTTCGGTTCATTTTACGGCCGAACGCAGCGTCCATTTCCGTGCGCTACAGATCGCAGAGAACGACGCGAAACAGAGACAAGAAAAAATGTGGGCTGGTTACGAAGAACCGAAA------GAGCAAGAAGTAAAAGAAGAGAATTCGGAGAGGAAGGTTAATTACAAGTCCATCGTTGTGACGGAAGTCAGGCCGGATCTTCATTTTTATGCACAGCACACGGAAGATGGTAAACGGTTAGAAGAGCTGATGGAACAGTTACGTCAGGAGTTTACCAACAATCCACCTCTGGCCGGTTCGTACGCTCCCAGAAAAGGCGATCTTTGTGCTGCCAAATTTGTC---GACGATCAGTGGTATAGGGCAAAGGTCGAAAAGGTCTCCGGG---AACATCGTCAGCATCTTCTATGTCGATTATGGAAATAAACAAAATACCGAATCCATAAAGTTGGCAGCTCTTCCAACAGTTTTC---CAAGGTGCCGGACATGCAGCGCATGAATATGCACTTGCCTGTGTTTCTCTGCCTAACGACAATGACATTTTGGAGGAGGCGCGCCACATCTTTACACAAGACACCGATAATCAAGAGCTTCTTCTGAATGTCGAATATACCACGAGCGGAATTAAGTATGCGACTCTGGTGACGTCGGAGAACAAGGATGACATCATTAAAGGCTTGGTTAGCGAGGGCCTCTTATTGGTCGAAAAGAGGAAAGAGAAACGATTGCAGAAGATACTCGGTGATTACCAACATGCCGAAGAAGCGGCCAAACGAAAACGATTAAACATCTGGTGTTACGGAGACATCACCGAGGATGACGCAAAGGAGTTTGGA [2673]

Sitalc ATGGCAACAACACAGGCTCCCCCACCGCCTGTA------------TTACAGCGAGGAATAGTTAAACAGGTGCTATCCGGTGATTCTCTAATCATCCG---AGGACCTCCCCGCAATGGTCCCCCTCCAGAGAAGCAGGTAATTCTCTCTGGCATCACAGCTCCCAAGTTAGCCAAGAGAATCGTTTCGCCTAGCGATGGTTCAGGGGTTCAGGAA---ACGAAGGATGAGGCATACGCATGGGAAGCACGAGAATTCTTGCGCAAGAAATTGATTGGCAAAGAAGTGGCTTTTTCAGTAGAGCATAAAGGAGCGTCTGCGTCCACCAGGGACTACTATATCGTTTATCTTGGAAAAGATCAAACGGGAGAGAACATGACTGAATCCCTCGTTTCTGAGGGTCTGGTGGAGGTCAAGAGGCCA---AACAAGCCCACAGACTACCAACAGAGGTTAGCGTCACTGGAGGACCAGGCCAAGGAATCAAAGAAGGGCAAGTGGTCCATGGACACAGAGTCGGATCACATCAGAGATATGAAGTGG---------ACCATCGAGAATCCACGCAACTTTGTGGACGCATTACATCAAAAACCCACAAAGGCTATTGTGGAGCATGTGCGAGACGGAAGCACTGTCAGAGTTCTACTATTGCCTGACTTCTACAGCATAACACTCATGCTCTCTGGAATAAGATGCCCA------------GTCTACAGAAGTCCGGAAGATAAAGAAGACTACGGAGATGAAGCCAGATATTTCACGGAGACTCGTCTGTTGCAGCGCGAAGTGGAGGTCATCCTGGAAAGCGTTTCTAACCAGAACTTCATCGGCTCAGTCCTGCATCCGAATGGCAATATCGCCGAGCTCCTTCTGAAGGAAGGCTTTGCCCGTTGCATAGACTGGAGTATGGCATCCGTTTCTACAGGGTCAGAGAAGTTGAGAGCAGCTGAAAAAGATGCAAAGGCTCGCCACAGTCGTATCTGGAAGGATTATTCCCCGACGGGTCTGACCATCGAAGCCAAGGACAAAGAGTTCACTGGCAAAGTTATGGAGGTGGTCAACGCTGACGCTCTGGTACTCAAGATG---GGCGAGGGAGTTATCAAGAAGGTTTTCTTGGCCAGCATTAGGCCGCCTAGGCAGAGCGACGATAAACAGAACGAA---------------TCGAAGGAGAAGGGTCGCAACTTCCGTCCACTCTACGACATCCCGTACATGTACGAGGCCAGAGAATTCCTGCGCAAGAAGCTGATTGGCAAAAAGGTCAACGTGGTTGTGGACTACATTCAGCCAGCATCC------------------AACTCTTTTCCTGAAAAAACGTGCACCACTGTCACCATCGGTGGAATCAATGTTGCAGAGGCTCTGGTGAGCAAAGGGCTGGCAACGGTTGTGAGGTACCGT---CAAGACGACGATCAGCGGTCGGCACACTACGATGAACTCTTAGCGGCAGAAATGAAAGCCCTAAAGAACGCCAGTGGCATCCACAATAAGAAGGAAGCACCTATTCACAGGGTGGTCGATGTCTCTGGGGATCTGTCGAAAGCCAAACAGTTCCTACCGTTCCTGCAGCGTGCCAAAAAGATGGATGCTGTTGTTGATTTTGTGGCCAGTGGTTCCCGCCTGCGTCTCTTCATTCCACGCGAGAACTGCCAGATCGTCTTCTTACTAGGTGGCATTTCTTGCCCTAGAGCCGCTCGTCAG---ACACCGCAGGGCCCACCACAGCCGGGGGAGCCCTACGGGGAAGAAGCACTCACATTCACAAAGGAGCTCTGTTTACAACGAGAGGTTGAAATTGAGGTTGAGTCAATCGACAAAGTGGGCAACTTCATTGGCTGGTTGTGGGTGGAGGGCGTGAACCTGTCAGTGGCATTGGTCAAGGAGGGTCTTGCCAGCGTCCACTTCACGGCAGAACGCAGTCAGTACCTCAGGGATCTTCAGAACGCAGAGGACAACGCAAAGAGGAAAAGGGAAAAGATTTGGCTTGATTTCGAAGAATCCAAA------GGAACGGAAATAAAAGAGGAATCGACGGAACGCAAAGTCAACTACAAGACTGTCATGATTACAGAAATCAGACACGACCTGAACTTCTATGCACAAAATGTGGATGACGGTCCCAAACTGGATGAGCTAATGACGAACCTGCGCCAGGAGTTCCATACCAGCCCTCCCCTGGTGGGGTCCTACACCCCCAAAAAGAATGACCTCTGTGCAGCCAAATTCTCGGGGGACAAACTATGGTACAGGGCAAGGGTCGAGAAGATCGCTGGC---GACAAAGTACAAGTCTTTTTTGTCGATTTTGGCAATAGAGAACTGACCAAAGTGACCATGTTAGCCAGCCTTCCTCAAGCGTAT---AGTGGGTTATCCAACGCAGCACACGAATATGCTTTTGCCTATGTCGTGGCACCACAAGACAGTGACCTGTGCCAGGCAGCTGTAGAAAAGCTATACGAGGAAGCTGACAACACCCGTTTGCTGCTGAACATAGAGTACCGTACCGGTGGCATTCCATACGTCACCATAGTGACGGAAGAGGAAAAGGAAGACCTAGCCAAGGGTCTCATCCTGGACGGCCTTCTTCAGGTGGAGAAGAGGAAGGAGAAGTGCTTCCAGAAGATCATCACTGAATACAGGGAGGCTGAGGCACAGGCACGGAAGAAAAGGCTAAATCTTTGGCAGTACGGCGACATCACTGAAGACGATGCCAAGGAATTTGGT [2679]

Sclero ATGGCAACAACACAACCCCCCCAACCTCAACCTCCTACGGTA---CAGCACCGGGGAATTGTAAAACAGGTCCAGTCTGGTGACTCGCTGATCGTTCGTTCATCAGTTGTTCGTAACGGTCCTCCACCTGAGAAACAAGTAATCTTATCTGGGGTCTCGGCACCAAAACTTGCTCGAAGACCAATTCCTGGAAATGAAAATTCTGCAGCACAGGAAGCAACAAAGGATGAGCCTTACGCTTGGGAAGCTCGCGAGTTCCTACGAAAGAAGTTGATTGGAAAAGAAGTCACCTTCATTGTGGAAAACAAAGCCTCGGCAACTTCTTCGAGGGAGTACTACATTGTCTATCTGGGAAGAGACACGACTGGAGAGAACGTTGCGGAAGCTCTGGTCTCGGAGGGATTGGTAGAAGTGAAGAGGCCA---AATAAGCCTACTGATTACCAGCAGCGCCTGGCGTCTCTGGAAGATGCTGCGAAGGAAGCCAAAAAGGGCAAGTGGTCTTTGGAATCGGAGACTGATCACCAAAGGGATGTTAAGTGG---------ACCATCGAGAACCCTCGTAACTTTGTGGACTCTCTTCACCAGACGCCCACGAAAGCGGTGGTGGAGCACGTGCGGGATGGAAGTACATTCCGTGTCGTGCTTTTGCCAGACTACCATTACATCACGCTAATGCTCTCTGGAATCAGGTGCCCA------------GTGTATAAAACTGCCGCAGATTTTGAAACCTACGGTGATGAAGCCAGGTACTTCTCGGAGACCCGTTTGCTTCATCGGGACGTTGAGGTTGTCTTAGAAAGTGTCTCCAACCAGAACTTCATTGGATCAGTTCTGCATCCAAATGGAAATATTGCTGAAGCGCTACTGAAAGAAGGATTTGCACGCTGTGTGGATTGGAGTATGGCGTCAGTTTCCACGGGTTCTGAGAAGCTGCGGGCTGCTGAGAAGTTGGCTAAAGAGAAGAAAGTTAGAGTGTGGAAAGATTACTCGCCAACTGGCTTGAACATCGATGCCAAGGACAAGGAGTTCAGTGGGAAGGTCATGGAGGTGGTAAACGCCGATGCTCTTGTTATCAAGATGGCTTCCGATAATTCCCCCAAGAAAGTGTTTCTGGCCAGTATACGACCACCTAGGCAGAGCGAAGATAAACCAGAGCAA------------------AAAGACAAGGGTGCCAAATTCCGTCCGCTCTACGACATTCCATACATGTACGAGGCTCGAGAGTTCCTACGCAAAAAGTTGATCGGCAAGAAAGTTAACGTAGTGATCGATTACATTCAACCCCCTTCC------------------AACTCTTTTGCAGAGAAGACTTGCTGCACCGTTACCATCGGAGGAATCAACGTTGCAGAGGCTTTGGTCTCTAAAGGACTGGCCACGGTTGTGAGATATCGT---CAAGACGATGACCAACGCTCGGCTCATTACGATGAACTTCTGGCAGCCGAAATGAAAGCTCAGAAAAACGGCAGCGGCATTCATAACAAAAAGGAAGCCCCTGTTCACAGGGTGGTGGAGATTGCTGGGGATGCAGCAAGGTCCAAACAGTTCCTACCCTTTCTACAACGTGCCGGCAAAATGGAGGCTGTCGTAGACTTTGTGGCCAGCGGCTCCCGCCTGAGGCTATTCATCCCTCGCGAAAATTGCCAAATCACATTCCTCCTGGGAGGCATCAGCTGCCCCCGTGCAGGCAGGCAA---GCGCCTGCAGGGGTCGGGCAGCCGGCGGAGCCCTGGGGAGATGACGCGCTCGCCTACACCAAAGACCTTTGCTTACAGAGGGAGGTGGAAATTGAGGTTGAGGCAATGGACAAAGCAGGCAACTTCATTGGTTGGCTGTGGGTGGAAGGGGTCAACCTGTCGGTGGCCTTGGTCAGGGAAGGTCTTTCCAGAGTGCACTTCACGGCGGACAGAAGTCCACACCTCAGGGATCTCCAGAACGCAGAGGACAACGCAAAGAGGAAAAGAGAAAAGATTTGGATCGATTTTGAAGAAACTAAA------AATGTTGAGATTAAAGAAGAAACAACAGAACGCAAAATCAACTACAAGACAGTTATCATCACAGACATTAAACCAGACTTGAACTTCTACGCACAAATTGTTGATGAGGGACCTAAGCTCGAAGAACTGATGAGCAACCTGAGGCAAGAATTTAGTACCAATCCTCCTACAGTAGGTGCTTACACGCCTAAAAAGAATGACATCTGTGCGGCCAAGTATTCCGATGACAATCAGTGGTACAGGGCCCGAGTTGAGAAGATTGCAGGA---AATAAAATACAAGTCTGCTTTGTTGATTATGGAAACAGAGAACAGACAACATCAACTATGCTGGCCACATTGCCTTCTACATAC---AGCAGTTTACCACACGCTGCCCACGAATATGCATTTGCCTATGTGGTCATGCCGAAAGACACGGACTTGAGACAAGCGGCGATCGACAAGCTGTATGGGGAAGCTGACAACTCCCACCTGCTATTGAATGTGGAATACCGGACGAACGGAATCCCACACGTGACGCTCGTGACACAAGAGGAGAAGGAAGACCTGGTCAAGGGCCTGATCACGGACGGCCTCTTGCAAGTGGAGAAACTCAAGGACAAATGCTACCAGAAGATCCTTACAGAATACAAGGAAGCTGAAACGCAGGCTAGGAAGACGAGGCTAAACCTGTGGCAGTATGGTGACATCACAGATGACGATGCCAAGGAGTTTGGC [2694]

Trogulus ATGGCAGAACCAGATCATCCCAATCAACCC---------------ACCTTAAAGGCATTCGTTAAACAGATTGTGTCCGGCGATTCTATTATTGTCAT---GGGTAAACCCAAGGGTGGACCTCCGCAAGAGAATCAAGTGAATTTGGCTTATATCATCGCACCAAAAATGGGACGACGAGCTAGTCCAAATCAAGAAAACTCACAGGAT---------ATAAAGGATGAACCCTATGCTTGGGAGGCCAGAGAATTTCTAAGAAAAAAACTTATTGGAAAGGAAGTATACTGCGCAGGGGAGTATAAAGTGGCTACGAAT---GATCGGATGTTTGCTATTGTTTATCTTGGCAAAGATGCTACTGGTGAAAACATCAATGAAGCTGTAATTACCGAGGGACTAGCTGAAGTAAAAAAATCT---CAGTCATCTAACAGCATGCAGCAGAAACTGTCAGTTTTAGAAGAACAGGCGAAATCAGCAGAAAAAGGCAAATGGAGCAGCGACCCACCAAGCTCTCATGTACGAAATGTGAAAAGC---------ACGGTAGAGAACCCAAGGAATTTTGTCGATTCCTTTCATCAGAAGCCAATTCCAGCTATTATCGAGCATGTCAGAGATGGAAGCACAGTTAGAGTTATGCTATTGCCAGACTTTTACAGTGTCATGGTCATGATGTCTGGAATTAGGTGTCCC------------ATTATCAAAGAT---GGTGTTGCAGAACCATATGCCCTGGAAGCAAAATATTACACTGAATGTCGTTTACTACATCGAGATGTTTCAGTTGTGCTTGAAAGTGTATCTAATCAAAGTTACTTAGGGACTATATTACATCCGAATGGCAATATTTCTGAGCTTTTAGTCAAGGAAGGATTTGCTCGGTGCGTTGATTGGAGCATTTCATTCGTTTCTACGGGACCTGAAAAACTTAGAGCTGCAGAAAGGCACGCAAAAGAAAAACGACTTCGTCTTTGGAAAGATTACACTCCCACAGGACCCGTCATTGATGCCAAGGAAAAAGAGTTTACAGGCAAAGTAACAGAAGTGATGAATGCTGATGCACTAGGAATCAAATTG---GCAGATGGGACAATCAAAAAAATTTTCCTCTCTAGTGTACGGCCACCAAGAAAACCTGAGGGCAATGCTGAGCAAGATAAACCGGAGAATGCAAAGGAAAAAAATCGTAATTTCCGTCAACTTTATGATGTTCCTTACATGTTTGAAGCGAGAGAGTTTCTCAGGAAAAAGCTAATAGGCAAGAAGGTCAATGTAACAGTCGACTATATTCAGCCTGCCACA---------------------ACTCTGCCCGAAAAAGTTTGCTGTACTGTCACAATTGGAGGAATCAATGTTGCAGAAACATTAGTCAGTAAAGGTCTTGCTACTGTGGTTCGGTATAAA---CAAGAGGATGACCGTCGTTCTACACATTATGACGAGTTGCTTGCAGCAGAGATGAAAGCGCAAAAAAGTGGAAAGGGCCTTCACAGTCGTAAAGAAACACCTATGCACAGAGTTGTTGACCTAGCTGGGGATCCTGGTAGGTCAAAGCAATTTTTCCCATTTCTTGAACGTGCTGGCAAAATGGAAGCAGTAGTAGAATTTGTGGCTAGTGGTTCCAGACTCCGGCTTTATATCAGCAGAGAAAATTGCCTTATTACATTCTTGCTGGCAGGCATTAGTTGTCCAAGAGCTTCTAGACAAGCCACAGCAGGTGGAGCTGCCATGGCTGGTGAGCCTTGGGGTGATGAAGCATTGTCTTATACCAAAGACCTCTGTTTGCAAAGAGAGGTTGAAATACAAGTTGAGTCAATGGATAAAGCAGGAAATTTCATTGGCTGGCTTTGGATTGATGGGCTGAATCTCTCGGTTGCTTTGGTAGCAGAGGCTCTCTCTAAGGTCCATTTTACAGGAGATAGGAGTTCATTTGCGCGAGACCTACAAATTAAGCAGACTCAAGCCAAGGACAAGAGAGAAAAGATATGGTCTGATTATGAAGAACCGAAGAGCATTGATGAGATAACGGAAGACATCAACAGAGAAAGAAAGATTAATTACAGGAAGGGATTCATTACAGAAATTAAGCCAGACCTAAATTTTTACATTCAATATGAAGAAGATGGTCCTAAAGTCGAAGAGCTAATGAATCAACTAAGGCAAGAACTGGTAGCAAGTCCACCTCTTCCTGGCTCTTACACCCCCAAAAGAGGTGAACTTTGTGCAGCACTTTTTAGC---GATGGTCTGTGGTACAGGGCAAAAATTGAAAGGGTCACAGGA---AACAGGGTTCAGATTCTCTACGTTGATTATGGAAATAGAGAAGAGACTGATTCAACAAAATTAGCATCATTGCCAACACTATAT---CATGGTGTTCCACCAGCTGCGCATCAAACAGCAATGGCCTGTATAAAATTGCCTACCGATGTGGAAATTTTGGCTGATGCTCGCAATGGCTTTCATCATGACACAAACAACCGCCCAATTAAAGTCAATGTGGAGTATCGTAAGGAGGGAATAGATTTTGTAACAATGATGACG---GAAGACAACGAAGACATTGTTAAGGGACTTATTGCAGAAGGACTTCTGCTAATCGAGAGCCGACAAGAAAAGAGATTTGAAAAGTTCCTCAAAGATTATTCCATTGCCATGGAGGCTGCCAAAAAGGAAAGGTTGAACCTCTGGCAGTATGGAGACTTCACTGATGATGATGCCAAGGAGTTTGGT [2679]

Ortho ATGGCACAACCTAATCAGCCTCAGCAAAATCAGCTCAAG------ACCTTAAATGGCGTTGTCAAACAGGTTTTATCAGGCGATTCAATTGTCATCCG---AGGGCCTTCTAAAGGAGGAGCTCCACCAGAGAAGCAAATCAATTTGGCTTATGTAATAGCACCCAAAATGGGACGACGGGCCAATCAAACCCAGGAA---------GAT---------GTTAAAGATGAGCCTTATGCATGGGAATCTAGAGAATTCCTAAGGAAAGAGGTCATTGGAAAAGACGTTCACTGTGTATGTGAATACACCGTGTCA---------GATAGATGGTTTGCAGTTGTACATCTTGGAAGAGATAGTAATGGTCAAAACATGAATGAAGCTGTGATTGCCGAAGGATTGGCAGAAGTAAAAAAATCA---CAGAGTGCTAGTAGCTTACAGCAGAAGCTATCTCTCCTAGAAGATCATGCAAAATCATCTGGAAAGGGAAAATGGGGCCCTAACCCACCAAGTGCCCATGTGCGAAATGTGACCAGC---------ACCATAGAGAACCCCAAAAATTTTGTTGACTCCTTCCATCAGAAGAAAATACCTGCCATTATTGAGTATGTTAAGGATGGGAGCACAGTTAGGGTCACATTGCTTCCAGATTTTTACAGTATCATGATCATGATGTCTGGAATAAGGTGTCCG------------GGTTTTAAAGAT---AACATACCAGAACCATATGCTGCAGAAGCAAAGTATCATACTGAATGTCGTTTGTTACACCAAGAAGTTGAAGTTTGTCTTGAGAGTGTTTCTGGCCTAAATTTCCTTGGAACTATTTTTCATCCTAAAGGCAACATCGCTGAGTTCTTAGTTCGAGAAGGATACGCCCGCTGCGTTGACTGGAGTATTCCTTTTGTAACTAGTGGCCCTGAGAAACTAAGAGCGGCAGAAAAGCATGCTAAAGAAAAACGCCTTAGACTGTGGAAAGAATATACTCCATCTGCGATCACAATTGATGAAAAAGACAAAACCTTCGAAGGAAAGGTTGTGGAAGTTATAAATGCGGATGCCCTTGGAATTAAATTA---GCTGATGGATCCATTAAGAAGATTTTTTTGTCTAGTGTTCGACCCCCCAGGCGGCCAGAAACAAACCCTGATCAA---------GAAAACCAAAAAGAGAAGAGCCGAAATTTCAGACAACTTTACGACATTCCCTACATGTTTGAAGCCCGAGAATTCCTGAGGAAGAAACTGATAGGAAGGAAAGTTCAAGTATCGGTTGATTATATCCAGCCAGCCACAGCCAGTCAGCAACCGGCCATGACCTTGCCAGAGAAGACATGCTGTACTGTTATTTTCTCAAATGTGAATATAGCAGAAAGTTTAATAGGTAAAGGTCTGGCCACAGTGGTTCGCTACAAG---CAAGAAGATGACCGACGTTCGGCAAGTTTTGATAACCTTCTAGCTGCTGAGACCAAAGCACAGAAGGATGAAAAAGGGTTGCATAGTCGAAAAGAAACTTCCATACACAGGGTCGTCGATTTAGCTGGGGATCCTGGAAAATCTAAACAGTTCTTTCCGTCCTTGGAGAGAGCAGGCAAAATGGAAGCTCTAGTGGAATTTGTTGCTAGTGGATCCCGACTCAGGCTGTATATCAGCAGACATAATTGCCTTATCACATTTTTACTTGCTGGCATCAGTTGTCCAAGAGCTTCTAGACAAGCAATGGGAAGTGGAGTTGCTACACCGGGTGAACCCTGGGGTGACGAAGCACTGGCCTTCACGAAAGCCCTATGCCTTCAGAGAGAGGTTGAAATTAAAGTAGAAGCAACAGATAAAGCCGGAAATTTCATTGGCTGGCTTTGGATCGATGGTGCCAACCTCTCAATTACTTTGGTCGCAGAAGGCCTCTCCAGGGTCCATTTCACTGGAGAAAAGAGTGAGCATGGCAAGGATCTTCAAGCCAAGCAGAAAATTGTACGGGATAGAAGAGAAAAGATTTGGTCAGATTACGAAGAACCTAAAGACAGTGATGAGATTAAGGAAGAAATCAACAGGGAAAGGAAAGTCAACTACAAGAAAGGCTTCATCACAGAAATTAAACCAGACCTCAATTTTTATATTCAATATGAGGAAGATGGTCCCAAAGTAGAAGAACTGATGAATAAACTAAGGCAAGAGTTAGTGGAGACTCCTCCTTTACCGGGATCCTATGTGCCTAGAAGAGGGGACCTGTGTGCAGCGCTTTTCAGT---GATGGCCTCTGGTACAGGGCGAAAGTTCAGAAGGTCACAGGG---AACTCTGTTAGCATACTCTATCTTGATTATGGAAATCGGGAAGAAACTGATGCAACCAAATTAGCTGTATTGCCATCATCTTAC---CATGGA---CCTCCAGCAGCACATGAGACAGCGTTGGCCTGTATAAAGCCCCCACCAGATGTGGAAATCCTGGCAGAAGCCCGCAACAGCTTTCACCATGACACAAATAACAGACCAGTTAAAGCGAATGTTGAGTATCGCAAAGAGGGTGTGGATTATGTCACACTTATGACA---GAGGACAATGAGGATATTGTCAAAGGACTCATTTCGGATGGACTTCTCATGATCGAAGACAGAAGAGAAAAGAGATATGAGAAATTCCTTAAGGATTATACAGCTGCCATGGAAACTGCCAAAAAAGAAAGGTTAAATTTATGGCAATATGGTGACTTCACGGATGATGATGCCAAGGAGTTTGGT [2682]

Hespero ATGGCTCAGCCTTCTCAACCTCAGCAAAACCAACCGCAATATGCGCCAATTAGAGGCATTGTAAAACAGGTGTCATCAGGCGACACTGTTATTATCCG---AGGTCAGCCCAAAGGTGGTCCTCCTCCTGAGAAAACGTTAAGCTTAGCTTACGTTATCGCTCCTAAATTGGGTCGTCGTCCACCCCCCAATGCGGAGAATCAGGCCGAA---------ATCAAAGACGAACCTTATTCGTGGGAAGCTAGAGAATTTCTTAGGAAAAAACTCATTGGCAAAGAGGTTTATTTTTTGATCGAATACAAAGTGCCTTCAGCTACAAACAGAGAGTTCGGAGTGCTGTATCTGGGGAAAGACGTTAACGGAGAGAATATGAACGAAGCGATCGTCTCCGAAGGATTGGTAGAAATCAGAAAAACTGGACAGAAGCAAAGCGATGTTCAATCAAAGCTGTGCGAATTAGAAGAGACGGCTCGTTCGGCCGGCAAGGGCAAATGGATTTCCGACTCTCCCAGTTCTCACATCCGAGAAGTTAAGCAC---------ACGGTTGAGAATCTGAGGAATTTCGTCGATTCGTTTCACAAGAAGCCTGTCAAAGCGATCATCGAGCACGTCAGAGATGGCAGCACCGTCCGTTGCACTCTCTTACCTGATTATTACAGCGTTATCATCGTCATGTCGGGAATTAGATGTCCG------------ATATCGAAAGAC---GGAGTACCCGAAACTCATTTTGAGGAAGCCAAATTTTACACTGAATCGCGATTACTGCACAGAGACATTGAGATTGTCTTGGAAAGTGTTTCCAATAACAGCTTGATAGGCTCCATTTTACATCCCAACGGTAGTATTGCGGAATTGCTGCTTAAAGAAGGATTAGCAAAATGTGTTGATTGGAGTATTACATTTGTTACGAACGGTGCCGAAAAGCTTAGAGCGGCTGAGAAGATTGCAAAAGAGAAACGTTTGCGCATCTGGAAAGATTACAGTCCGGCCGGAGTCGTAATCCAAGCCAAGGACAAAGAATTCCAAGGCAAAGTGACGGAGGTTATGAATGCGGACGCATTAGGCATAAAATCG---GTGAACGGGCAAATCAGAAAAATCTTTTTGTCGAGTATTCGCCCACCGAGATTGTCGGAGGAGAAACCA---ACCGAGGGAGGAGTGCCCAAAGAAGCGAAGAAATCGAATTTCCGACCCCTGTACGACATTCCTTACATGTACGAGGCCAGAGAGTTTTTGAGGAAGAAATTAATTGGGAAAAGAGTCAACGTAGTCGTAGATTACATTCAACCCGCTTCGACC------------GTCAATAATTATCCAGAGAAAATTTGTTGTACCGTCACAATTGGAGGAATCAACGTAGCTGAGACTCTTGTGGGCAAAGGCTTAGCAACTGTGGTCCGATATCGT---CAAGATGACGATCAGCGTTCAATGCATTACGACGAATTATTAGCCGCCGAAATGAAAGCTCAAAAAAGTGGGAAAGGATTACACGGCAAAAAAGATACACCCCTTCATAGAGTTGTAGATTTATCAGGGGATCAATCTAAATCTAAACAATTTCTGCCTTTCTTGCAACGAGCCGGCAAAGTCGAAGCCATCGTAGAGTTTATAGCCAGTGGTTCTCGACTTAGGTTATACATTCCAAGAGAGAATTGTCTTATCACTTTTCTATTGGGAGGTATTAGTTGCCCCAGAGCCAGCAGA------ACACAAGGCACTGCACCCATTCAAGGAGAACCGTGGGGAGATGAGGCAATGGTGTATACAAAAGATTTATGCTTGCAGAGAGAGGTCGAAATCGAAGTTGAATCTATGGACAAAGCTGGCAATTTCATCGGTTGGTTATGGGTAGAAGAGAAAAATCTCTCGGTGGAATTAGTGGGAGAAAGTTTGGCCAAAGTTCACTTCACAGCGGAGAGAAGTCCTTACGCTAGGGACTTGCAAATCAAGGAACATCAAGCCAAAACAAGAAGAGACAAAATTTGGTCCGATTTCGAAGAGCCGAAAAATGAAGACGAAATCAAAGAAGACATTGACAAAGAACGAAAGGTCAACTACAGAAAAGGTTTCATCACGGAGATTAAACCCGACCTTCATTTCTACATCCAATACGAAGAGGACGGACCCAAAGTGGAAGAATTGTTGACACAATTGAGACAAGAGCTTACGTCAAACCCCCCATTGCCCGGTTCTTACGTCCCCAAGAGGGGTGACACGTGTGCCGCTCTCTTCACC---GACAATTTGTGGTACAGAGCCAAAGTGGAGAAAATTGCCGGG---ACGACGATTAATATACTTTACATCGATTACGGAAACAGAGAAGTGACAGATTCGACAAAGTTAGCTCTCTTGCCAGGCTCGTTT---AAAGATGTTACTCCCGCTGCTCATGAACTTGCATTTGCATGCATCAAATTGCCTTCAGACGTTGAAGTTTTGGAAGAAGCTAGGCACGTGTTTCATCACGACGCGGATAATCGTCTTCTCAAAGTGAATGTAGAATACAGAGCGGCCGGTGTTGATTACGTAACGTTAATGACG---GAAGACAACGAAGACGTCGTCAAAGGATTATTGGGAGAAGGGCTCTTATTGGTCGAAAGCAGAAGGGAGAAACGATTTCAGAAATTCCTCAAGGAATACGAATTGGCAGAAGATGCGGCCAGAAAGAAAAGGTTAAATCTTTGGCAATACGGAGACTTTACTGAAGATGACGCAAAAGAATTTGGT [2700]

Leiobunum ATGGCTCAGCCACAACCGCCACTTAAA------------------CCCGTCAAAGGGATTGTGAAACAGGTGCTTTCAGGTGATTCTCTTGTCATTCG---AGATATTCCCAGAGGAGCTAGACCTGCCGAGTTCACAGTTAACTTGGCATATCTCATTGCTCCAAAAATTGCTAAAAAA---------------------------GAT---------GGACATGATGAACCGTATGCTTGGGAAGCCAGAGAATTTTTGAGAGAAAAAGTTATTGGGAAAGAAGTGTTCTTGGCTGTTGAGTATCAAATTCCAGCAACT---AAGCGAGATTGTGGAATTCTTTTTCTTGGAAGAGATGAATCTGGTGAAAATGTCTCTGAAGCACTGGTAGTAGAAGGACTAGCTGAAGTTAGGAGTTCGGGACAAAAGCTAAGCGAGGCCCAACAAAAGTTAAAGGACTTGGAGGCTATAGCAAAAACTGCTGCAAAAGGAAAATGGAGTAGTGACCTCCCTCAGCTTCATGTACGAGACATAAAGAAT---------GGAATAGAAAACCCAACAAATTTTGTAGATTCTTTAAAAAAACAGCCACAAAAAGCAATTGTAGAGCATGTACGTGATGGAAGTACCGTCAGAGTTTTATTGCTTCCTGATTTCTATAATGTTTTGGTACTTATTGCTGGAATTAAGTGTCCT------------TCCTGTGGGCCA---AATTCACCTGAAGAAGGTGGTGAAGAAGCAAAATATTTTACTGAGTCTAGACTCCTACAACGACAGGTTGAAGTGACATTGCTGGGGGTTAGCAATCAGAACTTTATAGGAACCGTGCAACATCCAAATGGAAACATTGCTGAATTTTTATTGAGCCAAGGATTTGCCCAGTGCTCAGATTACAGTTTAAAAATGATTCCTGAAGGAAGAGAGAAATTAAGAGCGGCAGAAAAATTGGCCAGAGACAAGAGACTACGAATTTGGAAAAACTACACGCCTTCTGGTGTTACCATAGAAGCTAAAGATAAAGAGTTTGCTGGGAAGGTGGTAGAAGTGGTTAATGCAGATGCTTTAGTGATTAAAACT---CCATCCGGTCAGATGAAGAAAATATTTTTATCCAGTGTAAGACCTCCCAGGCTTGAAGAAAACAAATCTTCAGAA---------ACTCCAAATGATCCCAAAGGAAGAAATTTTCGGCCTCTCTATGACATTCCATTTATGTATGAAGCAAGGGAGTTTCTAAGAAAGAAGCTTATTGGAAAAAAAGTCAGTGTTTCAGTAGATTATATTCAGCCAGCATCA------------------AACTCATTTCCTGAAAAAACATGCTGCACTGTTACTATTGGAGGAATTAATGTTGCTGAAACTCTTATTGGAAAAGGATTGGCAATGGTGGTACGTCACAAATCTGGAGATGTTGACCAAAGGTCATCTCATTATGATGAACTTATGGCTGCTGAAATGAAAGCCCAAAAGAGTGGAAAGGGTATGCACGGAAAAAAAGACAATGCTGTGCACAGAGTTGTGGATCTATCTGGGGATCTTTCGAAGTCAAAAGCATTTTTTCCATTTCTTGAACGAGCTGGCAAGATGGAAGCTGTGGTTGAATTTGTTGCTAGCGGTTCTAGACTTCGACTTTACATTCCCAAAGAAAATAAACTAATAACATTTTTACTTGGAGGTATATCTTGCCCTAGAGCAGCAAGACCA---GGACCAAATGGTACTAGAATAGAAGGTGAGCCATGCGGAGAGGAAGCTTTATTGTTCACTAAAGAGTTATGCTTGCAAAGAGAAGTTGAAATACAAGTGGAAGCTATGGACAAGGCTGGAAACTTTATTGGATGGTTGTGGGTTGAAGGAGTCAATCTATCTATTGCCTTAGTTGCTGAAGGCTTAGCCAGTGTTCACTTTACAGCAGAAAGATCACCATATGCCAGTGAACTTAGAATTAAACAAGACAGTGCTAAATCAAGAAAAGAGAAGATTTGGCTCAATTATGTTGAACCAAAA---GCAGATGAATTAATTGAAAATGATGATGTTGACAGAAATGTTAATTATAAGAAAGTGGTAATCACCGAAATCAAGCCAGATTTAAACTTCTATGCACAACATGAGGAAGATGGTCCTAGACTTGAAGAAATGCAATTACAAATGAGGCAAGAACTGACATCAAACCCTCCCTTATCTGGTGCCTATTCACCCAGAAAAGGTGACCTTTGTGCAGCAAAGTTTAGTGATGATGGTCTCTGGTACAGAGTTAAAGTAGAGAAAGTTACTGGA---AGTACAGTACAAGTATTCTATGTTGATTTTGGAAATAGAGAAGAAACTCAGTCAACAGTTTTAGCTGTATTACCTACACTGTAT---CATGGTGTTCCTCATGGTGCCCACGAATATGCATTTGCTTGTATTCAACTTCCAAAGGATCCTGAAATTATTGAAGAAGCTCGATATGTCTTCCACCATGACACCAATAACCATTCTCTTAAACTGAATGTTGAATATAAATTGGGTGGAGTAGATTACGTAACACTTGTTACA---GAAGATGGTGATGACATCGTAAGAGGTCTTATAAATGAGGGTCTGCTATTAGTAGAGAAGAAAAGGGAAAGAAGGTTCCAAAAATTTCTTAAAGAGTACATAAATGCAGAAGATGAAGCCAGAAAAAAGAGGCTAAACATCTGGCAATATGGTGATATTACAGAAGATGAAGCTAAAGAGTTTGGG [2646]

Proto ATGGCTCAGCCACTACCAGCGCTTAAG------------------CCCGTCAAGGGGATTGTTAAACAGGTGCTTTCAGGTGATTCCCTTGTCATTCG---TGATATTCCCAGGGGAGCTAGGCCTGCAGAGTTCACAGTTAACTTAGCATATCTCATTGCTCCAAAAATTGCTAAAAAG---------------------------GAT---------GGCAATGATGAACCTTATGCATGGGAAGCCCGAGAATTTTTGAGGGAAAAGGTTATTGGGAAAGAAGTTTTCTTGGCAGTTGAATACCAAATTCCAGCCACA---AAAAGAGATTGTGGCATTCTTTTTCTTGGAAGAGATGAATCAGCTGAGAATGTTTCTGAAGGAATGGTTGCAGAAGGACTTGCCGAAGTTAGAAGTGGT---CAAAAACTAAGCGAGGCACAACAAAAGTTAAAAGACTTAGAAGCTATTGCAAAGACTGCTGGGAAAGGGAAATGGAGCAGTGATCTTCCTCAACTTCATGTGCGAGACGTAAAGAAC---------GGAATTGAAAATCCAACCAATTTTGTTGATTCTTTAAAAAAACAACCACAAAAAGCCATTGTAGAGCATGTACGTGATGGAAGTACTGTCAGAGTCTTGTTGCTCCCCGATTTCTACAATGTTTTGGTACTTATTGCAGGAATTAAGTGTCCT------------TCCTGTGGGCCA---AATTCACCTGAAGAAGGAGGCGAAGAAGCAAAATATTTTACTGAGTCTAGACTTTTACAACGACAGGTTGAAGTGACATTGCTTGGCGTTAGCAATCAAAATTTTATTGGAACTGTGCAACACCCGAATGGAAATATTGCAGAATTTCTTCTGAGCCAAGGATTTGCGCAATGCTCAGATTACAGTTTAAAAATGATTCCTGAAGGAAGGGAAAAGCTGAGAGCAGCTGAAAAAATGGCCAGAGACAAAAGGCTACGAATCTGGAAAAACTACACGCCTTCTGGTGTAACCATAGAGGCTAAAGATAAAGAATTTGCAGGGAAAGTTGTGGAGGTTGTTAATGCAGATGCTTTAGTAGTTAAAACA---CCATCCGGTCAGATGAAAAAGATATTTTTGTCCAGTGTCAGACCTCCCAGGCTTGAAGAAAATAAATCTTCTGAA---------ACTCCAAATGATCCTAAAGGAAGAAATTTTCGGCCTCTCTATGACATTCCATTTATGTATGAAGCCAGGGAGTTTTTAAGAAAAAAGCTAATTGGGAAAAAAGTCAGTGTCTCTGTCGACTATATCCAACCAGCTTCT------------------AACTCATTTCCCGAAAAAACATGCTGCACTGTATCGATTGGAGGAATTAATGTTGCCGAAACACTTATTGGAAAAGGATTGGCAATGGTAATCCGCCATAAATCTGGAGACGTTGATCAGAGATCATCTCATTATGATGAACTTATGGCTGCTGAAATGAAAGCCCAAAAAAGTGGAAAAGGAATGCATGGAAAGAAAGATAATGCTGTGCACAGAGTTGTGGATTTATCTGGGGATCTTTCAAAGTCAAAAGCGTTTTTCCCTTTTCTTGAAAGAGCTGGCAAAATGGAAGCTGTAGTTGAATTTGTTGCTAGTGGTTCAAGACTTCGACTTTACATTCCCAAAGAAAATAAACTAATTACATTTTTACTTGGAGGAATATCTTGTCCAAGAGCAACACGGCCA---GGACCAAATAGTACTAGAATGGAAGGAGAGCCATACGGTGAGGAAGCTCTGTTATTCACTAAAGAATTATGCTTGCAACGAGAAGTTGAAATTCAAGTGGAAGCTATGGATAAGGCAGGAAACTTCATTGGTTGGCTCTGGGTTGAAGGAGTTAACCTATCAATTGCTTTGGTTGCTGAAGGCTTGGCCTCTGTTCACTTCACAGCAGAGAGATCGCCATACGCTAGTGAACTTCGAATTAAACAAGACAGTGCAAAATCGAGAAAAGATAAGATTTGGCTCAATTATGTTGAACCCAAA---GCTGATGAATTTATTGAAAATGAAGATGCTGACAGAAACGTTAATTATAAAAAAGTGGTAATCACTGAAATTAAGCCTGATTTAAACTTCTATGCACAACATGAGGAAGATGGTCCTAGACTTGAAGAAATGCAATTACAAATGAGACAAGAATTGACATCTAATCCTCCTTTATCTGGTGCCTATACGCCAAGAAAAGGTGATCTTTGCGCAGCAAAGTTCAGTGATGATGGTCTCTGGTACAGAGTTAAAATAGAGAAAGTAACTGGA---AGTACAGTACAAGTATTCTATGTTGATTTTGGAAATAGGGAAGAAACATCATCAACTGTTTTAGCTGCGTTACCAACGTTATAT---CATGGTGTTCCTCAAGGCGCCCATGAATATGCATTTGCTTGTATTCATCTTCCCAAGGATCCTGAAATTATTGAAGAAGCTCGATATGTTTTCCACCATGACACCAATAATCAATCTCTTAAACTCAATGTTGAGTACAAGCTGAGTGGCACAGATTACGTGACTCTTCTCACA---GAAGACGGAGATGATATTGTGAAAGGGCTTATTAATGAAGGTTTGCTACTGGTGGAGAAGAAAAGAGAAAGAAGGTTCCAAAAATTCCTTAAAGAATACATTAATGCTGAAGATGAGGCGAGAAAAAAGAGGTTAAACGTTTGGCAGTACGGTGATATTACTGAAGATGAAGCTAAAGAGTTTGGG [2643]

[TICKFAST_NCHAR=1062;60S ribosomal protein L3; part 10; SCSE]

Ixodes CAATGTCGCTACAAG---ACCACCACGCCCAAGCGCCGAAAGACGTACCCGTTCCACTGGTGGGCACCGCGCAAACGAAAGCACTTCGGAGAGGACCACCTCCTGAAGGAAAACATCGAGTTCCTCAAAGAAGTCGCCCAAAAGATGTACCTTCAGCCG---GGCGAGAGTCCTCTCAGGAATGAACCCTGGGAGAAAGGCGTGTGGACGCCGGACTCTGTTCGGACCGGCGTCATCGCCCGCAAGATCGGCATATACCCGATGTGGACCACCTCGGGTAAGCGAATCCTCACCACCCTACTGCAGGTGCTGGACAATCACGTGATCCGCTACATCCCGTCGGAAGAGTACGCCTCATCCAGGTTC------GGCTTCAAGGCGAAGGGCCGCGGCTGCCTCGTCGTGGGCGCCGGCAGCGCGGACCCGATGCTCTTCAAGGCCCCGTACCTCAGACTGTTCCAGGAGTCCGGCGTCATGCCCAAGCGCAAGCTGACGCGGTTCCTGATTACCGACGACGCCGCTATTCAACCCGGCACGCCTCTGTCGGCCGGGCACTTCCGAGCGGGGGACTACGTGAACGTGTACGGCAAGACACGGGGGCACGGCTTCCAGGGGGTCATGAAGCGCTGGAAGATGAAGGGCGGCCCGAAGTCCCACGGCACTACCAAAGCGCACCGGCGGCTCGGGGCCATCGGAGGC---------CCCCGGGGCATCATCCTCAAGGGAAAGCGGATGCCCGGACATATGGGCCAGGAGCGGAGGCTCTTGGTGGGTCTCAAGATCTGGAGGATCAACACAAAGCTGAATGTGCTTTTCGTGCAAGGTCCCGCCGTTCCGGGCGCCACCCTATCCTACGTGAACATCTACGACTCGCGGCACGGCAAGAAAAAGCACACAGAAGACAACCCGCCACCGTTCCCGACATACTACCCAGACCCGGAGAACCCG---CTGCCCGAGGAGATGTATGACAAAGAACTGCATCAGTTCGAGGCTCCCTCCGTTACCTTCGTTGACGAGGAGGTGGTTGACAAGAAGAAGGCCAAGGTGAAA [1038]

Siro TACGTTCCCAAATAC------------AGTAGAAAGTTTGGGCCAGAAATGATTCCGAAACATCTGAGGCGGAAGAGAGAACTCTATAAACAACAGTTTCTAACGGCAGAAAACAAAGAATTTCTGAAGGAT---GTTCAGTCTAGATCACTAAAACCA---GGCGAAAGTCCACTGAAAGAAGAACCTTGGCCGAGGGGAACGTGGACTCCAAATTCTGTAAGATGTGGAGTGATAACTAGAAAGATTGGCAGTTATCCAATGTGGATGAAAAATGGCAAAAGAGTGTTTTCAACGTTGTTGCAGATCGTTGACAATCACGTCATCAGGTATTATCCTCCAGAAAAATACGATCACACTGACACG------AAGGAAAGATTCAAGGGCTTGGCAGCTATTGTTGTTGGTTGTGATTCTGCAGATCCACAAATGTTTTCAGCTGAATATTCGGGCTTATTTAAAGAGTCGGGAGTTATGCCAAAAAGAAAACTCACCAAATTTTTCATTACTCCTAATTCTGCATTGCCACCAGGAACACCTCTGACTGTTGGACATTATAGAGTTGGAGACTATGTTACTGTTCACGCCAGATCAATTGCCCACGGATTTCAAGGTGTGGTGAAGCGTTGGGGATTCAAAGGAGGCAGAGCAAAGCACGGAAACACGAAAAGTCACCGACGACCTGGGTGTATTGGAGGCGGC---TCTAAAAAAGCTGGGATTTGGAGAGGAAAGAAAATGCCTGGAATGATGGGACAACGCAGGGTTTCGCTTCGTGGATTAAAGATTTTGAGGATCAACACGAAGTACAACGTCTTGTGGGTCAAGGGTCCAAACATACCGGGACACGTGCACCACTATGTCTACGTTACAGACTCTCACTCCTTTGGGAAATGCAAGACTGCAAACAGCCCACCGCCATTTCCTACTTATTATCCCGGGGAGAACGAGCCG---CTTCCGGAGGAGATGTACGCTTCGGACATTCACGCTTTCTCCGATCCCACCATCACTTACGAAGAAGAAAAAAAAGCGGCT------GTGGCCAAGAAATAA [1026]

Sitalc CAGGTGCGTGGAATCGGTCGCACGACCCGCAAAAGACGCACCACCCACCCTCCCAGGCACTACGTCCCCAAAAACAACAAC---CAACTCTTCGATTTTGTGACTCCCGAGAACAAACATTTATTGAAGGATGTAGCTGCTGATTTGTATCCTCCAAAG---ACGTCCAGTATTTTGAAAGATGCCCCGTGGGAGCGTGGCACATGGACTCCTGGTTCCAGGAGAACAGGCCTGATCTGTCGCAAGATTGGAATCTATCCGATGTGGAGGAAGAACGGGAAACGACTGCTCACCACACTTCTTCAGGTGGTGGATAACCACGTAATCAAATACCACCCACCGGAAGTGGTGGAAAAGAACTCTCCCAAACTGGCCAAGAAACGACCCGGGATGGGTCTGCTGATTGTGGGCGCGGAATCCACCGATCCAGAACGGTTCACAGGGCAGTACAACGGTCTTTTCAAAGAAGCGGGGGTTATGCCCAAGAAGAAACTTACAAGGTTTTTCATCACTCCTAACGCTGCTCTACCACCAGGCACCCCACTGTACGCTACCCACTTCCGACCTGGAGATTATGTCAACTGCCTCGGCAAAACGGTGGACCACGGCTTCCAAGGAGTGGTGAAACGCTGGGGTTTCAAAGGGGGC------AATTGGGGTCGCACAAAGACACACAGAAGACCAGGAAACATTGGAGCCGGAGCCGGCTGGCCAGACAATGTGTTCAAAGGAACTAAGATGCCGGGTCACATGGGATCGGAAGTCAGGATGCTCAGTGGTCTCAAAATATGGCGCATCAACACAAAATACAATGTGATATGGGTGCAAGGCCCAGCAATTCCCGGTGGCGTTAATTCCTTCGTAAACATTTACGACACCATGATCGAAAAAAAGTCTCACACTGCGGATAATCCACCGCATTTTCCTACTGCCTTTCCGGAGGACTGGGAAGAG---GTGCCGGAAGAATTGTACGACGAACAACTGCACAGGTTCACAGATCCTTCTATCACTTTTGAAGACAAAGACGACAAA---------CCCAAGCCAAAATAG [1038]

Sclero TCATTTCGAGAACTTGGAAAAACCACCCCTAAGCGAAGGAGAACTCACCCGAAAAGAGTTTGGGCTAAACGTAAATCAAAA---CTGGCTTTTGACTTTGTTACTCGGGAGAACAAAGACTTTATAAAGAATGTGGCCGAAGACATGTACCCCAAGCAA---AGTCCCAGCATCTTAAAGGAAGAACCCTGGGAAAGGGGCACTTGGACAACAGATTCTAAAAGAACTGGGTTGATATGCCGGAAGATTGGAATATACCCAATGTGGACAAAAACTGGCAAACGTTTTGACACAACACTACTTCAGGTCGTTGACAACCATGTTATTAAATATCATACACCAGAAGTTGTTGCCCAAAATTATCCCATGCTGGCAAAGAAGCACCCAGGAAAAGGAATCATGGTTATTGGAGCAAACAGTGCTAACCCTGAAATATTCACAGGTGAATACATTAACCTGTTCAACCAAGCTGGTGTGATGCCTAAGCGGAAGTTATCTCGATTCTGGGTAACACCAAATGCTGCTCTTAAGCCAGGTACACCTTTGTTTGCATCACATTTTCGACCAGGCGAGTATGTCAACGTCCGTGGACTCACGGTAGGACACGGATTTCAGGGTGTTGTAAAGCGGTTCGGATTCAAAGGTGGTCCAAAGTCTCATGGCTGCACGAAAAGCCATCGAAGGCCTGGAAATATTGGGGCTGGAGCAGGTCATCGTGATGAAGTTTGGAAAGGCACAAAAATGCCAGGACACATGGGACAAGAACGCAGCATTCTTATTGGTGTGAAGGTGTGGCGTGTCAACACGAAATATAATGTGATCTGGGTGCACGGGGGTGCCGTGCCTGGCCATGTTCATCACTATGTGTATGTGAATGACTGTGGCAGTGAAAAGAAAAGGCACAAGGAAGACAACCCTCCCCATTTTCCAACTGCATTTCAAGATGAGTGGGAAGAA---ATTCCGGAAGAATTGTTTGACGAGGAACTCCACAGATTTTCAGATCCTTCCGTAAGTTTT---------------------------GATAATGTCAAGTAA [1026]

Trogulus CGCCAACTACGAGTTGGAGAAACCAAATTTCGGAAGAAAGTACCACGTATTGCACCTTGGTGGCCTCAAAAAGAACTCACA---TTTAGAGACGAATATGTGTCTGATGAAAACAAAGAATACCTCCAAGAAGTGGTTAAGAAAACCTATTTGGCTTCT---GGGCAATCTCCACTGAAGGAACCTTTAGCTGCTAGAGGACAGTGGACCAATGACAGTCGGCGAACTGGTGTAATAGCTAAAAAAATTGGCATCTACCCAATGTGGACTACAAATGGCAAAAGAGTCGTAACTACACTTCTGCAGGTGGTAGATAATCACGTAATTCGCTATGTTCCACCTGAAAAAGTGAAAGAA---CGATTT------GGGATTCGATATGGAGATTTAGGTTTTCTCATAGTTGGAGCTGAAAATGGCGATCCACAATTGTATACTGCTGAATATAATGGTATTTTTACGGAAGCTGGGGTCATGCCAAAGAAAAGACTAACCCGTTTTACGATAAGTCCTGATGCAGCCTTACCTCCAGGAACCCCACTGTTTGCTACACATTTTCGAGCTGGTGATTTTGTAAATGTTGCTGGAGTAACTATAAATCGAGGATTCCAAGGAGTAATGAAAAGATGGGGTTTCAAAGGTGGGAGAGCCTCGCATGGAGTAACCAAAAGCCATAGAAGACCTGGCAATATTGGAGCCGGAGCGGGTCGAAAGGATAACGTTTGGAAAGGAACTAAAATGCCTGGTCACATGGGAAATGAAAGAAGAACAGCTGTTGGCTTACAGATCTTTAGGATAAATACAAAGTACAATGTACTGTGGGTGAAGGGAAATGCTACTCCTGGGGCAAACCATTCATACGTGTACATCATGGACTGCAAATTTCACAGGAAAATGCACGTAGAAGAAAACCCTCCTCCATTTCCAACATACTATCCGGAAGAAAACGAACCC---CAGCCTGAAGAAATCTGGGCACCCGATATTCACAGTTTTTCTAGTCCAACGATCACATTTGAAGACACTGAAGAA??????????????????????????? [1044]

Ortho CAAACGAGACAGTTGGGGAAGACTACCCTTAAAAGAAGAACTACACATGCCCCACGATGGTGGGTTCAAAGAAAAACAAAG---TTTAGTGAAGAATATCTTAGCAAAGAAAACAAAGACTATATCCAGGAAGTTATTAAAACCACTTGTCTAGAAGCC---GGGCAATCTCCACTAAAGGAACCACTATTCAAGCGTGGGACATGGACACCCGAATCTAGGAGGACTGGGGTCATAGCTCGAAAAATTGGTTCCTATCCACTGTGGACCGAAGGGGGCAAACGAGTTTTGGCAACATTATTGCAGATTGTGGATAATCATGTCATTCGCTACATACCACCAGAAAAAGTTAAAGAATCTTACTTA------GGTTATAGATATACAGACTTAGCATTTTTAGTGGTTGGAGCAGAGAGTGCTGATCCCCAACTGTTTACTGCTGAATATAATGGACTTTTCACTGAAGCTGGTGTTATGACTAAAAGAAAGCTTACCAGGTTCTTGATAACCCCCAATGCTGCACTAGCTCCAGGAACACCTTTGACGGCAACACACTTTCGAGCTGGTGATTTTGTGAATGTTTATGGGAAAACTGTCGGACGTGGTTTCCAAGGTGTAGTAAAACGGTGGGGTTTTGCTGGGGGACCAGCTGCTCATGGTAACACAAAAAGTCACCGAAGGCCAGGAAACATTGGTGCTGGAGCGGGTCGAAAAGATAATATATGGAAAGGAACTAAAATGCCTGGTCATATGGGACAAGAGAGGAGGACTCTAGCAGGTTTACAGATTCTGCGAATCAATTACAAGTATAATGTCTTGTGGGTTAAAGGTGATGCAGTTCCTGGTTCAACCCACTCATATGTTTACATTATGGACTGCAAATTGCACAAGAAAAAACACACGGATGATAATCCCCCACCTTTCCCTACATACTACCCAGATGAGACTGACCCC---TTGCCCGACGAAGTGTGGGCTAATAATATGCACAACTTTTCAAGTGAAACTATCACGTTTGAAGAAGGGGAGTCTCAAGTGGCAGCGGCAAAGAAGAAGTGA [1047]

Hespero CAAACACGCGACATGGGCGCCAATACGAAGAAACGTCGAACGACGCACGCACCTCGATGGTGGGTGGCCAGGAGGAGAAAA---TACGTAGAGGATTACTTGACAATAGAGAACAAACAGTTTCTCAAAGAAGTTGTTAACGAGAAATATCTAGATCCG---GGCGATACACCACTCAAGGACGATTTGTTTCAACGAGGCGCTTGGACACCACAGTCTCGAAGGACAGGGGTGCTGGCTAGAAAAATTGGCATTTATCCCATGTGGACTACAGAAGGGAAAAGATTTCAGACTACGTTATTACAAGTTGTGGACAACCATGTGATAAATTACGTGCCTCCTGAACAAGTACAAGAG---ACTCCTCAC---GGCTATAGAGCTAAAGGATTAGCCTATTTGATCATTGGGGCAGAAAGTTCAGATCCCCAAAAATACAGAGCAGCTTATAATGGACTATTCACTAAAGCCGGTGTAATGCCAAAGAGAAAATTAACAAAATTTATGATTACACCTGATGCGATTGTTCAACCAGGTACTCCATTATACGCCAGTCATTTTAGAGTAGGAGATTACGTAAATGTCTATGGGAAAACAGTTGATCACGGGTTTCAAGGAGTGGTCAATAGATGGGGCTTTAAAGGAGGAAGAAAAACGCACGGAGTTACAAAGAGTCATAGAAGAGGAGGAAATATAGGAGCAGGAGCGGGTCGAAACGACAATGTATGGAGAGGTACTAAAATGCCCGGTCATATGGGCTGTGAAAGAAGAACAGCTGTTGCATTACAAATATTTCGTATCAATACAAAGTACAATGTAATCTGGGTGAAAGGTGCGTCTGTACCTGGTCCTGTGCACTCATTCGTGCGTGTAATGGATTGTTTCCACGCTAAAAAGTGCCACACTGAAGAAAACCCACCCCCTTTCCCTACTTACTATCCGGAGGAGGAGGAAGAGAAATTAGCGGAACAGATCTACGCGGATCATGTTCATCAATTCGCTGCGGAAACTATTACGTACGAAGAGAAAGAACAAACCAAAAATGCTATCAAAAAGAAATGA [1050]

Leiobunum CAAACGCGGGATTTGGGTAAAACTACTCGCAAACGAAGGACTACCCATCCTCCTCGATGGTGGGTCAAACGGCGGGGTAAA---TATGCCTATGAAGGGTTAACCCAAGAAAATAAAGAATTCTTGCAAGAAACTATTCAGGAAAAAGCTATAAAAGCTTTGGAATCTAGTCCCCTTAAGGAAGCTCCGTGGGAAAGAGGAGTTTGGACTCCAGAATCTAAAAGATGCGGACTCATAGCTCGAAAAATTGGAATCTATCCTCTTTGGTTAAATAATGGGAGAAGAATATTAACAACACTATTGCAGGTGGTGGATAATCATGTTATAAATTATATACCTCCCGAATTGGTTACC------CGCCCT------GAGGCAAAATACAAAGGGAAAGGATTTTTAGTTGTTGGCGCTGAATCGGTGGATCCCCAAAATTACTCTGCTGCTTACAACGGGCTGTTTGAAAAAGCAGGGGTTATAACTAAAAGAAAACTATCTCGATTTACTGTAACACCGGATGCCAAAATACAGCCTGGAACACCTTTGTGTGCCTCTCATTTTAGAGTTGGTGATTATGTTAATGTGTTTGGGAAAACAGTTGGACATGGATTTCAAGGGGTTGTGAAACGATGGGGTTTTCGTGGTGGTAGAAAAACCCATGGTACAACAAAAAATCACCGTAGACCTGGCGCCCTTGGTGCAGGGACCGGTAGAAAAGATAATATTTGGAAGGGTAAAAAAATGCCTGGACACATGGGCCAAGAAAACAGGCTTTTAGTGGGTGTTAAGATTTTGAGGATAAACACAAAATATGACGTGATATGGGTCAGGGGACCTGCTGTACCTGGCCATGTTCACGCATTTTGTCAGATACATGATTGTTACGTGGATAAAAAACGTCACACTGAAGCAAACCCACCACATTTCCCAACATTCTACCCTGATGAGAATGAACCA---CTGCCCGAAGAAATATGGGACGCTTCGTTGCACAAATTCTCAAAGCCAACAATAACTTTCGAAGAGGAGGCAACAGCGAGTGTGGGC------AAGAAGTGA [1038]

Proto CAAACACGTGAGTTA---------ACACGCAAACGAAGAACAACCCATGCCCCACGATGGTGGGTCAAACGAAGGGGTAAA---TGGGGCTATGAAGGTTTAACCGCTGAAAACAAAGACTTCTTGCAAGAATCAATTCAGGAGAAAGCAGTAAAAACTTTAGAATTTAGCCCCCTTAAAGAAGCTCCATGGGAAAGAGGAGTGTGGACTTCAGAATCTAAAAGATGCGGTCTAATAGCTAGGAAAATAGGAATCTATCCTCTTTGGCTAAATAATGGGAAAAGAATATTAGCAACACTATTGCAGGTGGTTGATAATCATGTTATAAATTATATTCCACCGGAATTGGTAACC------CGCCCT------GAGGCCCAATACAAAGGGAAAGGGTTTTTAGTTGTTGGTGCTGAAACTGCGGATCCACAAAATTTCACAGCCGCTTATAATGGGCTCTTTGAAAAAGCAGGGGTTATAACTAAAAGAAAACTTGCTCGATTTACTATAACACCTAATGCCAAAATACAGCCTGGAACACCTTTGCACGCCTCTCATTTTAGAGTTGGTGATTATGTAAATATATATGGGAAAACAGTTGGACATGGTTTTCAAGGGGTCATGAAGCGTTGGGGTTTTGCCGGTGGGCCAAAATCCCACGGTACAACCAAATCTCACCGCAGACCTGGTGCTATTGGCGCAGGTGCTGGTAGGAAAGATAATGTTTGGAAAGGCAAGAAAATGCCTGGACACATGGGCCAAGAAAGGAGGCTTTTAGTGGGTGTTAAGATTTTGAGAATAAATACGAAATATGATGTGATATGGGTCAGAGGACCTGCAATACCTGGGCATGTCCACTCATATTGCCAAATATATGATTGTTACGTGGATAAAAAACGTCATACGGAAGAAAATCCACCTCACTTCCCTACATACTACCCAGATGAGAATGAACCA---TTACCGGAAGAAATATGGGATCCTTCGTTGCACAACTTTTCAGCACCAACAATAACTTTTGAAGAAAAA---GCTGAAAGTGCTGTA------AAGAAGTGA [1026]

[TICKFAST_NCHAR=612;Attractin and platelet-activating factor acetylhydrolase]

Ixodes GAGAGCTCGGACGATGACGACCGGTGGATGTCAGTGCACAAGGTGTTCCTGACCGAGGGTGCAGAAAAGGAGCCGGATGTGGTCATCTTCGGAGACTTCACGGTAGCATTTCTTCAGCAGTCCGAGGTGTGGGAAAAGTTGTTTGCTCCTTTGCATTGCCTCAACTTCGGCATTCCTGAAGATCGGACGCAGAATGCGCTGTGGAGGATTGAAAATGGCGAGCTGGACAACGTCGGCTCCAAGGTGGTGGTGCTGTCTGTTGGATGCAACAACTCTGGCGACAGTCCAGAGGCAGTAGCAGAAGGCGTACAGGCCTGTACAGAGGCCATTAGGAAACGTTTGCCCAATGCGCAGGTGGTTGTGTTGAAACTGCTTCCCTGCGGCCAGAAAGCCAATGCGCGGCGGGAACAGAGGCTCCAGGTGAACAACTTGCTGTCACGGGCGCTGAAGGGCCAGCCTGGCGTTCAGCTGGTGGACCTGGACCCGGGGTTTGTG------CGTCCCGACGGCACCATCAGCCACCACGACATGTTTGACTTCAATCACCTGAGCAAGCAGGGCTACAAGGCCGCCTTCGAACCCCTCGCAGAGCTGCTGGCCCAGCTCTTG [606]

Siro GAAGATGTCTTCGGAGACAACCGATGGATGTCTGTGCACAAGAGATATCTTTCCGAAGCAAAGGAAAAAGAGCCTGATGTTGTTTTTATTGGTGATTTTGTTGTTGCTATGGCTGCCCAGATGGAAGTTTGGGATAAACTTTTTATATCCTTACACTGTTTGAATTTTGGAATTTCCGAAGACAAAACGCAAAATGTTTTGTGGAGAATTGAAAATGGAGAATTAGGCACAGTGAATCCTAAGGTTGTAGTTCTTGCTGTCGGTGCAAATAATTACGAAGAAACAGCGGAAGAAATTTTTGAAGGAATTAAATCGTGCATTCAAGCAATTCTCAGTAAAGAATCTTCGGCACAGTTGGTTGTTTTGAAATTGTTGCCTAGTGGTCAAAACCCGAATCCACTTCGAGAGAAATTTAGCCAGGTTAATTCCCTCTTGGAAGCAAATTTCCACGGTGATTCGAAAGTCCACTTAACGAGTGTCGATCCTGGATTCGTT------AACACAGATGGCTGCATCAGTCGCCATGACATGTACGACTTTGCGCACTTGACGCGACAAGCTTACATGCGTGCCTTTGAACCGCTCGCAGATTTCTTGCAAGAATTACTA [606]

Sitalc GAGGACGAAATTGGTGATGGCAGGTGGATGTCAATGCACAAGAGGTTTCTAGTTGACGCCCAGGACAAGGAACCCGATGTAGTCTTCATTGGTGACTCTTTGGTAGCCAGAGTAGCTCAGAGCGAGGTCTGGGAACGTCTGTTCATCTCGTTGCACTGTTTGAACTTTGGAATCTGTACAGACAGAACGGAGAACGTTTTATGGCGCATCGTCAACGGTGAACTCAGCAACTGCATTCCTAAAGTAATAGTGTTGTCTGTGGGATCCAACAACGCTGATGAAACAGCTGAAGAGATTGCGCAGGGCATTCAGGGTTGCGTCGAATCTCTGCAAGCAGTGCAAGCTTCTGCTGCCATTGTGGTTTTGAAACTGCTCCCATGTGGTCAACACCCCAACAAGGTCCGCGACAAGTTTTCCGAAGTCAACAGCCTCCTAGATCAACACTTTGCG---ACCCCTGGTGTTCACCTCCTCAACTTAGACCCCGGCTTCGTG------GATAGCCACGGCAAAATAAGCCACCACGACCTCTACGACTACTGTCACCTGACTCGACCTGCCTACATTCGTGCCTTCGAGCCATTGGCTGATTTCCTGCAGGAGTTGCTC [603]

Sclero GAAGACAACATAGGAGACGGAAGGTGGATGTCAATGCACAAACGTTTTCTTGTGGAAGCTCAGGACAAGGAACCTGACGTGGTATTCATTGGCGATTCTCTTGTGGCAAGAGCTGCTCACAGTGAGGTTTGGGAGCAACTTTTCATCTCCCTTCACTGTTTGAACTTTGGCATTTGCAACGACAGGACAGAGAATATCCTGTGGCGAATTACAAACGGGGAGCTCGGCCAGTGCATTCCTAAAGTAATTGTTCTATCGGCTGGTTCAAATAACGTTGACGAAAGTGCCGAAGAAATCTCCGAGGGGATCGTAGCATGTGTCGATGCCCTTGTTTCTAACCAACCCTCCGCATCCATTGTAGTCCTGAAACTCCTCCCGTGTGGCCAGTTGCCCAACAAAGTGCGGGACAAGTTTACCGATGTGAACAATCTTCTGGATGAGAAGATGCTGTCGCAAACGGGAGTTCACCTGATGAATGTTGATCCCGGGTTTGTC------AACAGTCATGGCAGGATCAGCCATCACGATCTTTTTGATTATGCAAATTTGACTCGTAACGCCTACATGCGTGCCTTTGAACCGCTGGCCGATTTCCTACAGGAGTTGTTA [606]

Trogulus GAAGACTGCTATGGAGATGACAGGTGGAACTCTATGCACAAGAGATTTCTCATTGAAGCCAAGGAAAAGGAACCAGAAGTGATATTAATCGGAGATTTTGTTGTGGCATTAGCAGCTCATTCTGAGATCTGGGAAAAATATTTTATATCTATGCACTGTTTGAACTTTGGCATTAGTAGTGACAAAACACAAAATGTTCTGTGGAGAATCGAAAATGGTGAACTGGAGAACTGCAACCCAAAGATAGTTGTGCTGTCGATTGGTGCTAACAATTATGAAGATTCAGTTGAAGACATTGTGGAAGGAATTACTTCTAGTGCTAGCGCAATATTATCTCACCAACCTTCTGCTAAAGTCTTAGTTATAAAATTGCTTCCCTGCGGCCAGCACCCAAATTCTCTTCGAGAAAAATACCAGCATGTCAACGCTTGTCTAGAAACAGTTCTTAAGGATGTTTCAAATGTCCAGCTTGTTAATTTGGACCCCGGTTTTGTG------AATACTGATCATACCATAAGCCACCACGATCTTTACGATTATGTTCATCTCACCAGGAGTGCTTATTTGCGTGCCTTTGAACCATTGGCCGATTTGCTACAAGAGCTGCTT [606]

Ortho GAAGATTGCTACGGTGATGACAGGTGGAACTCAATGCACAAAAGATTTCTCATTGAAGCCAAAGAAAAGGAACCTGATGTGATTTTGATTGGAGATTTTGTTGTAGCATTAGCTGCCCATTCAGAGATTTGGGAAAAGTATTTCATTTCGATGCACTGTCTCAACTTTGGCATTAGTAGCGATAAAACCCAGAATGTTTTGTGGAGAATTGAAAATGGGGAATTAGAAAACTCTAATCCAAAGATTGTTGTTTTATCAGTTGGGGCTAACAACTATGCTGACTCTGTTGATGACATAACAGAAGGAATAACTGCTAATGCAAGAGCAATTTCCTCTCACCAGCCAGCTGCTAAAGTCTTAGTTATAAAATTGTTACCATGCGGTCAGTACCCAAACCCACTGCGAGAAAAATATCAACAAGTAAACAATCAATTAGAAACAGTCTTGAAAGATGCTCCCAACATTCAGTTGGTTAATTTAGACCCAGGTTTTGTGAGCACAGGCACAGACCACACCATCAGTCACCATGATCTTTATGACTATGTACATTTAACCCGCAGTGCTTATTCACGTGCCTTTGAACCATTAGCCGATTTGTTACAAGAACTGTTA [612]

Hespero ??????CGATACGGCGACGACAGGTGGAATGCGATGCACAAACGTTTCTTAATCGAAGCCAAAGAAAAAGAACCAGACGTTATTTTAATAGGAGATTTCGTAGTAGCATTAGCGGCACATGCAGAGATTTGGGAGAAATATTTTATATCCATGCATTGCTTGAACTTTGGCATTGCCAGTGATAAGACTCAGAATGTTCTGTGGAGGCTGGAGAACGGAGAACTCGATAACTGCTGTCCGAAGGTTATAGTTTTGTCTGTCGGAGCAAACAATTACGAAGACTCGGCAGAGGACATAGTCGAAGGCATCGTGTCCTGTTCTAAGGCTATCTTATCGCATCAACCATCGGCTAAAATTCTAGTCTTGAAATTATTACCGTGCGGTCAAAGCCCTAATTCCTTGCGTGAGAAATATCGTTTAGTAAACTGTCAACTGGACACTGCGTTGAGTAATGTCCACTCTGTCCAATTATTGAATATCGATCCTGGTTTTGTG------AATACGGATCAAACAATCAGTCATCACGACATGTACGACTATTGTCACTTGACCAGAACGGCTTATTCTCGTGCCTTCGAACCATTGGCCGATTTGCTACAAGAGCTTATG [606]

Leiobunum GAAGATATATATGGAGATGACAGGTGGATGTCTATGCACAAGAGGTTTCTAGTAGAGGGCAAAGTAAAAGAACCTGATGTCATTTTCATCGGAGATTCTGTAGTTGCATTAGCTGCACATTCTGATATTTGGGAAAAATATTTCATTTCAATGCATTGTTTAAATTTTGGCATAAGCGATGACAAAACTCAAAATGTTCTATGGAGGATTGAAAATGGAGAATTAGATAATTGCTGTCCTAAGGTTGTAGTGTTATCAATTGGTAGCAATAATTTTGAAGATACTGTAGATGATATTCTGAATGGAGTTGTAGAATGCACAAAAGCTATTGCATCTCATCAACCTTCAGCTACTATAGTAGTCTTGAAATTACTCCCATGTGGACTAAATCCAAACCCACTTCGAGAAAAATTTGCCCAGGTTAATGATAGATTAGATTTAGTTTTAAATGACATTCCCTTAGTTCAAATTTTAAATGTTGATCCTGGTTTTGTT------AATCATGATCTAACAATTAGTCACCATGACCTCTATGATTACGCTCATTTAACCCGTAATGCGTATTTGAGAGCCTTTGAGCCTCTGGCTGATTTATTACAAGAGCTGTTA [606]

Proto GAAGATATATATGGAGATGACAGGTGGATGTCTATGCACAAGCGATTTCTAATAGAGGGAAAGGTTAAGGAACCCGATGTCATATTCATTGGAGATTCTGTAGTGGCATTAGCGGCCCATTCTGATATTTGGGAAAAATATTTTATTTCAATGCATTGTTTAAATTTTGGCATAAGTGACGACAAAACTCAAAATGTTCTTTGGAGGATTGAAAATGGAGAATTAGATAACTGTTGTCCTAAGGTTGTAGTATTATCAGTTGGTAGCAATAATTTTGAGGATAGCACTGATGATATTCTAAGTGGAGTTGTAGAATGCGCAAAAGCTATTGCATCTCATCAACCTTCAGCTACTATTGTAGTTTTGAAATTACTTCCATGTGGTCTAAATCCGAATCCTCTTCGAGAAAAGTTTGCCCAAGTTAATGATAGATTGGATACAGTTTTAAATGACACCCCATTAGTTCAAATTTTAAATGTTGACCCTGGCTTTGTT------AATCATGATCAAACAATTAGTCACCATGACCTCTACGATTATGCTCATTTAACCCGTAATGCTTATTTAAGAGCCTTTGAGCCTTTAGCTGACTTACTACAAGAGCTTTTA [606]

[TICKFAST_NCHAR=537;COMM domain-containing protein]

Ixodes TTCACCAAAACGGCCAGCTTGCTGCAGGCCGTCAGCTTGATCAACCAGCTGGATGATACCAAGTTCAGCGCTCTCCTGGCACGCATACTACAGAAGCTGCACTTGAAGGACGAGCGTAGCTTCAGCGAGGAAGAGGAGTCTCGGCTGCAGAAGGCCTTTGCCCTGGGAGCCAAGGACGTCACCCTCGTCCTGGAGACTGTTTCCTTCATCCTCGAGCAGGCGGCCTTCCACGTGGCCAAGCCCCAGGTGCTGCAGGCGCAACTGACTGCCCTGGAGCTTACCGACACCAAGGTGCAGTGCCTGGTTCAAGCATGGACGGCCGGTGCCAAGCAGGTGGTGGAGAGGCTCAAGCACCACTCCCTGGCCGCCAGACAGCTGCAGGACGTGGGCTGGGAGCTGCGTGTGACGGGTGGCCAGCGCCACGCCGCACGTGCTCGGGCACCCCAGGCGCTGGTGGACCTGCTGGTGTCCGGTGGT---GCCGCCCACGACCACCTGCTGCTCCAGTTCTCCCACCAGCAGCTCTTTGGCCTCTAC [534]

Siro TTTGCGAAAACACCGCAACTTCTACAAGCAGTCACGTTGATTAACCGTATCGATGACACCAAGTTTCCATTTTTTCTATCTAGGATTATGCAAAAACTGCACCTAAAGGACGAAGAAAGTTTCACTGAAGAGGAAGAAGAGAAAATCCAAGTTTCGCTTTCACTCAAAAATGAAGAACTCAAACTTATTTTGGAAAGTTTAACTTTCATAATCGAACAGGCTGCTTATCATTTAGTGAAGCCGACTTTATTGCACCAATACCTTCAGGATCTATCCATAGATGACGAGAAAGTAATAGCTATTGTGCAAGCATGGACGACTCACGCCAAGTCAATCACCGAAAAGTTACGACAAACAACACTTGCGCCATCGCAACTGGTGGACGTTAATTGGCAACTTAGATTACAACTTGCACAATCGACCCTCAGCAAATTGAAACATCCTATTGGCGTCTTGGAATTGGTTTTATCTAAGGAC---GCAGCCAATAAAAGTCTTCAGTTTGAATTTACTCAAGATCAACTCTACAAACTTTAT [534]

Sitalc TTTACGAAGACTCCTAGACTTCAGCACGCAGTGCAGCTAATCAACAGTTTACCAAACCCCAAGTTTCCACTTCTTTTATCAAGAATAGTACAGAAGTTCCATTTAAAGAATGAAGAGAGTTTCAGTCAAGAAGAAGAAGAAAAGTTACAAGCATCCCTTTCCTTGAATGCAGAAGACGTTAAGCTTCTGCTGCAAACGTTATCGTTTTTCTTTGAACAGGCTGCTTATCACCTGTGCAAACCCCAGGTGCTTCAGCAGAACTTGGCTCTCATTGATGTCAGTGAAGAGAAGGTGGAGGCTATAGTGCAGGCATGGAGCAGTCAGGCTAGAATTGTGACTGAAGAGTTGCGCCAGAGTTCCCTGGCCCCTGCGCAACTCAAGGACCTGAGCTGGGAGCTGCGTGTGCAACTAGCACAGTCCTCCATTTCCAAACAGAAACAGCCAGTTGGGGTTTTGCAGCTGCAGTTGGATGGCCAG---CCCAAAAAGGAAAACCTTTCACTACAGTTCACGCAAGAACAGCTGTTCAGGTTTTAC [534]

Sclero TTCACAAAAACTCCGAAGTTGCAACAAGCCGTGGGCCTCATCAATAATTTAGATAATTCAAAGTTTCCACTTCTTCTTTCAAGGATCTTGCTCAAATGTCATTTAAAGGATGAGGAAAGCTTCACCACAGAAGAGAAGGAAAAACTGCAAAACTCTCTAGGATTGAATGCAAATGAACTGTGGCTTCTTCTTGAAACATTGTCCTTTATTTTTGAGCAGGCAGCGTATCACTTGTGCAAACCCCAAATTCTGAAACAAAACCTTGAAGAAATTGATGTCAATGAAGAGAAGATTGAAGCCATCATCCAATCGTGGGCCAGTCATGCCAAAGCGGTGACCGAGAGACTTCGCCAGCGCTCTGTGGCCCCTTTGCAGCTCAGCGACTTGACTTGGGAGTTGCGTCTGCAGCTAGCCCAGTCGTCCATTTCCAAAATGAACCAGCCAATAGGAACCCTGCAGTTGGAACTTGCCTCTCAG---AGCGGGAAGGAGAATTTGGGACTCCAGTTTACGCAGGACCAGCTATTCCATTTCTAT [534]

Trogulus TTTACAAGGACAGTTCGAATTGATGAGGCTGTATCCTTGATAAATAAATTGGATGACTCGAAATTTCCCCTGCTAATATCAAGGATTTTACAAAAGATCCATTTGAAGGATGAAGAAAATTTTACGCAAGAGGAAGAAGAGAAATTGCAGACTTCTCTTTCCTTAAATAGCAGAGAGATCCGACTGGTGTTAGAAACTCTTTCATTCATTCTGGAACAGGCTGCATACTATTCATCAAAGCCATCAGTATTAAACCATCATTTAGAACAAATAGGACTACAAGTCTCAAAGAGGGATTCTATGACACATTCTTGGACTTCAAGTGCTAAAGAAGTAACAGAAAAATTACGCCAACGCACACTAACTCCTTTACAATTAAAGGATGTCAACTGGGAACTTCGATTGCAGCTCAATCAATCTTCATTATCTAAAATAAAGAAAGCCATAGGAGTAGTTGAGCTCACTCTTTCCAGAGAGGAGAAAACAACTGAGAATTTGAATTTTGAATTCACTCATGATGAGCTTTATACATTTTAT [537]

Ortho TTCTCAAAAACAGCACGAATTGATGAAGCGGTATCATTAATCAACAAACTAGATGACTCAAAATTTCCTCTTCTGGTATCAAGAATTTTGCAAAAGATTCACTTGAAGGATGAAGAAAATTTTTCCCAAGACGAAGAAGAAAAACTTAAAAATTCTCTCACACTGGACAATAAAGAAATAAGGCTGGTGTTGGAAACACTTTCATTTATATTAGAACAGGCTGCTTATTATTCATCTAAACCAGCCACACTAAATCACCATTTGGAGCAAATTGGATTAAAAGATTCAAAAAGAGACTCTATGGTGCAATCTTGGGCTAGCAATGCTAAAGAAGTAACTGAAAAGCTACGCCAGCGCACATTAACACCTTTAAAACTAAAAGATGTTGGTTGGGAGCTAAGACTTCAACTCAACCAGCATTCACTGTCAAAGCTTAAGCAACCATTGGGAGTTGTCGAATTTACTCTCTCAAATGAAAACAAAACAACAGAGAATTTTAGCTTTGAATTCACTCACGATGAACTTTACGCATTCTAT [537]

Hespero TTCACTAAAACGAGCAAACTTGAGGAATCTATCGCGACGATTAACCAATTAGACGACTCTAAATTTCCGCTTTTAGTGTCGAGAATTTTGAATAAATTACATTTAAAAGATGAAGAGAATTTTAATGGGGACGAAATTGAGAAATTGGAGAATTCGTTGGGGATGAACAACGAAGATATTAGGAGAGTGCTAGAAACATTGTCATTCGTATTGGAACAAGCTGCTTATTATTCGTGCAAACCCGCAGTTTTGACCAATCATTTGGAACAAATTGGATTGTCGGAATCGAAATGCGTAGCAATGACTCAAGCCTGGACCAATAACGCAAAATCAGTCACGGAGAAACTTCGTCAACGTTCAATTTGTCCCGTCCAATTAAAAGACGTCGGTTGGGAACTCCGCCTCCAAATGTCCCAGAAATCATTGGGAAAATTGAAACAGCCAATAGGAGTGGTCCAATTGGGACTAGGGACGGACGGATCGGGGACAGAAACGATTCAATTCGAATTCAATCATCAACAATTGTATCAATTTTAT [537]

Leiobunum TTTCATTTAACAGCTAGGTTGGAAGATGCTATAAAAATAATTAATTACATAGATGAATCAAAGTTTCCTTTACTGTTATCGCGCATTTTGCAAAAGCTACACTTAAAGGAAGAAGAATGTTTTACCGAAGAAGAGGAGGTTAAATTACAGTCTTCATTTAGTGTAGATGAAAATCAACTAAGACTTCTATTGGAAACATTGTCATTTATGTTAGAACAGGCAGCTTATCATCTCTGTAAACCTTCAGTTTTTTCACAACAACTTGAAGCAGTCGGAATGGATCAAAATAAAATAGCAATCATGATTCAAACATGGACCAATAATGCAAAGACAGTTACTGATAAACTGCGTCAAAGAAATTTAGCCCCGGTTCAGCTGAATGATGTAAACTGGGAATTACGGCTTAAGTTAGCACAATCATCACTTGCAAAACTAAAACAACCCGTTGGAATTCTGCAATTAGATCTTTCATCAGGC---ACTAAGACAGAGAAAATCAACTACGAATTTGATCATTCCGAGCTTTATGCTCTTTAT [534]

Proto TTTCATTTAACTGCTCGATTAGACGATGCTATCAAAATAATAAATTACATAGATGAATCAAAGTTTCCGTTATTGTTATCAAGGATTTTACAAAAACTACATTTGAAGGAAGAAGAATGTTTTACAGAGGAGGAGGAAGTAAAATTGCAGTCCTCGTTTAGTATAGATGAAACACAACTACGACTTCTTTTGGAAACTTTGTCATTTATGTTAGAACAGGCGGCTTATCATCTATGCAAACCTGCAGTTTTTTCACAACAACTCGAGGCAGTTGGAATGGATCAAAATAAAATAGCAATTCTGATTCAAACATGGACAAATAACGCAAAAACCGTAACAGATAAACTGCGTCAAAGAAATTTGGCCCCAGTGCAGCTAAATGACGTAAACTGGGAATTAAGGCTTAAGTTAGCGCAGTCCTCTCTAGCCAAACTGAAGCAACCAGTTGGAATTTTGCAATTAGATCTTTCATCTGGC---AGTGCCACAGAGAAGATAAACTATGAATTTGATCATAGTGAACTCTATGAGCTTTAT [534]

[TICKFAST_NCHAR=375;DNA-directed RNA polymerase III subunit RPC9; SCSE]

Ixodes ATGAAGATTATCAAGGAGAGCTCGGCCTTCCTGAGCAATTTCGAAGTGCTCAAACTGCTCAAGGAGCTGCAGAGCGACAAGCCCAAGTCCGGCAAACACGCCAAGCACGTCCAAAACCTGGCCACGGTGTCGTACGAGACGGTCACCTACCTCGAACAGACCGCGTGCGCCCGTCAGAGCGAGGAGAACGTACAAAAGTTCCTCGAAGAGATCCGCGCGCTCCCTTTCACGCTGACCAAAGTGGAAAAGCTGCAACTCATCAACCACCGACCGACCACCTTCGTCGAGATCCAGCTGCTCATCGAGGAGAACGAGGAGCGACTCTCCGAGGACGACATGCAGACCATCCTCGACATCGTGGAGCGGACACTGCCC [375]

Siro ATGGAAGTTAAAAATGAAAATGCTGCTCTTCTTAGTAACTATGAGGTTTTAAATCTTCTAAAAGATTTACAAAATGAAAAG---AAAGGCCCCAAGAGTATGAAACGACAGCAGAATCTGGCAACTTTGTCATATGAAACAATCAAGTATTTGGAAAAGACACCATGTGTTCATCAAAGCCCAGATGCCATAAACAATTTTATGAAATCTTTGGAT------GCATTTTCACTCACGAGAGCTGAAAAACTTCAGTTGTTGAATCACAGGCCAACAACCCTCGTTGAAATCCAGCTGTTAATTGAGGAAAGCGAAGAAAGGCTGACGGAAGAAAACATGAACGAATTACTAGACGCTGTCAATCAACATCTCCCA [366]

Sitalc ??????GTT---GACGAAAATGCTGCACATTTGTCAAATTTTGAAGTGTTAAATTTATTAAAAGGTTTTCAGAGTGAATCG---AAAAATAGTAAAAGTTTGAAAAATCAGCAAAATCTGGCCACTCTCTCGTACGAGACAATAAAATATTTAGAAAAGACTCCATGTGCACTCCAAACTCCAGAGGTCCTAAAAACATTTCTGGAAGAAATCAAA------CGTTTCAATTTGACCAAAGCAGAAAAGCTGCAATTGCTCAATCATAGGCCATCATCTCTTGTGGAGATTCAGTTGTTGATCGAAGAAAGTGAAGAGCGTTTAACGGAAGACAACATGCAGGAGATTCTGGAGGTAGTTTCAAGAACCCTTCCT [363]

Sclero ATGGAAGTGATTGATGAAAACGCTGCCCTGCTCTCTAACTATGAAGTTTTAAAATTATTGAAAGGAATTCAGAGTGAGAAA---AAGGGCCGTGAAAGTTTGAAGCAGCAGCAAAATCTGGCAACCCTTTCCTACGAGACAATAAAGTATTTGGAAAAGACGCCATGTGCCCTGCAGAGCAATTCTGTCATAAAGAGTTTTATGGAAGAGATTAAA------CCATTTAATCTTACCAAAGCAGAAAAACTCCAGTTGTTGAATCACAGGCCTACATCATTGGTGGAAATTCAACTGCTGATTGAAGAAAGCGAGGAACGACTGAGTGAGGACAACATGCAAGATATCCTGAAAGTCGTCTCCCGGACGCTTCCC [366]

Trogulus ATGGAAGTGTTGGATGAAAATTGTGCTCTTTTGAGTAATTTTGAAGTTTTAAGTTTGCTGAAAGATCTGCAGAATGTAAAG---AAGCGTTCGGAATCT---ATGAAATGTGAAAATCTTGCCACACTTGCTTATGAGACAATAATGTACCTGGAGAAAACACCTTGTGCTCAGCAGACTTCTGAAGACGTTGAAAAATTTTTGAAAAAGATAAAA------AAATTTAATCTCACCAAAGCAGAAAAACTGCAGTTACTCAATCAGCGACCAACATCTTTGGTGGAAATACAATTGTTGATTGAGGAGAGTGAGGAGCGTCTCAGCGAAGAAAATGTCCAAGAAATTTTGAACATTATCTCTGAAACCTTACCT [363]

Ortho ATGGAGGTACTGGATACAGGATCTAAAATTCTTAGTAATGTTGAAGTTTTGTCTCTGTTGAAAGAATTACAAAGTGAAAAA---AAATATGCACAAGGT---AAACAGCAGCAAAATCTTGCAACACTATCTTATGAGACAATCATGCACCTGGAAAAAACTCCCTGTGCTCACCAATCACCTGGAGATGTAGGAACATTCTTGAAAGCAATTAAG------AAATTTGATCTCACTAAAGCAGAGCAGCTACAACTCCTAAATCAACGACCCACTTCATTGGTGGAGATCCAATTGTTGATTGAGGAAAGTGAAGAACGGCTAAGTGAAGAAAATGTACAAGAAATTCTAGAGATAGTGTCTCAAACATTGCCA [363]

Hespero ATGGAAGTATTAAATGACAATGAAGCCTATTTGAGCAATTACGAAGTTTTGAGTTTACTCAAAGGTTTACAAAATGAAAAG---AAAGACCCAAAAGCTATGAAGCAACAACAAAATTTAGCCACACTTTCCTACGAGACAATAATGTATCTTGAAAAGACCCCATGTAGTCATCAAAGCCCCGAGGCCATAGAGACTTTCATGAAAGCTATAAAA------CCATTTAATCTGACCAGAGCCGAGAAGTTACAGTTGCTGAACCAAAGGCCTACTTCTTTAGTGGAAATTCAATTGTTAATTGAAGAAAGTGAGGAAAGATTGACCGAGGAAAATGTTCAAGAGATTTTGGATTTGATTGGGAAAACTTTGCCT [366]

Leiobunum ATGGAGGTTTTAAACGATAATGCGGCGATGTTAAGTAATTATGAAGTCTTAAGCGTGCTAAGGGAGCTTCAATCTGAAAAT---AAAGGCCACAAAGTACAGAAACATCAACAGAATTTGGCGACTTTGTCCTACGAGACCATTAAGTATTTGGAAAAGAGACCATGTGCTCAACAAACGCCGGAAAATATCGAACAATTTATGGTTCAAATACAG------CCGTTCAATCTAACCAAAGCTGAAAAACTTCAACTTTTAAATCAAAGACCAACAACTTTGGTGGAAATTCAACTCATCATTGAAGAGTGTGAAGAACGTTTGACTGAAGAAAATGTGCAAGAAGTATTGGATATTGTTGCAAGTACTTTGCCA [366]

Proto ATGGAAGTCTTAAACGATAATTCTGCAATGTTAAGTAACTACGAAGTGTTGAGCGTTCTTAGGGAGCTTCAAGCGGAAAAT---AAAGGCCACAAAGTACAGAAACATCAACAAAATCTTGCTACTCTGTCCTACGAGACCATCAAGTATTTGGAAAAAAGGCCCTGTGCCCAACAAACAGCGGAAAATATCCAAGCGTTCATGATACAAATTCAG------CCCTTCAACCTCACCAAAGCTGAAAAACTTCAACTACTAAATCAAAGACCAACAACTTTGGTGGAAATTCAGCTTATCATCGAAGAGTGTGAAGAACGTTTGACGGAAGAAAATGTGCAAGAAGTGTTAGATATTGTTGCAAATACTTTGCCA [366]

[TICKFAST_NCHAR=447;F1F0-ATP synthase, subunit OSCP_ATP5]

Ixodes ATGCCTCCCCTAGCCGTCTTCGGAATTCCGGGACGTTATGCCACCGCCCTGTTTTCAGCGGCCTCGAAAGAGAAGAAGCTCGACACTGTTGAGAAAGACCTCCTGAAATTTAAGGGCCTGATTGAACAGGACAAGAGGTTGGCCCAGTTCGTCGATGACCCACTTATCAAGAAGTCACTAAAGAGGGATGCTCTGTCAGAGACACTGAAGAAGCAACAGTTCAACGCCCTCACCATCAATTTAGTTGGTGCTCTCTGCGACAACGGCCGGGCACGTGACGTCCGCGGCGTGATCAACGCGTTCAGCCGCATCATGGGTGCTGTACGTGGAGAGGTGCTGTGCGAAGTGGTGACTGCCAAGCCTCTTGATGCGGCGGCAGAGAAGGATCTGGAAGCTGCACTCCAGATGTTCCTCAAGAAGGGCCAAGTGCTGCTCATCTCCAAGAAG [447]

Siro AAACCTCCGGTCGCTGTATTTGGCATCGAAGGCCGTTACGCTTCTGCTCTGTATTCGGCAGCTTCTAAACAGAAAAAATTGGACAATGTTGAAAGCGAACTAAAAAAGCTGCAGGCGCTTCTAAAGACAGATGTGAAGCTTGCGGGATTTCTTTTAAATCCTTTGATTAAGGCTGCGACTAAAAAAGACGCTGTGTCATCTGCGTTAAAGAAACAGAACTACAGCGATCTAACTGTGAATTTAATTGGAGTTCTAGCAGATAATGGAAGATTAAACCGAATGAATTCAGTTATCAACTCCTTTTGTTCCATAATGAGTACGGTCAGGGGTGAAATCGTTTGCGAAGCCACTACGGCAAAGCCTCTCGATGCCGCTACATTAAAAGAACTGAAAGATGCATTACAAGGGTTTATGAAGAAGGGAGAAGTTTTACAACTGAACACAAAG [447]

Sitalc CAGCCCCCCCTTGCAGTTTTTGGCATCGAAGGAAGATATGCAACTGCCCTGTATTCTGCAGCCATGAAAGAAAAGAAGCTTGACGTTGTAGAAGGTGAGCTGGGAAGGTTCAGCGAACATCTCAAGAAGGACAAGAAGCTGAAGGAATTTCTGACCAACCCTCTTATTAAGGCATCTACAAAGAAAGAAGCTATGGAAACTGCCCTGAAGAAGGAGAAATTTACTAGTTTGGTCATCAATTTGATCGGCATAATGGCAGACAATGGGCGCATGAAGAAACTAGAAGCAGTCATTGGATCTTTCAACAAGATCATGAGTGCTGTCAGGAAAGAACTGGTTTGCGAAGTGGTCACTGCCAAGCCTTTAAATGCGGAAATGCAGAAGGAATTGCAGGCAGCTCTGGAAGGGTTTGCCAAGAAGGGAGAAGTATTGAAGGTGCAGAGTCGT [447]

Sclero AGGCCTCCCGTCCCTGTCTTTGGAATCGAAGGGCGATACGCCACTGCTCTGTTCTCGGCCGCTTCGAAAGAAAAAAAATTGGACATTGTTGAAGCAGAACTAAATAAATTCCAGGGCCATTTGCAGAAGGACAAGAAGCTTGCCGAATTTGTAGCTAATCCTTTGATTAAGGCCTCTATAAAACGTGAAGCAATGGAAACAGCTCTGAAGAAAGAAAAGTTTAGCAACCTCGTCATCCAGCTGATGGGCGTCATGGCCGACAACGGCCGCATGCCAAAAATCAACGCCGTTATTAATTCGTTCAGCCAAATTATGGGAGCCTACAGAGGCGAATTGGTGTGCGAAATAACTACGGCTAAACCGTTGGGCCCGGAAATGCAGAAAGAGCTGCAGGCCGCCCTGCAAGGTTTCACCAAGAAGGGAGAAGTTTTGAAAATTCAGAGCAAG [447]

Trogulus AAGCCTCCTATAGCTGTTTTTGGGATTAATGGCTGTTATGCAAATGCTCTCTACTCGGCTGCTTCCAAAGAGAAGAAACTTGATGCTGTTGAGTCCGAGCTGAAGAAGTTTAATGGTTTAATCAAGACAGATGTTAAATTAGCAGAATTTATGATGAATCCACTTATAAAATCATCTATTAAAAAAGATGCTCTTAGCACTGCTTTGAAGAAGCAAAACTACAGCAATTTAACCATCAATCTTATCGGTGTTATGGCTGATAATGGAAGAATGAAAAACATGACTGGAGTGATAAACACATTTGGTCAAATTATGGCTGCAGTTAGAGGCGAAGTAATCTGTGAAGTCACTCTTTCTAAGCCACTTGATGCAGTCATGATGAAGGAATTACAGACAGTCTTGTTAAGTTTTGTGAAAAAGGGACAAACCATTAACATAAATACAAAG [447]

Ortho AAACCTCCAATTGCAGTCTTTGGTATTGATGGGTCTTATGCTAATGCTCTCTATTCAGCCGCTTCAAAGGAGAAGAAACTAGATGCTGTTGAAACGGAATTGAAAAAGTTGGGGGAACTTATAAAGAAAGATACCAGATTTGCTGAGTTTATGCTGAATCCGCTTACAAAATCTACTGTTAAAAAAGAAGCGATAGGTGCAGCTCTTAAAAAACAGAATTTCAGCAATTTGACTATCAATCTTATTGGAGTAATGGCTGATAATGGAAGGATGAAGCACATGAATGGTGTAATTAATACATATGGACAGATCATGGCTGCCCTGAGAGGCGAAGTCATCTGTGACGTTACAACTGCCAAACCACTTGATGCTGTAACTTCGAAGGATTTGCAGGATGTATTAAAGGGATTCACAAAAAAAGGACAGAGCATTAAAATTAATACGAAG [447]

Hespero AAGCCCCCTATTGCTGTTTTTGGAATCGACGGTGTTTACGCCAATGCCCTTTACTCGGCCGCTTCCAAAGAGAAGAAATTAGATGCCGTCGAAACGGATTTAAAGAACCTTAAATCTCTAATTAAGAAGGATGTAAGATTAGCCGAATACATGGCTAATCCTTTAATCAAAGCAGCGGAGAAGAGAGAGAATATTGGCGTTGCGCTTAAGAAGCTCAACTATAGCAATCTAACGATTAATTTAATCGGTGCTTTGGCTGAAAATGGAAGAATTGGAAACATTAATAATTTAATCAATACGTTTGGACAAATTATGGCCGCCGTTAGGGGTGAAGTCATGTGCAGTGTCACTACGGCTAAGCCTTTGGATGCTGAAATGATGAAACAACTTCAAGAAGCCTTAGCCGGTTTCGTGAAACGGGGTCAAAATCTGAACGTTGAAACCAAA [447]

Leiobunum AAATCCCCTATAGCTGTCTTTGGTGTTGAAGGTCGTTACGCCACGGCTCTTTTTTCTGCGGCTTCCAAAGAGAAGAAAACTGATGTTGTTGAAAATGATTTAAAGAAATTTGGGTCTCTATTGTCCACAGATGTGAAACTAAGGGAATTTGTAAATAGTCCCCTGATCAAAACTTCAATTAAAAAAGATGCTTTGGAAACTGTCCTTAAGAAACAGAATTTTAGCAGCCTGACAGTAAATCTGTTAGGATTAATGGCTGAAAATAATCGCGTGAAGAATATAAAGCCCCTTATTGACATGTATGGTCTTATAATGGCGGCTGTACGGGGTGAAGTTATTTGCGAAGTAACTACTGCTAAGCCTTTGGATGCTGCCCAAAGTAAAGAATTACAAGATGCTCTTAAAGGTTTCATGAAGAAAGGAGAGTCAATTAAAATTCAAACTAAA [447]

Proto AAACCACCCATCACTGTTTTCGGTGTTGAAGGTCGTTATGCCACCGCTCTTTTTTCTGCTGCTTCAAAGGAAAAGAAGATTGATGTTGTTGAAAATGATTTAAAGAAGTTTGGGAATCTTTTGTCCCATGATGTGAAGCTTGAAGAATTTGTTCATAACCCGCTAATTAAAACATCAATCAAAAAAGAAATTTTGGAAACTGCTCTTAAGAAACAGAATTTTAGCAACCTTACAGTGAACCTTATTGGGTTAATGGCTGAAAATAATCGCGTGAAGAATATCAAACCCCTTATTGACACA??????CTGATAATGGCAGCTGTACGAGGTGAAGTAATTTGCGAAGTAACTACTGCAAAGCCCTTGGATGCTGCACAAAATAAGGAGTTACAAGATGCTCTTAAAGGATTCGTTAAGAAAGGAGAGGCCATCAAGATTCAAACTAAG [447]

[TICKFAST_NCHAR=420;N(6)-adenine-specific DNA methyltransferase]

Ixodes ???ATGGTGGGGATTGTCTGCATATCTTGTCCAACTCTCTACCGGAAACTGAAGGAGCTG---------------GAGTGTCACAAT---GAAGTTGTCCTGTTGGAATTTGACAAGAGGTTCGAGTGC---TATGGTGACGAGTTCATCTTTTATGACTACAATGATCCCTTGGGATTTGAGGAACGCTGGAAGGGCACGTGCGACGTGGTCGTGCTTGATCCTCCTTTTTTGTCCGAAGAGTGCCTCACAAAGGTATCCGAAACTGTCAAATTTCTCAACCCCAAGTTCATCATCTTATGCACAGGTGCCGTCATGGAACCGTACGCTCAAAGCCTT---CTGGACCTTCGTCCGTGCAAGTTTAAGCCAACACACACAAGAAAGCTGGGCAATGAATTCAAATGCTTTGCAAACTAT [396]

Siro CCTACCGGAAGAATAGCCTGCATTTCATGCCCTACAGTTTTCAAAAGTCTATTGACCATA---------------AAACCAGAGGAAGTGACAATTCAACTTCTGGAATATGACAGAAGGTTTTCCATC---TATGAAGAAGATTTTATTTACTATGACTTCAACGAGCCTCTTAACTTGAAAGAGGATTTACGAGAGACATTTAACGTAGTGCTCGCTGATCCGCCATTCTTATCGAGAGAATGCTTGGAAAAAACTGCGCAAACTGTCAAATTCCTTACTCAGGAGAAAATCATTCTTTGTACAGGTGCTGTGATGCAAGATATTGCCAAAGAATGT---CTGAGCGTAACGCCCGTCGAGTTCAAACCACAACACGCAAGAAATTTGGCAAACGAATTCCTTTGTTATTCAAATTTT [399]

Sitalc CCAAATGGGAAAATAGCATGTGTATCATGCCCCACAAGTTACCAGAAACTGAAGACTCTG---------------AAACCAGAAGGAGTACAGCTATTGTTGTTAGAGTTTGATAAGAGATTCTTGAGCGTAGCGGGAGAGGACTTTTTGTTTTACGACTACAACCACCCTGTA---ACACTGGAACACCTGCAAAGCCGTTTCGATGTTGTCTTGTCCGATCCACCCTTCCTTTCCGAAGAATGTCTTCAGAAATCTGCTCAAACTATCAAGTTTCTGGCAAAAGATAAGATCATTTTGTGCACAGGTTCTGTTATGGAGGAAGCAGTACAGAAGTGT---TTAGGACTCAAAGTTACTCAATTTAAACCCCAGCACTCTCGAAACTTAGGTAATGATTTCAGCTGTTTTACCAATTAC [399]

Sclero CGCAATGGAAGTATTGCCTGTGTCTCCTGTCCCACCTGCTATAAGAAACTTGTGTCCTTG---------------AAACCGGAAGGCGTTGGGCTGAAGCTGTTCGAGTACGATAGGCGATTTTCGTCTCTTGCTGGAAAGGACTTTGTACCCTTTGACTACAACCAGCCCGTG---ACGGACGAGGAACTCAGAGAGGGTTTCGACTTGGTGATTGCTGACCCACCTTTTCTGTCTCGGGACTGTCTCCTCAAGACGGCACAGACCATTCGCTTTTTGGCCAAAGACAAAGTACTGCTGTGTACAGGAACTGTAATGGAGGATATGGTCAAATCTTGCGACCTGGGTCTTCAAATCAGCAAGTTTAAACCTAAACATTCAAGAAACCTGGCTAACGATTTCAGCTGCTACATCAACTAT [402]

Trogulus CCGGGTGGAAGAATTGCGTGCATTTCTTGTCCAACCGTTTATTCCCGTCTGAGGCAAATA---------------AATCATAAGAATGCGCAACTAAAAATATTCGAATTTGACAAAAGGTTTGAGACT---TATGGATCGGATTTCATCTTTTATGATTATAATGAACCGCTCAATTTGGACACTGAACTAGAGGCAAAATTTGATATTGTTCTTGCGGATCCTCCATTTCTCTCAGAAGAATGTTTGGAAAAAATTGCAAAAACGATAAAATTTTTGACTAAGAAAAGAATAATTCTCTGCACAGGTACTATGATGAGTGAGTGCTCCAAGAAACTA---TTAAACCTAGAACTTTGTGATTTCAAACCATCACATGCTCGGAACCTTGGAAATGATTTCAGTTGTTTTTCAAATTAC [399]

Ortho TGTGGTGGAAGGATCGCCTGTGTATCATGCCCAACTGTATATTCACGTCTTCAAAAAATT---------------AATCAAGAAAATGTTGATTTGAAAATTCTGGAATATGATAAACGGTTTGAGAGC---TATGGTAATGATTTCATCTTCTACGATTACAATGAGCCGCTTAATCTGGATCCTGAACTTGAAGGAAGTTTTAACATTGTGCTAGCAGATCCACCTTTCCTCTCAGAAGAATGTTTGAACAAAGTGGCACAGACAATTAAATTTTTGACTAAGGCTAAGATTCTTCTGTGTACAGGCCTGGTTATGAGAGAACACGCAAAGGACTTA---TTAAATCTCGAACTTTGTGATTTTAAACCCTCACATTCCCGGAATTTGGGTAATGAATTTAGCTGCTTTATCAATTAT [399]

Hespero GCAAATGGAAGAATTGCTTGCATTTCCTGCCCGACTATATTCCGTTATCTTAAAAAGCAA---------------ACAGATACCAACTGTCAATCATTTCTGTTTGAATATGATTCTCGTTTTAATATT---TACGCCAATGAGTTTATTTTCTACGATTACAACCAGCCTTTAAATTTGGACACTAGTTTGAAAGAATCATTCGATATTGTTCTGGTGGATCCTCCATTTTTGGCCAGAGAATGTTTGCAGAAGGTTGCCCAAACAGTGAAGTTCTTGGCCAAAGGGAAAATCATTTTATGCACTGGTTTAGTAATGAAGGCAGATGTCCAAGAATTG---ATGAATCTAGAATTAACTGATTTCCAACCTACCCACGCGAGGAAATTAGGAAATGAATTTGCTTGTTTTACAAATTAT [399]

Leiobunum AATAATGGAAAAATTGTTTGCATTTCTTGCCCGACAGTATATAAGAAAATAAAAGATCTGCAGAAAACTTCACCAAATGAAAATGAAGCTAAAGTTTTATTGCTTGAACATGATTTAAGATTTGAGTGC---TACAATGAAGAATTTCTATTCTACGATTACAATGAACCACTCAATTTACCTGAAAACTTGAAAGGTCAATTTGATGTAGTTTTAGCAGACCCTCCTTTTCTATCTCGTGAATGTTTGGAAAAAGTGGCTCAAACTATAAAGTTTTTGACTAAAGATAAAATCATTTTGTGCACAGGAGCTGTGATGGAAGACATAGTTGCAGATCTC---TTAGGATTACATTCAGCTAATTTTAAACCAACTCATGAAAAGAAATTGGCTAATGAATTTAAATGCTTTAGTAATTAC [414]

Proto AATAATGGAAAAATTGTCTGTATCTCTTGTCCAACGATATTCAAGAAAATAAAGGATCTACAGAAAACTTCGCCAAATGAAAATGAAGCTCAAGTCTTACTGCTTGAACATGACTTAAGATTTGAATGT---TTCAATGAAGAATTTCTGTTCTATGATTACAATGACCCACTCAATTTACATGAAAACCTAAAGGGGCAATTTGATGTAGTTCTAGCAGACCCCCCTTTCCTTTCTCGCGAATGCTTGGAAAAAATGGCACAAACCATAAAGTTCTTGACTAAAGATAAAATAATTCTGTGCACAGGAGCTGTAATGGAAGACATAGTTGCTGATCTA---TTAGGATTACATTCCGCAAATTTTAAGCCAACTCATGAAAATAAATTGGCAAATGAATTTAAATGTTACAGTAACTAC [414]

[TICKFAST_NCHAR=435;NADH_ubiquinone oxidoreductase, NDUFB9_B22 subunit]

Ixodes GCTCATTTGGTCACTCGAGTCGTTTCGCACAAATTCCAGGTGTGCAGCCTGTACAAAAGGGCCCTGAGAAACCTGGAAGCATGGTGTGTCGAAAGAGACGACTACAGGATCCAGGCGGTCGAGCTACGGGAGAGGTTCGAGAACTACCGCCACATCAAGGATATGCGGATCGCCAAGGAGCTGCTGGATCAAGGCGAAGAGGAGCTGTTCGAGAAAGCGCACCCTCAGCTCTATCACCGTGAGCCCTTGCTGCTGCTTGGCCCGGTTGGCGGGAGGGACTCTTTCTCCCCGCAGGTGCTGGACTACTGGCATCCCCTGGAGAAGGCGCAGTACCCTGAGTACTTTGCCCGGCGAGAGGAGCGCAAGAAGGAGTACATAGCAAACTGGGAGAAGAAATACGGGAAGCCCCAGCCCGAGCACCAG---CACCACTAA [432]

Siro GCTCATCTGACGACAAGATTGTTTACACATACAAGAAAAGTTCAAAGTTTGTATAAGCGAGCGCTTAGAAATGTCGAAAGTTTCAACATGAGCAGGCTCACATATCGTTACGAGGCTGTACTTTTAAGACAAAGATTTGAGGAATTCAGACATGAGAAAGATATGAGGATTGCTAAACAGTTGTTACTCGATGGAGAAGAAGAGCTCTTCCAGAAAATGCACGCTCAGCCTAAGAAGTTTAACAACTCGCCAGGTGGCATCTGTTACGGCAGAGAAGTTCATCCTCCTGATTGGGTTTTGGATTTCTGGCATCCTCTGGAAAAAGCCCAGTATCCGAAATACTTTGCAACTCGCGAACTGCGTAAAAAGGAATACATTGAACGCTGGGAAAAAGAATTTGGTAAACCGGAGAAGAATGAT------TCTCACTGA [429]

Sitalc AATATTTATCAAACGAGAATTTTAACACATACTGAAAAAGTGTTAAGTTTGTACAAACGGGCTTGTCGAAATGCAGAAAGCTTCTGTGTAAATCGGAGGCAAGTTCGAATCCAGATGGTTCTCATGCGACAGCGTTTCGAGGAGAATCGTAATGTGATGGACCTGCGGGCGGCTGCTGCGCTGCTGAAAGCTGGAGAGGAAGAACTTGAGAAGAATATGCATTACAAGCCAAAGAAATTCATAACATCCCCTGGAGGCGTGGCTTTTGAACGTTACGGAGTATCTCCTGATTACGTTATGGATTTCTGGCATCCATTGGAGAAAGCACAGTATCCTACATATTTTGCACGTAGAGAGGAACGAAAGAAGGAGTACATCGAGAGATGGGAAAAAGAATATGGAAAACCAGCTATAGATGAT------CATCACTAA [429]

Sclero AATTATCTGCAAACGAAAGTTCTAACTCATACGCAGAAAGTTTGCAGTTTGTATAAACGTGCATTAAGAAATACAGAAAGTTATTGTACTATTCGTGAACAAGTTCGCTTCAATTGTGTAGTCTTGCGAGAGCGATTTGAGATACACAGACATGAGAAGGACATGAGAGTTGCCAACAAGCTTCTCATTGATGGAGAGGAAGAGTTATTCCAAAGCATGCACATTCAACCTTTGAAATTTTTTACTTCTCCGGATGGAACTGCACATGAGAGAGAAATGATAACATCAGATGGCGCTCTAGATTACTGGCATCCACTAGAAAAGGCTCAATATCCTGAATATTTTGCTCGACGAGAATTACGTAAACAACAGTACATTGAGAAATGGGAAAAGGAATATGGAAAACCTGAACCTACTGAAGGA---CATCATTGA [432]

Trogulus GCATTTTTACAAACGAAACATTTAAGTCATACTCAAAAAGTGTTAAGCTTATACAAACGTGCTTTGAGGAATCTAGACAGTCATGCAGAAGATAGATTAGAATTTCGAATTCAGGCAACTATACTGCGATCCCGCTTTGATGCTGCACGCAATGAAAAGGATTTGAGAGTTGCTGCCCAATTACTGTTGGAAGGCGAAAAGGAACTTCATAAAAATTTACACCCTCAACCCAAAATATTTGCTGCTTCTCCAGATGGAATTTTACAGGACCGTTACCTTACAAATCCTGATTGGTTGTTGGATTATTGGCACCCACTTGAGAAAGCGCAGTATCCAAAATATTTTGCTAAACGAGAAGTGAGGAAAACTGAGTATATCCAGAAGTGGGAGAAGCAGTTTGGAAAGCCTGACCCTGATGAAGGG---CACCATTAA [432]

Ortho GCATATTTACAATTGAAAAGTTTATCTCACGCCCAGAAAGTATGTAGTCTTTACAAAAGAGCATGCAGGAATGCAGAAAATTGGTGCTCTACTAGAACAGAAATTAGGATTGCTTGTGTAATGATGAGATCCCGATTTGATGCAGCACGAAATGAAAAAGACCTTCGAGTTGCAAATCAATTGTTAATAGAAGGTGAAAAAGAGTTGAAAACCAACAACGACGTACAACCAAAAATATTCTTAACTGCACCAACTGGTCTATTAACCGACCGTGCTTTTACACCACCTGACTGGGTGTTGGATTATTGGCACCCATTGGAAAAAGCCCAGTATCCAGAATACTTTGCCCGGCGTGAACAGCGGAAGGTTGAATATGTTCAAAACTGGGAGAAGAAATTTGGAAAACCTGATCCAAAGGACCAA---CTCCACTGA [432]

Hespero GCATATTTAACAACTAAAGTTCCAACCCATCGACAAAAAGTGATGAGTTTATATAAGCGCGCCCTTCGAAATATTGAAGCTCATAATTATGAAAGAACTGATATTCGCATAGAAAGCGTGATTATGCGTAAAAGGTTTGATGATCGCAAAGACGAAAAAGATCTAAGAGTTGCAGCCCAACTTTTAATTGAAGGAGAAGCAGAGTTGAGAGAAGAATGGCACATGCAACCCAAAACTTGGGCTGATTCAGCCACTGGAATATGCCAAGATCGCTACTTACCATCCCCTGATTATGTGTTAGATTATTGGCATCCTTTGGAGAAAGCACAATACCCTGAATATTTTGCCCGGCGAGAGCAACGCAAGGTGGAATATATTCAGAATTGGGAGAAAAAATACGGAAAGTTGGACCAACAGGAAGCGGGACACCATTAA [435]

Leiobunum AATTATTTGCAAACGAGGTATTTAACACACGCTCAAAAAGTGCTTAGTCTTTATAAAAGAGCTTGTCGAAATGTAGAATCCCATGAATTTTCTCGGGTAAATATTAGATTTAATCAAGTTATTTTGCGATCGCGATTTGATGCAAACAAAGATGAAAAAGATTTAAGAGTTGCTGCTCAACTCTTAGAAGATGGAGAAAAAGAGTTAATTAAGTACCTACATCCACAGCCTAAATACTTTCACGATTCACCTGGTGGAATATGTTTTAAACGTTACCCAACTTCTCCTGATTGGGTTTTAGATTATTGGCATCCACTAGAAAAAGCTCAATTCCCTGAATACTTTTCAAAGCGTGAACAACGCAAAAAAGAATACATTAAAATGTGGGAACAGAAATATGGAAAGCCAGATCCTGCTGAATTGCACGATCATTAA [435]

Proto AATTATTTACAAACGAGAGTTTTAACACATGCCCAAAAAGTGTGTAGTCTTTATAAGAGAGCTTGTCGAAATATAGAATCCCACGAATTCAATCGGGCAAATATTAGATTTCAGTGTGTTGTTTTGCGTGCGCGATTTGATACTAACAAGGATGAAAAGGATTTAAGAATAGCTGCTCAGCTTTTAGAGGATGGGGAAGAGGAATTGAATAAATTACTACATCCGCAGCCTAGATACTTTCATGATTCACCTGGAGGAATTTGCTATCAACGTTACCCAACTCCTCCAGATTGGGTTTTAGATTATTGGCACCCTCTAGAAAAAGCCCGATACCCTGAATACTTTGCAAAGCGTGAGCAACGCAAAAAACAATACATTGAAATGTGGGAAAAGAAATACGGAAAGCCAGATCCTGCTGAATTGCACGATCATTAA [435]

[TICKFAST_NCHAR=657;NADH_ubiquinone oxidoreductase, NDUFA9/39kDa subunit]

Ixodes CTGCGGAAAGGAACGGGAGGGCGGAGCTCCTTCAACGGCGTCGTCTGCACCGTCTTTGGGTCCAACGGCAGCCTGGGCACGTCCCTCATCAACAGGCTCGGGAAGATCGGCACTCAGCTGATCCTGCCGTACAGGTGCGAGTTCTACTTCATGCAAAGGCTCAAGCTCTGTGGAGACCTGGGACAGATCCACTTTCAGCCATTCAACCTCAAGGACGAGCTGAGCATTGCCAAGGCGATGAAGTACTCTAATGTCGTCATCAACCTCATCGGAAAAGACACCGAGACCAGCAACTTCCCCTTCAGCGAGGTGCATGTGAAGGGTGCCCAGACCATTGCCCGCATTGCCAGGGAGAGCGGGGTGCAGAAGCTCATCCACTTCTCCGCGCTCAACGCCACCGAGAGTCCCCGGCCCATCATC------AAGTTTGGGGGCTCCAAGTTCTACGCCAGCAAGTGGCTGGGTGAGCAGGTGGTGCGAGAGGAATTCCCGGACGCCATCATCTTCCGGCCGGCGGACATGTACTCCCATGAGGACCGCTTCATCCGCTACTACGTCAGCCACATTCGCAGGAACTATGTGTTG------ATGCCACTCTGGATCAAAGGCAATGGCATTGTGAAGCAGCCCTTCTTTGCGTTCGGACGTGCC [645]

Siro CTCAAACGGGGAACTGGCGGCAGATCTTCGTTTAGTGGGGTTGTGGCGACCATATTTGGTGCAAGTGGGTTTCTAGGAGGTCATGTTATTAATAAACTAGGTAAAGTTGGATCGCAGGTAATAATCGCACACCGTGGCGATCCCTATCGTATGCGGCCATTGAAACTGTGCGGAGATTTGGGGCAAATTTTGTACACGCCTATTGATTTAAAAGATGAAGCAACGCTTTACAAAGCGATGAAATATTCCAATGTTGTTGTCAATCTCATTGGTAAAGAGATTCCAACGAGCAATTTCTCTTTCGAGGATGTGCACGTGAAAGGCGCACGCAACATTGCTCGGATTGCTAAAGAGTCCGGGGTCCAGCGATTGGTCCACGTCTCGGCATTAAATGCTTGCGAACATCCAACCCCAGTGACCGGCAAATTCAAGAATGGGTCGCAATTTTATGCTTCAAAGTGGCGTGGAGAGCAGGCAGTTCGAGAAGAATTTCCCGAAGCCATAATCTTTCGTCCATCCGACATTTACGGGCAAGGAGACAATTTTTTGATGTACTATGGAGATTTTAACCGTCGCCAGATTCGGTAT------TTGCCCCTGTGGAACAAAGGAAAAAACATTTTCAAGCAACCGGTCTGCGTTTCCGATTTGGCC [651]

Sitalc CTCAGGAGGGGAACGGGAGGACGATCCTCGTTTAACGGAGTGGTCGCAACCGTATTTGGTTGCAGTGGCTTCTTGGGAAAACGTGTTTGCAATCAGCTGGGTAAAATCGGCACGCAGGTGATATTGCCTTACCGCGGATCTCATTATGATGTCTTACGACTGAAGTTGGTCGGAGACTTGGGACAAGTTTTATACTTTCCCTTCTATTTAAAAGATGAGGAGTTGTTGTACAAAGCCATGAAATATTCAAACGTGGTCATCAATCTAATTGGAAGAATAAATGAAACCAGCAACTTCTCATTCGAAGATGTCCATGTGACTGGAGCTCGCAACATTGCTCGTATCGCCAAAGAGTGTGGAGTCAAACGCTTGATTCACGTATCTGCGCTGGGCGCCACAGAGAACCCAAAACCTGTCATT------TTCAAGGGTGGCTCCAAATTCCTCGCTTCAAAGTGGCGTGGAGAGCAAGCGGTGCGGGAGGTGTTTCCGGAGGCCACCATTTTCCGCCCCTCAAGCATGTGGGGAGATGAAGACAAATTTCTCGTTTATTATGGAGCCCGCTGGCGTAGTAATCCTGACCGGAGATCGTTAGCTCTGTGGAACAAAGGCAAAGGGATCGTGAAACAGCCAGTCTACGTTAGCAATGTGGCC [651]

Sclero TTGAGACGTGGGACGGGGGGCCGCTCCTCTTTCAGTGGAGTCGTGGCCACGGTCTTCGGGGCAAATGGCTTGGTGGGACGCTCCGTTGTCAACCAACTAGGAAAAATTGGTTCCCAAGTGGTGGTCCCTTACCGCTGTGACCACTATAATGTACTACCTTTGAAGATGTGTGGTGATTTGGGACAAATTCTATTTTTTCCCATTCACTTGAAAGATGAGGAATCTCTCTACAAGGCCATGAAGTATTCTAACGTTGTCATTAATTTGATTGGGAGAATGGATGAAACTAGCAATTTCAGCTACGAGGATGTCAATGTGACAGGAGCCCGGACCATTGCCCGCATCGCTAAAGAGATTGGAGTGAAAAAATTAGTTCACGTCTCGGCACTCAATGCTGAAGAGCATCCTGCTCCCATCGTA------TTCAAGCAGGGATCTGGATTTTTAGCATCAAAGTGGCGGGGCGAAGAGGCTGTTCGAGAAGAATTCCCAGAAGCCATCATTTTCCGACCTTCCGTAATTTGGGGGGAAATGGACCGTTTCATCTATTACTATCCAAAATGGTATCGGTATGGTTTCAGCAAA---AAGATTCCCCTCTTCAAGAAAGGCGCAGGCATAGTCAAGCAGCCTGTTTACTTCAGCGATGTAGCC [648]

Trogulus CTTAAGCGCGGCACTGGTGGTCGGTCATCATTCAGCGGTGTTGTAGCCACCGTCTTTGGAGCAAATGGATTAGTGGGGAAAAGCGTAATAAACAAATTAGGCAAAGTCGGAACGCAGTTAATTTTGCCTTATCGAGGTGATCATTATGATGTGGTGCCGCTCAAGCTGGTCGGTGACTTGGGACAAGTTCTATATTTTCCTTTTGAACTGAAAAATGAGGATTCAATACGAAAGGCTGTGCAGTACTCAAACGTTGTCATTAATCTGATTGGCCGAGATTACGAGACCAACCACTTTTCATTCGAAGATGTTCATGTCACTGGCGCTCAAAGAATTGCGAGAATTGCAAAGGAAATGGGAGTTAAAAAGCTCATCCACGTTTCTGCTTTGAACGCGGTGGAAAAACCCACTCCAGTTTTA------TTGAAGAAAGGATCGCAGTTTCTGGCTTCTAAGTGGCGTGGGGAGCAAGCTGTCATTCAAGAATTCCCAGAAGCTATCATTTTCAGACCAGCTGATATTTGGGGTCCCGGAGACAGATTCCTTACATACTATGGTGGAATAGCTCGTCAGCACAGACATTAT------TTACCTATGTATAACCGCGGAGAAGGGATCTTCAAGCAGCCGGTTGACGTATCTAATTTAGGA [645]

Ortho CTTAAACGTGGGACCGGAGGCAGATCTTCTTTCAATGGAGTCGTGGCAACTATCTTCGGAGCAAATGGTTTCATTGGAACCAACGTCTTAAACAAACTTGGGAAGATTGGAACTCAGATAATTATTCCATACCGAGGGGATCATTATGAGATCATGCGACTGAAACTAGTTGGCGATCTTGGACAAGTTCTCTACTTCCCATTTGAGCTGAGAGATGAGGATTCAATACGCAAAGCCATGCAGTACTCTAACGTAGTCATCAACCTGATAGGCCGTGATCATGAAACCAGCAATTATAAATTTGAAGACGTACATGTCACCGGAGCCCGTAACATTGCACGAATTGCAAGGGAAATGGGTGTGAGAAAGTTCATTCACGTTTCTGCTTTGAATGCCAAGGAGAAACCTACACCTCTGATC------CTTAAGAATGGTTCTCAATTTCTTGCTTCCAAGTGGCGTGGAGAACAAGCAGTCATGGAAGAATTTCCAGAAGCCATAATATTTCGACCGGCTGATCTTTTTGGGCACCTAGACCGATTTCTCAATTATTACGGCAAATATTGGCGTCAAATTGCGTGTACC------TTACCTATGTATAATCGTGGAGAGGGGATCTTCAAACAGCCGCTATCTGGAAGTGACTTCGCT [645]

Hespero TTAAAGCGCGGCACAGGTGGCCGTAGCTCGTTCAGCGGTGTCGTAGCGACAGTGTTCGGTGCAAGCGGACTCGTAGGAGGAAATGTCGTTAACAAGCTGGGCAAAGTTGGATCGCAGCTCATTATACCATATAGGGGCGATCACTACAGCGTGCTCAGACTCAAATTGGCCGGAGATTTAGGACAAATTATATATTTTCCTTTCAATCTAAAAGATGAAAATTCATTGCGTAAAGCGATGAAATATTCAAACGTAGTTATCAATTGCATTGGCAAAGATTTCGAGACTGACAGTTTCTCGTTCGAGGATGTTCACGTGACGGGAGCTAGGAATATCGCCAGAATAGCCAGAGAAATGGGAATACAGAAATTGGTGCACGTTTCGGCACTGAATGCTCAACCCGACCCGGAACCCAGAATA------TTGAAAAAAGGATCAGGATTTTTAGCTTCAAAATGGCGAGGCGAGCAAGCCGTTCTCAACGAATTTCCCGATGCTGTTATATTTCGGCCTGCCGATGCGTGGGGTCAAGGAGATAGATTTATTCATTTCTACGGCTCATTGTGGAGGCAACATTTCCACTAT------GTTCCAATGTACAACAAAGGTGTGGGTGTTTATAAACAACCCGTTTCGTCTTCCGATTTAGCT [645]

Leiobunum TTGAAAAAAGGAACTGGTGGTAGATCTTCCTTCAGTGGAGTAGTTGCTACTGTTTTTGGAGCTAGTGGATTTGTGGGAAGACATGTTTGTAATAAATTAGGTAAAATTGGAAGTCAAATTATAGTACCTTATAGAGGCGATCATTATAATGTCCTCCGCCTTAAATTGTGTGGTGACTTGGGTCAAGTACTCTATCAACCATTTAACCTGAAGGATGAAGAGTCTCTTTACAAGGCTATGAAATATTCTAATGTTGTTATTAATTTAATTGGTCGTGACTATGAAACTAGTAACTTTTCTTTTGAGGATGTTCATGTTAAAGGAGCTAGAACTATTGCAAGAATAGCTCGCGAATCTGGAGTAGAAAGATTGATTCATGTTTCGTCACTCAATGTTTCTGAAAATCCAAAACCAATTATT------TTCAGAGGTGGATCAAAATTTTTGAAGTCAAAGTTTGATGGTGAAAATGCTGTAATAGAAGAATTTCCTGAAGCAATTATATTTCGACCAGCTGATATATGGGGTGCTGATGATCGTTTCATTCGCTATTATGCAAGCTATTGGCGATTACATGGTCTTAGC---ACTATGCCATTTTGGAATAAAGGTGAAGGTGTTACTAAGCAACCAGTGTATGTTTCTGATTTAGCA [648]

Proto CTGAAAAAGGGAACTGGTGGTAGATCTTCTTTTAGTGGAGTAGTTGCTACTGTTTTTGGGGCTAGTGGATTTTTAGGACGACATGTTGTCAACAAATTAGGTAAGGTTGGAAGTCAAATTATTATACCTTATAGAGGAGATCATTATGACGTTCTGCGCCTTAAATTGTGTGGTGACTTGGGTCAAATACTCTATCAACCATTTAACCTGAAAGATGAAGATTCCCTTTATAGAGCGATGAAATATTCTAATGTTGTTATTAATCTCATTGGCCGTGACTTTGAAACTAGTAATTTTCCTTTTGATGACGTGCACGTAAAAGGAGCAAGAGCTATAGCAAGAATAGCTCGTGAATGTGGAGTAGAAAGATTGATTCATGTTTCATCGCTTAATGTGTCTGAAAATCCAAAGCCAATTATT------TTTAGAGGTGGATCAAAGTTTCTGAAGTCAAAGTTTCATGGAGAAAATGCTGTAATTGAAGAATTTCCAGAAGCAATTATATTTCGACCATCTGATATTTGGGGATCTGATGATCGTTTCATTCGCTATTACGCAAGCTTGTGGCGATTACATGGATTAAGC---ACCATGCCATTTTGGAACAAAGGTGAAGGGGTTATTAAGCAACCAGTTTATGTTGCTGATGTAGCG [648]

[TICKFAST_NCHAR=408;NADH-ubiquinone oxidoreductase, subunit NDUFB10_PDSW]

Ixodes AGATTCTACAACGGCCTATACAACCTGTGGGACACGCCGGTCACTTGGTTTCGAGAGAAAGTGGTGGCACCCAACAGGAAGCAGTACTACTGGTACCACCGCCAGCTGCCGAGGGTGCCGGAAATCGACCAGTGCTACACGGACGATTTAATGTGCACGTTTGAGGCCAACGAACAGTACAAGAGAGACAGGGATGTTGACACAAGAATTCTGCAGATCCTGAGTCGGAGGCGCGACGACTGCTACATCTATGAGTCGCCCGAAACA---GAGAAGTGTAAGAAGCTGCACGAAGACTTCAGGGAGGCCGAGCTTAACTGGTTCATCAAATATGGCGACCTGGGCCCCCACGTGACGGTGGTGAACGCGTTCATGAAGCAGAAGCACCGGCTCGTCGCCGAGCGACGC [405]

Siro AGATTTGCTGATGCATTGGTTGCTGCTGTAGATGCTCCTGTTGTGTGGTTTAGAGAGAAAATTGTTGAACCGAACCGACCTACGAGTTACTACTACCACCAAGTTTTCCGTCGTGTGCCAACAATTGACGAATGTGATATCGATGACCGAACATGCTATTACGAGGCTAACCAACAATACCTACGAGACAGGAAGGTAGACGCAGCCATAGTGAGGCTTCTGGGTAAACGATTTGATGACTGCTTTTTTTATGAGTATCCACACCATAAAGAGAGATGCGTGAAGGAAGCAATTGATCGTGATGAAGCAAATTTAAATTATTTTATCAAATACGGCGATCTTGGAGCCTTCCACACCGTAAGAAAGTGTTTAATGAAACAGAAGCATCGTTTGATTTGGGAACGGCGT [408]

Sitalc AAAGCGGCCAACTTCGTAACAGCTATTTTTGATACCCCAGTGACGTGGTTTCGAGAAAACATCGTAGAGCCTAATCGTTCAGTCTACTATTACTACCACAGGCGTTATAGACGTGTTCCTGGAATCGAAGATTGCACCCTCGATGACATTGCCTGCAAATATGAAGCTTTTGAGCAATTCAAACGAGACAAAAAAGTGGATACGGAAATTTTGAACATCCTCCGCCGAAGGAAGAACGACTGCCTTCTATACGAGTCACCCAATCAGGAAGTCAAGTGTGCAAAGGTTATTCAGGACTTTAAAGAAGCTGAGCTAAATTGGTTTATCAAGTATGGTGATCTGGGTGCATATAGTAATGCCAAAGATGCTTTCATGAAACAGAAGCATCGGTTGATTTGGGAACGTAGG [408]

Sclero AAGTTTATTAATGCAGCATTTTTTGTTGTGGATACCCCGGTTACTTGGTTTAGAGAGGCTATTGTAGAACCAAATCGTTCAAAATATTATTACTACCACAGAAAATATCCAAGAGTTCCCACTATTGATGACTGTGAAGAAGAAGACTTTCCCTGTAGATATGAAGCAAATCAACAATACAAACGGGATTACCAAGTTGAATCAGAAATTCTGAACATATTACGTAAGAGGAAGGATGACTGCTACTTAGCTGAGTATCCAGATCATGAGAAAAGATGTCACCATGTTGAAAAAGATTACAAGGAAGCGGAGCTCAACTGGTTCATTAAGTATGGAGAACTGGGCATGTATGGTGATGCTCTCTGTGCTTACATGAAGCAGAAACATCGGATGATTTGGGAACGTAGG [408]

Trogulus AGATTTTATAATGCCTTGTTTAATATGGTGGATGGCCCCGTGACTTGGGTAAAAGAACGCATAGTTGAGCCAAATCAGAAGATATATCCCTATTACCATAAAAAATACCGCCGTGTTCCGACGATCGATCAATGTGAAGTAGGAGATGCTCTTTGCATTTATGAAGCCAACGAACAGTTTAAACGAGATCGACGTGTTGACAGTGAAATATTGAGTATTTTGCGTCGAAGAAAAGAAGATTGTTTCCTTTATGAGTACCCAAACCTT---GATCGCTGTACCGTCATTGCCAATCAATATGATGATGCAGCAGAAAACTGGTTTATTCGATATGGCGATTTAGGAGCATATTACCACGTAATTCCAGCCTTTATGAAGCAAAAGCATCGTTTGGTCTGGGAGCGTCGT [405]

Ortho AGGTTTTTTCAAGGAGTAGCAAATATTGTTGGGGGGCCTGTAACTTGGTTTAAAGAAAGTATTGTTGAACCCAATCAAAAGAAATACCCATATTATCATAGGAAATATCCCCGTGTCCCCACTATCGATGAATGTGAAGTTAATGATGTCCTATGCATATTTGAAGCCAACGAACAGTACAAGAGAGATAGAAATGTAGATGGTGAAATAATAAACATTCTGAGACGTAGACTGGATAATTGCGCAACTTATGAAAACCCTAATCACCAAGAACGTTGTGTTGAAATTCGGAAGATATACGAAGATGAGGCTGAAAATTACTTCATCCGATATGGTGATTTAGGTGCATTTCACCATGTGATTGGTGCCTACATGAAGCAGAAACATCGCATGATTTGGGAACGCAGG [408]

Hespero AAAATTACAAGGTTTTTCTTTAATGTGTTTGACGCTCCAGTCACTTGGTTCAGAGAAACTGTTGTCGAACCTAATCGAAGCACTTACTATTACTATCATCGCAAGTTTCCTCGAGTACCTAACATTGATGAATGTGAAGTTGGAGATCCTGTTTGTATTTTTGAAGCCAATGAGCAATTTAAGAGAGATAGGAGAGTTGATGGTGAAATTCTAAATATTTTGAGACGTCGGCAAGAGCAATGTAAGCTGTACGAGTATCCAAATCATAATGAACGATGCGCTGAGATCGGAAGGAAATATAAAGACACTGAATTGAACTATTTCATTAAATATGGTGATTTAGGAGGTTATCATCATGTCGCTAATGCTTTAATGAAACAAAAGCACCGTATGGTTTGGGAACGTCGC [408]

Leiobunum AGATTTGTAAATTACTTTTGCGATGTTGTTGAAGCTCCAGCTATTTGGTTCAGAGAAAGTGTTGTAGAACCTAACAGACCCAAATACAACTATTATCACCGCCAGTATCGTAGAGTTCCAACAATTGATGAATGTGAAGTTGGAGATACTATTTGCGTATTTGAGGCTGAACAACAGTTTAAAAGAGATAGACAAATTGACAGTGAAATTTTATGTATTTTACGTCGAAGGAAAGAGGATTGTTATTTTACTGAATATCCTAATACT---GATCGGTGTTCTAATCTTGTGGAGCAATATAACCATGCTTTAGAAAATTGGTTTATTAAATATGGAGATTTGGGGAAATATCAAGGAGCCATTGGTTGTTATTTCAAACAAAAACACCGCTTAATCTGGGAAAGGCGT [405]

Proto CGATTTATAAATTTAGCTTGCGATGTGATTGAAGCTCCGGCTATTTGGTTCAGAGAAAGTGTTGTAGAACCTAATAGGCCCAAATACAACTATTATCATCGTCAGTATCGAAGAGTACCAACAATTGATGAATGTGAAGTTGGCGATCCAGTCTGTATATTTGAGGCAGAACAACAATTTAAAAGAGACAGACAAATCGACAGTGAAATTTTAAATATATTACGTAGAAGAAAAGAGGATTGTTATTTTTCTGAATATCCAAATACT---GATCGTTGTGATAAACTCTTGGAGCAATATAATCTTGCTGTGGAAAACTGGTTTATTAAATATGGCGATTTGGGGAAATATCAAGGTGCTATAGCTTGTTATTACAAACAAAAACATCGCTTAATCTGGGAAAGACGT [405]

[TICKFAST_NCHAR=810;RING finger motif containing protein]

Ixodes TGTTCATTTATCAAGAAACCGAAA---------TTTAGGAAAGGCAATCAGCGAAAACGA---------AAGGGCAGCGATGAAGACGCTAGCAGCGAAGATGAAACCCAGATTGTCAAGAAAGACAAAAAATCGGAATTCTTGAATCCAATGATACAAGGTACATCATCAAAAAAGACGACAAAGAGCAATGTTGCAGCGCACAGTGACAGTGAGGAAGAACCGGTG------TCAGTTGGGGTGGCTTACAAGTCTGCTCGCACCACGGAAATGGAAGGTCCCAAGGATATGGGAGCTACTGCCATTCTTGAGATAGACACGGAGAAGGACAAGGATGCTCAGAGCATATTTGAACGCTCACAGCAGATCAACAAGGCGAGCTTTCCAAATATTTTTGCAGAACTGAAAG---GGAAAGCTGATGACAAGGTATACAGAGGGGTGAATAACTACACACAATACATCACCAAGAAGGACACGGCCCAGGGAAATGCATCAAGTGGAATGGTCAGGAAAGGGCCGATTCGAGCCCCAGAACATATCCGTTCTACTGTTCGGTGGGACTACCAACCCGATATTTGCAAGGACTACAAGGAGACTGGCTACTGTGGTTTTGGGGACAGCTGCAAGTTCATGCACGACCGGTCAGACTACAAACATGGCTGGCAGTTGGAGATGGAAATGGAGCGAAACCAGTACGGGGAAGAG---GACACAAGTCGTTACGAAATCAGCTCCGATGACGAAGACTTGCCCTTCAAGTGCCTCCTATGCCGCAAGTCCTTCGTCGACCCCGTCGTCACCAAG [780]

Siro TGCAAATTCTTGGTAAAGAAA------------AGGAAAAATCAGCAATCCAGAAAACGGAACGCCAGTGATGAAAGTAACAGCGGTGGAAGTAGCGCCGATGAAACCATTGTTTTCAAAAAAGGTAAAGCTAGCGGTCGACGTAATCCCATGATTCAAGGAACCAAAAATGTAACAACCAAAGAGAAAGTAAAT---------TACGACAGTGATGAAGAACCGAAA------GAATTGGGTGTCGTTTATAAATCGAGTCAAACGACTGATCGGTGCGGTCCCTCCGACATGGGGGCAACTGCCATCCTCGAAACCGAAACTGAAAAAGATAAGGATGCTCAAAGCATCTTTGAGAGGGCACAAAAGGTCAATGAT------------------------GACTTGAAAGATAACGAACAAGATGATAAAGTCTACAGGGGTATCAATAACTACAAACAGTATATTAAAAAGAAAGACACAGCACAAGGCAACGCTTCGAGTGGTATGGTTCGACAAGGTCCAATCAGAGCGCCCGAAAACATAAGGGCAACCGTGAGATGGGATTACCAACCAGATATTTGTAAAGATTACAAAGAAACTGGATATTGCGGTTTCGGAGATAGTTGTAAGTTTATGCATGATCGCAGTGACTATAAGCACGGCTGGCAACTCGAAATGGAAATGGCGAACAACACGTATGGCGAAGAG---GACACGAGCAAATACGAAATTGATTCGGACGAAGAGAAACTTCCATTCAAATGTTACATCTGTCGTAAGTCTTTCACAGACCCAGTCGTCACCAAG [756]

Sitalc TGTAAGTTCATGATCAAACGTCCGAATTTTCGAAGAGGTCAGCAAAATTCTCGTAAACGGAAC------CAGGAAAATAGCAGTGGAAACAGTAGTGATGATGAGACCGTAGTAGTAAAGAAAGACAAGAAGTCCGATCTAATAAACCCAATGATTCAAGCAACCAAGAAAGGGCAGGAAAAAACCTCTGTGAAC---------TACGACAGCAGCGGAGACGAGAAA------GATGTAACTGTGTCTTACAAGTCAAAAATGACAGCTGATAGAGAAGGCCCACGAGATATGGGTGCCACATCTATCGTTGAGACTGAAACAGAGAAAGACAAGGATGCCCAGAGCATATTTGAACGCTCGCAGAAGATCAATGAG------------------------GAGATAAAA---GGCAAAGATGATGACAAAGTATACCGTGGTATTAACAACTACGTCCAGTACATTAAGAAGAAAGATACTGCTCAAGGAAACGCCTCCAGTGGGATGGTTCGAAAAGGACCAATCAGAGCACCCGATAACTTGAGAGCTACAGTTCGTTGGGATTACCAACCGGACCTGTGCAAAGACTACAAAGAAACGGGTTACTGTGGTTTTGGTGACAGTTGCAAATTCATGCACGACCGGACTGATTATAAGTTTGGCTGGCAGCTTGAAATGGATTCGGGAAGCCACAATCAC---GACGACAATGAGGAAGGTAGATACGAAATTCCTGACGATGAAGATAATCTTCCTTTCAAGTGTTGCATTTGTCGTAAATCTTTTAAGAATCCTGTGGTTACTAAG [759]

Sclero TGCAAGTTTTTGATAAAACGCCCAACC------AAAAGGCAACAGAATGCTAGAAAACGACAA------GAAGTTACCAAAAGTGGGAGCAGTAGTGATGATGAAACGATAGTCGTTAAGAAAGATAAAAAAGACACTCTCAGAAATCCAATGATACAAGGAACCAAACTTCCGCATGAGAAACGGGCTGTCAAT---------TACGCAAGCAGTGGAGAAGAAAAC------GATCTTTCAGTTTCCTATAAATCAGAAAAAGTAACGGTACGCGAAGGTCCGAATGACATGGGTGCCACATCGACCATTCAAACGGAAACGGAAAAGGACAAAGATGCCCAGACTATATTTGAGCGCGCTCAAAAAGTGAACGAA------------------------GACCTCAAA---GGCCAGGAAGATGATAAAGTCTATCGAGGAATCAACAATTACGTTCAATACATCAAAAAGAAAGATACTGCGCAAGGCAATGCATCGAGTGGGATGGTGCGCAAGGGCCCGATAAGAGCCCCAGATAATTTGAGAGCAACCGTTCGCTGGGACTACCAACCGGACCTGTGCAAAGATTTCAAAGAAACTGGGTACTGCGGCTTCGGTGATAGCTGCAAATTTATGCACGATCGTACAGATTACAAGTTTGGTTGGCAGTTGGAAATGGAATCAACAAATCGT---------GATGAC---GAAGAAGGTAGATACGAGATTCCATCCGACGATGAGAATTTGCCGTTCAAGTGCTGTATGTGTCGTAAATCCTTTAAAAATCCGGTTGTGACCAAG [744]

Trogulus TGTAAGTTTATAAGGAAAAGGCCA---------AATCGTCAACAGAATGCACGGAAGCGGCAAATTCATAACAGTAGTAGTAGTAATGGTAGCAGCGAGGATGAAACTATTATTGTTAAGAAGGAGAAAATATCTAAATGGAACAACCCTATGGTGCAGGGAACTAAACCAGCCAAGAAACCCACTAGAAATGAACAAACAGTTGATGATGATGAGGAGGTGATAGATTCTAAAGCTATTGGAGTTTCTTACAAATCTAACGAGAGCACAACAGTTGCTGGCCCTTCAGACATGGGTGCAACTGCCACTGTACAAACGGAAACCGAAAAAGACAAAGATGCTCAAGCTATATTTGAAAGGGCTCAGCAGATCAATGAA------------------------GGTGTAGCT---GCTAAT------GATAAGGTTTATCGGGGTATTAACAATTACAAGCAGTACATAAAGAAAAAGGACACAGCCCAAGGCAATGCATCAAGTGGAATGGTTCGTCAGGGACCTATCAGAGCCCCAGAAAACCTAAGGGCAACAGTCCGATGGGATTACCAACCAGATTTGTGTAAAGACTATAAAGAAACTGGTTACTGTGGCTTTGGTGACAGCTGTAAATTCATGCATGACAGAACAGACTACAAATACGGGTGGCAGTTGGAAATGGAAGCTAACAACACTAATAAT---GAT------GATTCTGCAAAATACGAAATTAATTCAGATGATGAAAACTTACCATTTAAGTGTTTTATTTGTAGAGGTCGATTTAAAGATCCAATTGTTACAAAA [759]

Ortho TGTAAGTTTATAAGAAAAAGACCA---------AATCGACAGGAAAACACTAGAAAAAGGCAGGTC---CATAGTAGCAGCAGTGACAGCAGTGGTGGTGATGAAACCGTTATCATTAAGAAAGAAGCCAAGGCTGGGTTGAAGAACCCTATGGTTCAGGGAACAAGATTGGCTAAGAAACCGACTCGTGATGTAAAA------AGTGATGATGAAGAAGCTGAAGAT------GAAGTGGGTGTGTCTTACAAATCTAATCAAAGCACAACAGTTGCTGGACCATCTGATATGGGTGCTACTGCCACAGTCCAGACAGAAACTGAGAAGGATAAAGATGCACAAGCTATATTTGAAAGAGCCCAGCAAATAAATGAA------------------------GACTTGACT---------ACCGATGATAAAGTTTATAGAGGCATCAACAACTACAAACAGTATATAAAGAAAAAAGACACTGCTCAGGGCAATGCCTCAAGCGGTATGGTCCGACAAGGTCCAATAAGAGCCCCCGAAAACCTGAGGGCAACCGTGAGATGGGACTACCAGCCTGACCTCTGCAAAGACTACAAAGAAACTGGCTACTGCGGCTTTGGTGACAGTTGTAAATTTATGCATGACCGCACAGACTACAAATACGGTTGGCAACTTGAAATGGAAGCAAGCAATGCA---AAT---GAA------GATCCCTCAAAATACGAAATTGATTCTGATGAGGACAACTTACCTTTCAAGTGTTTTATCTGCAGGGGTCGTTTCAAGGACCCTATTATCACCAAA [741]

Hespero TGTACTTTCTTCATGAAAAGAGCATCA------AATCGTAAACAAAACGCCCGTAAAAGGGCC------GTCACAGATTCTAGCAGTAGTGGCAGCGATGAGGAAACAGTTGTTGTCAAAAGAGATAAGAAAAATGAATCGAAGAATCCAATGGTTCAAGGAACAAAACCTGCTAAAAAGCAAGTTTTGACT------------CATGAAAGTGAGGAAGAAATGGACCCTGAGAAATTCAGCGTTGCTTATAAATCCAATCAGGACACTTCATTAGCCGGATCATCAGATACTGGAGCTACAGCCACAATTCAAACCGAGACTGAAAAAGACAAAGATGCTCAGAGTATATTTGAAAGGGGCCAGATTGTTAATGAG------------------------GAGAATAAA---GAAAAAGGAGATGACAAAGTCTACAGAGGTATAAACAATTACATCCAATACATCAAGAAAAAAGACACCGCTCAAGGCAATGCTTCTAGCGGTATGGTTCGTAGAGGTCCTATTCGAGCCCCTGAAAACTTGCGTGCTACTGTTAGATGGGATTATCAACCAGATCTATGTAAGGATTACAAAGAGACAGGCTATTGTGGGTTTGGAGACAGTTGTAAATTTATGCACGACAGAACTGATTACAAATACGGTTGGCAGTTGGAGATGGATTCTGCTGCTAAT---CAC---GAA------GATGACGCTAAATATGAAATAAACGACGATGATGACAGCCTGCCTTTTAAATGTTACATTTGTAGGAAATCTTTTGTAGATCCTATAATCACAAAA [747]

Leiobunum TGTAAATTTCTCATGAAAAGA------------GTTCGGAAGGTTAATACCCGAGTACGT---------AAAAATAGAGATTCTTCAAGTGATAGTAATGATGAAACTGTTGTGGTTAAACCAACCAAGAAGCCATGCCTCAAGAACCAAATGATTCATGATTCTAATTCTAAAAAATCAAAGTCATATCAAGAAGAAGCA---GAAAATAGCAAGAGCGACGGTGAGCCCACAGATGTTTCTGTTTCCTACAAATCTACTGGA------TCATTTGAAGGTCCAAGTGATATGGGAGCGACAGCGACAGTTCAAACAGAGACAGAGAAAGACAAAGATGCGCAGAGTATATTTGAACGAGCCCAAAAAATTAACGAA------------------------GAGCTGAAAAAGAAACCGAGTGAAGATAAGGTTTATCGAGGATTAAATAACTACACACAGTTCATTTCAAAGAAAGACACTGCTCAGGGTAATGCCTCAAGCGGTATGGTGAGAAAAGGTCCCATAAGAGCTCCGGAAAATGTTAGAGCTACTGTAAGATGGGACTACCAGCCAGACCTCTGCAAAGATTACAAGGAAACTGGGTATTGTGGCTTTGGGGATAGCTGCAAATTCATGCATGACAGAACTGATTACAAATATGGTTGGCAACTAGAAATGGATGCCAACAACGAAGCTCAC---GATGAT---GATGATACAAAATATGAAATTGATTCTGACGAAGAGAAGCTGCCATTCAAATGTCTCATTTGTAGAAAATCATTTAAGGATCCTGTTGTTACTAAG [750]

Proto TGTAAATTTCTTATGAAAAGA------------GTTCGGAAGGTTAATACTAGAGTTCGT---------CAAAACAGGGAATCTTCAAGTGATAGTAATGATGAAACTGTTGTGGTAAAACCTTCGAAGAAGCCATGCCTTAAAAATCAAATGATACATGGATCTAAATCTAAAAAATTAAAATCATACGAAGAAGATGAA---AAAGGCAACAGTGATGAAGAACCCACTGGCGATGTTTCCGTGTCCTACAAATCTACTGGT------TCATTTGAAGGCCCAAGTGATATGGGAGCAACAGCAACCGTTCAAACTGAGACAGAAAAGGACAAAGATGCGCAAAGTATATTTGAGCGAGCCCAAAAAATTAATGAA------------------------GAACAGAAAAAGAATACTAGTGAAGACAAGGTTTATCGAGGATTAAATAACTACACACAGTTCATTTCAAAGAAAGACACGGCTCAGGGTAATGCCTCCAGTGGAATGGTGCGGAAAGGTCCCATTAGGGCTCCGGAAAATATTAGATCAACTGTAAGATGGGACTATCAACCAGACCTTTGCAAAGATTACAAAGAGACGGGTTATTGCGGTTTCGGAGATAGTTGTAAGTTCATGCATGACAGGACTGATTACAAGTACGGTTGGCAATTGGAAATGGATGCCAATAATGATACTCAC---GATGAC---GATGACACTAAATACGAAATTGATTCTGACGAAGAAAAGCTTCCATTCAAATGTCTCATTTGTAGAAAATCATTCACCGATCCGATTGTTACAAAA [750]

[TICKFAST_NCHAR=615;RNA polymerase II, large subunit; part20; SCSE]

Ixodes ATGCTGCTGATCCTCGACGACGACCACAAGCAACACCTCCACTTCATTCGCGACGTGGACGTCGCGGTAGCCCGGGAATTCTGCAAAATCGCCAACGAGTTCCTCCTCCGCGGCGTCAATCCCAAGGTGTACCACTCCGCTGCGCAGAAGCTCCAAGTCGAAGACGACGTCGTGCAGAACGCCGTGGAGGGCATCTCGCACCTCCTCAGCCAGGCGGCCAAGTTCAAGCTCAGCGAACAGCAGTTTCAGGAATCGCTTCTGCTGCTCAGCCTCCCCGAGCCGGTGTGCCAAGACTTGGTCGGCTATCATGTCGAGAACGAGAAGGGACTTCGGAAACTTCTGAGCAGGAAGGCCCTTCCGTGCTGGAGCTTCTCGGGTCTCGAGTGGCGACTGGAAGCCGAGGTAGCTAGGCGCTGCATCAAGTCTCATGTCCGGCCCCTGGTCACCTTCAGGCTCAAGCTCAAGAAGGAAGACGGGGAGAGCCTAGAAA------------------TCGTCCTCCAGACCGATCCTTCGAACCTTATTCATATGACAGAGTCGTTGGAGGAAGCGCTTCAGTGTGTTAAATCGCAGCATATGCGCAAGATTGTCAAACACGTC [597]

Siro ATGATATTGATTTTAAGTGACGAGCATAAAGAACACTTGCAGTTTTTGTCGCAAGCAAGACCAGAAGTAAGTAAAGAATTCTTGAGGATTGCCATAGAATTTTTAGTGAAAGGTTCAAATCCGAAAGTCTATCAGTCAGCAGCTCAAAAACTTGAAGTAAATGCGGAAACTGTTCAACATACAATAGAAGGGCTTATGTATCTGTTAACAGAAAGTATTAAAATGATGCTAAGCGAATTGGAATTCCAAGACTCGATTCTGAGCCTGGGATTGAGCGACGAACTGCAGAGCGAATTGCTGACTTGTTACGTGAACAACAAGCAACAAATAAGACAGTTTCTGGGTCAGAGTGCACTTCACGCAACTCATTTTAGAGATTTGGAATGGAGGTTTGATGTACAGCTAGCGAGTCGATCGCTTCGTCGTCAACTAACTCCTTTGGTCAAATTCAAGTTAACTTTA---GACAGGGACGGAGAGAGGAAAGAT------------------GTCATCTTGCAAACTGACCCTACGAACCTGGTTCACATGACTAACGTTTTGGAACAGGCTTTAAAAGAAGCCCAGTCCCAGCATGCACGGCGGCTTATGAGGCACATC [594]

Sitalc ATGTTCTTGATTTTAGAAAATGATAGTAAAGAGCACCTTTCGTTTCTGTCAAACGTGGAACCAGATGTTGCAAAGGGTTTGTGCAGAACAGCAATGGAATTTGTTCTGAAAGGCACCAACCACAAGTTTTACAGTATTTCTGCACAAGCAAATGGTGTGGATGCTTCTATTGTTAAAGACTGTGTGGAGGCCCTGATGTATCTTCTAGCAGAGAGCTCTAAAATGAACTTGAGCCGTTTGGAGTTGGAAGACTCCCTCCTGAGCCTGGGTCTGGCACCTGACGTGAAAGAAGAGCTGGCTTCTATCTACCTGCAGAACTCTGCTGAACTCAGACAGGTGATGACAGAGTCTCCTTCAGCCATTCCTCACCTCACCAATATTCAGTGGAGGCTAGATGTTCAGTTGGGCAGCAGAAGCCTTCATCAGGAGACCAGCCCCCTGGTCCTCTTACAGCTGAGCCTTTCAGACCGGCAG---AGCCCT------------------------CTGACCCTGCAGGCTGACCCCTCCAATCTACAGCACATGCTGGTAGTGCTAGAAGACGCCCTGGCGCAGGCAAAGTCGCAGCACACCAGGAGGACACTTAGGGGGCTC [588]

Sclero ATGTTACTTATCTTAGAATCTGGTAGCAAGGAGAAATTGTCTATTCTTTCCCACGTTGAGTCAGATGTTGCAAAAGAATATTGTCAAACTGCCAGAGAATTTATTTTGAAAGGAGCCAATCAGAAAATATACTCTGTTATTGCACAAAAATCTGGAGTTGAAATCTCTGCAGTAAAAGACTGTTTGGAAGCTCTCATGTACCTATTATCAGAAAGTGCCAAACACAACCTGAGTCAATTAGAACTCGAAGATTCCCTGGTGAGCCTTGGCTTGCCAGCCTTTGTCAAAGAGCACCTGAGTGCTACCCATAGCGAATCATGCACAGAACTTAGGAGGGTTTTGGTGCAGTCTCAATGTGGAGTACCACATTTCCTGGACTTGCAGTGGAGGCTAGATGTCCAGCTTGGCAGTCGAGTCCTTCATCGGCAAACAAGCCCTTTAGTGGTACTGCAGCTAAGCTTATCCGAC---CAGAAGCGACCC------------------------CTGACCCTGCAGACAGATCCTACCAACCTTCAGCACATGCTGACCGTCCTGGAGCAAGCTCTGGCGGAAGCAAAGTCTCAACACACTCGAAGAGCTCTCAGAGGACTC [588]

Trogulus ATGTTACTTATCTTAGACGATGAACATAAGCAACATCTTTCGTTTCTCACCAATGGTAATCCCGCCTTTTGCAAAGATTTCTGTATGCTCTCAACTGATTTCTTGAGCAAAGGAATAAATTACAAAACATTTATAACAGCATCACAACGCTTAGGAGTTGAGGTTACTGTTGTGAAAAATGCTGTCCTTGCTGTTATGCATCTGCTCACCCAAGCTATCCAACTTCGTCTAAGTGAATTGGAGCTTCAAGATTCTCTGATCAGCCTTGATTTTAATGAAGCTTCTCAGAAAGAGCTAATAAGCTGGTATACAGATAACAAACAGGGCTTGAGAAATGCTTTGGACAAAGAGCTTCTGGATCCACCAAGGTTTAAAGACCTAGATTGGCGATTGGATATCCAACTGGCGTCTCGGTGCCTGCGCCATCAAGCAACTCCTATATTTGAACTTCAACTTAGACTGCAGGACAGCTTAAAATCAGAAGAAACTTTGAACAACGAACATAGCCTGCGACTGCAGACTGATCCTTCTAATCTAATTCATATGACTCAAGTTCTAGAACAAGCATTAAAGGAAGCTAAATCTCAATTCACAGGACGAATTGCTAGAAACCTC [615]

Ortho ATGTTGCTTGTATTAGATGAAGATCATAAACAACATCTTTCGTTTCTCACAAGTGGCAATCCACCATATTGCAAAGATTTCTGCATACTGTCAACAGAGTTTCTAATAAAAGGGATCAATTGGAAAGCATTCACAACTGCATCACAAAAACTGGGTGTGGAAATTGCTATAGTCCGAAATGCAGTCCTGGCTGTCATGCAACTCTTGACTGAAAGTGTTAGGCTTCGTCTTAGCGAATCAGAGCTTCAAGACTCCCTGATTAGCTTAGGTTTTGATGAAGAATCCTGGAAGGAACTAGTCGATTGGTATATGGCTAACATGCAAACTTTGTGGCAAATTATGAACAAGACAATGCTCGATTCCCCGAGGTTCAAAGATTTGGATTGGAGATTTACAGTCCAACTTGCGTCCCGTTGCTTGCGCCAACAAGTAACGCCTCTCTTTGAACTCAATCTAACTCTTCAAAATGAACTCCAATCTGAAGGAGAAACTTGCCATTCACAC---ATCAGACTACAAACTGATCCTACAAATTTAGTCCACATGACCCAGGTCCTAGAACAAGCCCTTCAAGCAGCAAAATCTCAGCACACGCAAAAGACTACTAGAAGTCTA [612]

Hespero ATGCTTTTGGTATTAGACGACGATCACAAACAGCATATTGCATTCATAACCGATGGAAATCCAACTGTTTGTAAAGACTTTTGCACTTTATCCACCGAATTTCTGCTGAAGGGAATAAACCAAAAATTATTCAATACAGCAGCTCAAAAATTAGGTGTCGAAGTTAACATTGTCCAAAATGCAGTATTGGGAGTTATGCATATTTTAACAGAAAGTGGCAGAGCTATGTTGAATGAGTCTGAATTTTCTGACACTTTATTGAGTCTGGGTTTCGATGAAGTTTCCCGTCAATTCCTATCTGTATGGTATAATGAAAATCGACAGAATCTTCGAAGTATCTTGAATAAACCCTCCATCAATAGTCCACGTTATAAAGACATAGATTGGAGATTAGAAGTTCAATTGGCCAGTCGCAGTTTAAGACATCAAACAAGTCCACTTATTCAATTGGACTTGACCTTG------GAGGACGACAACGAGGCCAAAGGTCGTCGG---------GTTCATTTGCAGACTGATCCCACTAATTTGTTGCATGTCACTAAAGTCCTAGAGGAGGCCCTGAAAGAGGCAAAGTCCCAACACTCATCTAGAATTGCTAGAAATTTG [600]

Leiobunum ATGATTCTCATATTAGACGACGACCAAAAAAAGCATATTTCGTTCGTAACGGAATCAAATCCGGAAATATGCGAAGATTTCTGTTTACTTTCAATCGATTTCTTAACAAATGGAACCAATCCTAAAGCTTTCAGTAGTGCTGCTCAGAAATTGGGCAGTGATGTTGAAACTGTGAGAAATGCAGTTATGGGTATTATGAAACTGTTGATTGAAAGTGCAAAAGCTATGTTAACGGATACAGAATTTGAAGATTCCCTCTTAGCTCTGGGTTTTCCTAATACCTCTAAAGAGAAGCTTAGTAAATGGTTTTCGGAAAATATTACATTTCTACGTAACATTCAAGCTCAAAAAACTATTGGATCAACAGAATTTTTAGATTTTGATTGGAGATTTGAAGTCCAAGTTTCTAGTCGTTTTCTACGCCATCAAGTAACTCCACTTTTTCAGATGCAACTAACATTGAGGGAAAATTCGGATAATGAGCACTCCATAAATAAA---------ATAAGGCTACAAACAGATCCTGTTAATTTACTACACATGACCCAAGTACTGGAGCAAGCTTTGATACAAGCAAAATCTCAACATTCAAGATCAGTCATGAGACATATC [606]

Proto ???ATTTTAATATTAGACGATGAACAAAAGAAGCATATTTCGTTCTTAACGGAATCAAATCCGGAAATATGCGAAGACTTTTGTTTACTATCGATTGATTTCTTAACAAATGGAACAAATCCTAAAGCTTTCATTAAAGCTGCGCAGAAATTGGGTACCGATGTTGAAACTGTGAGAAATGCAGTTATGGGTATTATGAAACTGTTGATTGAAAGCGCTAAATCTATGCTAACGGATACGGAATTTGAAGATTCCCTCTTGGCCTTGGGTTTTCCTTCTACTTCTAAAGAGAAGCTCAGTAAATGGTTTTCAGAAAATAGGACATTTCTTCGTAATATTCAAGGTCAAAAAACTATTGGGTCAACAGAATTTGTAGATATTGATTGGAGATTTGAAGCTCAAGTTGCCAGTCGTTTTCTACGTCACCAAGTAACTCCACTCTTTCAGATGCAATTAACATTGAGAGAAAATTGTGATAATGACAGCTCCATCAATAAA---------ATAAGGCTTCAAACAGACCCTGTCAATTTGTTACACATGACTCAAGTACTTGAGCAAGCTTTGACAGAAGCAAAATCTCAACATTCAAGATCAATTGTGAGACATATC [606]

[TICKFAST_NCHAR=375;U11/U12 small nuclear ribonucleoprotein 25 kDa protein]

Ixodes GATCCTCTCCTGAGCAACCTGCATCCGGAGGTGACCGTGGAAGAGCTGAGGTCGTACCTGGCGCTCGAGCACGGCCAAGCCATGTCGCTCAGGGTGCTGAGGGCAGACGGCGAT------------CCCTACACGGTCGTGGTCGAGCAGAAGGCCACAATCCTCGACCTCAAGAAAGCGCTGCAGAGGCACGCCACCCTTCGCATGGCAAGAAAAGGTGTGAAGAGAGTGGTGAGCTGGAGGTACATTTGGAAAACCTACTGGCTGTCCTTCGAAGGTCAACCCCTGAATCAGGACAGGACGCTGCTGCGAGACACCGGGATCCGTAACAATAGCGAGCTGTGCTTCATCAAGCGACGCCGG---GAAAGATAG [360]

Siro GATCCGCTTTTGAATTATCTGCCGACCTGCGTGACGCTAGAGGAACTAAATTCGTTACTGGCGTTAGAACACGGTCGAGCCATGACAGTTAACGTTGGACGAGCGGACGGCGAA------------TGGTACAGCGTTGTGGTGGAACAGAAAGCAACAGTTTTGGATTTGAAAAAAGCTGTTCGCTATAATTTCACCATGAAACAGAAACGAGCCGGCATCAGCAGGATTATCAACTGGAAATACGTTTGGAAACGTTACTGGTTTTATTTCGAAGGTCAGAAGCTGACGCCAGACAAAGCTGTTTTGAAAGATTTTGGAATTAGAAATAATTGTGAAGTGACTTTTATGAAAAGATTACGA---GCGAGATAG [360]

Sitalc GACCCACTTCTGAACTACCTACCAGCCAATGTTACAAATGAAGAACTAAGCTCTCTACTGGCATTAGAAGACGGACGAGCGTGGAGTTTGTACGTGTATAGGGGACAGAATAACGCAGATTTAGAACCGTACAGAGTAGTGGTAGAACAGAACGCGACTGTTTTTGATTTAAAGAGAGCACTGCAATATGTGGTTAAAATGAAGATGTCCCGTGAAGGGTCCGAAAGGCTGATTACATGGCGTCATGTCTGGAAGAGTTATTGGTTGTATTTCGAGGGGCAGAAGTTGATGCCCGATAGTGCTACTTTGAACTCCTTTGGTATTAGCAATAATGCTAAAATTTCATTTATTAAACGACTTACT---GTCAGGTAA [372]

Sclero GATCCTTTGCTGAGTTACCTGCCCACGAAAGTGACGCTACAAGAACTGCAGTCGTTGTTGGCTCTCGAGCATGGCAGAGCGATGAGCATTTATGTTTGTAGGGCTCACGGCAAAGGGACCAACGATCCGTATCAGGTTTTGGTCGAGCAAGATGCCACTGTTATGGTCTTAAAGAAAGCGCTGCAAGATGTGATAACTTTGAAGCAGTCTCGTGAAGGTGGCAATCGGTTAATTAGTTGGCGCTACATTTGGAAAAGCTACTGGCTGTATTTTGAAGGACAAAAACTCGCCCCAGATAGTGCCGCTTTAAAGGACTTTGGCATAACCAACAATTCATACATTACATTTATTAAACGCCTTACA---CAGAGATAG [372]

Trogulus GACCCGTTACTTAGTTATCTTCCACCGAATGCAACTTTAGAAGAACTAAATTCCTTACTAGCTCTTGAACATGGCCGTGCTATGAATATTTTCATTCATAGAGGGGATGGTGAA------------AATTATTCTGTTGTGGTAGATCAAGATGCCACGGTGTTAGGTCTCAAGAAAGCAATTCAGTATCATTTCACAATGAAACAATCAAGAGAAGGCAGCAAACGCCTCATTAGTTGGAAATATGTTTGGAAAAGTTATTGGCTCTATTTTGAAGGACAGAAGTTAACTCCGGATCATGCTAAGTTGAAGGACTTTGGAATAAGAAATAATGCAGACTTAACTTTCATAAAAAAACTGAGG---ATGCGCTGA [360]

Ortho GACCCACTGCTTAGTTATCTTCCGCCAAATGCTACACTAGAAGAGCTAAACTCCTTATTGGCACTAGAACATGGCCGTGCAATGAACCTTTTGGTACACAGAGCTGATGGAGAG------------ATCTATTCTGTTGTAGTTGATCAGGAAGCCACAGTTATGGGTCTTAAGAAAGCTATACAATATCATTATACTATGAAACAATCTAGAGAAGGAAGCAAAAGACTGATTAGTTGGAAATATGTTTGGAAGAGCTATTGGCTATACTTTGAAGGACAGAAGTTAACACCAGATCAATCAAAATTAAAAGACTTTGGCATTCGAAACAATTCAAATTTGACTTTCATAAAAAAACTCAGATCTTCTAATTAG [363]

Hespero GACCCATTGTTAAGTTACTTGCCCCGTAGTGTCACTTTGGAAGAATTAAACTCAATGCTCGCTTTAGAACACGGACAGGCTATTAATGTGTTTGTGCATAGAGAGGACGGAGAA------------CAATATTCTATTGTTGTAGAACAAAACGCTAATGTTTTGACACTTAAAAAAGCAATTCGATATCATTTTACTATGAAGCAGGCTCGAGAAGGCAATAAACGACTGATTAGTTGGAAGAGTGTTTGGAAAAGCTATTGGTTGTATTTTGAGGGGCAAAAATTAACACCGGATTCGGCTAAATTGACCGATTTCGGCATAAGGAATAATGCCACTTTGACGTTTTTGAAAAGATTAAGAGCCCCTAATTAA [363]

Leiobunum GATCCGTTATTGAACTCATTACCTACCAGTGCAACATTGGAAGAATTGAACTCCTTATTAGCTTTAGAACATGGACGTGCTATGAGTGTAACTGTGTGGCGTGCAGATAATGAT------------TATTACTCAGTTGTTGTTGATCAGGATGCTACAGTTCTAGATTTAAAAAAAGGAATACGCTATAACTTCACGATGAAACAGAAAAGAAAGGGCATTAGGCAATTAATAAGTTGGAAATATGTTTGGAAAAAATTTTGGTTGTATTTTGATGGCCAAAAATTAACCCCCGACCATTGCAAACTAAAAGAATTTGGAATACGCAATGGTTCAGAATTAACATTTATGAAAAGATTGAGAGCAAAAAATTAA [363]

Proto GACCCGTTATTGAAATCCTTACCAACGAGTGCTACGCTGGAAGAATTGAACTCTCTGTTAGCTTTAGAACATGGACGTGCCATGAGCGTAACTGTGTGGCGTGCTGATAACGAT------------TATTATTCCGTGGTTGTTGATCAAGATGCGACGGTTTTAGATTTAAAGAAAGGAATACGCTATAATTTTACTATGAAGCAGAAAAGAGAAGGAATTAGACAATTAATTAGTTGGAAATATGTTTGGAAAAAATATTGGATTTATTTTGAAGGACAAAAATTGACCCCTGATCATTGCAAACTAAAAGATTTTGGGATTCGAAATGGTGCCGAGTTGACATTCATGAAAAGATTACGAGCAAAGAACTAA [363]

[TICKFAST_NCHAR=405;XM_002434569 hypothetical protein]

Ixodes CTCAGCAGACCCGCCTCAGAGTTCGGCAATGACCCCACCGTGGAATCGCTGTGGGCCATCAAGGCCGTAGAGCACATGGAAGTATACTTCAACCTCATCAGCGCTGTCGATCCAAAACTCCTCAAGTTGACCCCGCACGACGACGCGCTCTACAAGGACTTCAGAGCCCGATTCCCAGCGCTGAACGTGAAGCTACTCGTCGAAGCCGAGCTGAAAAGCGAAGATTCAAAGAAACAGTGGCGGGAATTCTGCATGAACTACGAGGGAGTCGTGGAAGACTATAACTACGCCACCATGGTCCGACTGGACAGCGAGAGCGACTACAGGGAAGACAACACGACCCTCGTGCCCCGCGTCCAGTTCTACGCCATCGAGATCGCGCGCAACCGGGAGGGACACAACGAC [405]

Siro ???????????????????????????????????????????????????????????????????????????????????????TTCAATTTGCTGCGCTCGGTCGATCCGCTTCATTTGAAGTTGACGCGGTACGACGACGAGATCTACGGTAAATTTCGTGAAGTATTTCCCGACCTCAAGGTCGACAAGATTCAGGAAGTCGAGATGAAGAGTGCGGAAGCCAAAGAGAAATGGAGACCGTTTTGCGATGAGTTCAAGGAC---ATTGACGACTACAATTTTGGAACAATGCTCAGGATAGACAGCAGTCAAGAATATGCAGATGAAAATACACTTCTTGTACCGAGGATCCAGTTTTACGCAATTGAGATCGCAAGGAACAGAGAAGGGTTCAACAAT [402]

Sitalc TTAAATAGGCCTGCAGAAGAGTTCGAAAATAATCCTGAAATAGAAACAGCATGGGTGATGAAAGCTGTCAAGCATATGGAAATTCATTTCAATATCCTGTGCTCCGTGAATCCCAAGTTCTTAAGACTAACAAGATTTGATGATGACATCTACGCTGAGTTTCGGAAGGATTTTCCTGATGTCTGCGTAAGCACTGTCGACGTTGAGGAGATGAAAGGAGACGACGGGAAAGAAAAATGGAGAGTGTTTTGCCAAAAATTTGAGCAT---GTTCCAGACTACAATTTCGGCACTTTGCTGAGGATAAACTGTGAAAATGAATACTGTGAAGAGAACACTTTTTTTGTACCCAGAATCCAGTTCTACGCCATCGAAGTTGCCCGAAACAAAGAAGGCTTCAATGAC [402]

Sclero TTAAATCGTCCCGCTGAAGAATTCGTAAACGATCCAAGTATTGAAACCGCTTGGGTGATGAAAGCAGTTAAACACATGGAGGTTCATTTTAATATTCTTTGTTCGGTTGATCCCAAACATTTACGGTTAACAAAATTTGATGAAGAGATTTATTCGGAGTTTCGTCATGATTTCCCGGATATTGAATTAAACCACATCAATGTAGAGGCTATGAAAAATGGGGAAGGAAAAGAGAAATGGAGAGAGTTTTGTCAAAAATTTGAACAA---GTGCCCGATTTCAATTTTGGTACATTGTTAAGAATATCAAGTGATGATGACTATAACAATGAAAACACTGTTTTTGTTCCAAGGATCCAATTCTATGCCATTGAAGTTGCCCGAAACAAAGAAGGATGCAATGAC [402]

Trogulus TTATGCAAACCAGCGGAAGAGTTTACAAATAATTCTGAAATTGAAACAGCGTGGGTCATAAAGGCATCTCAGCACATGGAAGTTTATTACAATTTAATTACTTCAGCGGATCCAAAACTACTTCAGCTCACTAAACACGACTCGGACATTTACAAAGAGTTTAGAAGCCATTTTCCAGACTTTAAAGTTGACTTTGTAGAAGAAGAGACCTTAAAGTCCAAGGAAGCCAAAGAGAAATGGAGAGACTACTGTGAAAAATTTAAACAC---ATGGATGATTACAATTATGGAACCATATTACGGCTCAACAGTTCCGGAGAATATAACAACGAAAACACTATATTTGTTCCCAGAATTCAATTCTACGCTATTGAAATAGCGCGAAATAAAGAAGGGCACAATGAC [402]

Ortho ?????????????????????????????????????????????????????????????????????????????????????????????TTGATAACATCCGCTGATCCCGGACTTTTGCGTCTCACCAAATATGACTCAGATATTTATGATGAATTCAGAAAGGATTTTCCGGATCTCCAAGTGGATTTAATAGCCGAGGAAACTTTAAAATCAAAGGACGCTAAGGAGAAATGGAGAAACTATTGCGAGAAATTCAAACAT---GTTGATGATTACAACTACGGAACAATTCTACGCCTCAACAGCTCCGGCGAATATACCAACGAAAACACAACTTTTGTTCCAAGGATACAGTTCTATGCAATCGAAATTGCACGTAACAAAGAAGGTTGCAATGAC [402]

Hespero TTATCAAAACCAGCCGATGAATTCGATAATGATCCCGAAGTGGAGATGGCGTGGGTTATGAACGCCACTCAACACATGGAGGTGTATTTTAACTTAATCACGTCTGTGGATCCTAAACTATTAAAACTGACAAAATTCGACGACGAAATTTATTCTAAATTTAAAATCGATTTTCCCGATTTCGATGTTCGATTAATCGACGAATCGAATTTGAAAAATGCTGAATCTAAAAAGAGATGGCGTTCGTTTTGTGAGGAATTTAAACAC---GTTGACGATTTTAATTTCGGCACAATCATCAGATTGAACAGTTCGGGAAAATACGAGGAAAATAATACGACGTTCGTTCCTCGTATTCAATTTTACGCTATCGAAATTGCCAGAAATCGAGAAGGACACAATGAT [402]

Leiobunum TTATCTAGACCAGCTGAAGAATTTGGAAATGACGCTGATATAGAAATGGCTTGGGTTATGAAAGCAACTCAACACATGGAAATTTATTTCAACCTTATCACATCTGTTGATCCTCAAATACTTAAACTAACGCCATATGATGACGATATTTACAAAGAATTTAGGAATGATTTCCCCGACTTAAGTATCAAAAGCTTAGATACAGAAAGTTTGAAAAGCAATGAAGCAAAGGAGAAGTGGAGACCTTTTTGTGAAAAGTTTAACCAC---GTTGAAGATTTTAACTTTGGAACAATTTTAAGGCTTGATTCTGAGGGAGAGTACAGTGATTCAAACTCAATTTTTGTTCCTCGCATACAATTTTACGCCATAGAAATTGCCAGGAATAGAGAAGGCTTCAATGAC [402]

Proto TTATCTAGGCCGGCTGATGAATTTGGAAATGATTCCGATATAGAATTGGCTTGGGTGATCAAAGCCACTCAACATATGGAAATTTATTTCAATCTTATAACATCTGTTGACCCTCGAATACTTAAACTAACAAGATACGATGAAGACATATATAAAGAATTTAGGAATGATTTTCCGGACTTGAATATCAAATGTTTAGACACAGAAAATTTGAAAAGCAATGAAGCGAAAGAGAAGTGGAGACCATTTTGCGAAAAGTTTAATCAT---GTTGAGGATTTTAACTTTGGAACAATTTTAAGGCTGGATTCTGAAGGGGAATACAGTGATTCAAATTCTATTTTTGTTCCTCGAGTACAGTTTTATGCCATTGAAATTGCCAGAAATAGAGAAGGCTTCAATGAT [402]

[TICKFAST_NCHAR=450;actin-binding protein Sla2]

Ixodes ATGTCGAAAGTTCATCGGACCATTATTCACGACGTCGCGGACATTGCGGGTCTAACCGCTTTCTCCTTTGGCGAAGAGGAGGTGGACCGGTACGTCATGGTATTCAAGAAGGAGTTTGCGCCCTTGGACGATGAACTGGCGGCCTACCGCAAAGGGGAAGAGTGGGACCCGGAAAAGGCAAAGGCAGTCGCCCGGCAAAAGGAGCTCGCCGCCGCAGCCTTAGCAGAGCAAGAC---------------AAGGAATCGAGCAGGGGGAGCACTCCCCAGACGGACTACCACGAGAAGTACGAGAAGTTACTAGGTCGGGAGTCTGGCAAGGACGCTGCGAAGATCACCACTCCCAACAAGCAGTTCGGCTTTGTGCCGAGCGAAAACAAGAGGGACAAGCGGACGGTAGAGCAAGCCCTTGCCGACATCCGTGCCAAGAAG---CAGAAG [432]

Siro ATGAACAAAGTTAAGCGTTCAATCATTCACGATGCATCGGACATCGCCGGTTTGTCCGCGTTTTCTTTTGGCGAAGAGGACGTCGACCGACACGTCATGCTTTTTAAAAAGGAATTTGCACCGTGCAACGACGAACTTTCTGCGTACAGAAGAGGCGAAAGTTGGGATCCCGAAAAAGCCAAAGAAATCGCCAGGCAAAAAGAA---GAAACCGAAGACGAAAACAGAACGCAGTCC------AGCAGCTCTAAAAAGGAGTCTATGAAGATAAGCGCCAATCACTACAAAGAGAAGTACGAAAAACTGTTAGGGAAAGACGCTGGAAAGAGCGCCGCTAAAGTCACAACACCCAACAAACAGTTTGGCTATGTTCCTAGCGAGAACAAGAAAGACTTACGATCCATCGAACAGACTTTAGCCGATCTCAAAGCAAGGAAAAAACAGAAA [441]

Sitalc ATGGACAAGATCCATCGATCTATTGTTCACGACGTGGCAGAGGTAGCTGGTCTAACGGGATTCTCGTTTGGTGAAGAAGATGTCGACAGGTACATCATGCTGTTTAAAAAGGACTACATCACAACCGATGAAGAGCTGCAGGCTTATCGAAAGGGGGAAGAATGGACTGAAGAGAAAGCTAAAAACGTTTTGAGGCTCAAGGCCCTGTCTCAGGAGCAGAAGGCAGAAGAGACGATCGTAAGGAGAGGAAAAGGACGTGAAGCTGAG---ACGAGCAACGAAGTGTACAAGAAGAAGTACGAGAAGCTTCTTGGTACTGACGCGGGCAAAGACGCTGCGAAGATAACCACACCTAACAAGCAGTTTGGATTTGTGCCAAGTGAAAACAAACGAGATCAACGATCCATCGAGCAAACGTTAGCTGACATCAGAGCCAAGAAACGGCAAAAG [447]

Sclero ATGAATAAAATACAACGGTCAATCATTCATGATGTAGCGGATGTCGCTGGGGTAACAGGGTTTTCTTTTGGAGAAGAAGACTTGGACAGACATGTTATGCTTTTTAAAAAGGACTATATAACAACAGATGAAGAGCTCATGGCATATCGCAGAGGAGAAGAATGGAATGAGGAAAAGGCGAAGGAAGTTATAAGGCTAAAGGAGTCCCTTGCCAAGGATCGTTTGTTGGAAGAGGAAGCCGAGAAAAAAAAAGGCAAGCAGACCGAGGAACCGGGAAGCTTGGTTTACAAGAAGAAATATGAAAAACTTTTGGGCCGAGATGCGGGGAAAGATGCAGCTAAAGTTGCAACACCCAATAAACAATTTGGGTTTGTACCAAGTGAAAACAAAAGAGATCAACGTTCTATTGAACAAACATTAGCAGACATAAGGGAGAAGAAACGTCAAAAA [450]

Trogulus ATGAATAAAATGATGAGGTCAATCGTTCACGACATTGCTGAAGTGGCTGGATTGACTGCTTTCTCATTCGGAGAAGAAGATGTCGACCGTCACGTTATGCTCTTCAAAAAAGAATTTGCTCCATCTGACGACGAGTTGCTGGCTTACAGGAAAGGAGAAAAATGGGACCCAGAAAAAGCCAAGGAATTGAGCATTCAAAGGGAA---TTAGCGCGGAAG---TTGGAAGAGAAAGAA------------------ACAGTTGAGGAAATAATCCCCGGTGTAAATTATAAAGAGAAATACGAGAAAATTTTAGGCAAAGATGCTGGAAAAGAAGCTGCGAAAGTAGCTACACCAAACAAACAGTTTGGAAATGTTCCGAGTGCCAACAAGAGAGACCAAAGGTCGATTGAACAAACCCTAGCTGATATCAAAGCAAAAAAGAAACTAAAA [426]

Ortho GTGAACAAAATGAAAAGGTCTATTATTCATGATGTAGCTGAAGTTGCTGGACTTACAGCTTTTTCATTTGGTGAAGAAGATGTCGATAGGCATGTTATGCTTTTTAAAAAAGAGTTTTCACCATCGGACGATGAGCTACAAGCTTACAGGAAAGGGGAAAACTGGGATCCAGAGAAAGCAAAAGAATTGAATTTGCAAAAGGAA---TTAGCCCGGAAA---CTGGAAGAGGAAGAC---------AGCAAACTAAAGTCCAGTGAAATCATTCAAGGTTTCAATTACAAAGAAAAATATGAAAAGATTCTTGGAAAAGATGCTGGGAAGGATGCAGCTAAAGTTGCAACACCTAATAAACAATTCGGAAACGTTCCAAGTGCAAACAAGAGGGATCAGAGATCTATTGAACAGACCCTTGCAGATATCAAGGCCAAAAAGAAGCAGAAA [435]

Hespero ATGTCAAAGATGAAACGATCAATTATTCACGAAATAGCGGAGATTGCCGGTCTGACTGGTTTTTCATTCGGAGAAGAGGATATAGACCGTTACGTGATGCTATTCAAGAAAGAAGCTGCCCCTTGCGATGACGAATTAGCTTCGTATAGGAAAGGTCAAGAATGGAGTCAAGAGAAAGGCAAAGAATTGATTAAACAACGAGAA---TTGGCTAAACAA---ATCGAATTAGAGGAATTG------AACGCTAAGCCGGAGGCTGAGCTGATACCCAATAGCAATTATAAAGAGAAATACGAAAAATTGATCGGAAAAGACGCGGGCAAAGACGCGGCTATAGTCGCTACACCTAACAAACAGTTTGGAAATGTTCCTAGCGCTAATAAAAGAGATCAGCGTTCAATTGAGCAAACACTCGCTGATATCAGAACAAAGAAAAAACAGAAA [438]

Leiobunum ATGCATAAAGTGAAGAGATCAATAATTCACGACGTTGCGGAAGTGGCGGGTTTAACATCTTTCTCGTTCGGAGAGGAAGACATAGATAGACATATCATGCTCTTTAAAAAAGAGTTTGCTCCTTGTGATGACGAATTACAAGCTTATAGAAAAGGGGAAACGTGGAATGCCGAGAAAGCCAAAGAAGTAAATCGTCAGAAAGAG---TTGGCAAAGAAA---TTAAAAGAAGAAGAA---------GAAAGGTTACAACCAAATGAAGTAACGCCCAACAGTAATTATAAAGAGAAATATGAAAAGTTATTGGGAAAAGATGCGGGTATAGATGCGGCTAAGGTTGCTAAAGTTAACGTACAGTTTGGAAACGTGCCTAGTGCTAATAAAAGGGATCAAAGATCCATCGAACAAACACTGGCTGACATTAGAGCTAAAAAGAAGCAGAAA [435]

Proto ATGCATAAAGTCAAGAGATCCATAATTCACGATGTTGCGGAAGTAGCGGGTTTAACATCTTTCTCATTTGGAGAGGAAGATATAGATAGACATATCATGCTTTTTAAAAAAGAGTTTGCGCCCTGTGACGACGAATTACAAGCTTATAGAAAAGGCCAAACGTGGGATGCGGACAAAGCCAAAGAAGTTAATCGGCAAAAAGAA---TTAGCGAAGATA---TTAAAAGAAGAAGAA---------GATAGGCTACAACCAAACGAAGTAACGCCGAACAGCAATTATAAAGAAAAATATGAAAAGTTATTGGGAAAAGACGCGGGCAAAGACGCAGCTAGGGTTGCCAAGGTTAACGTACAATTTGGAAACGTGCCTAGCGCAAATAAGAGGGATCAGAGGTCTATTGAACAAACACTTGCTGATATTAAAGCTAAAAAGAAGCAGAAA [435]

[TICKFAST_NCHAR=387;adenine phosphoribosyltransferase]

Ixodes CCCAGAGTGGAAGATATACGCCGTGCGATACGCTCATTCAAAGATTTCCCCAAGAAAGGTATCCTCTTTCGGGATGTACTTCCCATATTCCACAATCCGGACGTTTTTCGTAAGTTGATCGACGTTCTCGCCGAGCAAGTCCGAAGCTCCGTGCCAGATGTGTCGGCAGTTGTGGGCATTGAGGCTCGAGGCTTCATCATTGCACCTGCTCTCGCACTGGCTCTCAACGTGGCTTTCGTGCCTGTGCGCAAACCTGGCAAGCTCCCCGGCACGGTGAAGAGCCAGGCCTACACGTTGGAATATGGTGAAGACAAACTGGAAGTCCAGGAAGACGGCTTTCTCAAGGGTCGCAAGGTTGTCATTGTTGACGATCTGTTAGCCACTGGA [387]

Siro AGTAAAATAGAAGCAATTAAAAAATGCATCCAATCGTATCCCGGATTTCCGAAGGACGACATCGTGTTCCGAGACATAATGCCAATAATGAGGAACCCCGACGTGTTTGCAGATATGATTTCCCTATTTGTTGAATACATACAAAAGAACGCCCCAAACACGGCTGGATTGGCTGGCTTAGAAGCCAGAGGATTTCTCATTGGTCCAACAGTGGCTTTGCGTCTTGGTGTACCATTTATTCCCGTTCGTAAGGCCGGAAAGTTACCTGGCGAAGTTAAGCAAGCCAGCTACATACTAGAGTATGGACAGGCAACAATCGAGATCCAGGCTGCGGCAGTTCAACCGAAACAGCAAATCGTAATTGTTGATGATTTAATGGCTACAGGA [387]

Sitalc AGCCGAATCGAGAATATTAAGAATTATATACAATCATACCCTGATTTTCCAAAACCGGGTATAATATTCAGAGATGTGTTTCCCATTTTCCTAAACCCAAGAGTGTCGGACGACTTGGTAACCCTATTATGCGATCACATCATCCAGCACGAAGATGGAGTAGCGGCAGTGGCTGGTTTAGAGGCCCGGGGTTTTGTCTTGGGCCCCCTGGTGGCTGCCAAACTTCATCTGCCTTTCTTACCTGTTCGGAAAGCTGGCAAGTTACCTGGGGAGTTGGTCAAGAAATCATTTACTCTGGAGTACGGGCAGGCTGCATTTGAAGTTCAAAAAAATTCTGTTACTGCTGGACAAAAAATAGTAATCGTGGATGACCTCTTAGCTACAGGC [387]

Sclero AGCCGAATAGAAAACATCAAGAACTACATAAGTTCATATCCTGATTTCCCTAAACCAGGTATTCTGTTTAGAGATTGTTTCCCTATTTTCCGTAACCCGACCGTGTTGGATGATGTCATAGCCTTGTTCTGCGACCACATTGTCTCTCTGGAAGGGGGCATATCTGGAGTGGTAGGTTTGGAGGCCAGAGGCTTTCTATTAGGTCCTCTTATTTCCCAGAAGTTACGCCTGCCCTTCATACCTGTCCGTAAGGCAGGAAAGTTACCTGGGGAAGTAGTCAAGAAAAGTTTTCAGTTGGAGTATGGTCAGGCAGAATTTGAAATTCAAAAGGATTCTGTAGTTGCTGGTCAGAAACTCTTCATTGTTGATGACCTTCTAGCAACAGGA [387]

Trogulus GAACGATTAAAAAACATTAAAAATTGTATAACATCTTATCCAAATTTCCCCAAGGATGGTATCGTTTATAGAGATTTCCTGCCTATTTTACGAAACCCTTCAGTTTTGGAAGACATGATTTCAATATTTTGTGACATTATAAAGACAAATTATGATCAAACAAGTTTTCTTTTTGCACTTGAGTCCCGAGGTTTTTTGCTGGGTCCTCTGATTGCAGCGAAACTGAAGTTGCCCTTTATTCCCATAAGAAAGAGTGGGAAATTACCCGGAGATGTTAAGAAAATGAGTTATTCTTTAGAATACGGCAGGGATTCTTTGGAAATACAAGTTGGCTGTGTTAAAAAGGGAGATTCAGTTGTGATTGTAGATGACCTTTTAGCAACAGGA [387]

Ortho GAAAGATTGAAAAACATCAAAAACTGTATAGTATCATATCCTAATTTCCCCATAGATGGAATCATATTTAGAGATTTTTTTCCAATTTTCCGGAACCCATCTGTTTTGGAGGACATGATTTCAATCTTCTGTGACATAATAAAAACGAACTATGAGCCAGTCAGTCATATTTTTGGATTGGAAGCACGTGGATTCTTGCTTGGTCCTCTGATCTCCACCAAACTAAAATTACCATTTGTTCCTATCCGAAAGAGTGGAGCACTGCCGGGGGAAGTCAAGAACATAAATTACTCCCTTGAATATGGCAGCAATTCCATTGAAATTCAAGTTGGCTGTGTTAAAAAAGGAGACTGTGTAGTTCTTGTGGATGACCTTCTTGCAACCGGA [387]

Hespero GATCGGATTAAAAATATAAAAAATTGCATAGTATCGTATCCTAATTTTCCAAAGGATGGGATTATTTTCAGAGATTTCCTGCCCGTTTTGAGGAACCCGGGTGTCGTCGAAGATCTGATAACGATATTTATTGAATATATTCATAAAAGTCACGTGGGGATGACGTGCATTGTCGGACTAGAATCTCGAGGCTTTCTAATTGGTCCACTATTAGCCTTGCAATTGAGATTACCATTTATTCCTTTAAGGAAATGTGGAAAATTACCAGGGGAAGTCAAGCAAATGAGCTATTCGTTAGAGTATGGCACTAATGCTTTTGAGGTCCAATCGGAGGCTTTTAAAATAGACGACAAGGTCATAATTATAGATGATCTTTTAGCTACAGGA [387]

Leiobunum AATCGCGTGAAAAATATTAAGAATTGCGTAACTTCTCATCCGGATTTCCCTAAGGAGGGAATTATATTTAGGGACATACTTCCTGTTTTAAGGAATGTCTCTGCGTTTGAAGACTTAATTAGTTTAATGTGTGACAGAATTAAATCGAATGGGGATCAAGTCGATGGTATTATTGGTTTAGAGTCCAGAGGGTTTCTTTTAGGAACACCAATTGCTTTGAGACTTAAAGTGCCGTTCATAGCTATTCGCAAGAGTGGAAAATTACCGGGGAAAGTAAAACAAGTCAAATATGCCTTAGAATATGGAACTAGCTCTTTCGAAGTTCAAGAGGATTCAATTCTACCGAATAAGAAATATGTGATTGTAGATGATTTGCTGGCAACTGGC [387]

Proto ???????????????????????????????????????????????????AAAGAGGGGATAATTTTTAGAGACATATTCCCAGTTTTAAGAAATGTGTCGGCTTTCGAGGATTTAATTAGCTTGATGTGTGACAAAATTAAATCACATGGGGATCAAGTTGATGGTATAATCGGTTTAGAGTCCAGAGGATTTCTCTTAGGAACACCTATAGCTTTGAGACTTAAAGTGCCGTTTATTGCAATTCGCAAGAGCGGGAAATTACCTGGGAAAGTCCAACAAGTGAAATATGCCTTAGAATATGGATCCAGTTCTTTTGAAATACAAGAAGATTCGATCCTCCCAACTAAGAAATATGTGATCGTAGATGATTTACTTGCCACTGGG [387]

[TICKFAST_NCHAR=813;alpha-1,6-mannosyl-glycoprotein beta-1,2-N-acetylglucosaminyltransferase]

Ixodes ATGGGGGGGCTGACACCACGGTGGCTG---CGAAGGACTCCCGGGGCCTCTGCGGCGGTCAAGTGCCTGGCGCTCCTCTCCTTCGGCTCCTTCGTCTGGCTCCAGACGCGCGTGTCCGACCTCGGGGGTGGA------------------------------GCCGGCGTTGTTGAGGACGGCTCCGCCTCCCTCGGAAACCAGTCGTGGGCGGGGCTACTCCAGAAC---------GCCAGCCGGGAGCAGGAGATCCTGCGGGAGTACGTCGGACAGCTCAACGATGCGCAGTTTGTGCACAACCTGGACGAGTATGGGCCCCTGCTGCCCGGGGACCCCGTGGTGGTGGTGCAAGTGCACAACAGGTGGCACTACCTGTTGTCACTGCTCGACTCCCTGCGCGGCGCCGCGGGCATCAACCGGACGCTCCTCATCTTCAGCCACGACGAGCACAGCCCAGTTGTCGAGAGCCTGCCCATCAAAGCCAACTTCTGCAAGATGATGCAGATCTTCTATCCGCGTGCCATCCAGCTCTACCCCCTCGAGTTTCCCGGGACCGACCCGAAGGATTGTCCTCGCAACATTGGCAAACAGGAGGCTAGGAAGCTTGGCTGCAACAACGCCGAACATCCGGACTCCTTTGGCCATTATCGGGAAGCCAAGTTCTCTCAGACCAAACATCACTGGTGGTGGAAGATGAACCGCATCATGGATGGGCTCAACATTACGCGTAACCACTACGGGCCGTTCATTCTGCTCGAAGAGGACCACTATGTGGCGCCGGACTTCTTGCACGTGCTCCGCCTCATG [771]

Siro ATGGGGGTACTGTGTGGATGGTGCACTGCCCTGCTCCATCGCCATTTTGCGCAGATCGTCAAGAGCATCTTCTTGCTGTTTGTTGTTTCTTTTCTCTGGCTACAACTACGAGTCGACAGCATCAAACCGGACTTCCTCAGCCAGAGAGTCAAAGATGCGGCTGACTCCGAGGTGGCCACTTTTCTGGCCAACTTCAGTCGCTACATTGCCCATCACAATGGAACTAGT---------ACCCTACCCCCAATCCCAGAGCTGTGGTCTCTAGTCAGGAAGCTCAATAGGAAGCAGCAAGTCCGGAACGTTGAGCGTTTCGGACCTCTGCAACCCAATGACACTGTTATCGTGATCCAGGTCCACAACCGGCTTCAGTACTTGTACGCCCTAATAGAATCCATCAAGAAAATGGCCCGCGTCAACGAAACGCTACTTATTTTTAGTCACGATCTGTTTGATTTACAGATTAATCAACTTGTTCAACAGATTGATTTTTGTAAGGTTTTGCAGATTTTTTATCCTTATTCAATTCAGCTTTATCCAAACAGTTTTCCAGGAACCGACCCCAAAGATTGTCCCAGAGACATAAAGAAGCCTGCTGCCCTGAAGCTTGGCTGTATAAATGCCAAATCTCCCGATTCCTATGGTCACTACAGGGAAGCACGCTTTTCTCAAACTAAACACCACTGGTGGTGGAAGTTAAATCATATCATGGATCGAGTGCAGATGACTCGTAATCATCCGGGTCAGTTCTTATTCCTAGAAGAAGATCACTACGTTGCTCCCGACATCCTTCACGTTTTGGACCGAATG [804]

Sitalc ???????????????????????????????????????CGGCGGTGGGCCTCTCTGGCCAAGACTGCTCTCCTCCTCTTCGCTGCCTCGTTCATCTGGCTGCAGCTGCGCGTCTCCACCATCTCGCAGGAGTTCCTGAGCAGAAAAGGT---------GGAGACACGGAGGAGGCAGGATTCTTGGCCAATTTTAGCAGCTATTCTGGGTTTCAGCAAAACGTGACCAATGGCCAGCTGATGTCTTCCCCGTACCACTTGCGCAGGCTGGTACGCAACTTAAACAAGGAGGCCTATATTCTTAACAGGGAAAAATATGGTCCTCTTCGAGAAAATGACCCAGTCATCGTAGTTCAAGTCCACAATCGCGTTCAGTACTTGTATGCTCTCATTGAATCATTACGCAAAACAAGAGGAATTAATCGCACACTACTGGTTTTTAGTCACGACTTGTATAACCAGAACATCAATGAACTTGTCAAAGAAATTGAATTTTGTAAGGTTTTGCAGATATTTTACCCATACTCTATTCAGTTGTATCCAAAAGAATTTCCAGGGGATGATCCCAATGACTGTCCGCGGGACATGAAGAAGAAAGAGGCTCTGAAAAAAGATTGCAACAATGCAGCATATCCAGATTCGTATGGACACTACAGAGAAGCTAGGTTTACTCAAACTAAGCACCACTGGTGGTGGAAACTTAACCATATCATGGACCGCGTTGGGGCCACCAAACTGCACCAGGGACCCTTTATTTTTCTGGAAGAGGATCACTACGTGTCCCCGGACGTTCTGCATTTTTTAAACCTAATG [804]

Sclero ATGGGTCCCTGGGATTGGTGTCACGTG---CTGCTGAACCGGCGATGGACCTCATTGGCCAAGACGGCCCTCTTGCTTTTTGCTGCTTCCTTCGTCTGGCTTCAATTAAGGGTCGCTTCCATTTCCCAGGAATTTCTCACTCACAAAGCT---------CCAGATGCGGAGGACGTGGGATTCTTCGCCAATTTCAGCAGCTATTCCGGTCGCCAACAGAATGCAAGCAATTGGCAGATTGCGTCTTCCCCGGTTCATTTGCGGAAGTTGGTTCAAAGGCTCAATCAGGAGGCCTTTATAGCAAATAGAGAAAAGTTTGGCTCCTTACGAGAACGAGACCCTGTTATTGTTGTTCAAGTGCACAACCGTGTTCAGTACCTATATGCGCTCATTGAATCCCTTCGTAAAGCCAGAGGTATCAACCGCACATTGCTCATTTTCAGCCACGATCTGTATAACCAAAACATCAATGATCTTATCAAAGAGATTGACTTCTGTAAGGTTTTGCAAATATTTTATCCGTACTCAATACAAATGTACCCAAAAGAATTTCCAGGAGAAGATCCAAATGACTGTCCCCGCGATGCCAAGAGACCAGAAGCACTTAAAATGAGATGCAACAATGCAGCTTATCCAGATTCCTACGGACACTATCGAGAAGCCAGGTTCACACAAACCAAGCACCATTGGTGGTGGAAGTTGAACCATATTATGGATGGCGTGAGTGCTACGAAAAACCACCAAGGTCCTTTCTTATTTCTGGAAGAGGATCACTATGTAGCTCCCGATGTACTGCACGTGCTGGCTCTCATG [801]

Trogulus ATGGTTCTCTGGCGGAACTGTATTGCC---TTTATGCATCGCCGATTTCCTCAGATTATTAAGTCAATCTTTCTTCTATTTACTCTTTCATTCCTCTGGCTACAATTACGAGTTGCCAATATCACAACCGACTTTTTTGTCAAGCACAGTCCATTCGCTTCAGACAATGAGGTTGCCATTTTCCTAGCTAATTTCAGCACTTATTCTGCTAATTCTCAGGTGACCAAAAATATCACATTCCAAGTGTCACCTTATCTTCTGCGGAGAATAGTGAAACAAGCTAATGAAGACCAAACGATACGAAATGCTGAAATCTTTGGTCCATTAGAAACTGATGAAGCTGTAATTATTGTTCAGGTCCACAACCGTGTCCAATATTTGTATGCCCTGATTGAATCACTCAGGAAAACTCAAAGAATTAACCACACTTTACTAGTCTTTAGCCATGACTTTTACGATCCCAACATCAATGCCTTAGTTAATGAAATAGATTTTTGTAAGGTTCTGCAAATTTTCTACCCATATTCAGTTCAACTTTATCCACATGAATATCCTGGAGAAGATCCGAATGACTGCCCTAGAGATATAAAAAAACTTCTAGCCGTGAAAAGAGGCTGCAATAATGCATTGTATCCTGATTTGTATGGACATTACAGAGAGGCCAAATTTTCTCAAACAAAGCATCACTGGTGGTGGAAGATAAATCATATCATGGATGGCGTGCAGGCTACGAAGAAGCACAAGGGTCCTTTTTTATTTTTAGAAGAAGATCATTATGTTGCTCCTGATGTTTTACATGTTTTGAAGTTAATG [810]

Ortho ATGGTTCTCTGGTGGCGATGTACGGCC---TTTATGCATCGCCGATTTCCCCAAATCATCAAATCAATTTTGCTCCTTTTTACTGTTTCTTTTCTGTGGCTGCAGTTAAGAGTTGCTGACATAACAACAGACTTTTTTACCAAGCACAGCCCATTCTCTCCTGACAATGATGTCGCAGTCTTCCTGGCCAATTTTAGCACTTACTCGGTTACTTCACACCAAACAAGAAATGCCACATTCCAGCCATCACCTTATCTAATGCGACGAATAGTGAAAAAACTTAATGAAGACCAGACCATTCGAAATGACGGAAGCTTTGGTCCACTGGAACCTAATGATGCTGTTATCATCGTTCAGGTCCATAATCGTGTCCAATACTTGTATGCTCTCATAGAATCTCTCAGGAAAACTCAAGGGATTAACCGCACACTGCTAGTCTTCAGCCATGACCTTTATGATCCAAATATTAATGCCTTAGTCAATGAAATAGACTTTTGTAAGGTTTTGCAAATATTTTATCCGTTCTCAATTCAACTTTATCCGCATGAATATCCTGGACAGGATCCTAACGATTGTCCTAGGGACCTAAAGAAAACATTAGCTATTAAACAAGGCTGCAACAACGCACTTTATCCTGATTTGTATGGACATTACAGGGAAGCCAAATTTACACAAACAAAGCACCATTGGTGGTGGAAGATAAATCATATCATGGATGGGGTTCAAGTTACGAAGAAACACCTGGGTCCGTTTTTATTTTTAGAAGAGGATCACTACGTTTCTCCTGACGTTTTGCATGTCTTAAAATTAATG [810]

Hespero ATGGTTCAATGGCGGCGTTTGGCTGTTCAGTTTCTCTACAGACGTTTCCCGCAAATTGTCAAATCTATTTTGCTATTGTTTATGGTTTCTTTTCTATGGCTGCAATTGCGAGTGGCAGACATCACCACAGATTTCTTGGCCAAACACAGTCCGTTCACGCCAGACACGGACGTAACAGACTTCTTGGCCAATTTCACTATTTATTCGGCCAATGTGGAT---GTCAAGAACGGAACATTCCACCCTTCACCGTTTCTACTTAGAAGAATTGTCAAAAAACTTAATACAGATCAGATTATTAGAAATGAGAAATTGTTCGGGCAATTGGGTCCGGATGATGCTGTCATAGTCGTCCAGGTTCACAATAGGGTTCAATATTTATACGCATTAATAGAGTCATTGAAGAAAGTGCGAGGAATTAATCATACGCTGCTAGTTTTCAGTCACGACCTTTACGACCCTAATATAAACGCGCTAGTCAATGATATCGACTTTTGTAAGGTTATGCAAATATTTTACCCGTTTTCGATTCAATTGTATCCTCGAGAGTATCCGGGTGAAGACCCTAACGACTGTCCACGAAATATAAAAAGACCCGAGGCTGAAAAACGTGGTTGTAACAACGCAAATTATCCCGATTTGTACGGTCACTATCGTGAAGCCAAATTCACCCAAACTAAACACCATTGGTGGTGGAAATTGAATCGAATTTTCGATAGAGTACAAGCCACTAAAGCACATCTGGGACCTTTCTTATTCCTAGAGGAGGATCACTATGTCTCGCCAGATGCGCTGCACGTATTAAGACTGATG [810]

Leiobunum ATGGGCTTGTTGGACTTGTGTGCCATA---TTGTTCCGTCGCAGAGCCTACCAAATTATTAAGACTGCGTTACTTTTGTTCATGGTGTCATTTTTATGGCTTCAACTGCGAGTGGCCAACATTGCCTCCGATTTCTTTGCCAAAGAAAATTCTATCACCTCTGAGGCGGAGGTAGCTGTCTTTCTGGCCAATTTTACCAGCTACTCTGCCAATTCCCTTTTAATAAGAAATGCCACACTCCACACTTCTCCTTTTCTTCTACGTCACGTTGTGCATAAAATAAATGCTAATCAAGAAGTTAGGAATGTAAAAATATTTGGAGATCTTCAACCAGATGACTTGGTTATTATTATACAGGTGCACAATCGAGTTCAGTATTTATATGCTCTTATAGAATCTCTCAGGAAAACGCAAGGCATCAATCAAACTTTGCTAGTCTTCAGTCACGATCTGTATGATAATAATATTAATAGTTTGATAAAAGAAATAGATTTTTGTAAAGTGATGCAAGTATTTTATCCATACTCTATTCAATTATATCCAAATGAATATCCTGGGCAAGATCCTAATGATTGCCCTAGAGATATTAAAAAAATAGATGCTATGCGTAAGGGATGCAATAACGCACGTTATCCTGATTCTTATGGCCACTATAGAGAAGCTAAATTTACTCAAACTAAGCATCATTGGTGGTGGAAAATTAATCATATCATGGATGGTGTAAAAGTTACCAAAGACCATCTAGGGCCATTTTTATTTCTTGAGGAAGATCATTATGTTTCACCTGATCTGATTCATGTATTAAAAGTAATG [810]

Proto ATGGGCTTGTTGGACTTGTGTGCCATA---TTGTTCCGTCGCAGAGCCTACCAAATTATTAAGACTGCGTTACTTTTGTTCATGGTATCTTTTTTATGGCTTCAACTGCGAGTGGCCAACATCGCCTCCGATTTCTTTGCCAAAGAAAACCCTATCGCCTCAGAGGCGGAGGTAGCCGTCTTCCTTGCCAATTTTACCAGCTACTCTGCCAATTCCCTTTTAATAAGAAACACCACACTTCACCCCTCTCCTTTTCTACTTCGTCGCATTGTGCATAAACTTAATACTGATCAAGAAATTAGAAATGTCAAGATATTTGGAGAACTGCAACCGGATGACTTGGTTATTATTATACAAGTGCACAATCGAGTTCAGTATTTATACGCTCTTATAGAATCTCTCAGGAAAACGCAAAGCATCAACCAAACTTTGCTAGTCTTCAGTCACGATCTATATGATAATAATATAAATAGTTTGATAAAAGAAATAGATTTTTGTAAAGTGATGCAAATATTTTATCCTTACTCTATTCAATTATATCCAAATGAATATCCTGGGCAGGATCCTAATGATTGCCCTAGAGATATTAAAAAACTAGATGCTATGCGTAAAGGATGCAACAATGCGCGTTATCCTGATTCTTACGGACATTATAGAGAAGCTAAATTTACTCAGACTAAACACCATTGGTGGTGGAAGATTAATCATATCATGGATGGTGTAAAAATTACCAAAGACCATTCAGGACCATTTTTATTTCTTGAAGAAGATCATTACGTTTCACCTGATCTGATTCATGTGTTGAAACTAATG [810]

[TICKFAST_NCHAR=774;amphiphysin]

Ixodes TCTAGGAATCCTCTGAAACGACTGTCGAAGCCATCGACTCGCACCTCTCCTGGAGGTGAACTGGACGAGAAGACTGTCGAAGAATATGTATCTAAATTTGTTCGCCTGGAGACTGATACAAAGAAACTGCACAAGGATGCCAAAAAGTATGAGGATGCCATGCTTGCATTGTACCACTGTGAGAAAAAGATGAGCAGCGACCTGGCCAACGGAGAGCTGTGCAATGACAACCCCGAGCTGCGTAGACTCGTGGAAGAGTGGCTAAGTTTTGCATCGTCCATGGAT---TCTGCTGTAGACGATCACCTGGTGGCGATTAGACGTTGCCTCGTAGACCCCCTCAAGAGGTACCAGACAGTCTTCACGGAAGTGCAGGCGGCGCTCAAGCGTCGCAACCAAGTGGCCCAGGAGTGCGTCCGACTGGAGCAGCGTGCGGAGCGACTGGCAGGCCGTGAAACAACTGGTCCCAACCTGGCCAAGCTGAGCGAGTGCAAGCAAGCCCTCGAGGCGGCACGGGGCGACTTGCGTACCCAGGGTGCACTGCTGGCCCAGGACCTGCCACGCTGGTACCAGGCAAGCAACCTCTACCTGCAGCCCTGCCTTGAAGCCCTGATGCACAGCCAGACCCTGCACTGGGGGCAGGCAACAACGTGCGCGCACAATCTGCTCGCCCCAGGT---------------------------GCGAAGGTGCCCGTGGAGCAGCGCCTGGCCGCTGTCAGGGCCCTCTCCATCGTGGCACTGCCGTCGTAA [744]

Siro ATGAGTTGGAATCCGCTACGCAAATTATCGAGACAAAATTCGAGACCTTTTCAGCAGCATCAAGGCGATTACGATCTAGAAAAATGCGTGACGAACTTGGAACAGTTGGAAGTGGCCACCAAAAGACTTCACAAAGAATCGAAGAAATACGAGGACCTTGTTTTGGAGATGAACCGCAACGAGCAGAAGATGACCACCGACTTGTCGGGTAGCTTCCTGTGCCAGGAGGAAGATGACTTACGGAATTTGGTCGAAGATTGGCATTCATTCACTTGCGGACTGACG---AAAGCAGACGTTGAACTGATAGCTATTTTACAGCAAGTTTTAACAGACCCCGTCAAGAAATTTCACGGAATTTTCCCCGATGTTCGATTAGCGCTTCGCAAAATCGAACAAGTTCAGCAAGAATGCGCCAAGTTGAGACAGAAAGTCAGCAAACTGGAAGAACGCGAAGGTACTGGAATGAATATCGTTAAATTAGAACACGCCAAACACTCGCTTTCAGTTTGTATAGGACACGAACAGAGTTTGCACGCGACTCTGAAAAAAGAACTTCCTCAACTTTTGGAAGCCCGGATTGAATATTTTCAGCCAGTTATTGAAGCCGTGATTCGTGCCGAAGTCGCTCATTGGGGTGAAACGGCTAAGGTGTTTAGTGAGACAGTTCATCAGGTG---CCAAAGAACTAT------GTGCCCTCTGATGTTGCCCAAAGGCAAAGGATCACAAAAATTCAGGCGCTTTCCATCGTAGCA---GGATTGTGA [759]

Sitalc ATGAGTTGGAATCCGCTG---AAAAAATTTGGTAAGCCCTTGAGGCACCCG------CACGCCGGCGACGGTGATCTTGAGAACTATGTAAACAAATTGGAACAGCTCGAAACCGCCACGCACAAGCTTTACAAGGATGCTAAAAAGTATGAAGAACTGGTGCTAGAAACGACCAAAAACGAACAGAAAATCACCAAAGATCTGTCTTGCAGCGTCTTGTGTCAAGAAGAGGATGGACTGAGGTCACTCGTTGAGGAGTGGTTTACGTTTACGAGTAGTCTGAGT---AAGCCTGACGAAGATCTGATCGCAAATCTTCACCAGTCTCTTGTGGAACCTGTTAAGAAATTTCAGGGATTTTTCCCTGAAGTGAAGTTTGCGCTTCGCAAGTACGAACAAGCTCAGCAGGAGTGCGTGAGGCTTAAAACTAAGGTGGCACGTTCGCAGCAGCGCGACAATACCGGTACCAATATGGTGAAGTTGGAAAATGCTAAGCATTCGTTAGCTGCTAGCTCTAATAATGCTCAGTGTGTGCATGCATCTTTGAAGGAGGAGTTGCCGCACGTTTATGAGAAACGAATCGAGTACTTTCAGCCGACGATTGAAGCGTTGATTCAAGCTGAAGTTAACCACTGGGGAGAACAGACTCACATTTTTGCAGACTCAATAACGCAGTTA---CCAAAACAATTT---------TCTGCTGATGAAATTCAACAGAAAAGGATCAGAGAAATTCAAGGGCTTTCTATTGTGGCT---GGTACGTAA [747]

Sclero ATGAGTTGGAATCCGCTG---AAGAAGTTTGGCAAGCCTATGAGGACTCTACAATCCAGTGCCGACGATGGCGACTTCGAAAGTCACATTACCAGGTTAGAGGAACTCGAAAATGCTACCAGGAAGTTATACAAGGATTCCAAAAAATACGAAGACCTGGTGTTAGAAACCAACCGGAACGAGCAGAAAATCACCAAAGATTTGTCCTGCAGTGTTCTCTGTCAAGAAGAAGAAACGCTACGGCTTTTGGTTGAAGAGTGGTTTACGTTTGCGAACGGCTTAAGC---AAACCCGATGAAGATCTCGTCGCAAACTTTCACCAGGCCATGGTTGAGCCCGTCAAAAAATTTCAGGCCTTTTTTCCAGAGGTCAAGTTTGCATTACGCAAGTACGAACAGGCACAGCAAGAATGTGTGAAGTTGAAGGCCAAAGTGACACGATTGGAGCAACGGGAAAGCACCGGAACAAATATGGTCAAGCTCGAGCATGCGAAACACTCATTGGCTGCCAGTATGATTAATGCGCAAAGTATTCATGCAACGCTCAAGCAAGAAATACCTCAACTTCATGAGAAGCGCATCGAGTACTTCCAGCCGATAATGGAAGCTGTTATTCAAGCCGAGGTGAACCATTGGGGTGAAATGACGCACCTCTTTGCGAATGTTATCAAGCAAGTT---CCCAAACAGCAT---------TCGGCGGATGACGTGCAACTTAAAAGACTGCAAGAAATTCAAGGGCTCTCGATTGTCGCT---ACAACATAA [753]

Trogulus ATGAGTTGGAATCCCTTG---AAAAAGTTTTCCTCGAAACCACGATCTTTTCAC---TTCAACTACGATGCCGATTTGGATCAGTGCATTTCCAAATTAGATCGATTAGAAACAGTGACAAGAAAGTTGCATAAAGATGCCAAAAAATATGAAGAACTGATAATAGAACTCGGAAGAAGCGAACAAAAGATGACTGAAGATTTATCCAGCAGTCTTCTATGTCAGGAAGAAGACAGACTGCGTAGCCTGGTAGAAGAATGGCATTCCTTTGCATGCGGTTTAAGC---CAATCGGATGCCAACCTTGTAGCTAACCTGCATCAAGTATTAACCGACCCAGTGAAAAGATTCTGCGGTCTTTTTCCTGAAGTTCAATCTGCTCTAAGAAAACACGAACAGGCTGTAAATGACTGCGCTAAACAACGAACTAAGTTGGAGAAGATCGGCACCAAAGAGGATGTAGCCAGTGTTTTGCTTAAACGCGATCATGCCAATCGTTTGCTTAACTCCAGCATTGCCAATGAAAAATCTATACACGGAGTTCTTAAAAAGGAATTACCCACGCTTTACGATCGCAGAGTTGAGTACTTCCAACCCACAATCGAAGCTCTCATCCTTTCGGAGGTCAATCACTGGGGAGAGATGACTAATATCTTTGGTGAATTAGTGCAGGATGCC---CCTAAACAATAT------TCAACTCTGGATAGTGCTCAGCGACAAAGGCTCGCCAAATTGCAGGGCCTTTCTATTGTTGCT---GGGTCTTGA [753]

Ortho ?????????????????????????????????AAGACTTCTCGATCTATGAAT---TCCTTTGATGACGTTGATTTAGATCAGTGTATTTTTAATTTAAAAGAACTGGAAATTGTAACACGAAGCCTTCACAAAGACGCCAAAAAGTATGAAGAACTGATTTTAGGATTGAGTAGGATTGAGCAAAAAATGACCGAAGACTTATCAAGCAGTCTCTTGTGCCAGGAAGAAGATAAACTGCGTTGCTTGGTTGAAGAATGGCATTCATTTGCATGTGGTTTAAGT---CGATCAGATGCGGATTTGGTAGCCAATCTACACCAAGTCTTGACAGACCCAATCAAACGCTATAGTGGCCTTTTCCCGGAAATTAAATTAGCCCTGAAGAAGCATGAACAAGCTGTACAAAACTGTGATAGTATGCGAACCAAATTACAAAAGATTGCCTGTAAAGAAGACACAGCTCCAAATTTGCTTAAACGTGAACAGGCAAATCATTCACTAAATGCTAGCACCATAAAGGCTAGATCAATGCTTGAAGGCTTAAAAAAAGATTTGCCAACATTGTATGATCGCAGGGTAGAGTATTTCCAGCCCACTATCGAGGCAGTTATTCAATCTGAAGTCAACCATTGGGGAGAAATGACAAACATTTTTGGGGAACTGGTACATGATGCA---CCCAAGCAGTAC------TCAGCTTTAGATATAGCACAACGTCAAAGAGTGGCTAAATTGAGAGCCCTATCTATTGTTTCA---GGGTCATAA [756]

Hespero GCCAGCTCCTTGATGAGT---TGGAATCCTTTGCGTAAATTGTCCAAAGGGGGCGGTGGGAGTGACGATCGTGACGTCACCCGCAGCGTCAAAGACTTGGAACGCCTGGAGGCGGTGACGAGGCGACTGCACAAAGACAGCAAGAAATACGAAGAGTGCGTCTTGGAGCTGAGCAAGAGCGAGTGCAAAATGACCGAAGACTTGTCGAGCAGCGCCCTCTGCAGAGACGAAGAGCGACTGCGCATGCTCGTCGAAGAGTGGCACAGTTTC---TGCAAGGGGTGCAAGGACACAGACGACGAACTCGTGTCCAACGTGCACCAGTTGATGACGTTGCCTTGCAAGCAATTGCAGGCGGTGTTCCCGGAAGTGAGGCTGGCCCTGAGGAAGTTGGAGCAGGCCACCAACGACACGGCCAAGTTTCGGCTCAAAGTGAACCGGCTGCAAGGCAAAGAGGCGACGGCGGCCAACTTGGTCAAAATGGACCAGGCCAAACACGCGTGCGTCGTTAGCAGCAACGCAGAGTGCAACGCCAGAGACGTTCTCAAGCGGGAGTTGCCCACCCTGTACGAAAGGAGGGTCGAGTACTTCCAGCCTTGCCTCGAAGGGGTCATCAGGTCAGAGGTCAACCATTGGGGGGAGACCACTCGCATATTCAACCATTTGCTGGTCGACATT---CCGAAGCACAAC------GGGCAACTCGACGCCAGGATGAAGGCCAGAATGGACAAAATGCAAAGCTTGTCCATTGTT??????????????? [759]

Leiobunum ATGAGCTGGAATCCGCTC---AAAAAATTCGCTAATAAACCCCAATCGCGAATTTCCACCTACAATGAGGATGGTTTGGAAAATTACGTAAGCAAATTAGAAAAACTAGAAGCAGACGCACAAAAATTGCATAAAAATTCGAAGAAATACGAAGAGTGCATTTTAGAAATGAGCAAAAGTGAGAAAAAAATGACCGAAGACCTATCAAACAGTGTATTGTGCCAAGAAGAGGAGCAATTGCGTACTCTAGTTGAAGAGTGGCACTCTTTCGCATGCGGATTAGCG---AAAGCAGATGAGGAATTAGTAGCTAATTTGCATTACACATTAACAGATCCGGTAAAAAGATTCAAATTAATATTTCCCGAGGTAAAATTTGCATTGAGAAAATACGAACAGTTTAAACAAGATTGCGAAAAGTTACAGGCCAAAGTGGCAAGATTAGAAACTAAAGAAGGAACGGGTAATAATTTAGTTAAGTTAGATCACGCGAAACATTCCTTAAACTCTTGCGTGGCCAACAAACTGAACATGCAGAAATTGTTGAAACAAGAACTTCCTATTTTCTACGATAAAAGGATTGAATATTTCCAGCCGTGTATAGAAGCAGTTATTAGATCCGAAGTGGATCACTGCGGAAACGTTACAACGATTTTCAATAATGCTATTACCGAAAAACTACCGAAAAATTACAATGCCACAAGCACTGACATAAAACAGAGGGAAAGAATAGAACACATGCAGGCTTTGTCTATCGTT---------TCGTAA [759]

Proto ATGAGCTGGAACCCGCTG---AAAAAATTCGCTAATAAACCCCAATCACGGATTGCCACGTACAATGAAGATGGTTTGGAAAGCTACGTAAGCAAGCTGGAACAATTGGAAACAGACGCGCGAAAATTGCACAAAAATTGCAAGAAATACGAAGAATGCATTTTAGAAATGAGCAAAAGCGAGAAGAAAATGACAGAAGACCTATCAAACAGTATACTGTGTCAAGAAGAGGAGCAACTGCGTACTTTGGTTGAAGAATGGCACTCTTTCGCATGTGGGTTGACA---AAAGCGGACGAAGATTTGGTAGCGAATTTGCATTACACATTAACAGACCCAGTGAAAAGATTCAATGGAATTTTCCCGGAGATAAAATTTGCATTGAGAAAATACGAACAGTGTAAACAGGATTGCGAAAAACTACAGGTCAAAGTGGCCAGGTTAGAAACGAAAGAAGGGACTGGTAATAACCTAGTAAAGTTGGATCACGCGAAACATTCCTTAAACGCATGCGTAGCCAGCAAACTTAACATACAAAAGGTGCTGAAACAAGAACTGCCCATTTTCTACGATAATAGGATAGAGTATTTTCAGCCTTCTATAGAAGCTGTTATCAGAGCTGAAGTAGATCACTGGGGCAATGTTACGACAGTTTTCAACGATACTATTAACCAAGTA---CCTAAAAATTACAATGCATCGAGCACTGATATCAAGCAGAGACAACGAATAGAACGCATGCAGGCTCTGTCTATAGTTGCT---GGGTCATAG [762]

[TICKFAST_NCHAR=1650;amyloid binding protein]

Ixodes ATGGCGTCCCGCCTGGAGTGGATTCCAGATTCTCTGTACAACTCCGCCGTTACGACGCTGGTCAACAATTACCTCATCTTCCAGAAAGACCTGAAATACCTCCCCGAGAGCATCACCTTCGACATTTTTTATCAGCTCTACAAGCAAAACAAACTATGCTTCCTAGGCTTCGAATTCTCCGATTTGGATGTGTTCGCCAAGATATCCAAGGTGAAGGACAAGAGGCACCTGCTGCACCACTGCTTCCAGGCTGTGATGGACCACGGCACCCAGACGGCCCACTCCCTGGCCGAGACGTACCGCCAGCGCTGCTACTCGGCCTCCAGCCTCCCCGCAGTGCACCACGACTCTTGCCGTATTAAGCCCCCGCTTCCTTTCTGTGCAGGAAATTTTCTCAGCGATGCCGGCTGGTTCCGTGAGAGTGAGAAGATGCTACTGAGTTGCCTGTCCCTCTGCCGCCAGATG---GACGACTACCCACACTGGGTCTCTGCCCTGGAGTGCTGCCTCAGGCTGCTTCACGTTCAGCACTCGTACTGCAAGTTCACGGAGGCCATGGAAACGTTGGAGGAGACGAAGCTGTACATTGTCAAGTTGACCAACATGGGCCACTCGCTCAACCTAGCTGGCCTCTACTCCGAGTTCTCCTCCCTCTACTCCTGCCGGAGCCAATACAAGGAGGCGTACGACTGGGCCATGCAAGCTTTGATGCAGCTCAACTCCAATGCCCACCCTAAGTCAATAATCGACGTCTTGAGGCAGGCCTCAAAAGCCTGCGTCGTGAAGAGGGAATTCAAGCAGGCAGAGCTCCTCATCAAGCAAGCCCTTCATATGGCATGGGAGCACTTTGGCAGGGAGCATCCAAAAACGGCAGACACATTGCTCGACTACGGCTTCTACCTGCTCAACACGGACTCTGTGAGCCAGTCGGTGCGGGTGTACAGGGTGACGGGTGCTCTCGAATTTGTCGATCTCGAGGAACCTTCATCTGACCCAAACAAAAGCCATCCTCGGCTTTTGTTTGGGTCAGATTTTCCAATTTTTGGTATTGGTACAACAAAGCAATGTGAAAGC------TTGCAGACCTTATACATAGCAATGCATGTACTAATGGGTGTCTGGCCCATGGTAATGCATTGTTTGAGAATCCCTGCCTTACAGTCTTGGTCTTTTCTGTGCAGAGACCATGCCGAAAAGGCCATGCTCATCATGACCCGCCTGTTGCCGGAGGATCACCTGCT---CTTGGCATCTTCAAAGAGGGCCTTCGGCGAGATGAACGTGCAGACGGCCAAACACTACGGCAACCTGGGCAGGCTGTACCAGTCCATGCGCCGCTTCAAGGAGGCGGAAGAGATGCACCTGAAGGCGATCGACATCAAGGAGCGCCTGCTGGGGCCCGAGGATTACGAGGTGGCCCTGTCGGTGGGGCACCTGGCCTCCCTGTACAACTACGACATGAAGCTGTACAACCAGGCCGAGCAGTTGTACCTGCGCTCCATAGCCATCGGCACCAAGCTGTTTGGGGAAGCGTACAGCGGCCTGGAGTACGACTACCGGGGCCTGCTGCGGGTCTCCATGGAGATGAGCAAGACGGACCAAGTTCTGCGCTACACGCGGGTGCTGGAGGAGTGGCGCCAGCTGCGC [1638]

Siro ATGGCGGCGACGTTACATTGGATTCCAGATACTCTCTATAATTCAGCCATTTCATCTGTTGTAACGAACTATACATATTTTCAAAAAGAGTTAGCAACACTTCCAGATAATGTATTATTCGACATCTGTTATAAGCTTTACAAGCAGGGTCACCTGTGTCAACTGGGCTTTGAATTTTGCCACTTGTCGGTCTTTATTCGAGTCCTCAAGGTCAAGGACAAAAGGCACTTGTTACACCATTGCTTTCAAGCATTAATGGACCATGGGGCCCATTCTTCCCATTTGTTGGCCAATGCTTTCAAACAACACTGCAATATTATTCACAGA---------TCATCTCCTCAAGTCAAGGAATCTGTCATCCAATGCGGTTTCTCGCTAGGTGGTTTTCTGTGCGATGCCGGTTGGTTTCCCGATTCGGAAACGGTTTTTCTGGCGTGCTTCAAACTCTGCAAGGAAATG---AGCATATCTCCCAACTGGGTTCTTAGTTTGGAATGTTGTCTCAGGCTTCTTCACGTTCGCACGGTGTTTTGCAAATTCCCAGAGGCGGAAGAAACGTTTCGCGAGGCGCAGACTTGCATCGAACACCTGAAAGGC---CAACCCTCGGTCAACCTGGCTGCCATCTATGGGGAGTTTATCGTACTATTCTTCTGCAGGGGGGAATATACAGTGGCTTACAACTGGAGCGTGCGAGCGCTAAAAGAATTGACCAATAATCTTTCGCCACGGATCCTTTTAGACGTTTTGCGACCTGCGGCAAAAGCGTGCGTCGTTAAGCGAGAATTTAAGCGAGCCGAATTGCTGATCAAGCAAGCTCTCCATATTTCCAGAGAACACTTTGGCATAAAACATCCTAAAATGGCCGAAACGCTTCTGGACTACGGTTTCTATCTTCTCAATGTCGACAGCATTGCGCAGTCCGTTCAAATCTATCAAAAGGCTCTCGATATTCGTAAGAGCATTTTTGGTGGAACCAATTTGTACGTGGCGTCCGCTCACGAAGATTTGGCCTACGCGTCTTACGTCCACGAGTACAGCTCGGGAAAGTTTCAGAAAGCCAAAGAGCATGCAGACAAAGCATTGAAGATCATGACTCAATTACTTCCAGAAGATCATTTATTGTTGGCATCTTCCAAACGTGTTAAAGCTTTAATTTTGGAAGAAATTGCAATCGACAATCACGACAAAAACACCGAATTGGAACTTCTTCGTAAAGCGCAAGAACTTCATCTGACCGCACTGCGATTTGCACAGCAGGCATTTGGGGAGCTTAATGTACAGACGGCCAAACATTATGGCAACCTGGGACGACTCTATCAGTCTATGCAACGCTTTCCTGAAGCTGAACAAATGCATTTGAAGGCCATCAAAATAAAGGAAGAACTATTGGGACCAGAAGATTACGAAGTGGCCCTTTCCGTGGGTCACTTGGCCTCCCTGTATAATTACGATCTGAACCATTTCGATAAAGCCGAACATCTTTACCTCAGGTCCATAGCTATTGGGATCAAGTTATTTGGCGAGAGTTACAGCGGATTGGAGTACGATTACCGAGGCCTTCTACGAGTGTATATGGTACAAGGGGACAGTGAAAAGACCGTGCAATACACGGAGATCTTGCGTCAGTGGAAACAGTTGCGA [1635]

Sitalc ATGGCATCCTCTCTACAGTGGATGCCAGATTCTTTGTATAATTCAGCACTTTCAGCAGTAGTCCAAAGATATTCTCTGTTCCAAAAAGAAATATGCAATTTTCCTGAAAATGTACTTTTCGACGTATGCTACAAGTTGTACAAAGAGAACCGGTTATGCCAGTTGGGATTTGAGTTTTGTCACATCAACATATTCTCAAGAGTGCTCAAAGTCAAGGACAAAAGGCACCTTCTGCACCACTGTTTCCAAGCATTGATGGATCATGGGGCCAGAACATCGGAGCTCCTGATGGAGTCATTCACTCAACACTGCAAAGACACACTTGCCAACACTTGTTCCTCTGTCCATGTCAAAGACCATGCCATCAAATTTGGAATCACACTTGCTGAGTTTCTGGTGGATGCTGGCTGGTTCCCAGACAGTGAGCAAGTAATGGCTGCATCTCTCAAACTTGCTGTCAGCGTA---GATGACCCTCCTCACTGGCAAATGGCTCTTGAGTGTTGCCTCAGGTTAATCCATGTGAGAACGGCTTTCTGTAAATTTCAAGAAGCCACCGAAACGTTTGAGTTGGCCCAGGAGTACATAGCTAAGCTGCAGCAAGTAAATATCCCTGTGAATATGGCTGGCATATACGGTGGATTTGCTGTTCTCTTTTTCAGCAGAAGTCAATATAATGAGGCATACAAATGGAGTCTGAAAGCTTTACAACACCTTTCATCTAGTCTTCCTTTGAAGGTTGTTGTGGATGTCTTAAGACATGCAGCGAGGGTCTGTGTAGTGAAAAGAGAATTCAAGCGAGCGGAACTTCTAATCAAGCAAGCTTTAACTATAGCTGTTGAAAACTTTGGAAGGCAAAATTCCAAAACTGCAGATACGCTGTTGGACTACGCTTTTTATCTGTTAAATGTAGATTCTATTGCACTGTCCGTCAAAACATATCAGTCTGCCCTGGATATTCGTCTTAGTATATTTGGAGGGCAAAACTTGAATGTTGCCATTGCTCATGAAGATCTGGCCTATGCACTTTACGTCCACGAATACAGTGTTGGAAAGTTCGATGACGCAAGGGACCACGCGAACAAAGCAATGAATATCATGAATCATCTACTACCCGAAGACCACTTGCTCCTAGCCTCTTCAAAAAGGGTCAAAGCCCTAATTCTTGAGGAGATAGCCATAGATTTCCATGACAAACAAGAGGAACGACGCCTTCTGGGAGAAGCACAAGAACTGCACCTGACTGCCTTGCACCTTGCCAAGAGAGCGTTTGGAGAAATGAATGTCCAGACGGCCAAGCACTACGGAAACCTGGGAAGGCTCTATCAGTCCATGCAGAGGTTTAAGGAAGCGGAAGAAATGCATTTAAAGGCCATTGAAATCAAGGAACAGCTACTTGGTCCAGATGACTACGAGGTTGCACTTTCTGTTGGGCACTTGGCCTCGCTTTACAACTACGACATGCTCGTTTTTGACCGAGCTGAAATGCTTTACTTGCAGTCTATCAGTATTGGCATCAAGTTGTTCGGAAAGGGCTACAGCGGCCTTGAATATGACTACAGGGGTTTGTTGAGGGTATACATGACACAAGGCAACCATGAAAAGGCACTTGAATACACCAATATCTTACAACAGTGGAAACAACTTCGA [1647]

Sclero ATGGCATCCTCTCTTCAGTGGATGCCTGATTCGTTATACAATTCAGCTCTTTCAGCAGTTGTTCAGAGATATTCCCTGTTTCAAAAGGAGATATCAAATTTCCCCGAGAATGTCCTATTTGACGTATGCTATAAGTTGTACAAGGAGAACAGACTTTGCCAACTTGGGTTTGAATTCTGTCACATCAACATCTTCTCGAGAGTCCTGAAAGTCAAGGATAAAAGACATCTCCTCCACCATTGTTTTCAAGCCCTGATGGATCATGGAGCGCGAACTTCAAATTTGCTGGTGGAATCCTTTACGCAGCATTGCAAGGATGTTGTGGTGAAT------GCCTCCACTCACGTCAAAGACCATGCCATCAAATTTGGACTCACTTTAGGTGAATTCCTAGTGGATGCAGGTTGGTTTCCAGATAGCGAAAAAGCTGTTGCGGCCTCTTTACAACTTTCCTTGAGTGTC---AACGATCCCCCACACTGGCAGATGACCCTGGAATGCTGCGTCAGACTGATCCATGTTAGAACAGCGTATTGCAAGTTTCAAGAAGCAAATGAAACTTTTGAACTTGCCCAGGAATACATTGTGAGACTTCAGCAGCAGAATGTTCCTGTTAACATGGCTGGCATTTACGGCAGTTTTGCAGTCCTGTTTTTTAGCCGGAGCCAATACAACGAGGCCTACAAATGGAGCTTGAAAGCTTTACAGCATCTCACATCTAATCTACCAATTAAGATAATTGTAGACGTTTTGAGACATGCAGCTCGAGTATGTGTGGTCAAACGGGAATTTAAGAGAGCAGAACTTCTAATCAAGCAAGCTCTGACAATTGCTGTTGAGAATTTTGGCAGACAAAACTCAAAAACTGCAGACACTCTGTTGGACTATGCCTTCTATTTGTTGAATATAGACTCCATAGCCCTTTCAGTCAAAACATATCAGTCTGCACTGGACATTCGCTTGTGTACTTTCGGAGGCTACAATCTTCATGTTGCTATTGCCCATGAGGACTTAGCCTATGCTTTATATGTCCATGAATACAGTGTCGGAAAATTTGATGATGCCCGTGAGCATGCAAATAAAGCTATGGAAATTATGAACAGGCTATTATCTGAAGATCATTTGCTGCTGGCTTCTTCTAAACGTGTGAAAGCCCTAATCCTGGAAGAGATAGCAATCGACAATCACGATAAACAAGAAGAACGTCGCCTGTTAGGAGAAGCGCAAGAGTTGCACCTGACAGCTCTTCTGTTAGCCAAGAGAGCTTTCGGGGAAATGAACGTCCAGACAGCTAAGCACTATGGCAATCTGGGAAGGCTCTATCAGTCCATGCAGAGATTCAAGGAAGCGGAAGAGATGCACTTGAAGGCCATTGAGATCAAGGAGCAGCTGTTGGGTCCAGACGACTATGAAGTAGCGCTCTCGGTCGGCCACTTGGCTTCTCTCTACAACTACGATATGCTCGTATTTGAGAAGGCTGAAAGTCTATACCTGCAATCCATCAGTATTGGTATCAAGTTATTTGGCAAAGGTTACAGTGGACTTGAATATGACTATCGTGGCCTTCTTCGTGTTTACATGACTTTGGGGAATCACGAGAAAGCCATAGAGTACACAAATATCCTTCAGCAGTGGAAACACCTGAGA [1641]

Trogulus TTAACGTATATTTTGCATTGGGCTCCAGACTCACTTTATAATATATCAATAACAGGGATAGTTAATAATTATTCCTTATTTCAAAGAGAATTGCGAACATTTCCAGAAAATGTACTTTTTGATATATTTTACAAACTTTATAAACAAGGTCGGCTATGCCAATTGGGATTTGAACTCTCTCACTTGGACGTCTTTTCAAGGGTTTTAAAAGTTCAAGACAAAAGGCACTTGCTTAACCATTGTGTTCAAGCTTTAATGGACCATGGCGCACGCACAACAGGCTTTCTCGCTGACTCTTTCAAACAACATTGTCGCATTATTAAAAGT---------TCAACAGTTGAGGCTAAGCTCCGAACTATTACATTTGGGCTTAATCTTGCGGAGTTCCTAGAAGAAGCTGGCTGGTTTGAAGACTGTGAAAAAGTGGCCTTAGCCTCGTTTACACTTTGTCAAACGTTG---CAAGACTCTAGTCACTTGAAGTTATCTATTAATTGCTGCATGAGGTTGCTTCATTCTCAAACATCATACTGCAAATTTGCTGAAGCTAGTGAAACTTTTACAAAGGCCCTAAAAATTGTTGAAGAAATTAAACTCTCAAATGCACCCGTTAATTTAGCTGGTATCTACGGAAGCTTTATGGCAATGTATTTTAGCAGAAGCGAATACAATGAGGCATACAAATGGAGTTTAGATACAATGAAGGAGCTCAAAAACAATTTGCCTAAAAAGGTCGTCATTGATATTCTGAGACAAGCAACAAGAGCATGTGTTGTGAAACGAGAGTTTAAACGAGCAGAACTGCTAATTAAACAAGCTTTACGGCTTACCAAAGAAAACTTTGGAGAAATACATCCTAAAACAGCTGACACTCTTTTAGATTATGGCTTTTATTTGCTGAATATTGATTCAATTGCTAAATCTGTTAGAGTTTATCAGACTGCATTGGACATTAGAACGACTTGCTTTGGCGGTCATAATTTGCATATTGCCATTGCACATGAAGACCTTGCTTACTCTCTTTATGTTCAAGAATACAGCTCTGGAAAATTCGAAGATGCTAGAACACATTCTGAAAGGGCTCTTCAAATAATGGGTTCTCTACTATCTGACAATCATCATTTAATGGCATCTTCAAAGAGGGTGAAAGCTTTGATTTTAGAAGAGATGGCACTTGACAATCACGATAAAGTTGCAGAAATGAAATTATTAATGGAATCGCAGGAACTACATCTTGCAGCTCTGCAGATGTCGCGTCAGGCATTTGGCGAGAAAAATGTTCAAACAGCAAAACATTATGGAAACCTTGGGAGGCTCTACCAGTCTATGCACCGCTTTAGTGAAGCTGAAGCAATGCATTTGAGAGCAATTGAAATTAAGGAAGAGATTCTAGGAAAAGAAGATTATGAAGTGGCTCTTTCTGTTGGCCACCTTGCTTCTCTATACAATTATGACATGCTCAACTTTCAGAAAGCTGAAAAGCTTTATATACAATCAATCGAAATTGGCATAAAGCTGTTTGGAGAAGGATACAGCGGTCTCGAATATGACTACAGAGGCCTGTTACGTGTCTATCTAACAATGGGAGATCATGATAAAGCCAATACGTATACACAAATCCTTCATACATGGAAACAGATTCGA [1638]

Ortho ATGGCTTACTCCCTGCATTGGATTCCAGATACCCTTTATAATACCGCTATATCAGCGGCAGTTTCTAATTATTGTTTGTTCCAGAGGGAATTAAGAACCTTCCCTGAAAATGTACTTTTTGATGTCTTTTACAAGCTCTACAAACAAGGCCGACTTTGCCAACTTGGGTTTGAACTGTCACATTTGGACATCTTTTCCAGAATCTTAAAAGTACAAGATAAGCGGCACTTATTGAATCATTGTGTCCAAGCCTTGATGGATCATGGGGCCCGTACAACAAGTTTCCTTGCTGATTCATTTAAGCAGCACTGTCACATCATCAGGGGC---------ACAAATGCAGAATCTAAACTTCGAACTATAAAGTTTGGAATTAGTCTTGCTGAGTTTCTAGAAGAGGCTGGCTGGTTTCAAGATAGCGAAAAAGTGGCATTGGGCTCGTTCACACTCTGCCAAAGTTTG---GAAGATCCTAGTCAACTAAAGCTTTCACTTGAATGCTGCATAAAGCTACTACATGCTCAAACCTCCTATTGCAAATTCTCTGAGGCTTATGAAACGTTTTTGGAGGCTTTAAAGATTGTTGAAAAAATCAAGAACTCAGATCTTCAAATAAATTTGGCTAACATATATGGCAGTTTCATGTCCATGTATTTTAGCAGAAGTGAATACAATGAGGCTTACAAATGGAGCCTGAAAACTTTGAAAGAATTGAAAACCAGTTTGCCTACAAGGGTTATTGTGGATGTTCTTCGACATGCGACGAGGGCATGTGTAGTGAAACGGGAATTCAAGCGAGCAGAAATGCTAATTAAACAAGCTTTGAGTCTTGCCAAAGGATGTTACGGCTGCAAACATCCTAAGACAGCTGACACTCTTCTAGACTATGGTTTCTATCTGTTGAATATTGATTCTATTGCTCAATCTGTAAAAATTTATCAGAATGCATTAGACATTCGCATGAGTTGCTTTGGTGGTCAAAACCTGCATATTGCCATTGCCCATGAGGACCTTGCTTACGCCCTTTATGTCCAAGAGTACAGTTCCGGCAAATTTGAAGATGCAAAGTCCCATTCTGAAAAGTCACTGCAAATTATGTCATTTTTGCTCTGCGAAAATCATCATTTATTGGCATCTTCAAAGAGAGTTAAAGCCTTAATATTAGAAGAAATGGCTCTAGATAATCATGACAAAATGGCGGAGCAACAGTTGCTCATGGAGGCACAGGAACTTCATTTAGCAGCCTTACAGATGTCTCGTCAGGCTTTTGGGGAGAAGAACGTTCAGACAGCTAAGCATTATGGAAATCTTGGACGACTGTATCAGTCTATGCATCGTTTCACTGAAGCTGAACAGATGCACCTGAAAGCAATTGAGATCAAGGAAGAGATACTAGGGAGTGAAGACTATGAAGTTGCTCTCTCTGTTGGTCACCTTGCATCTCTCTACAACTATGACATGCTAATATTCCAGAAAGCAGAGAAACTGTACCTGCAGTCTATTAATATCGGCATTAAACTCTTTGGGCATGGCTATAGTGGTCTCGAATACGACTACCGAGGACTGCTCCGTGTCTATGTGACGCTCGGCGATACAGACAAGGCCAACCAGTACACACAGATACTGCATGAGTGGAAACAGATTCGG [1638]

Hespero AGACTGATTTCGTTGCAATGGATTCCCGATACGTTGTATAATTCTGCAATTGCTTCTGTCGTTAACAATTATCCGTTGTTTCAAAGAGAAATTAGATCATTCCCCGAAAATGTACTTTTTGATATTTGTTACAAACTCTACAAACAAGGCCGTTTATGTCAGCTTGGATTCGAATTTTGTCATTTGGAGATTTTCTCAAGAATTTTAAAAGTTAAGGATAAAAGACATTTATTACATCACTGTGTCCAAGCCTTGATGGATCATGGAGCTCAAACGTCTCACTTATTGGCTGATTCATTTAAACAACATTGCCACATTATTCGTGGA---------TCACCTATTGAAGCAAAACACAGGACAATTATATTCGGTCTTAATTTAGCTGAATTTCTGGAAGAAGCTGGCTGGTTTTTAGACAGTGAAATAGTTGCAGATGCCTCTTTTACGCTTTGTAAAACATTTTGTGAAGAATTGAAACAATGGAGAGTCGCTTTGGATTGCTGTATTAGATTGTTGCATTCTCAGACTTTATATTGCAAATTTTCCGAAGCCAAAGAGACATTTGCTGAATCATTACTAATTCTGGAGAAGATAAACGAATCTGGACAAAATGTGAACTTGTCCAGTCTTTACGGTAGTTTTATGGCAATGTTTTTCAGTCGAAGCGAATATAACGAGGCTTACAAATGGAGTCTTAAAACCCTGAAAGAATTGTCCTCTAACCTACCTAAAAGTATTGTGATTGACATCTTGAGACACGCAGCCAGGGCTTGCGTTGTTAAAAGAGAATTTAAAAGAGCAGAAATGCTCATTAAACAAGCTTTAAGTCTTGCAAAAGATACTTTCGGAAACAAACATCCTAAAACAGCAGATACACTTTTAGATTACGGATTTTATTTGTTAAATATTGACTCTATTGCTCAGTCTGTGAAGATATATCAAAGTGCTTTAGATATTCGTTTAAGTGCTTTCGGAGGAAGAAATCTGCACGTTGCTATCGCTCACGAAGATCTCGCTTATGCTTTATATGTCCAGGAGTACAGTTCGGGGAAATTTGAAGATGCAAAATTACACGCCCAAAAGTCCTTGCAAATTATGGTCCAACTTTTGCCAGAAAACCACCTATTATTAGCTTCGTCCAAAAGAGTCAAAGCATTGATTTTGGAGGAAATTGCAATCGATAATCACGATAAATCTACTGAATTGCGTCTGTTAATGGAGGCTCAAGAACTTCATTTGGCCGCTTTACAAATGTCCAGACTCGCTTTTGGAGAAATCAACGTTCAAACGGCCAAACATTACGGAAATCTCGGAAGATTGTATCAGTCCATGAAACATTTTAAGGAAGCTGAACAAATGCACCTGAAAGCTATTGAAATCAAAGAACAAATATTGGGACCTGAAGATTACGAAGTTGCCCTCTCTGTTGGGCATCTCGCTTCTCTTTATAACTACGATATGCTCATTTTCGAAAAAGCTGAAAAACTTTACTTGCAATCAATTAATATTGGCATCAATTTATTTGGCAAAGGGTACAGTGGCCTGGAATACGACTACCGGGGTTTATTGCGTGTTTATCTCACACTAGGGGATCACGTGAAAGCGACAACTTACACCCAAATTTTACATGAATGGAAACAATTCCGA [1641]

Leiobunum ATGGCTGGAATTCTGCATTGGTTTCCAGATACATTGTACAATTCAGCCATAAGAGCAATTGTTGCTAATTACCATTGTTTTCAAAATGATTTAAAGACATTTCCAGAAAATGTTGTATTTGACGTGTGCTACAAGTTGTACAAAGAGAATAGATTATGCCAGCTGGGGTTTGAGTTTTGCCAACTTGATTTATTTGCTCGAATTTTGAAAGTAAAAGACAAAAGACACCTATTACATCACTGTGTACAAGCTTTAATGGATCATGGAGCTCATACTACCAAACTCCTCTCAGTTGCATTCAAACGGAATTGTTTTCAT------------------ATGGCTCATCTCTCTAGTGAAAAGACGATTAAGTTCGGCCTCTCATTGGCTGAATTTTTAGCCGAGGCTGGCTGGTTTGAAGATAGTGAAGAAGTAGCCATGGCAGCGTTAAACGTGTGCAAAACTAGA---AATGATATAGAACACCTGAAACTCAGCTTAGAATGCAACATAAAATTGCTTTATGCTAGAACACAGTTTTGCAAATTCCTCGAAGCAAACAAAACATTCAAGGACACAATTGAGGTGGTTGAAAAACTAAAAACATTTAATGTTCCTATAAATTATTCTTGTGTTTATGGAGCTTTTATGGGCATGTATTTCAGCCGAAGTGAATATAGTGAAGCTTACAAATGGAGTCTAAGAGCTTTGAAAGAACTTACATCCAATATACCTGCAAATCTCATTCTCGATATATTAAGGCAGGCTGCAAGAGCTTGTGTGGTGAAAAGAGAGTTCAAAAGAGCTGATTTGTTGATCAAACAAGCGTTAAGCATAGCGAAAGAAAATTTTGGTGATAAACATCCAAGAACAGCTGACACTATGTTAGATTATGGGTTTTATCTCTTAAATGTAGATTCGGTTGCTCAGTCAGTCAAAGTTTACCAAACTGCCCTAGACATTAGACAAACGGCTTTTGGAGGTCACAATTTACTCGTGGCTATTGCCCATGAAGACTTGGCCTATGCATTGTATGTCCAAGAATACAGCACTGGCAAATTTGAAGACGCGAGGGAACATGCCGAAAAAGCCCTTCAAATAATGAACCATTTACTACCGCCCGATCATTTGTTGTTAGCCTCTTCCAAGAGGGTTAAGGCTTTAGTCTTGGAAGAAATTGCCATTGATAATCAAGACAAAACGGCCGAACATCAGCTGCTAATGGAAGCGCAAGAACTGCATTTAGCTGCTTTGAAAATGACCAGACTGGCTTTTGGGGAAATGAATGTACAAACTGCAAAGCATTACGGAAACTTGGGAAGACTCTATCAGTCAATGAGAAGATACAGAGAAGCGGAGCAAATGCACCTTAAAGCTATCGAGATAAAAGAAGAAATTTTGGGTCCAGAAGATTACGAAGTTGCTCTCTCCGTGGGACATTTAGCGTCGTTGTACAATTACGATATGAAAATTTTTGATAAAGCCGAACATCTGTATTTGAAATCTATCAATATAGGTTTGAAACTATTCGGAGCTGGGTACAGTGGGTTGGAATATGACTACCGAGGTCTCTTGCGCGTATATCTAACCTTGGGAAATCATGAAAAATACATGGAATATAACAATATCTTGCGTGACTGGAAAACAACTAGG [1629]

Proto ATGGCTGGAATACTGCATTGGTTTCCAGATACATTGTATAATTCTTCTATCAGGGCCATTGTGGCTAACTATCATTGTTTCCAAAATGATTTAAAGACCTTTCCTGAAAATGTGGTATTTGACGTGTGCTACAAGTTGTACAAAGAGAACCGATTATGTCAGTTGGGGTTTGAGTTCTGCCAACTTGATCTATTTGCACGAATCTTAAAAGTTAAAGATAAAAGACACCTATTACATCACTGTGTACAAGCTTTAATGGATCATGGGGCTCATACAACCAAACTACTGTCAGTTGCTTTCAAACGGAATTGTTTTCAT------------------ATGGCTCATCTCTCTAGCGAAAAGACAATTAAGTTTGGCTTATCATTGGCTGAATTTTTAGCTGAGGCAGGCTGGTTTGAAGATAGCGAAGAAGTGGCTTTGTCTGCATTAAATGTGTGTAAAACTAGA---AATGATATAGAACACCTGAAACTCAGCTTAGAATGCAATATAAAATTGCTTTACGCAAGGACGCAGTTTTGCAAATTCAACGAAGCCAACAAAACCTTTAAGGATACAATTGATGTGGTTGAAAAGTTAAAGACATTTAATGTTCCTATAAATTACTCTTGTGTTTATGGAGCTTTCATGGGCATGTATTTTAGTCGAAGTGAATACAGTGAAGCATATAAGTGGAGTCTAAGAGCTTTAAAAGAACTTACATCCACCATACCAGCAAATCTCATTCTCGATATTCTAAGACAGGCTGCAAGAGCTTGTGTTGTGAAACGAGAATTCAAAAGAGCAGATTTGTTAATCAAACAAGCTCTAAGCATAACAAAAGAAAACTTTGGTGATAAACATCCAAGAACTGCAGACACAATGTTAGATTATGGCTTTTATCTGTTGAACGTAGATTCAGTTGCTCAGTCAGTCAAAGTTTACCAAACAGCACTAGACATTAGGCAAACGGCTTTCGGTGGTCACAATTTGCTTGTGGCAATCGCCCATGAAGATTTGGCCTACGCATTGTATGTCCAGGAATACAGTACCGGAAAATTTGAAGATGCGAGGGAACATGCTGAAAAAGCCTTACAAATTATGAATCATCTACTTCCACCTGATCATCTTTTATTAGCCTCTTCTAAGAGGGTCAAAGCTTTAGTGTTGGAGGAAATTGCCATTGATAACCAAGACAAAACTGCTGAACACCAGTTGTTGATGGAAGCGCAAGAACTACATTTGGCTGCTTTGAAAATGACCCGACTCGCTTTTGGGGAAATGAATGTTCAAACTGCGAAACATTATGGGAACTTAGGGAGATTGTATCAGTCAATGAGAAGATACAGAGAAGCTGAGCAAATGCACTTGAAAGCAATTGAGATAAAAGAAGAAATTTTGGGTCCGGAAGATTACGAAGTTGCTCTCTCAGTTGGTCATTTAGCTTCTCTTTACAATTATGATATGAAAATTTTTGATAAAGCAGAGCAGCTCTATTTAAAGTCAATCAATATAGGGTTGAAACTGTTCGGAGCTGGTTACAGTGGATTGGAATATGACTACCGTGGTCTATTACGTGTTTACCTGACCTTGGGAAATCATGAAAAATACATGGAGTATAACAATATCTTGCGTGATTGGAAAACAACACGC [1629]

[TICKFAST_NCHAR=300;armadillo repeat-containing protein]

Ixodes AAAGAGCAAGTTCTAGCCAATCTCGCCAACTTCGCCTACGATCCGGTGAACTACGAACATTTCAAACGACTGAACATCGTGAACCTTTTCATAGATCAGCTT---GACAGTAGTAGCGATGCTTTGAAAGAGTACGCCATCGGCGGTATCTGCAATCTGGCTTCAGATCAGTCATTTCGCGCTGAAGTTGTCAAGCCCGAACACATTGCAAAGGTGGTTGACTGCTTATCAAACTCGCAAGAGGAAACTGTGCTTTCGACCATTACTACACTGGTATACCTTGGATTGTCTGGTTGCAAA [297]

Siro AAAGAACAAATTCTCGCAAACTTGGCCAATTTTGCTTACGATCCAATCAACTATGACCATTTTCGAAAATTGAATATTATAACGCTTTTCTTAGAGCATGTG---GATGAAACGAATGAAAACATAGTTGAATATGCCGTGAGTGGAATTTGCAATTTGGTGTTGGATGAAAGTAATCGGCAATGGGTGCTAGCAAAAGGTGGAATCGCAAAAATTCTTCGCTGCTTGTCCAGCCCCAGGGAAGAAACAGTTTTATCGACTATCACGACCCTCATATTTATGGTCGCTCCCGATACAAAA [297]

Sitalc AAAGAACAAGTACTTGCCAATTTGAGTAATTTTGGCTATGACCCTATCAACTATGAACATTTTAGAAAACTTAACATCCTTGCTATATTTTGTGAACAACTG---AACAGCTCTTCAGAAAAATTGGTTGAATTTGCAGTTGCAGGACTCTGCAACTGTGCACTAGATCCTGAAAACAAGAACTATATTCTTAGAAGTGGTGGAGTGAACAAAATACTATGCTGCCTTACAACCAGCAATGAGGAGACGCTGTTGTCAGCTATCACAGCACTCATGTTTCTAGACGCCCCAGAATCGCAC [297]

Sclero AAAGAGCAAATCCTGGCGAATCTTGGGAATTTTGCCTACGATCCAATCAACTATGAGCATTTCAGGAAATTGAACATCATAAACTTATTTCTCGAACAAATG---AACAGTTCTTCTGAGCGTTTTGTGGAGTTTGCACTTGGAGGAATTTGCAACTGTGTTTTAGACCCCGAGACCCGGAAGGAAGTTCTCCAGGGCGGAGGACTTCAGAAAGTGCTCTGTTGCTTGTCATGCTCCAACGAGGAGGTCCTGCTGACTGCCATCTCCTGTCTCATGTTTCTACATACACCTGAATCTAGA [297]

Trogulus AAAGAACAGGTTTTGGCAAATTTGAGTAATTTTGCCTACGACCCAAAGAATTTTGAACATTTCAAAAAGCTTAACATATTAAATCTGTTTCTAGAACAGCTT---GATGATGAGTCCCAAATGTTGAGAGAGTTTGCAATTGGTGGAGTCTGCAATGCTGTCTTTGATTCAGAAAATGCTGCAACTATTATTAATCGCGGAGGACTACCAAAAATAATTAAATGCTTGTGCAGTCAAAGAGAAGAAACTTTGATATCAACATTGACAGCAATGATAATTTTATTCTCACCAGCCACGAAA [297]

Ortho AAAGAGCAAGTTTTAGCTAATCTTAGCAATTTTGCGTACGACCCAAGAAACTTTGACCATTTGCAAAACCTCAATGTGTTCAGCCTGTTTTTAGAGCAGCTT---GAGGGTGAATCAGAGGATCTCCGAGAATTTGCAGTTGGTGGAATCTGCAATGCTGTATTTGATCAAACTAATGCTGACATGATAATAAACCTTGGTGGAATACCAAAAATACTGTCATGTTTATGTAACGAAAGGGAGGAAACTTTAATATCAACAATGACTGTGATGATAACGTTATCTTCACCCACCACAAAA [297]

Hespero AAGGAGCAAATTTTGGCCAATTTGAGCAATTTCGCGTACGATCCCAAAAACTACGAGTCACTTAGGAAATTGAACGTTATTAGCTTGTTTTTAGCCCAATTG---CAAAGTGGTTCGGACAAGCTTATCGAATACGCCATTGGGGGACTTTGTAATGCAGTTTATGACCCTGAAAATGCAAAGGTTGTCAATAATTTAGGTGGCATATCTAAAATCATTCAATGTTTGAATAGCCAAAACGACGACATTTTATTATCGACTATGACTACGATCATAACACTATTCAATACAAATACTAAA [297]

Leiobunum AAAGAACAAGTTCTTGCAAATTTAAGCAATTTTGCTTATGACCCTACAAACTACAACTTTTTCCATGAATTAAATATTATATCACTATTTTTAGATCAAATAGATCAATCAACATCAGAAGTGTTATGTGAACACGCCTTAGGTGGCCTTTGTAATTTATCAGCAGATTCTGAAAATCGGGAAAAAATTATAAAACATGGTGGAATACCAAAAATTGTACAGTGTTTATCATCTCAGAGGGAAGAAACTATTATAAACACTATGACTACATTACTTCAACTAATAGATGGAACAACAGTT [300]

Proto AAAGAACAAGTCATTGCAAACTTAAGCAATTTTGCATATGACCCTACAAACTACAATTTTTTCCATGAACTTAATGTTATATCGCTGTTTTTAGATCAAATAGAACAATCAACATCAGACAATTTACGTGAATATGCCTTAGGTGGGCTGTGTAATTTATCAGCAGATTCAGAAAATCGGGAAAAAATTGTAAAATGTGGTGGAATACCAAAAATTGTGCAATGCTTATCATCTCAAAGGGAAGAAACTATAATAAACACTATGACTACTTTGCTGCAGCTAATAGATTCAACAACAGTT [300]

[TICKFAST_NCHAR=969;brain protein 2]

Ixodes ACAGCCACAGACGATGGACTGTCATTTCTAGATCAGCACAGGCAACTGCTCAAAGCTACGGCTTCCCTCGTA---CACGACCCGTGCCCTCAAGTGGTCTCAAAAACGTATGATTGTCTCGTAAATGCCACGTCACATCCCTCCATAGCAGCCTACAGCTTGGAAGAAGACTGC---CAAGGTCTCTTGGAACACGTCGTCAAGTCCATACTTCAGAATGGCG---ACGACAACGTCCTCAAGTGTGCAGCCATCCTGTCGAACGTCACGCAGACAAAGCCAGGCTCCGAGAAAGTCTGGACCACACTGACGTCGCTGGGCAAGACGGAAGAACTCCTAGAAGCCTTTGTCTCGACCGACGGCGAGTCTAGTAATCGGGACCACCTCGGCTTTGTGTTCTCAAACCTCAGTCAGCTCATTTCTGTGAGAAGATGGTTCTTGGATAAAACAGCATCAAGGATACACAAAATGTTTCCATTTCTCACGCTGGAAACATCTACAACGCGAAAGCATGGTGCAGTAGGAACGTTAAGAAACTGCTGTTTCGAAATGGACCACAACGAGTGGCTTCTGGGCCCCGAGGTGGACATCCTGCCCCGCCTGCTCTACCCGCTCATGGGCCCCGAGGAGCTGGACGAAGACGAGATGGAGAAGCTGCCCCTGGACCTGCAGTACCTGGGGACCGACAAGCAGCGGGAGAGGAGCCCAGAGATACGCAAGATGCTCATCGAGGCCCTCACACAGCTGTGTGCAACCAAGGAGTGCCGTAAGGTGATCAAGGACAGCGGGGCGTACTACGTGCTTCGGGAGCTGCACAAGACGGAGCTCTGTCGACCGGTGGTGGTTGCCTGCGAGAACCTGGTGGACATCCTGATCAGCGATGAACCGGAACCGGGCATGGAGAACCTCAAGGAGGTCGAAGTGCCCGACCATTTGGTCAAAAAGTTCCACAAGTACAACGAGGAACTG [960]

Siro ACTGCAACAGATGATGGCTTACAAATTTTAACGAAAGACTGTAAATTTTTGAAATGCCTTGCCGCACTATTA---AACTCTGACTCGGAAATTATAATGAACCAGGTTCTGAAATGTTTTGTGAACTTGACCAGCGTAGAGTCGGTGATAACCGAACTCCGTCAGTTAGATGAAGAATTGGATTTTGTAGCAAAACTACTTTCTTTGCTCACGTACGAAACGAGTCGCAGTTTTACTCCTATGGTTGCGATGATTCTCTCGAATATTACAAGGCAAGCGTTTATGTCCGAGTTGGTCTTAAGCAAATTAACGGATGTTGGTGGAATGGTGAAATTAATGATTCTACTGCGTAAAGGGATTCCAGAGTCATCGTAT---CACTACATCGCTTCGATCATAGCAAACTTGACTCAAATCGCTAATGGCAGAAGATTGCTTATGGAC---GATAAGGAAGAATTTAAAAATATTTTGCCCTTTACTTTGTATATGGATTCGGAAGTGCGACGCTTAGGAATTGTAGGAACACTTCGAAATTGCTGTTTTGATACGGAATATCATTCGCTACTATTGGACGAGAAAATGAACTTGCTATCATATCTTCTTCTGCCATTGGCTGGTCCCGAAGAATTTGATGAAGAAGACAATGAGAAATTGCCGTTGGATTTGCAGTATTTAGACAACAATAAACAGCGTGATCGCAATCCAGAAATTCGTAGACTGCTAATAGAAGCTCTCACGCAGCTTTGTGCCAAGCCAGAAGGTCGCCGTATGTTACGAGACAAAAACGTCTATGTGATTATGAGAGAATTGCACAATTGGGAGAAAAAAGCTGATGTCATCGTAGTTTGTTTGAACTTAATTGATATTTTAATTGGTGACGAACCACAGCCGGGAATGGAAAATTTAAAAGAAATCGACATTCCAACGGAAATGGTTGAAAAATTTGAAAAAATGGACGAAGAATTG [960]

Sitalc ACGGGGTCAGCAGAGGGCACTGAACACATTCTTGAGAGCAAAGATTACCTTCAAACAATTTGTAAACTCTTTTCAAACCATTCCAACAACTTTGTTGCCGAGAAGGCTCTAAAATGTCTCATAAATCTAACAGCCGATGAACAGTATGTTCAGAAAGTAACTGAAGCCTGC------CCCGAACTTGTGAGTGACCTTGTAAAGACCATTTTGTCCGGAACG------CAATGCGTTCAGTTAGCGCTGATGGTGTTCGCAAACGTCACTCGTCTCGAATCCATCTGTGAGGAAACTTTGAAGATCGTCACAGCTCATGGTGGATTGCGCAAGTTTGTGGAATTACTGAATTCCAACATCAACCGGGACCTGTAC---GATTTTACTGCCATGGTTTTGTCTAACCTAAGCAGGGGATCTGCACCCAGAAGAGAACTAATGTGCAATGACTTGGAACTATTGAGGATACTACTGCCTTTCACGATATACCCAGACTCTGGAGTACGAAGACTGGGAGTTGTGGCAACTATTCGCAACTGCTGCTTTGACATGGAGTACCACCCAAGCCTTCTGAAAGACGACTGTGGTCTTCTGTCTGCGATTTGTCTACCGTTGGCGGGACCAGAAGATCTTGATGATGACGAAATGGAGAAGTTACCTTTGGAGTTGCAGTATTTAGGAGAAGACAAGAAGAGAGATTCAGACCCACAAATTCGAAAAATGTGTATTGAAGCTCTCAACCAGTTGTGTGCCACCAAACAAGGCCGCAGTGCTCTTCGAGAGAAGGGCATTTACTACATCCTCCGAGAGCTGCACAAAAGTGAAACCGATCGTTCCACTTCGGTCGCTTGTGAGAACGTTGTAGACATTCTGATCGGAGACGAACCAGAACCACAGCATTCCAATTTGAAAGAGATTCAAGTTTCACCGGAGTTGGAGGATCGGTTTAACAAAATGAACACAGAATTA [954]

Sclero ACTGGCTCAGGAGAAGGCCTCTCCTACTTGATAGAACATTCTGAATATATGCAAGTGCTATGTGATGTGATTTCGAAAGATACTTCCTTTCCCATGATTGAAAAGGCTTTGAAATGTTTATTAAATTTAACAGCTGACGATGTTCACATACATAAATATATCGACACATGT------CCAAAACTTATGTCCACTTTAATCAGTCATGCCTTTACTGAGCAG------AAGTGTGTCCAATTAGCTTTCATGGTTTTAGCAAATATAACACGTGTGGAATCGGTTGTGGCAATAACTCTCACAGAAATAAATAATCATGGAGGCTTACAAAAATTGGTGGAAATGTTGAATCGACTCGAATCCTCCGACCAGTTT---CATTACACCGCTTCAATTCTTTCCAACTTGAGCCAAAGACCGGAAGCCAGAAAACAGCTGATGGCAAACAAATTTGATTTGTTTTCTAAACTCTTGCCTTTTACAACTTATTCCGTTTCTAGTGTGAGAAGACTCGGAGCCGTCGGAGCCATAAGAAATTGCTGCTTTGATACAGAATATCACAATGAATTGCTAGATGACAAACTCAACGTCTTAGTTTCCCTTTGCCTGCCTCTAGCAGGGCCAGATTCCCTTGATGAGGAAGATATGGAGAAATTGCCTTTGGATCTTCAGTATCTGGGAGAAGAGAAGAAAAGGGAAGCCGATCCTCGGATCAGGAAAATGTGTATTGAAGCACTTCACCAGCTTTGTGCAACCAAACATGGCCGAGAAGTATTCCAAACGAAAGGCATCTACTATGTTTTGAGGGAGTTGCATAAAACTGAGGAAGACCAATCCGTTGTCATTGCTTGTGAAAATTTGGTGGACATTTTAATTAGTGATGAACCAGCTCCTGAATATTCAAATCTCAAAGAAGTGCATATTCCCATTGATTTGGCAAACAAATTTGATAAAATGAACATGGATTTA [954]

Trogulus ACAGGTTCTGAAGGAGGTATCAACATCATAACTGCCAAGAAATCCTATGTTGAAATTCTTGCTACATTGATT---GAACCTTTAAAATCTGAATTATGCAAATTGTCTCTAAAATGTTTTGTTAATTTATCGACTGAGAGCCTTCTTACAGAAAAAATAATCGAATTTCAA------CCTAATTTAATAGAGAAACTATTATTAGTAGTGTATTCAAACACA------GAGTGTGTTTCAATTGCCATATCTGTTTTATCAAATATGACAATTTCAAGCCTTGCCAGCAGAGTGCTCCTTGAGGCTATAAATGCTAATGGAGGAACAGTTGCTTTTTTAAATCTTATGAAACATGGAAAACCGGATTCAGAGTAC---CATTATATGGCCTTAATTCTCTCAAATTTAACGCAGCTGCCAGAAACCAGAAAATTATTAATGAGTAGAGAAGACATTTTAATTAAGAACCTGTTTCCTTATGCAACATATGCCAACTCAACGACCAGAAGATTAGGGATTGTGGGTGCAATACGAAATTGTTGTTTTGATACTGAAAACCACGAATGGCTTTTAAGTGAAGATATTAATATCCTTCCAACACTATTACTTCCTTTGGCTGGCCCTGAAGAATTTGATGAAGAAGACACAGAAAAACTTCCATCTGAACTTCAATATCTGACTTCTGACAAAGAAAGAGATTCCAATCCTGAAATCCGAAAGATGCTAATTGAGGCAATAAACCAGCTCTGTGCAACGAAACCCGGAAGAAAGGTTCTGAAAACAAAAGGTGTGTACCTAATCCTGAGGGAACTGCACAAATGGGAGAAGGACGCTGCAGTACTTGTGGCCTGTGAAAATGTAGTCGATATTTTAATCAGCGATGAACCGCCTCTTGAATTGGAAAACCTGAAAGACATTGAAATACCCGATCACCTTGCCAAAAGGTTTGACGAGATAAACGATGATTTG [951]

Ortho ACTGGTTCTGGAGAAGGTATTGATAACATTGTAGAAAAGGCATCCCACGTAAAAACCCTAGCCTCATTATTA---GAACCACCA---ACAGATTTAACAGTAATTTCGTTAAAATGTTTTGTAAACTTGACCGCAGAAACGCTGCTAACCGAAAAAATTATTGAGGCAGAA------CCAGACTTGATCAATAAGCTTAAAACATTAATATTTTCGGATTGC------AAGTATATCTCCGTTGCAATAATGGTCTTGTCAAACATGACAATTTCAAAAATTGCCAGTAAACAACTACTAGACTGTATG---------GACGGAATATTTGGCCTCTTAGACCTTATGAACTGTGGCAAACCAGCTTCAGATTAT---GACTATTTGGCATCAATCTTATCCAATATAACACAACTGCCAGAGACTAGAAGGTTACTTATGGAAAAAGAAACTTTCCTT------AAGTTGCTTCCTTATACAACATACAAGGATTCACAAATTAGAAGAAAAGGATTGGTTGGTGCTATTAGAAACTGCTGTTTTGACATTGACCACCATGAATGTCTCTTGAGCGATGAGGTTGATATAATTCCTAATCTGCTACTTCCATTGGCCGGACCAGAAGAGTTTGACGAAGAAGATACAGAAAAGCTCCCATTGGAACTACAATATCTGAGTTCAGACAAAGAAAGGGATTCATGCCCAGAAATCCGAAAGATGCTGATTGAGTCACTCAATCAGCTTTGTGCAACTAAACAAGGAAGGAAAACCTTGAAAGAAAAAGGTGTGTACTTGATTCTCCGGGAACTTCACAAATGGGAAAAGGATTCAAGTGTTATCGTAGCTTGTGAAAACTTGGTAGATATTCTAATTGGCGATGAACCACCTCCAGAAATGGAAAATCTGAAAGAAGTTGAAATCCCAGATGATCTAGCAAAGAAGTTTGATGATATGAACGAAGACCTG [933]

Hespero ?????????????????????????????????????????????????????????????????????????????????????????????????????????????????????????????????????????????????????????????????????????????????GACCGTTTTGTCTCCAAAATTATGGATTTGATATATTCGAAATCT------GAATTTTGTTCAACCTGTTGCATGGTTTTGTCCAATATAACACGCTCTCAGAAAGCGAGTGAATGCGTATTCCAGTACTTGATCCGAAATGGTGGACTAAGTCCATTTTTGGATTTAATTCATTGTGGAAAACCGGCCGCTTCTTAT---AATCTCTTGGCGTCAGTTTTGGAGAATTTAACCCAACTTCCAGAAGCTAGAATTTTGTTGTTGAACAAGAGCGATGCTTTGATAAAAAGCCTATTTCCATACACTACATACTTGGACTCGGTGGTTAGAAGATTAGGAATGATCGGAGCTATTAGAAATTGCTGTTTTGACTCAGCGTATCATGAATGGCTACTGAGTGACGAAGTTGATATTATCCCCCCTTTATTATTGCCTTTGGCCGGTCCTGAGGAATATGATGACGAGGATACCGAACAACTTCCGTTAGAGCTTCAATATTTAGGAGAAGAGAAAAATAGGGATCCTAGTCCTGAAGTTCGGAAAATGTTAATAGAGGCATTACATCAGTTGGTTGCTACCAAACACGGTCGTGCTGTGTTTAAACGCAAAGGGGTTTACTTGATTCTCCGAGAGCTTCACAAATGGGAGAAAGAACGACATGTTGTTGTAGCGTGTGAGAATTTGGTTGACGTGTTGATTAGTGACGAACCTCCGTCTGAAATGGAGAACTTGAAGGAAGTTAACATTCCTGATGATCTGGTTGAAAAATTTGATAAACTAGATCAAGATTTG [960]

Leiobunum ACAGGCAACAGCGAAGGTATTCTATACGTATTA---CGCCAAAACTACTTACAGTGTTTATGCAACTTAGTT---GAAACCAATGATATAAACATTGTTGACCTTGCTTTAAAATGTCTCGTAAATTTGTCAGGGGAAGATTTGTTTTTCAATGCCCTTCAAAGTATAGAAAAT---AATAATCTGTTACATAATGTAACGCAAATGATCTATTCATCCTCA---GATAAACTATACACAAATGCTTTAATGTTTCTCATGAATGTTACCCGATTTAATACTGTCAGTAAACTGGTGCTAGCTTCATTAGTTTCAAATGGGGGACTTAAAAAATTTCTAGAATTGTTAAATAGTGGAAAACCAGATTCAAATTAC---CATTTGATGGGTCCCATTTTATGCAACTTGACATCATTACCAGAAGCTAGAAAAATGTTATTAGAT---GGTGATAAATTAATAAAAAATTTGCTGGCTTACACAACTTACAAAGATGATGAATTAAGGAAATTGGGCATTATAGGAACCATTAGGAACTGTTGTTTTGAAACTGAATACCACGAACTCTTGCTTGGAGAAGAAATTGACATCCTTCCTGCTTTGCTTCTTCCAATTGCTGGCCCTGAAGAATTTGACGAGGATGAGAATGAAAACCTACCGTTAGAACTTCAATATCTAGATGAAAGCAAAACGAGAGATTCAAATCCTAAAGTTCGGTTAAGAGTGATAGAAGCTCTTAATCAGTTATGCGCCACTAAGTTTGGAAGAAATAAACTCAAAGAAAAACAAGCTTACTTAATCCTCAGAGAACTTCATAAATGGGAGAAAAACAGAAAAGTTATAATTGCGTGTGAAAATGTAGTGGACATTTTAATAGGAGATGAACCACCTGAAGAACTGGAAAATTTCAAAGAAATTGAAATACCAGAAGATTTGGCTAAAAAATTTGAAAAAATTGATGATGATTTG [951]

Proto ACTGGTAGCGAAGAAGGCATTCAACATCTATTA---TGTGAAAAATATTTTTTGTGCTTATGTGGCTTAGTT---GAAGTCAATGACATAAATGTTGTTGATCTCGCTTTAAAATGCCTAATAAATTTGTCGGGGGAGGATTTGTTTTTCGACCTACTGCAAAGTACAGAA------AATAATCTATTACAAAATGTAACTCAAATGATCTACTCATCCTCT---GTAAAACTAAACACTAATGCATTTATGTTTCTCATGAATGTAACACGGTTTAATACTGTCAGTAAACTAGTACTAGATTCGTTAATGCTACATGGGGGAATTAAAAAATATATTGAGTTGTTAAATAGTGGAAAACCAGAATCAAATTAT---CATTTAATGGGCCCAATTTTATGCAACCTGACTTCCTTACCAGAAGCTAGAAAAATGCTACTAGGT---GAAGATAAATTAATAAAAAATTTGCTGGCTTACACAACTTATGAACACGATGAATTGAGAAAACTAGGCATTGTAGGAACTATCAGAAACTGTTGCTTTGAAACTGAATATCACCAACTCCTACTTGGCGATGAAATTGATATCCTTCCAGCTTTGCTTCTTCCAATTGCTGGCCCCGAAGAATTTGACGAGGATGATAATGAAAAGCTACCATTAGAACTACAGTACTTAGATGACAGTAAAACGAGGGATCCAAACCCTAAAGTTCGATTAAGATTAATAGAAGCCCTTAATCAGCTCTGCGCCACTAAAATTGGAAGAAACGTGTTAAAGGAAAAACAAGCTTACTTGATCCTGAGAGAACTTCACAAGTGGGAGAAAAATAGGAAAGTTGTTGTTGCCTGTGAAAATGTTGTGGACATACTCATAGGTGATGATCCACCTGAAGAACTTGAAAATTTCCAAGAAATTGAAATTCCAGATGATTTGGTTAAAAAGTTTGAAAAAATTGATGAGGATTTG [948]

[TICKFAST_NCHAR=300;carrier protein]

Ixodes GGAAAATGTATATCATCGGCAATGAAAAAAAGCGTGCGTGTTGTCGTGCCGCGCTTTGTCTTGAACACGTACCTGATGGCGCACTACAAGGAGAGAACCGAGTACGATGTTTTCGACGCCAATGAAGAATGCAAGCCCGGTGATTGGGTAATTATCAGGGAGCTTCCGGAACGCCTGAGCCTCAAGATTGCACACAAGGTTGAAAAAATTGTCTTCAAGGACGGCAACATCATTGATCCGTTGACAGGTCAAAAGTGCATGTTCACCGATTATGTGAAAGATGTTGTACCGAGAGAGTGA [300]

Siro GCAAAAGTTATTCGGTCCCCGATGAAAAAAACCATTTTAGTCAATGTTCCCATAATGAAAATGGACGAACGCATGCAAATGCATTTCCGTGAAAATGACGAATACAATGTGTACGACCCTGAAGAAGTATGCAAACCTGGAGATATCGTTCTCATTCGTGAATTGCCTGCGGCAATCAGTTTAACAGTGAAACACAAGTTGGAGAAAGTCGTGTACACACTCGGCGAAATGATCGACCCGATAACGGGAAATCGCTGCTTTGGAACCGAATACGTGAGTGAAGTGGATCACATCGGCGAA [300]

Sitalc GTTGTAGATTTAATTACGCCTGTTAAAAACACAATTAGGGTATTTACGCCAAAGCTAACGATGAATAAAGTTTACCTGATGTACCTACGATCGAATGACGAGGTATTCGTTCACGACCCAGCTGGAAAGTGCCGACCTGGTGACTACGTACTTATTAAAGAGTTACCCGAACCCATGAGCATTCTGGTGAAGCACAAGTTGGAGAAAGTGGTGTACCAAGTCGGCCATTTTGTTGATCCGGTGACAGATCGCAAATGTATGGGATCGAAGGACATCCAAGATTTAGATACTCTTGGAGAA [300]

Sclero GTTGTCGATTTAATGACCCCAGTCAAAAATACAATTCGTGTTCTGTCACCAAAATTAGTAATGAATGAACGATTTAATGTGTACCTGAGAGACAACGAAGAGATGTTTGCTCACGATCCCCAAAGCCAATGTCAGCCAGGAGACCATGTCCTCATCAGGCTTCTGCCTGAACCTATGAGCCTGCTGGTCAAATATCAGCTTGAAAAAGTCGTGTACCGAGTGGGGAACATTATTGACCCAGTCACGGGCAAGAAGGCCTTCTACACCAGGAGCGCGGAGGATCTGAATTTCCTGGGGGAA [300]

Trogulus GTCGTTGATTCAGTAACTCCTATTAAGCAAACAATAAGAGTCCTCGCACCAAAATTAGTTATGGATCAACGTTATAAAATGTATTTTAAAGAAAATGAGGAGTATTTTGCACATGATGCTAAGGAGGAGTGCAGGCCAGGGGACTTAATTATAATTCAAGAGATGGAAAAGCCTATCAGTTTACGTGTGAAGCACGAGTTTGTCAAAATTGTGTTCCAAAGTGGCAACATTATTGATCCTTTAACAGGGAAGAAGTGCTTAGGCGACAAATATATAGATGACATCAGGCAATTGAGTGAA [300]

Ortho GTTATTGATTCTGTAACTCCAGTAAAGCAAACTATAAGAGTGTTTGCTCCTAAATTAGTGAGGGATGAACGTTATAATATGTATTTCAAAGAAAACGACGAATATTACGCTCACGATCCCAATGAAGAATGCAAACCCGGTGATATTGTATTAATTCAGGAACTACAAAAACCTCTGAGCTTAAAAGTGAAACATACACTGGTGAAACTGATTTACGAAGAGGGCAACATAATCGACCCCATAACGGGACGGAAATGCATAGGTGACAAATTTGTGGACGATATTCAGCAGCTGGGAGAC [300]

Hespero GTCGTCGATGCTTTAACGCCGTTAAAACAAACTGTTAGAGTTACGGTACCCAATATGGTTAAAGACGAAAGACTTTTAATGTATTTTAAGGAAAGTGAGGAATATTTTGCCCATGATCCAACGGAGGTTTGTAAACCTGGAGACATAGTTCTTATTAAGGAACTTCCGAAACCTTTAAGTTTAAAAGTGAAACACGAACTAGTAAAAGTTATTTACGAGGATGGAAATATCATTGATCCTATTACAAACCGAAAATGCTTGAAAGATAAATATGTGGACGAACTGGAACATTTGGGTGAA [300]

Leiobunum GTCGTTGATTCACTAACTCCTTTAAAAGAAACTATAAGAGTTTTTGTTCCATTTATGAATCGAAATATGAAATATAATTTGTATTATAAGGAAAATGAGGAGTTTTTAGTCCACGATCCAGAGCAATTGGCCAAACCTGGTGATATAATTTTAATTAAAGAACTATCTCAAAGGTTAAGTTTAAAAGTAAATCACGGATTAGTGAATATCATTTACCAAAGTGGAAATATTATCGATCCTGTTACTGGAAAGAAATGTTTTGGTGACAAATACGTTGAAGACATGGAAACA????????? [300]

Proto GTCGTGGACTCACTTACTCCTTTGAAAGAAACAATAAGAGTTTTTGTTCCATTCATGAATCGAAATATGAAGTATAATATGTATTATAAAGAAAATGAAGAGTTTTTAGTACACGATCCAAAACAAATAGCCAAACCTGGTGATATTATTTTAATTAAAGAACTGCCGCAAAGGTTAAGCTTAAAAGTAAACCATGGTTTAGTTAATGTTGTTTACCAACTTGGAAATATTATCGATCCTGTTACGGGGAAGAAAAGTTTCGGTGACAAATATGTAGAAGACATGGAGTCACTTGGAGAA [300]

[TICKFAST_NCHAR=669;caveolin]

Ixodes AAAGACAAGAGCTCCAGGTCCGGCTCTCGCGCCAACCTGAACGAGTCCACCCTGCCGCTCCTGGACGACGTGGCC------------GACACCAAGGGCGAGGAACTCCGCAAGGAGAAGATCGAGCTGGAGACCAAGGTGGGCGGCGCGGACTCTGGGTCCAAGGGCGAAGATTCGAACGCGGCAACG---------GCCGAGCCCGGCTCGGACACCGGCAAGGACGTCGCAGAGTCCGACAAGGAGGACAAGAAGGGCTCCAAGAAGAAGGCCAAG------------AAGGAGAAAAAGGAGAAG------------------AAAGTCCACAAGAGGTCGCCGTCTTGCGTCGAGACGCTCACCATCGGGCTCAACCTCCTCGATCGGGACGAGAAGCACGTCAATGACCACGTGAACCTGGTGTTCGAGGACGTGCTGGCGGAGCCCGACGCGAGCCACGGCTTCGACGGCGCCTGGAAGCTGACGTACCTGGTGTTCTCGACCACGCGGCTCTGGTGCTACCGGCTGCTGTCGGCGCTGCTGGCCCTGCCGTGCGGCTTCACCTGGGGGCTGCTCTTCTCCATCCTCTCGCTGCTCCACGTCTGGCTCTTCACGCCCCTGCTCCGCATCTTCGACGTGCTCCTGCACGTGCTCCACAGGGCG [618]

Siro AAGGACAAAAGTTCGCGTTCCGGCTCAAAGGTCAATCTGAACGAGTCGACCCTGCCGCTGTTAGATGACCAGGAGACG---------GAAGGCAAAGGAGAGACGCCGGAGAAGGAAAGGATCGAGCTCGAGCTGAAATCGGAATTGGAAAAAGAAGATGAAGGAAAAGAAAAAAACGAAGAT---------------------------------------GGTCAAGTGGAAGATGGAGATAAAAAGAAAAAAGATAAAAAAGACAAGAAAGAAAAA------------AAGAAGAAAGAGGAGAAA---------------ATCAAGACACATCGGAGAAGCGTCTCTTGCGCAGATACACTTACCGTTGGACTCAACTTACTGGACAGAGACGAGAAGAAAGTTAATGACCATATTAACTTGGTCTTCGAGGATGTTCTAGCTGAACCAGATGCGACTCACGGATTCGATGGCGCTTGGAAGTTTGCCTTTGTGGTATTCTCTGGTACAAAGCACTGGGTGTACCGCATCTTGGCTGCTATCCTGGCTCTTCCATGTGGGCTACTGTGGGGTCTGGTCTTCTCATTGCTGACCCTCGCCCATGTCTGGGTCTTCACACCCTTCCTTCGTACCCTCGATATCGTCTTTCTCCTCGTCCGTAGGATT [594]

Sitalc AAAGACAAG---AGTTGCGGTGGTTCAAAGGCTGATCTGAACGAGTCCAATATGCCTCTTCTGGACGACCAAGTG---------------------GACAACAAGTCCATCAAAGAGAAGATCGAGATGGAAACC---------------------------------------------------------------------------------------------AAAGAGGTGGACAACGGAGAACTGGCCAAAGAACAAGAGGCCGAGGAAGAG------------AAGAAAACGAAGAAGAAG------------------AAGGGGCACAAGAGGACAGTCTCCTGTGCAGACACAATGACCATTGGCATCAATCTTCTAGACAGAGATGAGAAGAAGGTCAACGAGCATGTCAATCTGACTTTCGAGGACGTACTGGCCGAACCCGACTCGAGCCAATCCCTTGCAGGATCCTGGAGGTTGGCCTATACCATGTTCTCCGGGAGTCGCCTTTGGATGTACCGCCTGATGACCGCAGTGCTGGCCTTGCCTTGCGGACTCGCCTGGGGGATCGTCTTCTCTCTTCTCACCATACTTCATGTTTGGCTCTTTACCCCACTGCTCAAAACCTTGGATCTAGGATTTTTCGTTATTCGAAGGGTT [522]

Sclero AAGGAAAAA---AGCCTGGGCGGGTCAAAGGCCGAACTGAACGAATCGACGCTGCCTTTGCTCGACGACCAAGTG---------------------GACAACAAGTCGGGCAAGGAGAAGATTGAGATGGAGTGC---------------------------------------------------------------------------------------------ACCAAGGAGACGGACAACGGCGAGACCAAGGAACCGCCCAAGGAGGAGGGG------------AGCAAGAAGAGCAAGAAG------------------AAGGGCCATCGCCGAGCAGCGTCTTGCGCCGACACCATGACCGTCGGGATCAACCTTCTGGACCGGGACGAGAAGAGAGTCAACCAACATGTCAACTTGACCTTCGAGGACGTCCTAGCCGAACCGGACTCCAGCCAAGGACTGGCCGGATCCTGGAAGTTGTCCTACGCCGTCTTTTCCACCACTCATCTGTGGGTCTACCGTCTGGTCACCGCCCTGCTGGCCCTGCCCTTCGGACTGCTCTGGGGACTCGTCTTCTCGCTGCTGACCATCCTGCACGTCTGGATCTTCACGCCCCTCCTCAAGACCATGGACCTAGGACTGCACGTGATCAGGAGGGTA [522]

Trogulus AAAGATAAAAGTTCTCGATCTGGCTCTAAAGTAAATTTAAATGAGTCCACGCTGCCCTTGTTAGACGACCAAGAGGTGATTAGCTGCGAGGGCAAAGGTGACACTCCAGAAAAGGAAGCCATCGAGATGGAAACAAGGTCCGCCGATGAAAAAGGAGACGAAGGAACCGAGAAGACAAACGAAGTAGAGGAGGCC---AATGACCACAAGGAGGACCACAAGGACGACCAGAAAGAAGACAAGAAGAAAAAGAAGAAAGAGGAAAAGGAAAAAGAAAAG------------AAGGAGAAGAAAGAGAAGAAGAAGGAAAG---CGTCAAGGGACACAGAAGGACTGTCTCTTGTGCTGAGACCTTGACGGTGGGACTCAACTTACTGGACAGAGACGAAAAGCGAATCAACGATCAGGTTAACCTCGTTTTCGAAGACATCCTAGCCGAGCCGGATTCGAACCAAGGCTTCGAAGGAGTATGGAAACTAACATACGCCGTCTTCTCTGGGACCAAACAATGGGCTTACAAACTTCTTTCCGCAGTCTTAGCTCTTCCCTGTGGCATAACTTGGGGTCTGATCTTTTCAATTTTGACACTGATTCACGTCTGGCTGTTAACACCCCTGCTTAGGACACTGGACATCGTCTTCTACGTAATCAGAAGGGTC [651]

Ortho AAAGACAAAAGTTCTCGATCTGGATCCAAAGTCAACCTAAACGAGTCGACGCTGCCACTGCTAGACGACCAGGAGGTCATCAGCTGCGAAGGCAAAGGCGAGACGCCGGAGAAGGAGTCCATAGAGATGGAGACCAGGTCGGCCGACGAGAAGGGAGACGAGGGAACGGAGAAGACCATCGAAGCTGACGACCATAAGGACGACAACAACGAAGACCACAAGGCAGAGAGCAAGGAGGACAAGAAGAAGAAGAAGAAGGAAGAGAAGGAGAAGGAAAAG------------AAAGAGAAGAAGGAGAAGAAAAAGGAGAGCGCCGTTAAGGGACACAGGAGGACTGTCTCCTGCGCTGACACCCTCACCGTAGGACTCAACTTACTGGACAGAGATGAGAAACGTATCAACGATCAAGTTAACCTTGTCTTTGAAGATGTAATAGCAGAGCCGGATTCGAACCAGGGCTTCGAGGGTGTCTGGAGACTAAACTACGCAGTCTTCTCCGGTACCAAGCAATGGGCCTACAAGCTTCTATCCGCCATCTTGGCCCTTCCATGCGGCATCGCATGGGGTCTGATCTTCTCCATCCTGACACTGACACACGTGTGGCTACTCACGCCTCTACTCAGGACCATGGATATTCTCTTTCACGTTATCAGAAGGGTC [657]

Hespero GAAGAAAAAAGTTCCCGC---GGTTCCAAATTGAATTTGAACGAGTCCACGTTGCCATTGTTGGACGACCAAGAGGTGATCAGTTGCGAGGTCAAAGAGGACACTCCCGAGAAGGAGCAGATCGAGATGGAGACAAGGTCGGCCGACGAGAAAGGAGACGAAGGGACCGAGAAGACAGACGAGACGGCG------------------ACCGAAGAGGCCAACGACGACAAAGAAGGGGACAGCAAGGACAAGAAGAAGAAGAAAGACAAGAAGGACAAG------------AAAGAGAAAAAAGACAAG---------------GTCAAAGGGCACAAGAGGACCCTGTCGTGCGCAGACACCCTGACGATCGGACTCAATCTCTTGGACAGGGACGAGAAACACATCAACGATCAGATCAATTTAGTTTTCGAAGACGTTTTGGCTGAACCCGACGCCACTCAAGGTTTCGATGGTGTTTGGAAACTAAGTTTTGCAGTGTTTTCCGGTACCAAGCAATGGGTTTACAGGATTCTAGCCGCCATCTTGGCTTTACCTTGCGGTATTGCCTGGGGTCTGATTTTCTCCGTGCTTACATTGGCTCACGTTTGGGTTCTGACACCTCTACTAAGGACGCTGGAGATTTTATTTAACGTCGTACAAAGGGTC [621]

Leiobunum AAAGATAAGAGTTCCAGGTCGGGTTCCAAGAGCAATCTGAACGAGTCGACGTTGCCGCTTCTGGACGACCAGGAGACGATC------GAAATGAAGGGCGACACTCCCGAGAAGGAGAAGATCGAGTTGGAGACGAGGTCCAGTTCCGAGACCGAGAAGGGGGACGAGGGCAAAGATGACGGACGCGCCGAGGACGGGGAAAAGACTGACGGCGAGGCGGAGGGAGGAAACTCCACCAGCAAAAAGGACAAGAAGAAAAAGAAGGACAAGAAAGAAAAGGAGGAAAAGGCCAAAGACAAGAAGGACAAG---------------GTGAAAACCCACAGGAGAACCCTGTCCTGCGCCGACACGCTGACCGTCGGACTCAACCTTCTCGACAGAGACGAGAAACACATCAACGATCAAGTTAACTTGGTATTCGAAGATATCTTGGCGGAACCGGACTCAACTCAGGGCTTTGACGGAATTTGGAAACTGACGTACGCCGTCTTCTCGGGCACCAAGCAGTGGGTCTACCGCATCCTATCGGCACTATTGGCCTTGCCGTGTGGAATCGCGTGGGGACTAATCTTTTCGATATTGACCATCGTCCATGTCTGGGTTTTGAGTCCTCTACTGCGCACTTTGGATATTGCCTTCTACGTTGTCCGAAGGGTG [648]

Proto AAAGACAAGAGCTCCAGGTCGGGTTCCAAGAGCAATCTCAACGAGTCGACGTTGCCACTGCTGGACGACCAGGAGACGATC------GAGCTGAAGGGCGACACGCCCGAGAAGGAGAAGATCGAGATGGAGACGAGGTCCACCTCCGAGACGGAAAAGGGCGACGAGGGCAAGGAGGACGGACGCGCCGAGGACGGCGAGAAGACCGACGGCGAGGCGGAAGGAGGCAACTCCACCAGCAAGAAGGACAAGAAGAAAAAGAAGGACAAGAAGGAAAAG------GAGGCCAAAGAGAAAAAGGACAAG---------------GTCAAGACCCACCGGAGAACCTTGTCCTGCGCCGACACACTCACCGTCGGGCTCAACCTTCTCGACAGAGACGAGAAACACATCAACGATCAAGTCAACTTGGTATTCGAAGACATCTTGGCCGAACCCGACTCAACTCAGGGCTTCGACGGAATTTGGAAACTGACATACGCCGTCTTCTCCGGCACCAAGCAGTGGGTCTACCGCGTCCTGTCGGCACTATTGGCTTTGCCGTGCGGAATCGCCTGGGGACTCATCTTTTCCATATTGACCATCGTCCACGTTTGGGTTTTGAGTCCTCTATTGCGTACCTTGGACATCGTCTTCTACGTAGTTCGAAGGGTC [642]

[TICKFAST_NCHAR=678;cytochrome C oxidase assembly protein]

Ixodes AAAGAGGACGGCAAGAAGAAGAAAGGGCCAATAACGTGGAAGAGCCTCGGAATCACTTTTGGAATTGGCGGTGCCCTCCTAGGCTGGATGTTCTACATCAAGAAAGAGAAGCAGCGAGAGATGGACCGAGAGCGTAAGCGGTCGCTGGGCAAGGCGGCCATCGGGGGTTCCTTCGAGCTGGTGGACCACCACGGCCAGCCCAAGAGCAGTAAGGATTTCCTGGGAAAGTGGCTCCTCATCTACTTCGGCTTCACCCACTGCCCAGACATCTGCCCGGACGAGCTGGACAAGCTGGGCAAGGTCATCGACATCGTGGACAACGAGATCAAGGATGTGGCACTCCAGCCGCTCTTCATCTCCATCGACCCCGAGAGGGACGACGTCAAAGCCGTCGCGGCGTACGTCAAAGAGTTCCACCCGAGGATCCTAGGCCTGACGGGCTCCAAAGAGCAGGTGGAGCGGGCCTCCAGGGCGTTTAGGGTCTACTTCAGCGCGGGGCCCAGGGAC---GAGGAGCAGGACTACATAGTGGACCACACGGTCATCATGTACCTGGTGGATCCGGACGGGGAGTTTGTGGACTACTACGGCCAGAACCGGACGGCCAGGCAGGTTGCCACGGCCATCCAGCTGCAAGATGTCAAGTACAAGCGTGCCAAGAGTGGGGCCTGGTTCTAG [675]

Siro AAGAAAGAACAGGAGGGGAGACAGACTCTCATATCATGGAAGACAGTAGGAATTACATTCGGCATTGGAGGTGTTTTCCTAGCCGGAATGCTGTACATTAAGCGCAAAAAACAAATGCAGATTGATCGTGAAAGAAAACGTACTTTGGGCAAAGCTGCAATTGGCGGGTCGTTTGAGCTGATCGACCACAACGGGAAACCTTGTTCAAACAAAGATTTCTTGGGCCAGTGGGTCTTGATCTATTTTGGCTTCACACATTGCCCAGACATATGTCCGGACGAAATTGAAAAAATGGTTCTAGTTGCCGATCTTGTTGAGCAAAGA---AAACTACCACGTCCAATAACCCTTTTCATTACCGTCGATGCCGAGAGGGACAGCGTAGAAGCAGTCGCCGCATATATTAAAGAATTTCACCCAAAACTGATTGGTTTGACTGGCAACAAGGAGCAAATAGAGAAAACAAGTCGGACATTCCGTGTTTACTACAGCGCTGGACCAAAAGAT---AACACAAACGATTACATAGTTGACCACACAATCATTATTTACCTAATAGATCCAGAAGGTGAATTTCTGGATTATTATGGACAAACCAAGACAGCGGAACAGATTGCGGACGGAATCCAAATTCAAATGTCTAAAAGAAAT---AGGCAGAAGAGTTCTTGGTTTTAG [669]

Sitalc AGAGATAGTAAAGGAAATAAAAAATTTCCTGTCACATGGAAAAGCTTGTTCATCACTACTGCCATAGGTGGAACCTTCTTAGCTGGGATGATGTATACGCGAGATATTAAACGGCGTGAGCTTGAACTGGAGCGCAAACGCACATTGGGAAAAGCCAACATTGGAGGCCCATTTGAACTAGTGGACCAGAACAATAAACCATTCAGCAATACAAACCTCCTGGGAAAGTGGGTACTCATCTATTTTGGATTCACACACTGTCCTGATGTTTGTCCTGATGAGATGGAGAAGATGGTGGAAGCTACTGAATTGCTAGAGAAA------GGTGGAATTCCAGTTCAACCAGTCTTCATCACAGTTGATCCTGAGAGAGACAACGTCAAAGCTGTTTCAGAATATATCAAAGAATTCTCTCCTAAACTGATTGGACTGACAGGAACTCCTGAACAAATAGACAAAGCCACCAGAGCCTATCGTGTCTACTACAGTGCTGGACCCAGAGAAATCTCGGAGAACGATTATATTGTGGACCACACTATAATTATATATTTGGTTGATCCTGATGGAGCTTTTGTTGATTATTATGGTCAGAATATGACTGCACAACAAATATGTGATGGTGTCATGATAACCAAGGCCAAGTTTGAT---TCACTGAAGCGTTCCTGGTTCTGA [669]

Sclero AAATCGCCTAATCCTAAAGAAAATAAGGTATTTTCATGGAAAACGTTAGCCATAACTTCTGCAATTGGTGGAATTTTCCTTGGTGGAATGTTGTACGTAAAGAAGAAAAAACAACATGAACAGGAACTGACACGCAAGCAGTCTCTGGGTAAAGCAAGTATCGGAGGTCCATTTGAGCTGATTGACCAGACTGGCAAAACTGTGACCAATAAAGACTTCCTGGGCCAATGGCTGCTCATCTACTTTGGCTTCACACACTGCCCAGATGTCTGTCCCGATGAGATGGAAAAAATGGTTCAAACAAGTGATATTTTGGAGAAA------AAGGGAATCCATGTTCAGCCCATTTTCATCACGGTGGACCCAGAAAGGGACGATCATAAAGCAGTGGCGGCGTACGTTAAAGAGTTTTCCCCAAAAATGATCGGTCTGACTGGTAGCCGGGATCAAGTTGATAAAGCCTCACGAGCCTACCGTGTCTACTACAGTGCTGGGCCCAGGGACGAGGCAGACGAAGATTACATTGTGGATCACACAATCATCATTTACTTGATCAACCCTGACGGGGAATTTTTGGATTACTATGGACAAACCGTGGATGCAGAACAGCTTGCAAATGGCATCATAATTAACAAATCAAAATACGACATGTTACATAAAAAGTCTTGGTTTTAA [672]

Trogulus AAGAACCAACCTATTAAAGCGAAAAGTCCAATAACGTGGAAGAGCGTAGGTATAACGTTTGGCATCGGCGGCGCAATCATATTAGCAATGCTTCACTTCAAGAAATTAAAGCAAGAACGAATGGAAAAAGAGAGAAAACGTGTTATTGGCAAAGCAAAGATTGGGGGCAATTTCAACCTCGTCGACCACAACGGAAAACCTTGCTCGAGTAAAGATTTTTTTGGCAAATGGGTATTGATATATTTTGGCTTTACAAACTGTCCTGATATCTGCCCAGATGAAATAGAAAAGATGATCACAACTGTAGACATACTTGAAAAACGC---AACGTTACTAAAGTCCAGCCACTGTTTATCACAGTAGACCAAGAAAGGGATAACATTAAAGCAGTGGAATCTTATGTTAAGGAGTTTTCACCCAAGCTAATCGGTTTGACTGGCAGTAAAGAACAGATTGATGAAGCAACAAGGTTGTTTAGAGTATATTACAGTGCCGGACCAAGAGAC---AGTGATAATGACTATATTGTAGATCACACTATCATAATGTATTTAATTAATCCCGATGGAGAGTTTGTTGACTATTATGGGCAAAATAAAACTGCCGAAGAAGTTGCGGATGGTATTACTTTGAGCAAAATGAAATATGAA---------TCAAAATCATGGTTCTAA [663]

Ortho GAAAAACCTGTTGGCCGAGGGAAAGGCCCAGTCACATGGAAAACTGTTGGCATTACCTTTGCCATTGGCGGTGTGTTTTTAGCTGGAATGCTTTATGTCAAAAGGTTGAAACATGCAGAAATCGAAAAGGATAGGAAGCGAGCCATTGGAAAAGCAAAAATCGGAGGACCTTTCAACCTTGTGGATCACAATGGAAAACCCTGTTCTAGTAAAGACTTTCTGGGACAGTGGCTACTAATGTATTTTGGATTCACAAACTGTCCAGACATCTGTCCTGATGAAATAGAAAAAATGGTTAAAACTGTAGACTTACTAGAAAATAGG---AATGTTACTAAAGCTCAGGCCTTGTTTATAACAGTGGATCCAGATCGAGATAATGTGAAAGCAGTTGCCTCTTATGTTAAAGAGTTCTCTCCAAAATTGATTGGTTTAACAGGCAGCAAAGAACAAGTCCATGAAGCAAGCAGGAATTTCAGAGTATATTACAGTGCAGGGCCTAAAGAT---AAAGACGCAGACTATATTGTTGATCATACCATCATAATTTATCTAATCAACCCAGATGGAGAATTTGTTGATTATTATGGCCAAAACAAAACAGCTGAAGAAATAACAGATGGTGTAGCTCTAAATAAGGCAAAATATGAG------AGTGCTAAATCTTGGTTTTAA [666]

Hespero AAGCCAAAACCGGGCAAAGGAAAAGGGCCTATTACATGGAAAAGCCTTGGTATTACATTCGCTTTAGGAGGTGTATTAATGCTATGGATGTTATTTGCTAAAAAGAAAAAACAAGCAGAAATTGAAAAAGAGCGAAAACGAATGATTGGCAAGGCAAAATTGGGCGGCCCTTTCAATTTAACCGATCACAATGGCAAATCGTGCTCCACCAAAGATTTCTTAGGTCAATGGGTGCTCATTTATTTCGGGTTCACTCATTGTCCCGATATTTGTCCAGACGAAATTGAAAAAATGATTCAAGTTGTCAATGTTTTAGAGAATAAA---AACGAGGTCAAAGTTCAGCCTTTGTTTATAACGGTCGACCCCGACCGTGACAGCGTAGATGCGGTGGGCAAATACGTCAAAGATTTCTCTCCCAAATTGATCGGCTTGACCGGTTCTAAAGAGCAAATAGCCGAAGTGACCAAGAAATATAGAATTTATTATTCGTCCGGACCCAAAGAC---GAAGACAACGATTACATTGTCGACCATAGCATTATTATGTTCTTGATCGACCCACAGGGCAATTACGTAGATTACTATGGACAGAATAAGAATGCGGAGGAAGTCTCTAATGGAATATTATTTAATATTGCCAAATTTAAT---TTAGCCAATAGTTCGTGGTTCTAA [669]

Leiobunum AAGAAAAGCTTTGGAAAGGGAAAAGGGCCAATTACATGGAAATCTCTTGGAATCACATTTGCTGTTGGAGGAACGTTTTTATTAGGAATGTTGTATACCAAAAACAAGAAACAGAGAGAAATTGATCTAGAGCGAAGAAAAGCTCTAGGTAAAGCAAAAATTGGTGGCAGGTTCGATTTGATTGATCATAATGGAAAACCTGCAAAAAGTGAAGACTTTTTTGGAAAGTGGATCTTGCTTTATTTTGGTTTCACCCATTGTCCTGATATTTGTCCTGATGAAATGGAGAAGATGGTAAAAGCGACTGATATTTTGGAGAAAACA---CTGACAGTGGATATTCAACCTTTATTTATTACTGTGGATCCTGACAGAGACACAGTGCCAGCTGTAGCTGCTTATGTCAAAGAGTTTTCTCCAAAACTCATTGGATTAACTGGATCAAAAGAGCAAATTGATCAAGCTTCGAGGGCTTTCAGAGTTTATTACAGTGCTGGTCCAAAAGAT---GCCCAAGCTGACTACATTGTTGATCACACCATAATTATCTATCTAATAAATCCTGAAGGTGACTTTGTTGACTATTATGGCCAAAATAAAACTGCGGATGAAATAGCTAATGGTGTTACATTTAGCATAGCAAAATACAAT------GCCAGGAACTCTTGGTTTTAA [666]

Proto AAGAAAGTCTTTGGAAAGGGCAAAGGACCTATTACCTGGAAGTCTCTTGGAATTACATTTGCAGTTGGAGGAACATTTTTATTAGGGATAATGTATACTAAAAACAAGAAACAGAGAGAAATTGATTTAGAGCGAAGAAAAGCTCTTGGTAAGGCAAAAATTGGTGGAAGGTTTGATTTAATCGATCAAAATGGGAAACCTGCAAAAAGTGAAGATTTTTTTGGAAAATGGGTTCTACTTTACTTTGGGTTCACCCATTGCCCAGACATTTGCCCTGATGAAATGGAAAAGATGGTGAAAGCAACTGATATTCTTGCTGAG------AATAAACATGAAATCCAGCCTGTATTTATTACCGTGGATCCTGACAGAGACACAGTACCAGCTGTTGCTGCTTATGTCAAAGAGTTTTCTCCAAAACTCATTGGCTTAACTGGATCTAAAGAGCAAGTTAATGAAGCAACAAGGGCTTTTAGGGTTTATTACAGCGCTGGCCCAAAAGAT---GCGCAAGCAGACTACATTGTTGATCACACCATAATTATCTATCTAATAAATCCTGAAGGTGATTTTGTGGATTATTATGGACAAAATAAGACTGCGGAAGAAATTGCAAATGGTGTTACGTTTAGCATAGCAAAATATAAT------GCCAGGAACGCTTGGTTTTAA [663]

[TICKFAST_NCHAR=663;cytochrome P450]

Ixodes ATGTTAGAATTTGCAATATTTGCCGTGTGTTTCGTTGTCTTTCTGCTGATGCTCGTCGTCTATCTTTACCCGGGCTCT------AGCAAA---CAGACGACCATTCCTGGTATCGACCCGTCGGATCCAAAGGAAGGCAATGTTGGAGACATTGTCCAAGCAGGGGGACTCAAGAATTTCCTGCTCTCATTGCACAAGGACCATGGACCAATCACTTCGTTCTGGATGGGCACAAAGCTTGTCATCAGCATCGGTGATGCGGAGCTGTTCAAAACGCAAACGCACATCTTCGATAAACCCGCGGATCTCTTTGAGCTGTATCGCGACCTGGTTGGAGCAGACAGCATCTTGTTCGCCAATGGCGCAGAAGCACGCCAGAGGCGCAGGCGCTTGGACGACGTCCTTACGGGTGACCGCATCAACGATTTCGTCGGTCCCTTGCAAAAGATGTGCGCTGAAGTGGTGGATCGTTGGGAGAGTCTACCTGCGGAAGAGCACGTGCCCATCTACCAATACATGTATGCGCTCTGCATGAAGACCTGCACCCGTCTACTCTTTGGCGACTACTTCTTTGATGACAAACATGTCTTGGAGTTCAGCAGGGCCTTTGAGCTGTGTGTCAAGGAACTGGAAGAGATGGCTGGTGGCATGGTCCCAGACCCT [654]

Siro ATGCTTGATTTTGCTATATTTGCTGTTACATTCGTATTATTCCTCCTGCTGTTAGTGTTTTATCTTTATCCGGGATCC------AGAAAG---CAAACCACTATTCCTGGACTTGATCCATCGGACGCGAGGGAAGGTAATATTCAAGATGTCATAAAGGCCGGAAGTTTGCATCAGTTCCTGAGCGAACTGCACGCTGACCATGGACCAATCGCTTCCTTCTGGATGGGACAGAAATTGGTTGTTAGTTTGGCGGCGCCTGATCTCTTCGAACAGTATCTGCAGGTTTTTGATAAACCAGTTGAGCTGTTCAGCTTGTACCAACCAATCGCTGGCGAGGGTTCCTTGCTTTTCACCAATGGGATCGAAGGAAGGAAAAGACGGCAGGCATGGGAGAAATTTCTGAATAGGGAGAATTACAAACTGTTGTCGCCAGTGCTCACCAAGTTTGCGTCTGAACTGTCGGACAAATGGGGATCTCTACCGGCTGACCAGCACATTCCTCTGCACCAATACACAAGCGCCCTTTGCATGAAAATCTGCAGTAATATTGCATTCGGTGACCACTTCAGCGATGATAAGAACGTTGTGGAGTTCAACCGAAATTTTGAAATTTGTTGGACTGAGCTAGAAGCCAGAGTCGGTGGATCGTTGCCAGACCCC [654]

Sitalc ATGTTAGATTTTGCAATTTTTGCCATAACGTTTGTTGTTTTTCTTCTAATACTGGTTTTCTATTTATATCCTGGTTCCTCGAGCAAGAAG------ACTAACGTTCCGGGCTTGGATCCATCCGACACTGCAGATGGCAATATTCCCGACATCCTTCAGTGTGGAGGGTTCCAGCCGTTCCTGAGTGACCTCCACGCGACCTACGGAGGCATTGCTTCCTTCTGGATGGCCAGGGACCTCGTCATCTCTGTTTCAACACCTGAACTCATTAAAGAGGTTCAGCAGGCATTTAATAAACCAGAGGAACTGTTCAGCCTGTACAGTTCTGTGGTTGGGAACAGGAGTCTCCTTCATTTGAGTGGTTTGGAGGCCAGGAAGAGGAGAGAGATCTGGAACACCTTCTTGGACACT---------GACTTTGTGCTGCCAAAGGCTATCGAGTCTGTGGAAGAGTTGTCAGTAAAACTGTGTTCGTTGCCCAAAGACCAGCATATTCCCCTCTTGTGCTACACCAGAGCCCTCTGCAGCAAGATCTGCTGCCAACTCGCCTTCGGTGACTACTTTCAGGAC---GAGCAAATTCTCAAGTTTGGACGCATTTTTGAAACGTGCTGGTCTGAATTGGAAGCCCAAGTGTCCGGTTCTCTTCTGGAGGAA [645]

Sclero ATGTTGGATTTTGCTATTTTCGCAACAACTTTTGTAATATTTCTTATAATATTAGTATTCTATTTATACCCGGGATCATCGAGTAAGAAA------ACAAATGTTCCTGGCATTGATCCATCGGACTCTGCAGAGGGTAACATTGCCGACATTCTTCAGGCTGGCGGACTAGACCCTTTCCTCAAAGACTTGCACGAGCGCTTCGGTCCCGTTGCTTCCTTCTGGATGGGGAGCACCCTGGTGGTGACCGTTGAAAAACCAGAACTGCTAAAAGAGCAGCAACAGGTTTTCAATAAACCTGAAGAACTCTTCACGATATTCTCTTCGCTGGTCGGCAGTCATAGTTTGCTGACGTTGAACGGTGGCGAGGGACGCAAGAGACGGGAGATCTGGCAAGAATTTCTGAAACTA---------GACACTCTTCTAGCGCCCGCCGTAGAATTTTCCGAAGAGCTGACGGACCGCCTTTCCACCCTTCCCAAAGACCAGCATGTTCCATTGCAGTCGTACGCTAGAGCTCTCTGTATGAAAATCTGCTGCCGATTGGCGTTCGGCGACTTCTTTCAAGAT---GAGCAAGTCCTCAAGTTCAGTCGCATCTTCGAAACTTGTTGGTTAGAACTGGAGGCTCAGATGTCCGGTTCAATACCAGAAAAA [645]

Trogulus ATGTTAGATTTTGTTATTTTTGCGATCACATTTACAGTATTTTTAATAGCTTTGGTGTTTTATCTATATCCTGGATCA------AGGAAG---CTAACAACTATTCCTGGACTAGATCCTTCAGATGCCAAGGAAGGGAATATTTCGGATATTCTTCAAGCAGGAAGCTTGCCACAATTTCTGGAAACATTACATAAACGCTATGGAGAAATTTCATCTTTCTGGATGGGCACAAATTTAATAGCTAGCATTGCATCGACAGAACTCTTTGAGCAGCAGAAACATGTTTTTGATAAACCTGCCGATCTCTTTGCTCTCTATCATCCATTTGTTGGTGATGGGAGTATTTTATATGCTAATGGTGCAGAAGGCAGAAAGAAGCGCAAAATTTGGGATAAATATTTCAGCCTTCAAAATAGCAAGAGATGCATTTCTTTTTTAAAAGAGTTTGTCAGTGAACTTTCGGAACAGTGGTCTGTGTTACCACCTGATCAGCATATTCCACTTTACCAATATATGGCAGCGTTCAGCATGAAAATTTGCACTAAAATGGCCTTTGGTGAACATTTTGATGACCCAAAAGCAGTTGTAGAATTCAAGAGGAATTTTGAAATTTGTTGGCTCGAATTGGAGTCCAGAGTTTCAGGATCTGTTCCTGAACCA [654]

Ortho ATGTTAGATTTTGTCATATTTGCTGTTACCTTTGCGATATTTCTGGTGGCATTGGTATTCTACCTTTACCCCGGATCA------AGAAAA---TTGACAACCATTCCTGGACTAGATCCTTCAGATGCAAAAGATGGCAATATTACTGACATCCTACAAGCTGGCAGCCTCCCCCAATTCCTGGATACATTACATAAACGTTATGGGGATATATCTTCATTTTGGATGGGTACCAGATTAGTCATCAGTATTGCCTCGCCTGAGCTTTTCGAGCAACAAAAACATATTTTTGATAAGCCAGCTGACTTGTTTGCTCTATATCTTCCATTTGTCGGGGAAAGCAGTATCTTATATGCTAATGGTGGTGAAGGTAGAAAGAAACGAAAAGTCTGGGACAGCTACTTTAGCTCGCAAAACAATAAAACCAGTATTTCAACCTTAAAAGAGTTTGCTAAAGAACTCTTGGACCAATTGAGCCAGCTTCCAGGTGACCAGCACATTCCGCTGTTTCAGTATATGGCAGCTTTTTCCATGAAAATATGCACAAAAATGGCATTTGGAAAATATTTTGATGATCCCAAAACCGTTGTAGAATTCAAGCAGAACTTTGAAATTTGCTGGCTAGAATTAGAGTCCAGAGTTGCTGGATCAATACCTGAACCT [654]

Hespero ATGCTCGATTTCGTCATTTTCGCAGTGTCTTTTTTGGTGTTTCTCGTAGTGCTTGTATTCTATTTATATCCTGGTTCT------CGAAAA---CACACTACAATTCCAGGTCTAGATCCATCTGATGCAAAAGATGGCAATATATCCGACATCTTACAAGCCGGCAGTCTACCCGAATTCTTAAAAAACCTGCATAATCGCCATGGAGATATTTCATCGTTTTGGATGGGAACCAGTTTGGTTGTCAGTATCGGATCTACAGAATTATTCGAGCAACATTTACACGTTTTTGATAAACCAGCTGAGCTATTTGCACTATACAAGCCATTAGTGGGAGAAAGCAGCTTATTATATTCGAATGGGCCTGAAGGCAGAAAGAAACGAAGACTGTGGGAATCGTTTTTCAATGACAAGATTTTCCACTCTTCTCTGCCTATGCTTAAAGAGCAAATAAGCGAATTCTGCGGTAAATGGTCCACGTTAGACGAGGATCAACACGTCCCGTTGTATCAATACATGGTCGCATTCAGCATGAAAATATGCTCTAAATTGGCGTTTGGAGACTATTTCAACGATCCCAAAATGATTGTGGAATTTAATAGAAATTTTGAAATTTGTTGGTCCGAATTGGAAGCTCGTGTGGCCGGATCCATTCCAGAACCA [654]

Leiobunum ATGTTGGATTTCGCTATATTTGCAATAACATTTGTGATTTTCTTAGTTTTGCTGGTATTTTATTTGTACCCTGGATCA------AGAAAA---CAAACAACTGTACCTGGATTAGATCCATCTGATTCTAAAGATGGAAACATTTCAGATATCCTACAAGCTGGAAGTTTCCCAGAGTTCCTGAAAAATTTGCATGATCAATATGGTGAAATTGCCTCCTTTTGGATGGGTACCCAAATTGTGATTAGCATTGGATCCCCGAAACTATTTGAACAGCAAATTCATGTGTTTGACAAGCCTGCTGAAATGTATGCCTTATACAGACCTCTTGTTGGAGAAAACAGTATATTATATTTAAATGGTGCTGAGGGAAGAATGAAACGTCAAATTTGGGATAGTTATTTCAGTGCTGATCAACTCAATGTTTTAGTCACCCATTTATCACGACTTACTAAAGAACTTGTGGAAAAATGGTCAACTCTTCCTAAAGATCAACATATACCATTATACCAATATATGGCGGCACTCTGCATGAAAATTTGCTCTCAACAAGCTTTTGGTGACTTCGCCAATGAC---AAATCAATATTGGAGTTCAACAAAAACTTTGAAATTTGTTGGTCTGAAATGGAAGCCCAGGTAGCTGGTCAAATTCCAGAACCA [651]

Proto ATGTTAGATTTTGTCATATTTGCAATAACATTTGTGGTCTTCTTGGTATTATTAGTATTTTATTTGTACCCTGGCTCT------AGAAAACGCCAAACTACAGTACCTGGATTAGATCCATCTGATTCTAAAGATGGAAACATTTCAGATATCCTACAAGCTGGAAGTTTCCCCGAGTTCCTGACAAAATTACATGATCAATATGGAGAAATTGCCTCCTTTTGGATGGGACCACAAATTGTCATTAGTATTGCATCACCGAAATTGTTTGAACAGCAAATGCACGTGTTTGATAAGCCAGCTGAAATGTTTGCTTTATACCGGCCTCTTGTTGGGGAAAACAGTATTTTATATTTAAATGGTGCAGATGGAAGAATGAAACGTCAAATTTGGGATAGCTACTTCAGTTCTGATCAATGCAATGTTTTAATCACCCATTTGTCAAGACTAACTCAAGAACTGGTGGAAAAATGGTCTTCACTACCTAAAGATCAACACATTCCATTATATCAATACATGGCAGCCCTCTGCATGAAAGTTTGCTCCCAACTGGCATTTGGTGACTTTGCCAACGAC---AAATCCATATTAGAGTTTAACAGATGCTTTGAAATTTGTTGGACAGAAATGGAAGCTCAAGTAGCTGGTCAAATCCCAGAACCT [654]

[TICKFAST_NCHAR=693;cytochrome b5 domain-containing protein]

Ixodes ACCGCCGAGGAACTCCGCAAGTACGATGGTACCTCCGACGTGCACGGCCTCTATCTTGCGTTGTTGGGACGCATATACGACGTGCAGAAAGGCGCCGAACATTACCGGCCCGGAGGCGGCTATGCGCACTTTGCAGGTCGCGATGCTTCGAGGGCCTACGTCACCGGTGACTTCTCGGAGGCGGGCCTCACAGACGATTTGCAGGGGCTTTCAGACGAATCGCTTCACACGTTCACGCAATGGGTTGATTTTTACGAAAAGGACTACAGATTCGTAGGCAAACTTGCCGGCAGGTACTACACCAATGAGGGCGAGCCAACCGAGGAGCTGCAGAGGGTGCTGGCAGCTGTGGAGAGGGCCAAGGAGAAGGAGTACCTGACCAAGGAGGCGAGCAAGGTGTTTCCACCATGCAACTCTGAGTGGACACAGGAGGGGGGCACCAAGGTCTGGTGTAGCACACAGAGCGGCGGCATCCAACGCAGTTGGGCCGGCGTGCCGAGGCAGTTCTTCGAACCCGAGACTCAAGTGAAG------------CGCTGCGTGTGCGTGCGCACCACGGGGCCCCCCTCGGATGCCGCGGTG------------------GGAGCCAAGCACAACAACCGGGGCGACCTAGACCACCCTCAGCTGAAGGAGTATCCAGGTTGCCAGCCCACTTCGGACACCTGC [663]

Siro ACACAGGAAGAGTTGAAAAAATATGACGGGAGTCCGGATAGTTTGGGTTTGTATGTTGCTCTACTTGGAAAAGTTTACGATGTCGAAAAGGGCGCCCAACATTACAAACCTGGAGGCGGTTATTCGTTCTTTGCCGGTAGGGATGCTTCGAAAGCCTACGTTACAGGGGATTTTACAGAAGAAGGATTAACAGACGACATTACAGACTTCACAGATGATCTGGTTTACAGTCTTGTTGAGTGGCATGGATTTTATGATAAAGAATATGTTTATGTCGGAAAAGTAATTGGACGATATTTTGATAAGTATGGAAAGGCAACGGAAAACATAGCGAAAGTTCAAGAAGCTGTAGAAAGGGTTAAAGCAGCAAAACAGAAAATGGAAGATGAAAAACAAATTTTTCCACCTTGTAACTCTGAATGGGAACATGCAAAGGGAGGGAGGGTTTGGTGTTCGGAAAGTAGTGGGGGTGTTGAACGAACATGGAAAGGAGTTCCAAGGCAGTACTTCAGACCAGGAGAAAGTAATGCA------------AAATGTGTGTGTGTCAGGACAACAGGTCCACCGTCTGTTCCCATTGAAGGCAT------------TACCGATACAGAAAGCGATCGTGGAGATTTAGATAATCCTAACATGAAAGAATATGACGATTGTTCAGCAGATGCAGAATCTTGT [669]

Sitalc ACGCCGGTTGAGCTTCGCAAATATGACGGAACTGCGCATAGCTTGGGTCTTTACCTTGCTGTTTTGGGAAAAGTATACGATGTACAGTCGGGGAGTCAACATTATCGTCCAGGCGGAGCGTACGCCTTTTTTACGGGAAAAGATGCAAGCCGAGCGTACTTGACTGGTGACTTTCAGGAGAGTGGACTGACTGATGATCTGAGCGGCCTATCTGATGATGTTATTCTAGGGATGGCTACTTGGCTGGACCTGTATGAAAAGGATTATGAATATGTTGGAAAGCTTACTGGCCGTTACTATGATGCCAGTGGTAAGCCAACAAAAGAGCTGTTGAAAGTTGAAGAGGCCATGGAGAGAGGTCGCAAAGGACAAAAGAAGGAACAGGATGAAAAGCAAATCTTTCCTCCCTGTAACTCTGAATGGGACCAAGTAAAAGGAGGAAGAGTCTGGTGCACCAAACAGAGTGGAGGTATAACTCGAGATTGGATTGGAGTCCCTCGTATGTTGAAGTCCAAGCCAGCTTCTTCAGCT------AAGCCTCGTTGCGCTTGCGTCAAAACTACTGGCCCACCATCCTATGTGGCACAATTTCAACC---------TGCGACTATTGACTTGACTAGAGGAGATCTGGACCACCCTCTTCTCAAGGAGTATCCCAATTGCCCACCGGAATCTGAAAGTTGT [678]

Sclero ACTGCAGCCGAACTTCAAAAGTACGACGGATCACCTGACAGTTTAGGTTTATACCTTGCACTTTTGGGGAAAGTCTACGACGTGCAAAAAGGAAGTCAGCATTATCGGCCCGGCTCTGCATATGGCTTTTTTGCTGGTAAAGATGCTACAAGAGCTTACTTGACTGGTGAATTTAAAGACAGTGGATTAACAGACGATGTGACGGGTCTTTCCGACGACATGATTTTAAGTTTAAAATCCTGGACTGATCTTTATGAGAATGACTACATCTATATTGGTAAACTGATCGGGCGGTACTACGACGAAGCGGGTAAGGGCACTCCTGCCCTAACTGCGGTCCACCAAGCAATGGAGAGAGCCCGGAAGGGCGAGAAAAAGGAGCAAGATGAGAAAGAGATTTTCCCACCTTGCAACTCTGAATGGAGTCAAGAAAAAGGCACAAGGGTGTGGTGCACCACGCAAAGCGGAGGTGTAACTAGAGATTGGGTAGGAGTACCTCGACAGTTGATGACAAACCCCGCTTCTGCGAGCGGTGGAAAGCCTCGCTGTGCCTGCGTCAAAACAACGGGACCTCCTTCAGATGTTGCTAAGACCAAGCC---------TGCTACTATTGACATCACTCGTGGAGATTTAGACAACCCTCATCTCAAGGAATACATCAATTGCGCTCCAGATTCAATCTCCTGT [684]

Trogulus ACTGCCGAGCAACTGGGCCAATACGACGGCAACGCCGGCAGCCAGGGCCTGTACCTGGCCATTTTGGGCAAGGTGTACGACGTGCAGGCCGGAAGCCAGCATTACAAACCGGGCAGTGCCTACGGAATCTTTGCTGGAAAAGACGCCTCTCGGGCTTTTGTGACAGGTGATTTCGAAGATAAAGGTTTAATCGATGAAGTAATCGATCTTCCTGGTGAACAACTGCTTTCTTTACAGAGCTGGGCAGATTTCTACGCTAAAGATTACATTTATGTTGGTAAAGTCATAGGTCGGTTCTACGATGAGAATGGCGCTCCGACTGATTACAGTAAAAAGTACGACAAAGCTTTATCCGAAGCGAAATCCCAAGAACTGAAAGAACAAGACGAGAAAAAGATCTTCCCACCTTGTAACTCAGAGTTTAGTCACGAACTCGGTGGTAAAGTCTGGTGTACCAAGATGAGCGGAGGAAGCCAACGGGACTGGGTTGGAGTTCCCAGGAAGTTGTTTCGACCTGGCAAGTCTGATTAT------------AGGTGCGCTTGTGTCCGAAACAAGGGGCTTGCCCTAACCAGGTACGGC---------------------AAAGAGGGCAGCAGTAACGGCGACTTAGATCATCCTCATTTGAAAGAATACCCAAACTGTCGCAGCGATTCCGATACATGT [660]

Ortho ACTGCAGAGGAACTCGCGAGATATGACGGATCAGCGGATAGCCCTGGTCTCTACCTGGCTATTTTGGGAAAGGTTTATGATGTGGAAGAAGGCAGTCAACATTATAAACCTGGCAGCTCCTATGGAGTTTTTACAGGAAAAGATGCTTCCCGCGCTTTTGTTACTGGCGATTTTGAAGAGAAAGGTTTGACTGATGAAGTTGTTGACCTTTCGGGCGATCTCCTATTGGGCCTACAAGATTGGGCCGAATTCTATGCCAAAGACTACAAATACGTTGGTAAAGTTGTAGGGCGATTCTACGACCAGAATGGCGTCGGGACGGATTACAAGCTTAAATATGAAGAAGCCCTGGCCATGGCAAAGGAAGAGAAATCTAAAGAAAAAGACCTAAAGAAGATATTCCCTCCTTGTAACTCTGAGTGGAGTCATGATCTTGGTGGAAAGGTCTGGTGTTCTCCAATGAGCGGGGGTATTGAAAGAGATTGGGACGGAGTACCAAGGAAATTCTTTGAGCCTGGCAGAACTGATCCT------------AGATGCGTTTGTGTACGGAACAAGGGCCCTGCGTCAACTAATTACGGA---------------------AAAGAGGGTAGCGACAACGGAGATCTAGATCATCCTCATCTCAAAGAATACGCAAACTGTCTCAGTGATTCTAATACGTGC [660]

Hespero GGGTCAGACGAACTTAGCCAATACGATGGAAGCGCGGGAAGTCCGGGTTTGTATTTGGCCGTTTTGGGACTAGTTTATGACGTAGAAAAGGGCAGTCAACATTATAGACCTGGAAGTTCGTACGGGTCATTTACAGGTAAAGATGCTTCCCGAGCCTTTGTCACCGGCGATTTTACAGAAAAGGGCTTAACTGATGATATCATTGACCTACCAGAAGATCAGATACTTGGGATTCAAGAATGGGCAGAATTTTACGATAAGGAATACACTTATGTCGGTAAAGTGACGGGTCGATATTACGACCAAAATGGAAATCCTACCGAATACAAGCAAAAAGTGGAAGATTTGTTCCAAAAAGCATTAGAAAAGACCAAAAAAGAAACCGAAGACCGTGAAATATTCCCTACATGCAATTCAGAATGGAGCCATGACCTAGGAGGTCGAGTATGGTGCACTACCATGAGCGGTGGTACGGACCGAGGTTGGGTAGGAGTTCCTAGGCAACTTTTCACACCGGGAGTCGCTAATCCA------------AGATGTGCCTGCGTTCGAAATGACGGTCCACCATCTACGGCTCAA------------------------AAGAGCGATGGTCAAAGAGGGGACCTAGATCACCCACATTTAAAAGAATATCCGGATTGTCCTAGCGATTCCGAGACTTGC [657]

Leiobunum ACTAAGGAGGAATTAGCCAAGTACGACGGGGGAGCTGACAGTCCCGGTCTGTACTTGGCACTGTTGGGGAAGGTGTATGACGTCACTAAAGGGGCGGCGCATTACAAACCGGGAGCGGGATATTCTTTTTTCACCGGCAAAGACGCATCGCGGGCGTACGTTACTGGAGATTTCACGGAGGAAGGACTTAGCGACGAAGTTCTCGATTTAAAACCCGATCAATTATTGGGTATAGAAGAATGGACGTCTTTTTACGAGAAAGACTACGAGTATGTCGGAAAATTGGTAGGACGTTACTACGATAGCGAGGGAGAGCCTACGGATTATTTTCATAAGGTGGAATCGGCCTTGGAGCTAGCCCGGGCGACGGCCCAACAAGAGACCGACGAACGCAAAATCTTTCCTCCGTGCAACTCGGAATGGAGTCACCAGGAAGGGGGGAAGGTGTGGTGTAGTCAAATGAGCGGAGGAATAGAACGCGAATGGGTGGGCGTACCGAGAAAGCTCTACAGACCTGGTGAAGATACTTCC------------AGATGCGTTTGCGTAAAGAATTACGGGCCTCCAACGGAAGGTTTGGGTCTCAAGGCGGAACCGGAAGCTAATAATGGTAAAGGAATGGGTGACGTGGGTCATCCGCATCTTCGGGAGTACGAAAATTGTCACCCAGAGGATGTCACTTGT [681]

Proto ACGAAGGAAGAATTAGCGAAGTACGACGGAGCGGGTGACAGTCCCAGTCTGTACTTGGCAGTGTTGGGGAAGGTATACGACGTCACTAAAGGGGCGGATCATTATAAGCCGGGAGCGGGCTACGCATTTTTCACCGGTAAGGACGCGTCCAAGGCCTACGTTACTGGAGATTTCACGGAAGATGGGCTAACGGATGACGTACTCGATTTAAAACCCGACCAATTACTGAGTATTGAAGAATGGACATCTTTTTACGAGAAAGACTACGATTACGTCGGTAAATTGATAGGACGTTTTTACGACGACAAAGGGGAGCCTACGGATTATTTTTACAAGGTGGAAATGGCTCTGGAAGAGGCTCGGGCGCAGAGTCGAGAAGCCGCTGATGAACGCAAAATCTTCCCGCCTTGCAACTCGGAATGGAGCCACCAAGAAGGGGGCAAAGTGTGGTGCAGCAAGATGAGCGGGGGTGTCGAACGCGACTGGGTCGGCGTACCGCGGAAGTTGTACAAGACGGGTCAGGAGAGTTCC------------AGATGCGTTTGCGTTAAGAACTATGGACCACCCACGGAGAGTTTGGGCCTTAAAGCGGAACCGGAAGCGAATTTTGGCAAAGGAAAGGGCGACACGGGTCATCCGCACCTTCGCGAGTACGACAACTGCCATCCCGAAGAAGTTACCTGT [681]

[TICKFAST_NCHAR=870;esterase D]

Ixodes ATGGAA---------GCACTGACGTTGGTATCCAGTAACAAGTGCTTTGGCGGTCTGCAGAAAGTGTACAGTCACTTTAGCTCAACGCTGAAATGCACCATGAAGTTTGCTATCTTTCTGCCTCCGAAGTCGGAGGCGGCT---CCCGTTCCTCTGCTCTACTGGCTTTCCGGACTGACCTGCACCGAACAGAACTTTATTCAGAAGGCTGGGGCCCAGAAATATGCCAGCGAACTGTCCCTGGCGATCGTTTGCCCCGATACCAGCCCACGTGGGTGCAACATTGAGGGAGAGGAGGACAGCTGGGACCTTGGCACTGGCGCCGGGTTCTATGTCGACGCGACGCAGGGAAAGTGGAAAGAGAACTACAAAATGTACTCTTATGTCACGAAAGAGCTTCCATCTCTCATG---GAGAACTTCCCAGTTGACACCAAGAGGCAGGGCATTTTTGGACACAGCATGGGCGGTCACGGCGCCCTCACCTGCGCCTTGAAGAATCCCGGCATGTACAAGAGCGTGTCTGCGTTCGCCCCAATCTGCAACCCCGTCGACTGCCCCTGGGGCAAGAAGGTCTTTTCCAACTATTTGGGCGAT---GACCAGAAGGCCTGGGAAGAGCACGACGCCACCTGCCTCGTCCAGAAGTACAGAGGCCCTCCCCTGATGCTCCTGGTTGACCAGGGAACGGAAGACGGTTTCCTGAAAGACCAACAGTTGCTTCCGGAGCGTCTTTTGGAGGCCAGCCAGAAAAATGGTGTTGGGATCACGCTCAGAATGCAAGAGGGGTACGACCACAGCTACTTTTTCATTGCAACATTCATTGAAGATCACCTCAAGCACCATGCA---------GCTGCCTTGTGA [843]

Siro ATGGGA---------GAATTAACAGAAGTATCAAGTACAAAATGTTTTGATGGCTACCAAAAAATCTTCAAGCACGAAAGCGAAGAGTTGAAATGTTCAATGAATTTTGCCATTTATTTACCATTGGCTTCTGAAAAGACT---AAATTACCTGTACTCTATTGGCTCTCAGGACTAGAATGTACAGAACAGAATTTTATTCAGAAATCTGGTGCTCAGCGTTATGCTTCTGAGCATGGATTAATAATCGTCTGTCCCGACACCAGCCCACGAGGGTGTGGGATTGAAGGTGAAAACGACAGTTGGGATCTTGGAACGGGAGCCGGATTTTATATTGACGCCACTCAACCAAAGTGGGCCAAGAATTATCGGATGTATTCTTACGTCACTAGCGAGTTGCCCAAATTGGTGGATGCGAACTTTTCGACAATCCCTGGTAAAAAATCCATTTCTGGCCACAGCATGGGAGGACACGGTGCTCTGATTTGCGCTCTTAAGAATCCCGGAATGTATTGTAGCGTTTCGGCTTTTGCTCCAATCTGCAATCCAACTGAAGCTCCCTGGGGTCAAAAGGCATTAGCGGAATATCTCGGTGAC---GACAGAAATGCTTGGAAACAATACGATGCAACTCATTTGGTACAAAACTCTTCTGGTTTGGAACACAGCATTCTTATCGACCAGGGAGGTGCCGATATATTTAAAGAGAAAGGTCAACTATTGCCAGAAAACTTTGTTTCCGCAAGCGCAGCTAAGGGAGTCTCTGTTATCTATAATTTACGATCGGGTTACGATCATGGATATTATTTCATTGCTTCATTCATTGGCAATCATATTAAGCACCATTCC---AGTTGTTTAAGAAAATAA [852]

Sitalc ATGGTT---------AGCCTGAAGCAAATTTCTAGTACCCGTTGTGTTAGTGGTTATCAGAAAGTTTATAGTCATGAGAGCAAAGTTCTAAAATGTAATATGAACTTCTCAATCTACCTTCCTCCCCAAGCTGATAACGACCAGAAAGTGCCTGTGCTCTACTGGCTTTCAGGTTTGACGTGCACAGAGCAGAACTTTATTCAGAAGGCCGGAGCGCAGAGATATGCTGCCGAGCAGGGAATTGCCATCGTGGGACCTGACACTAGCCCCCGAGGCTGCGGAATCGAAGGGGAAGAGGATAGTTGGGATTTTGGAACAGGAGCCAGTTTTTACGTGGATGCCACCCAACCCAAATGGAAGGACCACTACCGCATGTTCTCTTACGTCACTCAGGAGTTACCTGCTTTAATCAACGACAACTTTCCAATTCAAGCAGATCGACAAGCTATTTGTGGTCACAGCATGGGAGGCCACGGAGCTCTGATCTGTGCCTTGAAGTGCCCGGGGCAGTACCGCAGCGTTTCTGCCTTTGCGCCCATCTGCCACCCGACCCAGTGCCCGTGGGGAAAGAAAGCCTTCTCGGGCTATTTGGGGGAAGAGAATAAAAGCCAATGGGAAGAGTACGACGCCACCCACCTGGTGGCAAAGTACGAGGGACCCCCGCTCACCATCCTTATTGATCAGGGCAAAGATGACCAGTTCATTGAAGAGGGTCAACTTTTGACGCAAGATTTTGTGACTGCCTGTCGGGACAACAGAATGCCACTCATATACAGACAACAGGAGGGCTACGACCATTCCTATTTCTTCATCTCTTCTTTCATCGGGGAACACATCAACCACCACGCC---AACTTCCTGAATGCGTAA [858]

Sclero ATGAGT---------AATTTAATACAAGTATCAAGTGCGCGTTGCTTTGGAGGTTTTCAAAAAGTATTCAGTCATGAAAGCAAAGCATTGAAATGCAAAATGAACTTCTCCATTTACCTCCCGCCGCAAGCTGACAGCGAAAAAAAGGCGCCAGTGATGTATTGGTTGTCTGGGTTAACTTGCAACGAGCAGAACTTCATTCAAAAGGCCGGAGCCCAGAGGTACGCCGCTGAGCACGGAATCATAATCGTGGGCCCCGATACAAGCCCCAGGGGCTGTGACATCGAAGGAGAGGACGCCAGCTGGGATTTCGGCTCCGGGGCTGGTTTTTATCTTGATGCGACCGAGGACAAATGGAAAACTAACTATCGCATGTTCACTTACGTAACGGAGGAATTGCCAGAACTGATCAAAGATAATTTTCCAGTCAGAGCCGACCGACAGACCATTTCCGGACATAGCATGGGAGGTCACGGGGCCTTGGTGTGCGCCCTGAAATGTCCCGGCCAGTACAAGAGCGTCACAGCATTTGCCCCCATCTGCCACCCGACCCAGTGCCCTTGGGGTCAAAAAGCTTTTGCTGGGTATTTGGGCGACGGGAACAAGGAATCCTGGGACCAATGGGATGCCACGTGCCTTCTAAAACAGTACCAAGGCCCGCCGCTGGATATTTTGATTGACCAGGGTAAAGAGGACAGTTTTCTGGGCGATGGGCAGCTGATGCCTCAGAGCTTCGTCTCCGCTTGCCGCGACAGCCGAATGCCCTTGGTTTATAGGCTGCAGGAGGGCTATGACCACAGTTACTTCTTCATTGCATCCTTCATTGGAGAACACATATCCCACCATGCCAAACATCTCTTCTCTGGTTGA [861]

Trogulus ATGACC---------GGTCTGAAAGAAATCTCATCGTCTCGATGTTTCGGAGGCTTACAAAAGGTTTTTAGCCATATAAGCAAGGCACTAAAATGTCAAATGAATTTTTCTATATACCTGCCCCCTAAATCTGAAGATGTT---AAAGTACCGGCTCTTTACTTTCTTTCTGGGCTTTCGTGTACGGAAGAGAATTTCATACAGAAGTCTGGGGTTCAAAGATTTGCTGCTAAACATGGCTTAATAATAGTTGGTCCGGACACCAGCCCAAGAAATTGTAATATTGAAGGAGAGGATGACAGTTGGGATTTTGGAACAGGAGCAGGCTTTTATGTAAATGCTACAGAACCAAAATGGAAAGAAAATTATCAGATGTTTACGTATGTAACAGACGAGTTGTGGACATTAATCAATGAAAATTTTCCAGTGATTGTTGATCGCCAATCGATATTTGGTCACAGTATGGGTGGTCACGGAGCTTTGATTTGTGCCTTAAAGTGTCCTGGAAAATATCGGAGTGTTTCAGCATTTGCTCCAATTTGCAACCCAATAGAGGCACCGTGGGGTAAAAAAGGTTTTTCTGGATATCTAGGTGAT---GACAAAGAATCTTGGAAGGAATATGATGCCACTCACCTAGTAAAGCAATACAAAGGGCCTCCATATAACATATTGATCGATCAGGGCGAAAGTGACAAGTTTTTGACTGAA---AACCTTCTACCTCAAAATTTTGTTAAAGCATGCGCTGAAAGCAAAATGCCAGTTGTTTACCGACTGCAAGAGGGGTATGACCACGGATACTTTTTCATATCAACTTTTATTGAAGATCATATTGAACATCATGCT---CGCATTTTGCAAAATTGA [849]

Ortho ATGGGT---------TCTATTGTAGAGGTTTCGAGTACCAAATGTTTTGGAGGATTACAAAAGGTTTTTAAGCATGCCAGTAAAGTTCTTAATTGCGAAATGAAGTTCTCTATATATCTGCCTGGAATTACGGAAGATAGA---AAAGCTCCCGTTCTATATTTCCTTTCAGGGCTTACATGTACCGAGCAGAACTTCATTGAAAAATCTGGAGCCCAAAGACATGCTGCTGAGCATGGAATAATTATAGTGGGCCCAGATACAAGTCCAAGAAATGTCAACATTGAGGGTCAAGATGAGAGCTACGATTTTGGTTCAGGAGCTGGCTTTTACTTGAATGCTACAGAACCAAAATGGAAAGAACACTACAACATGTTTTCTTATGTAACTGAAGAGCTTCCTAGCATAATCAACAACAACTTTCCTGTAATTCCAAATCATCAATCCATATTTGGTCACAGTATGGGTGGTCATGGAGCGCTGGTTGCTGCCTTAAAATGCCCAGGAAAATATCAAAGTGTTTCAGCTTTTGCACCCATTTGTAATCCCTCTGTAGGACCATGGGGAAAGAAGGCATTTTCTGGATATTTGGGTGAA---AATGAAGAACAGTGGAAAGAATATGATGCAACATATCTAGTCAAGCAGTACAGCGGTCCACCTTATAATATTTTAATAGATCAGGGCAGTGCCGATAAATTTTTGCCCAGC---GAATTGTTTCCTGAAAATTTTGTAAAAGCTTGTGCTGAGAGTAGAATGCCAGTGGTATATAGATTACAAGAGGGATACGACCACAGCTATTATTTTATATCAACTTTTATTGGAGACCACATAGAACATCATGCT---CGGATTTTGAATTGTTAG [849]

Hespero ATGCTGAAT------GCCATGAAGGAGATTTCAAGCGTCAAGTGTTTTGGAGGCTTGCAGAAAGTTTTCTCTCACGAAAGCAAAGTCCTAAAGTGCCAAATGAATTTCTCTGTTTATCTGCCACCACAAGTTGAATCGGAT---AAAGTTCCTGTACTTTATTTCTTATCAGGTTTAACCTGCACAGAACAGAATTTTATTCAAAAATCCGGGGCTCAAAAATACGCATCGGAACATGGAATAATCATTGTTGGACCGGACACTAGTCCAAGAGGGTGTGGAATAGAAGGAGAAGATGTCGATTGGGATTTTGGAACTGGAGCCGGTTTCTACGTAGATGCAACTGAACTCAAATGGAAAGAAAATTACAATATGTTTACTTATGTTACTAAAGAGCTTCCAGATCTAATTGATGAAAATTTTCCGACTCTACCTGACAAGAAATCAATTTTTGGCCATAGCATGGGAGGTCACGGAGCTCTTATTTGCGCTTTGAAATGTCCTGGAAAATACTGCAGTGTGTCTGCATTTTCCCCTATTTGCAATCCGACTCAATGTCCATGGGGAATAAAAGTGTTTAATGGTTACTTGGGTTCA---GATGAGGAAACTTGGAAAGAGTACGATGCTACATTGCTAGTAAAACAATATAAAGGACCACCGTATACTATACTAATCGACCAGGGCAAAGATGACAGTTTCCTAAGCAAA---GAACTTCTGCCACAAAAGCTTGTGGAGGCTTGTGCCCAAAATCGAATGCCAATTATATATCGATTGCAAGAGGGTTACGACCATAGTTATTTTTTTATATCCACTTTTGTTGGTGAACATATTGAACATCATGCC---AAACACCTTAAAGCCTAA [852]

Leiobunum ATGACAGGAAGTGCCACTTTAAAATTAGTTTCTAGTGCAAAATGCTTCGGTGGGTTACAGAAAATATTTTCTCATCAAAGCAAAGAACTGAAATGTTACATGAATTTCTCGGTGTATTTGCCTCCCCAGGCTGAGGATGGA---AAAGTCCCTGTGCTTTACTTCTTATCTGGTTTAACTTGCACTGAGAAAAATTTCATTGAAAAGTCTGGTTTCCAGAAATATGCAGCACAGCATGGAATCATGGTTGTTGGACCTGATACTAGTCCCAGAAATTGTAATATTGAAGGAGAAGATGAAAGTTATGATTTTGGAAGTGGCGCAGGTTTTTATGTGGATGCTACTGAACCGAAATGGAAAGAGAATTATAGAATGTTCTCATACATTACGAATGAGTTACCAGCATTAGTCAATGAAAATTTACCAGCAATAGCTGATAAACAATCAATTTTTGGACATAGCATGGGTGGGCATGGTGCATTGATTGCTGTTTTGAAATGTCCTGGTAAATATAAAAGTGTGTCAGCCTTTGCGCCAATATGCAATCCAGTTAACTGTCCATGGGGAGTAAAAGCTTTAACGGGATACCTTGGATCC---GATTCTGAAAAATGGCTAGCATATGATGCTACTAGTCTTGTGAAAGATTATAAAGGGCCACCTTATGATATTTTAATTGACCAAGGAAAAGAAGATAATTTCTTGGAAAGA---GAACTTCATCCACAAAATTTTGTAAAAGCTTGCAGTGAAAACAGAATGCCTGTCACACTTAGGCTTCAAGAGGGTTATGATCACTCCTACTACTTTATCGCTACATTTATGCAAGATCACATTGCACATCATGCC---AAATATCTGTTGTGTTGA [858]

Proto ATGACTGGAAATTCCACAATAAAATTAATTACAAGCGCGAAATGCTTCGGTGGGTTCCAGAAAATATTTTCACATCACAGCACGGAACTGAAATGCGACATGAATTTCTCTGTATATTTTCCTCCCCAAGCTGAAGATGGG---AAAGTTCCAGTGCTTTATTACTTATCTGGTTTAACTTGCACCGAGAAGAATTTCGTCGAAAAATCTGGCTTTCAAAAATTTGCAGCTCAACATGGTATCATGGTTGTTGGACCCGATACCAGTCCTAGAGGGTGCAATATTGAAGGAGAAGCCGAAAGCTACGATTTTGGAAGCGGTGCGGGGTTTTACGTCGACGCTACCGAACCAAAATGGAAAGAGAACTATCGGATGTTTTCCTACGTTACCAAAGAGTTGCCAGCTTTAGTCGTGGAGAATTTACCCGCAATTGCTGATAAACAGTCAATTTTCGGACATAGTATGGGTGGACATGGCGCGCTGATTGCCGCTTTAAAATGCCCCGGCCAATATAAAAGCGTGTCAGCTTTTGCGCCTATCTGCAATCCGACCAAATGTCCCTGGGGAATCAAAGCGTTCACTGGTTATCTTGGTTCT---GACGCACAAAAATGGGAAGAGTTCGATGCGACTTGCCTCGTAACGGATTACAAAGGACCACCTTATGACATTTTAATCGACCAAGGGAAGGAAGATAATTTCTTGGAAAAA---GAACTCTTCCCTCAAAATTTCGTTAAAGCTTGCAGCGAAAATAGAATGCCCGTTACGCTTAGGCTTCAAGAGGGTTACGATCATTCGTATTTTTTCATAGCTTCGTTTATGGAAGATCACATAGCCCATCATGCA---AAATATCTTTTTGCTTAA [858]

[TICKFAST_NCHAR=2076;eukaryotic translation initiation factor 3 subunit C]

Ixodes TATGAAGAGATTACGGAGATAATCAAACAAATCAAGAACCACAAGAAAATAAAAGACATGAGCAAGCTCTTAACAAGCTTTGAGAACCTCACCAAGGCGTTTCAGAAAGCCAAGATGGTGATAGAGAAGGAGGAAGGGGGCAACATCCCCAAGTTCTACATCAGGAGCCTGGTGGAGCTGGAGGACTTTGTGTCGGAGTGCTGGGAGGACCGCGAGGGCCGCAAGAACATGTCCAAGAACAACTCCAAGTCCCTGGCGACGCTACGGCAGAAGCTGCGCAAGTACAGCAAGGACTACGAGGCGGACATCACCAGGTACCGAGAGAACCCTGAGGAGGAGGACGAAGAGCGGGACGAGGACGACGAA------AAAGACTCTGATGATGACGACGACATGAGCTTCAAGAAGAAGCCAGCTGCCAAG------GTCGAGAAGGATGAACCCGCTTCCAAGTTCATGAAG---GGCGGCTCTGACGACGAGGAATCGGACGACGACTCCATGGACTGGGGTTCCTCCGAC---GAGGGTGAGAGTTCTCTGTCCAGTGACGACGAGAAGTACGGCGGGAACCTGGCCATCAAGTTCCTCAAAAAGGCCGGAGGTGACAAGGACGAGCAGAAGGAG---CAGCGCCGGCGGGAGAAGAAG------CGTGAGCAGAAAGACAAGCGCAGGGACGACGACGAGGAGGGCTGGGAGGAGGTCAAGGGGGGCGTGCCCCTCATCATGGAAAAGCCCAAGATGTTTGCCAAGGATGCAGAGATCAACCACGCGGTGGTGCTGAAGAAGCTGAACGAGATCCTGGCAGCCAGGGGCAAGAAAGGCACGGATCGGTCTGAGCAGATTGAGCTGCTCAGCGAGCTACTGACCATCTCCCAGGCACACAACCTGGGGCCGGGCTTCCAGCTCAAGATCATCTTTGCCGTCGTGTCGGCCATCTTCGACTACAACCCCAACATCGCCACCTGCATGAAGCACGACATGTGG---CAC------AATAGCAACGCTTTGCAACAGGGTGTTGAAACCGGCAGAGATTTG---GTTCTGTGGCTAAGTGTGCGGCGGGAGCTTTCACCTCGCACGCCGTTCAGCTGCAGTGCGTTGGCACAGCCGGCCACCACGCTTCCCTTCGGAATGGGCCCTAACAGTGGGTCGCCGCTTCCCCAGGGTTGCGACGCCCACTCGCCCGAGTACGTGGAGCGTCTGAAGGACGAGAAGAATGTGTGCCGCATCATTGAGCGCCTCCAGAGCTACCTC---GAGGGCAGAGGCTCTGCCTCGGAGCTCTGCCGCATCTACCTGCGCCGCATTGAGCACCTCTACTACAAGGATGAGGAGGGAGTCC------------------------------------------------------------------------------------------------------ACCGGGCAGAGCGGGTGGCTCCG---------------ATGCGCTCCACGCTGAACAGCCTGGAGCAGATGGAGCACCTGTGCAAGTACATCTACGCCAAGGACAGCACGGACCGGATCCGGACACAGGCCATCCTGTGCCACATCTACCACCACGCCCTGCACGACAACTGGTACCAGGCCAGGGACCTGATGCTTATGTCCCACCTGCAGGAGAGCATCCAGCACTCGGACGAATCTGACTGGCTGCTTCACGGAAGAAGTGTGCAGCACCTCTATCGAGCT------CGGGGCAATTTCGACGCACGGCTTCGGACCCGAAATTCGTCGCTGTCTTGGCGTGACAAGGTGCCGGGAAACTGCAAAACTGGCCTGTTGCCCCAGCGCCAGCACGAGCGCACCTCCGAGCAGGAGAAGATTGAAAAGCGCCGCCAGATGCCCTTCCACAAGCACATCAACCTGGAGCTGCTCGAGTGCGTCTACCTGGTCTCGGCCATGCTGCTCGAGATCCCACAGATGGCCGCTCACGAGTTAGATGGTCGCAAGAGGATGATCAGCAAGTCATTCCACTACCAGCTACGCAACACGGAGCGCCAGCCTCTTGTTGGT [1911]

Siro TATGAAGAGCTCACAGATATTATTAAACTCATAAAAAACCACAAGAAAATTAAAGATATGAGCAAACTATTGACAACATTTGAGAATCTGATACGAGCTTTGCAAAAGGCAAAGCTGATTATTGACAAAGAAGAAGGTGGCGTGACTCCCAAATTTTACATCCGTTGCCTCGTTGAGTTGGAGGACTTCATTAATGAGTGCTGGGAGGACAGGGATGGTCGCAAGAACATGTCTAAAAATAACTCCAAGTCGTTGGCAACGCTGAGGCAGAAATTACGCAAATACAACAAAGACTTTGAGAACGATATTAATGTCTATCGAGATAACCCTGATTTGGAAGATGATGAGAAAGAGGATGAAGAAGAA------GCTGAATCGGAAGAAGAAAAGGAAGATTTTTTCAAGAAAACA------CCAAAG------GAAGAGAAAGAGGAACCAAAATCCAAGTTTTTGAAA---GGTTCCGATGATGAGGAATCTGACGATGACGATTCAATGGACTGGGGGTCGTCG---TCAGAAAGCGAGACGTCCTCCTCCAGCGACGACGAGAAATACGCCGGAAATTTGGCCGCCAAATTCTTAAAAAAGACGACG---GACAAGGACGAACAAAAGGAGAAGAAACGA------GAAAAGAAGCGAGAGAAAGCCAAGGATCGTAGGAGAGACGATGAAGATGAAGGCGAATGGGAAGAGGTCAAAGGTGGAGTCCCGCTCATTATTGACAAGCCGAAAATGTTTCCAAAGGATGCCGAGATTAACCACCCGGTGGTAGTGAAGAAGTTCAACGAAATTTTAGCTGCAAGAGGCAAAAAAGGAACCGACCGAAGCGAGCAGATCGAACTCTTGAACGAACTGCTTTCCGTTTCCAATGTCAACAACCTGGGTCCCGCCATGGAAGTCAAGATTATGTTTGCAATTGTCGCAGCCGTTTTCGATTACAATCCCAACATTGCAGCTTGTATGAAGTCAGAGATCTGGCAAAAGTGTTTGGATTACATCAACAGTCTTTTGGACCTTCTTATGGACAATCCATCG---ATTGCTGTTGGCGAACACATTGCCGAAGAGTCGGAAATCATTGTGGCGCCACCCTACAGGGTCAGGGGTTGCATCCTGACTGTGATTGAACGCATGGACGAAGAATTTGTCAAGATGCTT---CAGGGTTGCGACGCTCATTCTCCCGAGTACGTGGAGCGACTCAAAGATGAAATCAGTGTGTGTCTAATCATTGAGAGGTTGCAAGAGTACATG---GAAGGAAAAGGCACCACATCTGAGCTTTGCAGAGTTTACCTCAGACGCATCGAACACATTTATTACAAGTTCGACGCCAACGTCTTCAAGCGGCGAGAAGCG------------------------------------------TTGTCCAAAATAACGAAA---------------------CACGATGGCGAGAAGCAAGATCTTCCC---------------GAACTACCTACTGAAACCAGCATTGACGTCATCGATCAGTTGTGCAAATACATCTACGCCAAGGACAATACGGACAGAATACGAACCCAGGCTTTGTTGTGCCATGTCTACCATCATGCGCTTCATGACAACTGGTTCCAGGCTAGAGATCTCATGTTGATGTCTCACTTGCAAGATACAATCCACCATTCTGATCCTCCAACTCAGATCCTGTACAATCGCTCCCTTGTGCAGTTGGGTCTTTGCGCATTTCGTCACGGAAACATTAAAGATGCCCACAATGCCTTGCTGGACATCCAGAGCGGTGGTCGGGCCAAGGAATTGATAGCCCAGGGGCTGCTGCCCCAGCGTCAACACGACCGCACAACAGAGCAAGAGAAGATCGAAAAGCGTCGTCAGATGCCGTTCCACAAACACATTAATCTGGAACTTCTAGAATGCGTCTATTTGGTTTCTGCTATGCTTCTAGAAATCCCATACATGGCTGCCCACGAGTTGGACGGGCGCAAACGGATGATCAGTAAATCATTCCATCACCAATTACGTAACAGCGAGCGCCAAGCCTTGATTGGT [1956]

Sitalc TTCGAAGACATCTTTGAAATTATTAAGCTTATTAAGAATCACAAAAAGATTAAAGACATGAGTAAGCTTCTAACTTCTTTTGAGAACTTAACCCGGGCGTTACAAAAAGCTAAACCGGTCATTGATAAAGAAGAATCGGGTGTTACACCGAAATTCTACATACGATGTTTAGTCGAGCTGGAGGACTTCATAAGTGAATGCTGGGAAGATCGGGAGGGCCGCAAGAACATGTCCAAAAACAACTCCAAGTCGCTCGCAACATTGCGACAGAAACTAAGGAAGTACAACAAAGACTTCGAGAAATACATCAGCGATTATCGTGAGAATCCTGAGGCCGAAGAAGAGGAGAAAGAAGACGAAGAGGAA---CAGCTATCCGATGATGCTGACTCTGACGACGACTTCAAGAAACCAACC---ATTAAA------GCTGAGCGAGAAGATACAAAATCAAAGTTCCAGAAA---GCTTCG---GATGATGAGGATTCTGACGACGATTCGATTGACTGGGACATGTCCCACTCTGAGAGTGACAGCTCATCATCCAGTGATGACGAGAAGTACGCCGGTAACATGGCTGCCAAGTTCTTGAAGAAATCAACG---GACAAAGATGACGCAGGAGAAGGAAAGAAA---------AAGAAACGA---GGTGGAGAGAAGAAGAAGAAGAGAGATGACGATGAAGGCGAATGGGAGGAGGTCACTAAAGGTGTCCCACTTGTGGTGGAGAAACCAAAGATGTTCCCGAAGGACGCTGAAATTAACCATCAGTTAGTTTTGAAGAAGTTGAACGAGATCATGGCTGCCAGAGGAAAGAAAGGTACTGACCGTAACGAACAGATAGATCTTTTAACGGAACTGATGAACATTTCCAACGCCAACAATTTGGGCCCTGGAATGGAAGTTAAGATTTATTTTGCCGTTGTGGCAGCCATCTTTGATTACAATCCGAATATTGCGACCTGCATGAAAGGAGAAATGTGGCAGAAGTGCTTAGAGACCATTCAGACTTTGATTGATATTCTGGAAGAACACACGACC---ATTACGGCAGGAGAACACATTTCGGAAGACTCCGAACAGCTGAATAAGGCACCATACCGTGTACGTGGATGCATTCTGACTGTCATAGAACGAATGGATGAGGAGTATACTAAGATGCTT---CAAGGTTGTGATGCTCATTCACCCGAGTATGTAGAACGTCTAAAAGACGAGAAGACAGTATGCTTGATTATCGAGAATCTGCAGAACTACCTA---GAAAAGCGTGGCACATCCTCGGAGCTTTGCAGAGTTTACTTGCGACGTATTGAACATATTTACTACAAGTTCGATCTGAGTGTGTTCAAGCGCCAACAGATC---------------------------AGCGAGCATACAGAGAAAGAAGCCAAACCAGAG------------------GTTACTGAAGAGGAAAAGCAAGATTTACCC---------------GAATACCCCCAGGAGACGAGTCTCGAAGTTATGGAGAAGCTCTGCAAGTTTGTCTACGCGAAGGACAACACTGATCGTATTCGCACACAAACCATGCTTTGCCAAATTTACCACCTTGCTCTTCATGACCACTGGTACGAAGCTAGAGATCTAATGCTGATGTCTCATCTTCAAGATTCTATTCACCACGCTGATCCTCCCACTCAGATCATGTACAATCGTGCGCTGGTCCAACTTGGAATATGTTCATTCAGGCACGGAAACATCAAAGATGCTCACAATGCCCTTCTTGATATCCAGAGCGGAGGACGCGCAAAAGAACTTCTTGCCCAGGGACTTCTACCGCAGAGGCAGCACGAACGAACAGGAGAACAAGAAAAGATTGAAAAACGTCGTCAAATGCCATTCCACAAACACATTAATTTGGAGCTCTTGGAGTGTGTATATCTTGTTTCTGCCATGCTGCTGGAGATACCCTATATGGCTGCCCACGAACTGGAGGGCCGCAAACGCATGATCAGTAAGTCTTTCCATCACCAGCTGCGTAACAGTGAACGCCAGGCCTTAGTTGGA [1974]

Sclero TATGAAGATATTTTGGAAATCATCAAACAAATTAAAAATCACAAGAAAATCAAAGACATGAGTAAATTATTAACAACTTTTGAGAACCTGACCCGAGCACACCAGAAAGCCAAACCGGTTATTGACAAGGAAGAGTCCGGAGTCACGCCCAAGTTCTATGTCCGGTGTCTCGTGGAACTGGACGATTTCATTAATGAGTGCTGGGAGGACCGGGAAGGTCGTAAGAACATGTCCAAGAACAATTCCAAGTCCTTGGCCACCCTGCGGCAGAAGCTGCGCAAGTACAACAAGGACTTTGAGAAATATATCTCGGACTACAGAGAGAACCCTGAAGCTGAGGAAGAGGACAAAGAAGAGGACGAGGAAGCCGGGGTTTCTGAGGGTGATGATTCTGACGATGACTTTAAGAAGCCAGCAGTAGCCAAAGTGGATCGGGAGGGAGGAGAAGCCAAGGCCAAGTTCCAGAAG---GGCTTTATGGATGATGAGGAATCCGATGATGATTCCATTGACTGGGAGATGTCCCATTCCGAGAGTGACGACTCTTCATCCAGTGATGACGAAAAATATGCTGGGAATATGGCTGCCAAATTCTTGAAGAAGTCGACA---GACAAGGACGATGCCGGGGAAGGGAAGCGC---------AAGAAGAGG---TCTGAC---AAGAAGAAGAAAAGGGAGGACAGTGATGGAGAGTGGGAGGAAGTCACCAAGGGAGTCCCCCTGGTGGTGGAAAAACCCAAAATGTTTGCTAAAGATGCAGAGATCAACCACCAGCTGGTTTTAAAGAAACTAAATGAGATCATCGCCGCTAGGGGTAAGAAGGGAACAGATCGCAGCGAACAGATTGACCTCCTGACGGAATTGATGACTATCTCCAATGCCAACAACTTGGGTCCAGGAATGGAAGTGAAGATCTACTTTGCCATTGTGGCAGCCATCTTTGACTATAACCCCAACATCGCCACCTGCATGAGAGGGGAAGTCTGGCAGAAGTGCTTGGAACACATCCAGATTCTGCTCCTGATCCTAGATGAGAATGCCAGC---ATCACGGTCGGGGAGCACATTGCGGAGGACTCTGAGCTTATCAGTGCAGCCCCTTACCGCATCCGAGGATGCATCCTGACAGTGGTCGAACGCATGGACGAGGAATACACCAAGATGCTG---CAGGGTTGTGATGCCCACTCCCCAGAATATGTGGAACGGCTAAAAGATGAGAAGACTGTTTGCGAGATCATCGAACACCTGCAGAACTACTTG---GAGAAGCGGGGTACCTCCTCAGAGCTCTGCAGGGTTTACCTCAAGCGCATTGAGCACATCTACTACAAGTTCGACTTGAGTGTGTTCAAGCGCCGTGAGATC------------CAGGACGGCCAGTCGCGGAGGTGTTCCGCAACAGAAAGCAAGCAGCCCACGCCAGAGGAAGAAGAGATCGGAGAGGAAGAGAAGAAGGACCTTCCA---------------GAATACCCGTCCGAGACCAGCCTGGAAGTCCTGGAGATCCTGTGCAAGTTTGTGTATGCCAAGGACAACACGGACCGCATCCGCACCCAGGCCATGCTCTGTCAGATTTATCACCTGGCCCTGCATGACCATTGGTATCAAGCCAGGGACCTCATGCTCATGTCCCACTTGCAAGACTCCATTCACCATGCCGATCCACCCACGCAGATCATGTACAACCGAGCTTTGGTTCAACTGGGCTTGTGCTCCTTTCGGCATGGCAACATCAAGGATGCTCACAACGCCCTGCTGGACATTCAAAGCGGCGGAAGGGCAAAAGAACTCCTTGCACAGGGCTTGTTACCCCAGAGGCAGCACGAGCGCACGGGGGAACAGGAGAAGATTGAGAAGCGTCGGCAGATGCCCTTCCACAAACACATCAACCTGGAACTGCTGGAGTGTGTCTACCTTGTCTCTGCAATGCTCTTGGAGATTCCTTACATGGCCGCCCATGAACTAGAAGGGCGAAAACGTATGATCAGCAAATCATTCCACCACCAGTTGAGAAACAGCGAGAGGCAAGCTCTAGTTGGT [2019]

Trogulus TTTGAAGATCTGATGGAAATCATCAAACAAATTAAAAACCACAAGAAGATAAAAGACATGAGTAAACTGCTAACGAGTTTTGAAAATTTGACCAAAGCCTTGAACAAGGCCAAGCCTGTCATAGATAAAGAAGCAGCTGGAGTCATTCCAAAATTTTACATTAGGTGTTTGGTTGAACTAGAAGACTTTATTAATGATTGCTGGGAAGACAGAGAAGGTCGTAAAAGCATGTCCAAAAACAATTCAAAATCACTGGCCACTCTAAGACAGAAACTTCGTAAATATAATAAAGATTTTGAGAGCCAAATTAGTGAATATCGAGAAAATCCAGAAGCTGAAGATGAGGATCGAGATGATGATGAAGAG------AGTGGAAGTGAAGATGATGATGAC---AGTGAAATA------------ACAAAA------GATGATAAGGAAGATGGAAGAGGAAAAATTTTAAAACCAGGATCAGATGAGGATGAAGATTCAGAAGCCGACTCATTCGACTGGGGTTCATCTGACTCGGAAAGTGAAAGCGAGCTCTCTTCCGATGATGAAAAATATGGAGGTAACTTGTCCATGAAATTTCTCAAGAAGCCAAGT---GACAAGGACGAAGTTGACACAAAAAAGAGA------GACAAGAAAAGA---AGCAGTGATAAGAAAAGCCGAAAGAGAGACGAAAATGATGGTTGGACAACTGTTAGAGATGGCGCTCCACTTATCATTGAAGTTCCAAAGATGTTTGCTAAGGATGCAGAGATTAATCACGGTTTAGTACAGAAGAAACTGCAAGAAATCTTGGCTGCGAGAGGCAAGAAAGGAACTGATAGAAGCGAGCAGATAGAGCTACTTGGCGAGCTGCTCAACATTTCTAAAACTCACGAACTTGGCCCTGCAATGGAAGTCAAAATAATCTTTGCTGTTGTAGCTGCTATCTTTGATTATAATCCCAACATTGCTACTTGCATGAAAACTGAAGTTTGGCAGAAATGTTTAGAAAATGTAAACCTCCTCTTGGATCTCTTGACAGTGAACTCTCATCATTTAACTGTTGGAGAGCATGTGGCTGAAGACTCTGAAGTTCTTGCTGTGACTCCTTATCGTGTCAGGGGATGCATACTGACCGTGGTGGAAAGACTTGATGAGGAGTTCACCAAGATGTTG---CAAGGCTGTGATGCCCACTCTCCAGAATACGTGGAACGGCTTAAAGATGAGAAGACTGTTTGCAACATTATACAGCGTTTACAAGACTATTTG---GAAGGGAAAGGAACTTCTTCAGAACTATGCCGAATTTACCTACGTAATATTGAACACATTTATTACAAATTTGATTCAAGAGTTTTGGAACAACGAGAGCGT------------AAGAAAAATTTAACAGTTGTAGGAGGGATGTTGTCAGCAGCAAATGAAAATGAAACTGAAAAGAAAGATGAGTCTCCAGAAGAGAAGGCTATTGAA---------------ATAACTGAAACGGAAACTTCCTTGGATGACATGGCTCGGCTATGTAAGTTCATCTACGCAAAGGATAATACAGACAGAATAAGAACACAGACCATGCTGTGTCACATATACCACCATGCTTTGCATGACAACTGGTTTGAAGCTAGAGATTTGATGTTGATGTCTCATTTACAAGATTCCATTCACCATTCTGACCCACCCACACAGATTTTATATAATCGTGCACTGGTCCAGTTAGGCCTTTGTGCTTTTCGTCATGGCAACATTAAAGATGCTCATAATGCTCTGTTGGAGATCCAAAGCGGCGGCCGGGCTAAAGAGCTTCTGGCACAAGGCCTTCTACCACAGCGTCAACACGAGCGTACAAGTGAGCAAGAGAAAATAGAAAAGCGTCGCCAGATGCCTTTCCACAAGCACATAAATTTGGAGCTCTTGGAATGTGTCTATTTAGTATCTGCCATGTTACTGGAGATCCCTTACATGGCGGCTCACGAGCTGGAAGGTCGCAAACGCATGATAAGCAAATCTTTTCACCATCAATTGCGTAATAGTGAACGTCAAGCGCTTGTTGGG [2004]

Ortho TTCGAAGACCTTGTTGAGATCATTAAACAGATTAAAAACCACAAGAAAATTAAAGACATGAGCAAGCTCTTGACAAGTTTTGAAAATCTGACGAAAGCTCTGAACAAGGCGAAACCTGTGATCGATAAAGAGACAGCTGGAGTTATTCCAAAATTCTACATTCGATGCCTGGTTGAACTTGAAGATTTCATCAATGAGTGTTGGGAAGACAGAGAAGGACGTAAAAGCATGTCCAAGAACAACTCAAAGTCTCTAGCCACCTTAAGACAGAAACTCCGTAAATACAACAAAGATTTTGAGTCTCAAATCACAGACTACAGAGAAAACCCGGAAGCTGAGGATGAAGAAAGAGACGACGATGAGGAG------AGTGGAAGTGAAGATGATGACGACGAAAGTGAAATG------------GCCAAA------GATGAAAAAGACGAAGCAAGGGTAAAATTTGCCAAA---ACCGGATCAGAAGATGAAGATTCTGATGCAGATTCATTTGACTGGGGTTCCTCCGGCTCAGAAAGTGAGAGTGAACTCTCCTCTGATGATGAGAAATACGGTGGTAATCTGGCAATGAAGTTTTTGAAGAAATCAACT---GATAAAGATGATGGGGATACCAAAAAAAGG------GAAAAGAAAAGA---ACCAGTGACAAGAAGAGCCGCAAGAGGGACGAGGATGACGGTTGGACTCGCGTTAAAGATGGAGTTCCACTCATCATTGAAGCACCCAAAATGTTCGCTAAAGATGCAGAGATTAATCATGGTCTAGTACAGAAGAAATTGCAAGAGATTCTAGCAGCTAGAGGCAAGAAGGGAACAGATCGCAGTGAGCAGATAGACCTCCTGGGAGAATTGCTAAACATCTCCAAGGCTAATGACCTTGGCCCAGCAATGGAAGTCAAGATCATATTCGCTGTAGTTGCTGCCATTTTTGATTATAACCCAAACATAGCAACTTGCATGAAGACAGAGGTATGGCAGAGGTGCCTGGAGAATGTAAATCTCTTGCTGGATCTCCTATCAGAAAATGCTCATCATCTAAGTGTTGGAGAACATGTCGCTGAAGATTCAGAGACCCTGGCAACTGTACCCTATCGCGTCCGAGGATGTATCCTGACGGTAGTAGAACGACTGGATGAAGAATTCACTAAAATGCTG---CAAGGATGTGATGCTCATTCCCCGGAATATGTGGAGAGATTGAAAGATGAGAAAACAGTCTGCGCCATAATTGAACGTCTGCAAGACTACCTT---GATGGAAAGGGAACTTCCTCCGAGCTGTGCAGAGTTTACCTCCGGAACATTGAACACATTTACTACAAATTTGATCCCAAGGTTTTGGAACAGAGGCAGCGT------------AAAATGAACCTTTCAATTGTAGGAGGAATGATTTCCGCCACAAGCGAA---------------------AGCGAAGAAACTCCAACACCAGCTCCT---------------GAAGAGACCGAAACAACATCTCTAGCCCAGATGGATCATCTTTGCAAGTTCATCTACGCGAAGGACAACACTGACAGAATCAGGACACAGACCATGCTCTGCCATATCTACCACCACGCTCTACATGACAACTGGTTTGAAGCCAGAGATTTAATGCTCATGTCTCATCTGCAAGATTCCATTCACCATGCTGATCCCCCAACCCAGATCCTGTACAATCGTGCCTTAGTGCAGCTTGGCTTATGCGCCTTCCGCCATGGTAACATCAAGGACGCCCATAATGCACTCCTTGAGATTCAAAGTGGAGGTCGCGCCAAGGAACTCCTAGCTCAAGGCCTGCTCCCACAGCGCCAACACGAGCGAACTAGCGAACAAGAAAAGATTGAAAAACGTCGCCAGATGCCCTTCCATAAACACATTAACTTGGAACTATTGGAATGTGTGTACCTTGTCTCAGCCATGTTGTTGGAAATCCCTTACATGGCGGCACACGAATTAGAAGGACGTAAACGTATGATCAGCAAGTCTTTCCATCATCAGCTCCGAAACAGTGAGCGCCAGGCTCTGGTTGGA [1983]

Hespero TTCGAAGATTTGGTGGAGATAATCAAACAAATCAAGAATCACAAGAAAATCAAAGATATGTCCAAGTTGCTAACGAGCTTCGAGAATCTAACGCGAGCGTTCAATAAAGCCAAACCGGTCATAGATAAAGAAGAAGATGGAGTTACGCCCAATTTCTATACCCGATGTTTGGTCGAATTGGAGGATTTCATCAACGAATGCTGGGAGGATAGAGAAGGACGCAAAAATATGTCCAAGAATAATTCAAAGTCTTTGGCTACGTTGCGCCAGAAATTGCGCAAATATAACAAAGATTTCGAGGCGTTGATAGGAGCCTACAGAGAAAACCCAGAGGTAGAAGCGGAGGAACGCGAAGAGGAAGAAAGC------GGTTCCGAAGATGACAAAGATAGCGATTACATAAAGAAACCCGCA---GTTAAA------GAAGAGAAAGAGGATGTTAAAGCCAAGTTTCTGAAA---GGTTCC---GATGACGATGACTCCGATGCTGATTCGTTCGACTGGGGGTCATCCGACTCCGAGTCGGAGAGCGAGCTCTCCAGTGACGATGAGAAATACGCGGGTAATCTAGCCGCAAAATTCTTGAAGAAATCGACC---GATAAAGAAGACGGAGATATCAAGAAAAGCAGG---GACAAGAAACGA------GACAAGAAATCTAAGAAACGGGATCTGGACGAGGATGGTTGGAAGCAAGTCGTTGGAGGCGTACCGTTA------GAAAGGCCTAAAATGTTCGCTAAAGATGCCGAAATCAATCATCAGCTTGTCTCCAAAAAACTGACCGAGATCTTAGCGGCCAGGGGCAAGAAAGGAACCGATCGTAACGAGCAATTGGAACTGCTAGGAGAATTGCTGAGCATCTCTAAAGCTAACGACTTGGGACCCGCTATGGAAGTGAAGATCATTTTCGCCATTGTAGCGGCCATCTTTGATTATAATCCCAACGTAGCTACGTGCATGAAGACAGACACCTGGCAGAAATGTCTCGATAATGTCAATATATTGCTCGATTTGCTCATTGAGAATAATACT---CTGGTCGTTGGGGAACACGTAGCTGAGGATTCGGAGGCTCTGGCTGTGCCGCCATACAGGGTTAGAGGGTGCATTCTGACTGTGGTTGAAAGATTAGACGAAGAATTTACTAAGATGTTG---CAAGGTTGCGACGCCCATTCCCCCGAATACGTAGAACGGCTCAAAGATGAGAAAACAGTCTGCAATATGATCCAACGTCTTCAAGATTATCTAGTCGAAGGCAAAGAGTCTAGTTCCGAGCTATGCAGAATCTATTTACGCAACGTAGAGCACATCTACTACAAATTCGACCAGAATGTCTTGAAGCAGAGAGAGGTTATACAGAAGGAGAATTGCTCTATTATCAGCGGTATGATTCAAAGTCCCGGCACGGAGAAAATAATACCGGCCAAACCTGCGCCACCTGTTGAAGAGACAACAGCTCCCGCTACCGAAGAAACCCCCGAAATTAAGGAGACTAGCATCGACACAATGGATTTCCTCTGCAAATTCATCTACGGGAAAGATAACACGGACAGAATCCGTACCCAAGCTATGCTATGCCACATCTACCATCACGCTTTGCACGATAATTGGTTCGAAGCCAGAGATCTGATGCTAATGTCTCATTTGCAAGATTCTATTCATCATTCCGATCCTCCTACTCAGATTCTGTACAACAGGGCTTTGGTACAGCTCGGATTATGCGCATTCAGACACGGCAATATCAAAGATGCCCATAATGCTCTGTTGGAGATACAGTCCGGAGGAAGGGCAAAAGAGTTGTTGGCTCAAGGGTTATTGCCTCAGAGACAACACGAGAGGACCGGAGAGCAAGAGAAAATTGAGAAAAGGCGGCAAATGCCCTTTCATAAGCATATTAATCTGGAGTTGTTAGAATGCGTTTATCTAGTGTCTGCTATGCTGTTGGAGATTCCTTATATGGCGGCTCATGAATTAGAAGGCAGGAAGAGGATGATTAGCAAGTCTTTTCATCATCAGTTGAGGAATAGCGAGAGACAAGCTTTGGTAGGA [2031]

Leiobunum TATGAAGAGTTAGTTGATATTATCAAACAAATCAAGAACCACAAGAAAATCAAGGATATGAGTAAACTTCTTACCAGCTTTGAGAATCTTACAAGAGCATTAAACAAAGCTAAGCTTGTAATAGAAAAGGAAGATGGTGGCATCACTCCTAAATTTTACATCCGTTGCTTGGTTGAATTGGATGATTTCATCACAGAATGTTGGGAAGACAGAGATGGTCGCAAAAATATGTCCAAGAATAACTCAAAGTCCCTAGCTACATTACGTCAAAAACTAAGAAAATATTGTAAAGATTTTGAAACTGCTATCAATGACTACCGTGAAAATCCAGAGGCTGAAGAAGATGAAAAAGAGGAGGAAGAAGCA------TTATCTGATGATGATAGAGATAGTGATTATTCAAAAGCCCCTGTTGCAGTCAAA------GAGGAAAGAGAAGAGGTTAAATCCAAATTCTTGAAG---GGATCA---GATGATGAAGAGTCTGATGATGATTCCATTGATTGGGGTTCATCAGATTCGGAGACGGAAAGCGAATTATCCAGTGATGATGAAAAGTATGGAGATAATTTGGCTGTTAAGTTCTTGAAGAAACAACCTGGTGACAAGGAAGAAAAGGAAGTAAAGAAACGA------GAGAAAAAGAGA---------------GATAAAAAGGGAAAAAAGCGAGATGGAGATTGGGTTGATGTTAAAGGTGGAATTCCAATAGTTGTTGAGAAACCAAAAATGTTTGCTAAAGATGCAGAAATCAATCATCAGCTTGTACAGAAGAAGCTGGTTGAAATTCTAGCTGCTAGAGGGAAAAAGGGAACTGATAGAAGTGAACAAATTGAACTTCTTGGAGAATTACTTAGCATTTCTAATGCCCACGAGTTAGGAGCTGGAATGGAAGTGAAGATCAGATTTGCCATTGTTTCAGCCATTTTTGATTATAATCCCAATATCGCAACTTGCATGAAAACTGAAGTTTGGCAGAAATGCCTAGAAAATATAAACATACTTCTGGACCTGCTTATAGCCAATTCCACC---CTTATAGTTGGAGAACATGTTTCTGAGGACTCTGAATCCTTGGCTGCTCCACCATACAGAATAAGGGGATGCATCTTGACTGTTGTAGAACGTTTGGATGAAGAATTCACAAAAATGTTG---CAAGGTTGCGATGCTCATTCTCCAGAATATGTTGAGCGGTTGAAGGATGAAAAAACAGCGTGCAACATCATTGAAAGATTGCAAGCCTACTTG---GAGGGAAAAGGAACATCTTCAGAACTATGTCGCATCTACCTAAGGCGCATTGAACACTTGTATTACAAATTTGATCCAAATGTTCTTAAAAGAAAAGTTCAT---CAAGAAACTGCCAGCGCTCTGCTCAGTGGCGTAATCAATACATCACAGGCTATAAATGGAAATCAAGAAGATGAAAAAGAATCTGAAGAGAAGAAAGATTTGCCT---------------GAAGAACCTGTAGAAACAAGTTTAGAAATGATGAGTAAATTATGTAAATATATTTATGCCAAGGATAACACTGACAGAATTAGAACCCAAGCTATGCTTTGCCATATTTACCATCATGCTTTACACGATAATTGGTTTGAAGCCAGAGATTTGATGTTGATGTCCCACTTGCAAGATTCAATTCAGCATGCGGATCCACCTACTCAGATCTTGTATAATAGGGCTTTAGTTCAGTTGGGACTTTGCGCATTTAGACACGGAAATATAAAAGATGCTCATAATGCCTTGTTGGATATTCAAAGCGGAGGAAGGGCCAAGGAACTCTTGGCGCAAGGTCTCCTACCCCAGAGACAACATGAAAGGACTGGAGAACAAGAGAAGATTGAGAAGAGACGACAGATGCCATTCCATAAGCATATTAACTTGGAGCTTCTTGAATGTGTATATCTTGTCTCTGCCATGTTATTAGAGATTCCTTACATGGCAGCTCATGAGCTTGAAGGCAGAAAACGAATGATCAGCAAATCATTCCATCATCAGCTTCGAAACAGTGAACGTCAAGCATTGGTAGGA [2010]

Proto TACGAAGAGCTGGTTGATCTTATTAAACAGATCAGGAACCATAAGAAAATCAAGGATATGAGTAAACTTCTAACGAGTTTTGAGAATCTGACTAGAGCTTTAAACAAAGCTAAGCTGGTAATAGAGAAGGAAGACGGTGGCATCACCCCAAAATTTTACATCCGTTGCTTGGTGGAATTGGACGATTTCATCAACGAATGTTGGGAAGACCGAGATGGCCGTAAAAATATGTCGAAGAATAATTCAAAGTCTCTAGCTACATTACGTCAAAAGCTAAGAAAGTATTGCAAAGATTTTGAAACTGCCATCAACGACTACCGTGAAAATCCGGAGGCTGAGGAAGATGAGAAAGAAGAAGAAGAAGCC------GTATCCGATGATGATAGAGACAGCGATTACTCGAAAGCTCCGGTAGCTGTTAAA------GAAGAAAGGGAAGAGGTGAAATCTAAATTCTTGAAA---GGGTCA---GATGATGAAGAATCTGACGACGATTCCATCGATTGGGGTTCGTCAGATTCGGAATCAGAAAGTGAAATATCCAGCGACGATGAAAAGTACGGGGATAATTTGGCTGTCAAGTTCTTGAAGAAACAGCCTGGTGACAAAGAAGAAAAGGAAGTGAAGAAACGG------GAAAAGAAAAGA---------------GATAAAAAGGGGAAAAAAAGAGACGGAGATTGGGTTGATGTTAAAGGCGGAATTCCGGTCATTGTCGAGAAGCCCAAAATGTTCGCCAAAGACGCGGAAATCAATCATCAGCTTGTACAAAAGAAACTGGTGGAAATTCTAGCGGCCAGAGGAAAAAAGGGAACAGATAGAAACGAACAAATCGAACTTCTCGGAGAATTACTTAGCATTTCTAACGCTCACGAGTTAGGAGCTGGAATGGAAGTCAAGATCAGATTCGCTATCGTGTCAGCCATCTTTGACTATAATCCCAATATCGCCACTTGCATGAAAACCGAAGTTTGGCAAAAATGCCTGGAAAATATTAACATCCTTCTGGATTTGCTGATCGCTAATTCCACT---CTCATAGTTGGAGAACACGTTTCCGAAGATTCCGAATCCCTGGCAACCCCGCCGTACAGAATTCGGGGATGCATCTTGACCGTTGTCGAACGTTTGGATGAAGAGTTCACCAAAATGTTG---CAAGGTTGCGATGCTCACTCTCCAGAATACGTTGAAAGGTTGAAGGACGAAAAAACGGCCTGCAACATCATTGAAAGATTGCAAGAGTACTTG---GAAGGGAAAGGAACATCTTCGGAACTATGTCGAATCTATTTAAGACGCATTGAACACTTGTATTACAAATTTGATCCAAACGTATTGAAAAGAAGGGCTAAC---CAGGAAACTCCCAGCGTCGCTGTCAACGGAGTGGACGAAACGCCTCAACCCGAAACAGAAGAAAAAACCGACGAAATAGAATCTGAAGAGAAGAAAGACTTGCCC---------------GAAGAACCCGTAGAAACAAGTTTAGAAATGATGAGTAAATTGTGTAAATATATCTACGCCAAGGATAACACTGACAGAATCCGAACCCAAGCTATGCTCTGCCATATTTACCATCACGCATTGCACGATAATTGGTTCGAAGCCAGGGATTTAATGTTGATGTCCCACTTGCAAGATTCAATTCAGCACGCCGACCCTCCCACTCAGATTCTGTACAACAGGGCTTTGGTTCAATTGGGCCTTTGCGCTTTCCGGCATGGAAATATCAAAGACGCTCACAACGCCTTGTTGGATATTCAAAGCGGAGGAAGGGCCAAGGAATTGTTAGCGCAAGGTCTTCTGCCCCAAAGGCAACACGAGAGGACCGGAGAACAAGAGAAGATCGAGAAGAGACGGCAAATGCCATTCCACAAACACATCAACTTGGAACTTCTCGAATGCGTTTATCTGGTCTCTGCCATGCTTTTAGAAATTCCATACATGGCTGCTCACGAACTCGAAGGCCGAAAACGTATGATCAGTAAATCTTTCCATCACCAGCTTCGTAACAGCGAACGCCAAGCGTTAGTTGGA [2010]

[TICKFAST_NCHAR=858;ferric-chelate reductase]

Ixodes ATGCCCTCCGGGAGCCGCTACTCCGGCTGCGGAAGCTCTTTCGGCTGCTACGGCATTCCTCAGGGCTGCCAGAAGAAGGGCTCTTGCTCCCTGCTGCTCACGCACAAGGCCGACAGGGACGGAGTCCTCTACGAGCTCGCTGCTCCTTCCACGGGCG---CCGACATGTGGATCGCAGCCGGGCTGTCCGACTCCAAGGCTATGGAACATGCTCACGTCGTAGAGTGCCTCCTGAAGGGTGGT---CAGACCAGCATCCGGGAGTCCTGGAACGGA---------GACGGTTACAAGAACTCTCTCGTAAAAAGGACTTCG---GGCGTGGACTACAGAAAAAGGGAACATGCGGACGGAATGTTAACGTGCTCCTGGCATCGCAGTCACCTTACGAATATTGGGACACAGTCATTTAACACCCTGAAAAACAAGTACCACCTCTTACTAGCCTCTGGTCCGCTTAAAGCTGGCACGGATGAAAAGAGCCAACATAAACACCGAGGTGGCACAGACAGTCCCGTCAACCTGCAGCAGAGCGAAGTGTCTGTGAGCGCGTCTGACTCCGATCTTTTCATCAAGCTTCATGGTTCATTGATGATAACTGCATGGATCCTGTTTGTCAGCGTTGGAATTCTGCTTGCCCGCCACTACAAAAATGTCTGGGAATCTACAATGATCTGTGGCGTTAAGCCCTGGTTCGCCTTTCACAGAGGCCTGATGATGCTCTCTGTTGCTATGATGATTGCTGCGCTCGTACTCATTTTCTACAGACTCGGGGGATGGAGTCCGAAT---GAAAACCCACACTCTATTCTCGGAATAGTCGCCTCTGTGTTAGGCTTGACACAGGTAAGC [837]

Siro TCGACAATGCTAGACGATTACGGCGTCTGCGGCATCACCAAAAGCTGCTTCGGTTTGCCGCAGAACTGCATCGAGAGAAAAGTCTGCGAAGTTCTGTTGACTTACATCAAGTCGAACGCGTCCCTCGATTTTAGCATGCTGGCCGCTTTTGGAGATTCCAATCCTCGCTGGGTCGGGGTGGGAATTTCCGAGGACGACTCGATGGGCGGCGACAGCGTAACGGAGTGCTTGGTACAAAATGAC---CGCGTCACGGTTCAACAGAGTTGGAACACGGAGCCC---AAAAAAGGCAACAAAGTGTTGACCAATCCAACTCTG---GGCATCAACGACGCTTCTGGAAGCATTGTGGATGGAGTCATGACCTGCAGATGGAAGCGTCCACTAGTGACCAATATTCAGGGTGTTACTTTCAACTTGGTCGATAACCGGTATTATCTGATGTTGGCCAAAGGAATCGTTCGTAGC---------AATTCG---AAAGTTCAACACACGAGCAGGTTGGCTTCGGAGCATTTGGTCGACTTCAATAAAATCATGTTGATTTCTGGAAAGCCAAATTACCTGTTGAAAATTCACGGATCTTTTATGGTTGTTGCTTGGATTGGCCTCGTAAGCATTTCCATCTTGCTGGCTCGTCATTACAAAACGGTTTGGGAAGACAGGACGTTATGCAAGCTCAAAGTTTGGTTTGCCTGTCATCGAACATTGATGGTACTTGCCGTGTCCATGATGATTTCTGCTTTTGTAATCATCTTCATTTACGTCGATGGTTGGAGTCAGGTTGCACCAAATCCTCACCCCATTTTGGGATGCGTTTGTACTGGTTTAGCAGTACTTCAGCCCATT [837]

Sitalc CTATTATCCCAAGACAACTACGACGTATGCGGTATCAAAAAGGGATGTTTCGGTCTTCCATCGGACTGCATCAGCAAGAAATCCTGCGAAGTTCTGCTCATGTACCTGCCCCAACCTGACGGCATTGAGTTCGAAATTACGGGGGCTGCCAGGAACGGGACAGAGACCTGGGTCGGGATGGGACTCTCAATGGACAAGTTCATGGGTGAGGACAGTGTGACTGCCTGCTTGAACCCATCAGGT---GGCGTAATAGTTCAGCAGAGTTGGAACGTACCTGAT---CGGAAGAGGAACGTTGCCATTGACAATCCTCACCAA---GGTCTCAGCGACATCAGAGGTTTATTGTCAGACGGCACCATGACCTGCAAGTGGAAGCGACAACTGACCACCAACATCGAAGGGATCACCTTCAATCTGACCAGTGACACATTCTACCTCCTGCTTGCCTATGGACCCACATCGTCA------GACGGGGAG---AAGAGGAAGCACACCCGTTATCTTGTTTCGAACAGTGACGTCAACTTCACCAAGATAGAGCTGGTGGCTGGAAAACCAGATTATCTTATCAAGATACACGGTTCCTTTATGATCATTTCTTGGGTGGGTTTCATCAGCGTGGCTATTCTTATTGCAAGGCACTACAAGAACGTCTGGGAGAGTGACACCCTGTGCACACTCAAAGTCTGGTTCTTTTGCCATCGAGCTTTGATGACGACAGCAGTCTCTTTAATTATAGCTGCTTTTGTCTGTATTTTCATTTACGTCGACGGTTGGAGTCAGGTT---CCAAATCCTCACCCAATTCTAGGCTGCGTGTGCACAGGTCTCGCTGTTATACAGCCCATA [837]

Sclero CCTTCAACGGACGTTTCCTACAAATCGTGCGGGGTCACGAACAGCTGTTTCGGACTACCCTCCAACTGTGTCCCGAAAGAGAACTGCCAAATTCTTTTGACCTATGCTCTGGACAGAGATGGGGTCATCTTCACGATTACCGGCACTTCCCTTAGCAGTCAGAACGCCTGGATCGCCGTCGGACTCTCGCGAGACGCTAAGATGGGAGAGGATAGTGTCACCGATTGTTTAGTATCTGAAGCGGAATCGATAACAGTCCAGCAGAGTTGGAATCTTCCA------GGATACAGGAATGAAGTTCTGAAACAAGGAAAACAA---GGCTTGAGTGCAATCTATGGGAAATATTCCGATGGAGTTCTCACTTGTCGCTGGAAGCGACAAACCACAACTAACATCAAAGGCATTACCTTTAATTTGACTTCCGATGAATACCATTTATTGCTGGCCATGGGAAAAACAGTTGGG---------GGAGTC---AAAGTAAAGCATGAGAAAAACATTGTCTCCGAACAGTTGGTGAATCTCACCAAAATCGAAGTAGTAGCTGGCAAATCCACCTACCTCATCAAAATTCACGGCTCTTTCATGGTGATCGCTTGGATTGGTTTCGTTAGTGTGGCCATACTCTTGGCCAGGCACTACAAGAACGTTTGGGAGAACGATACCCTTTGCACTCTCAAAGTCTGGTTCGCTTGTCACAGAACTCTAATGATGTCTGCAGTGTCCCTCATCATAGCTGCTTTTGTTTGCATTTTCGTCTATGTGGATGGTTGGAGTGAGGCT---CCGGGTCCCCATCCAATCCTGGGCTGCGTCTGTACCGGCTTGGCGGTTATACAGCCCGTC [834]

Trogulus CCGAACCAACAAGAAATCTACGATGTTTGCGGAAAAACTAAAGGTTGTTTTGGGATGCCAACAACATGCGTTGGGCGACGCAACTGCGATCTGCTTCTAACGTACATTCCGCAAAAGGATGGAGTTGCTTTTGAATTGATCGCCACTTTGGATAATGGATCTGACTATTGGATTTCAGTCGGAATCTCCGAAGATAAAAACATGGGTGACGACACTGTGACTGAATGCTGGACCAGTGATGGCAAAACTGCGGTTATTCGTCAAAGTTGGAACGAAGGGAACTTTAAGAAAAGGAACAAAGTTCTGGTCAATTCTCACCTG---GGAATCAGCGGAGACCTGGTTCAATACAACGGCGGAGTTTTAAGCTGTCGATGGAACAGGCAATTCTTGACTAACGTCGAAGGAGTCACCTTTAACCTGAAGGAGAACAACTACCATCTTCTTTTGGCTAAAGGACTAATCAGAAGAGGACAAAATGCTGAG---AAAATAAAGCACAACCATTACCTGCAATCCCAAGACAAAGTAAACTTGACCAGGATCTCCGTTGTTGCGGGAAAACCAAATTATTTGGTCAAGATTCATGGTGCTTTTATGGTCGTCGCTTGGATTGGAATTGTCAGTGTTTCCATTCTTCTCGCTAGACACTACAAAATTGTTTGGGAAAATAAAACAATCTGTGGAAGCAAAGTTTGGTTTTTCTGTCATCGAAGTATGATGTTGTTGGCTGTGTCCATGATAATAGCAGCATTTGTGGTCATTTTTGTGCATGTGGATGGCTGGAGCCAGGTTGCACCTAATCCACATCCTATTCTGGGATGCATCAGCACTGGATTAGCCGTCATCCAGCCCATT [852]

Ortho TCTAAGGAACAGGGTGCTTATGAAGTCTGCGGCAAGAGCAAAGGATGCTTTGGAATGCCCACCATGTGTGTTGAACGAGCCAACTGCGACCTACTCCTCATGTACACCCCACAGAAAGACGGAGTTGCGTTTGAGCTGACTGCTGCCTTGGACAATGGCTCTGATTACTGGATCTCTGTGGGAATATCAGAGGATAAGAATATGGGTGATGACACAGTAACAGAATGCTGGACGAGCGATGGAAAAACAGCCATTATTCGTCAAAGCTGGAATGAAGGAAATTTCAAGAAGAGGAACAAAGTCTTAGTAAACAGTCATTTG---GGTATTAGCGGAGACACCGTCAAGTACAACGGTGGTATCCTAACCTGTCGATGGAACAGGCAGTTTTTGACCAATGTTGAAGGAGTTACTTTCAACCTTAAGGAAAATGTATACCATCTTCTTTTGGCTAAAGGACTGATTCGTAGAGGACCATCTGCTGAA---AAGATAAAGCACAACCACTATTTGCAGTCTCAAAACAAAGTTAACTTGACTAAAATTGAAGTAGTGGCTGGAAAGCCAAATTACCTTGTCAAAATTCATGGTGCATTTATGGTTGTGGCATGGATTGGAATCGTCAGTGTCTCAATCTTACTAGCCAGGCATTACAAAATTGTTTGGGAAGATCGCAAAATCTGTGGAAGCAAAGTCTGGTTTTTTTGTCATCGAAGTTTCATGCTTCTTGCCGTCGCCATGATTATAGCTGCTTTTGTTGTTATATTCTTACATGTGGATGGCTGGAGTCAGGTGGCTCCTAACCCGCATCCCATTCTAGGATGCATTAGCACAGGACTAGCCGTAATTCAGCCAATC [852]

Hespero GACGCCAAACTGGGCTCGTACGACAGTTGCGGCAAGACCAAAGGGTGCTTCGGGATGCCCACCGAGTGTGTCCAGCGGGAGAATTGCGCCGTGTTGCTCATGTACACGCCGCAGACCGATGGCGTCGACTTTGAAATGACCGCCGCGCTGGACAATGGCTCAGACTATTGGATATCGGTCGGCATATCGGAGGATAAGTCGATGGGAGGCGATAGCGTGACCGAATGCTTGACCAGCGATGGAAATTCGGCCATTGTCCGGCAGAGTTGGAACGAAGTGCAC---AGGAAAATCAACAAAGTGCTCGGGAATTCTGGCTCTGGAGGAATATCGAACGCTTCCGTCTCTTATTCGCAAGGCATTCTGACCTGTCGTTGGAAGCGTAAACTGAACACGAACGTCGAAGGGGTGACCTTTAACCTCGCCGAAAACAATTATTATTTGCTGTTGGCCCGCGGGCCACTGTCCAGA---------GTGGAG---AAACTCAAGCATCGATTTCATTTGCAATCTCAGAACAAAGTCAACTTGACCACCATCGAAGTAGTGGCGGGCAAACCGAATTATTTGGTCAAGATTCACGGTGCTTTCATGGTTGTGGCTTGGATCGGACTGGTCAGCGTGTCCATTCTATTAGCCAGGCATTACAAATCAGTGTGGGAGGATAAAACTCTATGCGGGGCGAAAGTCTGGTTCTTCTGTCATCGAACCATGATGGTTTTGGGCGTAGGTATGATCGTGGCCGCTTTCGTGGTCATTTTCCTGCACGTGGACGGCTGGAGTCAAGTGGGCCCCAACCCTCACCCAATATTGGGTTGCGTTTGCACCGGATTGGCCGTAATTCAGCCAATC [843]

Leiobunum CCGGAAGAATACGGCACTTACGAAGTTTGCGGCAAGACGAAAGGGTGTTTCGGCATGCCAACCTTATGCGCCGAGAGAAGAAATTGCGAAGTATTACTCATGTACACCCCGCTACGTGACGGGGTTGCGTTCGAATTAACCGCCGCTTTGGATAATATCTCCGATTGGTGGATCGCGGTTGGTCTCTCGGAGGACAAGAAAATGGGAGGCGATAGCGTTACCGAATGCTTGACACACGATGGTTCGCAAGCCGCCATTAGGCAAAGCTGGAATGAAATTAAT---GCGAAACGTAACAAAGTTTTGGGAAATCCGTTATTG---GGGATGAACATGGCTTCGGTCGAATACTCCGGTGGAATTTTAACTTGCAGATGGAAGCGGCAATTGTTGACTAATATTGAAGGAGTTACCTTCAATATTGCCGAAAACAACTATTACCTTATGTTAGCCAAGGGATTAGTCAAAAGGAGTCCAGCCGGGGAT---AAAATTAAACATAAACTGTATCTTCAATCTCAAAATCAAGTAAATTTAACAACTATCGAAGTGGTTGCGGGAAAACCAAATTACCTTGTTAAAATTCACGGTGCGTTCATGGTAGTCGCTTGGGTAGGTTTAGTCAGCATTGGTATACTGTTAGCCAGACATTATAAGGCAGTCTGGGAAGATAGCAAAATATGCAAGGACAAAGTTTGGTTTTTCTGTCACAGAAGTTTAATGATGTTGGCGGTAGGCTTAATTATAGCGGCCTTCGTAGTGATATTTTTGCATGTAGATGGTTGGAGCCAGGTTGCCCCAAATCCACATCCGATAATAGGTTGTGTTTGCACTGGCTTAGGAGTATTACAACCTATC [849]

Proto CCCGAAGAGTACGGCACTTACGAAGTTTGCGGAAAAACCAAAGGGTGCTTCGGCATGCCTAACCAATGCGCCGCCAAGAAAAACTGCGAAGTCTTGCTCATGTACACCCCGCTGCAAGATGGGGTCGCGTTCGAATTAACCGCCGCGTTAGATAGCGCCTCCGATTCGTGGATCGCCGTTGGACTGTCAGAGGACAAAAAAATGGGCGGAGACAGCGTAACCGAATGCCTGACTAGTGACGGTACGCAACCCGCGATTAGACAAAGTTGGAATGAAATTAAC---GCGAAACGCAACAAAGTGTTGGGAAATCCGTTGCTT---GGAATGAGCATGGCATCTGTCGAATATTCGGGCGGAATTTTAACATGCAGATGGAAAAGGCAGTTGTTGACTAATATCGAAGGAGTTACCTTTAACATTGCCGAGAACAACTATTACCTTATGCTAGCCAAAGGCGTAGTCAAAAGAAACGCCGCCGGGGAA---AAAGCCAAGCATAAACTATTCCTTCCATCCCAAGACCAAGTGAATTTAACAACTATCGAAGTGGTTGCTGGAAAACCAAACTACCTTGTCAAAATTCACGGTGCTTTTATGGTTGTCGCTTGGGTTGGATTAGTTAGCATTGGCGTACTGTTAGCCAGACATTATAAGGCAGTCTGGGAGGATAGCAAAATATGCAAGGACAAAGTGTGGTTTTTCTGTCACAGAAGTTTAATGTTATTAGCAGTTGGATTAGTCATAGCGGCCTTCGTAGTTATATTTCTACATGTGGATGGTTGGAGCCAGGTTGCTCCAAATCCGCATCCAATAATAGGATGCGTTTGCACTGGTTTAGGCGTCTTACAACCCATT [849]

[TICKFAST_NCHAR=636;gamma-soluble NSF attachment protein]

Ixodes ATGCTAAGCCTGCCTCTTGAGGTGCAGCACGTTTTCTTCTTCTCAAGTGGCAAGCAGCTGGAGCAGGCTGCACTGGTGAGCAGAGACATGGGGGACCTGAACGCAGCCGGAGAGCTGGTGGACCGGGCCTCCCGCCTCTTCGTAGACTCCCGCAGCCCAGACACTGCCGCCCTCACGCTGGAGCGGGGAGCCAAGATCATCGAAAACAAGCATCCTCAGGCAGCAGTAGAGTTCTTCAACAGAGCAGCAGAAATCGTTTCGGTGGAGGACCGGCCCCGGCAGGCGGCGGAATTCTGCGGCCAGGCGGTCCGGCTCTTGCTCAAGACATCCAGGTGGGAGGAGGCAGCGGAGGCCATTGGGCGTCAGCGGGAGTTCCTGCGGGAGGCCGGGGACGAGCGTGCGGTGGGGCGCCTGGTGGTGGCCTCCGTGCTCGTCCACCTGGCCCGGGACGACTTTGTGGCCGCGAGCAAGGCCCTCGACCAGGGCGCAGGGTTCCTGGAGGGGGAGGAGCAGGACACCCTGTCCACGCTGCTGGAGGGCTGCGACCAGGGGGACCCCGACACCGTGGCACGCGCCCTCAAGAGCCCCTTCCTCAGGCACATGGACACCGAGTATGCCAAGCTGGCCAGGATGCTG [636]

Siro ATGAAAGCAGCCGACTGCTACGGAAAGAATCAATCATACTTTTCTGCCGCAAAGAGTTACGAACAGGCTGCTTTGATTAGCAAAGAGCTGAATGATTATGAAGCTTTGGTCCAATTGGTTTTAAGGGCATGTCAACTGTTTCGAGAGCACGGCACCCCCGACACAGCGGCCATGACCCTTGAAAAAGGGGCCAAGATGATCGAAAGCAAGTTTCCCGAACAGGCCATTGATTTTTATCGACAAGCAGCAGAAATTGTTTCCAACGAAGATCGACCAAAACAAGCAGCCGAGTATACGGGAAAAGCCGCCAGGCTATTGGTCAAGCTGAAAAGGTACGACGAAGCCGTTGTCTGTCTACGTAAAGAGATGGACTGTCACGTAGAATCGGAGGACGAAGGGTCGGCGGGTCGCCTGGTGGTGGCGCTAGTTCTTCTACATCTGACGCAAGAGGATTACGTGGCAGCAGATCGAGCCTATAAGGAAGGAGCCTGTTACGTCTCTCGTGACGAAGCCGGAGCTCTAATTGACCTCTTGGAAGGTTTTGACCAGGGAGATTACGAACAGATGTACAACGCTCTTAATCACCCTTTCATCAAACACATGGACATTGAATATGCAAAGCTGGCAAGAAACATT [636]

Sitalc ATGAAAGCCGCAGACTGTTTTTCTAAGAATCATTCATACTTTTCAGCTGGCAAAAGCTACGAACAGGCAGCCATGGTGTCTAAGGAGTTAGAAGACTACGAGTCAGTGGTCCAGTTGGTGGGCAGGGCTTGCAAGCTGTTCATCGAACACGGTGCACCGGACACTGCGGCCCTCACTTTGGAAAAGGGAGCAAAGATCGTAGAACCAAAGTTTCCAGAAAAAGCTATCGAGCTTTATAGCCAAGCTGCAGAAATAGTTTCAATTGAAGACAGGCCAAAACAAGCAGCTGAATATTTATGCAAAGTTTCACGTTTATATGTTAAACTAGAAAGGCACAACGAAGCCATGGGTATGTTGCGCAAGGAGCTGGCCAGTCAGGTGGAAGCGGAGGACTGGGCGTCCGCCGGAAGGATCGTCATTGCCCTGGTAATCCTGAATCTCTACCTGGAGGACTACGTGGCCGCCGAGAAGGTCTTCAGAGACATGGGGAGTTACGTGTCATCGGATGAGAAAATGGCGCTGAGCGACCTCTTGGAAGGTTACGATCAGGGGGACCCTGAACTCTGCAGCAGGGCTCTTTCGCACCCTTTTTTCAAGCACATGGACGTAGAATATGCCCGTCTTTGTCGGAATCTG [636]

Sclero CTGAAGGCTGCAGACTGTTATACAAAAAACCAATCATATTTTTCTGCAGGAAAGAGCTACGAGCAAGCGGCCATGGTCTGCAAAGACTTGGGCGACATGCCGGGTGTTTGTCAACTTGTGGAAAGGGCTTGCCAACTCTTCAGGGAAAATGGAACTCCCGACACAGCAGCACTCACATTACAGAAAGGGGCCAAAATCATTGAAGGCAAGTTTCCAGAAAAAGCTGTAGCACTTTATTGTCAAGCAGCAGAAATTGTTTTGCTTGAAGACAGACCGAAGCAAGCAGCAGAATATTCAAGTAAAGCTGCAAGGCTTTATGTCAAACTTGAAAGGTTTGAAGAAGCCCTGGTAATGCTTCGTAAGGAATTGACTAATCAAGTGGATGCCGAAGACCCAGCCTCAGCCGGGAGGATTGTCATCTCTCTGGTCTTGTTGCACTTGACCATGGAAGACTATGTGGCAGCCGAAAAAGTCTTCAGAGAAATGGGATCTTACGTGTCTCGTGATGAAAGTATGGCGCTGATTGAACTTCTGGAAGGCTACGATCAAGGAGATCCAGAACAATGCTACAGAGCTCTCGCACATCCCTTCTTCAAGCACATGGATGTGGAATATGCCAGGCTGTGCCGAAACCTG [636]

Trogulus CTTAAAGCAGCTGATTGTTATGCAAAGAATCAATCATATTTTTCTGCAGCCAAGAGTCTTGAACAGGCTGCATTTGTGAGCAAAGATTTAGAAGATTTCAATGGGGTTGTAGAATTGGTGAGTAGAGCCTGTCAGCTTTTTCGTGAACATGGAACTCCTGACACTGCAGCAATGTGTTTGGAAAAAGGAGCCAAAATGATTGAAAGCAAATATCCAGATCAAGCAGTAGACTTGTATATCAAAGCAACTGAAATTGTATCACTAGAAGAAAGGTCTAAACAAGCTGCAGATTATGCCAGCAGGGCTTCACGTCTCTTAGTAAAACTTAAAAGATACGACGAAGCCATTACAATGTTGAAAAAAGAGATGGAGAATCATTTAGAAGCCGAAGATGGTCCATCCGCTGGACGTGTAGTCATTGCACTTTGCCTTTTACATCTTTCTTTAGAAGACTTTGTCGCCGCTGATAAAGTCTACAAAGAATTTGGAAGTTATATCCCAAGAGATGAAAGAGCAGCAATGGGTGATGTTCTCGAAGGTTTTGATCAAGGAGATCCAGAAATGGTTTACAATGGTCTTAACAATCCATTCTTCAAACACATGGATGTTCAGTATGCCAAATTAGCTCGAGATCTT [636]

Ortho CTAAAGGCTGCTGACTGTTATGCAAAGAATCAATCGTATTTTTCAGCAGCCAAAAGCTTAGAGCAAGCTGCATTTGTAAGCAAAGATTTGCAAGACTATAATGGTGTTATAGAACTAGTCAGCAGGGCATGTCAGCTTTTTCGTGAACATGGTACTCCTGACACTGCAGCAATGTGCTTGGAAAAAGGAGCTAAAATGATTGAAAGCAAATATCCAGAGCAAGCTGTCGAACTGTACATGAAAGCTGCTGAAATTGTATCGCTTGAAGAAAGGTCTAAACAAGCTGCAGACTATGCCAGTAGAGCATCCCGTCTTCTAGTTAAACTCAGACGCTTTGACGAATCAATTTCAATGTTGAAAAAAGAGCTTCAGAACCACTTGGACGCCGAAGATGGACCATCTGCTGGGCGTTGTGTAATTGCTCTGTGTCTTTTACATTTAACATTGGAGGATTTTGTAGCAGCAGATAAAGCATATAAGGAATTTGGAAGCTATATTCCAAAAGACGAGCGGGCTGCAATGGCTGATGTTCTTGAGGGCTATGATCAGGGAGATCCGGAATTAGTATTCAATGGTCTAAACAACCCATTTTTCAAACACATGGATGTACAGTATGCCAAATTAGCACGTGAACTG [636]

Hespero TTGAAAGCTGCTGATTGCTACGGGAGAAATCAATCATATTTTTCGGCTGCAAAGAGTTTGGAACAAGCCGCTTTAATCAGTAAAGAACTGGAGGATTTTCCTAAAGTTATTGAGCTGGTCAATAGAGCTTGTCAATTATTTCGAGAACACGGAACACCTGATACTGCAGCAATGTGTTTAGAAAAGGGGGCAAAGATAGTCGAGGCTAAATTTCCAGAACAAGCTGTAGAGCTTTACGTAAAAGCTTCGGAAATAGTGGCGCTTGAAGATAGAAGCAAACAAGCAGCTGATTACGCCAGTAGAGCTTCGCGTCTCTTAGTCAAATTGAAACGGTACGATGAAGCTATCGCGATGCTACGCAAAGAATTAACCAATCACATTGAAGCGGAGGATGAACCTTCTGCTGGAAGAGTCGTTATTGTGCTTTGCATATTGCATTTGACTTTGGAGGATTATGTTGCTGCAGATAAAATATTTAGAGAATATGGAGGTTTTGTTCCGAAGGATGAAAGCATGTCTTTAACCGATCTCTTGGAAGGATACGATCAAGGTGATCCTGCTCAGGTTTACGCAGCCCTATCCAACCCATTCTTTAAACATATGGATGTCCAGTATGCAAAATTGGCTCGAGAATTA [636]

Leiobunum CTCAGGGCTGCAGATTCTTTTGGAAAAAATCAATCATATTTCTCTGCTGGCAAAAGTTTGGAGCAAGCCGCTTTTATAAGTAAAGATTTGGAGGAGTATGATAAAGTTATCGAATATGTTTCTCGAGCATGTCAACTTTTTAGAGAACATGGTTCTCCTGACACTGCTGCTATGGTGCTCGAAAAGGGAGCCAAAATGATTGAGCAAAAATTACCAGATAGAGCTATTGAGTTATACTGTAAAGCCGCAGAAATTGTTACGCTTGAAGAAAGGCCAAAACAAGCAGCCGATTATGCAGGAAAAGCCTCAAGATTATTGGTGAAATTAAGGAGGTATGATGATGGTGTTACAATGCTCCGTAAAGAATTACAGAGTCATTTAGAAGCGGAAGATGGCCAAGCAGCTGGTCGACTAGTCATAGCCTTATGTTTGCTCCACCTCACTCTAGAAGACTTTGTTGCGGCTGACAAAACCTTTAAAGAATACGGAAGTTACATTGGTAGGGATGAAAGAATGTCACTAGTTGATGTACTTGAGGGATATGATGAAGGTGACCCGGAAAAAATATCGAACGCTCTTAATAATCCATTCTTTAAACACATGGATATTGAATATGCCAAACTTGCCCGTAATCTC [636]

Proto CTCAGGGCTGCGGATTCTTTTGGTAAAAACCAAGCATATTTCTCTGCTGGAAAAAGTTTAGAACAAGCAGCGTTTATAAGCAAAGATTTAGAAGAATATGATAAAACTATTGAATATGTCACTCGGGCTTGCCAGCTCTTTAGGGAGCATGGTTCCCCTGACACCGCTGCTATGGTACTTGAAAAGGGAGCAAAAATGATTGAAAGTAAACTACCAGATAAAGCTATTGAATTATACTGTAGAGCTGCAGAAATTGTTACTCTTGAAGAAAGACCGAAACAAGCTGCAGATTATTCCGGGAAAGCTTCACGATTATTAGTTAAATTAAGAAGGTATGACGAAGGCATTACGATGCTCCGTAAAGAACTATTGAGTCATTTAGAAGCTGAAGATGGCCAAGCAGCTGGTCGCCTAGTCATAGCCTTATGTTTGCTCCACCTCACTCTAGAAGACTTTGTCGCAGCTGACAAAACTTTTAAAGAATATGGAAGTTACATTGGTAGAGATGAAAGAATGTCACTTGTAGACGTACTCGAAGGATATGACGAAGGGGACCCAGAGAAAATATCGAATGCTCTTAATAATCCATTCTTTAAACATATGGATATCGAATATGCCAAACTTGCACGCAATCTC [636]

[TICKFAST_NCHAR=312;general transcription factor 3C polypeptide; SCSE]

Ixodes GACGAAACCTACGTGCTGGTCGAACTGACGGGCGTCATTGACAGCGAGGTGATGAACTTGGCGGGCAAGACGTGTTGTATGCTCGGCGTGGACACGGCCGAGCCCGTGCTCAAGCTCGGCTCGTACCTGTTCACGGGTGAGTACAGCGACACCGTCGGGACGTGCGTGCTCTTCGAGGACATGGGACTGGCCAGCGAGCCGTCTACTTCCAAGTCGGTGTCGTCGTTCAGCAGGCACCCGCCCAAAGTTCTCAGGTACTTAGCCAAGACCGAGAAGCGACTGGACATGAAGACGTCCTTTCTGCAGGAAAAA [312]

Siro GACGAAGCGTTGGCGGTCGTCGAATTGAACGGAATTATTGACGAAGATGCTATGCGACCT------CAAATATGCAAAATTCTTGGAGCAGAAACGGGCAGGCCAGTGTTGCAAATGGACAGTCATTTTTTCACCGGAACTTACGAAGAAATCGTCGGCACCGCCGCCATTTTTGAAGAAGCTAAGCCTAACTCGGACAATAAA---------------------------------------CCCTCATTGAAATACGTTTGTACAACCACAAAGAAACTGATATTGCAAAGATCATTTCTTGAAAAGAAA [267]

Sitalc GAAGAGGTTCAAGTTTTGGTAGAACTGGCGGGAATTATCGACCCCGAGGTTCTCCAGCTTCAGAACAAACAGTGTAAGGTGCTGGGCCTCGACACCCAGAATCCCGTGCTTCAAATTGACAATTACTTCTTCACCGGGCCCTACGAAGAGATAATCGGTACGGCTGTGCTCTTCGAAGAAGCGAGCGACGG------------------------------------------------------CGCGCTGAAACACGCGTCCAACTGTTCCAAAAAACTGAACATGAAAAGAGCCTTTCTTGAAGAAAAG [258]

Sclero GAAGAAGTTGAAATTCTTGTTGAACTTTCAGGAATTATTGATTCAGAAGTGCTCTCTTTACAAGACAAGACGTGCAAAGTTTTGGGGCTTGACACGAACAATCCCGTTCTTCAAATTGATAATTACTTCTTTACAGGACCCCACGAAGAGATTGTGGGCAGTGCAATTTTACTGGAAGAAGCAGGCGAGGG------------------------------------------------------ACATCTTAAACTGGCTTCCATGACTACCAAGAAACTACCAATGAAGAGAGCGTTTTTAGAGAAGACA [258]

Trogulus GAAGAAATTTATGTCTTAGCAGAAATTTCTGGAGTAATAGACTCCGAGGTATTACAACTTCAAGGAAAATCATGTAAGATCCTTGGTCTGGAAAGTCAACGACCAGTTTTGCAAATTGATGAGCTAGTGTTTGCTGGAGAGCACGATGAAAGTCTCGGTACAACCGTTATATTCCAAGAACAAGAAAGTAGTACATCTGTTGCAGTTAAT---------------------------------AAAGAGTTGAAGTTTCTTTGCCATACGCCTAAGAAACTTAATTTTAAACGCGCATTTCTAAGAGAACGC [279]

Ortho ??????????????????GCAGAAATTTCTGGGGTTATTGACTCGGAAGTTTTACAACTTCAAGAAAAAAAGTGTAAGATACTCGGTCTGGAAAGTCAACAGCCAGTTTTACAGATCGATCAACTAGTATTCGCCGGAGAACACGATGAGAGTTTGGGGACAACTGTTGTGTTTGAAGAAACGTGTGAAAATTCCGACAAG------------------------------------------AAAGATCTGAAGCTACTTTGTCATACCTATAAACAGTTGAACTTTAAAAGAGCTTTCCTTAATGAAAAA [270]

Hespero GAGGAGGCTTTTGTATTAGCCGAAATAACTGGCGTAATCGACTCCGAAGTGTTATCGTTGGACAATAAATCTTGCAAGATATTAGGATTAGAAAGCGACGAGCCTATCTTGCAAATAGAAGGTTTCTTTTTCGCGGGCAAACACGACGAAAGCGTGGGCACGAGCGTGTTTTTCGACTCCAAACTTAAAGACGTCACT---------------------------------------------ACACACGCGGTGGCCCCGCAGGGTGCAGAAAAACAATTAGAATTGTTGTGCCATACGACCAAGAAAATT [267]

Leiobunum GAGGAAGTGTTAGTTTTAGCAGAAATTTCGGGAATCATAGACGAACAAATGTTAAATCTGAAAGACAAAAAGTGCAAAATTTTAGGTATGGAAACAAATGAACCCATCTTGCAAATTGGCAATGTTTTCTTTGCTGGTGATCATGATGAATGTGTGGGAACTGCTGTGATATTTGAAGAAAAAGCCTCCACAGAAGATGCCAAA---------------------------------------AAGAAATTGGAATTCTTTAATTTAACATCTAAGAAGATAAAGTTTAAAAGGGTATTTCTCAAAGAGAAA [273]

Proto GAGGAAGTGTTAGTTTTAGCTGAAATATCTGGAATCATAGACGAACAAATGCTAAATCTGAAAAACAAAAAGTGCAAGATTTTGGGTTTGGAAACCAATGAACCTATCCTTCAAATTGGTGACGTTTTCTTCGCTGGGGACCATGATGAATGTGTAGGAACTGCAGTGATATTTGAAGAAAAAAGCGCCGATGAAGATACCAAA---------------------------------------AAGAAATTGGAATATTTTGACATAACTTCGAAGAAGATAAAGTTCAAAAGGGTATTCCTCAAAGAAAAA [273]

[TICKFAST_NCHAR=990;glutaminyl cyclase; part40]

Ixodes TGGCCCCGAGATCTGCGGCCGCTGGCCCATCATGATCTGCTGTACATGGGACAGATTTCGGAGGAGGACAGGGGGGACTTCAACGCCACCCTGCGGAACTTTCTCGTCCCCAGGGTTGTGGGGAGTCAGAAGCACCGGGAGGTCAGGGAGTTCATTGTGAGGTCGCTCAAGGATCTCGACTGGGATGTGGAAGAGGACTGCTTCGACGGCCAGACTCCGCATGGGATAAAACCATTTTGCAACGTGATAGCGACGCTCAACCCAAGCGCGTGTCATCGGCTGGTGCTGGCCTGCCACTATGATTCCCTGCTCCACAAGGAAGGAACCTTCATTGGTGCTACCGATTCTGCTGTGCCGTGTGCTCAACTGCTGTACTTGGCGAGGTCCCTGAATGGCAAGCTGCAGAACCAGAAGACGAGAGGCGATGGTCTGACGCTTCAGCTGGTCTTTTTCGACGGGGAGGAGGCATTCGAGCGCTGGAGCTCGCACGATTCCCTCTACGGCTCGCGGCACCTCGCCCAAAAGTGGCACGAGGACCGAACGTCGGCCGAGAGG------------------CTTGAGAGCTGCCTCGAGAGGAGCGAGATCGCCAACCAGATCGACCGAATGGAAGTGATGGTGCTCCTGGATCTGCTCGGGGCAGAGAATCCACGTTTCTACAGTTACTTCGGGGAGACTCAGCCCGTTTACCGGAGGCTCGTCAACATCGAGAGCCGGCTCAACGACGCTGGGCTGATGGAACTTCCACGTCGCCGACGGAGGACCAACTACTTTTCCAACAGTTCAACGGTGGGCTTCATCGAGGACGACCACATCCCCTTTCTCAAGAGAAGCGTCCCCATCGTGCACATCATCCCGAGTCCCTTTCCAGACGTTTGGCACACGCTCGACGACAACGAACAGAACCTGCATCACCCGACCATCTCCAACCTGAACAAGATTTTCAAGGCATTCGTGTCGGAGTACCTGCAGCTA [972]

Siro TGGCCGCATGCATTGTTGGAATATAGTGACAATTCATTGTTAAGCATAGCACAGAATATTCCAGAGAGTTATGATGATTTTAACAAAACGCTGGACAAGATCCTCAAAGTTCGTGTTCCAGGCACCGATGGACATAAGCAAGTTCAAGACTACATCGTGGAATCCATGACGGATCTGGGATGGAAAGTCGAAGAAGACCATTTCACCGAACAGACTCCAGTTGGGAAGAAACCCTTCACGAACATTGCAGCATTTCTCAATCCGGACGCCTGCCGGTTTCTGGTTTTGGCCTGTCATTATGATTCCAAGTTGAGTTCCAGCAACAAGTTTTTAGGAGCTACCGATTCAGCCGTGCCTTGTACCATGATGATCCATTTGGCCAGAATGTTAAAAAACGAACTACAGCAGAATAAGGAGAAGTCGCAAAATCTAAGTCTTCAGTTCCTGTTCTTCGATGGTGAAGAAGCCTTCCGCTATTGGTCAAGAACAGATTCCCTCTATGGATCGCGCCACTTGGCCGAGAAATGGAACAAAACCCCTTATCTGCAGGGCAGG---------AAAGTTCCCAAGCAATGTGTTAACCTAAAGAAGGATGCTGTGTCTTTCCTTGATCAGATGGATGCCATGGTTCTTCTGGACCTTATTGGCACTGCTAATCCTCGCTTTTACAGTTACTTTGGTGATACCCACGCCCTCTACACCCAATTTGTTGGAATAGAAAACAAGCTGAACGATATGAAGCAAATGGAGTCGCATCCTCCTCGTACACGAACCCAGTATTTTAACCCTCAGCTCAGTTACGCCGGAGTTGAAGACGATCACGTTCCATTTATGAAACGAGGTGTCCCTATTGTCCACCTGATACCTAACCCTTTCCCTGACGTCTGGCACAAGGACAGTGACGATCGAGCACATCTGCACCACCCCACTATCTGCAACCTCAACAAGATTTTGACCATCTTTGTTTCCGAATATCTGCACCTC [981]

Sclero TGGCCACACCAAGTGTTGCAGTACAGTGATGCCAACATGGGAAATTTGGCCCAGACTGACAACGGACACATGCTGGACTTCAACGAAACTCTAGACGCTATCCTAAGGCCAAGAGTGCCGGGCTCAGAGGGAAGCAGGAAAGTCAGAGAGTACTTGAAGTCAAGTATGTCCGACCTTGGCTGGCACATAGAGGAAGACACCTTCGAGTCGCCAACCCCGATGGGTGTTAAGCCCTTCTCGAATGTGATGGCCACTTTGGATCCGGAAGCTTGCCGTCACCTGGTAATGGCCTGCCACTTTGACTCGAAGGTCTCTCGAGAGGACACGTTTCTAGGAGCCACCGACTCGGCCGTGCCCTGCGCCATGCTGGTCCATTTAGCTAAATCCCTGAAGGAACCCCTGCAGCAGAGTCCTCTGAAGGGA---AATGTCAGTATTCAGTTGCTTTTTTTGGACGGAGAGGAAGCGTTTCAGCAGTGGAATGAAAAGGACTCTCTCTACGGTTCCAGGCACCTGGCAGATCTTTGGAACAAGACCCCCATTCTAAAGAGCACT------------------GGGGGCTGTCGGCGGAACGGAGAAAATATGGTCTCTAGACTAGATCAAATGGATGCTTTCGTTTTGTTGGATTTAATCGGCGCTTCCAATCCTCGGTTCTACAGTTACTTCAGCAATACTTTCGGCCTTTACGCTAGGTTGAATGCTATTGAACAAAAACTGAATGAAATGCAGTTGCTGGAGACCGAAGCACCCCAAGCAAAGACTCGCTACTTCGCTAACAAATCTAGTTTTTCTTTCGTCGAAGATGATCACATTCCCTTCATGCAAAAAGGTGTCCCGATCATCCATTTGATTCCCACTCCGTTCCCTGCCGTCTGGCACAAAGACTCGGACAATCGTGAAAACCTTCATCACCCTACCATCAACAATTTGAATAAGATATTCAGAGTCTTTGTCTCGGAGTATTTGCATCTT [969]

Sitalc TGGTACCACCAGGTGAGCCAGTACAATGACAACGCCATGTTCAACCTGGCCCAGACTGACGAGGGCCACATGGTGGACTTCAACGATACCCTGGACGAGATCCTCAGACCACGGGTCCCGGGATCACCCGGAAGCAGGAGGGTCAGACAGTACATAGTGGATAGCTTGACGGACCTGGGTTGGAAGGTGGAGGAGGACTCGTTCGAGTCCGAGACCCCGCTGGGGGTGAAGCCCTTCACCAACGTCATGGCCACCCTGGACCCCACCGCCTGTCGTCATCTGGTCCTGGCCTGTCACTACGACTCGAAGCTGTCCCGGGAAGACACCTTTCTGGGGGCCATCGACTCGGCCGTCCCCTGCGCCATGCTCGTGCACCTGGCCAAGGCCCTCAAGGAGCCTCTCGAGCAAAGTCCACAGAAAGGC---AACGTGAGCTTGCAGCTGTTGTTCCTGGACGGAGAGGAGGCGTTCCAGCAGTGGTCCGAGAAGGACTCTCTGTACGGGGCCCGCCACCTGGCTGACCTCTGGAACAAGACCCCGCCTGCCATCACAAAAACCTTTGAGAACCAGAACCGGGTCTGTAAGAGGAACGGAGAAACCATGGTCTCCAGGCTGGACCAGATGGACGTGTTCGTTCTCCTGGACCTGATAGGATCGGCCAATCCACGCTTCTACAGTTACTTCAGCAATACCTTCAACCTGTACGGCAAGCTACATTCGATAGAGGAAAAGCTGAACGATCTGCGAATGTTGGAGACCCCGGCCCCGGCTTCAAAGACGAAATATTTCGCCAACAAATCCAGTTTCTCTTTCGTTGAAGACGACCACATACCCTTCATGCAGAGAGGAGTGCCAATCATCCACATGATCCCAGTTCCCTTCCCCGCCGTCTGGCACAAGGACTCTGACAACCGAGAGAACCTCCACCACCCGACCATCAACAATCTCAACAAAATCATCCGCGTATTCGTCTCCCAGTACCTGCACCTC [987]

Trogulus TGGCACCACAGCATGTATCGCTACGATGACACAGCCGTCCTTTCTTTAAGCCAAACGAGTTCCCATCACCACGACGACTTCAACAAGACCCTAGAAGCCATTCTGAAGCCCAGAGTTCCAGGAACCGAGGGTCACAAACAAGTCAGAGAGTATCTAGTGAAGTCTATGTCTGATCTGGGTTGGACAGTGGAAGAAGATTCCTTCAGTGCACAGACGCCCATGGGACGAAAGCCCTTCACCAATGTGGTGGCTTCTTTGCACCCCAGTGCCTGTCGGTACTTAGTTTTGGCCTGCCACTACGATTCTAAATTGATGAAAGGAAAGATATTCCTCGGAGCCACCGATTCAGCTGTTCCTTGCGCCATGCTCGTTCACCTTGCAAGGTCACTCGATGGAGCACTCAGGAAC---AAGAACCATTCC---ATGCTGAGCTTACAAACCATCTTCTTCGACGGCGAAGAGGCTTTCAAAGATTGGACCAAAAAAGATTCCCTTTATGGATCACGCCACCTAGCGGAGGTGTGGAACAGGTCTCCTCCCACAGCT------------------------AAAGACTGC------------CCAAAAGCGGTTTCCAGGATCGACCAAATTGATGTTTTAGTTTTGCTGGATCTACTCGGAGCGGCCAATCCAAAGTTTTATAATTTCTTCACCGAAACCAGTGAAGTTTATTCGAGGTTTCCAGCTATTGAACGATTATTGAACAATTTGGAGGAAATGGACGGTCCACCTCCTCAAGGGGCAACCAGTTATTTTGGCAGTCAAACTTCCTGGTCACGGATCGAAGACGATCATGTGCCATTCATGGAAAAAGGTGTTCCAATTGTACATGTCATTCCATCGCCCTTTCCATCAGTCTGGCATCGGGAATCTGATAATGGATCCAATCTGCATCAGCCTACGATTGACAACCTAAATAAAATTCTTAGGATCTTCGTTTCGGAGTATCTACATTTA [948]

Ortho TGGCATCACAGTCTATATCGGTACGATGATAACGCCGTCCGATATCTAAGTGATGCTGGGCCTGATCACTTTGATGACTTCAATAAGACGCTGGACGCCATTTTGAAGCCTAGAGTTCCTGGGACTGAAGGTCATAGACAAGTTAAAGAGTATTTAGTTCAGTCGATGGTTGATTTGGATTGGGCAGTCGAATTAGACTCGTTCAGCGCTCAGACCCCAGTTGGACGGAAAGCCTTCAGCAACGTGATAGCCTCGCTTCACCCTAGCGCATGTCGATATCTTGTGCTTGCCTGTCATTATGATTCCAAGATGATGAAGGGG---ACGTTCCTAGGTGCCACGGACTCAGCTGTTCCTTGCGCCATGCTCGTACACTTGGCCAGGGCGCTTGATGGTGCACTGAGGAAC---AAGAACCATACC---ACGTTGAGCCTTCAGCTGATATTTTTCGATGGTGAAGAAGCTTTCAAAAGCTGGACGTCGAAAGACTCTCTCTATGGGTCGCGTCATTTGGCAGAAACATGGAACCGATCTCCACCAACCAGA------------------------GAAGGATGCACCAGGAAAACCGAAAGGATGGTTTCCAGATTGGACCAAATGGATGCCCTGGTCCTGTTGGATTTATTGGGTTCAGCCAATCCAAAGTTCTACAATTACTTTTCCGCTACTGAAGAAGTATATAGCAGGCTTCCAGCTATAGAGAAGCTGCTAAACAACCTAGACGCCATGGATGGTCCACCGCCTCAAGGTACAACCAGTTACTTCGCCAGTAAAACATCCTGGTCTTTGGTCGACGATGACCATGTGCCCTTCATGGAGAGGGGTGTTCCTATTATTCACGTCATTCCATCACCCTTTCCAGCTGTATGGCACAAGGAATCAGACAACGGTTCCAATCTCCACCACCCAACGATCTCCAATATGAACAAAATCTTCAGAGTCTTCGTCTCGGAATACCTGCACCTG [957]

Hespero TGGCATCACAACCTGTACCGTTTCGATGACCTTTCCATCAAATCGCTGGCCGAAAGTCCTAACAAGTATTTGGACGACTTCAACAAAACTTTGGACGCCATTTTGATACCACGTGTTTCTGGATCGGAAGGAAACAAACGCGTCAGAGAGTATTTGATAAAATCTCTGGAGGATTATGGATGGACAATCGAACAAGACTCGTTCACGTCTCGGACCCCAGTGGGTCGCAAACCGTTCAGCAACGTGATCGCCACCCTCAACCCTTCCGCTTGCCGCCATCTTGTATTGTCCTGCCACTACGATTCCAAACTGAGCCGAGAGGACACGTTCTTGGGGGCCACAGACTCGGCTGTGCCATGCGCCATGTTGGTTCACTTGGCTCGGGTGCTGGACGGGCCGTTGAAGGAGCAGAAGCAGGCCACGGACACGCTGAGTCTTCAGCTAATGTTCTTCGACGGGGAAGAGGCTTTCAAGACATGGTCAAGCAGCGACTCGTTGTACGGGTCAAGGCATCTGGCTGAAACGTGGAATAGGGAAGCTCCCAAGATTAAAGAG---------------GGGCAAGAATGTCGCAAAAACGGGGAGACTATGGTGTCCAGGCTCGACCAAATTGACGTGATGGTTTTATTGGACCTGTTGGGCGCCGCCAACCCGAAATTCTACAATTTCTTCCCGGAAACGAAGACGATTTACTCCCGATTGCCTGCGATCGAGCAACTCCTGAACGGGCTGGACATGCTGAGCTCGCATCCCCCCCTGTCGGCGACCCGATACTTCGACACTAAAACGTCGTATTCGGGGGTCGAGGACGACCACATTCCGTTCATGGAGCGAGGGGTGCCCATCATCCACGTGATCCCCACCCCCTTCCCGCACGTGTGGCACCAGGAGACCGACAACAGGCACCATTTGCACCACCCGACCATTGACAACCTAAACAAAATTATGAGGATATTCGTCTCCGAATATCTGCATTTA [975]

Leiobunum TGGGCACACGAGTTAAGGCGGTTTGATCAAAATTCTTTGCTTGCATTGGCTGAGACAAATAACCTTCATCATGATGATTTCAATAGAACCTTAGACAATTTATTAAAAATTCGTGTACCAGGAACTGAAGGTCACAGACAAGTCAGAGAATATATATTCAATTCTATGAAAGATCTTGGCTGGTATGTTGAGGAAGATACGTTTACATCTAGAACACCGCATGGAAGAAAACCTTTCAGTAATGTTATTGCTACTCTCAACCCAAATACCTGTCGACAATTGGTCTTGTCATGTCATTATGACTCCAAATATAGCAAAAATAATAGGTTTTTAGGAGCAACTGATTCGGCTGTTCCTTGCGCAATGATGATACATTTAGCTAGAGTGTTACACGAATCTCTTCAAGATCAAAAGGCAAAGTCGAAAGATCTTAGCTTGCAGTTTATATTTTTTGACGGTGAAGAAGCTTTTGATAGGTGGACTAGTACTGACTCTCTTTATGGAAGTCGTCATTTAGCTGCTATGTGGAATAAAACACCCCCTACGCCTGGTGAA------------------AATAAATGCTTTAGAAATAATGAACCCATGGTTTCAAGGCTGGATCAAATGGATGTTATGCTTTTATTAGACTTAATCGGCTCTGCAAACCCCAAATTTTTCAATTATTTCCCTGAGACTAGTGCAATATACAACCGTCTTCCAAACATTGAACAAGAAATGAATCTTTTGAAGTCCTTAGAAAGTCATCCACCTCAGACCAACACTGATTATTTTGATACCAGAAGCTCTTGGGCTCATGTTGAGGATGACCATGTTCCTTTCATGCAAAAAGGTGTTCCCATCATTCATTTGATTCCCAGCCCATTTCCTGCTGTTTGGCACAAGGATTCTGACAACAGAGATAACTTACACCACCCCACAATGGCTAACTTGAACAAAATTATCCGTGTTTTTGTGTCAGAATATTTGCATTTA [972]

Proto TGGGCCCACGAGTTGCGGCGGTTTGATCAAAATTCTTTGCTTACATTGGCTGAGACAAATAACCTCCATTATGATGATTTCAATAGAACCTTAGACAATTTATTAAAAATTCGGGTACCGGGAACTGAAGGTCACAGACAAGTCAAAGAATATATATTCAATTCTATGAAAGATCTTGGTTGGTATGTTGAAGAAGATGTATTTACATCTAGGACACCTCATGGAAGAAAGCCATTTACTAATGTTATTGCCACTCTCAACCCGAATACCTGTCGCCAGTTAGTGCTGGCGTGTCACTATGACTCTAAGTACAGTAAAAATAATAGGTTTGTAGGCGCAACAGATTCAGCTGTCCCCTGCTCAATGCTGATTCATTTAGCCAGAGTGTTGCACGAATCTCTTCAAGATCAGAAAGCAAATTCTCAAGATCTTAGCCTCCAATTTCTATTTTTTGATGGAGAAGAAGCTTTTGAAAGGTGGACCAGTACTGATTCTCTTTATGGAAGTCGACATTTAGCTGATCTTTGGAATAAAACACCACCTACCCCTGGCCAA------------------AATAAATGCTATAGAAATAATGAACCCATGGTTTCCAGGCTTGATCAAATGGATGTTATGCTGTTATTAGACTTAATTGGCAGTGCAAATCCCAAATTTTTTAATTACTTCCCTGAAACAAGTCCAATATATACCCGTCTTCCAAATATTGAACAAGAAATGAATTTGTTGAAGTACATGGAAAATCACCCTCCTCAGGCAAACACTGATTACTTTGATACCAGAAGCTCTTGGGCTCATGTTGAGGATGACCACGTTCCTTTTATGAAAAAAGGAGTTCCCATCATTCACCTGATCCCCAGTCCATTTCCTCACGTTTGGCATAAGGACTCTGATAATAGAGATAACTTACACCACCCAACAATAGCTAACTTAAACAAAATTATTCGTATTTTTGTTTCAGAGTATTTGCATTTA [972]

[TICKFAST_NCHAR=1305;glycosyltransferase]

Ixodes TACGACCACTTAGAGTTTCCTGGAGTGGTACCGAGATCCTTCGTCGGCCCTCTTCTAGTCGCCATTTTCGCAGGCCCCTGGGTTGCAGTTAGCACATTCATGGGCTTGGAAAAAATTGTGGCTCAGTACATAGTGAGAGCTGTCCTGAGCACCATGGTTGTGTTTGCCTACCGGGAGTTCTGCCGTGCGCTTCAAAAAGAATTTGGGGGCGTGATGACCAACTGGCTGACGCTGCTGACGGTGTCGCAGTTTCACTTTGTGTTCTACATGTCGCGGCCTCTCCCCAACACGTTTGCCCTCGTGTTTGTCCTGCTGGCGTACAGCTTCTGGGTTGAGCAGAGGCACGTTCCCCTCATCCTGTCCAGCGGAGTGGCCGTCCTCGTGTTCCGCACGGAGTTGGCCTCTTTGCTCGGCATCATCCTCCTCATCGAGCTGTTCTCGGGGAGGCTGGGCATTCTCAAGCTCCTCAAGTGGGGCATTCCGACTGCAGTTGTTCTGTTAGGGGCCACGATCGTGATCGACTCTTACTTCTGGCAGCGCTGGCTGTGGCCAGAAGGAGAAGTCCTCTGGTTCAATCTGGTGCTCAACAAGAGCTCCGAGTGGGGTACTTCACCCTGGTCCTGGTACTTCTACTCTGCCATCCCGAGGGCACTGTGCAGCTCGCTGATACTCCTTCCAGTGGGAGTGTGGGTCGACTCGAGGGTGAAAGTGATGCTCCTTCCGGCCGTCGGATACGTGATCCTCTACTCCTTCCTGCCCCACAAGGAGCTCAGGTTCATCGTCTATGTGTTTCCCCTCTTCAATGTGGCAGCTGCCAGGGCCTGTCTCTTCGTCTGGAACAGCAGGCTCAAGTCCAGCTGGCACGTGGTGTTCACGCTGGGCGTGTGCATGCACCTGGTGGCCAACCTGTGTGCCACGGGCCTCCTCCTCTACATCTCCAGCTACAACTATCCCGGGGGACACGCACTCAAGAAGTTGCACGAACTCGAGGCCGGCTTTCCGGTTGCCAACATCCACATCGATGTGTATTCCGCTCAGACAGGAGTCTCCCGATTTGGAGAATTGAATCCTCACTGGAGGTACAACAAGACTGAGAACCTGGTCGCGGGAGGCCTGACCATGCACAGCTTTACGCACCTGCTGGTGGAGGGAAAGTCGATGCACAGCAGCTCAGTGGTGCCCTACCGGGAGACGCACGAGCTCGTGACGGCCGTGGAGGGCTACAGCCACGTGCGGCTCAACTACGCTGCCTTCCCGCCCGTGCTCGTGCGCACGCGGCCCAGGGTCCTCGTCTTGCGGCGCAGG [1305]

Siro TATGATCATTTAGAATTTCCTGGAGTCGTACCACGTTCATTTTTAGGACCAATCGTATTAGCTTCCTTATCGTATCCTCCGGTATTTCTACTGAAAATCTTTGGTCTATCAAAGTTCTGGGCACAGTATGTAGTTCGTGCTACTTTGGCAACCCTGGTAATCCTGGCCTTCCGGAAATTTCGACAATCGATTCAAAAGGAATATGGAGTTGTCACGGCGACATGGTTTACCTTTATTACCGTTTCCCAGTTCCACTTCATGTATTATTTGTCACGAACTTTGCCGAATACTTTCGCCCTTGTTTTAGTTCTATTGGCATTTCATTTCTGGTTTGAGCAAAGGTACAAGATGTTAATTTTCACGAGTGCAGCTGCGACGCTAATATTTCGTTTTGAATTGGCTCTACTTTTGGGCACCATCGTCTTAATCGAGTTGGCCAATAAGAGACTCAAATTTTGGCAACTCCTGAAATACACCATTCTCTTTGGTTTGGCTTTGATAGGCTTGACGGTTGTTGTGGATTCTTATTTTTGGCGACGGTTAGTTTGGCCTGAAGGGGAGGTCGTCTGGTTCAATATTATTCTTAACAAGAGTCACGAATGGGGTACCTCACCATGGGCCTGGTATTTCTATTCGGCAATACCTCGTGCATTAAGTTCCTCTATATTTCTGGTTCCACTTGGACTACTAATGGACAAGAGAGTCTCATTCTTCCTGTTTCCCGCACTACTCTTTGTCACCCTGTATTCCTTCTTACCGCACAAAGAGCTGCGTTTTATTATTTACGTCTTTCCTTTGCTAAATTTGGCAGCTGCGCAAGCTTGTACGTATATATGGAATCATAGATTAAAGTCAATGTATTACGTCTTCTTTACCTTGATGGTTTGCCTCCATCTGGTGGTGAATTTATTCTCCACAGTATTACTGCTTCAAATCTCTTATTATAACTATCCAGGCGGTTGGGCTTTAACGAGACTTCATCGCTTAGAAGGACATCGAACAGATGTTTATCTTCATATTGATGTCTACTGCGCTCAAACAGGCATTAGTAGGTTTGTGCAGAGCCACTCTCATTGGAGGTATAACAAGACAGAAAATCTGACTCCCGGTAGCCCAGAAATGATGGCGTACACCCATCTATTAATGGAAGGAAGATCTTTTTACAGCAGTGCAGTTGTTCCTTATCAAGATACCCATGAATTATTAGACAGTGTGCCAGGCTTTAGTCATTTTAAGTTGGACTATGCCACTTTTCCACCTGTGAAAATACGGACAAAACCAAAAATTCTTATACTGAAAAGAAAA [1305]

Sclero TATGACCACTTGGAGTTTCCTGGGGTGGTTCCCCGTACCTTCCTGGGACCCCTGGCACTGGCGTTTATCGCCTATCCGGGAGTCTCGCTTATTCAACTTCTCAACATCACCAAGTTTCTGGCTCAGTTTGTGGTGCGTGGCTTCCTGTGCCTGCTTGTACTGATGGCATTCTACCGCTTCCGGGTCTCCGTCCAGCGCCAGTTTGGGGCCGTGACCGCGACGTGGTTGCTTCTGATCACAGCCTCGCAGTTTCATATGGTTTACTACATGTCCCGCACCTTGCCCAACACCTTTGCTTTCGCTCTGGTGCTGCTCTGTTACCATTACTGGTTGGAGCAAGATCATCGGGCCCTGGTGGTGGTGTCGGCAGCAGCGGCCATTATTTTCCGGGCAGAGACGGCTATCCTGACAGGGCTCATGCTCATGTTGGAACTCTTTTCTGGACGGCTTCGCCCTCTCCAACTTCTCAAGTTTGCTGTTCCCTCTGCCATCTTCATGCTGGGTCTGACCATCTCTGTGGATTCTTATTTTTGGCAGCGCCCTCTGTGGCCGGAAGGGGAAGTGTTGTGGTTCAACCTGATTCTGAACAAGAGCGGAGAATGGGGTAGAAGTCCCTTCGCCTGGTACTTCTATTCAGCCATTCCGCGAGCTCTCAGCGCCTCGGTCTTCCTGGTGCCAGTGGGGGCCATGGTTGACCGAAGAATCTCGCTTCTCCTGGTTCCTCCCTTGGGTTTCGTACTTCTTTACTCCTTTCTTCCTCACAAGGAACTGCGCTTCATCGTCTACGCCTTCCCTTTGCTCAACGTGGCGGCCGCCAGGGCTTGCACATTCATCTGGCACAGTCGTCTGAAGTCGTACCTATGTGTGGGCCTGACCCTCCTGACCTGCCTGCACCTGGTGGTGAACTTGGCCACCACTTCTCTGCTGCTTTACGTCTCCTATCACAATTACCCCGGGGGTTGGGCCATGGCCCGGCTTCATCAGCTAGAAGGGCACCGTACTGGTGTGCAAGTACACATGGATGTTCTGACTGCTCAAACGGGAGCATCTCGCTTCATCCAGGTTAACCCACACTGGAGGTACAGCAAGATCGAGAACCTGTCACCGGGAAGTGCGGAGATGATGTCCTTCACGCATCTGCTACTAGAGGGCCGCTCCTCGTACAGCAGCAGTCATCAACCGTACAAGGACAGCCACAATATTCTGGATACGGTGCACGCCTTCAGCCACTTCAAATTAGACTACACCGTTTTCCCTCCCGTCGTCATCAGGACCAAACCCAAGATTCTGATTTTGCAGCGGAAA [1305]

Sitalc TATGACCACCTGGATTACCCCGGAGTGGTTCCCCGCACGTTCCTGGGTCCTCTGATTCTGGCCTTCATCTCGTACCCAGGGGTCTCACTCATCCAGCTTCTCAACATCACCAAGTTTCTGGCACAGTTTGTGGTTCGGGGATCTCTGACGCTACTGGTCTTGTTGAGTTTTTACAAGTTCCGAGTCTCGATCCAGCGTCAGTTTGGTCATGTGACTGCAACGTGGTTGCTTTTAATCACTGCTTCTCAGTTCCACATGGTCTACTATATGTCGCGCACTTTGCCAAACACATTTGCACTGGTTTTAGTTCTTCTGGCCTACCACTTCTGGCTTGAACAGGATCACAGGAGCCTGATTGTGACAGGGGCGGGCGTGGCCATCATCTTCCGGGCGGAGACTGCCATCCTGATGGCCCTCATGATTATGATCGAAGTCTTCTCTCGAAGACTATCCGTCTTGACTCTCATCAAGTATTCTCTTCCTGCTTCAGTTCTTATGTTGGGTTTGACTAGCGCGGTGGACACATTTTTCTGGCAGCGCCCTCTGTGGCCAGAAGGGGAAGTCCTGTGGTTCAACCTTATTCTCAATAAAAGCGGAGAGTACGGGACCAGTCCGTTTGCCTGGTATTTCTACTCTGCCATTCCTCGGGCGTTGAGTAGTTCCGTGTTCCTGGTCCCGCTGGGTTTGTACGCAGATCGTCGTGTGGCCTTGCTCCTCCTCCCTCCCCTCAGTTTCACCTTCCTCTACTCTTTCCTCCCTCACAAGGAGCTACGATTCATCATCTATGTTTTCCCGTTGCTCAACGTTGCTGCTGCCAGAGCTTGTACATACATTTGGCAAGGTCGCTTGAAGTCGTACGCAGCGCTGTGTTTGACTTTGTTAACGTGTCTCCACCTGATAGTCAACATAGGAACCACTTCGTTGCTTCTTTACGTTTCCCATTATAACTACCCTGGTGGCTGGGCAATGGCCAGGTTGCATCAACACGAGGGACACAAAACGGGTGTGTCTGTCCACCTAGATGTATTCACCTGCCAGACTGGAGCCTCCAGGTTTATAGAAATCAACCCTCACTGGAGGTATAGCAAAGCTGAAAACCTACCTCCTGGTGGTCCCGAGCTTATGTCGTTCACCCATTTAGTACTGGAGGGTCGCTCCGCGTTTAGCAGTAGCGTTCAGCACTACAAAGACACTCATACCATAATAGACACAATCCCTGCGTTCAGCCATTTTAAACTGGACTATACGATGTTCCCACCAGTGGTTATAAGGACCAAGCCAAGGATACTAATTTTAAAACGGAAG [1305]

Trogulus TTTGACCATCTCAAATTTCCCGGTGTTGTACCCCGAACTTTTATTGGTGCCATAATAATCTCAGTACTGGCCTCACCTTTTGTAACCTGTATAAAGCTATTGGGAATTAGCAAATTTTATTCACAGTATGTTGTTCGAGCAACTTTAGGGCTTGTAGTGATCCTCGCCTTCCGCAAGTTCCGTATTTCAGTGGAAAGTAAATTCGGAGTTCCAACAGCAACATGGCTGACATTGATAAGCGCTTCTCAGTTCCACTTTTCTTTTTATTTGACCCGAACATTACCAAACACTTTTGCAGTTGCATTAGTTCTTTTGGGGTATCACTTTTGGTTAGACCAAAAGCACAAGAACTTGATTATCACTGCTGCAATCTCTGCTATTATCTTTCGAGCTGAAACAGCATTACTACTTGGAACTATAGTTCTTATAGAGCTAGCAACTCGCCGTGTTTCTTTACAGAATGTTTTTAAATGGGGAATTTTATCAACTGGTGTTTGTTTTGTTGCTACATATACTATTGATTCTTATTTTTGGAGGAGACAATTATGGCCTGAATTTGAAGTCTGGTATTTCAATGTGGTGCAGAACAAGAGTTCAGAGTGGGGTACATTCCCATGGGCCTGGTATTTTTATTCGGCCCTTCCTCGTGCCCTTGGCTCTTCCATTCTTTTGTTGCCTCTAGGCTGGTTTTCAGACAGGAGGGCTACCGTCCTGTGTATACCAGCATTACTCTATATCATTGCTTTCTCTTTTCTGCCTCATAAGGAAATGAGGTTCATTGCTTACAGTTTTCCTTTGCTGAACATTGCTGCTGCTTCAGCTTGTAATTATATATGGAATCATCGGCTGAAATCATATTTGTGGGCCTTCTGTTGTTTATGTACATGCTTCCACTTGGTGGTTAATGTGGCTACAACATCATTACTACTGTATATTTCTCATAGCAATTATCCGGGTGGTTGGGCATTGCATAATTTGCATAAGTTGGAACATAATCAGAGAGATGTTCGTGTACATATAGATGTATTTAGTGCTCAGACAGGAGTGTCTAGGTTTACTCAACAGGAGCCAAACTGGAGGTACTCCAAAGATGAGAGGATTACTCAGAGTAAAGAAAAAATGTTACGATTTACTCATCTTCTGGTGGCAGGAAAATCAGCTTACAGCAGTAGTGTTGCGATGTATCGCGACACTCATGATGTGTTAGCCATAATTGAAGCTTTTAGCCATCTGAAGGTGGATTATACTGCTTTTCCCCCTGTGCACGTCAGAAGAAAGCCAAAAATTCTTATTCTGAAGAGGAAA [1305]

Ortho TTTGATCATCTGGAGTTCCCGGGTGTGGTTCCTCGTACATTCATTGGAGCTTTAATAGTATCGGCCATTGCATCGCCTTTTGTATTATGTATGAATTTATTGGGAATTTGCAAATTTTATGCACAGTATGTCGTCCGAGCGACACTGGGGCTACTAGTCATTTTGGCATTTCGAAAATTCCGAATCTCAGTTGAGCAGAAGTTTGGAATTCCTACTGCAGTGTGGCTGACTTTGATCAGTGCATCACAGTTCCATTTTGCATTCTATTTGACAAGGACATTACCAAACACCTATGCGGTTGGGTTAGTGCTTTTGGGTTACCATTTCTGGTTAGACCAAAAACACAGGAAATTGGTTATTACAGCTGCAATCTCAGCTATTATTTTTCGAGCCGAGACAGCTTTATTGCTTGGAATAATGATCCTTTTAGAAATTCTGACCAGACGTTTGTCACTCTGGAAAGTCATAAAATGGGGGATTTTGTCAGGAGGGTTTTGTTTTCTTGCGACCTACACAGTAGATTCATATTTCTGGGGGCGGCGTTTATGGCCGGAAGGCGAAGTTTGGTACTTTAATGTTATAGAAAACAAGAGCTCGGAATGGGGAGTATTACCTTGGGCTTGGTATTTCTATTCAGCTATACCCCGGGCTCTGGGATCTTCAGTACTTTTAGTACCGTTAGGGTGGTTTGCCGATAGAAGAACAACTGTTTTATCACTCCCGGCGTTGCTTTACTTAATTGCCTTTTCGTTTCTACCTCATAAGGAACTGAGATTTGTGGCATATTGTTTTCCCCTTCTGAATATTGTGGCAGCCTCTGCTTGCAATTATATATGGAACCACAGGTTAAAGTCTTATTTCTGGATTTGTTGCTGTTTGTTCACCTGCCTACACCTCATTATAAACCTAGCAACAACATCGTTACTACTCTACATCTCTCATAGTAATTATCCTGGTGGTTGGGCAGTTCATAATTTGCATCATCTTGAAAACAATCAAAAAGATGTTTATGTTCATATTGACGTGTTTACTGCTCAAACTGGAGTATCCAGGTTTACTCAGCTAGATCCAAATTGGAGGTACTCGAAAGACGAGAAGATATCGCAGAATAAAGATAAAATGCTGCGTTTTACTCACCTCCTGGTTGAGGGAAAGTCAGCATACAGCAGCAGTGTCTCATCCTACCGTGATACACACGATGTACTTGCTATAATTGAGGCCTTCAGTCATCTGAAAGTTGACTATACCGCATTTCCACCTGTTCATGTCAGAAGAAAACCAAAGATTTTGTTATTGAAACGGAAA [1305]

Hespero TATGATCACTTGGAGTTTCCTGGAGTTGTTCCTCGTACATTCATTGGCGCTCTCTCTGTGGCCATTATTTCAGCACCATTAGTTATTTTTATAAATGTAATAGGAATAAGCAAGATATATGCTCAATATGTTGTACGTGGAATCTTAGGTTTGCTGGTTGTTCTGGCTTTTCGGAAATTTCGAATTTCGGTTCAAAAATGGTTCGGAATTCAAACTGCAACATGGTTGATACTAATTACAGCATCACAGTTTCACTTTGTATTTTATTTAACTCGAACTCTTCCAAACACTTTTGCTATGATATTGGTTCTTTTATCTTATCACTATTGGCTGGAACAAAATCATCGCTCATTAGTGGTTATGACGAGTATGTCTGCTATAATTTTTCGGGCGGAAACAGCTTTACTTCTAGGCAGCATTATATTGATTGAGTTACTATTGAAACGAGCATCTCTTTTGAAGGTGCTAAAATGGGGAATTCTAGCCTCAACCATTTCTTTTGTCTTTACATTTGGCATTGACTCATTTTTCTGGAGACGCCACTTGTGGCCAGAAGGAGAAGTTTGGTGGTTCAATGTGGTGCAAAATAAGAGTGGTGAATGGGGTATTCTTCCTTGGTCTTGGTACTTTTACTCTGCATTACCACGTGCTCTTGGAACTTCTTTCTTTCTATTACCTGTCAGTTGGATTGTTGATAAGAAGGCTACTGTGCTTACATTGCCCGCATTGGTTTACATTGCTGCATTTTCCTTTTTGCCTCACAAGGAACTTAGATTTGTAGCTTACAGCTTCCCATTATTAAACGTTGCAGTATCATCAACATGCAATTACATATGGAATCATCGCTTCAAATCTTATGGTTGGATGTGCTGCTGCCTTCTCACCTGTCTTCATTTGCTGGTGAATTTATCCTGTACCTCTTTGTTACTGTACATTTCCCATAGTAACTATCCCGGTGGTTGGGCAATTGATGCAGTTCACAAGTTGGAACATGAACAAAAAAATGCGAGTGTACATATTGATGTTTTTTGTGCCCAAACTGGAATATCAAGGTTTTCAGAAATATATCCTACCTGGAGGTATAGCAAAGAAGAGCATCTTTCTCCCGGAAAGTCCAAGATAACTACATTCTCTCATTTACTAATAGAAGGGAAATCTGCATACAGTAGTAGCGTTTTGCCATATCGAGAATCACACGAAGTACTTACTGTTATTGAAGCTTTCAGTCACTTGAAAGTTGACTATACTGCCTTTCCACCATTGCTTGTTAGAAGAAAACCAAAAGCTTTGGTATTAAAGAAAAAA [1305]

Leiobunum TTTGACCATCTTGAGTTTCCTGGTGTTGTTCCCCGAACGTTCATTGGTGCCATCGTAGTTTCTATATTGTCATCACCATTTGTTTACTCTATACAAGCAGCGGGATTGAATAAGTTTTATGCTCAATATGCTGTTCGTTTTGTTTTAGCTTTTTTAGTGATAGTTGCTTTTAGAAAGTTTCGAATATCTGTTCAAAAACATTTTGGTGAAGTCACAGCTACATGGGTCACACTGATGAGTGCCACGCAATTTCATTTTGTTTTCTACATGTCCAGAACCTTGCCAAATACATTCGCCTTAATTTTAGTTTTATGGGGTTTCCATTTTTGGCTGGATAGAAACCCCAAATACTTAGTAATAACTACTGGAGTAGTTTCTGTCATATTCAGGGCTGAAACTGCATTACTTTTGGGAACTCTCATTTTAATTGAGTTATTTTTTCGAAGACTGTCATTTTTTAAATTAATTCAGTGGGGCTTATATTCTACTCTCACATCTTTAGCTTTAACATTTACAATTGATTCCTACTTTTGGAGACGCAACCTGTGGCCTGAGGGTGAAGTTTGGTGGTTTAATGTTGTGGAAAACAAGAGCTCTGAATGGGGGACTCTTCCTTGGGCGTGGTATTTTTATTCTGCACTCCCCAGAGCTTTGAGTTCTTCAATTATTCTAGTTCCTGTAGGATTGGTTGTGGATAGCAGATCTCGTATTTTGGCATTTCCAGCTTTGTTCTACATTTTTCTGTTTTCTTTTCTACCTCACAAAGAATTACGTTTCGTCGCCTATGCCTTGCCCTTGCTCAATGTTGTAGCTGCATCAGCTTGCAACTACGTATGGAATCATCGTTTGAAATCTTTTGTCTGGGTGGTGTTTTGTTTAATGACATGCTTGCATTTGGTGGGCAATTTAGCCACTACATCACTCCTACTTTACATTTCGCATCATAACTATCCTGGTGGGCAAGCCCTCCATATATTACATGACATAGAAACTAATTTTGACGACGCCCATGTTCATATTGATGTGTATTCAGCTCAAACTGGGGTGTCTAGATTTGGCCAAATTAATACAAATTGGAGGTACAGTAAAGATGAAAACCTGAAATTTCCGAAAGATAAAATT---GGGTTCACTCATTTATTACTGGAAGGAAAATCAGTCTACAGCAGTAGCATCCTTCCATTTCGTGATAGTCATAAAATACTGACTACTGTCGAAGCTTTTCAGAATCTAAAAGTGGACTATACTAGATTTCCCCCCGTCCATGTACGAACTAAACCCAGAATCATGCTTTTGAAACGAAAA [1302]

Proto ???????????????????????????????????????????????????????????????ATTATATCATCACCGTTTGCTTACGCTATTCAAGCAGCAGGATTGAATAAGTTTTACGTTCAATATGTTGTACGTTTCGTATTAGCATGCTTGGTTATAGTTGCCTTTAGAAAGTTCCGTATATCCATTCAAAAACATTTTGGAGATGTAACAGCCACATGGGTAACCCTGATCAGCGCCACGCAATTTCATTTTGTTTTCTACATGTCCAGAACCTTGCCAAATACATTTGCCTTAATTTTAGTTTTATGGGGTTTTCATTTCTGGCTGGATAGAAACAATAAGTACTTGGTAATAACTACGGGAATAGTCGCTGTCATATTCAGGGCCGAAACTGCGTTACTTCTGGGAACTCTTATTTTAATCGAGGTGTTTTTTAGACGGCTATCATTTTTTAAACTCATTCAGTGGGGTTTATATTCTACCCTTACATCATTAGCTTTAACATTTACCGTTGATTCCTATTTTTGGAGGCGTAATCTGTGGCCAGAGGGTGAAGTTTGGTGGTTTAATGTTGTGGAGAACAAGAGCTCAGAATGGGGGACGCTGCCTTGGGCTTGGTATTTCTACTCCGCAATTCCCAGAGCATTAAGCACCTCAATTATTCTAGTTCCTGTAGGATTGGTTGTGGATAGCAGATCTCGTATTTTGGCGTTTCCAGCTTTCTTCTATATTTTCCTGTTTTCCTTTCTCCCTCACAAAGAATTGCGTTTCATTGCCTATGCCTTGCCCTTGCTCAATGTTGTAGCTGCATCAGCTTGCAAC??????????????????TTGAAATCATTTGTTTGGGTGGTGTTTTGTTTAATGACTTGCTTACATTTGGTAGGAAACCTAGCCACTACTAATCTTCTTCTTTACATCTCCCATCACAACTATCCCGGTGGACAAGCCCTTTCTATATTACACGACATAGAATCTAATGTTGATGATGCTTATGTTCATATCGATGTGTACTCTGCTCAAACCGGAGTGTCCAGATTTGGCCAAATTAATTCTAATTGGAGATATAGTAAAGATGAAAACCTCAAAGCACCGAAAGATAAAATT---GGGTTCACCCATTTATTACTGGAAGGAAAATCTGTTTACAGTAGTAGCATTCTCCCCTACCGCGATAGTCACAAAATAATGGCCACAGTCGAAGCTTTTCAGAATCTGAAAGTGGACTATACCAGATTCCCCCCTGTACATGTCCGGACTAAACCCAGAATTATGCTTTTGAAACGAAAA [1302]

[TICKFAST_NCHAR=546;golgi SNAP receptor complex member]

Ixodes GAAGATCTAAGGAAACAAGCCCGGCACCTTGAGAATGAAATCGACCTGAAGCTTGTTTCCTTCAGCAAGTTGGGCACGGGCCTTGGCTCTCGAGAT------GTCAAGAGCGACGGCTTGGACACAGCTCCCCTTCTGAGC------------AGTGACCACATGTTTGAGACCATGACCCTGGAGATTGAACAGCTCCTCTCAAAGCTGGGGGACGTGAACGACCAGATGTCCCAGGGCCAGCAGCTCCCGTTTGGG------CAGCACGCCCCAGGGGGCGCCACGGTGGTACACACCCTGCAGCGGCACAGAGACATCCTGCAGGACTATGCCCGGGAGTTCCAGAAGACGCGCGCCAACGTCCAGGCACAACGCCAGCGGGACCTCCTGCTCGGCTCGGTGCGCAAGGACATAGAATCATATAAGAACTCTTCCAGCTTGAGCCGGCGGTCAGACGGGTTCCTCAAGGAGCACGAGCACCTCAGAAATTCGGACAGGATGGTTCATGACCAAATCAACATTGCAATGCGAACAAAGGATGAG [522]

Siro GAAGATCTGAGAAAACAAGCAAGACAACTGGAAAATGAACTCGATTTAAAATTAGTATCATTCAGCAAATTAGGAACCAGTTATGGGAACAAAGAT------TTGAAAAATACAAGCCTCGACACGTTGCCTTTGCTTGGCGGGACCAAC---AGCGAACATGTTTTCGAAACGATGGCGCTTGAAATCGAACAGTTACTTTCTAAATTAACGGAAATCAATGACAAAATGTCGGACTACACCCAGATGCAA------------GGTCAAACGGCGCCTAGCGCGACTCTTCTGCACACGATCCACAGACACGATGAAATTCTGAAAGACTATTCCAGAGAATTTCGTAAAACCCAAGAAAACTTAAAAGCACGTCGAGAGAAAGAGGAGTTATTAGGTTCCGTACGCAAAGACATTGACACGTACAAGAATTCTTCGGGTCTTAATCGACGAAACGAGATGTATTTGAAAGAAAATGAACATATACGAAGTTCTGATCGACTGGTAGATGAACAAATTAGAATTGCAATGAAAACTAAAGATGAA [525]

Sitalc GAAGATTTACGTAAACAAGCACGTTTACTAGAGAACGAAATCGACGTAAAACTACTGTCATTTAGTCGTCTTGGAGCAGGGTACGGTAGCTTACAG---------------ACGCCACCGGAATCAGCTCCTCTACTAAACGGAAGTCGT---AATGAACGTATGTTTGAAACTATGGTCCTTGAAATCGAACAGCTGTTGGCCAAGTTAACCGAAATCAACGACAAAATGTCCCCTTTTGGTGCAACGACATCAACAGCGGGTGCTTCAGGTTCTCCCAATGTAATGATAGCACACACTATTCAGCGACATCAAGATATTTTAAAGGACTATTCGCTCGAATTCCGAAAAGTTCGAAACAACATCCAAGCAAAGAGGGACAAGGAAGAGTTAATGGGTTCTGTCCGAAAGGACATTGATACTTTCAAGTTAAACACTGGCGTAAATCGCCGAACAGAACAGTATTTAAAAGAGCATGAACACATTCGCAGTTCGGATAAATTGGTCGATGAACAGATTAAAATAGCCATGAAAACTAAGGATGAT [528]

Sclero GAAGATCTTCGCAGACAAGCACGTTTATTGGAAAACGATATCGACGTCAAATTATTGTCGTTTAGCCGTCTAGGGGCTAACTACAGCAGCTTTCAG---------------CCCCAACCCGAATCAACGTCTCTGTTGAATGGGAGTAGCAGAAATGAACGAATGTTCGAACCCATGATGTTGGAAGTTGAACAATTATTAGCAAAGCTGACGGAACTTAATGACAAAATGGCCCCGTTTGGTACCCCCTCTTCAAACTCC---CCTTTAATGCCAAATTTACAAATGATGACTCATACTGTTCAACGCCACCAGGATATCCTGAAAGACTATACACGAGAGTTCAGAAAAGTGCGTTCCAATATCCAAGATAAACGTGACAAAGAGGATCTGTTGGGATCAGTCAAGAAAGATATTGACGCATTCAAAGGAAACTCTGGATTGAATCGCAGAACGGAACTTTATTTGAAAGAACACGAGCATATACGCAGTTCCGATAAACTGGTCGATGAGCAAATTAAGATCGCTATGAAGACTAAAGATGAC [528]

Trogulus GAGGATCTAAGGAAGCAGTCGCGCCACTATGAAAACGAGATTGACATGAAGCTGGTCTCCTTCAGCAAGCTGGGCACGGGATACGGCAGCCGCGAATTCGTTTCCAACAGCGCCAACCACGAGGCAGTCCCCCTGCTTAACGTCTCCAAC---GCTGATCATGTCTTCGAGACGATGTCTTTGGAGATTGAGCAACTTGTAGTCAAGCTAACAGAGATCAACGACAAGATGGCGTCCGCTGGACAAACG------------------------TCACCCAATGCGGCATCCGCCCACACGATCCAGAGACACAGGGAGATCCTCCAGGACTATTCCAAGGAATTTCAGAAAATCAAGGCGAACATCCAGGCCTGCAGGGACAGAGAGGAGCTCCTTGGTTCCGTCCGCAAGGATATAGATTCGTTCAAGAACTCTTCAGGGCTGAATCGACGGACTGAGATGTACCTGAAGGAGAACGATCACATCAGGAACTCCGAGAGACTGGTCGATGAGCAGATCGGTATCGCTATTAAGACGAAAGAGGAT [519]

Ortho GAAGACCTACGGAAACAAGCACGGCAGTATGAAAACGAAATCGACCTTAAGCTTGTCTCTTTCAGCAAGCTAGGCACAGGATATGGTGGCCGAGAT------TTCACTAACAGTAATCATGAAACAGTTTCACTACTTAATGCAACAAAC---ACAGACCACATGTTTGAAACCATGGCCCTAGAAATTGAACAGTTAATCTTGAAACTCACCGAAACCAATGATAAAATGGCTTCAGCTAGTCAGATA------------------------ACTCCTAGTGCAGCAGCAGCACACACCATACAAAGACACCGAGAGATACTGCAGGATTATTCTAAGGAGTTCCAGAAGATTAAAGCTAACATCCAAGCCCGTAGGGATCGTGAGGACCTCCTGGGCTCGGTCCGAAAAGACATTGACTCTTTTAAAAACTCCTCTGGTTTGAACCGGCGAACGGAAATGTTTCTGAAAGAGCACGAGCACGTCAGGAGTTCGGAACGACTCGTAGACGAGCAGATTGGAATTGCCATCAAGACCAAAGAGGAT [513]

Hespero GAAGAGTTGCGCAAACAAGCCCGTCAATTCGAAAACGAAATAGACTTAAAACTGGTGTCGTTCAGTAAATTGGGCACGGGAAACCGCGACTATTCC------------AATTCCAATCATGAAACGGTGGCGTTGCTTAGCAGCTCCAAT---AGCGATCACATGTTCGAAACAATGGCATTGGAAATAGAGCAATTGCTTTTAAAATTAAGCGACACTAATGACAAGTTAACGAGCGTGAGTCAATCCAGC------------------TCGCCCATCAGTCCCGCTACTTCCCACATGATTCAAAGGCATAGAGAAATACTTCAAGATTACTCTAAAGAGTTCCAGAAAATCAAAGGCAATATTCAAGCCCGAAGGGATAGAGAGGACCTGTTGGGTTCTGTGCGCAAAGACATTGATTCTTATAAACATTCGTCGGGTTTAAACCGAAGAACCGAAATGTATTTGAAAGAGAACGAGCATTTGAGAAGCTCCGAACGACTCGTGGACGAACAAATCGGAATCGCTATGAAAACTAAAGAAGAT [513]

Leiobunum GACGACTTGAGAAAACAAGCCAGGCTTCTGGAAAATGAAATCGACCAGAAGTTAATTTCATTCAGTAAACTTGGAACTGGTTACGAGAACCGCGAA------TATGGAAACTCAAACTTGGACACCGTGCCTTTATTATCAGGGGCTAAC---AGCAGTCACGTATTTGATACCATGGCGCTGGAAATAGAGCAACTTTTATCCAAGTTAACAGATCTAAACGACAGAATGACCTCCCTGTACCAAAATATA------------ACATCAACGACGCCTAATCATTCCTTGGAACATACGATACAACGCCATAGAGAAATTTTAAAGGACTACTCTTTGGATTTTAAGAAGATTCGGAGCAATATACAAGCCAGAAGGGATAGGGAAGAACTTTTAGGATCAGTTCGTAAAGATATTGACGCTTACAAAGTATCATCCGGACTGAACAGAAGGTCTGAGCTTTTTTCCAAAGAACACGAACATATTAGAAACTCCGACCGACTTGTGGATGAACAAATCAGGATTGCAATGAGAACTAAAGATGAT [525]

Proto TCAGATCTCAGAAAACAAGCTAGGCTTTTGGAGAATGAAATCGACCAGAAATTAATATCATTCAGCAAACTAGGAACCGGTTACGAAAACCGCGAA------TATGGGAACGCAAGTTTGGATACGGTGCCTTTATTATCAGGCGCTAAC---AGCAGTCACGTATTTGACACCATGGCATTAGAAATCGAGCAACTTTTATCCAAATTAACAGATCTGAACGATAGAATGACATCTTTATACCAAAATATA------------ACGTCGACGACACCGAACCATTCCCTGGAACACACTATACAACGCCACCGAGAAATTTTGAAAGACTACTCTTTAGATTTTAAGAAAATCCGCAGCAATATACAAGCGAGAAGGGATCGAGAAGAACTTTTAGGATCAGTTCGTAAAGACATCGATGCTTACAAGGTCTCGTCCGGGTTAAACCGAAGAACCGAGCTATTTTCTAAAGAACACGATCATATTAGAAACTCCGACCGACTTGTGGACGAACAAATAAGGATCGCAATGAGGACTAAAGATGAT [525]

[TICKFAST_NCHAR=744;heat shock protein 70 (HSP70)-interacting protein]

Ixodes ATG---GAAAGCATGGAAAAGGAGCAGCTGCTGCAGTTGCAGGCCTTCGTGGAGCTCTGCAGGCACAAGCCAGAGGTGCTGCACAAACAGGAGCTGGCTTTCTTCAAGAACTACCTGGAAAGGTGGGGCGCCTCGTGGTCGAATCGG------------------------------------------------------------------------------------------------------------------------------ATGGTGTCGCGTAAGGAGTTGGACAACACCGGCGTGATCGAGCCGGAC---AATGAACCGCCCTTTGAAAATGGTGATGCTAGCATCGAGGTGACGGACGAGATGCTGGAAGAGAGCTCTGAAAAGAGGTCCCAAGCAATGGAGCTTCAAAATGAAGGGAAACTGGAGGAGTCGATAAAGCTGTGGACGGAGGCCATTTTGAAAAACCCGTCCGGTGCCGTTCTTTTTGCCAAGCGTGCCAATGTCCTGCTGAAACTGGAGAAACCCAACGCTGCCATCAGGGACCACGGGCTGGAGGACGCCAACCGGGCACTGGAGTTGAACCCAGACCAACCCCTGGCCTACAAGATCAGGGGACGAGCCAACAGGCTGCTTGGGAACTGGGAAGAAGCTGCCAAGGATCTTGCCATGGCCTGCAAGCTGGATTACACGGACGAGGCCAACGAGTGGCTCAAGGAGGTGATGCCGAAT [612]

Siro ATGGCATCTCAAATGAGCCCAGAGGATTTGGTTCAACTGCAAGGGTTTATACAAGTGTGTCGAAGCCAACCCGACATTCTTCACATTCCCGAACTGTCGTTTTTCAAAACTTACCTCGAAAGCATGGGTGCTAGAATTCCTGAAGTT---------CGAAAACCTCCAACACCATCTAAGAAT---------------------------------------------GCAAAAGTTGAAGATCCTGAA---GTTGAAATACTGGAGGAAAGTGAAGAAAGCGAAATTGAGCTGGATAATTCGGGCATTATTGAACCAGAC---AATGACGACCCACAGATGATGGGAGACGATGAATTAGAAGTGACAGAAGAAATGTTGGAAGAAAGTGATGAAAAGCGGTCGGCTGGTCAGGAAGCTTTGAGCGAAGGAAAACTGGAAGAAGCTTTAGAGCTTTTTACGCAAGCTATTCTAAAAAATCCTCAGGGGGCAGCACTATATGCTAAACGTGCAGGGATAAATTTGAAGTTGTTAAAACCTAACGCTGCCATCCGCGATTCT---------------AACAAAGCTCTGTCGATAAACCCGGACCAAGCCTTTGCTTTCAAGTGTCGAGGACGAGCTCACCGGTTGCTTGGCGAGTGGGAAGAAGCGGCAAAAGATTTGGCAACGGCTTGCAACATTGATTACACTGACGAAGCGAACGAGTGGTTGAAAGAAGTCTTGCCGAAT [669]

Sitalc ATGGCTCACAACATGAAAGAGGCGCAGTTGAAGCAGCTCCAGGATTTTGTGGAGCTCTGTAAACATAAACCAGAGGTTTTGCATATTCCTGAACTCTCCTTCTTCAAGAATTACATCACAAGCCTTGGAGCTCAGGTGCCCGACTGC------------CCTCCACCAGCTCATGAGGAAGACTGCGGTTGCTCTTCGCCCAAGGCCACCCCATCAGCCCCGACGGAAGAAAAGCCAAAAGAAACACCT---GAGCCACAACCGGCAGAAAGTGAAGAAAGTGATTTGGATCTGGATATGTCTGGAGTGATTGATGGAGAC---AGCGATGAGAGCCAGGAGATGGGAGACGACAGCGTAGAGGTAACTGAAGAAATGATGGATCAGAGTTCAGAAAAAAGGTGTGAAGCGCAGGACGCCATGAACGAAGGTAAATTGGAGGAAGCCCTACAGCTGTTTACAGAAGCCATCTTGAAGAATCCCTCAAGTGCTGCCATATTTGCCAAAAGAGCTGGTGTTTTCTTGAAGCTTAACAAGCCCAACGCTGCCATTAAAGATTGT---------------AACAAGGCACTATCGCTTAACCCAGACCAGTCGGTGGCGTTCCGGTACAGAGGAAGAGCACACAG?CTACTTGGAAATTGGGAAGACGCCTCTCGAGACCTGGCCACAGCGCAGAGGATCGACTATTCAGATGAAGCCAACGAGTGGTTGAAAGAAGTCATGCCTAAC [711]

Sclero ATGGCATGCAACATGAAAGATAAGCAGCTGGAACAGCTTAAGGAATTTGTGACCTTATGCCAAGCCCAACCAGAAGTCCTGCACATACCAGAGCTTGCCTTTTTCAGAGTCTACATTGAGAGCATGGGAGGCAAGATTCCTGCTCCA---CCTGCAGCACAGTCCCCTACACCTGAAAGCCCAACATCTCCTACTGCTCAAAAGCCCACCCCTTCTGCTTCCAAAGAAAAAACAGCAGAAGTGCCGACACCCGAACCTCAGGATGATGAAAGTGAAGAGAGTGACCTAGAATTGGATATGAGTGGAGTTATTGAACCTGAT---GATGAGGCACCTCAAGAGATGGGGGATGACAGCATAGAGGTTACAGAAGAAATGATGGATGAAAGTGGGGATAAGAGGTGCCAAGCCCAAGAGGCATTCGGTGAAGGAAACCTAGAAGAAGCATTAAAATTGTTTACAGAAGCTATCCTGAAAAATCCAATCGGTGCTGCCGTATTTGCGAAGAGAGCAGGTGTGTATATTAAACTTGGTAAGCCAAATGCGGCTATCAGAGACTGC---------------GATAAAGCCTTAGCTCTCAATCCAGATCAAGTAGTGGCATTCCGCTACAGAGGAAGGGCACACAGGCTTCTTGGAAACTGGGAAACATCTGCGCACGACTTAGCGACAGCCCAAAAAATTGACTATTCTGATGAAGCAAACGATTGGTTAAAAGAAGTCATGCCAAAC [723]

Trogulus AAGATGGCGGGTGTCAACCACGCTCAACTTGTTCAGCTTCAGGAGTTTGTGGCACTCTGCAAGAAAGAGCCTGCTATTATGCATATGCCTCAACTAAAGTTTTTCAAAGATTTTCTTGAGAGCTTGGGAGCAAACATTCCGGAGGCC---ACCGAGACTGTAACCGAAGAACCTGAATCACCAAAACCAAATACTTCTTCAGAATCAAAAAATAAAGATCCGGAA---TCACTTTCATCTGACTCTGAA---ATTGGAGAACCCGAAAGTAGTGAAGAAAGCGAAATAGATTTGGATAATTCTCTTGTAATTGAACCAGAT---GCAGATGAACCTTATCCTATGGGTGATGATAGTATAGAGGTTACTGAAGAAATGATGGATCAAAGCTCCGAGAAGCGGGGTGAAGCTCAAGAAGCTCTTTCTGAAGGTAAATTGGATGAAGCCATTAGCCTTTTTACTGATTCCATACTGAAGAATCCACAGAGTGCAGCACTTTACGCTAAAAGAGCTGGGGTTTTACTGAAACAAAAGAAACCTGTTGCTGCAATACGGGACTGT---------------GACAAAGCCTTGCAGCTAAATCCAGACCAGGCCTTGGCCTTTAAATTTCGAGGAAGAGCACATAGCTTGCTGGGAAAATGGGAAGAAGCTGCTTTAGATTTGGCAAATGCTCAGAAAATTGACTATTCTGATGATGCTAATGAATGGCTGAAAGAAGTTATGCCCAAT [717]

Ortho AGTACTGGTAACATGAGTGCAGCTCAGCTCCATCAGCTTGAAGAATTCGTCAAACTTTGCAAAGAACATCCAAATTTAATGCATATGCCGCAACTGAAATTCTTCAAGGATTTTCTTGAGAGCTTTGGAGCATCTATTCCAGTTATCCCAACCGAACCGCCCAGTCAAGATCCGGAATCACCAAAGCCTAATACATCCTCACAATCAGAAACGAAAGGACCTCAG---TCACCATCATCAGGATCAGAAGATATTGAAACTGATGAAAGCAGTGAAGAAAGTGAATTAGAATTGGACACTACCCTTGTAATTGAACCAGAT---TCTGACAAACCTTATCCTATGGGAGATGAAACTCTAGAGGTAACTGAAGAGATGATAGATGAAAGCTCTGAGAAGCGAGGCCAAGCTCAAGAGGCCCTTTCTGAAGGCAAACTCGATGAAGCCATAACCCTATTCACAGAAGCTATACTGAAAAACCCCAGCAGTGCAGCAATGTATGCAAAAAGAGCAGGTGTCCTTTTAAAGCAAAAGAAGCCAGTTGCTGCAATAAAGGATTGT---------------AACAAAGCCTTGGAACTGAATCCGGATCAAGCCTTAGCATTCAAATTTCGTGGAAGGGCAAATAGCTTTCTTGGTAAATGGGAAGAGGCGTCGTTGGATTTGGCTAATGCTCAGAAAATTGACTACTCAGATGACGCCAATGAGTGGTTAAAAGAAGTCATGCCCAAT [723]

Hespero AAACCGTTTGCCATGAATAACAATCAGCTTTTCCAACTCCAAGAGTTCGTTAAATTGTGCAAAAAGAATCCTGGCATTGTTCATATGCCGGAATTAAAATTCTTCAAGGAATATTTGGAGAACTTAGGAGCTACTCTTCCCGATCGT------CCGCCCCAAGATGAGAAAATTCCATCTCCTAAACCTAGCGGTTCT---------AAAAACAAAAACGAAGAT---CACGATATGACCGATGATATA---GACAGTGATATCGAAAGCAGTGAAGAAAGTGAACCTGATATTGATAATTCAAATGTAATAGAACCCGAT---ACGGATGATCCTCAAGCTATGGGAGATGACACTTTAGAGGTCACTGAAGAAATGATGGACGAAAGTTCGGAGAAAAGAGGTCAAGCCCAAGAAGCTTTTTCCGAAGGGAAAATTGATGAAGCTATTCAACTATACACTGAGGCCATTCAAAAAAATCCACAGAGTGCAGCGTTATATGCTAAAAGAGCCGGAATTTTTATCAAGCAACAGAAACCAATGGCTGCCATTAGGGATTGC---------------AATAAAGCTTTAGAATTGAATCCCGATCAAGCTGTTGCTTTCAAATTTCGTGGCAGAGCTAATAGCCTAATTGGAAAATGGGAGCAAGCGGCATTGGACTTGGCTAATTCTCAGAAAATCGATTATTCCGACGACGCCAACGAATGGTTGAAAGAAGCTATGCCTAAT [705]

Leiobunum ACTATGTCGGGATTAAACAAAGCTCAATTGGAGCAACTTGAATCATTCATCAAGGTTTGCAAAGAAAATCCAGCAATTTTGCATATTCCTGAACTGAAATTTTTCAAAAATTATCTCACTGGTTTGGGTGCTAGGATACCAATTCCA------AATTTTAAAGTACCCGAGGATGAA------AAACAAGAGTCTTCA---------AAGATGGAACATGGAATG---GAAGATGAACCAGAGGTGGTT---GAGGAGGTAGCAGAAAGTAGTGAGGAGAGTGAAGTTGAATTAGACAATACTGGTGTAATAGAAGCTGATGAAATTAGCGAACCACTTCCTTTCGGAGATGATTCTGTCGAGGTAACTGAAGAAATGATGGATGAATCAGCTGAAAAACGTGCTCAAGGACAAGAAGCTATAAGTGAAGGAAAACTTGAGGAAGCCATAGAATTATTTACCCAAGCAATTCAGAAAAATCCTCAAAGTGCTCCGTTATATGCTAAAAGGGCTGGAGTGCTCATTAAACTGCAAAAACCAGTAGCTGCTATTAGAGATTGT---------------GACAAAGCACTTACCTTGAACCCAGATCAAGCATTGGCATATAAATTTCGTGGAAGGGCAAACCAATTGCTAGGAAAATTTGAAGAAGCTTCTTTAGACTTGGCGAATTCTCTTAAAATTGATTACAGTGATGATGCCAATGACTGGCTTAAAGAAGTTACGCCAAAT [702]

Proto ACTATGTCAGGATTAAACAAAGCTCAACTGGAGCAACTTGAAACATTCATTAGTGTTTGCAAGGAAAATCCAGCAATTTTGCATATTCCTGAATTGAAATTCTTCAAAAGTTATCTCATTAGTTTGGGTGCTAGGATACCAGCCCCA------ACGTTTAAAGTACCAGAGGATGAACTTCCAAAAGAAGAGCCACCA---------AAGAATGAACCTAAAATGGAAGAAGATGAACCAGAGGTTGTT---GAGGAGGTGGCAGATAGCAGTGAGGAAAGTGAAATTGAATTAGATAATTCTGGTACAATAGAAGCTGATGAAATTGGGGAACCACTTCCATTTGGAGATGATTCTATTGAGGTAACTGAAGAAATGATGGATGAATCAGCAGAAAAACGTGCGCAAGGACAGGAAGCCATGAGTGAAGGAAAACTTGATGAAGCGATAGATTTATTTACTCAGGCTATTCAGAAGAACCCTCAAAGTGCTCCATTATATGCTAAGAGGGCTGGGGTACTAATTAAACAACAAAAGCCAGTGGCTGCTATTAGAGATTGT---------------GACAAAGCTCTTACCTTGAACCCAGATCAAGCATTGGCATACAAATTTCGTGGAAGGGCAAACCAATTACTAGGAAAATTTGAAGAAGCTGCTTTAGACTTGGCAAATTCTCTTAAAATTGACTACAGTGATGATGCCAATGACTGGCTCAAAGAAGTTACACCTAAT [711]

[TICKFAST_NCHAR=1212;histone acetyltransferase type B catalytic subunit]

Ixodes ATGTCGAAAGGGGTCCTCGACGCCTATGTTTTGTCCGGAAATGAAGTGGTGGAATTCAAACTAGTTTTGAACGAAGACGACCTGGAAAACGACGACATAGCGTTTCCTCCTGAAATGTCCCACCAGATCTTCGGCGAAGGAGAAAGCATCTTTGGTTATCGAGACCTAAAAGTGCAGCTCTACTATGCAGCATGCTCCCTCACCACCTACCTTCACATGTCGTACACCGAAAAGATCACCTTAAAAAAGAGTGATGGTGTCAAGGCGGATAACGTTTTGAAGATGATTGGTGAGAAGCTGCCCCCTGGTGTGCTCTCCAACCTGGATGACTTTGTGGCAACACTCCCCAAGGAGCCGTCCTTCCGACCTTTCGGGACCCACCTGCACTCATTC------ACCGTCAACAAAGGGACAGCCTCAGCACAGACTTTTAAGCTGTACAAGGCTGACATCACC---GTTCCCGGTTTCGTCGACTATCATGCCAGGATGGAGAGCTTCATTCTGTGGTTTATCGATGCTGCCTCTTACATAGACTCGGACGATGAGAAGTGGGAGTACTTTGTCCTGCACGAAAAGAAGCTTGTCAATGGCAAGGTGTGCTACCCCTTCGCCGGTTATGCCACCGTCTACAGATACTATGCCTATCCCAGCAACATCCGGCCCAGGATAAGCCAAATGTTTATTCTTCCACCATTCCAAAAGAAAGGGCTGGGAGCAGAAATGCTCCAGGGCATCTACAACTACTACACAGGATGCAGGGATGTCATTGACATCACCGTGGAAGATCCGTCCACCACATTCATCCGGCTTCGAGACTTTGTGGACTCCAAAAACTGCCTGACCCTGCCCTCTTACAGCAGGCAACATCTACACAAGGGCTTCTCCGAAGAGATGAGGAAAGAGGCCCAGGAAAAGTTCAAGCTGAATAAGAAACAAGCACGAAGAGTGTATGAGATTCTCCGTCTGCGCGTCACCAACACAGCGAACCCCGGCCAGTACCAGAAATACAGGCTGGAAGTGAAGAACCGGCTCAATGCACCATACCAGGCAAGTAAGGCGGACGTGGAGAAGCTGCGACGGACCATGTCGCCGGAAGAGTTTGAGGCAACGATGCAGTGCCTGAACGCTCAGAACCGGATCGAGCAACTCGAGAGCCAGTACCGGGAGCTGGAGCTGGAGTACCGGCACACACTGGAGCGTCTGGCG [1203]

Siro CTAGCTGGCGGCGTCCTCGACGCTTTTGTGACTTTTGCCAACACTGCAGTGAAAATGAAATTAGTTAGCAAAGACGACGACGTACAAAATGATGCAATTGCCTTCTCACCAGAGTTTTCGCATCAGCTTTTTGGCGACAGTGAAAGTATTTTTGGTTATCGTGATCTCAAAATTCAACTCTACTACACGGCGTCCCGACTATCGACGTATTTGGGAATAACTTATGCTGAAAAAATAACTGCGGAAAAAAGTGGAGGCGTCCAGCCAGACAATGTTCTACAAATTATTGGTGAAAAACTTCAATGTGGTTATACGTCAAATCTCGATGATTTCGTCGCATCTCTAAAAACGGAAAAAGGTTTTCTTCCGATGGGCGAAAAACTGCACAGCTTT------AAGATAGAAGACAGTTCTAACGCAAAACGTTGCTACGAGATTTACAAGTGCCACGCCGAT---TGGCCAGGATTTAAGGAATATCACGCACGAATGCAAACGTTTATTCTTTGGTTTATCGATGCCGCTTCTTATATTGACACGGATGACGATCGTTGGGAATTTTTTATTCTATTTGAGAAGACTTTGACAAACGGAACGCAACTTCACAAGTTTGCAGGATACGCAACTGTGTACAGATATTACGCTTACCCTACAAAAACCCGGCCAAGAATTAGCCAAATGCTTATTCTCCCACCTTATCAAAAGCAAGGCTTAGGCGCTGAACTACTACAGACCGTTTATAATTGGTTCGTTCCTAATACAGATGTAGTTGATATTACAGTCGAAGATCCGTCAGAAATGTTTACCAGATTACGCGACTATGTCGATGCCAAAAACTGCCTCAAACTACCGAGCTTCAAAGCTCCGGGAATTGAAAAAGGGTTTAGCGAAGATATGCGTTTCGAAGCTCAAGCGAAATTCAAACTCAACAAAAAACAGGCCAGACGAGTTTACGAAATCCTAAGGTTTAAAGCTACAGATAAAACGGACAGAGAAGCTTATAGAAATTATCGATTAGACGTTAAAAGGCGGATCAATGCTCCGTATCAGCGGCAGCAATGTGATTTGGCCAAACTCAAGCACACTCTAAGCCCAGACGAGTTTCGTGCGACCCTTCAGTGCATGAATCGCGAGCAAAGAATCGAACAGCTGGAAAGCCAGTATCAAGATCTGGAATCGGAATACAGCCGAATTGCCGATAGATTGGAT [1203]

Sitalc TTATCTAAAGGACACTTGGAAGAGTATGTTTGTCCGGCAGAACAAGCTGTGGCACTCAAACTGGTTACTAACGAAAACGACATCAAAGACTCCGGTAAAATCTTCCATCCAGAATTCTGTCATCAGATCTTCGGAGATGGAGAAAGCATATTTGGGTACAAAAACCTTCAGGTGAAGCTGTACTTCTCAGCAGGCAGGCTAACTACTTACCTTGGAATCAAATATACAGATAAGATTCCTCCAACAAAAAGTGAAGGTGTAAAGCCCGATGATATAGTAAAAATCCTGGGGAAATACTTGCAGCCTGGCTTCCTGACTAACATCAACGACTTCACATCTGCTCTCTCCAAAGATCAGAAATTTGAACCCTTCGGAGAAAAACTTCACAGTTTC------TCGCTTCCAAATAAAGAAGAC---AAACAAACGTTTGAAGTGTACAGATGTGATATCACA---ACCAATGGTTTCCGTGAGTACCACGAACGAATGCAGATATTCGTGCTCTGGTTTATAGATGCAGCATCTTATATAGACGCAGATGACGATAAGTGGGACTTCTTTGTTCTGTACGAACGACGAGTGGTCGATGGAAAAGCCAAGTATTTCTTTTGTGGTTATACAACAGTATACCAGTACTACGCATTTCCCGTTAAAACACGACCAAGGATCAGCCAAATGCTGGTTCTCCCCCCGTACCAGAGGAAAGGATTAGGAGGTGAACTCTTACAAGTCATTTATAAGTGGTACATCAAAGAACCTGATGTACTGGACATCACAGTGGAAGATGCCTCTGAAGCATTCACCAAGTTGCGAGACATTGTGGATGCACAGAACTGCTCCAAACTGGATTCGTTTCAGAAGGATAAACTCAAAAGTGGCTTCTCCAAAGAAATGTGTCTAGAAGCTCAGAGTAAACTCAAGATCAACAAGAAACAAGCTCGTAAGGTTTACGAAATTTTGAAATTGAAGGCAACCAACTTCAAAAATTATGATGAATATCGTGGCTACAGGTTGGAAGTGAAGAAAAGGCTAAATGCACCTTATCAGAAACAAAAAGAAGATATGAAGAAGTTAAAGAAAACTTTGTCAGCAGAAGAATATGAAGCAACCATGCAGTGTTTCAGCCGAGAACAGAGAATTGAACAGTTGGAAAAACAGTATCGTGAGCTTGAAGACGAATACAGGAGTATTCTGGAAAAACTGGCC [1200]

Sclero TTATCAAAAGGAAATCTTGAAGCGTATGTCTGCCCTGCAGAGGAAGCCGTTGAAATCAAACTGGTTTCAAAGGAGTCGGACATAAAAGATAACAGTTTGATTTTTCACCCAGAATTCTGCCACCAAATATTTGGCGATGGGGAAAACATATTTGGTTACAAAAATCTCAAAGTTCGCCTTTATTACACAGCAGCAAGACTTACAACCTACCTTGGAATGAAATATACTGATAAAATTTCTCCAAGTAAAAGCGAAGGAGTGAAGCCTGACGATGTGATGCAAATAGTCGGCGAACAATTACAACCTGGATTTTTGACTAACCTGAACACGTTCACTTCTGCTTTGTCCAAAGAATCGAGCTTTAAACCTTTCGGGGAAAAGCTTCACAGCTTTACT---GCAAATGATCAAGAAGTTTCTTCCAAAGATTGTTACGAGGTCTACAAATGTGATATCACC---ACTGAAGGGTTTGTCAACTACCACGAGCGAATGCAAACGTTTGTCCTTTGGTTCATCGATGCAGCCTCCTATATCGATTCCGATGATGACCGTTGGGAGTATTTTGTCTTATTCCGAAAATATACTTTGGACGACAAAACCATGTACGCCTTCGTTGGATATACAACTGTATACCAATATTACGCGTACCCAAGCAAAACAAGACCTAGAATCAGCCAAATGATAATTTTGCCACCTTACCAGAAGAACGGCTTAGGAGCGGAACTATTAGAAGTGGTTTACAGGTGGTACATTAAAGAGTCTGACGTACTCGACATCACAGTTGAAGATGCCTCTGAAGCGTTCACGAGAATAAGGGATTTTGTGGACGTTAAAAACTGCATGAAACTTGCCTCCTTTCATAAAGACAAGTTAAAGCTCGGCTTCTGTGAAGCTATGCATCAGGAGGCCCAGAACAAACTTAAAGTCAATAAGAAACAAGCTCGCAAAATATACGAAATATTGAGGTTAAAAGCCACGGATCTGAAAAACAAGCAAGCCTATCGAGAGTACAGACTGGACGTGAAAAAGCGATTAAATGCTCCTTTTCAGAAACAAATAGGTGATATGAAGAAGTTGAAGTCTATACTCTCCGATGAAGAATACGAAGCAGCGGTACAGTGCAACAGTCGAGACCAAAGGATTGAACAGCTCGAGAGTCGATATCGTGAACTGGAAGACGAGTACCGGAACATCATCGAGAAGCTAGCT [1206]

Trogulus CTATCTAGAGGTATTCTTGATGCTTATGTGTGCTCAGCAAATGCAGCTATTGAAGTTAAACTAGTAACTGAAGAGGCTGATATTGAAGACGAGGAGATTGCATTTTATCCAGAATATACGCACCAGTTTTTTGGCGATGGAGAAAGCGTATTTGGATATCGTGACCTTAGAATTCAGCTGTATTATTCCTCATGTCGCCTTACGACATATCTGGGTGTATCTTATACAGACAAAATAACCAAAGAAAGGAGTGATGGAGTTCTTCCTGATGATATTTTCAAGATAATTGGAGAAAAGCTGCAGCCAGGATTTGTAACTAATTTGGATTTATTTACATCAAGTTTAAAGAAGGAAGTAACCTTTAAACCTTTTGGAGAGAAGCTTCATAGCACT------TCAATTAATGATGAAGATAATCAGAAAAGAACCTTTGAGGTTTACAAGTGTGATACCAGT---TGCCCAGGATTTCGTGACTACCATCAGAGAATGCAGACTTTTGTTCTATGGTTTATTGATGCTGCCTCTTATATTGAAATTGATGATGACAGATGGAACTTTTTTATTATGTATGAAAAGTTTTTATTAAATGGAAAGTCCTTGTATGCATTGGTAGGATTTACAACAGTATACCGCTACTATGCTTATCCAGAGAAAGTCAGACCACGAATTAGCCAAATGTTAATTCTTCCACCATTTCAAAAGCGAGGTTTGGGTGCAGAACTTTTACAGACAATTTATAATTGGTACGTCAAGGATTCTACAGCTATAGATATAACCGTTGAAGACCCATCAGAGAGTTTCACAAGCTTACGCGATTATGTGGATTCAAAGAATTGCATGAAACTTGTGAGCTTTCACCCAACAAAACTTTGTAAAGGATTTTCCCAAGAAATGAAACAGGAAGCTCAAGCTAAACTTAAACTCAATAAGAAACAAGCTAGAAGAATTTATGAAATTCTGAGACTCCAATCAACAGATACCAAAAATTGTGAAGCTTACAAATCATATCGTTTAGATGTCAAAAACAGGCTCAATGCACCCTATCAGCGCCAAAAAGAGGATATGCTGAAGATTCAGTGTAGCCTTTCTCGGGATGAACTGAAAACAATGTTGCAGTGCTCAAGCAGAGAACAGCGCATTGAACAGTTAGAGAAACAATATCGAGATCTTGAAGATGAATATAGACATGTTCTTGAGAGGCTGGCG [1203]

Ortho TTATCCAGAGGCATTCTCGATGCTTACGTCTGTTCAGCTAACGCTGCTGTTGAAATTAAACTTGTGACAGAAGAAACTGATATTGATGACGACGATCTCACATTTTCTCCTGAATACACACACCAGTTCTTTGGAGATGGTGAAAGCATTTTTGGCTACCGAGACCTTCGAATACAACTCTTTTACTCTGCTTGTCGTCTGACAACTTATTTGGGCGTCTCTTATACAGATAAGATCACAAAAGATCGAAGTGACGGAGTTCTGGCTGATGATATATTCAAGATAATCGGAGAAAAACTGCAACCTGGTTACTTGACCAATTTGGATTTGTTCACCACAAGCTTAAAAAAAGAGGCAGCGTTTAAACCGTTCGGAGAAAAGTTGCACGGTTTA------ACAATCAACGATGAAGATAACCAAAGAAGAAATTTTGAAATCTACAAATGTGATACTACCTCGAGTACCGGATTTCGTAATTACCACGAACGTCTACAGACATTTGTTCTTTGGTTTATCGATGCCGCTTCCTACATCGATATCGACGATGACCGATGGAATTTTTTTGTTATGTATGAGAAGTTTTTATTGAATGGCAAGCCTTTGTATGCGTTGGTCGGTTTCACAACGGTCTATCGCTACTATGCCTATCCAGAAAAAGTTAGGCCTAGGATCAGTCAAATGCTGATACTACCGCCATTTCAAAAGAGGGGACTAGGTGCTGAGCTATTGCAGACGATCTATAATTGGTACATTAAAGATTCGTCAACTGTGGACATAACAGTCGAAGATCCTTCGGATAGTTTCACAAGTTTGCGTGATTTTGTGGATGCCAAGAATTGCATGAAACTGCTCAGCTTTCATCCTTCCAAACTCTGCAAGGGATTTTCTGCAGACATGAAACAAGAAGCTCAATCCAAACTTAAACTAAACAAAAAACAAGCCAGAAGAGTTTATGAAATTTTGCGTCTTCATTCGACGGACACCAAAAACTGCGAGGCTTACAGATCCTATCGTCTAGATGTCAAAAACAGGCTGAATGCACCCTATCAGCGCCAGAAAGAAGACATGATGAAAATCCAGCACTCGCTATCACGCGACGAAGTGAAAACAATGCTACAGTGTTCAAGCCGTGACCAACGCATTGAACAACTAGAGAAACAATACCGAGATCTAGAAGAAGAGTACCGCCATGTTCTCGAAAGGTTGGCT [1206]

Hespero TTGTCTCGAGGTCTTCTCGACTCGTACGTTTGTTCCGCCAATTCGGTGATCGAAATGAAATTAGTGGCCGAAGATGGCGACATCGACGATGAAGATTTGAAGTTTTATCCTGAATTTACCCATCAGTTCTTTGGAGATGGAGAAAGCATATTTGGTTATCGCGACTTGCGTATCTTGCTGTATTACTCTGCGTGTCGTCTGAGCACATATTTGGGTGTACGCTACACGGATAAGATAACGAAAGAACGTAGCGACGGTGTTCAAGCTGACGATATAATGAAGATAATTGGAGAGAAATTGCAGCCCGGTTACGTTACCAGTTTGGACCAGTTTGTCACTGCCTTGAAGAAAGAACATTGCTTTAAACCATTCGGAGAGAAATGGCATTGCCTG------AGATTAAACGATGTCGAGAACACAAAAAGGACATTTGAGGTTTACCGTTGCGACATTGGT---TGTCCCGGATTTAAAGATTTCCACGAACGGATGCAGAATTTTGTGCTGTGGTTCATCGACGCAGCCTCCTATATTGACGTAGACGACGAAAGATGGGATTTCTTTACTATGTATGAGAAATTCACCTCCAATGGCAAAACAATGTACGCCGTTGTTGGTTTCACAACCGTCTATCGTTACTACGCTTACCCTGCGAAGATGCGTCCTCGTATAAGCCAAATGTTGGTGTTACCCCCCTTTCAGAAGAGGGGGTTGGGTGCAGAATTGCTGCAGGCCATTTACAATTGGTACATCAAAGAGCCCGACACTGTGGACGTTACGGTCGAAGACCCGTCCGAGAATTTTGTCAATTTGAGAGATTTTGTGGACGCGAAGAATTGCATGAAATTGGTCAGTTTTCATCCTTCCAAATTGCCCACCGGATTCAGCCCGGAAATGAAGCAAGAGGCACAAGCCAAGCTCAAGATTAACAAGAAACAAGCGAGGAGAATATACGAGATACTCAGATTACACGCTACGGACACGAAGAACGAAGACGCGTACAGAGCGTACAGGCTGGACGTTAAAAATAGATTAAATGCTCCTTATCAGAGACAAAAAGGCGACATTCAAAAACTCCAATTAAGTTTATCGGCTGACGAATTAAAAGCAACTATGCAATGCACAAATCGAGAACTTCGAATGGAACAATTGGAAAAACAATATCGAGATTTGGAAGACGAATATCGACATACTCTCGAAAGACTGGCA [1203]

Leiobunum TTGTCACGGGGCAGTCTAGATGCCTATGTTTGTTCCGCTAATTCTGCAATTACTATGAAATTAGTATCTCACGAGGAAGATTTACATGAT---GATATTGAATTTTATCCCGAATTTACTCATCAATTCTTTGGTGATGGGGAGAGCATATTTGGATATAGAAATCTTAAAATAATGCTGTATTACACTGCGGCTCAGTTAAATACGTACCTTTCAGTTTCTTACACTGACAAAATAACTGAAGAAAAAAGTGGCGGTGTTAAACCTGATGATATAATGAAAATTATCGGGGAAAAATATTTAGCAGGATTTGATACGAACATCGACACTTTTCTCTCCTCATTGACAAAAGAGAGTACCTTTCGACCATTTGGAGAAAAACTTCATAGCGTAGATGTAGCCCTCAAAGAAGAAGGAGATTTAAAAAGAACTTTCGAAATATACAAATGTGACACAAGT---TGTCCAAATTTTACCAAATACCACGAACGTATGCAAACCTTTATTATGTGGTTTATTGACGCTGCTTCGTACATTGATGTTGACGATGACAGATGGGATTTCTTTATTGTTTATGAGAAATATGAAGAGGATGGTTGTTTGAGATATGCTTTTGTGGGCTACACTACTGTCTATCGTTACTACGCTTATCCAGCTAAACAAAGACCTAGAATAAGTCAAATGTTAATTCTACCTCCTTTCCAAAAACGTGGTTTGGGAGCGGAATTGCTACAAACAATTTACAAGTGGTATATTAAGGATTCAAACACGATTGATATTACAGTGGAAGATCCATCTGAGAATTTTTCGTATTTAAGAGACTTCGTTGATGCTGAAAACTGCTCAAAATTATCTTCATTTCAGCCGTCCAAATTGATTAAAGGTTTTAGCGAAGAAATGAAGAAAGAAGCTTTGTTAAAGCTTAAAATGAATAAAAAACAAGCTAGAAGAGTTTATGAAATTTTGCGGCTCAAAGCGACTGATACCAGAAACAAACAAGCGTACAAAGCGTATCGTCTTGACGTTAAAAATCGGTTAAATGCACCCTATCAAAGGCAGAAAAATGATTTGGCCAAGCTGGAACACACATTAAGCCCGGAAGAATTTAACAAGATGATGCAAAATGAAGGCCGCGAGCAGCGTTTTGAGCAACTAGAGAAGCAGTATCGGGATTTGGAAGATGAGTATAGACACACTTTGGAAAGATTAGCG [1206]

Proto TTGTCACGGGGTAGCCTAGATGCCTACGTTTGCTCTGCTAATTCTGCGATTACGATTAAATTAGTATCTCACGAAGAAGATTTGCACGAT---GATATTGAATTTTATCCCGAATTTACACATCAGTTCTTTGGTGATGGGGAAAGCATATTTGGCTATAGAAATCTTAAAATAATGCTGTATTACTCCGCGGCTCAGTTAAATCCATACCTTTCAGTCTCTTACACTGACAAAATAACTGAGGAAAAAAGTGGAGGTGTAAAACCTGATGATATTATGAAAATTATTGGAGAAAAGTATTTAGGGGCTTTTGAAACAAACATAGACACGTTTGTTTCTTCATTGAAAAAAGAATCTAGCTTCCGACCTTTTGGTGAAAAACTTCATAGTGTTGATGTAGCTCTCAAAGAAGAGGGGGATTTAAAACGAACTTTTGATATATACAAGTGTGACATGAGT---TGTCCCTATTTTCGAAAATACCACGAGCGGATGCAAACATTTATTATGTGGTTTATTGACGCTGCCTCTTATATAGATGTTGACGATGATAGATGGGATTTCTTTGTTATTTACGAGAAATACGAAGAAGATGGCTGTCTGATGCATGCGTTTGTTGGTTATACAACCGTGTATCGTTACTACGCATATCCGGCCAAACAGAGACCCAGAATCAGCCAGATGTTGATATTACCTCCTTTTCAAAAACGCGGTTTGGGAGCTGAATTGTTACAAACTATTTATAAATGGTATATTAAAGATTCGGATACGATTGATATTACAGTTGAAGACCCGTCTGATAATTTTGCAAATTTGAGAGACTTTGTTGATGCTGAGAACTGCTCAAAGCTACCTTCATTTCATCCGTCCAAATTGAAGAAAGGTTTCAGTGAAGAAATGAAGCAAGAAGCCTTGTTAAAGCTTAAAATTAATAAAAAACAAGCCCGTAGAGTTTATGAAATTTTACGACTTAAAGCAACTGATACAACAAATAAAGTTGCCTACAAAGCCTATCGTCTTGATGTGAAAAATCGTTTAAATGCGCCCTATCAAAGGCAAAAAAATGATTTGGCGAAGCTGGAACACACACTAAGTGTCGAAGAATTTACCAAAATGATGCAAAATGAAGGGCGTGAGCAGCGTTATGAACAACTAGAGAAGCAGTATCGGGATCTGGAAGAAGAATATAGACATACTCTAGAAAGATTAGCG [1206]

[TICKFAST_NCHAR=561;hypothetical protein XM_002400131]

Ixodes AAAAAGCTGAAGCTGAAGTTGGAGAGCTGGCGGGAGGTTGTCCTTCCTCTAAACTCTGTCCTCGTCTGGAACCAGCCGTACTACCCGGGATGCGTCTTTGGCGTCGTCTCCACCCTCTTTCTGCTGCTGTGGTACATGGAGCCCTCCCTGCTGACCACCCTTTCCCTGGCTGGCATGGTGGCCTGTCTGGCGGACTACCTGGTGCCCCTGGCCTGCTCACACCTCTGCAGCCAGGACAAGTGGACGAGCCAGCACGAGCGTCAGCTTCAGGACATCTGCCTCGAGGTGGTACACGCGAAGCATTCTCTCATGGGCTTCTGCACTGCCATCAAAACAATGAAGCAGGAGAAGCCCAAGATGTACATCATTGTGGTGCTGGTGTCCCTTCTGCTCGTGGCCTGGATTGGGAATATCTTTGACAACCTGTTCCTCACATACCTCATCGTTCTCTTTGTGGCCATGGTGCCCGGCATGAAGCGCCACGGGATCTTCAAGAAGTACTTCTCGGTTGTGGTCCTCTCGATCCGGAATCTCATCTCCTCCAAGATGAAGAAGAACTGA [561]

Siro ????????????????????????????????????????????????????????????????????????????????????????????????????????????????????????????????????????????????????????????AGCCTTTCAATAATTGCCATCATTTTTTCGCTTCTGGACTACCTAGTCCCTCTGCTCAGCGCCAGCTTTTTTGATCCCAACCAATGGTCGGGGCCACAGGAGCAACGGTTCGAAGAGATCTGCAGCGAGGTTGCCCATTGTAAGCAGTCCATTGTGGGCGTGTGGCATTCGGCAGCGCAACTGAAAGAGTCCAAACCCAAAGTGTACTTTGCCGTGACTCTGGCATCTCTGGTTCTCTTCGCTTGGATCGGCAACGTTTTCGACAACCTCTTTCTCACCTACCTAACAGTGCTTTTTGTGGTGATGACTCCCGGAATGAAACAAAACGGAACGGTTCAGAAGTACGTGGCAAAGGGAGCGCTGGCCCTTAAGGAGAAGTTGGGC---AAAGCCAAGAAGAACTAA [558]

Sitalc AAGCACTTGACGCGTGTACTTGAGGGATGGCGAGAGATCCTCCTTCCTCTGCACTCAGTCCTGGTCTGGGAGCAGCCTTACTTTCCTGGAGTCATAGCTGCTGTCGTCAGTTTCTTGTTTTTAATAATCTGGTACTGTGATCTGTCTGTCCTGACAACCTTGTCCCTGGTGGGCCTCCTGGCATCCATCTTGGATTATGCGGTCCCCCTTCTGAGTACCAATATTTTCGACCCTGCAAAGTGGACTGGCAGTCAGCAACGTCAGTACGAGGACATCTGTCAGAATCTGGTTCAGTCGAAACATTGCCTTTCTAAGTTTGGCTCAACACTCAAGAACCTCAAGGACAGCAAGCCAAAAGTGTACTTTGTGGTTGTCCTGGTGATGCTGATTTTACTGGCTTGGATCGGGAACGCAATTGACAACTTGTTTCTCACTTACCTTATCGTGTTGGGTCTGGCCATGGTACCTGGTATGCAGCGAAACGGCACTATCAAGAAACTGGTTCTACAAGCTATCCAGATGGTACAGAGCAAATTTGGAGCCAAAGAGAAAAAGAACTAA [561]

Sclero AAGAAAGTGATGCGAAAACTGGAAGGCTGGCGAGAAATTCTCATTCCGGTCCATTCCGTTCTGGTCTGGGAAAAGCCTTTTTTTCCGGGAGTCATCATCGCCCTCAACACATTTATTTTCATAACGATTTGGTACTTTGATTTGTCTGTGCTGACCACGTTGTCTATTGTGGGCCTGCTGGCCACGGTTCTAGATTTCTCGGTTCCCCTGATCAGCACCAGCCTGTTCGACCCAGCAAAATGGACGGGCGTCCAGCAACGTCAGTACGAAGAAATCTGTCAGAATCTGGTTCAGTCCAAGCATTGCTTCGGCCAATGTGTCTCTACTTTCAAGAACCTCAAAGAGAGCAAACCTAAAGTGTATTTTGTGGTTGCTCTCGTCAGCTTGATTCTGTTGGCCTGGATTGGAAACACCATAGACAACTTGTTCCTCACGTATAACATTGTGTTGATGTTACTAATGTTGCCAGGCATGAAGCAAAACGGAACAGTACAGAAGATGCTTGGAAAAGCTGCCCTTGCAATCAAAAACAAGCTCGGG---AAGGAGAAGAAAACCTAA [558]

Trogulus AAGCAATTGAAAAGAAAGCTGGAAGGATGGCGGGAAGTTTTGCTCCCAATCCACTCTGTTCTGGTCTGGGAAAAACCCTTTTTCCCCGGGATCATTGCTGGAGTTGTCACTGCAGTTTTTCTAATGATTTGGTACCTCGATCTTTCTGTTCTGACTACTCTTTCCATCATGGGACTTATTGGTTCGCTATTGGACTACGCTATTCCACTTCTCAGTTCTAATCTTTTCGACCCCAACCAGTGGAATGGAGCTCAAGAAAAGGAGTTTGAGTCAATCTGTAAGAACACGGTCCTGGCCAAGAATTGCCTCTGCGATACCTGGAATTCGCTTGGAAAGATGAAGGAGAGCAAACCCAAAGTGTACTTTGTTGTGGCTCTCAGCACCTTGGTTCTGATGGCGTGGATTGGGAATGTCTTTGATAACCTTTTCCTGACATACCTGATTGTTTTGTTTATCGCGATGGTTCCTGGGATGAAGCGCAATGGAACGGTCAAGAAACTGGTCACTCAGGCTGTCATGATTATCAAAGATCGAATGGGA---AAATCTAAACTCAATTAA [558]

Ortho AAGCAGCTAAAGAGAAAACTAGAAGGATGGAGAGAAGTCTTGCTCCCAATTCACTCTGTTCTCGTTTGGGAAAAGCCATTTTTCCCCGGCATCATTGCAGGAGTTGTTACAGTTGTTTTCCTTATGATCTGGTATCTTGATCTGTCAGTTCTAACCACCCTCTCAATCATCGGCTTCATTGGTTCACTTTTGGACTACACTATCCCTTTAATCGGTGCCAGCCTCTTTGATCCCAACCAATGGAATGGAACCCAGGAGAAGGAGTTTGAAACCATCTGCAAGAACCTAGTTTACTCAAAGTATTGTGTCTGTGATGCCTGGAACTCTTTGGGAAAGATGAAAGAGAGCAAACCAAAAGTGTATTTCGTTGTTGCGCTGAGTACACTTGTTCTTTTGGCCTGGATTGGGAATGTTTTTGACAACCTGTTCCTCACATACCTGTTAGTGTTGTTCTTGATCATGCTCCCTGGCATGAAACGAAACGGGACGGTCAAGAAGCTTGTAACTCAAGCCGTTCTGATCATCAAAGACCGTTTGGGCAAAACTACTAAGGTCAATTGA [561]

Hespero AAGCACTTGAAACGCAAGCTAGAAGGATGGAGGGAAGTTATATTGCCCCTGCATTCTGTCATGGTGTGGGATAAACCATTCTATCCTGCCGTTATTAGTGGAATTACTACCGTCGTTTTCATGTTGATTTGGTATCTGGATTTGTCCGCATTGACCACACTGTCTGTGATTGGTTTATTGATCTGTCTGCTCGATTATACCGTCCCTTTGGTCAGTTCGAGTTTCTTCGATCCCGAGAAGTGGAATGGCACACAGGAGAAGCAATTCGAGGGTATTTGCAAGAGTTTGGTTGCGACCAAATATTGCGCTATCGACGCATGGAAGTCTATGGCACAGATGAAAGCCAATAAGCCCAAAGTGTATTTCGTGGTTGTACTGAGCACTTTGGTTCTATTGGCTTGGATCGGCAATGTTTTCGACAATTTGTTTCTCACTTATCTCATCTCCGTTTTTGCGCTGCTTTTGCCCGGCATGAAACGCAACGACACCGTCAAGAAGTTCGTCACTCAGGCCGTTCTCATTATCAAAGAGAAAATGGGAAAAACTAAGAAAATTGATTAA [561]

Leiobunum AAACACCTTAAACGTAAACTTGAAGGATGGAGAGAAATTCTACTCCCTATTCATTCTGTATTAGTTTGGCAAAAACCTTTTTATCCTGGAATCCTTGCTGGAGCTTCTACTTTAATTTTTTTATTTATTTGGTATTTGGATCTGTCTGTTTTAACAACTCTCTCATTTCTTGGATTCTTTTTCTCCTTACTTGATTATGCAATTCCTCTTATTAGCGCAAGCTTTTTTGATCCAAATCAATGGAATGGGATTCAAGAAAAACAATTTGAGCAAATTTGCCTTGATTTGGTAGAGGCAAAGCACTGTGTGTGTGATTCATGGAAAACCCTTGGTCATATGAAGGAGAGCAAACCAAAAGTGTATTTCGTCTCTGTATTATGTGGTTTGATCGCTTTAGCCTGGATTGGAAATATTTTTGATAACCTGTTTTTGACATACTTATTGGTTACTGGTTTAGTTCTTTTACCTGGAATGAAGGAAAATGGAACGGTCAAAAAGTTTGTTTCTCAAGCTGTTCTGATGGTGAAAGAGAAAATTGGA---AAATCAAAGACAAATTGA [558]

Proto AAACAACTTAAGAGAAAACTTGAAGGGTGGAGAGAAATATTGCTCCCCATACACTCTGTATTAGCCTGGCAAAAGCCTTTTTACCCTGGGGTCCTTGCTGGAGTTTCTACCTTAATTTTTCTATTTATTTGGTATTTGGATCTATCTGTCTTAACTACTCTTTCCATAATTGGACTTCTTTGTTGCCTTCTTGACTATACTATCCCCCTTATTAGTGCAAGCCTCTTTGATCCCAACCAATGGAATGGCACTCAAGAAAAGCAGTTTGAGGAAATTTGCCATGGGTTGGTGGAGGCAAAGCACTGCGTGTGTGATTCGTGGAAAACCCTTGGCCATGTGAAGGAGAGCAAACCCAAAGTGTACTTTGTCTCTGCATTGTGCACTTTGATCACATTAGCCTGGATTGGAAATGTTTTTGATAATCTTTTCTTGACGTATTTGTTGGTGACTGGCTTAGTTCTTTTACCTGGTATGAAGGAAAATGGAACAGTCAAGAAATTTGTCTCTCAGGCCGTTTTGATGATCAAAGACAAGATTGGA---AAATCAAAGACAAATTGA [558]

[TICKFAST_NCHAR=459;hypothetical protein XM_002400216]

Ixodes ATGGCGGATGCTTCGGCGCTTGCAGCCAGCGTGAAAGATATCGGCGAAGTGCATTCCAGACTGTTCGACCACAAGCCATTCCTTCAAGGAGAAATAAAATATTTCATCAAAGAATTCGAGGAAAAACGGAATGATCGTGAGGTACAGCGACTGTTCGAGATTCTGGAGAATGTCACCGAAATCAGGGAAACTCAAATTGATCGGATTTGCCGGAATTCCGACCAGAAACTCTGCAACCTGACGGGAAACCTGGAAGTTGCCCTCAGCATGTGTAACAAGATCCTAGAAGCAGAGGACAAAATCAACGTTGCCGAAGACTTGTCCGAAAGGAGAAAACAGCGACAGTGTGAGTGGGAGAAGTTTATGCAGGACATCAAGGACAAGACTGCAAGGATGGATGAGGCATTCCAGCAGAAGGAAAGGGAAGTCATAGACCATTTCAGGTGTCTCCAGGAGAAG [459]

Siro ATGTCTACTCCGGAA---------AACATTCTGAAAGATGTCGGCGAAATACATGGCCGTTTATTTGACCATCGCCCATTTCTACAAGGAGAAATTAATTTTTTCCTGAAAGAATTTGAGTGCAAGAGAAATGATCGAGAAGTTCAACAATTATTTGAAATTTTAGAAAACGTGACTGAAATTAAAGAGACTCAAACCGACAAAGCCTGTCGTCTTGGCGAGGCTCACCTCTGTGGGGTCACGTCAAATTTGGAAGTTGCGTTATCCATGTGCAACAAAATTCTTGAAAGAAGCACTCTAAACAATACCAATGAACTTTTGAGAACGAGAAGGGAAAAACGTGAACAAGAATGGCAAGAATTTGTCAGAGACAACCGAGATAAGTCCAATAAAGTGGACAAGGCTTTTGCAACGAGGGCCCAGGATTTGCGAGACCATTACGTCGAACTAGAAAACAAA [450]

Sitalc ATGGCTCCTGGTGCGAGT------GCAGTGATTAAAGATCTAGGTGAAATTCACGGAAGGCTTTTTGATCATCGGCCGTTTCTTCAAGGAGAAATATCACTGTTCGTAAAAGAATTTGAGGAGAAGCGTAACGATCGAGAAGTACAACAGCTGTTTGAGATTCTGGAGAACGTAACGGAAGTCAAGGAGACCCAAATAGAGAAGGCCTGTCGTTTGGGAGACGCCCAGCTCACAAATGTCACTACGAATTTGGAAGTGGCGCTCAGGATGTGCAACAAAATTGTGGAAAGAGAAAATCTAACTGATGTGGAGTCCAAGTTGTGTGAACGGAGAGCCCAGAGGGAGCAGGAATTTGAACAGTTCTCCAAAGATATGGTCGCCAAGAACATGTTGGTGGACAAGATGTTTGAAGAAAAGGAACAAGAAATGAAGGATTATTATGCTCAGTTAGAAGACGCT [453]

Sclero ATGGCACAACTTGAGAAT------ACGATTCTGAAGGATTTAGGAGAGATTTATGGGAGGCTGTTCGACCATCGTCCATTTCTTCAAGGAGAAATAAGTTATTTTGTCAAAGAATTTGAGGAGAAACGAAACGACCGTGAAGTCCAGCAACTATTTGAGATTTTAGAAAACGTCACAGAAATTAAGGAAACCCAAATTGAGAAAGCCTGTCGTCTGGCAGACAATCAGCTGTGCAGCGTCAACACAAACTTGGAGGTAGCTTTGAGAATGTGTAACAAGATTGTTGAAAGGAGCGTACTAACAGACGTGGAAAGCAAATTGTGCAAACGACGGGAGCAGCGGGTACAAGAAATGGTGAGCTTTGTCGAGGATATGGCAACTAAGAACTGTCGTGTGGACCAAATGTTTTCCCAAAAGGAGCAAGAGATGCGCAACTATTACATCCAGCTGGAGGAGACT [453]

Trogulus ATGACGACAGCTTCTGCAATT---ACTTTAACGAATCACTTGGGAGAAATTTATGGAAGACTTTTTGATCATCGTCCTTTTCTAAATGGAGAAATAAATTTCTTTCTCAAGGAGTTTGAGGAAAAGCGTAATGATAGAGAGGTTCAGCAACTTTTTGAAATCTTGGAAATGGTGACAGAAGTCAAGGAGACACAGATTGAAAAGGCATGCCGGCTTGGAGAAATGCATCTTAGTAATGTTACAACGGACTTAGAAGTTGCATTAAGGATGTGCAACAAAATTGTTGAAAGAGGAACGGATGAAAGCATGAGTGACGTATTGAGGCTGCGCCGGGAGGCTCGCTCACGTGAGTGGGACGAGTTTGTAAATGAACTTTCAATTAAGAACACCAAAGTTGACAAAATGTTTGAAGAGAAAGAGGACGAACTAAAACGCTATTACAAGGACCTACAAGGGAAA [456]

Ortho ATGTCTGGAACGGTTGGAAAT---AATTTAACGAAAAATCTAAGTGAAATTTACGCGAGACTGTTCAACCACCGAGCGTTTCTGGATGGCGAACTAACATTTTTCCTGAAAGAATTTGAGGACAAGAGGGAAGACAGGGAGGTACAACAACTATTCGAGATCCTAGAAACGGTGACCGAAGTCAAGGAGACGCAGATTGAGAAAGCCTGCAGACTCGGGGAAGCTAACCTCAGCAATGTCACGACTGATCTTGAGGTGGCACTCAGGATGTGCAATAAGACAATAGAAAGAGGAAGCAAAGAAAACACAGGTGAGGTCCTGAGACTCCGTAGGGAGGCAAGGTCTCGAGAGTGGGACCACTTTGTGGAGGATCTGGCCCTCAAGAACACCAAAGTGGACAGAATGTTTCAAGAGAAAGAAGATGAACTCAAGAGATATTACCAAGACCTCCAGACCAAG [456]

Hespero GTTTCTAATCAAGCTGTGAAT---AATTTCGTAAAAGATTTGGGTGAAATTCACGGCCGACTCTTTGATCACAGACCGTTTTTAAGCGGAGAAGTCGCGTTTGTATTGAAGGAGTTTGAGGAAAAACGGCAAGATAAAGAAGTTCAGCAGCTATTTGAAATCTTGGAAGTTGTTACTGAATTAAAAGAAACTCAAATTGATAAGGCGTGTCGATTAGGAGAAGCTCATTTGTCTAATGCAACTACAAACTTGGAAGTTGCTTTGCGTATGTGCAATAAGATTGTGGAGCGGGGAGTTCTTGCAAATGCTGATCAAGTATTAAAGTCCAAACGTGAAGACAGAGAGCTAGAATGGCAAAATTTCAAAACCGAATTGGCCGAGAAAAATAATAAAGTGGATAAAATGTTTGGAGAGAAAGAAGATGAATTGAAGAAATATTACGCGGATTTAGAAGAGAAA [456]

Leiobunum ATGCCTGTACAACTAGAAAAT---AATATTATAAAAGATTTAGGAGAAATACATGGAAGACTGTTTGACCACAGACCATTTCTTGAAGGCGAAATAAATTTGTTTATTAAAGAATTTGAAGAAAAGCGCAATGATAAAGAGGTTCAGCAATTATTTGAAATTTTGGAAACTGTGACAGAAATTAAAGAAAGCCAGATAGATAAAGCATGTCGAACGGGAGAAGCTCACCTAAGCAATGTCACAACTAATTTGGAAGTAGCATTACGTATGTGCAACAAAATAGTTGAAAGGGGAGTCTTGGAAAATTTTGATGACCTTCTCAAACCAAGAAGAGAGGATAGAGAACAAGAATGGAACAATTTCACTACAGATATGCTGGAAAAGAACGTTAAAGTTGATAAAATGTTCACAGATAAAGAAGAAGAATTAAAAAAATTCTACGCAGAGTTAGCAGAGAAA [456]

Proto ATGCCTGTACAACTGGAAAAT---AATATTATCAAAGATCTAGGAGAAATACATGGAAGGCTATTTGACCACAGACCATTTGTTGAAGGAGAAATAAATTTATTCATTAAAGAATTTGAAGAAAAGCGCAACGATAAAGAGGTTCAGCAATTATTTGAAATTTTAGAAACAGTAACAGAAATTAAAGAAAGTCAGATAGATAAAGCTTGTCGAACTGGAGAAGCTCACCTTAGCAATGTCACGACTAATTTGGAAGTAGCATTACGTATGTGCAACAAAATAGTTGAGCGGGGAGTCTTGGAAAATTTTGATGAGCTCCTCAAATCAAGAAGAGAGGATAGAGAAGAAGAATGGAATAATTTCAAAACAGATATGGCTGAAAAGAATGTGAAAGTTGATAAAATGTTCACGGAAAAAGAGGAAGAATTAAAAAGTTACTATGTGGAGCTAGAAGAGAAA [456]

[TICKFAST_NCHAR=1011;hypothetical protein XM_002400277]

Ixodes ATGGCGGCGCGG---------GTGACGGGAACCGCAAGGGTTTTAAACAAATTGCACGCCTCAATAGACTCCGGGAATTATTATGAGGCCCATCAAATGTACAGGACGATTTATTTCAGGTATCAAAGCCAGAAGAAATATGACGAGCTCAAGGAATTGCTGTACGACGGAGCCATCAAACTGCTCACGCTGCAACAGGTATGCGCTTCCTCTGTCCGGGCTTGTCTCGCTTCATACCCTCTCCCTCAAACGCATTCCCTGCCCCTC---------CCAGAGCGGCTGGGGCGGCTGCACAGCCTGCTGGCCGCGCACTCTCCCGAGCGGATGACCTTCCTGACCCGGGCCCTGCAGTGGTCGAGCCCGCCCTCCTCTTCCCCTGAC------GCGCCCTCCCGGGGCCACCCGGCCCTCCACCGGCTGGTGGCGCTCACCCTGTGGAAGGAGAAGAACTACCAGGAGTCGCGCTACCACTTTGTCCACTCGACGGACGGCGACGGCTGCGCCGCCATGCTGGTCGAGTTCCAGACGACCAAGGGGTACAGCAGCGAGATCGACCTGTTCATCGCACAGACCGTGTTCCAGTACCTGTGCCTACGGAACCCGTCGACTGCGACGCTGGCCTTCTGGGCGTACACCAGGCGGCACCCGAGCGTGCCCAGGGGTCCCCCCTTCTACCTTCCGCTGCTCAACTTCCTCTGGTTCCTGCTGCTCGCCGTCGAGAGCCGCAAGCTGGCGGTGTTCACGGTGTTGTGCGAGGAGTACCAGCCCACCATCAGCCGAGACCCCAGCTATCCCCAGTACCTGGACAAGATTGGGCAGCTGTTCTTCGGCCTGCCGCCACCCCCTAAGCCACAG------GGGGTGTTTGGAAACCTAATACAGACGTTGCTGGGAGGTCTGGAGGACGACGATCCGGGGACGTCTCACGGAGCG------GCCGGGACA------------TCGCCGGTGGCCCAGGTTCAGCCGGAGGAGCTGGACTAG [963]

Siro ATGGCGGCTAAA---TCTCGAGGGCAAGGTTTGGAAAGAGTTTTGAGGAAATTAAAATGTAGTATTGAAAGCGGAAATTATTATGAAGCACACCAGATGTACAGAACATTGTATTTTAGGTACGTTGGTCAAAAGAAGTATAATGAAGTTTTAGAACTGCTTTTCGATGGATCACTGCTGTTGTTGCGACACAAGCAGCTAACGAGCGGAGCTGATTTGGCAAATTTGCTCGTTGAGGTTCTTCAAAAAGCAGAAGAAACTGTTACAGAGGAATATCTAGAAAAACTGGCCAGGCTATATGGTTTCATGGAGGCAGATTCTCCCGAAAGGTTAGAGTTCTTGACCTCTGCCCTAAGGTGGTCAAGT---------ACGGAAAACGAC------AGTTGTCGGCGTGGCCATCCTCGTTTGCACCAATTAGTTGCACTAACACTTTGGAAAGAAAAGAATTATGCGCAGTCTCGATATCATTTCATTCATTCTACTGATGGCGATGGCTGTGCGTCTATGTTGGTCGAGTACCACGTTAGTAAAGGCTATCCCAGTGAAGCTGATCTCTTCATTGCCCAGGCTGTCCTACAGTACCTGTGCCTTAAAAACAAGTCAACTGCAACGGTTGCTTTCTATGCATATACAGAGAAACATCCATCTGTAGTCAGCGGTCCTCCTTTCATCCTGCCTTTGCTTAATTTCCTATGGTTTCTTTTACTAGCAATTGAAAATGGAAAACTAGCCGTCTTTACAGTTCTTTGTGAACAATATGAACCTTCAATAAAACGAGATCCGACATACCCAGAGTACTTGGACAGGATTGGCCAGCTGTTTTTTGGTTTGCCTCCTCCCCCGCCAAAACATAGT---GGGATTGTTGGGAACTTTTTACAGTCATTCCTAGGTGGCTTTGATGATGACGATACAGGTCGAGACGCTGCATTG------CCCGGACCATCA---------CAGAAATTACAATCAGTTCAACACGATGAATTAGATTAA [975]

Sitalc ATGGCAGCGAAA---TCGAGAGGGCAAGGTTTGGATAGAGTAAGAGGTAAATTAAAAACCAGTATAGAAACTGGGAATTATTATGAAGCTCATCAAATGTACAGGACGCTATACTTCAGGTATATAAGTCAAAAGAAGTTCGACTCTGCTCTTGAGTTATTGTACGATGGTGCTGTGCTCTTGTTAGAACACAATCAGTTAACCAGTGGAGCTGATTTAGCAAGGCTGGTGATCGAGGTTTTAAACAAGTCAGAAGTTCCTGTTTCAGATGAGTATATCGAGAAACTGAGTTATTTGTATGGTATGATGCAAGCAAATTCACCGGAACGCCTGGAGTTCTTGAACTCCGCAGTCATTTGGTCCAGC---------CACGGAGGAGAG------GGATTCTCCTACGGTCACCCGCGACTACATCAGCTCATTGCTCTTAAATTATGGAAGGAAAAGGACTATGCACAGTCAAGGTTTCACTTTATTCGTTCCACGGATGGCCAGGGATGCGCCACCATGTTGGTCGAATATCAGATCAACAAAGGATACCCTAGCGAAGTCGACCTATTTATTGCACAGGCAGTTTTCCAGTATCTATGCCTTAAAAACAAGTCAACAGCGGTGCTGGTCTTTGATGCGTATACAGATCAACACCCCCAAGTTCACAAAGGACCCCCTTTTCTTCTCCCCCTCCTCAACTTTATTTGGTTTTTACTCCTTGCAGTTGAACAGGGTAAATTAACAGTGTTCACAATACTATGCGAACAATATCAACCATCAATAACAAGAGACCCGACCTATCAAGAGTACTTGGACCGGATTGGTCAACTTTTCTTTGGTCTGCCTCCTCCACCAGCGAAGCAGCAA---GGAATATTTGGTAACTTTCTCCAGACATTTCTGGGAGGTCTAGAAGATGGAGACATGAACAGTGATGACATGGAGGGTGGGCCCGGACCATCG---------CGTCAGCTACAGTCCGTACAGCATGAAGAGCTAGATTAA [981]

Sclero ATGGCAGCCAAG---TCTAGAGGCCAGGGTTTGGACAGGGTGTGGGGTAAATTAAAAACCAGTATCGAAAATGGTAATTATTACGAAGCTCACCAAATGTACAGAACTCTGTACTTTAGGTATACAAGTCAAAAGAAATATGACTTGGCTCTCGACTTATTATACGATGGTGCTCTCTTACTGCTGCAGAACAATCAATTAACAAGTGGTGCAGATTTAGCGCGTCTCATGATAGAAGTCCTCACTAAAGCAGAACTCCCTGTTTCAGATGAATATCTCGAAAAACTAAGTCGCCTTTATGGCATGATGCATTCGGACTCGCCGGAGCGCTTGGAGTTTTTAAACGCGGCTGTCATGTGGTCTAGT------------CGAGGAGAG---CAGGGGTTCCCTTGTGGGCATCCACGGCTACATCAGCTGATTGCCCTTAAGCTTTGGAAAGAGAAAGACTATGCTCAGTCTCGATTTCACTTTATCCGTTCCACGGATGGGACTGGCTGCGCATCCATGTTGATTGAATACCAAATCAGTAAAGGCTACCCAAGTGAAATAGATCTGTTTATTGCCCAGGCTGTTTTCCAGTACCTATGCCTTAAAAACAAATCAACTGCCGTGTTGGTGTTCGACTCATACACGGAGCAGCACCCGCAGGTTCCGAAAGGTCCCCCGTTCCTCCTGCCGCTTCTTAACTTCCTCTGGTTTCTTCTTATGGCTGTGGAACAAGGGAAATTGGCAGTATTTACAGTGTTATGTGAACAATATCAACCATCTATAACCAGGGATCCAACGTATCCAGAGTACCTGGATCGCATTGGCCAACTATTCTTCGGCCTTCCTCCTCCCCCCTCCAAACAGCAATCGGGACTCTTTGGTAATCTTTTCCAGTCACTGATCGCGGGTCTAGAAGAAGGGGACATCGTGGGCGATGAGCCAGAGGGCGCCCCGGGGCCGTCC------TCCAGTCAACTGCAGCCGGTGGAACACGAGGAACTGGACTGA [987]

Trogulus ATGGAAAATAAA---GTGAGAGGCCAGGGCCTAGAAAGAGTTTTAAAGAAATTAAGAACCAGCATCGATAAAGGAAACTACTATGAAGCTCACCAAATGTACAGAACGTTGTATTTTAGGTATCTTAATCAAAACAAATATACCGATGTTTTAGAATTACTTTATGATGGAGCTACTTTGTTTCTTAAGCACAACCAGTTAACAAGTGGCAGTGATTTAGCATATCTGTTGGTGGATGTATTAGTAAAATCAGGAGCCGGAGTCACGGAAGAAAACATTGAAATGCTGGGTCAATTGTATAGCCTGATGGAAGCAGAATCAGTGGAACGTAGTTCTTTTTTAAACGCTGCTATTAGGTGGTCCACCCAGCAGAACAATCATGGAGAG------AGCTACATTCGCGGTCATCCAAGATTACATCAATTAATAGCACTCGCTTTATGGAAAGAAAAAAATTACCCGCAAGCTCGCTATCATTTCATTCGTTCAACAGATGGTTCCAATTGCGCAACCATGTTGGTGGAATATCACGTCAGTAAAGGATATCCCAGCGAAGTCGATCTCTTCATTGCTCAGGCAGTTTTTCAATACCTGTGCCTTAAGAATGCACCAACAGCGAATATCGTATTCTACGCATATACGAAACAACATCCTAGTGTTCATTGTGGTCCACCTTTCCTTCTTCCACTTCTCAATTTCATCTGGTTTCTTCTAACAGCGCTTGAACAAGGCAAATTGACTGTCTTTACTGTACTTTGTGAACAATATCAGCCATCTATAACGAGGGATCCCATGTACCACGAGTACCTAGACAAAATTGGACAACTGTTTTTTGGTCTCCCACCTCCGCCAAAGCAACAAGGT---GGATTAATAGGAAACTTAATTCAGTCTCTGATGGGAAGCATGGATGACGGAGAGGTTGAGAGCAAT---------------CCTGGCCCTTCT---------GTCCGTCTTCAGCCAGTGCAACATGAAGAGCTCGATTAA [975]

Ortho ATGGAGAATAAA---TCAAGAGGACAAGGTTTAGAAAGAGTTTTAAAGAAATTAAAAGCCAGTGTTGATAAAGGAGATTATTATGAAGCACACCAAATGTACAGAACTTTATATTTTAGGTATCTTAATCAAAATAAATACAATGATGTCTTAGAGCTACTATATGATGGAGCTACTTTGTTTCTGGAACACAATCAGTTGACAAGTGGTAGTGACTTAGCGTATCTCTTTGTTGATGTGCTTGTAAAGTCAGGTTCAGCAGTCACCGAAGAACACATTGAGAAATTAGGTCAGCTGTACAGTTTAATGGAAGTGGAATCTGTTGAACGTAGTACATTCTTAAGTGCCGCTGTTAGGTGGTCGACACAGCAAAATAACCATGGAGAA------AGCTATATCCGTGGTCACCCCAGGTTGCATCAGCTTATCGCCTTGGCACTATGGAAAGAGAAAAACTATCCACAAGCTCGCTACCACTTCATTCGTTCGACGGATGGTACTAATTGTGCAACAATGTTAGTGGAATATCATGTCAGCAAAGGGTTTTCCAGCGAAGTTGATCTCTTCATTGCTCAGGCAGTTTTTCAATACCTGTGCCTTAAGAATGCATCTACAGCCAGCATAGTTTTCTATGCTTATACCAAACAACATCCAGGTTTGCATTGTGGTCCACCGTTCCTCCTTCCCCTTCTTAATTTTATTTGGTTTCTTCTAACAGCACTTGAACAGGGTAAGTTGGCCGTATTTACCATACTTTGTGAACAGTACCAGCCATCTATAACAAGAGATCCTATGTACCATGAGTACCTGGACAAGATTGGTCAATTGTTTTTTGGTCTCCCTCCTCCACCTAAACAGCAAGGG---GGCTTAATAGGCAATTTAATTCAATCATTGATGGGCAGTATGGATGATGTTGATGTAGAGAACACC---------------CCTGGACCTTCT---------GTAAGTCTCCAAGCAGTGCAACATGAAGAACTTGACTAA [975]

Hespero ATGGAGACTAAA---TCGAGAGGTCAAGGCTTAGATCGAGTTTTGGGTAAATTAAAAGCGTCGGTAGAGAAAGGAAATTACTACGAAGCACATCAAATGTACAGAACGCTTTACTTTAGATATGTCAATCAGAAGAAATATAATGATGTATTGGAATTGCTTTACGACGGAGCGACGCTTTTTCTTAAACATAACCAGATGACGAGCGGCAGCGATCTCGCTTATTTGTTAGTGGACGTACTCGTTAAATCAGAAATGGACATTACAGATGAAAATATTGATAAAATTGGTCGTTTATATGGTTTAATGGAAGCCGAGTCTTTAGAGCGAGGTTCATTTCTCAACTCCGCCATTCGTTGGTCAAGTCTGGGAGGGGGTGGG---GAGGGTCAAGGTCATTCCAGAGGTCATCCTAAGCTGCACCAATCGGTGGCTCTCCATTTATGGAAAGAGAAGAATTATGCGCAAGCTCGTTACCATTTCGTGAGGTCATGTGACGGCCCTAATTGCGCAACAATGCTCGTCGAATATCAAATGAACAAAGGATATCCGAACGAAATTGATCTTTTCATAGCTCAGGCCGTCTTTCAGTACCTGTGCCTTAAGAATAAATCTACGGCTAACGTTGTGTTCTACGCTTATACCAAACAACATCCCGGTGTCCATTGCGGACCACCTTTCTTATTGCCATTACTCAATTTCTTATCGTTTCTATTGTCAGCCTTAGAACATGGCAAATTGACTGTGTTTACAGTCCTATGCGAACAATATCAACCATCCATTACCAGAGATCCAATGTACCACGAATATCTGGACAGGATTGGTCAAATATTCTTTGGACTCCCACCCCCGCCAAAGCAGCAAGGT---GGATTAATAGGAAATTTGATTCAGTCATTAATTGGCAGTTTGGATGATGGAGAAATGGAAGAGGAAGGAGGAAAT------CCGGGTCCA------------AGTAGAAAACAACCCGTACAGCATGAAGAATTGGACTAA [984]

Leiobunum ATGGAGTCTAAAACGACGAGAGGACACGGATTAGAAAGAGTTTTGGGAAAATTAAAAGCCAGTGTTGAAAATGGAAATTACTATGAAGCGCATCAAATGTATAGAACATTATATTTCAGGTATTTTAGTCAAAAGAAATATGGGGAGGTGTTAGAGTTGCTATACGATGGTGCTGTTTTATTCATTAAACATAACCAAATGGCAAGTGGTTCAGATTTAGCAAATTTGTTAGTTGATGTACTTGTGAAATCAGGGTCCCAAGTTACTGATGAGTATATAGATAAATTAGGCCGTCTCTATAGCATTATGGAATCTGATTCTATAGAAAGGAATGCATTTCTGAATGCTGCAATTCGTTGGTCAGCTCATGCAGAGTCTGGA---GAAAGT---GTTTATCCATCTGGACACCCACGTTTACACCAGTTAGTCGCATTATCTTTATGGAAAGAGAAAAGCTTTGCACAATCTAGATATCATTTTCTACGATCAGCTGATGGGACGAGTTGTGCAAGTATGCTTATTGAATACCATATAACGAAAGGTTATCCAAGTGAGGTTGATCTATTTATCGCTCAGGCTGTTTTTCAATACCTGTGCCTTAAAAATAAATCTACTGCCACAGTGGTATTTTATTCATATACTAAAGAACATCCTGATGTCAGTTGTGGACCACCATTCCTTTTACCCCTTTTAAATTTTCTCTATTTTCTATTAACTGCTCTAGAACAAGGTAAATTAGCTGTATTCACAGTTTTATGTGAACAATATCAACCTTCCATAACCAGAGATCCGATGTATCATGAATACCTTGATAGAATCGGTCAACTGTTCTTTGGTTTACCTCCACCTCCAAAACAACAACAAGGCGGATTGTTTGGAAATTTGATTCAGTCTTTAATTGGAAGCCTAGATGACGATGGTGATGAAAATGAAGAGGTT---------CCCGGACCTTCAACAGTGAAGCCTGTTGTTCATCAAGTTCAGCATGAAGAGTTGGACTAA [996]

Proto ATGGAGTCCAAAACAACGAGAGGACAAGGATTAGATAGAGTTTTAGGAAAATTAAAAGTTAGCGTTGAAAATGGGAATTACTATGAAGCGCATCAAATGTATAGAACACTATATTTCAGGTATTTTAGTCAGAAGAAATATGGAGAAGTGTTAGATTTACTATATGATGGTGCTGTTTTGTTCATTAAACATAACCAAATGGCAAGTGGCTCAGATTTAGCAAATTTGTTAGTTGATGTACTTGTGAAATCGGGGTCCCAGGTTACTGATGAGTACATAGATAAATTAGGCCGCCTATATAGCATTATGGAATCTGATTCTATAGAAAGGAATGCGTTTTTGAATGCTGCAATTCGTTGGTCAGCTCATGCAGAGTCTGGA---GAAAGT---GTGTACCCATCTGGTCATCCACGTTTACACCAGCTAGTAGCACTATCATTATGGAAAGAGAAAAGCTTTGCACAATCTCGATATCACTTTCTACGGTCTGCTGATGGTTCAAGTTGCGCGAATATGCTTATTGAATATCATATAACAAAAGGTTATCCAAGTGAGGTTGATCTATTTATCGCTCAGGCTGTTTTTCAATACCTGTGCCTTAAAAATAAATCTACTGCCACAGTGGTATTTTATTCATATACGAAAAAACATCCAGATGTCAGTTGCGGACCGCCATTCCTTTTACCACTTCTAAATTTTCTCTATTTTCTATTAACAGCTCTTGAGCAAGGTAAATTAGCTGTATTCACAGTTTTATGTGAACAATATCAACCTTCCATAACCAGGGACCCGATGTATCACGAATATCTTGATAGAATTGGCCAGTTGTTCTTTGGTTTACCTCCACCCCCAAAACAACAACAAGGAGGATTATTTGGAAATTTGATTCAGTCGCTTATTGGAAGCCTAGATGATGATGGTGATGAAAGTGAAGAGGTT---------CCTGGACCTTCCACAACAAGACCTGTTCTGCAACCAGTTCAACATGAAGAGTTGGACTAA [996]

[TICKFAST_NCHAR=777;hypothetical protein XM_002400991]

Ixodes AAGTCGTCCCGCAAGGAAGGCTACGAGCGTCCGGAGCCCCCA------AACACGACTCCGTACGCCGCCAGGTTCCGCGAAGGCCTCCGGGAGCTCAAGACGGAGTTCGGACTCCTCAAGGAGGAGATGCTGCAGACGCTCCGCTGCGACCCCAAGTACTACTTCCACGGAGACACCGAGGTCCTCTGGAAGTTCGACTCGGCCAAAGCGGTGGACGACTGGATCGTGACCGCCGATCGAGACAACGACGAGGGCTTCTCGACGGCCGACTTCACGCTGGGGCCCGGCATGACGGGCGTCTTCTCGGGGCATCTGGACACGAAACCGCCGAAGGACGGGCGCGTGCTCCACACGGGATACTGCAACATCAGGTCTCCGAGGGCCATGCGCTCTTTCGGCCGCAACGCGAGCTACGACTGGTCCGCGTTCACGCACTTGGAGCTGCGGGTGCGTGGGGACGGGCGGGCGTACATGCTGAACATTGGCATCGACGGCTACTTCGACGTCACCTGGCACCTCATATACAACTTCGCGCTGTACACGCGCGGCGGTCCGTACTGGCAGATTGCACGGATCCCGTTCTCGCGGTTCTTCCTGAGCAACAAGGGCCGGATACAGGACAAGCAGGGCCCGGTCCCGCTCACGCACGTGCGCAGCCTGGGCATCACGTGCGCCGACGCCGTGCCCGGGCCCTTCAGGCTCGAGATCGACTACGTGGGCGCCTACATCGACGAGGCGCACTCGGAGGAGTTCGCGTACGAGATGTACGAAACGCCG [771]

Siro GAACCGTACAGGGAGGGCGATCGTCGCTACGTCCGTGAA---------GATAAAAGACCAACTATCAAGAAAATTCGAGAAGGTATGTCATCAGTGAAAGGAGAAATTGCATTGTGGAAAGAAGAAATGAGAGACAGATTTGTAAACGACCCCAAAGTATTTCTGCCTGGAGATACAGAGATACTTTGGCATTTCGATTCTAAGGAAAATCTAGACAAATGGATTGTGACTTCCGACAAAAGCAACAACGATGGATTCAGTGAATGTGAACTCGTGAAGAGCAAAAGCGGAAAGGGACTGTTTTGTGGTACCCTGTCCACTGAACTACCCAAAGATGGGCGAATCATCAAGGCTGGCTACTGCAACATATCTACGATGAAAGCGAAAAAATCCTTTAATAGGGATACATATTTAGATTGGTCACCCTTCACACACATGGTAATGAGAGTTCGAGGCGATGGGAGAACATACATGATTAATCTGCATACGACAGGGTACTACGACATCACGTGGCACGATATGTACAATTTCTTTCTCTACACGAGAGGTGGACCTTACTGGCAAATGGCTAAGGTGCCATTCTCCAGATTTTTCCTGAGCAATAAAGGCGTGCTTCAGGACAAACAATGTCGTGTTCCGCTCGACCAAATTAGCAGATTCGGCATAACGTGTGCAGACAATTTGCCTGGAAATTTCCAGCTTGAAATCGATTATATTGGACTTGAATCGGATGAAAACTTCACCGAGAATTTTGCGTACGAAACTTACCAATATGGA [768]

Sitalc GAGTTTTATAAAAAAGGCGATGAAAAATATGAACAGCAAGATAAAAAGGACGACAAGTCTACAATCTGGCACATGAAGAATGGATTTACGATCATGAAAAAAGAAATCCTTTTGTGGAAAGAAGAAGTAAAGGAAAAAATGCAGGGACATCCAAAGATATTCGCACCTGGAGATACAGATTTTATATGCAAATTCGGCAAGAAGGAGGACCTTAATAAATGGATTGTGACTTCGGATAAAGATAACGACGATGGCTTCAGCGAGTGTGAATTACTGCTTAACAAATTCAACCATGGCCTTTTCCAAGGAGTTTTGGATAGACGACTACCGAAAGATGGACGAGTCATTCGAACAGGATACTGCAATATGACAATAAGAGCACCTCCGAAATCTTTCATGCGTCAATCCTATTTCGATTGGGAACCCTTCACTCACCTTGTGTTCAGGATCCGAGGGGACGGACGATCCTACACCATTATTCTTGATATTAGTGGTCCTTATGATGTTACGTGGTTTGACACCTACAATTTCGTTCTCTACACCAGAGGCGGACCCTACTGGCAAACAGCTAAGATCCCATTTTCAAAGTTCATGTTGTCCACCAAAGGTCGAATTCAAGACAAGCAAGGCCCATTTTTGAAGGACCATGTCAAGTCCTTGGGAATCACGCTCGCGGATCGCAGCCCTGGTCCGTTTCATTTAGAGATTGACTACATAGGACTGTATTGGGACCTCAACCATACTGAGACATTTGCTTATGAAATGTACCATGTGCCT [777]

Sclero GAGAAATACCGAAGTGGAGATCTTAAATACGAAAGGGTTCCAGAAAAGGATGACAAACCATACTTTTGGCACATGAAAAACGGCTTTAACATAGCGAAACGTGAGACAACCTTATGGTTAGAAGAATTTAGAGAGGACCTTAGGTTTGATCCCAAATCGTATAATCCTGGAGATACGGACATTTTTTGGAAATTCAACAATAATGAAGATCTGAGTAAGTGGATTGTGACATCTGACAAATCGAACGAAGACGGACACAGCGACTGCAGCCTGTCATTGACAAAGTACAACTACGGTCTATTTCAAGGGACCTTGAGTACCGTTCTCCCAAAAGATGGGCGAATCAGTCGTGCTGGATATTGTAACATCACCAACAAGAAACCCAATAAATCTTTCATGAGGAAGAGCTACTACGAATTGAAACCTTTCACCCACCTGGCCATGAGGTTTCGAGGCGACGGGCGCAGTTACGGCCTCAATCTTCTGACTCCCGGTTTCTACGATATAAACTGGCACGACACTTACAATTTTGTCCTGTATACTCGAGGAGGACCCTATTGGCAGACCGCCAAAGTTCCATTCTCCAAGTTCTTGCTGTCTAGCAAAGGTCGGATCCAGGATAAGCAAGGGCCTGTTCCCTGGGATCGGGTGCACGGGATTGGAATCACTTGCGCCGATCAGAACCCGGGGCCGTTCAGTTTAGAGATAGACTACATTGGTCTGCACTGGGACCCCAATCACACGGAAGAATTTGCCTACGAGTTGTATCAGTGGCAC [777]

Trogulus GAACCAAGTCGAAAAGGCGGCTCAAGGTATGACAAGCCC---------GATAACAGACCGGTGAAGGACCAGCTGAAGGAAGGTTTGAGTCTTTTAAAACGAGAAATCAAAGTTTGGACGGAAGAAGTCAAAGAAAATCTTAACATGGATCCCAAATTTTATTTACCTGGCGACTTGGAGGTTGTCTGGAAGTTCGATAGTGAGGAACTTGTTAAAAATTGGATTGTAACGGCTGATCGTGATAACAACGAAGGCTTCAGTCATTGCAAATTCGAATTCAGCAAGAACAAGAAGGGACTTTTTCACGGTTATATAGATAAGACATTACCTAAAGATGGCAGGATAGTTAAAGCTGGTTATTGTAATCTAACTTCGCAAAGAGTTGTGAAATCATTTTTGCGTGAAACGTATCTGAACTGGGACATGTTTACTCATTTGATAATGAGGGTACGAGGTGATGGTAGACCATACATGATAAATCTCAATACAGCTGGATATTATGATATCCAATGGCACGATCGACACAACTTTATTTTATATACACGAGGAGGACCTTATTGGCAAACTGCAAAAATTCCTTTTTCAAAGTTTCTTCTGTCCAGCAAAGGTCGAATTCAAGACAAGCAATGTGCAGTTCCGTTAAATAGAGTTAGTGGCTTAGGAATAACGACAACAGCCAAAAAGAGCGGACATTTTCAATTAGAGATTGATTATATTGGGGTTTATTTTGATGGTGATCATACAGAAAAATTTGCTTATGAAATGTATCAGGTACCA [768]

Ortho CAGTCCAGTCGCAAGGGAGATAAAAGATATGAGAAACCTCAT---ATAGATGACAGGCCATTAAAGGAAAAGTTGAAGCATGGTTTTCGGCTTATAAAAAGAGAAACTGCAGTCTGGTATGAAGAGATGCAAGAGAAATTAACGTGCGATCCTAAGTTCTACATGCCTGGCGACACAGAAGTTATTTGGAATTTTGATAGCCAAGATAATCTGAAAAAGTGGATTACAACTTCAGACAGTGATAACAGTGATGGATTCAGTTACTGCAATTTGGAACTCAGCAAGAATAAGAAGGGCTTGTTTCATGGTTATCTAGACAAGAGGTTACCAAAAGATGGAATAATATATAAAACTGGGTATTGCAACGTTTCATCAGTAAAAGCTAGAAAATCTTTTCTGAGGAAAGCCTACCATGAATGGGAAGAATTCACACACTTGATTTTGAGAATACGAGGAGATGGCCGCCCTTACATGTTACAGCTTCATACATCTGGTGATTTTGACATCCAATGGAATGATCAGTATAATTTTGTTCTATACACAAGGGGAGGACCTTATTGGCAAATTGCTAAGATCCCATTTTCTAAGTTCTTCCTGGCTAGCAAGGGTGACATTCAGGACAAACAGGCCCCAGTACCATTGGGACGAATAACCAATTTAGGTATTACATGTTCCGGAAAGAAAAGTGGACATTTTCAGTTAGAGATTGACTATATTGGGGTTCATTGGGACGGGGAGCACAAAGAAAAGTTTGCTTATGAAATGTACCAGATTCCA [774]

Hespero GAACAATATCGAAAAGGCGATAAGAAATACATAGTTCCCCAAGAGCCAGGACTTAAGCCTAAAATCGAAAGGGTCAAACATGGGTTATCATTGCTCAAAGAAGAGATCCAGTTATGGACCGAAGAAGTCAAAGAGAAATTAACTTGCGATCCTAAAATATTTATGCCTGGTGATACAGAAATTGTTTGGACGTTTAACAGCGAAGAAAATTTAAAGAAATGGATCACAACTTCAGATCGTGACAACAAGGAAGGTCACAGTTATTGTAATTTCGAGCTGAGTAAAAATAAAAAAGGTTTATTCAGCGGTTACATCGATAAAACATTGCCCAAAGATGGAGTTACGGCCAAAACGGGATATTGTAATATAACAGCAATGAAAGCAAAAAAATCTTTTCAACGTGATTCTTTTCTGGACTGGACAATGTTCACTCATTTAGTGCTCAGAGTTAGAGGGGACGGAAGACCTTACATGATTAATCTACTCACCTCTGGGTATTATGACGTGCAGTGGCACGATATGTACAATTTTGTACTCTACACCAGAGGAGGACCCTATTGGCAAATAGCTAAGCTTCCATTCTCGAGATTCTTCTTGTCTAGTAAAGGCCGTGTTCAGGACAAGCAATGTCGCGTACCTCAAGGCAGCATAACTAGTCTAGGAATAACGAGCAGTGCTAGCAAAAGCGGAGAATTTAGATTAGAAATAGATTACATAGGAATTCATTTCGATCCGCAACACGAAGAACTATTTGCGTACGAAATGTATCAAACTCCA [777]

Leiobunum GAGCCTCACAGAAGAGGGGACCGAAAATACTTGAGAGAA---------GATAAAAGAACGAAGTTAGAAAAAGTTAATGACAGTTTGCATTACTTAAAAGAAGAAGTTAAGTTATGGACTGAGGAAATGCAAGAAAAGTTCACTGGGGACCCTAAAATCTACGAGCCGGGCGATACGGATATAATTTGGACTTTTAATAGCCTTGAACACTTAAATAAGTGGATAGTCACAGCTGATAAGGATAATAATGATGGATTCAGTCACTGCTCTTTCGAGCTTAGCAAAAACAAAAAGGGTCTTTTCCACGGTTATTTAGATACTCAAACACCCAAAGATGGTGAAACCTACCGCTCTGGTTATTGTAATATATCATCTTTAAAAGTTAAAAAAGCATTTACTAACTTAGCCTACATGGACTGGACAATGTTTACTCATTTGGTTTTGAGTGTAAGAGGTGACGGACGCTCTTACATGATAAACATATTAACGACAGGTCCTTTTGATTTATCGTGGCATGACATGTTCAGTTTTGTACTTTACACTAGAGGAGGACCATATTGGCAATTTGTTAAGATTCCATTTTCAAAATTTCTTTTCTCTAGTAAAGGCAAAATCCAAGATAAACAACAACCTATCCCATTAAATAGGATTGTCAATTTGGGAATTACATGTGCAGATAATATTACGGGGCCTTTTCAATTGGAACTAGACTATATTGGGGTTCATTATGACGCTAACCATAAAGAAATATTCGCATACGAAATGTATCAAACACCA [768]

Proto GAACCTCACAGAAGAGGAGACCGAAAATACTTAATAGAA---------GATAAAAGACCTATTTTGGAACAAGTAAAAGACGGCTTAAATGTGTTAAAAGGCGAAGTTGAATTATGGTCCGAAGAAATGCATGAAAAGTTTACGGGGGACCCCAAATTCTACGTACCGGGTGATACCGATATAATTTGGGCTTTTAATAACCAGGAACAGCTTAAAAAATGGAAATTTACAGCTGATAAGGACAATAATGATGGTTTTAGTCACTGTTCTTTTGACCTTAGCAAAAATAAAAAGGGTCTTTTCCACGGTTACCTAGATACCCAAAAACCGAAAGATGGCGAAACCTACCGATCTGGTTATTGTAATATATCAGCTTTGAAAGCCAGAAAAGCTTTTAAAACCTTAGCCTATTTGGACTGGACACCATTTACTCATTTGGTTTTGAACGTAAGAGGAGATGGACGAGCTTACATGATAAATATATTAACATCAGGATATTACGATTTAACATGGAATGACATGTTTAGTTATGTACTCTACACGAGAGGAGGGCCATATTGGCAATTTGTGAAGATCCCATTTTCAAAATTTCTGTTCTCAAGTAAAGGCCGAGTTCAAGATAAACAACAACCCATACCGCTAAATAAAATTGTAAATGTGGGAATTACGTGTGCAGATAATATAACAGGGCCTTTCCAACTGGAACTAGACTACATTGGGGTTCATTACGATCCTAATCACAAGGAAATATTTGCATACGAAATGTATCAAACGCCA [768]

[TICKFAST_NCHAR=588;hypothetical protein XM_002402735]

Ixodes ATGGAGACTGATGTTGCCCTGCTCCTCGGCATCATTGTCGTTTTAATTCTGCTTTTGCTGAGCATCCTCGCTTTTCTCGGGTACTGCGGTCTCTTCGCGCCAATAGACATTAAGGCCGAGAAGCCACCCTTCAAGGACCTCGAAGTGGCGTACAAGTTCTGCCGCGGATCCTACAAGAACTCCGGTGCCCATTTCACCGAAGTTCATAGCATCGCACCCTCGCTAAAATGCATCGGAGTCTACTATGACGACCCG---AACGAGGTAGAGACGTTGCACCTGCGGTACCTGGTAGGCGTGGTCCTCAATGACACGGAGAGGCCGGCAACCAAGGAGGCGAAGCAGCAGCTGGAGTCGGAAGGCTACAAGACTGCCAGCTTCCCCGCAGTCGACCACGCAGTCACCACAGTGTTCCCCTTCCGCGGTTCCGTGTCCGTCATGATCGCGATGGCCAGAGTCTACCCCAAGCTCCGAGAGTACATTAAGGAGAAGCTGCTCTGTGCCCGGCCCCTGATCGAGATCTACGAAGGGGACCGAGTCGTGTTTGTCTGCCCCCTGGCCAAGCAGGACGAGTTCTACGTCGAGGAA [585]

Siro ATGGAGACCGATATGGATGTCCTTCTTGGGCTGCTAGCTATTTTGCTGCTTCTTTTATTAACTATCTCCGGTTATGCCGTTTATAGTGGATTATTTCACAAAATACAAATCAATGCTTGTAAACCGCCTTTCGAAAGTTTAATGATAGCATACAAATTTGCGCGTGGACCTTACAAAGAGTGCGGACACTTGTTTACAGAAATTCATTCTCTGGTTCCTCATTTGCAGTGTATCGGCGTTTATTACGATGATCCT---AAACAGGTGTCACCAGAGAACTTGCGGTACTTGGTCGGGGTCATAGTTGGAAATAAAGAAATGACTGCTAGCTCGGAAGACCAAGATGTTTTGCACAGCAAGGGTTACCGTTTCGCTACATTCCCTTCTGTCGATCATGTTGTCATGACGGCGTTTCCATTCCGAGGGCGTGTCTCTATTGCCATTGGTGTGGCAAGGGTCTACCCTGTACTGAGTAGTTATATTGAGGAGAAACGGCTTTGTGCTCATCCGATGATGGAGATTTACAGGGATTCCGACATTCTCTTCGTTGCACCTCTTTCAAAACAAGACGAATTTTACGTCCCCGAA [585]

Sitalc ????????????????????????????????????????????????CTATTATTCTTCACTATATTTGTATATGTGGTTTATTCTGGATTATTTGTAAATATCGAAGTAAAAACTTGTAAACCACCCTTTGATGCCCTGACAGTGGCGTATAAATTTGCTAGGGGTCCATATCGTAATTCTAGTAAGCTCTTCACCCAAGTCCACTCCCTTGTACCGGACCTGGACTGTTTTGGTATTTACTACGACGACCCA---CACAGGGTGTCTGCGGGAGGTCTACGTTACAGCGTAGGTGTTGTTATTGCTGATGGGAAGTTCACAGCAGACGACGCAACCAAGCAGGTCCTACAGGACGAAGGATTCAAACTTGCGACATTCCCCAACGTGGACCATGTTGTCATGTCCTCTTTTCCCTTCAACGGAACTCTATCCATTATGATCGCAGTTGCCAGGGTTTACCCTCATCTGGGCGAATACATTCGGGATCGTAAACTATGTGCTCATCCATTCCTCGAGATCTACGGCAAAAACGAGATCTTATTCTTAGCTCCTCTCTCGAAGCAAGACGAGTTCTATGTTCCGGAA [585]

Sclero ???GAAGCGGATGGATTTCTGGTTGTCGGAATAGTTTGCTTGTTGATTCTTCTCTTTGGAACAATATTTGCTTATGTGATTTACTCGGGACTGTTCACAAATATAGAGGTCAAAACATGTAAACCGCCTTTTGAGGCTCTTACCGTAGCTTACAAATTCGCTCGCGGCCCTTACAAGAATTCAAGCGAACTCTTCACCCAAGTTCATTCTTTAGTTCCTGACTTGGATTGTTTTGGAATATACTACGATGATCCC---CACAGGATTTCTCCAGGTGGATTACGTTACACCGTTGGTGTTGTCCTTGAAGATGGCACGTTCTGCGCAGATGACGAGACCAAGCAGCTTCTGCAAGATGCCGGATACAAGACTGCCATCTTCCCGGCCGTGGACCACGTCATCATGTCCAGCTTTCCTTTCAACGGAACGCTGTCCATCATGATTGCAGTTGCCAGGGTTTACCCACACCTGGGAGAATACATCAGGGAACGCAAATTATGTGCCCATCCATTCCTGGAGATTTACGGAAAGAACAATATTCTTTTTGTTGCCCCGTTATCAAAGCAAGATGAATTTTATGTTCCAGAG [585]

Trogulus ATGGAAACGGATGCCTACATATTCTTGGGCATCCTGATTCTCTTGCTCCTGCTCTTGACCACCGTACTGGCCTACGCGGTATATTCCGGCTTATTTCTCGAAATCGAAGTCAAGTCTTGCCGGCCAGATTTTCAGAGCTTGACCGTGGCTTATAAATTTGCTCAGGGTGTTTACCGAGAGTCCAGCGAACTTTTTACTGAGGCGCATGTCCTGGCCCCGGATTTGCATTGCATCGGCATCTACTATGACGATCCT---CGCAAGGATTCGCCCACTGGACTTCGATACATCGTTGGGGTGATAGTGAATGATGGCAAGCTGTCTGCTGACAAGGAGACTCTCGACCGCTTGACCAAAGAAGGATACGCATGTGCCACATTCCCCGCCGTTGATTACGTCGTCACAACAACTTTCCCTTTCAAAGGAACTCTTTCCATCATGATAGCTATCGCTAGGGTCTATCCCGTTCTGGCACAATACATTAAGGAGCATAAGTTGTGCGCTTATCCTTTCATCGAGATCTACGCGGATGAAAAGATCCATTTCATAGCTCCTCTGGCCAAACAAGACGAGTTTTATGTTGATGAA [585]

Ortho ATGGATTCCGACAGCTACTTGTTTCTGGGCATAATCGCCCTTTTAGTTCTTTTAGCCATAACATGTTTCGCGTTTGCAATTTATTCAGGTTTGTTCGTGGATATTGAAGTGAAATCATGTAAACCTGCATTTGAAAGTATGACTGTGGCTTATAAATTCGCCCGAGGCCTTTACCGCGAAGCCAGCGAACTTTTTACCGAGGCCCATGCCTTAGCACCTGACTTGCATTGCATAGGAATCTACTATGACGATCCG---AGAAAAGTATCTCCAACTGGTCTCCGTTACATCGTGGGAGTCATAGTTAACGACGGGAAGGTCTTAGCTGACGAAGCAACAACAGAACGCTTGAAAAAGGAGGGCTACAGCTTGGCTACATTCCCAGCCATCGACTATGTAGTCATGACGACATTCCCATTCAAAGGAACGCTATCTGTCATGATCGCAATTGCCAGGGTCTACCCTGTCATGGCTGAATACATAAAAGAACATAAGCTCTGTGCTCATCCTTTCATTGAGATCTACGGGGAAGAAAGCATCCACTTTGTGGCTCCTCTCGCCAAGCAGGACGAGTTCTACGTCAAAGAA [585]

Hespero ATGGAGACGAACATGTATTTGTTGATCGCTATTTTAGCCATTTTGATTTTATTATTCTTGACGATATTCGCGTACGTCGTTCACTCTGGTTTATTCGTCGAGATTAACGTGAAATCGTGTAAACCTCATTTCGATAATTTGGACATTGCTTACAAATTCGCCCGCGGCCCTTACAAAGAGTCCAGTGAACTGTTCACCGAGGCTTACGCAATCGCGCCAGATCTCAACACAATAGGGGTGTATTACGACGACCCC---AGAAAGGTGTCCCATGATGGATTGCGTTATCTGGTTGGGGTCATTATCAACGATGGTAAAGTGACCGCCAGCGACGAAATTCGACAACGTTTGACAGAAGAAGGCTACAAGTTCACCACTTTTCCCGCTGTGGACTATGTGGTGATGACCTCATTCCCTTTTAAAGGGACAGTGTCTGTCATGATCGCCATTGCAAGAGTATACCCAAGGATGGCCGAATATATCAAAGAGCACAAGCTTTGCGCTCACCCTTTCATTGAAATCTACCAACGAGATCAAATCATTTTCATGGCTCCTTTGGCCAAACAAGATGAATTCTATGTCGTAGAG [585]

Leiobunum ATGGAGACCGACTACTTTATGCTGTCTGGAATTTTGCTGCTCCTATTTCTATTGTTTTTAACCATATTTATTTTCGCTGTTTATTCGGGTTTGTTCTTTAACATTGAAGTAAAAACGTGTAAGCCTCCTTTCTCCGAATTGCAAGTCGCTTACAAGTTCGCCCGTGGTCCATATCGTAACTCTAGTGAGCTTTTTACCGAGGCCCATATGTACGCGCCCGATCAAAACTGTATTGGGATTTATTATGACAACCCCCAAGAACATCAAACCATGGATGGCTTACGATACATGGTTGGGATCATTCTTAATACCAGCAAACATGAAGCTGATGAAAAAACAAAAGAAACCTTGTTAACAAAGGGATACAAGATGACCACATTTCCCGCCGTTGACTATGTTGTGATGACAAGCTTTCCCTTTAAAGGAACTCTATCTATTATGCTCGGTGTAGCCAGGGCTTATCCAGAACTGCGAGACTACATAAGGTCCCACAAACTATGCGCACACCCTTTTATTGAAATATACGATGAAAAAGACATTCTGTTCGTTGCTCCACTGTCAAAACAAAGTGAGTTTTACATCCCAGAG [588]

Proto ATGGAGACCGACTATTTTATGCTGTCTGGAATTTTGCTGCTCCTGTTTCTTTTGTTTTTAACTATATTTATTTACGCTGTTTATTCGGGTTTGTTCATTGAGATCCAGGTTAAAACCTGTAAGCCTCCTTTCTCCGAACTTCATGTCGCGTACAAGTTCGCTCGTGGTCCATACCGTAACTCCAGTGAACTTTTCACCGAGGCCCATACCTATGCACCTGACCAAAATTGCATAGGAATTTATTATGACAACCCGCAAGAACATACCACCATGGATGGTCTGCGATACATGGTTGGAGTTATTCTCAACACCAGCAATCACGAAGCTGATGAAAAGACAAAAGAAGTACTTCTGTCAAATGGCTACAAGTTGACAACATTTCCAGCTGTTGACTACGTTGTGATGTCATCGTTTCCCTTTAAAGGAACTTTATCTATTATGCTTGGTGTAGCTAGGGTTTATCCGGAACTGCGAGACTACATTAGGTCCCTCAAACTATGCGCACACCCTTTTATTGAAATATATGACGAAAAAGACATTCTGTTCATCGCACCTCTGTCGAAACAAAGTGAGTTTTACACCTCGGAG [588]

[TICKFAST_NCHAR=669;hypothetical protein XM_002404569; part 50]

Ixodes GACGAAGAAACCAGGCTAGCCGCCGACATCGACACAGCCAACTGGGCGGGCTCTCAATCCGGAAGGCTCAAACAAGACTACGGACCCAAGGTGAATTTCAAGAAGAAGCGAGTAAAATCGGACGGGTTTCGGGGCCTACCTGCCTACTATACCCGAATCGAAGAGAAGATGCGCGAAAAC---GAGACCGTATCGGACTTTGAGGCGGTCGAACTGTGCAACTTGGACTACCACCCGAAACGCGGATCGTGCATCGACCCCCACTACGACGACTGGTGGCTTTGGGGCCCGCGTCTGGTGACGTTCAATCTGTTGTCTGACACGGTGCTTACGCTGTCCAGGCCCGAGGAACACTGGATGCTTACCGACGAAGATCTGATCGAGTCTGGGGACAGAGGCGCAGACGACGTCGAGCGGTTGGCGGCGGCGGCCCCGTTGCAGCACGGACCTGATTGCGGTTCGTGGTCGGACGCGTTCAAGGACTCTATCTCAACCCGGGTGCCGCCCAGGTTATGTGTCGTTCAGGTCTCGTGTCCTCGTAGGTCTATGCTGGTGCTGTGGAGTGACGCCCGGTACAAGTGGCACCACTCTATCCTCAGGGAAGACGTGAAGAGTCGGCGCGTGGCGATGACCGTCCGCGAACTCTCCGAGGAGTTTCTGGACGGGGGA [666]

Siro GATGAAGAAATTTATTATGCAAACCAAATTGATAGTCTGCCATGGATAGATTCACAATCAGGACGTAGGAAACAGGATTTTGGACCGAAGGTTAATTTCAAGAAGCAAAAAGTGAAGACGTCATCATTTACTGGTTTCCCATCATTCACCAAACAAATTTTCGCGAAACTTCAAAAACAA---TCTTTGCTCGATGACTTTCAAGCCGTTGAACTTTGCCATTTAGAATACGATTCTGAACGAGGTTCCTCCATCGAACCTCACATGGATGATAGTTGGCTTTGGGGAGAGAGACTTGTGACATTGAATCTCTTGTCCAAAACAGTTCTTACTTTGACTCCAGGCATTGCAGAATCCTCTGTTCCTCGTGAT---------------------------------------------------------------------------------------------------------------------------------------------TGTGCAATTCGAATAGACATGCCAGCCAGATCTTTGCTAATCCTACAGGGAGAGGCCAGGCATCGCTGGCTTCACGAGGTACAGAGAGAGGACATTGAGTCCCGTAGATTAGCAATGACGTGGAGGGAGTTATCAAAGGAATTTGTGGCTTCACGT [525]

Sitalc GTTGACGAACAGAAATTGCTTCAAGCCATCGACAACAATACATGGACAGCATCACAGTCTGGACGTCATAAACAGGATTTTGGACCAAAAGTAAACTTCAAAAAGAAGAAAGTGAATGCAAGTGTATTTACTGGATTCCCTGTTTCAGTTCAACCTCTTATCAAGAAAATACAAAACATACATCCACTACTCCAAGACTTTAAACCAGTCGAGTTATGCAACCTTGAATACATACCTGAAAGGGGTGCATCAATTGATCCCCACTTAGACGATTCCTGGCTTTGGGGAGAACGTCTTATAACAGTCAATATGCTTTCTGATACATTTCTCACTCTCACA---------CCCTGTCAGTCTGCTAAAGTTGGT---------------------------------------------------------------------------------------------------------------------------------------------TTTGAATTACTGATACCAATGCGTAGGTTTTCATTAATTTGCTTGTATGGTGAGTTACGCCATAAATGGTATCATGCCATTGCCAGGGAACACATTACACAGCGTAGAATTGCAATGACATTTCGAGAATTATCTGAAGAATTTGCTGATAATGGC [519]

Sclero GCCGAAGAATCTAGATTCATTCAATCGATTGATAACTATCCATGGATACCATCACAGTCAGGACGAAAAAAACAGGACTTTGGACCCAAGGTCAATTTCAAGAAACAGAAAGTAAAAACAGATAATTTCCGCGGCTTTCCGGGATTCGTGAAACCTTTAATAAAAATGGTTCAGCAGGCTGATTCCTTCCTTGAGGACTTCCACCCCGTAGAACTCTGTAACTTGGAATATGTGCCAGAAAGAGGCGCGGCAATTGACCCACACCGGGACGATTCCTGGCTCTGGGGTGAACGGCTCGTCACCATAAACATGCTCTCGGACACGTACTTGACATTAACT---------CCCTGCGATTCTGAAACGACGGCC---------------------------------------------------------------------------------------------------------------------------------------------TTCGAAGTTCTTGTTCCGATGCGAAAACGGTCCTTGGTCATTCTTTACGGCGCAGCCCGTCACCAATGGTTGCATTCAATATCCAGGGAAAATATCATCCACAGAAGAGTTGCAATGACTTTTAGAGAGTTGGCTTCCGACTTTCTGCAGAACGGA [519]

Trogulus ACTGAAGAGGCCAGATTAGTACAGTCAATGCTAAAATTTGAATGGATTTCTTCACAATCAGGCCGCAGTAAACAAGATTTTGGACCCAAGGTTAATTTTAAAAAGAAGAAAATTAATACCGCAACATTTTGTGGTTTTCCATTGGAAATGAAATTTTTGTTAGAGAAATTTAAAGAAGTA---CCCAAGCTTCATGATTTCTTTCCTGTGGAGCTTTGCAATTTGGAGTACATCCCAGAGAGAGGTTCTTCTATTGATCCTCATTTTGATGATTCATGGTTGTGGGGTGAACGCTTAGTTACAATCAATTTAGAAAGTGACACAAACTTAACATTGACA------CAGCTGTGCTCAGATAATGAACTTTAT---------------------------------------------------------------------------------------------------------------------------------------------AATAGAGTTATTGTTCCAATGCCTCGGTATTCCTTATTAATTTTGGAAGGTGAAGCCAGACACGAATGGATGCATTCAGTACATAGAGAAGACATAAGGAAACGCAGAATAGCTATGACATTTCGTGAGCTCTCCACAGACTTCTTACCTGGTGGA [519]

Ortho ATAGAAGAAGACCTATTAATTCATTCCATACAGACGTTTCCATGGGTTCTATCACAGTCTGGTCGCCACAAACAGGACTTTGGACCTAAAGTAAATTTCAAAAAGAAGAAAGTTACAGCATCGTCTTTCAAAGGTTTCCCATCTGGAATTGAATTTCTTATTGACAAAGTGAAAAAAGTA---GAAGAGCTTAGTGGATTTGTTCCAGTGGAGCTTTGTAATTTGGAATATGTCCCAGAACGAGGCTCAGCTATTGATCCCCATGTTGATGACAGCTGGCTATGGGGTGAACATCTAGTCACCATTAACCTAGAAAGTGACACTTATCTTAGACTAACACCATTGAATCTGAATCCAGACAGTGAAACCATT---------------------------------------------------------------------------------------------------------------------------------------------TCTGAAGTGATTATCCCAATGCCTCGATTTTCGTTATTAATATTATCGGGTGAAGCAAGGCATCAGTGGATGCATTCAATTTTGAGAGAAGACATTACAGAAAGAAGAATTGCTATGACCCTCAGAGAATTGTCCAAAGAATTTTCACCCGGGGGA [525]

Hespero GAAGAAGAAAAATTGCTTTTAAAAGGAATTGAGAAGTTCCCTTGGGTGGTATCACAATCGGGTCGTTTAAAACAGGATTTCGGTCCAAAAGTGAATTTTAAAAAGCAAAAAATCAAAACGACCAATTTCAATGGTTTTCCCGAAGATGTTAAATTCCTAATAGATAAAGTCTGCAATACA---------TTAGAGGATTTTGCACCCGTTGAATTGTGCAATTTAGAATACACTCCCGAAAGAGGTTCTGCAATTGATCCGCATGCGGATGACTCGTGGCTATGGGGCGAGCGATTGGTGACACTCAATTTACAAAGTTACACCAATCTAACATTAACG------CTACCACCTGGGGATAATTTCAAGCCT---------------------------------------------------------------------------------------------------------------------------------------------GTTCAAGTGAAGATTCCAATGCCTCGATTTTCCTTGATTATTTTAGAAGGAGAAGCGAGACATTTGTGGTTTCATTCTGTTTCGAGAGAAGATATTAAAGACAGACGCGTGGCAATGACATTCAGAGAACTGTCTGGACAATTCATTAAAGGGGGT [513]

Leiobunum GAAGAGGAAAAAAATATCATAAATCACATTGAAAAACAGAAATGGGTTGTTTCTCAATCAGGACGTTATAAACAAGATTTTGGCCCAAAAGTAAACTTTAAGAAAAAGAAGATTAAAGTGTCTACATTCGAAGGGTTTCCAAAATGGGTTAAAAGTTTAATAGACAAACTGAATCAAATC---CCTCAATTGGATAATTTTTACCCCGTTGAACTTTGCAATTTGGAGTATACTCCAGAGCGTGGATCCTCCATTGATCCACATAAAGATGATTCTTGGCTTTGGGGAGAAAGACTTGTTACTATTAATTTGAAGAGTTTTACTTTCTTAACACTAACT---------GCTAACAATAGCTTTGATAAT------------------------------------------------------------------------------------------------------------------------------------------------CGGAAAATTTTAATACCAATGCCACGTTGTTCAATGCTTATTCTACAAGGAATTTCGAGGCATATTTGGCTTCATTCTATATCTAGAGACCATATAAAAAGTCGAAGAATAGCAATGACATTTAGGGAACTATCCAATGAATTTTTGCCTCATGGA [513]

Proto GAAGAGGAAAAACATTTTCTACAGCAAATTGAAAATCACAAATGGGTTGTTTCTCAATCGGGCCGTTATAAACAAGATTTTGGCCCAAAGGTTAATTTCAAGAAAAAGAAAGTTAAAGTGACTGCCTTTGACGGTTTCCCAATATGGGTCAAGTGTTTAATAGACAAACTGAAGGATGTC---CCTCAACTTGATAATTTTTATCCAGTTGAACTGTGCAATTTGGAGTATACTCCGGAACGAGGTTCATCCATTGACCCCCATTTAGATGATTCTTGGCTTTGGGGAGAACGCCTAGTTACCATTAACCTAGAGAGTGATACTTTTTTAACTCTGACA---------AATCCCAATTCAAACAATGAAAAC---------------------------------------------------------------------------------------------------------------------------------------------AGAAAGATTTTTATACCAATGCCACGGTGTTCACTGTTGATTCTACAAGGAAATTCCCGACATATTTGGCTTCATTCTATTTCTAGACACCATATCAAAAGTCGAAGGATTGCAATGACATTTAGAGAACTCTCTGAAATGTTTTTGCCACTTGGG [516]

[TICKFAST_NCHAR=906;hypothetical protein XM_002404584]

Ixodes ATGGCTCGAAACGCTGAGAAGGCGATGACCACCCTGGCGCGGTGGAGAGCCGCGCAGTTGGCCGAACAGGGCAAGGGCGTTCAGGAACGGAGGCCCTTTCTGGCCTCCAACTGCCACAACCTGAGGAAATGCGAGAAGTGGAGGATGCAGGTCATCCGAGAGATCGCCAAGAAAGTGGCCCAGATACAGAACGCTGGGTTGGGCGAATTCCGTATAAGGGACCTGAACGATGAAATCAACAAGCTTCTACGAGAGAAAGGCCATTGGGAAGACAGAATCAAGGAGCTGGGGGGACCAGACTATGGGAAAGTGGGGCCAAAAATGTTGGATCAAGAAGGCAGAGAAGTTCCTGGAAACCGAGGGTATAAATACTTTGGAGCAGCTAAGGACCTTCCAGGTGTGCGGGAGTTATTTGAACAAGAACCACCGCCTCCGCCAAGACGCACTCGAGCTGAGGTGATGCGGGACATTGACGCGGACTACTATGGCTACCGGGACGACGACGACGGCCTGCTGGTGCCGCTGGAGCAGGAGCAGGAGAGGGTGGCTGTGGCACAGGCAGTCCGGGAGTGGAAGACGCTCAAGCGGGCCGCCATGGAGGGA------------GACAAGGCCGCCGCAAAAGAGGCCAAGAAGGGCGACAAGGACGAAGAGGACATCTACGCCGTGGAACCAGAGAGTGATGAGGAAATGGAGGATGAG---------CCTGCAGCAGAGTCTGCA------GAGGAGCAAGCAAGGTTCATCGCTCACGTCCCTGTGCCTTCACAAAAGGAGGTTGAAGAGGCCCTTGTGCGAAGGAAAAAGCTGGAACTCTTGGAAAAATATGCCAGCGACGTGTTACAGCAGGAGTCTGAAGATGTGAAACAAATGCTGGGTCTCCAGTAG [879]

Siro ATGGCAAGGAATGCCGAGAAAGCAATGACAACGCTTGCTCGTTGGCGGCAAGCACAGCTAGACGAGCAAAATAAAGCAAAACAGGAACGACGCCCTTACTTGGCGTCTGAGTGTCACAGCTTACGTAAGTCGGAGAAATGGAGAATGCAAATCATCAGGGAAGTTGCCAAAAAAGTCGCACAAATACAAAATGCTGGTTTGGGAGAATTCAGGATACGTGATCTGAACGATGAAATCAACAAGCTCTTAAGAGAAAAGGGGCACTGGGAAGTTCGAATAAAAGAATTAGGCGGACCTGATTATGCTAGAGTTGGACCCAAAATGCTGGACCACGAAGGAAAAGAAGTGCCAGGCAATCGTGGATACAAGTATTTTGGGGCAGCAAAAGACTTGCCTGGAGTGAGAGAACTCTTTGAACAGGAACCACCTCAGGCACCAAGGAAAACTCGAACGGAATTGATGAGGGCGATTGACGCAGACTATTACGGTTATCGCGACGACGACGACGGAATCCTCGTTCCTCGTGAGCAAGAAGAAGAGAAGAAAGTCGTTGCCAAATCGCTGGAGGATTGGAAGGAAAAGAAGGACGCACCCGTT------------------------------------TCCGAGGAAGCGAGCAAGGATGACGAGGAAGATATTTACGCTACAGCTGTGGAACCAGTTAGTGACGATGAAGAAGAATAC------GTTGGGGATGGCGAAGAA------GATGATCAGCCGAGGTTTATTGCTCACGTTCCTGTTCCTTCTCAAAAAGAGATTGAAGAGGCTTTGCTAAAGAAAAGGAAGATGGAGTTACTGGAGAAGTATGCTAGCCAGACATTGCAA---CACGAAACAGAACAAGTTAAAGAATTAATACCAAAATAA [855]

Sitalc ATGGCAAGGAATGCAGAAAAAGCTATGACGACCCTGGCTCGCTGGCGAGCAGCTCAGTTGGCAGAGCAGGGTAAAGGTGTCCAGGAACGTCGACCGTACCTGGCGTCGGAATGCTCGGACGTCAAAGAAGCTCAGAAATGGAGGATCCAGATCATCAGAGAAATTGCCAAGAAGGTGGCTCAGATTCAAAATGCTGGTTTGGGCGAGTTTCGGATCCGCGACCTGAACGATGAAATCAACAAGCTTCTCCGAGAAAAGGGTCACTGGGAAGTTCGCATCAAAGAACTGGGAGGACCAGACTACTTTCGTTTTGGGCCAAAGATGTTGGATCACGAAGGAAAGGAAGTGCCCGGAAACAGAGGCTATAAATATTTTGGAGCTGCCAAGGATCTACCAGGCGTTAGAGAACTCTTTGAACACGAACCTCCCGCACCTCCACGTAAAACACGTGCAGAGCTGATGCGCAACATAGACGCAGACTACTACGGCTACCGCGATGATGACGATGGCATTCTTATCCCTCTGGAGCAAGAGGCGGAACGGGAAGCCATAGCCAAAGCCCTCACCCTCTGGCAACAGAGGAAGGAGTCCAATACAGAC---------------------------------AAAGAACAAGACGAGGAAGAAGAAGAAGAGGATATTTACAAGACCCTGCCTGAGCCGTTGAGTGACGATGAGAAT------------GCTCAGCCAGATGACGAA------GGGGCAGAACCCAAGTTCATTGCCCATGTGCCTGTGCCCACCCAGAAGGAGGTTGAAGAAGCTCTTATCAGGAAAAAGAAGATGGAACTTCTTCAGAAGTATGCCAGCGAGACTTTGAGA---GCAGAAGTGAGGCACGTGGAAGACTTGCTTACCAGTTGA [852]

Sclero ATGGCTCGAAATGCAGAAAAAGCCATGACCACGTTGGCCAGGTGGCGGGCGGCGCAGCTGGCTGAACAGGGCAAAGGGGTTCAAGAGCGAAGGCCCTATCTGGCTTCCGAGTGCTCTGACGTCAAGCAGGCTCAGAAGTGGAGAGTCCAAATCATCCGAGAGATTGCCAAAAAGGTGGCACAAATTCAGAATGCTGGCTTGGGAGAGTTCCGCATCCGTGATCTGAACGATGAAATCAACAAACTTCTCCGGGAGAAAGGCCACTGGGAAGTCCGGATCAAGGAGCTCGGAGGCCCGGACTACTTTAGATTTGGACCAAAAATGTTAGATCATGAAGGAAAGGAGGTTCCAGGTAATCGTGGATATAAATATTTTGGTGCTGCCAAGGACCTGCCAGGAGTCCGAGAATTATTTGAACATGAACCTCCTCCATCGCCGCGTAAAACTCGAGCAGAACTGATGCGCAACATCGACGCCGACTACTACGGCTACCGCGACGATGACGATGGTATCTTGATTCCCCTGGAACAGGAGGCAGAGCGTGAAGCTGTGGAGTCTGCTGTCGCCCTCTGGCGGGAGAAGAAAGAGGCCAGCGCTGCCGAA---------------------TCTTCTCACCAGGAGGAAGAAGAAGAGGAAGAAGAGGAAGACCTCTATAAACGGCTGCCGGAACCAATGAGTGACGAAGAAGGA------------GCCGTGGCAGAGGAAGAA------GGAGCAGAGCCCAAGTTCATTGCCCACGTTCCTGTTCCCAGCCAAAAAGAGGTGGAAGAAGCGTTGATTCGCAAGAAGAAGATGGAGCTGCTACAGAAGTACGCCAGCGAAACCCTGAGG---GCAGAGGTCAGGTACGCAGAAGATCTCATGACAAACTAA [864]

Trogulus ATGGCAAGAAATGCAGAAAAAGCCATGACAACCCTAGCTCGTTGGAGAGCAGCTCAGCTCGCTGAACAGGGCAAAGGTGTGCAAGAGCGAAGACCATATTTGGCTTCTGAATGTTCCGATCTTAAAGCGGCGGAAAAGTGGAGGCTACAGATTGTACGTGAGATTGCGAAGAAAGTAGCTCAAATACAAAATGCTGGACTTGGAGAATTCCGCATTCGTGACCTTAATGATGAAATTAATAAACTTTTACGAGAGAAAGGACACTGGGAAGTTAGAATAAAGGAACTAGGAGGACGAGATTATGCGAGGTATGGGCCCAGGATGTTGGACCATGAAGGCAAAGAAGTTCCAGGAAATAGGGGATATAAGTATTTTGGAGCAGCAAAGGACCTTCCTGGTGTAAGCGAACTATTTCAGCAAGAGCCACCTCCAGCGCATCGGAAAACTCGAGCGGAATTGATGAAAGATATTGATGCTGATTATTACGGTTATCGAGATGACGATGATGGTTTGCTCGTTCCATTGGAGAATGAAGCTGAAAAGAAAGCTGTTGCAGCTGCTGTAGCTCGCTGGAAAGAGAAAAAGGAAACAGCGGAGACGGAG------GAGAGTGAAAATAATGAAATTGTAGAAGAACCTGTTACTGTTGCTGTCGAAGAAAATATTTATTTTGCCATTGAAAGTGATGACGAGGATGAGGATAATTCT------GAAAAAGAAAGAGAAGAAGAAAGTTTACAGCAACAGCAGCAATTTATTGCACATGTTCCTGTCCCATCGCAAAAAGAAATTGAAGCAGCCCTGTTAGAGAGGAAGAAGCAGGAACTTCTCAATAAATATGTCAGCGAGTCTTTACAA---GCCGAGAGTCAAAATCTGAGAGACCTTATGCATCATTGA [891]

Ortho ATGGCTCGAAATGCGGAAAAAGCCATGACCACATTGGCTCGCTGGAGGGCAGCACAACTAGCGGAGCAAGGAAAAGGGGTACAGGAAAGGAGACCCTATCTCGCCTCGGAATGCTCAGATCTAAAAGAGGCCGACAAATGGAGGCTACAGATTGTCAGAGAAATTGCAAAGAAAGTGGCTCAGATTCAAAACGCCGGACTCGGGGAATTTCGAATCCGTGACCTAAACGATGAAATCAATAAACTGCTGAGAGAAAAGGGTCACTGGGAATATCGAATCAAAGAACTGGGTGGCCGAGATTATGCCAGGTACGGTCCCAGGATGTTGGACCACGAGGGCAAAGAGGTTCCTGGGAACAGGGGTTACAAGTATTTCGGTGCAGCCAAAGATCTCCCAGGGGTCAGTGAACTATTCCAGCAAGAACCTCCTCCGGCCCATCGCAAGACCAGGGCGGAGCTCATGAAGGACATAGATGCAGACTACTACGGATACAGGGACGACGACGACGGCCTATTGGTTCCCCTGGAGATGGGGGCAGAGAAAGAGGCTGTCTCTACTTCGGTGGGTAAATGGAAAGACAAGAAG------------------------------------------------GAAGAGCAAGGGGCAGAGGCTAGCGAGGAAAACATCTACACTGCCATTGAGAGCGAAGAGGAAGACGAGGAGGATGGT---------GGAGGAGGGGAGATGGAAGACCTTCAACGCGAGCAGCAGTTCATTGCCCACGTTCCGGTCCCGTCCCAGAAAGAGATTGAGGCTGCCTTACTCGAAAGGAAGAAACGAGAACTGCTCAACAGATACGTCAGCGAATCATTGCAG---GCCCAGAGCCACGACCTCAAAGATCTTTTACATCATTAA [846]

Hespero ATGGCGCGAAATGCAGAAAAAGCAATGACAACTTTAGCCCGTTGGCGTGCCGCCCAATTAGCCGAACAGGGCAAAGGTCAAGAACAACGACGACCTTATTTGGCATCAGAATGTGGAAATCTTCATAGAGCTGAGAAATGGAGAATGCAGATTGTTAGAGAAATTGCCAAAAAAGTGGCACAAATACAAAACGCCGGACTTGGAGAATTTCGAATTCGAGATTTAAATGATGAAATTAATAAACTTTTACGGGAGAAAGGTCATTGGGAGTATCGAATCAAAGAACTGGGCGGTAGAGATTATGCTCGTTATGGTCCTAAAATGCTGGATCATGAAGGCAAAGAAGTGCCTGGAAATAGAGGATACAAGTATTTTGGAGCTGCCAAAGAGTTACCTGGAGTTTGGGAGCTATTTGAACAAGAACCTCCTCCCCCTCCTCGCAAATCCCGTGCTGAATTAATGCGAGATATAGACGCAGACTATTACGGTTACCGTGATGATGATGACGGATTGTTAGTTCCCTTAGAAATGGAGACTGAGAGAGCAGCGGTTGCCGCGGCCATTGAAAAGTGGAAAACAAAGAAAGAAGGTGCTGTTGCGGAGAAAAGCGAATCTGTCGTTAAGGAAAAAAACATTGAAACTGTCGAAGTAGTAACGGAAGAGGATATTTATACCGGAACGAATGACATTGAAGACGACGAT------------------TCTGACAAGGAATTAGAGGACAGCACAAATCAACCTCAATTCGTTGCCCACGTTCCTGTGCCTTCCCAAAAAGAAGTCGAAGAAGCGCTGCTAGAACGTAAAAAACAAGAATTGCTGCAAAAATACGTCAGCGAATCGATGCAA---GCCGAATCGCAACAGCTCAAAGAATTGCTTAATCATTGA [885]

Leiobunum ATGGCCAGAAATGCAGAAAAAGCCATGACAACATTAGCTAGATGGCGAGCAGCACAGTTAGCAGAGCAAGGTAAAGGCTCCCAAGAAAGAAGACCTTATTTGGCGTCCGAGTGCTCCGATTTAGCTGCATGTGACAAATGGAGGTTGCAAATTATAAGAGAAATCGCCAAGAAAGTTGCCCAAATTCAAAATGCTGGACTGGGAGAATTCCGAATCAGGGACTTAAATGATGAAATTAATAAATTACTTCGTGAGAAACGGCATTGGGAAATAAGAATAAGAGAGCTAGGTGGAAGAGATTATCTGAGATACGGACCTCGAATGCTAGATCATGAAGGAAGAGAAGTTCCCGGGAATCGTGGATACAAGTATTTTGGAGCTGCTAAGGATCTTCCTGGTGTTAGGGAACTTTTCGAACACGAACCCCCACCGGCAATGCGAAAAAGTCGAGCTGAATTGATGAAGGATATTGATGCCGATTATTATGGATACCGTGATGATGATGATGGCCTATTGATTCCATTAGAGAAGGAACAAGAAAAAATTGCTGTAGAAATGGCTATTGCTGAATATCAAAGAAAGAAAGAAGCCAGCCTAAATGAA---------GCCCCGAAGAGCACCACCGGAGAAGAAACAACTTCAAATGCGACAGATGAAAATATATATTCTATTGCACATAGCGATGATTCAGATGATGAAGATTCAAGTTCTTTCGGGGGAACAGATGAAGGAAGGGTTTTACCTCAACAACAATTTATAGCCCATGTTCCTGTACCATCTCAAAAAGATATAGAGGAAGCTCTGCTAGAGAGGAAAAAGCGTGAACTTTTAAACAAATATGTCAGTGAATCGTTGCAA---GCTGAAAGTGAACAACTGAAGGAATTAATGCCACATTAA [894]

Proto ATGGCTAGAAATGCAGAAAAGGCTATGACAACGTTAGCTAGATGGCGGGCAGCACAACTAGCTGAGCAGGGCAAAGGTTCGCAAGAACGGAGACCATATTTGGCCTCCGAATGCGCAGATTTAGCAGGATGTGACAAATGGAGGTTACAAATTATAAGAGAAATTGCGAAGAAAGTTGCCCAGATCCAAAATGCCGGTCTTGGAGAATTCCGAATCAGGGATTTAAATGATGAAATTAATAAACTTCTTCGTGAGAAACGGCATTGGGAAATAAGAATACGGGAGTTGGGTGGAAGAGATTATCTGAGGTACGGACCTCGAATGTTAGATCATGAAGGAAGGGAAGTGCCTGGAAATAGAGGATACAAATATTTTGGAGCTGCGAAGGATCTCCCAGGTGTTCGAGAACTCTTTGAACACGAACCACCACCGGCAATGAGAAAAAGCAGAGCAGAGTTGATGAAGGATATTGATGCTGACTATTATGGGTATCGCGATGATGACGATGGCTTACTGATTCCGCTAGAAAAGGAACAGGAAAAAATTGCCATTCAAGCAGCCATTGCTGATTACCAGAGAAAGAAAGAAGCCAGTCTAAATGAC---------GCCCCCAAC---ACTGCCGTCGAAGAAGCGATTACAAAAACAACAGATGAAAATATATACACTATGGAGCATAGTGATGATTCTGATGATGAAGATTCAAATTCATTTGAGGGACTTCATGAAGGGAGGGTTTTACCTCAACAACAATTTATAGCACATGTTCCAGTGCCATCGCAGAAAGATATCGAGGAAGCTCTGCTAGAGAGGAAAAAGCGAGAACTTTTAAACAAATATGTCAGTGAATCATTGCAA---GCGGAAAGTGAACAACTGAAGGAGTTAATGACA---TAA [888]

[TICKFAST_NCHAR=309;hypothetical protein XM_002405038]

Ixodes ATGATGCACGGGTGGATG------GAGAAAATACCGGACCAAGTTGGGTTCCTTATTTTGAACTCCGACGGAGGTGTCATCAGCTCTGGAGGTGAACTGGAGAATGAAGAGAGAATCGGTGGAATCATTCACAAGATGGTCTACTGCGCGGACAAGAGCGACCTGATG------CCAACCGACAACAGAGATCCCGTCAACAGAATGTCTATTCACCTCGGCAACTACTTTTATGCTGTGACGCTGTCAAATCAGAAGATTTACGTCTCCAAGAGGATGTACATACCAGCAGAGCCAATAAATGTTTGA [297]

Siro ATG---------------------GATCGCATTCCACAGCAGACGGGGTTTTTGGTCATCAATGAAGACGGGGGTGTTGTCTCGTCAGGTGGGGATCTTGAAAACAATGAGCATGTGGCCTCTGTCGTCATGGCGATGGCCAGGGTTGGCGACAGCACAGACTTC---------GGCGAAGATGGCAACGATCATTTCAAACGAATAACGATTAACTACGATGACCATCTCTACGTTGTGGCTTTTAATAACAAGAAAATCCATGTGGTGAAGAGAAAATACCTTCCAGTGGAACCAATACTCGCCTGA [279]

Sitalc ATG---------CCAGGAATTTTAGAGAACGTTCCTCACCAGACGGGCTTTCTCATCATCAATGAAGATGGTGGTGTCAGTTCTTCCGGCGGCGACCTTGAAAATGATGAACACACCGCAAACGTTCTAGTCGATCTGGTCAGAATGGTGGACCGAAAAGAACTGTTT------CAGTCTGGAAACATGGAGAACATGAAACGTATATGCGTTACTTTCGATGACCATTTCTATGCAGTCACGTTGTCAAACAAGAAACTGCACGTGGTCAAAAGGAAGCTGATTTCCAACGAGCCTGTCCTGGCGTGA [294]

Sclero ATGACTATG---CCCGGACTACTTGAAAATATTCCTCAACAAACTGGATTTCTGACAATAAATGAAGACGGAGCTGTCAGTTCGTCTGGCGGCGACTTGGAAAATGATGAACATACAGCAAATGTGGTTGTAAACCTAGTGAAACTTGCTGATCATAAAGATCTACTAAGAAGTTCATCAGAAACATTGGAACCATTCAAAAGAGTCACAATAACCTTTGAGAATCACATGTACGCCATCACATTATCAAACAAAAAATTACATGTTGTGAAAAGGAAATTGTTCTCCAATGCACCAGTTTTGGCTTGA [306]

Trogulus ATGTCGCAGCATTCTTCGTTGTTGGAACAAATACCTCATCAATCCGGATACCTCATTATAAACGAAGATGGAGGAGTCAGTGCATCTGGCGGAGATCTCGAAAATAATGAACACATAGCCTCCGTCATCTCAAACATAATAAGAATGGCTGAGAAGAATGACTTATCA------TCTATGAAAGAACAGGAACAATTCCGGAAACTCTCTATCAACTTTGAAGATCACATTTATGCAGTTGCAGTTAACAACAAAAAAATTCATGTTGTGAAAAGAAAATATATCCCAATGGATCCTGTTATCGCCTAA [303]

Ortho ATGTCGCTG---GCATCATTGCTGGAGCAAATACCACATCAAACAGGTTACTTGATAATAAATGAGGATGGTGCAGTCAGCGCATCTGGAGGAGACCTGGAAAATAATGAACATGTTGCTTCGGTCATCTCAAACATTGTAAGAGTTGCTGAAAAGAGTGATCTTTCT------TTGGCTAAGGAAATAGAACAGTTCCGAAGATTATCCATAAACTTTGAAGATCACCTGTATTCAGTAGCAGTTAATAACAAAAGAATTCATGTTGTGAAGCGAGCATATATACCAACAGACCCGGTGTTGGCTTAA [300]

Hespero ATGACGTTT---CCTGGCGTGATGGACCACATTCCGAACCAATGCGGTTATCTAATCATTAACGAAGATGGTTCAGTTTGCGCTTCTTCGGGCGAGCTTGAAAATGATGAACGAGTGGCCAATATCATCTTGAATATCGTTAGGATGGCAGAGAAAAATGAATTGTCT------ATTAATAAAGACAATGATCGATTCAAAAGGATTTCGATAAATTTCGAAGATCATCTATACGCTGTGACGGCCATTAACAAGAAAATCCACGTCGTCAAACGAAAATACGTGCCAATGGATCCTGTTGTTGTATAA [300]

Leiobunum ATG------------------CTGGATCACATTCCACTTCAAACTGGTTATCTTATAATCAATGAGGATGGAGGTGTTAATGCTTCTGGAGGAGATCTGGAAAATGATGAACTGCGAGCTGGTATAATTCTCAATATTGTAAGAATGGCAGAAAAAACTGACTTGATG------ACTTGTGAGCCCAACGAAAAGTTCAAGCGGTTATCAATTAACTATGATGATCACCTTTATGCAGTATCAGTTTTTAATAAAAAAATACATGTTGTTAAGAGGAAATACATTCCAAGTGATCCTATTATGGCATGA [285]

Proto ATG------------------TTGGAACATGTTCCACATCAAATTGGTTATCTAATAATCAACGAAGATGGGGGTGTTAATGCATCTGGAGGTGATTTGGAAAATAATGAACAAGTTGCTGGTATAATTCTAAATATTGTGAGAATGGCAGAAAAAAATGACCTAATG------GTTTGCGAACCAAATGAAAAGTTTAAGCGGCTATCAATTAACTACGACAATCATCTTTATGCTGTAACAGTTTCCAACAAGAAAATTCACGTTGTTAAAAGAAAGTATATACCAACTGATCCAGTTATGGCTTGA [285]

[TICKFAST_NCHAR=1509;hypothetical protein XM_002405366]

Ixodes CGCAAGGCGTGCAGCAAGGAGAAGCGCGCCAAGCTGCTCACGCAGCTGACGGGACTGGTCAAGGGGCGCGTCAAAGAGCTCATCTTCGCGCATGACACCGCCAGGGTCATCGAGTGCATGGGCGACCTGGGCACTCCAGAACACCGCAACCTCATCTTCGAGGAGGTGAAGGAC------ATGATTGTGTCCATGAGCAAGTCCAAGTACGCCAAGTTCATCGTGCGCAAGATGCTCAAGAACGGGACCCCGCAGCAGAAGGAACACATCGTGAAGGCCTTCAACATGCAGGTGGTTCAGTGCCTGCACCACGCCGAAGCGGCCTCAGTCTTGGAGACCATCTACAACGAGCACGCCAACGCGGTCCAGCGCTCGCAGCTCCTGCAGGAGTTCTATTGCAGGGACATGGCACTCTTCAAAGAGGACAAGGTGGTCACCTTCAAGGACGCCCTCGAGAAG---TCTGCCCAGCCGGCCAAGACTCTCGAGGACCTCAAGGAGATCCTGATGAAGATTGTGGGCAAGCCTGTGCTGGGCCACAGCATCATCCACCACGTCTTCCTGGAGTTCTTCAAGAACGCCGACGAGAACTCTCGCTCTGAGATGATCCGCGCTCTGGGTGGATCGGTGATCGAGATGCTCCACACCAAGGATGGGTCCCGTGTCGCCATGCAGTGTCTCTGGCACGGGACGGCCAAGGACCGCAAGGCCATCGTCAAGAGCTTCAAGACACACGTGGTCAAGATCGCCAAGGAGGAGCACGGCCACATGGTGCTCCTGGCGGCCTTTGACTGCGTCGACGACACCAAGTTGGTGGACAGCACGGTGACGGCAGAGCTGCTCCGGGAGCCGCTGGAGCTGATGACGAGCGCGAGCGGACAAAAGGTGCTCTCGTACCTGGTGGCTCCAAGGGACCCCCGCGTCTTCCACCCGAGCATCATCGAGATCCTTAAGGAGGGAGATGCCAGTGTCACCAGCAAAAAGGATCCACAGGTACGAAGAGAAGAGCTGCGCAAGGCTCCGGCCGCCGCGCTCGCCCAGCTGATAGCAGACAACGCCGAGCTGTTCCTGACGCACAACGGCCCTACCGCCGTCGTCGCCTCGGTGATTCTGACGAACCTCCGGGAAGGCGAAGCCACGGAAGCCTTCCGCAGCATCGCCAGGCTCCTGGCGAACTCCCCGTACGAGCCGAGCGCAGACCCGGAGAAGGCGCACCTGTTCGACCGCAACCACGCCCGCTTCTTCCTGAAGAAGATGACGCTGCACGACAGGGCG---------AGCAAGCAGGGGCAGAACTGCTTCAGCGCCATCCTGGTGGACGAGGTGCCCAAGGAAGCGCTGGTGACGTGGATCGGGTGCAACTTCGGGGCCTTCCTCCTTGTGAACTTGCTGGAGACCGGCGTGCCGGAGGTGGTGCGGGTGGTGAAGGAGGCGCTGGCCGGAAAGCAGAGTCTGCTCAAGAAGAGCGAGCTCAAGGGGTGCGAGGCGCTGAGGGAGAAGCTG [1491]

Siro AGAGACGACTGCGGTAAAGAGAAGCGAGAGAAATTACTCAAAGATTTGATGAATTTAGTGCAAGGGCACGTCAAACAGCTCATCTTTGCTCACGACACGGCTCGTGTGATCGAATGCATCGTCGCCCTGGGTTCGGAAGATCAGCGAACTGCTTTATTTAATGAACTGAAAGAC------AATATTGTCTTGTTGGCATCATCCAAATACGCCAAGTTCTTTCTTATAAAACTACTCAGTTATGGCACGAAACAGCAGAAAGATCTGATCATCAAATCACTAGAAGGGAATATTGTGTCTCTCATAAAACACACGGAAGCTGCTGCTGTGGTTGAATGTGCTTATAACGAATATGCGAATGTCAAACAGAGGTCAGCCATGGTGCAGGAGTTTTACGGGCCGTCGTTTAAGTTTTTTAAGGACGCGACGTCTACCAGTTTGTCCGAGTTGATTACTAAGGAGCCGGCGAAGAAAGACGAAATCTTAAAATTCATGAAGCCAACGTTACTCCGCATTTTGGAGAAATCCGTCATCCGACACAGCATTGTTCATCACGTATTGCGCGAATTTATGGACCATTGCGATGAAGGCGGACGTACGGAGCTAATCCAAGCTCTTCGCACTGGTTTAATGGAGATATTGCATACGAAAGACGGATCTCGTGTGGCCATGACTTGCATCTGGAATGGCTCGACCAAGGATAGAAAGACCATTCTGAAGAGCATGAAGACACGCGTGATCAAGATTGCCAAGGAAGAGCAGGGCCATCTGGTGTTACTTGCAGCATTTGATTCGGTCGATGATGTGAAATTGATCGAACGAATCATCATCCAGGAGTTGATTAACGGTTGTTTAGAACTATCCAGTGACCAATTTGGAAGGAAAGTTTTGCTCTACCTAATAAGCCCGAGAGATGGCCGTTACTTTCATCCTGACGTTGTCAATATTCTGAAACGAGGAGACAGTAATCCCCACAGCAAAAAAACTGCCACCGTCCGTCAGGAGGAATTGAGACAGTGCGTTTCGGCAGAGCTGCTCAAATTTGTGGCAGAAAACGCAGAGCATCTGCTTTCAAGTAAT---CCCATGTGCATGCTTTTACTGTCCATTCTGAAGCATGCTTCG---GGCGATACCACAAACGCTATGAAGTCGCTTGCCGATCTTTTAACAGAACCTTATCATCCGCCT------GCGGGAGTGGACGTACACATTGTCGAACACGTTGGGCTGTCGTTTGTATGCAAGAAGCTTCTGAAGGAAAGCTCCAAA------GAGAAGAACAACAACTCGCAACAGTTTGCAACATTGCTGACGGAAACAATCCCGACTGGGATTTTAAGAACTTGGATTGAATGCAATCGAGGAGCTTTTTTGTTACTAAACCTCCTTGAAACGAACATTCCTTCAGTGGTGAAGAGAGTGAAGAATGAATTGAAATCTGTTAAACATGTACTGGAATCGAAAGAATTTAAAGGAGCTGAGCTCTTGGTTAAGAAGTTA [1485]

Sitalc AGGGATGATTGTTCTGAAGAAAAGAAAAAGAACCTTATTTCTGAATTAATGAATCTGATCAGAGGAAATGTAAAGCAGTTCATCTTTGCACACGATACAGTAAGGGTGATTGAATGCTTAGTTGCAGCTGGAACTATTGAGCAAAGAGCACAACTTTTTGATGAGCTGAAAGGT------GATATACTTTCTGCTGCAAAGTCGAAATATGCGAAGTTCTTTTTAATCAAGATTTTGAATTATGGGACTAAGGAGCAAAAGGACACCATTATGAAATCTTTTAAAGGTCAATTGGTATCTCTTGTTAAACATACTGAAGCAGCACATGTTGTGGAAACAGCTTTCAATGATTGGGCAAATGCAAAGCAAAGATCGGAAATGGTACAAGAATTTTATGGGCCAAGCTTTGCTGTTTTCAAGGACGACAGTGCGAAAAACTTGGAGGATATCCTTGTTAAACATCCAGACCAAAAAGTTTCTATTGTGAATCACATAAAGGAAGTTCTGTTGAAGATTACTGAAAAGTCTGTGATTCGTCATAGCATAGTTCATCATATACTTCGAGAGTTTATGGTCCACGCAGACAACAAAAGCAGATCAGAACTGATTGAAAGTATCCGTGATGCAGTTGCAGAAATTCTTCATACGAAGGATGGCTCTCGAGTTGCCATGAACTGCATTTGGCATGGCACTGTCAGGGATCGCAAGAAAATGGTGAAAAGTTTGAAAATGCATGTTTGCAAAGTAGCCAAGGAGGAACAGGGTTCTTTGGTCTTAATGGCCACCTTCGACTGCCTGGATGACAACAAACTAGTGCAACAAGTTATATTGAGTAAATTATGTGACAACTTCCTTGAGCTAGCAGAAGATGTTCATGGCCGCAAAGTCCTTACGTACCTGCTTTGTCCCAGGGACAGTCATTTCTTCCACCCTGATGTTGTCAACATTCTCAAAGCAGGAGATAATAATGAACATAGTAAGAAGAGCCCTGAAGTGCGAAGAGAGGAGTACAGGCAAGCAGCGTCACCTCTTCTTTTAAAGGCTGCGAGTGAACACTTGGACACACTTGTCAAATCAAAT---GCAACATGTATGCTTCTGCTGGCCATTATCACACATTCTACT---GGTGACAAAAGAGAAGCTCTGGAGAGTTTGTTTTCACTTCTTCATTCCCCGTATCAACCCAAA------AGCGGAACTGATCTGCATCCATTGGAACAACCCGCCATGCACTTTCTTTTGAAAAAACTTTTTCAGCATGAAAAGTCAAAGAAGCCGGATGATGAAACTCTTGCACCCTTCAGTGAAATCTTAATATCTTCTTTGCCTGAAGGTATCATGAGGACATGGGTAGATTGCAACAGAGGAACATTTCTACTGCTTAATTTGCTAGAGTGTGGTGTTCCATCAGTTGTTGCACGAATAGCAAAGGAGCTGAAGGGAATGTACAGCCACTTGAAACTCAAGAAATACAAAGGTTCTGAGCTGCTTCTGGCTGAGCTA [1491]

Sclero AGAGAGGATTGTGCTGAAGATAAGAAGAAGAGTCTTTTGGCCGAACTTATGAACTTAATCAAAGGTCGAGTCAATAAGCTCATCTTTGCCCATGATACTGTTCGAGTTGTGGAGTGCATTCTGTCAATTGGAACTCAAGGCCAAAGGACTCAGCTCTTTGAAGAACTCAAAGCTTCCTGCAACTTTATCTTCATGGCCAAATCAAAATATGCCAAATTCTTTCTTGTCAAGATATTGAATTATGGAACGAAAGAACAAAAAGATCAGATTGTGAATTCCTTCAAGGGGGAGCTTGTCTCGCTAGTGAAACATACTGAAGCCGCTCTGGTTGTGGAGACGGCCTTCAACGAGTATGCCAATGCCAAGCAGAGGTCAGAAATGGTTCAAGAATTTTATGGTCCCAGCTTCACACTGTTCAAGGATCCGGAAGCCCGAAACCTGACGGCCGTCCTGGACAAGCATCCGGAACAGAGGCAGGCCATCCTGAGCAACATCAAGGAGATGCTCTTAAAGATTACAGAAAAATCTGTTATCCGGCATAGCATTGTACATCACGTTTTACGTGAATTTCTGAGCCAGGCGGATGCTGCCAGTCGGGCAGAGATCATCGAGAATATCCGTGACGCAGTCCCCGAGATGTTGCACACCAAGGATGGCGCTCGGGTGGCCATGAGTTGTCTTTGGCACGGCAACGTCAAGGATCGCAAGAAAATTATTAAAAGCTTGAAAATGCATGTTTGCAAAGTGGCTAAAGAAGAACATGGTTCGTTGGTGTTAATTGCCCTGTTTGATTGTGTGGATGACTCCAAGCTGATTGAGCAAGTCATCATCAAGAAATTGTGCGACCACTTTGAGGAACTGGCCCAGGACGTCCACGCCCGGAAGGTTCTCTTGTACCTGCTGTGTCCCAGGGACAGCCACTTTTTCCATCCGGATGTCATCAACATTCTCAAAGCGGGAGACGACAACGTGCACAGCAAGAAAATCTCAGAATTACGGAGAGAAGAACTAAAGGTGATGGCCTCACCCCTTCTGCTCAAGTTAGCAAGCGAGCACATGAACGTTCTCATCAAGACGAAC---GCGACCTGCGTTCTGCTTTCAGCCATCTTACAAAATTGTAGA---GGGGACCGAACGGAGGCCCTGAAAAGCCTGGCCCGGCAATTGGCCTCCGATTATCAGCCCAAG------ACTGGATCGGAACTGCATCCTCTGGAGCAGCCGGCCGTTCACTTCCTAACCAGAAAGCTCTTGCAGAGCGAGAGG------------AATTCCAAAGACATGGCTCTCTTCAGTGAAGTTTTGGTAAACAGTGTTCCTGAAGGCATGATGAAGAAATGGACAGAATGCAATAGGGGGGCATTTCTACTACTCAATCTTCTTGAAAGCGGGGTTGAATCTGTGACGACAAGAGTCAGAGAGGAAATGACAGGACTTCACAAACATCTCGGTTCCAAGGATTTCAAGGGAGCACAACTACTGCTTGCTCACCTC [1485]

Trogulus AGGGAAGATTGCCCTAAAGAAAAGAGCCTAAGTTTAGTAAGCAGATTAATGAATTTAGTAAAGGGTCATCTGAAAGAACTTATTTTTGCCCATGATACTGTCAGAGTCATTGAATGCCTAACGACACATGCAAGCCCAGCACAGCGATCCATGATATTTCTCGAGTTGAAAGAC------TCTATTTTACAATTGGCTCAAAATAAATATGCAAAATTCTATTTGATAAAGGTTTTGAGATGTGGCACAAAAGAAGAAAAGGATTTCATAGTCCAGGCGTTTATGGGAAAAGTAATAAAAAATATTCGCCATATAGAAGCAGCTGTAGTTTTAGAAACAGCGTATAATGAACATGCCAAAGCTAAGCAAAGAATTCAATTGGTGCAAGAATTTTACGGGCCAACATTTGCAGTCTTTAAAGATCAAGATATTTCAAATTTAAAAGATGTTTTGGATAATAATCCTGATCAGAAAAATGCCATCCTGACTCACATGAATGAAACTCTGGTTAAGCTAACATCTAAAACCGTGTTACGTCATAGCATTGTCCATCATGTCCTTCGCGAATATCTTACTTTGGCTGATTCGCAAGGCCGCACTGAAGTCATTGAAGCTTATAAAGAAAATTTGCCTGAAATTCTTCATACTAAAGATGGTGCACGTTCTGCAATGATGTGCTTATGGCATGGCACCACAAAGGACCGAAAAATTATTGTAAAAAGCATGAAAACCTTTGTAACCAAGATTGCAAAAGAAGAACACGGGCACATGGTTTTGTTGGCCCTTTTCGACTGTGTGGATGATACAAAGCTTGTCAGCAAGTCCATACTAAATGAAATAATTCCACGAATTGCGGAACTAGCTGGAGATCCTTACGGCCGTAAGGTACTGCTCTATTTAATAAATCCTAGAGACAGACTCCACTTTCATCCTGATGTTGTCAACATTCTTAAACAGGGTGATGGAAACGCTACCAGTAAGAAGGACAGTGATATAAGAATAAAGGAGCTCAGAGAAGTAATTGTTAAACCAGTAATGGAATATTTAAAAGAAAATGTGACAACATTACTGGAATCAAAT---TTCAATTGCCTTTTTGCACTGACTGTTCTTCAAAATGTG------GAGGACCTCACTGAAGGTTACGAAGCATTAATTAACTGCTTAATTGCTAAGCCATATTTACCTCTT---GAAGGTAAAGAATTGCATCCCATTGAACACCAGGCTTTCCAGCTGCTTCTTAAAAAACTTCTCCTTGCTGATTCCAAG---------CAGGATACTAAAAAAGTTGCCTTTTCAGAAGTTTTGATCAAACTTGTTTCTATTGAAACAATGAAGACTTGGATCCAGTGCAATCTAGGTTGTTTTCTTCTTTTGAGACTTTTGGAATCAAATCATGCAACTGTTATAGAAAAGACAGAAGAAGCAATGACTGGAATGAGGGGAAGTTTATCCGACAAGAAATATAAAGGAGCAGAATTGCTATTAAGCAAATTG [1482]

Ortho AAGGAAGCCTGTAAAGGTGAAGAACGAACCACATTAGTTAGTAAACTGATGAATTTGGTAAAAGGTCATCTGAAAGATCTTATATTTGCTCATGACACTGTCCGGGTTGTTGAATGCTTGACCACACATGCAAGTCCAGAACAGAGTTCAAGAATCTTTGATGAACTGAAAGAT------TCAATCATAGTAATAGCACAAAACAAATATGCAAAATTTTATCTGGTGTCGGTTTTGAGACATGGAACAAGAGAGCAAAAAGATGAAATCGTCAAAGCTTTCATGGGGAAAGTAGTCAAAAATATTCGACACGTCGAAGCTGCTTCTGTATTAGAAATGGCTTACAATGAGCATGCTAAGGCAAAACAGAGGATTGATCTGTTGCAGGAATTCTATGGACCCACATTTGCTGTCTTCAAAGATCAAGATATCGTCAGTTTGAAGGATGTCTTGGAAAAGAATCCACTTCAGAAGGACAGCATATTAAAGCACATGAATGACATCCTCATTAAACTGACTGACAAATCAGTTGTACGTCACAGCATGATTCATCATGTCCTACGAGAATATATAACTCTTTCCAGTCCTGAAGGCCGAGCCGAAGTTATTGAAGCCTACAAAGAACATCTGCCAGAAATTCTTCATACTAGAGATGGATCTCGAGCAGCCATGATGTGCCTTTGGCACGGTACCACGAGAGACAGAAAGACCGTTATAAAAAGCATGAAAACTTTGGTAGTAAAAATCTGTAAGGAAGAAAATGGTCATATGGTATTGCTGGCTCTGTTTGATTGCGTGGATGACACAAAGCTGATTGAAAAATCTATTCTCAATGAAATAGTTCCAAAAATTGCAGAACTCTTTGCTGATCCTTATGGCCGAAAAGTACTCATCTATCTAATCAACCCAAGGGACAGCCACCATTTCCATCCCGGTATCCTCCAAATTTTGAAAATGGGTGATGGAAATGCCCATAGCAAGAAAGACCAAGACATAAGGAGAAAAGAACTGAAAGCTGTTATTGAGAATCCATTACTGGCTTACTTTACCAACAATGTACCATTACTGTTACAATCAAAT---TTCAACTGTTTGTTTGGCATCGCTGTGCTTCAGAATGCG------AGCAATGTTTCCGTCGCCTATGAAGCTGTAGTCCTGAATTTAATAGCAGAGCCATACACT---------GAGGGAAACAGCTCACACCCCCTCGAGCAACGTGGATATGAACTCCTAGCGAAGAAACTTCTTCTCGATGATGCCAAA------CAGTCTTCAGAGGATTCTGTGGCCTTTTCCGACATCCTCATAAAGCATGCTTCTACAGAGATGCTTAAAAACTGGATCCATTGCAATAGAGGTTGCTTTTTACTGTTGCGCCTACTGGAAACAAATCGTCCAGAAGTGGTGGCTCACATCCAGAAAGCAATGACTGGATTGAATACAAAATTATCTGTTAAGCCGTATGACGGGGCAAGGCTTTTGCTGAATAAATTG [1479]

Hespero AGAGAGGATTGTCCTAGTGACAAACGAGAGAAACTCGTCAACAGTTTAATGAACCTCGTCAAAGGACATGTTAAGCAATTGATTTTCGCCCACGATACTGTTAGAGTGATCGAATGTTTAATGAAGTACGGGAAAGCGGAACAACGATCGATGCTATTCACAGAATTAAAAGAT------TCTATACTTCTGTTGGCTCAAAACAAATATTCAAAATTCTTCTTGTTGAAAGTGCTAAGATGCGGCACTAAAGAAGAGAAAGATTCCATCTTAAATGCATTTTTTGGACACGTTATAAAATTCGCCAGACATACTGAAGCTTCTGCTGTTTTGGAAGCCGCATACAATGATTATGCTCGCGCCCGTCAACGTATCGATTTGGTGCAAGAGTTTTATGGCCCCACATTCCGAGTATTTAAAGATCCGAGCATCACAAATTTGAAAGATGCTTTAGATAAATTGCCTGCTCAGAGAGATGCCATATTGCAACACATCAACGAAATACTCATCAAACTAACGGACAAATCTGTAGTCCGCCACAGTATCATTCACCATTTGCTGCGAGAATACTTGAGTTTGGCTCCAGAGAAAGGTCGTTCGGAGGTCGTCGAGGCTTATAAAGAAGTTTTACCCGAAATCTTGCACACTAAAGATGGTTCTCGTTGCGCAATGATGTGTGTTTGGTATGGAACAGCGAAAGACAGGAAATCGATCCTGAAAAGTTTGAAGAGCTTGTGCTTAAAAACAGCTAAAGAGGAACACGGATGTTTGATGCTCATGGCAATTTTTGATTCGGTTGATGATACCAAATTGGTTGAGAAAATTGTTCTTAGTGAGATCCTTCCAAATTTGAAAGAACTAGTTTTGGATCCAAACGGTCGCAAAGTTCTTTTGTACTTGATGCAACCGCGAAGCAGTCTGCATTTTCATCCGGACGTAGTCACAATCTTGAAACAAGGTGACGGTAACGAGCACAGCAAAAAAGACGTTGATTTAAGACGGAACGAATTGAGGAAAATGTCGTCATCTGCACTTACGCAATATTTGTGCAACAATATGAACACTTTGTTGCAATCAAAT---TCTAACTCAATGTTTGTTTTAACAGTCCTTAAAGGATTA------GATGATCCAACTCAAGTTTTTGAATCTATTGCTAATTTCGTGTTGGAAACTCCTTATCATCCC---ACACAAGGGATCGATGTGCATCCGATCGAAGCAACTGGATGCCAAATGCTCATCAAGAAATTGGTGTTAAATGATGGG---------------AATTCAGATTCAGTTCAATTTTCGACAGTTCTAATATCATTAGCACTTCCAAAAGTTTTGAAATCTTGGACTGAATGCAACGTAGGAGCTTTTCATCTATTACGCCTTTTGAGGTGTAATATTCCATCCGTTGTGGCTAAAGTAAGAGAAGAATTAATCGGAATGGATAAGAAACTATCCAAGAAAACCTACAAAGGCGCAGAGTTTCTACTAGAAGAATTG [1476]

Leiobunum AGAGAGGACTGCTCTGATGAAAAGAGACAGACATTATGCAAAACATTGATGTCTATTGTTCAAGGGCATGTGAAGGAACTAATCTTTGCTCATGACACTGTAAGAGTCATTGAATGTCTGATGGCACATGGAAATAAAGATCAAAGAAGTGCTCTTTTTGAAGAATTAAAAGAT------TGCATAGTGTTGCTTGCAAAATCAAAATATGCCAAATTTTTTGTTTTGAAAGCTATGAAATATGGGTCGAAATTGGAAAGAAATGCCATTATTCAATCTTTTGTTGGAAATGTTTCTAAATTAGTCAACCACAGCGAAGCTTCAAGTGTTCTGGAGGAGGCTTATAATGAATATGCAAATGCGTCTCAAAGGGCTTGTCTTGTTCAAGAGTTTTATGGCCCGACTTTTGCTGTTTTCAAGGAAGAAGGAATTACAGGCCTTTCCAGTGTTTTAGAAAAACACCCAGACCAGAAAGATTCAATTATGTCACATCTTAAAGAACTTTTGATGAAACTTACCGAAAAGCTTGTTGTAAAACATAGTATAGTTCATAGTGTTCTTAAGGACTACCTTAGCCTTGCTGATGAGAAAGGTCGGGCGGAAGTAATTGAAGCTTGGCGCGAATCTTTGGCCCAGATTGTTCATACACGGGACGGTGCCCGTTCTGCCATGATGTGCTTATGGCATGGAACTACCAAAGATAGAAAAGTCATACTGAAAAGTTTCAAGACTTTAGTTTCAAAAATCGCAATGGAAGAACATGGATCATTAGTTTTAATGGCTTTATTTGATTGCGTTGATGATACCAAATTTGTCGAGAAAGTTATTATTAATGAATTACTTCAAGATATGACAAAACTTGTAAATGATAACCATGGGCGAAAGGTTCTAGTTTATTTACTTTGTCCCCGTGATTCTCATTATTGCCATCCCGATATTGTTTCAATTTTAAGTAAAGGAGATGGTAATACACATAGTAAAAAAGAGGCAAATGTAAGGCAAAAAGAGTTGGCTAAAGTAGTTGAAGGATTTTATTTAAATTATGTTTCGGAAAACATAGCGACATTGATTAAAAATAAT---GCTACCTGTCTGTTCACATTAACTATTCTTCAGAAGGTTTCT---GGTGATAGAAGTGAGGCATTAATTTCCCTTGCAAAGTTTATGTTATCAGAGCCTTATCACCCTCCCCAAGAAGGAAATGATTTCCATGCTATAGAAAAAAGGGGTTGCAATTTGTTAATCAAAAGATTAATTCAAGAAGATAAACAAACT---AGAGATGTTGTTGACAAAGTTTTTTTCAGCAATGTTTTAGTAAATCAAGCTGCTCCAGGTATGCTTAAAACTTGGATTGAGTGTAACAGAGGAGCTTTTATTTTATTAGATCTTTTGGAAACAATGCAACTTTCGGTTGTGGATTTTGTATCTAAGGAGTTAAAAGGGCTAAATAAGAGACTGTCCCAACTGAAATATAAAGGCGCAGAATTACTCCTTAAGAAATTA [1494]

Proto AGAGAGGACTGTTCTGATGAGAAAAGACAGACATTGTGCAAAACACTGACATCCATAGTACAAGGACATGTTAAGGAACTAATCTTTGCTCATGACACTGTCAGAGTCATTGAATGTCTTATGGCTCATGGAAATAAAATCCAAAGAAGCGCGCTTTTTGAAGAATTAAAAGAT------TGCATAGTGCTGCTTTCAAAGTCAAAATATGCCAAATTCTTTGTATTAAAAGCTTTGAAATATGGATCTAAAGCAGAAAGAGATGCCATAATTCAATCCTTTGTTGGAACTGTCTCCAAGCTAGTGAACCACAGTGAAGCCTCAAGCGTCCTCGAAGAAGCTTACAATGAATACGCAAATGCCACTCAAAGAGCTTGTCTCGTACAAGAGTTTTACGGGCCCACGTTTGCTGTCTTTAAAGAAGAGGGCATCACCGGACTTAACAGTGTTTTAAATAAGCATCCAGAACAGAAAGACTCCATAATGAACCATCTTAAAGAAATTTTGATGAAACTTACTGAAAAGCTAGTGGTCAAACATAGTATAGTTCACAGTGTTCTTAAGGACTACCTAAGCCTTGCTGACGAGAAAGGTCGAGCTGAAGTAATTGAAGCTTGGCGCGAGTCATTGGGCCACATTGTGCATACTCGTGACGGAGCACGTTCAGCAATGATGTGTCTATGGCATGGAACAGCGAAAGATAGGAAGGTCATTCTGAAAAGCTTCAAAACTTTAGTTTCAAAAATTGCGATGGAAGAACACGGATCATTGGTTTTAATGGCGTTATTTGATTGCGTTGATGATACTAAATTTGTAGAGAAAGTTATTATTAACGAATTACTACAAGATTTGACGAAACTTGTAAATGATAATCATGGACGTAAAGTTCTGATTTATTTACTTTGTCCCCGAAATCCTAACTACTATCATCCGGATATTGTTAACATATTAACTAAAGGCGATTTTAATACGCATAGTAAGAAAGAGACAGCCGTTAGACAAAAAGAGTTGGCTACCGCGGTTGAGAAGGTTTATTTGAAGTATGTTTCGGAAAATATAGCTTCGCTAATGAAAAACAAT---GCTACATGTTTGTTCATGTTAACTATCCTTCAAAAGGTTTCT---TGTGATAGAACCGAAGCTTTTTCAAATTTCGCAAAGTTTATACTATCAGAGTATAGGCCACCC------CAGGGAAATGATTTTCACCCTATAGAACATAGGGGTTGTAATTTGCTAATGAAAAAATTAATTAAAGATGATCAACAAACT---CGAGATGTTGTTGATAAAGTTTTCTTTAGTGATGTCTTAGTAAATACGGCATCCCCAGGTATCATCAAAACTTGGATTGAGTGTAACAGAGGAGCGTTTATTTTATTAGATCTATTGGAAACAAAACGAAAGTCGGTTGTTGATTTTGTAACCAAGGAGTTAAAGGGACTAAACAAGAGGCTGTCCCAATTTAAATATAAAGGTGCCGAATTACTCCTTTTAAAGTTA [1488]

[TICKFAST_NCHAR=2328;hypothetical protein XM_002408178]

Ixodes GTCGCCGCGACCGTGTACGAGCTCGGCGGCACTACCACCAAGACGTCGCCCAATGTTCCGGCACCCTGGATCACGGCGGAAACAGACTGCGAGCTGGTGGGGCACAAGTACCTGGCCTGTCTCGACGCCACGGCAATGTCCCTCCGAGTCATGGCACTTGGGGAATCAGCTTCCTTCCGGGATGTGCCCCTGGCGTCCTTGGGACTCCAGCTCCAGGAGCCGACCTCCGCGCCGACCCTGGAGCGGCTGGACATCGCGGGACCCGCTCAGGAAGAACCGTACCTGGCGCTGCGCATGGCCCGTCGCGGGTTCGCCGTCCTCCGGATGACCAAGTCGGGAGTGCAGCTGGAGAAGCTGCTGCCCGAGGCGACACACCTCGCGTGCGCGCAGCTCAACGGGACTCCGCTTTTGGGCGTCGTGGCACCCGCGCAGGGCGAGGACCGGGCCCACGGGCTCACCATCTACGAGCTGGGGAGCCCCTGGCAGGAGCGCAGCGATCTCGAGGGTCAGTTCACGCTGCCGCCTACGATGGGCGACGTGTGGCGAGTCCACCTGCTGCCCTTCCTGCGCAAAGACCAGAGCCCCAGCTACAAACTGCTAGTGGTGACCCAAGACGAAGCGATGCTCCTGTTCCACCCACAGGGCCGTTTGCTGTGGACCCGCGAGGAGGCGCTCGCCTCGGTGCTGGCGGCACAGTTCATCGATCTGCCCTTGTCCGAGACGGATGCCAAGATCGAGCAGGAGTTTGGGGATGGCAATGCT---AACCTGGTGTCCATGTTCGTGACACGCATCACGATGCAGATCTGCCAACTCCAGAGCCTGGTTGTGGGCCTGCTGGCGGGGCTGCAGGGA------GGCATGGGCCGGCAGGAGTCGGCCGAAGCCAGCCAGGACTTGACGCGAGACAAGTTTGGCTTTAACAAGGTCATCCTGCTGAGCACAGCGGCCAGGAAACTGTTTGCGCTGGACAATCGCAATGGACAGATCATCTGGAGCCAGCACTTTGCAGAGCTGGTACCACTGAGCGAGGCC---GGCTCGGAGAAACTACCAATCTTCGTTCAGCGAACGACAGCCCATTACCCGCATCCGCCGCGCTGCACGGTTCTGGGCAAGCACCGAGACTCCGGAACTGGCTACCTGGTGTCTGTGAATCCCATCACGGGAGTGGTGTTGGACCAACGCGAGCTCCCCTACCGGGTGTTGCAAGCGACTGCCCTTCCCTTCCTGGACGAAGAGTACACCAGGGGCCTCCTGTTCCTGGATAGTGCCCTGAGAGTGCACACATACCCGTCCAGTGCTTCCGAACTACTGGTAGAGCACAAGGATACACAGTACTTCTTTCTGGCGAACCCCACCACGGGCACATTGACGGGTTACAGCCTAAGGCCTTCAACCAAGTCCCAGCTGGCCGTGGAACGAGTCTGGAGCGTGTCATTGCACCAGGATGGCCAAACCATCAGCCACGTCGTGATGCGGAACCCTCTGGAACATGTCCACTCCCAGGGCCGTGTGATGGGAGATCGCAGCGTCCTTTACAAGTACCTCAACCCGAACCTGGTTGCCGTGGTCACGGAGGGAGTGGACAGTGTGCACAAAGTGGCCAGTATCAACATCCACCTGGTGGACGCGATCACAGGTGCTTTTCTCTACTCGGCCAGTCACAAGCGTGCCCGTGGACCCGTCCACTTAGTACACTCGGAGAACTGGATTGTGTACTGCTACCACAACGAAAAGTCGCGTCGAGCAGAGGTCTCCGTGGTGGAGCTATACGAGGGAGCCACCCAAAGCAACACGTCTGCCTTCTCCTCTTTCACGTCCCCTCCGGCAGCCCTGGTGGAGCACCAGAGCTACGTGTTCCCAAGCTCCATCGAGGCCATGAGTGATACCGTCACCGAGAAGGGCATTACCAGCAAGCATGTCCTGGTGGCCTTGCCCAGTGGTGCCATACTAGAGCTACCCAAGGCACTCCTGGACCCACGAAGACCCATCACACCTACTGCCATGCACCGCGAAGAGGGCCTGATACCCTACATGCCCGAGCTACCCATCAGTGCAGAGCACATCGTCAACTACAACCAGTCCGTGGCCAGGGTGACGGGTTTTGCCACTGCTGCCTCTGGACTGGAGTCCACTTGTCTTGTGGTGGCTTACGGCCTGGACCTGTTCTACACCCGCGTAGCACCCTCCAAGACGTTCGACATCCTCAAAGACGACTTTGACCACGTTCTTATTTCGGTTGTGCTAACCCTCCTCATCCTTGTCTCGTACGTGAGCAAAAGGTTCTCGGCCAGAAAGGCGCTGCGGGCAGTCTGGAAGTAA [2316]

Siro GTATCAGTTACAATTTATGGCATCCTAGATGCCTTGGCTAAAAATTCGCCAAAGATTTCCGCACCCTGGATCACTTTGAAATCAAGTTGTTCGTTTGTTGGAAGCAAATATTTTGTCTGTGCCGACCAATCCAGTCGCAGTTTGCAACTCTTGCCAATCGCTGGCGGGGTCAAGTTCACGCAAGTTCCATTGGCCATGTTGGGGCTCCAATCGAATCAGCCATTGGTCGATTTTTCGGTGCATCCTTTACCTTATCAAGAA---ACGTCAGCTCAGCCGCTCTTTGCCGTTCAGCTTGGAAATGACGGAGTCATGCTTGCCCGAGTCACCCCTAATGGTGTCAAAGTTGTGAAAGTTTTTCCACAGGCTCTTCATATGACGGCGAGCATTCTTGATGATAAATTCTCTGTCTTTGTTACTATCCGTCGAGACGAAGATTATCGGATTCTCGTAATTGATGCTGACAGT------------GGCAAGGAAATGCCAGAATTAGAAACCGTCTTCTCCGTACCAGCAGATCATGGACAATTAGAAAAAGTTTTTGTTGTACCCTTTGCCAAGAAAGATAAGACTAGTAGCTACAAGATTATTCTTTCGATGCAAGATCATGCCATTTTGTTATTGCACAATCAAGGGAAAATTTTGTGGACCAGAGAAGAATCTTTAGCATCAATCGTTGCAACAGAAATAATTGATTTACCTTTATCCGAAACGGATGCTAAAATCGAAGAAGAATTTGGAGCACAGGACGCT---AACATAATTAACATGCTCGTCACTCGACTGACGATGCAACTGCTGCAGCTTCAAACGTTCCTTCTAAGGACGATCGCAGGTCTGCAAGGA------CATACGGACAAA---ACGACGGGCGACGAAACTCCCGATCTTACGCGAGACAAGTTCGGCCTGCACAAAGTTATTGTGGCAGCAACGAAACCTGGAAAAGTTTTTGGGATCGATAATTTGTCCGGACAGATCGTTTGGCGACACTTGATTCCGAATTTAACTCCATTTAATCGAAAT---AATTTGCCTCACTTTTATTTGATGGTTCAAAGGACGACAGCTCATTTTCCTTTTTCGCCAGTCTGCACTCTGATCGGTCGAGCAAAGGACGTGGATAATGGCCGACTGTACTCGTTTAATCCAATCACTGGGTCGTTTGTTGCTAGTCACACTCTCGCGGCACCAATCTTGCACGCGGCGATGCTCGGCATGATTGATGATCAATATCTGCGAGGAATTCTATTGGTCCATCCTAACAACAAGGTGCAATGCTTCCCGAGCGAAAGCAAAAATGTTTTACTGAAAACAAAATCAAGTCACTATTTTTTCTTGGCCAATATGACGACAGGAATTTTAACCGGGAATACATTTCACTTTTCTTCAGATGAGGATGTTAAAGTCACTGAAGTATGGAAGCTTAATCTCCCTACTGACAATCAAAAAGTCACTCAAATTGTTATGAAAAGACCTGCAGAGCACGTTCACTCGCAGGGTCGAGTGATGGGAGATCGTAGCGTACTCTATCGATATTTGAATCCTAATTTAGCTGTTGTTGTAACGCAAGGAAATGATCCCATTCAGAAGTGTTAT---TTGAATTTATACATGGTGGACGCAGTTACGGGAGCAATCGTGTATTCTAGCTCGCATAAACGGGCAAAGGGTCCGGTCCACGTCGTACATTCCGAGAACTCGATTGTCTACAGCTACTACAACGACAAGTCTCGCCGAATGGAGATGTCGTCAATCGAATTGTACGAAGGGAAAAGTCAAAGCAACACGACAGCGTTTTCATCTCTAAGTCCGCCACCGTTGCCCCTCGTCGAACATCAAACGTACATCTTTTCGGGAGGAATCGAAGCGATGACAGACACTGTCACTGATAAAGGAATAACCAGCAAACATTTACTTATTGCTTTACCAACTGGTGGAATTGTCGAATTACCGAAAGCTTTTCTAGATCCACGTAGACCCATCAATCCAACACCTGAGCACAGGGAAGAAGGTTTGATTCCGTATATGCCCGAACTTCCCATATCTGCAGAAGGAATTATTAATTATAATCAAAACATTTATCATGTTCGAGGCATTCAGACGGCTCCTTCGGGGCTGGAATCAACTTGTCTCGTCTTTGTTTATGGACTTGATCTTTTCTATACACGAGTGACTCCATCGAAAACATTTGACATTTTGAAAGATGATTTTGATCATCTCCTCATTTCAGCAGTTCTCTTAGGACTTCTGCTCTTCTCGTACCTCACTCGTAAATTGGCAGCTCGCAAAGCACTTCACGCTGCTTGGAAATAA [2295]

Sitalc GTTCAAGTTACCGTCTACAGGATCCTGGAGGCTAATGCCAAAACATCACCACAAATATCTGCACCATGGATTCATTCGAAAACAAGCTGCGATTTTGCTAGCACCAGTTACTTGGTTTGTGCCGTACAGTCTAGCCAAACGTTACAACTTCTGTATTTGAAAAATGGAGTCAGTTTTGTGGCCGTGCCTTTGCAAGCCCTGGGTCTTCAGTCCAAAGATTCTGGAGCGGAGATCCGTGTGCGTGCTCTGCCGTACCAAGAA---ACTTTAGGGCAACCTATTTTTGCCGTTCAACTGGGCAGGGATGGTATCTTTATCGGAAAGGTCGAAGCTTCCGGAGTCAAACTGATCAAGCTTCTGCCAAAGGCTGTTGATGTGACTGCTACTATTGTGGATGAAAAGTTTATCATATTTGCATTGGTAGAAGATGAAAGTGGTTACAGTATAATTGCGTTCGACCCGACTGAA------------AATACTGAAGTGCAAGATCTTACAGGAAACTTAATTTTGTCGCCCGACCATGGGAAAGCTGAACAGCTATTTGTCCTCCCATTTGCTAAGAAAGACGGAAGTTATGGTTACAAGATTATGTTATCTTGTGAGGATCACTCACTCCTTCTATTCCACCAGCAAGGTAAAATGCTGTGGTCCCGAGAGGAATCTCTGGCGTCCATTATTTCAGCCGAGATGGTGGATTTGCCCCTCTCCGAAACAGACGCAAAAATGGAGGAGGAGTTTGGGGCCTCTAATGGT---GACATGGCAAGTATGCTCCTGACCCGTCTGAGTTCACAGCTGCTTCAAATGCAGTCTTTCGTAGTCAAAGTGATCCAGGGCATTCAGGGTCAGCCAGAATCGGCCAAGAGCAGCGGACTCGTCGGACAGTTTGACATGACACGCGACAGATTTGGACTGCACAAGGTTATCGTGGCTGTTAGCTCAGCTAGAAAGGTTTTTGGACTGGATAGCCTGACAGGTCAAATTGTATGGAAACACTTTGTGGGTGGAATCACCCCTTTGACCTTGAAC---GGAGAGCCTTTCTTCTACATGTTTAATCAACGGACAACAGCCCATTTTCCAAGACCACCCGTTATGACAATTATTGGGAAGAGGAAGAATGGTCCCCAAGGGTTCGTGCATTCCTTCAACCCTATCACAGGTGGAGACATCACAACACAGACATACGATCAACCGATCGTTCAAGCCATGTTACTGGGTCAACCTGACGAGCAGTACCTCCGAGGAATTATACTTCTAGACTCCAATTTCAAGGTTCACGTTTACCCTACCAGCTGTCTGGAAGAGGCACTGAAAAGTAAAGATATCCACTACATGTTTGTGGCAAACGTCACCACCGGTACTCTCAGTGGGTACAGCTTT------------GGCGAAGGCTTGCAGGTGTCGTCAGTTTGGCAAGTTCACTTTCCTGTTGAAACTCAACTCATTACGCAAACTATCACCAAAAGGCTCTCTGAACATGTGCACTCTCAAGGGCGTGTGATGGGAGATCGCAGCGTTCTCTACAAATACCTTAACCCTAACCTGATCGTTGTGGTCACGGAAGGCACCGATCCCATTCAGAAAGTAGTGGCACTGAACCTGTACCTGGTGGACGGGGTGACGGGACTCATCGTTTACTCGGCCAGTCACAAGAGGGCCAAGGGACCAGTTCATCTTGTTCATACAGAGAACTGGATTGTGTACAGTTACTACAACGAGAAGTCTCGTCGTGTGGAGATGTCATCGTTGGAGTTGTACGAGGGTATGAGCCAAAGCAACACCACCGCGTTCTCGTCCCTCAGCCCGCCTCGCCTCCCATTGGTTGAACACCAGACATTCATTTTACCTGCAGGAGTTGATGCTATGATGGACACTATCACCGAGAAAGGCATTACGAACAAACACCTGCTGGTGGCTTTGCCACCAGGTGGCATAGTGGAGCTGCCCAAGATTTTCTTAGACCCACGCCGGCCAATCAACCCA------GATCATCGTGAAGAGGGAGTCATCCCATACATCCCCGAACTTCACGTTCCAGCGGAAGGCATCATAAACTACAATCAAAGTGTTATGAACGTCAAAGGCATTCACACTGCTCCCTCGGGACTCGAATCGACATGCTTGGTGTTTGCCTACGGTCTAGATCTGTTCTACACAAGGGTGACTCCCAGCAAGACATTTGACATTCTGAAAGATGACTTTGACCATTTCTTAATCTCCGCTGTGCTTATAGCTCTGCTCTTTTTCTCTTATTTATCACGCAAACTGGCTGCAAGAAAAGCTCTACGTGCTGCGTGGAAGTGA [2289]

Sclero GTTCAAATCACAGTTTACAGGATTCTGGAAGCGAACGCCAAAACATCGCCACAGATTTCTGCTCCTTGGATTCACTCTAAGACCAGTTGCGACTTTGCAAGCAATAGTTACTTTATATGTGCCGTACAGGCATCGCAAACTCTGCAATTGCTGTATCTGAAAAACGGAGTCAGTTTCGTGGCTGTTCCTTTACAAACCTTGGGACTTCAGTCCAAAGACTCGAACGCTGAACTGCGCGTGAGGTCTTTGCCGCTGCAGGAC---ACGTCCAGTCAACCCATGTTCGGGGTGCACTTGGGCAAGGACGGCATTCTCATCGGAAAGGTCGAGGCCTCAGGGGTCAAGGTCATCAAAGTTTTACCCAAGACTATTGATGTTACTGCAAGTATTATTAACGAAAAATACATCATATTTGCTCTTCTAGAAGATGAAAATGAATATAATGTGATCGCTTTCGATCCCAGCGAC------------AATTCTGAAATCGCAGAGTTTAAAGGCAACTTTGTTCTGGCCAACGACCATGGGAAAGCGGAACAGATGTTTATCTTGCCCTTCACAAAGAAGGACGGAATGCAAAGCTACAAAATCCTGGTGACCACCGAAGATCACTCCCTTCTCCTCTTCCACCAGCAAGGAAAAACCCTTTGGACTCGGGAAGAATCTCTGGCCTCCATAATATCGGCAGAAATGATGGACTTGCCGCTCTCCGAGACAGATGCTAAAATGGAAGAAGAATTTGGGGCTTCTGATAGC---AATATGGTTAGTATGCTCTTGATGCGTCTGACCTCTCAGGTTGTGCAGCTGCAGACCTTTTTCTTGCGAACACTGACCGGGATTCAAGGG------GACTCGGCCAAGGAGAATGGAATCGCCTCGCACAAAGACCTCACTAGGGACCGCTTCGGACTTCACAAAGTCATTGTGGCTGCCACCGCTGCTCGCAAGGTATTTGGCATCGATAGCCTGTCCGGTCAGATTGTATGGAAACATTTTGTTCCTGACATTGTTCCACTGCGGCTCAAC---GATAAACCCTTCTTCTACCTTTTTGTTCAAAGAACTACGGCACACTTTCCGCGACCACCAGTTTTGACAATCATCGGAAGATCAAAGGACGGTCCCCAAGGCTATGTTCATTCCTTCAATCCGATCACGGGAGGTTCCGTCTCCAGCCATCAATTTGATCGTCCAATCGTACAAGCCATGCTCTTAGGACAGCCCGACGAACACCACCTGAAACCCATTTTACTTCTGGACTCGGAATTCAAGAGCCACGTTTACCCGTCCAGCTGCATGGATACTGCACTACGTTTCAAGGACACTCACTACATGTTCATCGCAAACTCCACCACAGGCTTGCTGACCGGCTACACCTTT------------GGAGAGTCTCTCCAAATATCTCCTGTGTGGCAGGTCAATTTTCCAATTGAATCTCAAACGATCACTCAAGCAGTCACCAAAAGGTTATCGGAGCATGTGCACTCGCAGGGCAGAGTTATGGGAGATCGGAGTGTCCTTTACAAGTACTTGAACCCCAACTTGATTGCAGTCATCACAGAAGGAAATGATCCCATTCAGAAGGTAGTGGCCCTGAATTTGTATTTGGTCGATGGCGTGACCGGCCTCATCGTTTACTCTGCTAGCCACAAGAGGGCCAAAGGACCTGTCAACCTTGTTCACACCGAGAACTGGATTGTGTACAGCTATTACAACGAGAAGTCCCGGCGAGTGGAGGTGTCTACCCTGGAGCTCTACGAAGGCTTGAGTCAGAGCAACACCACAGCCTTCTCGTCTTTTAGTCCCCCGCAACTCCCCCTGGTGGAACACCAAACGTTTATCTTGCCGGCAGGTGTGGAAGCCATGATGGACACCGTCACTGAGAAGGGCATCACCAATAAACACCTCCTCGTTGCCCTTCCACACGGAGGCATCGTTGAACTACCGAAGGCCTTCTTGGACCCTCGTCGACCTATCAACCCACTGCCGGAACACCGCGAGGAGGGTCTGATCCCTTACATCCCGGAACTTCATGTGCCAGCCGAAGGAATCATCAATTACAATCAGAGTATCCACAACGTCAGAGGTATCCACACTGCGCCTTCCGGACTCGAGTCGACCTGCCTGGTCTTCTCTTACGGACTAGATCTGTATTTCACGAGAGTGACACCATCGAAAACTTTTGATATTCTGAAAGACGACTTTGACCATCTCCTCATTTCTGCCGTCTTGATTGCACTCTTGCTTCTGTCGTACGCTACACGAAGGCTGGCAGCAAGAAAAGCCTTGAGGGCTGCCTGGAAGTAA [2289]

Ortho GTACAAGCCACCATCTACAGTGTCTCTGAAGCTAATGCTAAAACATCTCCCCAAATTTCGGCCCCATGGATACAAGCAACTACAAGTTGTGCATTTGCTAGCAGCAGTTACTTTGTTTGCATTGTCCAATCATCAAAAACACTTCAGCTTCTGTATTTAAACAGTGGAGTCAGCTTTGTCGCTGTGCCACTTAAGTTCCTAGGCCTTCAAACAAATAATATGAATGCTGAACTGAAGGTCAAACCTCTGATGCATCAAGAC---AGTTCAACTCAGCCCATGTTTGCAGTAAATTTGGGAGGTGATGGAATGTTGCTGGGCAAAGTAGAAGCATCTGGTGTCAAAGTTGTCAAGGTTCTCCCGCAGGCTGTGGATGTTACAACATGTTACTTCAGTGGAAAGTTAACTATTCTTGCTGTCCTTAAATTTGAAGAGCAATACAAAGTGGTTGCTTATGATCCTGAAAGT------------GGTAACGAAATACCAGAGTATGCCGGATCTTTTACCCTATTTTCTGACCATGGAAACATTGAAAATGTCTATATCCTACCCTATGCCAGAAAAGATAATTCCATGAGCTACAAGATTTTATTGACAACAGAAGATAGCGCAATTAACCTATTTCATCAACAAGGTAAACTCCTATGGACTAGGGAAGAAGCACTGGCTTCAATCATTTCCACCGATATGTTGGAGCTACCTTTGTCCGAAACAGATGCTAAAATAGAAGAGGAATTTGGTCTAGCTGGAGCA---AATGCACTGACAATGCTGCTCACCAGGCTGACGACACAATTGTTGCAGATCCAGAACTGTTTTCTGAAAACCTTGGATGGAATTCAAGGG------AAAAAAGAT---------GAGCTGCTGCTCAGTAAAAGCCTTGTTCGGGATAGATTTGGATTACATAAAGTCATCGTTGCTGCGACAGAATCTGGAAAAGTATATGGAATAGATAGTCTGAATGGTGCAATAGTCTGGAAGAAACTAATTCCGGGCATATCACCAATAGTTTCAACTGTGCAGGATCCATTGTTCTTCCTTTTTGTCCAGCGCACCACTGCACATTTTCCTCACCAACCTGTGTGCACACTACTTGGCCGACAGAAGGGTTTAGAAGAAGGATATCTTTTCTCCTTCAATCCAATATCCGGAGCCGAAATTGGTTCTCAGAAGCTGCCATTCAAGGTAGCCCAAGCTATTATGCTGAGTGAAATGGATAGAAACTACATGAAGGGTATCGTCTTACTAGATCCACAGAAAAGGGTTCATGTTTATCCACCTGAGTCGGAAGAGGCTCTGTTGAAATCAAAGAATTCTTGCTATGTCATGTTAGCGGATTCCTCCATTGGAATGGTTACTGGATATAGCTTTGCGCTTTCATCCCCAGGGGATTTAAAATCTTCTGAAGTCTGGAGGATAACTTTACCCATTGAACAACAGTCAATCACTCAAGTTCATCTTAAAAAGATTTCAGAGCATGTCCATTCACAGGGTCGTGTAATGGGTGACCGAAGTGTTCTGTACAAATATTTAAATCCCAACTTGGCTGTGATTGTTACGGAAGGAACCGACCCTATCAACAAATATGTG---TTGAATCTATATCTTGTGGACGCAGTTACTGGTCTAATCGTCTATGCATCGACTCATAAGCGATCACGTGGACCTGTACATGTTGTTCACTCTGAGAATTGGATTGTTTACTCGTATTACAACGATAAATCTCGTCGCGTTGAGCTCTCATCAATTGAACTCTACGAGGGAAAGAGCCAGAGCAATACAACTGCCTTCTCGTCTCTGAGCCCACCCGCTCTCCCTCTAGTGGAGCATCAGAGCTTTATATTCCCTGCTGGGATTGAAACAATGACGGAAACTGTAACGGAAAAGGGCATTACCAATAAACATATCCTAGTTGCCCTGCCTCTTGGTGGAATTGTTGAACTACCTAAGGCATTCATTGACCCACGTCGCCCTGTGCACCCACAGCCTGACCACCGGGAAGAGGGTCTGATCCCTTATATTCCGGAACTGCCTGTTGCATCAGAGGGTATCATCAATTACAATCAAAGTGTTTTCAATATTAGAGGAATTCATACTGCACCTACGAGCCTTGAGTCTACATGTCTGGTGTTTGCATATGGTTTAGATCTGTTCTACACACGTGTTACTCCATCAAAAACCTTTGACATTCTGAAAGACGATTTTGATCATTTACTGATTTCTGCCGTACTCATCGCCCTATTACTCTTCTCTTATGCTGCAAGACGGTTAGCGGCTCGTAAGGCCCTACGTGCGGCTTGGAAATAA [2292]

Hespero GTACAGGCGACCATATACCGGATATCCGAAGCTAACGCCAAAACATCACCGCAAATATCGGCACCGTGGATACAGGCTTCAACCAGCTGTGCATTTGCAAGTAGTAGTTATTTTGTGTGCGCTGTACCGTCGTCTAAAACTCTACAATTGTTGTATCTAAGCAACGGAGTTAGTTTCGTGGCTGTACCGTTAAAGTTTTTGGGTCTACAAACGAATAACGCTAATGTAGAATTGAGGGTGAAATCTTTACCGTACCAAGAC---GGTTCAACTGCGCCTATGTTTGCGGTACATTTGGGTACCGACGGTATTTTATTAGGTCAAGTGGAAGCCTCGGGGGTGAAGGCGATCAAAGTATTGCCTAATGCCGTTGATATTTCGACTAGTTTGATCAGTAATAAATTAACAGTGTTCGCAGTGTTGAAAACGGAAAACCAATATAAGATATTGGCTATAGATCCGGTAGAC------------GGTCAAGAAATAGCCGAGTTTGGAGGTTCTTTCGTACTGGAAGAGGAACACGGCAAAATTGAATCGACTCACGTTTTGCCATACTCAAAGAAAGATAATTCTCTGAGTTATAAAATCTTGGTTATAACGGAAGATCACGCTCTAACGCTGTACCATCAACACGGCAAAATACTGTGGACCCGAGAAGAGTCGTTGGCTTCGATCGTAGCGACCGAGTTTATCGATTTGCCGTTGTCCGAAACGGATGCTAAAATAGAAGAAGAATTCGGAGCGTCGGACGCGAACAACGCGATTAATATGCTAATGACGCGATTGACCGCTCAATTAATACAATTGCAATCTTTTTTCCTCAAAACAATGTCCAGCATTCAAGGG------GACATTGAG------ACGGAAGATGGGACTGCCAATAATTTGGTCAGGGATAGATTCGGATTGCATAAGCTGATCGTTGCCGCTACAAGCTCGGGAAAAGTGTTCGGTATGGACAGTATGAGCGGTAATATCATATGGAGTAAATTCATCCCGGATCTGAATCCGATAATGGGCAAT---AATAAAGCTCAATTTCAATTGTACGTTCAACGCACTACCGCTCATTACCCTCATCAACCGGTGTGCACCGTGGTCGGAAAA---TCCGGATCCAAGCAGAGTTATTTGCACAGCTTGAATCCGATAACGGGAAATTTTATAAGTTCTAGAAAATTCCCATTCGTGGCCATTCAGAATATGATGCTGTCCGATATGGATAAGAACCACATGAGAGGACTAGTATTGGTCGATCCCCAGCTCAATGTTTACGTTTATCCTGTCGAAAGCGAAGAAATTCTCCTCAAATCCAAGAACAATCATTTCATTATTTTGGCCAATACTAATACAGCCCTATTGGGAGGATACAGTTTTGCATTATCAAAACCAAATGAATTAAAAGCAACTGAAGTTTGGCGAATCTCTCTACCGGTAGAACAACAAGAAATTTCTCAAATTAAACTAAAACGATTTAACGAACACGTTCACTCTCAAGGTCGCGTAATGGGCGATCGTAGTGTGCTATACAAATACTTGAATCCGAATCTAGCCGTCGTTGTAACGGAAGGCAGCGATCCATCAACCAAATATTTG---CTAAATATCTTCTTGATCGATGGCGTAACGGGCTTAATAGTGTATGCGTCCACTCATAAGAGAGCCAGAGGACCAGTTCACGTTGTGCATACCGAGAATTGGATTGTGTATACGTACTACAATGAGAAATCCCGTCGTATGGAATTATCCTCTATCGAATTATACGAGGGCAAAAGTCAAAGCAATACAACCGCATTCTCTTCGCTAAGCCCACCCGGTTTGCCCCTTGTGGAACATCAAAGCTTTATATTGCCAACTGCAGTAGAGGCTATGACCGACACTGTCACCGAGAAAGGAATTGCTAATAAACATATTTTACTGGCGCTACCATTAGGCGGAATTGTGGAACTACCCAAAGCTTTTGTGGACCCTCGCCGGCCTATTCACCCCTTACCGGAGCACAGAGAAGAAGGCCTTATACCGTACATACCGGAATTGCCGATTGCATCCGAAGGAATTATCAATTACAATCAAAGCGTTTTTAACATAAGAAATATTCATACAGCACCGGCCGGATTAGAATCGACTTGTCTCGTTTTCGTATACGGATTAGATTTGTTCTACACTCGAGTGACTCCGTCTAAAACCTTCGATATTCTGAAAGACGATTTCGATCATTTGCTAATTTCTGCCGTTTTAGTGGCGCTATTAGTTTTCTCTTACGCCACTAGGAGACTAGCGGCCAGGAAAGCACTCAGAGCAGCCTGGAAATAG [2292]

Trogulus GTACAGGCAACTGTATATAGTATTTCAGAGGCCATGGCTAAGACTTCGCCACAGATTTCAGCTCCATGGATACAGGAGTCTACCAGTTGTGCCTTTGCCAGCAGTAGTTACTTTGTGTGCATCGTCCAGTCATCTAAAACACTGCAGCTCCTCTACTTGAATAGTGGAGTCAGCTTTGTTGCTGTTCCCCTTAAGTTCCTAGGACTGCAGACAAGCATAATGAGTTCTCAACTGAAGGTAAAATCACTCCCTCATCAGGAT---AGCTCAACGCAACCAATGTTTAGTGTTAATCTAGGCAATGATGGAATGCTCCTTGGAAAGGTTGAAGCCTCTGGAGTCAAAGTGATCAAAGTTCTGCCAAATGCTGTTGACATCAGCACTTGTATTTTCAGCGGAAAGCTGATGGTCATCGCCATTCTGAAAATTGAACAGCATTACAAGACTGTTGTATTTGACCCAGAAAGT------------GGTAGCGAAATACAAGAACTTGGTGGAACTTTTACACTCTCTGAAGATCATGGTGCTATTGAGAATTTTCTTGTGTTGCCTTATCTGAGGAAAGATAATTCAGTTAGCTACAAAATTCTCTTGAACACGGAAGACAGTGCAATCAGTCTTTTTCATCAACAAGGCAAACTATTATGGACTCGAGAAGAAGCCTTAGCTTCTATCATTGCCACAGAAATGCTAGAACTTCCCTTATCAGAAACAGATGCTAAGATTGAAGAAGAATTTGGATTGGTTGATGCC---AATGCTTTAAGCATGTTGTTGACAAGGTTGACTACACAGCTTGGCCAAATGCAGAGTTTCTTCCTAAAGACACTGGCTGGGCTGCAGGAT------CATCAAAAT------CGAGAACTTCTTTCTACAAATAACCTTGTGCGTGATAAGTTTGGTCTTCACAAGGTCATAGTAGCAGCCACCCAATCTGGGAAGGTTTTTGGAATAGATAGTCTGGATGGACAGATCATCTGGAAGAAATTAATTCCAAACTTGTCTACAATGGGCTCCAGC---CAGTCTCCTTTGTACTTCCTCTTTGTGCAGCGTACTACAGCTCATTTCCCATATCAACCTGTCTGCACTATTTTGGGAAAACAGAAGGGATTAGAGGAGAGTTACATTCACTCGTTCAACCCAATTACTGGTTCAGATGTGGGATCGTCAAAGCTGCCTTTTAGGAGCAGTAAAGCCGTAATGCTGATCGACGTGGATAGTACATACATGAAAGGAATTATTATCATGGATTCAAACAAAATGGTTCATTTATATCCAGCTGAATACGAAGAAACATTTCTGAAATTGAAAGGCTCTTATTATGTAATGTTGGCTGATCCCGCTCTGGGAACTGTGACAGGATACAGTTTTGCACTTTCTCTTCCTGGTGCTCTGAAAGCTACCGAGGTCTGGCGAATCTCACTTCCTCTTGACCAGCAAATCATCACCCAAGTTCACCTGAAGAAAACCAATGAACATGTGCATTCACAAGGTCGGGTCATGGGTGACCGCAGTGTTCTATACAAGTATTTGAATCCCAATCTTGCTGTCATTGTAACTGAGGGAATGGATCCAGTTAATAAATATTTG---CTAAACGTTTACCTAGTGGATGCAGTTACGGGTTTGCTGCTTTATGCATCTTCTCATAAACGCTCAAGAGGACCTGTTCATGTTGTACATTCTGAAAATTGGATTGTGTATTCCCTCTACAATGAAAAGTCTCGTCGTGTGGAATTGTCCTCCATCGAACTCTATGAGGGCAAGAGCCAGAGTAATACGACTGCATTTTCATCACTTAGTCCACCAGCCTTACCTTTGGTGGAGCATCAAAACTTCATTTTCCCAGCTGGAATTGAAGCCATGACACAAACACTGACAGAAAAAGGAATTACTAATAAACATATTCTTATTGCTCTTCCTTTGGGTGGCATTGTGGAATTGCCTAAGGCATTCGTAGATCCACGTCGACCAGTGCACCCCCAACCTGAACACAGGGAAGAAGGGTTGATTCCCTATCTGCCAGAATTGCCTGTTGCCTCTGAAGGTATCATAAACTACAATCAAAGCGCCTATAACATCAGGAGTATATACACAGCACCCACAGGACTTGAATCAACTTGCCTTGTCTTTGCCTATGGTTTAGATCTATTCTTTACTCGGGTCACACCATCTAAGACGTTTGACATCTTGAAAGATGACTTTGATCATTTGCTCATTTCTGCTGTGCTCATTGCACTCATGCTGTTTTCTTATGGAACTAGAAGACTTGCATCCCGTAAAGCCCTGCGAACTGCATGGAAATAA [2292]

Leiobunum GTACAGGTCACAGTTTTTAGAATATTGGAAGGAAATGCAAAAACTTCACCACAAATATCCGCACCTTGGATACAAAGTCGCACAAGTTGTGATTTTGCCAGTGGTACATATTTTGTTTGTGCTGTACCATCAACTAAATCTTTACAAGTTTTGAACTTAAACAATGGAATTGGTTTTGTTGAAGTTCCGCTTAAGCTTCTGGGGCTACAAACGAACAAACCTAATGCTGAACTAAAAGTGCAATCAATCCCCTATCAAGAT---AGCTCCGATGAACCTGTTTTTGCCCTTCATTTAGGAGATGATGGCATATTTTTGGGGCGTATAGAAACATCAGGCATGAAAATTGTCAAAGTTCTACCAAAAGCTTTGGAAGTCAGCTCTTCTGTTATTAAAGGACAATTTACTATATTTGCCGTAATTAATGAGAAAAACAATTTCAAAGTTATAGCTCTAAGTCCAACAGAT------------GGAAAAGAAATTGAAAATCTGTCTGGTGAATTTTATTTGCCTCCCGACCATGGCTCAATTGAAAAGTTTTTTGTACTACCATACTTGAGAAAAGATGGTTCAATGAGCTATAAAATGCTTCTTGGTACTAGTGATGATGCAATAATATTAATTCACCAACAAGGCAAAGTTATTTGGACGAGGGAAGAATCTTTGGCTTCTATAATTACTACCGAAATTTGGGACTTGCCCTTATCGGATATGGATGTTAAAATTGAAGAAGAATTCGGAGCTTCCGATACG---AACGTATTCGCGATGCTACTGACAAGATTGTCTTCGCAACTTTTACAGTTACAGACTTTTCTATTAAAAACTTTATCGCGTTTACAAGGA------GAAGCTGAAACA---CAAGTTACTGGTTTTGGAAATGACTTGGTTAGGGATAAATTTGGTTTGCATAAAGTTATCGTTGCTGCTACTAAACCTGGAAAGGTGTTTGGAATCGACAACTTGTCCGGAGATATAATATGGAAGAAACTCATCCCA---ATATCACCCCTCAGTACTAAC---AACAAACAGTTTTATCATTTGTTTGTGCAACGAACAACAGCTCAATTTCCCCACCCAGCAATTTGCACAATTCTCGGGAAAACAAAGGATGAAGATGTGACTTACCTTCATTCCCTGAATCCCATAACTGGAAGTGATGTAACTACAAAGAAATATGATTTTGAAGTAATCCAAGTAATGATGTTATCCGAAATGGATGATAATCACATTAAAGGCCTTGTTCTTTTAGACAAAAACTTAAAGGCACATGTTTTTCCACAATCGGCCGAAAAAATATTCCTGCAAACAAAATCAAGTCATTACATATTTTTAGCAAACACTACAAGTGGTATTTTATCAGGATATACATTTATGGCCTCAAAACCAGGGTCTCTAGAAGCAACGGAAGTATGGAGAGTATCAATTCCAACTGAGTATCAACAAATTACTCAAGTTGTGTTGAAGAGAATTACAGAACATGTGCATTCACAGGGACGCGTCATGGGTGACAGAAGTGTTCTCTATAAATACTTAAATCCGAATTTAGCTGTCATAGTTACAGAGGGAAGTGACCCTGTTCAAAAATATTGT---TTGAACATTTATCTTTTGGATGGAGTAACTGGCATGATAATTTACTCTTCAAGTCATAAGAGATCTCGCGGACCAGTTCATATTGTACACTCTGAAAATTGGATTGTGTATAGCTACTACAATGAAAAATCGCGCCGAATGGAAATGTCTTCCATCGAACTATATGAAGGAAAAAGTCAGGGCAATACCAGCGCTTTCTCATCATTAAGTCCTCCGCCCATCCCGATTGTAGAGCACCAATCTTTCATATTTCCAGCTGGTGTTGAGTCCATGACTGACACAGTCACTGAAAAAGGAATAACCAACAAGCATATTTTAATTGCTCTACCATTGGGAGGAATTGTGGAGTTACCCAAAGCATTTTTGGACCCAAGAAGACCGATACAACCACTCGCAGAACACCGGGAGGAGGGTTTGATACCTTACATTCCCGAACTACCGATTACATCCGAAGGAATTATTAATTATAACCAAAGTGTTATGCAAATTAGAGGAATTCACACAGCACCATCCGGATTAGAATCTACATGTCTGGTTTTTGCATATGGCTTAGATATTTATTACACTAGAGTGACCCCATCAAAAACATTTGATATTTTAAAAGATGACTTTGATCATTTGCTTATCTCGGCAGTTTTAATTGCTCTACTGTTATTGTCATACATGACGAGAAAATTAGCAGCAAGAAAAGCTCTAAGAGCTGCTTGGAAATAA [2292]

Proto GTCCAAGTTACGGTCTTTAGAATTTTAGAAGGAAATGCCAAAACTTCACCTCAAATATCAGCTCCTTGGATTGAAAGCCATACGAGCTGTGATTTTGCCAGTGGTACATATTTTGTTTGTGCTGTACCATCCACAAAATCATTACAAGTTCTAAACTTAAACAATGGAATTGGTTTTGTTGAAGTTCCACTTAGGCTTCTGGGATTGCAAACTAATAAGCCAAATACTGAACTGAAAATACAATCTATCCCTTATCAAGAC---AGCTCTGATGAGCCAGTGTTTGCCCTTCATTTAGGAGATGATGGCATATTTTTGGGACGCGTTGAAGTATCTGGCATGAAAATTGTCAAAGTTCTACCAAAAGCTTTGCAAGTGAGCTCATCTATTATTAAAGGACAATTAACTCTATTT????????????GAGAAAAATAATTTTAAAGTTATGGGTCTAAATCCGACTGAT------------GGAAAAGAAGTTGAAAACCTAGGTGGTGAATTTTATTTACCTCCGGATCATGGCTCAATTGCAAAGTTTTTCATATTACCATACTTGAGGAAAGATAGCTCAATGAGTTATAAAATGCTTGTATCAACTAGTGATGATGCTATACTATTAATTCACCAACAAGGTAAAGTCATTTGGACTAGGGAAGAATCTTTGGCTTCCATACTAACTACAGAAATTTGGGATTTACCTTTATCGGATATGGATGTTAAAATTGAAGAAGAATTTGGAGCTCCAGATACT---AATGTTTTCGCAATGTTACTAACGAGATTGTCTACCCAACTTCTGCAACTGCAAACTTTTCTATTAAAAACTGTATCGCGATTGCAAGGT------GAAGCTGAAACGTCTAAAGTAACTGGTTTTGGAAATGATTTGGTCAGGGATAAATTTGGTTTACATAAAGTGATAGTTGCCGCTACTAAACCTGGAAAGGTTTTTGGAATTGATAATCTGTCAGGGGAAATAATATGGAAAAAACTAATCCCG---ATTGCACCTCTTAACGCTAAT---AACAAACAGTTTTATCATTTATTTGTGCAAAGAACAACCGCTCAATTTCCCCACCCAGCCATTTGCACTATTCTTGGCAAAGGACAAAATGAAGATGTGACATACCTTCACTCTCTGAACCCGATAACTGGGAGTGATGTAACTGCGAAGAAATATGATTTTGAAGTAATCCAAGTGATGATGTTATCCGAAATGGACGATAATCACATTAAAGGCCTAGTTCTTTTAGACAGAAACTTGAAGGCACATGTGTTTCCGCAATCAGCCGAAAAAATATTCCTGGAAACAAAATCAAGTCATTACATATTTTTAGCAAACACTACGAGTGGTATTTTATCAGGATATACATTTATGGCTTCGAATTATGGGTCCTTGGAGGCAACGGAAGTATGGAGGGTATCAATCCCAACAGAGTATCAACAGATAACCCAAGTTGTGTTGAAGAGAGTTACTGAACATGTGCATTCTCAGGGACGTGTAATGGGTGATCGAAGCGTTCTCTATAAATACCTAAACCCTAATTTGGCTGTTATAGTTACAGAGGGAAGTGACCCAGTTCAAAAATATTGC---TTGAACCTTTATCTTTTGGATGGGGTAACTGGAATGATAATTTACTCCTCAAGCCATAAAAGGTCCCGCGGACCAGTTCATGTTGTACATTCTGAAAATTGGATTGTGTATAGCTACTACAATGAAAAATCTCGCCGTATGGAAATGTCTTCCATTGAATTGTACGAAGGAAAAAGTCAGGGCAATACCAGTGCCTTCTCCTCCCTGAGTCCTCCCGCCATTCCCATAGTGGAACACCAATCTTTCATATTCCCCGCTGGTGTTGAAGCAATGACCGACACAGTTACCGAAAAAGGAATTACCAATAAGCATATTCTAATTGCTCTACCCTTGGGAGGAATTGTGGAGTTACCCAAGGCATTTTTGGACCCTCGAAGACCGATACAACCTCTTGCTGAACACCGAGAAGAGGGTTTAATACCTTATATTCCCGAATTACCAATTACATCAGAGGGAATTATCAATTACAATCAAAGCGTTATGCAAATTAATGGAATTCACACAGCACCATCGGGATTGGAATCTACGTGTTTAGTTTTTGCGTATGGCTTAGATCTCTATTACACCAGAGTGACACCATCAAAGACTTTTGATATCTTAAAGGATGACTTTGATCATTTACTAATCTCGGCAGTTTTAATTGCTTTACTGTTATTATCGTACATGACAAGGAAATTAGCAGCGAGAAAAGCTCTAAGAGCGGCTTGGAAATAA [2295]

[TICKFAST_NCHAR=438;hypothetical protein XM_002409307]

Ixodes TTCCCTATCGCCAAGCTCGCCGCGCTCGCCTTCCGGCAGCTGAGCAAGCCGCTCGCGAACCGCATCAAAGCCGGCGCCAAAAAGAGCCCCTTCTTCCGAACCTACGTGTGCATGCCGCCGGCCCAGATCTACCACTGGATCGAGGTGAATGTCAAGATGCGCATCCTCAACCTGGGCAGACCCACCGACGTGCCCAAGCTCAACGAAGCCATGGCCATCGAACTGGGCTCCGAACTCCTCGGCGAAGTCATCATCTTCACGTCCGCCGCCGCCACCCTGGTCGCCGAGTACGTACGGCAAGCGCGCAACGAGCGCCTCAAGGAGCAGGCCAAGGAAGACAAGCACTGCTGCCTCGAGCGCGAGGTGGAGGAGCTCCGCTTCCTCGCAGAGATGCACGAGGCCCAGATAAGGCACCTGTCCAGGCTCGTAGGTGACAAG [438]

Siro TTTCCCTTAATGAAGTTAAGTATTTTAGTAATTAAACAATTCGCGAAACCTGTGGCGAATTACTTTATAAAAAAAGCGAAGAAAAATTATGTCTTGAGAACTTACCTAGTGATGCCGTTTGGTCAATTTGTTCACAAAATTCAACTTAAAGCGAAGATGCGTGACCTCGACTTGGGAAAACCAACTGAAGTGAAGCAGATGAGTGAAAATATGGCCGTTGAACTAGGGGCAGATGTTCTGGTAGATGTGTTCGTCTCTGGAGCAGTGATCGGCCTAACTGCCTGGTATTATGGCTACGATAGGAAGCATACAGCAATAAAAGAAGCAGCCAGAAATGAGCGAACAGAATGTTTGGAGAACCTTGTGATAGAGCAAAAGCACCAGTGTATTGAACTCGAAACACGTGTAAAGGAACTCGAGATAATGGTTTGTGCAAAA [438]

Sitalc TTTCCTATCATCAAGTTAGGAGCACTAGCTATTCGTCAGCTGGCCAAACCTCTTGCGAATCGCATCAAGTTGAGTGCAAAAGCTAGCCCCTTCTTTCGAACCTACATATGTATGCCACCAGCTCAACTGTACCACTGGATAGAGGTCAACGTTAAAATGAGAATGATGAACCTGGGAAAACCAACAGATGTTCCGAAACTAAACGAAAACATGGCCATCGAGCTGGGCGCAGAGCTGTTGGGCGAAAGCATCATCTTCGGCGTTGCCGTCTTCACAATGACGGCCGAATACATCCGATCTTCCAACAAAGAAAAGGAGAAGGAAGCAGCTAAAGAAGCTCGCTTCAGCCAACTCGAGACGACCGTCAACGAATTGAAGTTCACAGTGATACACCAGGAGGCTGAGATTAGACATTTGACGAGGGTGGCGCACGAACGA [438]

Sclero TTTCCAATCGTCAAGTTAGGTGCTTTAGCTCTTCGCCAGCTCTCCAAACCTCTGGCAAATCGAATCAAATCACGGGCGAAAGCTAGTCCATTTTTTCGAACCTACATATGTATGCCACCAGCTCAGCTGTACCACTGGATCGAAGTGAACGTGAAAATGCGATTACTGAACTTAGGCAAGCCGACGGAAGTGCCAAAATTGAACGAGAACATGGCTATTGAATTAGGAGCCGAACTCTTAGGGGAGAGCATTATCTTCGGCACGGCCGTGTTTACGCTCGTAGCGGAATACATCCGGTCTTCCCTCAAAGAGAAGGAGAAAGAAGTCGCCAAAGAAGAACGCTTCCGAGTACTCGAAGAGAACGTGCAAGAGTTGAGGTTCACCATTATTCACCAGGAAGCGGAGATTCGACATCTGACGCAAGTGGCGCACCGGAAA [438]

Trogulus TTTCCCATCGTTAAATTAGGAGCTTTAGCTATTAAACAGTTTAGTAAACCCATAGCGAACCGTATCAAAGCCAGTGCAAAATCGAGTCATTTTTTCCGGACCTACATTTGCATGCCTCCAGCCCAGTTTTACCACTGGGCGGAGGTGAATGTGAAGATGCGGTTGCTGAACCTCGGTAAGCCGACGGATGTGCCCAAGTTGAATGAAAACATGGCTATCGAACTGGGCGCAGAGTTGCTGGGTGAAGGAATCATATTCTTAATAGCAGCTACCACACTGACAGCCGAATATATCCGATCGTCGCGTAAAGAGCAAGAAAAAGAAGCGGTTAAAGAGGCTCGCTTTAAAGAACTCGAGAACATGGTCTCGGAACTGCAGTTAACTGCCATGCAACAGGAAGCCGAACTCCGGCACATGGAAAGACTAGTTCATAACAAG [438]

Ortho TTCCCCATAGTTAAACTTGGAGCATTAGCTATAAAACAGTTAAGTAAACCCTTGGCGAACCGGATAAAATCAAGTGCAAAATCAAGCCATTTCTTCCGAACCTACATATGCATGCCTCCTGCTCAGTTTTACCATTGGGTTGAGGTAAATGTGAAGATGAGGATGCTGAATTTGGGAAAACCAACTGATGTGCCTAAACTTAATGAAAATATGGCAATTGAACTGGGAGCTGAACTTCTAGGAGAAGGGATTATATTCTTTACAGCTGCAGGCACTCTCACAGCAGAATATATACGCTCGTCAAGAAAAGAACAAGAGAAAGAGGCTGTCAAAGAAGCTCGCTTTGAAGAACTAGAAAATATGGTCTCAGAGCTGCAGTTAACTGCAATACAACAAGAGGCAGAATTAAGGCATATGGAAAGGTTAATTCATAACAAA [438]
[truncated: 1,922,479 more chars]
