# Supplementary material for: Phylogenomic Resolution of Paleozoic Divergences in Harvestmen (Arachnida, Opiliones) via Analysis of Next-Generation Transcriptome Data
Source: PLoS One. 2012 Aug 24;7(8):e42888. doi: 10.1371/journal.pone.0042888 (PMC3427324; doi:10.1371/journal.pone.0042888)
Supplement: Text S2 — Results from gene ontology analysis. (DOCX) [file pone.0042888.s005.docx]

**Text S2**

**Methods -** Representative nucleotide sequences (usually *Ixodes*, scorpion for 9 SCORP matrices where *Ixodes* was unavailable) for four separate matrices (SCORP, TICKFAST, TICKMED, TICKSLOW) were imported into Blast2GO V.2.5.0 (<http://www.blast2go.com> [72]) for bioinformatic functional annotation. Sequences were compared to the NCBI nr database using blastx and QBlast (max e value 1e-15), saving a single best hit per query. Four default mappings were run on Blast results, and these mappings were subsequently annotated using default settings. A reduced set of gene ontology (GO) terms was produced using GOSlim, using the generic GOSlim online database. Directed acyclic graphs were produced using default settings for both biological process and molecular function [1].

**Results -** Although there is variation in the relative proportion of GO terms among matrices, all matrices share the same set of common GO terms. Shared molecular function annotations include binding (nucleic acid, protein and nucleotide binding), catalytic activity (hydrolase and transferase), and structural molecule activity. Shared biological process annotations include metabolic process (biosynthetic, cellular metabolic, macromolecule metabolic, etc), biological regulation, and localization.

References

1. Ashburner M, Ball CA, Blake JA, Botstein D, Butler H, et al. (2000) Gene Ontology: tool for the unification of biology. Nat Genetics 25: 25-29.
