# Supplementary material for: Phylogenomic Resolution of Paleozoic Divergences in Harvestmen (Arachnida, Opiliones) via Analysis of Next-Generation Transcriptome Data
Source: PLoS One. 2012 Aug 24;7(8):e42888. doi: 10.1371/journal.pone.0042888 (PMC3427324; doi:10.1371/journal.pone.0042888)
Supplement: Text S3 — Results of alternative clock analyses. (DOCX) [file pone.0042888.s006.docx]

**Text S3**

Calibration 1 (no sequence data, auto-correlated rates)

(Siro: 4.422993, ((Sitalc: 2.550676, Sclero: 2.550676) '0.176-4.632': 1.534257, ((Proto: 2.542816, Leiobunum: 2.542816) '1.622-3.959': 1.182841, ((Ortho: 1.752611, Trogulus: 1.752611) '0.983-3.378': 0.943323, Hespero: 2.695934) '1.307-4.261': 1.029723) '2.898-4.832': 0.359276) '3.104-5.016': 0.338060) '3.281-5.211';

Calibration 2 (no sequence data, auto-correlated rates)

(Siro: 4.559285, ((Sitalc: 3.089499, Sclero: 3.089499) '0.158-4.916': 1.312589, ((Proto: 3.799514, Leiobunum: 3.799514) '2.901-4.938': 0.436036, ((Ortho: 1.845583, Trogulus: 1.845583) '0.988-3.558': 1.905659, Hespero: 3.751241) '2.898-4.857': 0.484309) '3.298-5.049': 0.166538) '3.427-5.141': 0.157196) '3.504-5.258';

Calibration 3 (no sequence data, auto-correlated rates)

(Siro: 4.742820, ((Sitalc: 2.764524, Sclero: 2.764524) '0.155-4.846': 1.781130, ((Proto: 2.724441, Leiobunum: 2.724441) '1.635-4.255': 1.603586, ((Ortho: 1.816716, Trogulus: 1.816716) '0.988-3.720': 1.126340, Hespero: 2.943057) '1.322-4.538': 1.384971) '3.686-4.993': 0.217626) '3.889-5.143': 0.197166) '4.049-5.307';

------------

[Calibration 1, run1, independent rates]

(Siro: 5.076774, ((Sitalc: 2.276032, Sclero: 2.276032) '1.951-2.682': 2.730449, ((Proto: 0.593026, Leiobunum: 0.593026) '0.495-0.724': 3.170610, ((Ortho: 1.554963, Trogulus: 1.554963) '1.332-1.827': 1.427490, Hespero: 2.982453) '2.606-3.440': 0.781184) '3.299-4.322': 1.242844) '4.404-5.739': 0.070294) '4.474-5.810';

[Calibration 1, run 2, independent rates]

(Siro: 5.078455, ((Sitalc: 2.277466, Sclero: 2.277466) '1.956-2.674': 2.730328, ((Proto: 0.594922, Leiobunum: 0.594922) '0.497-0.721': 3.171104, ((Ortho: 1.560603, Trogulus: 1.560603) '1.334-1.837': 1.423441, Hespero: 2.984044) '2.603-3.440': 0.781982) '3.300-4.324': 1.241767) '4.402-5.735': 0.070662) '4.477-5.807';

[Calibration 1, no data, independent rates]

(Siro: 4.434750, ((Sitalc: 2.511107, Sclero: 2.511107) '0.159-4.597': 1.580010, ((Proto: 2.534963, Leiobunum: 2.534963) '1.623-3.960': 1.193065, ((Ortho: 1.733786, Trogulus: 1.733786) '0.985-3.322': 0.935151, Hespero: 2.668938) '1.305-4.247': 1.059091) '2.901-4.827': 0.363089) '3.113-5.007': 0.343633) '3.299-5.212';

---------

[Calibration 2, run 1, independent rates]

(Siro: 5.224562, ((Sitalc: 2.362811, Sclero: 2.362811) '2.055-2.803': 2.791946, ((Proto: 0.697844, Leiobunum: 0.697844) '0.525-2.051': 3.218333, ((Ortho: 1.618349, Trogulus: 1.618349) '1.405-1.900': 1.510339, Hespero: 3.128688) '2.817-3.700': 0.787489) '3.549-4.500': 1.238580) '4.663-5.867': 0.069804) '4.753-5.944';

[Calibration 2, run 2, independent rates]

(Siro: 5.232494, ((Sitalc: 2.357156, Sclero: 2.357156) '2.076-2.752': 2.810237, ((Proto: 0.617136, Leiobunum: 0.617136) '0.523-0.757': 3.279539, ((Ortho: 1.615830, Trogulus: 1.615830) '1.418-1.883': 1.482282, Hespero: 3.098112) '2.812-3.533': 0.798562) '3.543-4.436': 1.270718) '4.709-5.887': 0.065101) '4.780-5.958';

[Calibration 2, no data, independent rates]

(Siro: 4.582649, ((Sitalc: 3.111233, Sclero: 3.111233) '0.162-4.904': 1.294698, ((Proto: 3.732680, Leiobunum: 3.732680) '2.882-4.844': 0.477772, ((Ortho: 1.852695, Trogulus: 1.852695) '0.989-3.559': 1.898074, Hespero: 3.750769) '2.895-4.842': 0.459683) '3.296-5.025': 0.195479) '3.455-5.141': 0.176718) '3.563-5.269';

------------

[Calibration 3, run 1, independent rates]

(Siro: 5.395779, ((Sitalc: 2.420295, Sclero: 2.420295) '2.106-2.818': 2.910947, ((Proto: 0.638156, Leiobunum: 0.638156) '0.530-0.798': 3.388409, ((Ortho: 1.664192, Trogulus: 1.664192) '1.440-1.955': 1.527209, Hespero: 3.191402) '2.840-3.649': 0.835163) '3.606-4.585': 1.304677) '4.788-6.080': 0.064537) '4.859-6.154';

[Calibration 3, run 2, independent rates]

(Siro: 5.400593, ((Sitalc: 2.422757, Sclero: 2.422757) '2.105-2.822': 2.912946, ((Proto: 0.634316, Leiobunum: 0.634316) '0.534-0.765': 3.393197, ((Ortho: 1.666032, Trogulus: 1.666032) '1.443-1.948': 1.524724, Hespero: 3.190756) '2.841-3.655': 0.836756) '3.607-4.596': 1.308190) '4.789-6.097': 0.064890) '4.858-6.172';

[Calibration 3, no data, independent rates]

(Siro: 4.741987, ((Sitalc: 2.782597, Sclero: 2.782597) '0.153-4.859': 1.761180, ((Proto: 2.737736, Leiobunum: 2.737736) '1.640-4.279': 1.589956, ((Ortho: 1.818148, Trogulus: 1.818148) '0.988-3.739': 1.142825, Hespero: 2.960973) '1.324-4.547': 1.366720) '3.688-5.006': 0.216084) '3.888-5.140': 0.198210) '4.044-5.305';
